# Intraspecific Versus Interspecific Scaling of Metabolic Rate: Tests of the Metabolic Theory of Ecology Across Biological Hierarchies

**DOI:** 10.3390/biology15010084

**Published:** 2025-12-31

**Authors:** Jiayin Wang, Lei Zhao

**Affiliations:** Beijing Key Laboratory of Biodiversity and Organic Farming, College of Resources and Environmental Sciences, China Agricultural University, Beijing 100193, China; jiayinwang@cau.edu.cn

**Keywords:** Metabolic Theory of Ecology, body size scaling, activation energy, intraspecific variation, hierarchical scaling

## Abstract

The Metabolic Theory of Ecology (MTE) proposes universal constants for the scaling of metabolic rate (*BMR*) with body mass and temperature. However, the validity of these constants across different biological hierarchies, specifically within versus between species, remains debated. Using a comprehensive dataset of 3767 metabolic measurements across 174 species, we tested for systematic differences in scaling relationships. We employed a unified linear mixed-effects model to estimate intraspecific parameters and phylogenetic generalized least squares (PGLS) regression for interspecific comparisons. Our results reveal a decoupling of scaling parameters across hierarchical levels. The overall intraspecific mass-scaling exponent (*b* = 0.760 ± 0.012, fixed effect ± SE) was not significantly different from the phylogenetically corrected interspecific exponent (0.768 ± 0.023). In contrast, the overall intraspecific activation energy (*E* = 0.601 ± 0.016 eV) was significantly higher than the attenuated interspecific value (0.403 ± 0.073 eV). Taxonomic variation was prominent for mass-scaling, with fish exhibiting a significantly higher exponent than most other groups, whereas activation energy did not differ significantly among groups. We conclude that while the mass-scaling relationship converges through interspecific averaging, the sensitivity of metabolism to temperature is robust within species but becomes diluted in broad-scale comparisons. This demonstrates that metabolic scaling is inherently hierarchical, necessitating scale-explicit models rather than the pursuit of universal constants.

## Simple Summary

Why do a tiny insect and a large fish have such different energy needs? Scientists use a framework called the Metabolic Theory of Ecology to predict how an animal’s energy use relates to its body size and the temperature of its environment. A key question is whether these predictions work the same way for individuals within a species as they do for comparisons between different species. We analyzed metabolic rate data from 174 species, using statistical approaches that account for species differences and evolutionary relationships. We found that the effect of body size is highly variable within species but converges toward a consistent scaling pattern across species. In contrast, the influence of temperature is strong within a species, but appears weaker when comparing different species. This shows that the “rules” for energy use depend on the scale of observation. Our findings will help build better models to predict how species, from the smallest insects to the largest fish, will respond to environmental changes like global warming.

## 1. Introduction

Metabolism represents a fundamental process that bridges environmental energy flow and biological activity, serving as the cornerstone of energy conversion and material recycling in living systems [1,2]. The metabolic rate of an organism not only influences its growth, reproduction, and survival, but may in turn be affected by these biological processes, reflecting a bidirectional relationship between metabolism and organismal performance [3]. Metabolism thus exerts, and responds to, selective pressures that collectively shape the structure and function of ecosystems [4,5,6]. Recent empirical and molecular studies have highlighted this central role of metabolism in shaping physiological and ecological processes across diverse taxa. For instance, Sentís et al. (2025) demonstrated that invasive birds modulate their basal metabolic rate in response to ambient temperature, illustrating the ecological plasticity of metabolism [7]. Similarly, Riccieri et al. (2024) showed that genomic adaptations associated with metabolic pathways underlie life-history transitions and chemical defense mechanisms in blister beetles, linking metabolism to evolutionary innovation [8]. In ectothermic vertebrates, Warner et al. (2025) found that elevated nest temperatures did not alter residual yolk metabolism in hatchling turtles, indicating robust physiological regulation of energy use [9]. Collectively, these findings underscore that metabolism serves as a mechanistic bridge connecting individual physiology to ecological and evolutionary dynamics. The Metabolic Theory of Ecology (MTE) has provided a powerful, unifying framework for explaining biological patterns across scales by linking metabolic rate to body mass and temperature through a fundamental equation [10]:

(1) $$\mathrm{BMR}\;={\;\mathrm{BMR}}_{0}\cdot M^{b}\cdot e^{E(T-T_{0})/kTT_{0}}$$

Here, *BMR* is the basal metabolic rate, *M* is body mass, *T* is absolute temperature, *b* is the scaling exponent, *E* is the activation energy, *k* is Boltzmann’s constant, and *T*_0_ is a reference temperature. Although the Metabolic Theory of Ecology (MTE) assumes additive effects of body size and temperature on metabolism, several studies have shown that these factors may interact in complex ways [6,11,12,13,14]. For instance, temperature may alter the biochemical efficiency or transport constraints that underpin mass-scaling relationships.

A key prediction of MTE is the prevalence of universal scaling constants, notably a 3/4-power law for the mass-scaling exponent (*b*) and an activation energy (*E*) of approximately 0.65 eV, reflecting constraints imposed by fractal resource distribution networks and thermodynamic biochemistry, respectively. This prediction regarding the scaling exponent traces back nearly a century. The seminal observation by Kleiber established that across species, basal metabolic rate (*BMR*) scales with body mass (*M*) approximately as 3/4, a pattern which suggested a surprising universality in biological design [15]. This ‘Kleiber’s law’ spurred decades of research aimed at deriving its mechanistic basis, most notably the resource-distribution network model proposed by West, Brown, and Enquist (WBE), which theoretically derived the three-quarter-power scaling exponent [16]. These foundational works culminated in the formalization of the Metabolic Theory of Ecology (MTE). However, the purported universality of these constants has been increasingly challenged by empirical evidence. Glazier, in a comprehensive review, argued compellingly that the scaling exponent is not invariant but varies systematically [11]. Observed scaling exponents frequently deviate from 3/4, ranging between 2/3 and 1, and exhibit considerable variability, particularly at the intraspecific level [4,13,17]. For example, Clarke provides a comprehensive assessment of MTE applied to animals, showing that scaling exponents and thermal sensitivities often deviate from the proposed constants, especially across different taxa and physiological types [1]. Comparative studies of endotherms (e.g., birds vs. mammals) reveal systematic differences in scaling slopes and intercepts related to taxon divergence time and metabolic strategies [18]. Mechanistic work further demonstrates that thermodynamic constraints and organismal physiology can lead to differing scaling relationships when analyzed within versus between species [19]. Empirical data for ectothermic vertebrates show that metabolic scaling within species responds to environmental temperature and activity, indicating that intraspecific scaling is modulated by ecological and physiological conditions [14].

This variability suggests that metabolic scaling may not be a fixed physiological constant but rather a statistical outcome influenced by biological scale, physiological plasticity, and evolutionary adaptation [3]. A significant conceptual issue is that MTE models are often parameterized using among species values, which may overlook intraspecific variation. This practice can lead to statistical artifacts, such as the compression of slopes in interspecific analyses, potentially obscuring the true relationship between metabolism, mass, and temperature within species [20]. In response, scaling relationships should be investigated independently across different biological levels, from within individuals to among species [3].

Furthermore, recent studies have indicated that the relationship between metabolic rate, body mass, and temperature is not only influenced by physiological factors but also by ecological context. Although MTE posits a universal scaling between these variables, recent research suggests that this relationship is modulated by ecological hierarchy and habitat stability. For example, Mähn et al. (2025) demonstrated that habitat stability can alter the temperature–metabolism relationship across ecosystems, potentially diluting the universality of metabolic scaling at macroecological scales [21]. This highlights the need for more nuanced models that integrate ecological factors alongside physiological principles, suggesting that metabolic scaling is not solely governed by thermodynamic constraints but also shaped by environmental factors and the ecological scale of observation.

Comparisons between intraspecific and interspecific metabolic scaling are not new, and several influential studies have demonstrated that scaling relationships can differ across biological hierarchies. Bokma (2004) showed that interspecific scaling exponents are statistically sensitive to sampling structure and need not reflect intraspecific relationships [22]. Glazier (2005) synthesized extensive empirical evidence indicating that both intra- and interspecific scaling exponents vary systematically with metabolic level, activity, and ecological context, a conclusion further reinforced by his later work emphasizing a paradigm shift away from universal scaling laws [11,17]. At a finer hierarchical scale, Norin and Gamperl (2018) provided compelling empirical evidence that metabolic scaling exponents differ among hierarchical levels within a single species, from within individuals to populations [20].

Despite these advances, most previous studies have focused primarily on body-mass scaling exponents and have rarely integrated temperature dependence into a unified hierarchical comparison. Moreover, intraspecific and interspecific scaling parameters have typically been estimated using different datasets or analytical frameworks, complicating direct quantitative comparison. Here, we extend this body of work by jointly analyzing mass and temperature scaling across hierarchical levels using a single, phylogenetically curated dataset and a unified statistical approach. This allows us to explicitly test whether mass scaling and thermal sensitivity behave similarly or diverge across biological hierarchies.

We specifically address the following questions:

Q1: Do the scaling exponents (*b*) relating body mass to metabolic rate differ systematically between intra- and interspecific analyses? We hypothesize that intraspecific exponents will show greater variance and may differ from the interspecific exponent due to narrower body size ranges and the influence of uncontrolled physiological and environmental factors.

Q2: Does the activation energy (*E*) relating temperature to metabolic rate differ between hierarchical levels? We hypothesize that interspecific averaging will attenuate the apparent temperature sensitivity, resulting in a lower interspecific activation energy compared to the mean intraspecific value.

Q3: Do body size and temperature have interactive effects on metabolic rate within and across species, and how do these effects compare? We test this by including a body-mass × temperature interaction term in both intraspecific and interspecific models to assess whether the effects of mass and temperature are additive or synergistic.

By addressing these questions, this study aims to provide a robust empirical test of metabolic theory and contribute to a more nuanced, scale-explicit understanding of metabolic scaling in ecology.

## 2. Materials and Methods

### 2.1. Data Compilation

We compiled a dataset from the literature, comprising 7335 entries on basal metabolic rate (*BMR*, in mL O_2_ h^−1^), body mass (*M*, in grams), and temperature (*T*, in Kelvin) across different species (see Supplementary Materials for the dataset). The dataset encompasses 1591 species, including unicellular organisms (prokaryotic and eukaryotic forms), invertebrates (insects, arachnids, malacostracans, myriapods, and clitellates), and ectothermic vertebrates (amphibians, reptiles, and fish). The body mass of these species spanned a wide range, from 10^−14^ g to 3.40 × 10^4^ g, while *BMR* ranged from 2.77 × 10^−15^ to 6.73 × 10^3^ mL O_2_ h^−1^.

To ensure data quality and suitability for phylogenetic and hierarchical analysis, we applied the following sequential screening criteria: (1) Records with species names listed as “Unknown” were removed (11 records excluded). (2) We selected species for which data were available for at least three distinct body mass values and at least three distinct temperature values. This criterion aimed to capture intraspecific variation along both key metabolic axes while maintaining robustness for within-species regression. In practice, most species were represented by far more data points (mean = 21.8, median = 10, range = 3~252 observations per species). Because the intraspecific parameters were estimated using a linear mixed-effects (LME) model that jointly fits all species while weighting by data availability, this threshold balances inclusivity with statistical robustness. This step yielded 186 species and 4061 records. All unicellular taxa were omitted due to insufficient intraspecific replication across both body mass and temperature. (3) To incorporate phylogenetic information, we matched the species names from the filtered list to the Open Tree Taxonomy (OTT) using the “rotl” R package (version 3.1.0 for rotl and version 4.4.0 for R). A total of 174 species were successfully matched, resulting in a final curated dataset of 3767 records. This phylogenetically informed dataset was used for all subsequent analyses.

### 2.2. Phylogenetic Tree Construction

For the 174 matched species, we constructed a phylogenetic tree using the function tol_induced_subtree from the “rotl” package, based on the Open Tree Taxonomy identifiers. This function retrieves the minimal subtree containing the specified species from the synthetic Open Tree of Life. The resulting tree was used to model the evolutionary non-independence among species in the interspecific analysis (see Section 2.4). The package “ggtree” (version 1.0.21) was used to show the phylogenetic tree.

### 2.3. Analysis of Intraspecific Scaling Parameters

To estimate the intraspecific scaling parameters while incorporating all available data, we employed a linear mixed-effects modeling framework. This approach jointly estimates the overall within-species scaling relationships (fixed effects) and the deviation of each species from these averages (random effects). A single linear mixed-effects model was fitted using the lme function in the “nlme” package (version 3.1-167). The model was as follows:

(2) $$log\left({BMR}_{ij}\right)=a+b\cdot log\left(M_{ij}\right)+E\cdot T_{ij}^{\prime }{+\alpha }_{i}+\beta_{i}\cdot log(M_{ij})+\gamma_{i}\cdot T_{ij}^{\prime }+\epsilon_{ij}$$

Here $T^{\prime }=(T-T_{0})/kTT_{0}$. The coefficients *a*, *b*, and *E* are the population-level fixed effects for the intercept, the mass-scaling exponent, and the activation energy, respectively. The coefficients *α_i_*, *β_i_*, and *γ_i_* are the random effects for the *i*th species, representing its deviation from the population-level intercept, mass-scaling exponent, and activation energy. *ϵ_ij_* is the residual error term. In this model, species was included as a random factor on the intercept, the mass-scaling exponent, and the activation energy. The species-specific parameters for the *i*th species were calculated as $b_{i}=b+\beta_{i}$ and $E_{i}=E+\gamma_{i}$.

### 2.4. Analysis of Interspecific Scaling Parameters

To obtain unbiased estimates of the interspecific allometric and thermal scaling parameters while controlling for shared evolutionary history, we performed a Phylogenetic Generalized Least Squares (PGLS) regression on species-level mean data. For each of the 174 species in the phylogenetically curated dataset, we calculated the mean values of log-transformed body mass (*logM_avg_*), mean values of transformed temperature term ($T_{avg}^{\prime }$), and mean log-transformed metabolic rate (*logBMR_avg_*). These species-level averages served as the data points for the interspecific analysis. A PGLS model was fitted to estimate the effects of body mass and temperature across species. The model was implemented using the gls function in the “nlme” R package (version 3.1-167 for nlme and version 4.4.0 for R), with a Brownian motion model of evolution specified via the *corBrownian*(*phy* = *tree*) correlation structure, where *tree* is the phylogenetic tree constructed in Section 2.2. The model formula was:

(3) $$log\left({BMR}_{avg}\right)=a_{0}+b_{0}\cdot log\left(M_{avg}\right)+E_{0}\cdot T_{avg}^{\prime }$$

Here the coefficients *b*_0_ and *E*_0_ are the phylogenetically corrected interspecific mass-scaling exponent and the phylogenetically corrected interspecific activation energy, respectively. In addition to the additive models, we fitted extended versions of both the LME and PGLS models that included an interaction term between body mass and temperature to examine whether body size and temperature jointly influence metabolic rate.

### 2.5. Statistical Analyses

The distributions of the species-specific parameters (*b_i_* or *E_i_*) obtained from the mixed-effects models were summarized. A one-sample *t*-test was used to compare the mean intraspecific coefficients (*b_i_* or *E_i_*) against the corresponding phylogenetically corrected interspecific estimate (*b*_0_ or *E*_0_) from the PGLS analysis. The final dataset consisted of 174 multicellular ectothermic species. Unicellular taxa were excluded from hierarchical analyses due to insufficient intraspecific replication across both body mass and temperature. For comparative analyses, species were assigned to five phylogenetically coherent groups: (1) Annelids (including Clitellata only); (2) Arthropods (Archinida, Malacostraca, Insecta, Collembola, Branchiopoda, and Hexanauplia); (3) Amphibians (including Amphibian only); (4) Reptiles (including Lepidosauria only); and (5) Fish (majorly including Actinopterygii and Chondrichthyes).

One-way ANOVA followed by Tukey’s post hoc test was employed to compare the intraspecific scaling exponents (*b*) or activation energies (*E*) among these groups. One-sample t-test was used to compare the intraspecific parameters for each group with the interspecific parameters. All statistical analyses and plotting were conducted in R (version 4.4.0).

## 3. Results

### 3.1. Phylogenetic Tree

**Figure 1:**
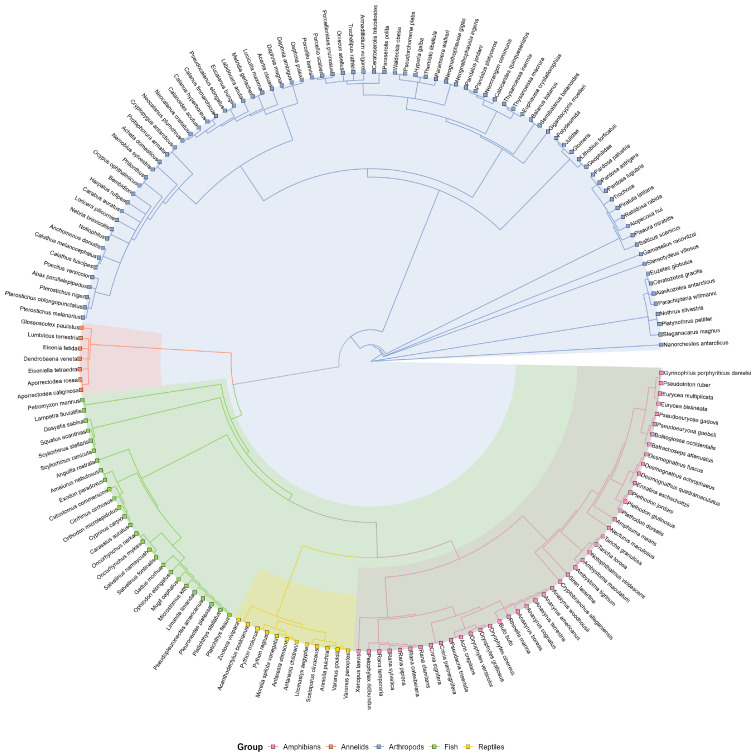
Phylogenetic tree of the 174 focal species. All species are divided into five categories: purple represents amphibians, red represents annelids, blue represents arthropods, green represents fish, and yellow represents reptiles.

Figure 1 presents a class-level taxonomic circular tree constructed for 174 focal species. This visualization classifies the species into six distinct taxonomic groups: micro-invertebrates, fish, macro-invertebrates, insects, amphibians, reptiles, and fish, which is consistent with our classification based on information retrieved and validated from the NCBI Taxonomy database.

### 3.2. Scaling Exponent (b): Intraspecific vs. Interspecific

**Figure 2:**
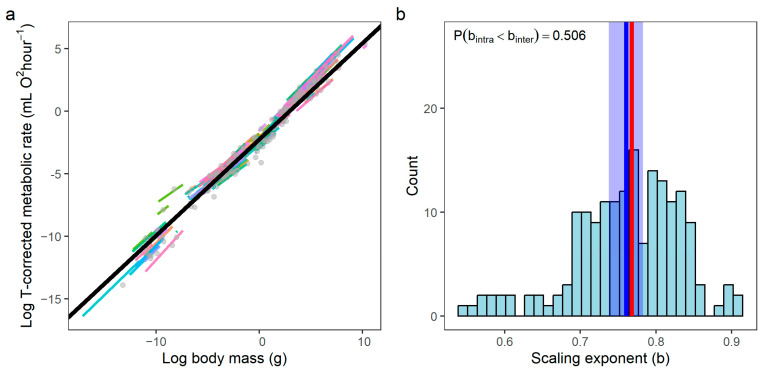
Scaling of metabolic rate with body mass. (**a**) The relationship between metabolic rate and body mass after controlling for the temperature effects. Colored lines represent the species-specific (intraspecific) relationships (i.e., *b_i_*) derived from the LME model, with each color corresponding to a different species. Gray points indicate the species-level mean values for body mass and the corresponding metabolic rate. The solid black line depicts the phylogenetically informed interspecific relationship (i.e., *b*_0_) obtained from the PGLS analysis. (**b**) Frequency distribution of the intraspecific mass-scaling exponents (*b_i_*) estimated from the linear mixed-effects model. The blue vertical line indicates the overall (fixed-effect) intraspecific trend (*b*), and the red vertical line marks the interspecific exponent (*b*_0_) from the PGLS model.

The analyzed species exhibited substantial variation in individual body mass and corresponding metabolic rates (Figure 2a). Based on the linear mixed-effects (LME) model, the overall (fixed-effect) intraspecific mass-scaling exponent was 0.760 ± 0.012 (mean ± SE; *t*_3591_ = 66.01, *p* < 0.001). However, the species-specific exponents (*b*_i_) derived from the model showed considerable variation around this mean (Figure 2b), with a standard deviation of 0.072 (coefficient of variation = 0.094). Phylogenetic Generalized Least Squares (PGLS) regression on species means yielded an interspecific mass-scaling exponent of 0.768 ± 0.023 (*t*_173_ = 34.06, *p* < 0.001). The mean of the intraspecific exponents was not significantly different from this interspecific value (one-sample *t*-test: *t*_173_ = −1.328, *p* = 0.186).

**Figure 3:**
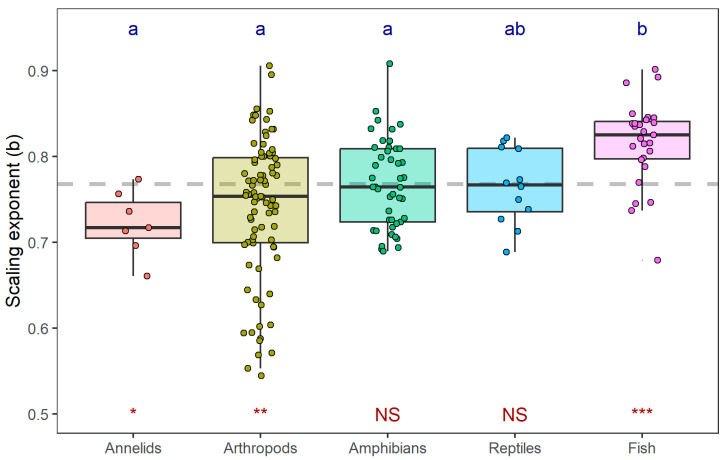
Variation in mass-scaling exponents across taxonomic groups. The boxplot displays the distribution of intraspecific mass-scaling exponents (*b_i_*) for each taxonomic group. Different lowercase letters above the boxes indicate statistically significant differences between groups, as determined by one-way ANOVA followed by Tukey’s HSD post hoc test (α = 0.05). The red asterisks below each box denote the significance of the difference between the mean intraspecific exponent of that group and the interspecific exponent (*b*_0_; the horizontal dashed line) obtained from PGLS, assessed by one-sample *t*-test (*** *p* < 0.001, ** *p* < 0.01, * *p* < 0.05, NS: not significant).

Analysis of variance indicated significant variation in intraspecific scaling exponents among taxonomic groups (ANOVA: *F*_4169_ = 7.143, *p* < 0.001; Figure 3). Fish displayed a significantly higher mass-scaling exponent (0.816 ± 0.049, mean ± SD) compared to several other groups, including amphibians (0.768 ± 0.051), arthropods (0.741 ± 0.082), and annelids (0.722 ± 0.038) (Tukey HSD: *p* < 0.05). The exponent for reptiles (0.765 ± 0.044) was not significantly different from that of fish. Compared with the interspecific mass-scaling exponent, fish showed significant higher mean intraspecific exponent (*t*_26_ = 5.125, *p* < 0.001), while annelids and arthropods showed significant lower mean value (for annelids: *t*_6_ = −3.190, *p* = 0.019; for arthropods: *t*_82_ = −2.974, *p* = 0.004).

### 3.3. Activation Energy (E): Intraspecific vs. Interspecific

**Figure 4:**
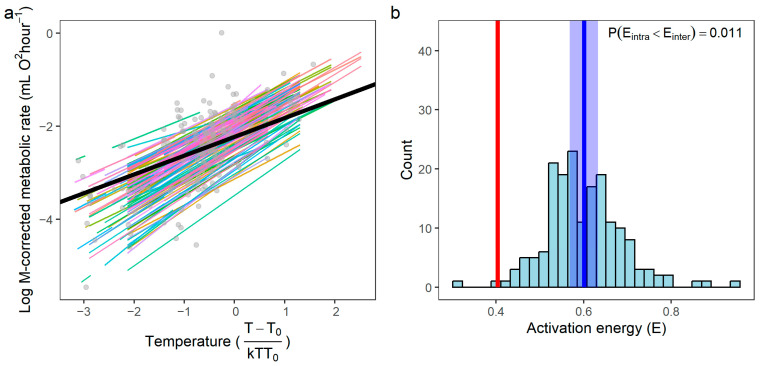
Temperature dependence of metabolic rate. (**a**) The relationship between metabolic rate and temperature after controlling for the body mass effects. Colored lines represent the species-specific (intraspecific) relationships (i.e., *E_i_*) derived from the LME model, with each color corresponding to a different species. Gray points indicate the species-level mean values for transformed temperature and the corresponding metabolic rate. The solid black line depicts the phylogenetically informed interspecific relationship (i.e., *E*_0_) obtained from the PGLS analysis. (**b**) Frequency distribution of the intraspecific activation energies (*E_i_*) estimated from the LME model. The blue vertical line indicates the overall (fixed-effect) intraspecific thermal sensitivity (*E*), and the red vertical line marks the interspecific activation energy (*E*_0_) from the PGLS model.

From the regression lines, it is evident that the intraspecific thermal trends (colored lines) have steeper slopes than the interspecific trend (black line; Figure 4a). Based on the linear mixed-effects (LME) model, the overall (fixed-effect) intraspecific activation energy was 0.601 ± 0.016 (mean ± SE; *t*_3591_ = 36.65, *p* < 0.001). The species-specific activation energies (*E*_i_) derived from the model ranges from 0.312 to 0.946 (Figure 4b), with a standard deviation of 0.088 (coefficient of variation = 0.146). Phylogenetic Generalized Least Squares (PGLS) regression on species means yielded an interspecific activation energy of 0.403 ± 0.073 (*t*_173_ = 5.560, *p* < 0.001). The mean of the intraspecific activation energies was significantly different from this interspecific value (one-sample *t*-test: *t*_173_ = 29.73, *p* < 0.001), and only 2 of the 174 intraspecific activation energies are lower than the interspecific value.

We tested the potential interaction between body mass and temperature on metabolic rate. In the PGLS analysis, the interaction term was non-significant (*p* = 0.883), and inclusion of this term slightly increased the model’s AIC from 348.7 to 350.7 (ANOVA comparison: *p* = 0.881). Similarly, in the LME analysis, the interaction was non-significant (*p* = 0.374), and the model with interaction exhibited a higher AIC (6165.9) than the additive model (6155.2), with ANOVA indicating that the additive model provided a better fit (*p* = 0.003). These results suggest that body size and temperature effects on metabolic rate are primarily additive across the dataset.

**Figure 5:**
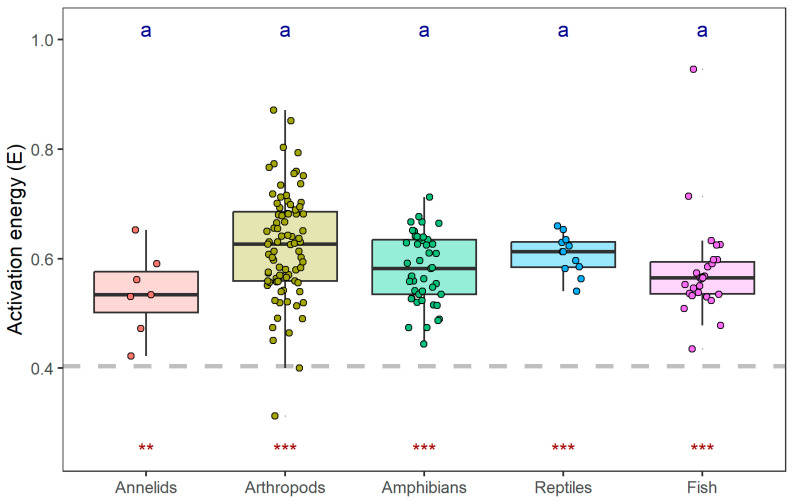
Variation in activation energy across taxonomic groups. The boxplot displays the distribution of activation energies (*E_i_*) for each taxonomic group. Different lowercase letters above the boxes indicate statistically significant differences between groups, as determined by one-way ANOVA followed by Tukey’s HSD post hoc test (α = 0.05). The red asterisks below each box denote the significance of the difference between the mean activation energy of that group and the interspecific value (*E*_0_; the horizontal dashed line) obtained from PGLS, assessed by one-sample *t*-test (*** *p* < 0.001, ** *p* < 0.01).

We detected significant difference in intraspecific activation energies among the taxonomic groups (ANOVA: *F*_4169_ = 3.54, *p* = 0.008; Figure 5). However, post hoc comparisons using Tukey’s HSD test did not identify any statistically significant pairwise differences between specific groups at the *α* = 0.05 level, despite the overall ANOVA significance. Arthropods exhibited the highest mean activation energy (0.623 ± 0.098, mean ± SD), followed by reptiles (0.608 ± 0.036), amphibians (0.581 ± 0.064), fish (0.577 ± 0.001), and annelids (0.538 ± 0.076). Compared with the interspecific activation energy, all six groups showed significant higher mean values (*t*-test, *p* = 0.003 for annelids, and *p* < 0.001 for the other groups).

## 4. Discussion

Our analysis of over 3700 metabolic rate measurements reveals distinct and systematic differences in how body mass and temperature correlate with metabolism within species compared to across species. These findings challenge the notion of universal metabolic constants and underscore the importance of a hierarchical perspective in metabolic ecology.

### 4.1. Hierarchical Scaling of Metabolism with Body Mass

We found no significant difference between the overall intraspecific mass-scaling exponent and the phylogenetically corrected interspecific exponent. This convergence around 0.768 approximates the classic interspecific 3/4-power law, but, as emphasized by Glazier (2005, 2022), this apparent central tendency should not be interpreted as evidence for a universal constant [11,17]. Rather, it likely reflects statistical averaging across clades and lifestyles that exhibit inherently different metabolic scaling relationships. For example, Glazier (2005) showed that pelagic invertebrates have significantly higher scaling exponents than non-pelagic species (median 0.916 vs. 0.747), illustrating how ecological mode and taxonomic affiliation shape metabolic scaling [11]. Consistent with this, our dataset reveals that fish exhibit higher mass-scaling exponents than most other groups, indicating that variation in scaling parameters reflects taxon-specific physiology and ecological adaptation rather than universal constraints [23].

Two non-mutually exclusive mechanisms can explain this increased intraspecific variability. First, from a physiological perspective, the metabolic rate of an individual is highly plastic, influenced by factors such as activity level, nutritional state, and ontogenetic stage [24,25]. Within the constrained body size range typical of a single species, these non-mass factors can disproportionately influence the estimated scaling slope. Furthermore, the interaction between body mass and temperature can modulate the exponent; congruent changes in mass and temperature can steepen the slope, while opposing changes can flatten it [14]. Second, from a statistical standpoint, the narrow body size range within species (often 1–2 orders of magnitude) increases the relative influence of measurement error and environmental noise on slope estimation, leading to greater variance [23]. However, as emphasized by Glazier (2006, 2022), this statistical effect is not the sole explanation [17,26]. Intraspecific scaling relationships are also shaped by biological and ecological factors, such as individual plasticity, environmental heterogeneity, and adaptive evolution, that operate primarily within species or populations. These influences make environmental effects on metabolic scaling more readily detectable within species than across species, where such signals tend to be averaged out. Thus, both statistical and biological processes contribute to the greater variability observed in intraspecific metabolic scaling.

The significant differences in scaling exponents among taxonomic groups further emphasize that phylogeny constrains metabolic scaling. The higher exponents in fish, compared to other groups, likely reflect divergent life-history strategies and physiological adaptations. A similar pattern was reported by Glazier (2005), who found that pelagic invertebrates exhibited markedly higher metabolic scaling exponents than non-pelagic taxa (median = 0.916 vs. 0.747) [11]. This convergence suggests that organisms inhabiting three-dimensional aquatic environments, such as fishes and pelagic invertebrates, tend to have steeper metabolic scaling, possibly because continuous swimming, buoyancy regulation, and high aerobic capacity impose stronger size-dependent energetic demands than in benthic or terrestrial species constrained to two-dimensional habitats. Similar conclusions have been reached by several previous studies [17,18,26,27], which demonstrated that metabolic scaling exponents vary among taxa due to differences in physiology, life-history strategies, and evolutionary history. Thus, both phylogenetic history and ecological mode of life jointly influence metabolic scaling, reinforcing that the “typical” scaling exponent is not a universal constant but a context-dependent outcome of evolutionary and ecological diversification.

### 4.2. Hierarchical Scaling of Metabolism with Temperature

Our finding that the intraspecific activation energy is consistently higher than the interspecific value indicates that temperature sensitivity diminishes at broader phylogenetic scales. This provides strong support for the “hierarchical attenuation hypothesis”, where the strength of temperature dependence diminishes from physiological to evolutionary scales [28,29,30]. Notably, the relationship between temperature and metabolism shows substantially greater scatter across species compared to the relationship between body mass and metabolism (compare Figure 2a and Figure 4a). This pattern is not an artifact of outliers but reflects a fundamental biological difference: while body mass exerts relatively consistent geometric and physiological constraints on metabolic processes, the underlying mechanisms are multifactorial, involving not only resource-distribution networks but also surface-area scaling, metabolic-level boundaries, and ecological or physiological modulation [17,31]. In contrast, thermal adaptation tends to be more labile, reflecting diverse biochemical and ecological adjustments across taxa. Species evolve diverse physiological strategies (e.g., metabolic compensation, shifts in thermal optima) to cope with their specific thermal niches. Consequently, when pooling data across species adapted to different climates, these varied strategies lead to a noisier aggregate relationship, further illustrating how ecological and evolutionary context dilutes the apparent temperature signal at broad phylogenetic scales.

Physiologically, species adapt to their thermal environments through mechanisms like mitochondrial density adjustments or enzyme concentration changes [32,33]. Cold-adapted species may upregulate metabolism, while warm-adapted species may downregulate it to maintain optimal function [34]. When data from species adapted to different thermal niches are pooled for an interspecific regression, these local adaptations average out, resulting in a flatter overall slope. Statistically, this interspecific averaging masks the full physiological sensitivity observable within a species acclimated or adapted to a specific thermal range. Additionally, sampling biases may favor data from organisms within their optimal thermal ranges, further contributing to the observed attenuation of the interspecific activation energy.

### 4.3. Implications for the Metabolic Theory of Ecology (MTE)

Our findings necessitate a refinement of the standard MTE framework. The theory’s core equation remains a valuable heuristic, but its parameters (*b*, *E*) should not be interpreted as immutable constants. Instead, they are hierarchical variables whose values depend on the biological scale of inquiry. This interpretation is consistent with critiques of the MTE by Glazier (2014, 2015) [3,12] and Glazier & Gjoni (2024) [6], who argued that the theory’s mechanistic assumptions are overly rigid and fail to account for context-dependent variation, particularly the potential interactive effects between body size and temperature on metabolic rate. At the intraspecific level, metabolism is plastic and responsive to immediate environmental and physiological conditions, whereas at the interspecific level, the observed relationships represent the aggregate outcome of evolutionary optimization and phylogenetic constraints [19,35,36].

Several authors have emphasized that body size and temperature may interact in determining metabolic rate [6,11,12,13,14]. Our analyses, however, revealed no significant interaction between these variables in either the PGLS or LME frameworks, and the inclusion of this term did not improve model fit. Therefore, our primary findings, regarding hierarchical differences in mass- and temperature-scaling, remain robust under an additive assumption. Nonetheless, we acknowledge that mass-temperature interactions may emerge under specific ecological or physiological contexts not fully represented in our dataset.

Prior analyses have questioned the mechanistic foundations of the Metabolic Theory of Ecology (MTE). O’Connor et al. (2007) demonstrated that the MTE’s predictions of temperature dependence and scaling are often inconsistent across taxa and ecological contexts, suggesting that its mechanistic underpinnings are incomplete [37]. Similarly, Martins (2023) argued that, although MTE is framed as a mechanistic unifying theory, it often functions more as a statistical description of tendencies emerging from diverse taxa and environments [38]. Our empirical findings, particularly the decoupling of mass-scaling exponents and activation energies across hierarchical levels, align with these critiques. They indicate that metabolic scaling is inherently hierarchical and context-dependent, and that a fully mechanistic ecological metabolic theory must explicitly incorporate scale, phylogeny, and ecological variability.

The recognition that biological scaling relationships can vary across hierarchical levels, from individuals to species and higher taxa, has a long history in comparative biology [11,20,26,27,39,40,41,42]. Building on this foundation, our results reinforce that metabolic scaling is inherently hierarchical and context-dependent. Therefore, a truly predictive metabolic theory must explicitly incorporate this multilevel structure. Rather than searching for universal constants, future work should focus on understanding the processes that cause scaling parameters to shift across biological levels. Approaches such as phylogenetic comparative methods (e.g., PGLS) and hierarchical Bayesian modeling are particularly well suited for disentangling the influences of physiology, ecology, and evolution on metabolic scaling [43,44].

We recognize that the present analysis, by necessity, excludes unicellular taxa due to insufficient within-species replication. Unicellular prokaryotes and eukaryotes exhibit distinct metabolic scaling patterns [17,45], which might alter the overall interspecific scaling exponent if incorporated. Our findings therefore apply specifically to multicellular ectothermic organisms.

## 5. Conclusions

In conclusion, our study demonstrates that metabolic scaling is inherently hierarchical, and the parameters of the MTE equation are scale-dependent rather than universal constants. The key findings are that the mass-scaling exponent (*b*) exhibits greater variability at the intraspecific level than observed across species, while the activation energy (*E*) is significantly lower in interspecific comparisons than the mean intraspecific value. This decoupling indicates that the mass-scaling relationship converges through interspecific averaging, whereas the temperature sensitivity is attenuated. Therefore, recognizing this scale dependence is crucial for accurately modeling ecological processes across different biological levels.

## Data Availability

The data used in this study can be found in Supplementary Materials.

## Supplementary Materials

The following supporting information can be downloaded at: https://www.mdpi.com/article/10.3390/biology15010084/s1, Table S1: The data used in this study. M indicates body mass in grams, T indicates temperature in °C, and BMR indicates basal metabolic rate in mL O^2^ hour^-1^. References [46,47,48] are cited in the Supplementary Materials.
