# Supplementary material for: Intraspecific Versus Interspecific Scaling of Metabolic Rate: Tests of the Metabolic Theory of Ecology Across Biological Hierarchies
Source: Biology (Basel). 2025 Dec 31;15(1):84. doi: 10.3390/biology15010084 (PMC12785121; doi:10.3390/biology15010084)
Supplement: Supplementary file 1 [file biology-15-00084-s001.zip › biology-4019536-supplementary.pdf]

Supplementary Materials

Intraspecific versus Interspecific Scaling of Metabolic Rate: Tests of the Metabolic Theory of Ecology Across Biological Hierarchies

Jiayin Wang [1], and Lei Zhao [1,*]

[1]Beijing Key Laboratory of Biodiversity and Organic Farming, College of Resources and Environmental Sciences, China Agricultural University, Beijing 100193, China; lei.zhao@cau.edu.cn
[*]Correspondence: lei.zhao@cau.edu.cn; Tel.: +86-18518276956

Table S1: The data used in this study. M indicates body mass in grams, T indicates temperature in ^{o}C, and BMR indicates basal metabolic rate in mL O^2 hour^{-1}.

Index	Group	Species	M (g)	T (°C)	BMR (mL O^2 hour^{-1})	Source
1	Unicells	Acetobacter aceti	7.5000E-13	30	8.1000E-14	Makarieva et al. 2008
2	Unicells	Acholeplasma laidlawii	4.0000E-14	37	1.0080E-14	Makarieva et al. 2008
3	Unicells	Achromobacter ruhlandii	2.0000E-13	30	5.4000E-13	Makarieva et al. 2008
4	Unicells	Achromobacter sp	6.0000E-13	30	9.0720E-13	Makarieva et al. 2008
5	Unicells	Achromobacter sp	6.0000E-13	30	3.7800E-12	Makarieva et al. 2008
6	Unicells	Achromobacter viscosus	6.0000E-13	30	3.7800E-12	Makarieva et al. 2008
7	Unicells	Achromobacter xerosis	5.0000E-13	30	2.0700E-12	Makarieva et al. 2008
8	Unicells	Acidovorax facilis	3.0000E-13	30	3.7800E-13	Makarieva et al. 2008
9	Unicells	Acinetobacter baumannii	2.0000E-12	30	4.3200E-12	Makarieva et al. 2008
10	Unicells	Acinetobacter calcoaceticus	2.0000E-12	30	2.4120E-12	Makarieva et al. 2008
11	Unicells	Acinetobacter johnsonii	2.0000E-12	30	1.6560E-11	Makarieva et al. 2008
12	Unicells	Acinetobacter sp	2.0000E-12	25	1.1880E-12	Makarieva et al. 2008
13	Unicells	Acinetobacter sp	2.0000E-12	30	2.1600E-12	Makarieva et al. 2008

14	Unicells	Acinetobacter sp	2.0000E-12	30	2.8800E-12	Makarieva et al. 2008
15	Unicells	Alcaligenes eutrophus	8.0000E-13	33	1.1952E-11	Makarieva et al. 2008
16	Unicells	Aminobacter lissarensis	6.0000E-13	25	1.7280E-13	Makarieva et al. 2008
17	Unicells	Amoebobacter purpureus	3.6000E-11	30	7.1280E-11	Makarieva et al. 2008
18	Unicells	Amoebobacter roseus	5.0000E-12	30	4.8600E-12	Makarieva et al. 2008
19	Unicells	Amoebobaeter pendens	5.0000E-12	30	7.6500E-12	Makarieva et al. 2008
20	Unicells	Anabaena flos-aquae	2.2000E-11	25	5.1480E-12	Makarieva et al. 2008
21	Unicells	Anabaena variabilis	1.4000E-11	39	7.0560E-12	Makarieva et al. 2008
22	Unicells	Anabaena variabilis	1.4000E-11	39	3.5280E-11	Makarieva et al. 2008
23	Unicells	Anabaena variabilis	1.4000E-11	24	4.5360E-11	Makarieva et al. 2008
24	Unicells	Anacystis nidulans PCC 6301	4.0000E-12	25	3.6000E-13	Makarieva et al. 2008
25	Unicells	Anacystis nidulans PCC 6301	4.0000E-12	39	2.3040E-12	Makarieva et al. 2008
26	Unicells	Anacystis nidulans PCC 6301	4.0000E-12	25	1.9440E-12	Makarieva et al. 2008
27	Unicells	Anacystis nidulans PCC 6301	4.0000E-12	39	5.6160E-12	Makarieva et al. 2008
28	Unicells	Anacystis nidulans PCC 6301	4.0000E-12	39	9.0000E-12	Makarieva et al. 2008
29	Unicells	Anacystis nidulans PCC 6301	4.0000E-12	25	3.4560E-12	Makarieva et al. 2008
30	Unicells	Anacystis nidulans PCC 6301	4.0000E-12	40	1.2960E-11	Makarieva et al. 2008
31	Unicells	Anacystis nidulans PCC 6301	4.0000E-12	40	1.6560E-11	Makarieva et al. 2008
32	Unicells	Anacystis nidulans PCC 6301	4.0000E-12	25	6.1200E-12	Makarieva et al. 2008
33	Unicells	Anacystis nidulans PCC 6301	4.0000E-12	39	1.8000E-11	Makarieva et al. 2008
34	Unicells	Anacystis nidulans PCC 6301	4.0000E-12	40	2.0880E-11	Makarieva et al. 2008
35	Unicells	Aphanocapsa PCC 6714	1.0000E-11	25	2.7000E-13	Makarieva et al. 2008
36	Unicells	Aquaspirillum itersonii	9.0000E-13	30	1.6200E-12	Makarieva et al. 2008
37	Unicells	Arthrobacter crystallopoietes	1.7000E-12	30	6.1200E-14	Makarieva et al. 2008
38	Unicells	Arthrobacter globiformis	5.0000E-13	30	7.4700E-13	Makarieva et al. 2008
39	Unicells	Arthrobacter sp	2.0000E-13	25	2.7000E-14	Makarieva et al. 2008

40	Unicells	Arthrobacter sp	1.5000E-12	30	3.2400E-13	Makarieva et al. 2008
41	Unicells	Asterionella formosa	3.9000E-10	7	1.4700E-10	Makarieva et al. 2008
42	Unicells	Asterionella formosa	4.1000E-10	10	1.1904E-10	Makarieva et al. 2008
43	Unicells	Asterionella formosa	4.1000E-10	14	1.6103E-10	Makarieva et al. 2008
44	Unicells	Asterionella formosa	4.1000E-10	11	1.4701E-10	Makarieva et al. 2008
45	Unicells	Asterionella formosa	4.1000E-10	11	1.6797E-10	Makarieva et al. 2008
46	Unicells	Asterionella formosa	4.1000E-10	16	2.4502E-10	Makarieva et al. 2008
47	Unicells	Asterionella formosa	4.1000E-10	6	1.4701E-10	Makarieva et al. 2008
48	Unicells	Asterionella formosa	4.1000E-10	5	1.4000E-10	Makarieva et al. 2008
49	Unicells	Asterionella formosa	4.1000E-10	10	2.8701E-10	Makarieva et al. 2008
50	Unicells	Asterionella formosa	4.1000E-10	16	5.9505E-10	Makarieva et al. 2008
51	Unicells	Asterionella formosa	4.1000E-10	5	3.1498E-10	Makarieva et al. 2008
52	Unicells	Azomonas agilis	1.3000E-11	26	4.9140E-11	Makarieva et al. 2008
53	Unicells	Azorhizobium caulinodans	5.0000E-13	30	3.4200E-12	Makarieva et al. 2008
54	Unicells	Azospirillum brasiliense	1.0000E-12	37	4.8600E-12	Makarieva et al. 2008
55	Unicells	Azospirillum lipoferum	4.0000E-12	37	2.8080E-11	Makarieva et al. 2008
56	Unicells	Azotobacter chroococcum	1.4000E-11	30	6.3000E-11	Makarieva et al. 2008
57	Unicells	Azotobacter vinelandii	5.0000E-13	30	1.3500E-13	Makarieva et al. 2008
58	Unicells	Azotobacter vinelandii	5.0000E-13	30	2.7000E-12	Makarieva et al. 2008
59	Unicells	Bacillus cereus	3.7000E-12	37	9.3240E-12	Makarieva et al. 2008
60	Unicells	Bacillus cereus	3.7000E-12	30	2.0646E-11	Makarieva et al. 2008
61	Unicells	Bacillus cereus	3.7000E-12	30	6.4602E-11	Makarieva et al. 2008
62	Unicells	Bacillus firmus	9.0000E-13	30	2.1060E-12	Makarieva et al. 2008
63	Unicells	Bacillus megaterium	7.0000E-12	30	4.1580E-12	Makarieva et al. 2008
64	Unicells	Bacillus megaterium	7.0000E-12	30	1.6380E-11	Makarieva et al. 2008
65	Unicells	Bacillus megaterium	7.0000E-12	30	3.1500E-11	Makarieva et al. 2008

66	Unicells	Bacillus megaterium	7.0000E-12	37	5.9220E-11	Makarieva et al. 2008
67	Unicells	Bacillus megaterium	7.0000E-12	25	7.3080E-11	Makarieva et al. 2008
68	Unicells	Bacillus popilliae	8.0000E-13	30	1.1520E-13	Makarieva et al. 2008
69	Unicells	Bacillus pumilus	7.0000E-13	30	6.3000E-13	Makarieva et al. 2008
70	Unicells	Bacillus stearothermophilus	7.0000E-13	50	1.0332E-12	Makarieva et al. 2008
71	Unicells	Bacillus subtilis	1.4000E-12	30	8.3160E-13	Makarieva et al. 2008
72	Unicells	Bacillus subtilis	1.4000E-12	37	1.6380E-12	Makarieva et al. 2008
73	Unicells	Bacillus subtilis	1.4000E-12	30	1.4112E-12	Makarieva et al. 2008
74	Unicells	Bacillus subtilis	1.4000E-12	30	8.0640E-12	Makarieva et al. 2008
75	Unicells	Bacillus subtilis	1.4000E-12	37	1.8900E-11	Makarieva et al. 2008
76	Unicells	Bdellovibrio	3.0000E-13	30	1.3500E-12	Makarieva et al. 2008
77	Unicells	Bradyrhizobium japonicum	7.0000E-13	29	1.2600E-13	Makarieva et al. 2008
78	Unicells	Brucella melitensis	3.0000E-13	34	3.5100E-13	Makarieva et al. 2008
79	Unicells	Burkholderia sp	7.0000E-13	30	2.6460E-12	Makarieva et al. 2008
80	Unicells	Cellvibrio gilvus	2.0000E-12	30	1.3320E-11	Makarieva et al. 2008
81	Unicells	Chaetoceros furcellatus	1.3000E-10	0.5	1.0296E-10	Makarieva et al. 2008
82	Unicells	Chaetoceros furcellatus	1.5000E-10	0.5	2.0250E-11	Makarieva et al. 2008
83	Unicells	Chaetoceros furcellatus	2.0000E-10	0.5	2.0160E-10	Makarieva et al. 2008
84	Unicells	Chaetoceros furcellatus	3.0000E-10	0.5	1.4040E-10	Makarieva et al. 2008
85	Unicells	Chlamydomonas reinhardtii	2.0000E-09	22	3.2400E-09	Makarieva et al. 2008
86	Unicells	Chlamydomonas reinhardtii	2.0000E-09	25	5.4000E-09	Makarieva et al. 2008
87	Unicells	Chlamydomonas reinhardtii	2.0000E-09	35	1.1160E-08	Makarieva et al. 2008
88	Unicells	Chlamydomonas reinhardtii	2.0000E-09	15	3.4920E-09	Makarieva et al. 2008
89	Unicells	Chlorella kessleri	9.0000E-11	27	2.2680E-10	Makarieva et al. 2008
90	Unicells	Chlorella pyrenoidosa	3.0000E-11	25	4.1580E-11	Makarieva et al. 2008
91	Unicells	Chlorella pyrenoidosa	3.2000E-11	25	5.6448E-11	Makarieva et al. 2008

92	Unicells	Chlorella pyrenoidosa	3.4000E-11	25	9.1800E-11	Makarieva et al. 2008
93	Unicells	Chlorella pyrenoidosa	4.9000E-11	25	1.6758E-10	Makarieva et al. 2008
94	Unicells	Chlorella pyrenoidosa	7.5000E-11	25	3.1050E-10	Makarieva et al. 2008
95	Unicells	Chlorella pyrenoidosa	9.2000E-11	25	3.3120E-10	Makarieva et al. 2008
96	Unicells	Chromatium vinosum	1.5000E-12	30	5.9400E-13	Makarieva et al. 2008
97	Unicells	Coccochloris peniocystis	1.0000E-12	40	3.2400E-12	Makarieva et al. 2008
98	Unicells	Coccochloris peniocystis	1.0000E-12	30	2.5200E-12	Makarieva et al. 2008
99	Unicells	Coccochloris peniocystis	1.0000E-12	20	1.5300E-12	Makarieva et al. 2008
100	Unicells	Coscinodiscus sp. C38B	2.7500E-07	18	2.9205E-07	Makarieva et al. 2008
101	Unicells	Coscinodiscus sp. CoA	6.2000E-06	18	6.0264E-06	Makarieva et al. 2008
102	Unicells	Delftia acidovorans	8.0000E-13	30	1.8720E-12	Makarieva et al. 2008
103	Unicells	Delftia acidovorans	8.0000E-13	30	1.8720E-12	Makarieva et al. 2008
104	Unicells	Delftia acidovorans	8.0000E-13	30	2.1600E-12	Makarieva et al. 2008
105	Unicells	Delftia acidovorans	8.0000E-13	30	4.3200E-12	Makarieva et al. 2008
106	Unicells	Desulfovibrio salexigens	1.5000E-12	30	3.5100E-12	Makarieva et al. 2008
107	Unicells	Ditylum brightwellii	1.1800E-07	18	6.3720E-08	Makarieva et al. 2008
108	Unicells	Dunaliella salina	1.5700E-09	20	1.1304E-09	Makarieva et al. 2008
109	Unicells	Dunaliella tertiolecta	6.9000E-11	15	1.0805E-10	Makarieva et al. 2008
110	Unicells	Dunaliella tertiolecta	7.3000E-11	15	1.4454E-10	Makarieva et al. 2008
111	Unicells	Dunaliella tertiolecta	8.4000E-11	15	2.4192E-10	Makarieva et al. 2008
112	Unicells	Dunaliella tertiolecta	9.0000E-11	15	4.2120E-10	Makarieva et al. 2008
113	Unicells	Dunaliella tertiolecta	1.0400E-10	15	5.4288E-10	Makarieva et al. 2008
114	Unicells	Dunaliella tertiolecta	1.1200E-10	15	6.2496E-10	Makarieva et al. 2008
115	Unicells	Dunaliella tertiolecta	1.1500E-10	15	7.0380E-10	Makarieva et al. 2008
116	Unicells	Dunaliella tertiolecta	1.2000E-10	18	2.0520E-10	Makarieva et al. 2008
117	Unicells	Dunaliella tertiolecta	1.2000E-10	18	5.8320E-10	Makarieva et al. 2008

118	Unicells	Dunaliella tertiolecta	1.2000E-10	18	7.3440E-10	Makarieva et al. 2008
119	Unicells	Dunaliella tertiolecta	1.2000E-10	18	8.6400E-10	Makarieva et al. 2008
120	Unicells	Dunaliella tertiolecta	1.2000E-10	18	1.9440E-09	Makarieva et al. 2008
121	Unicells	Dunaliella tertiolecta	1.2000E-10	18	1.9440E-09	Makarieva et al. 2008
122	Unicells	Dunaliella tertiolecta	1.2000E-10	18	2.0520E-09	Makarieva et al. 2008
123	Unicells	Emiliania huxleyi	6.4000E-11	15	9.2160E-11	Makarieva et al. 2008
124	Unicells	Emiliania huxleyi	6.4000E-11	15	9.6768E-11	Makarieva et al. 2008
125	Unicells	Emiliania huxleyi	6.4000E-11	17.5	1.1520E-10	Makarieva et al. 2008
126	Unicells	Emiliania huxleyi	6.4000E-11	15	1.2672E-10	Makarieva et al. 2008
127	Unicells	Emiliania huxleyi	6.4000E-11	15	1.3824E-10	Makarieva et al. 2008
128	Unicells	Emiliania huxleyi	6.4000E-11	15	1.3824E-10	Makarieva et al. 2008
129	Unicells	Emiliania huxleyi	6.4000E-11	15	1.3824E-10	Makarieva et al. 2008
130	Unicells	Emiliania huxleyi	6.4000E-11	15	1.6128E-10	Makarieva et al. 2008
131	Unicells	Emiliania huxleyi	6.4000E-11	15	1.7280E-10	Makarieva et al. 2008
132	Unicells	Emiliania huxleyi	6.4000E-11	15	1.7280E-10	Makarieva et al. 2008
133	Unicells	Emiliania huxleyi	6.4000E-11	15	1.8432E-10	Makarieva et al. 2008
134	Unicells	Emiliania huxleyi	6.4000E-11	15	1.9584E-10	Makarieva et al. 2008
135	Unicells	Emiliania huxleyi	6.4000E-11	17.5	3.4560E-10	Makarieva et al. 2008
136	Unicells	Emiliania huxleyi	6.4000E-11	17.5	3.8016E-10	Makarieva et al. 2008
137	Unicells	Enterobacter aerogenes	3.0000E-13	30	5.4000E-13	Makarieva et al. 2008
138	Unicells	Enterobacter cloacae	9.0000E-13	30	5.0220E-12	Makarieva et al. 2008
139	Unicells	Enterococcus cecorum	4.0000E-13	30	2.7360E-13	Makarieva et al. 2008
140	Unicells	Enterococcus faecalis	2.0000E-13	30	1.0800E-14	Makarieva et al. 2008
141	Unicells	Enterococcus sp	8.0000E-13	30	4.7520E-12	Makarieva et al. 2008
142	Unicells	Escherichia coli	7.0000E-13	37	4.0320E-12	Makarieva et al. 2008
143	Unicells	Escherichia coli	7.0000E-13	26	5.6700E-13	Makarieva et al. 2008

144	Unicells	Escherichia coli	7.0000E-13	30	8.4420E-13	Makarieva et al. 2008
145	Unicells	Escherichia coli	7.0000E-13	37	1.5120E-12	Makarieva et al. 2008
146	Unicells	Escherichia coli	7.0000E-13	35	1.8900E-12	Makarieva et al. 2008
147	Unicells	Escherichia coli	7.0000E-13	37	2.5200E-12	Makarieva et al. 2008
148	Unicells	Escherichia coli	7.0000E-13	30	1.8900E-12	Makarieva et al. 2008
149	Unicells	Escherichia coli	7.0000E-13	30	2.2050E-12	Makarieva et al. 2008
150	Unicells	Escherichia coli	7.0000E-13	37	7.8120E-12	Makarieva et al. 2008
151	Unicells	Euglena gracilis	9.7000E-09	20	1.5714E-08	Makarieva et al. 2008
152	Unicells	Euglena gracilis	1.0300E-08	20	1.2978E-08	Makarieva et al. 2008
153	Unicells	Flavobacterium capsulatum	3.0000E-13	30	3.4020E-12	Makarieva et al. 2008
154	Unicells	Fragilaria crotonensis	3.0000E-10	16	1.2420E-09	Makarieva et al. 2008
155	Unicells	Francisella tularensis	1.0000E-14	37	6.6600E-15	Makarieva et al. 2008
156	Unicells	Gloeobacter violaceus	7.0000E-13	25	3.5280E-12	Makarieva et al. 2008
157	Unicells	Gloeothece sp. PCC	1.8000E-10	20	1.2960E-10	Makarieva et al. 2008
158	Unicells	Gloeothece sp. PCC	1.8000E-10	20	4.2120E-10	Makarieva et al. 2008
159	Unicells	Gloeothece sp. PCC	1.8000E-10	20	5.1840E-10	Makarieva et al. 2008
160	Unicells	Gloeothece sp. PCC	1.8000E-10	20	6.4800E-10	Makarieva et al. 2008
161	Unicells	Gloeothece sp. PCC	1.8000E-10	20	1.0368E-09	Makarieva et al. 2008
162	Unicells	Gloeothece sp. PCC	1.8000E-10	20	1.1664E-09	Makarieva et al. 2008
163	Unicells	Gloeothece sp. PCC	1.8000E-10	20	1.2960E-09	Makarieva et al. 2008
164	Unicells	Gonyaulax polyedra	2.1000E-08	20	2.1546E-08	Makarieva et al. 2008
165	Unicells	Gonyaulax tamarensis	1.0653E-08	15	5.6567E-08	Makarieva et al. 2008
166	Unicells	Gonyaulax tamarensis	1.3036E-08	15	4.9276E-08	Makarieva et al. 2008
167	Unicells	Gonyaulax tamarensis	1.3306E-08	15	5.4608E-08	Makarieva et al. 2008
168	Unicells	Gonyaulax tamarensis	1.4137E-08	15	3.8933E-08	Makarieva et al. 2008
169	Unicells	Gonyaulax tamarensis	1.5150E-08	15	3.2724E-08	Makarieva et al. 2008

170	Unicells	Gonyaulax tamarensis	1.6210E-08	15	8.2866E-08	Makarieva et al. 2008
171	Unicells	Gymnodinium nelsoni	1.4800E-07	20	2.9304E-08	Makarieva et al. 2008
172	Unicells	Haemophilus influenzae	1.4000E-13	37	1.2600E-14	Makarieva et al. 2008
173	Unicells	Haemophilus parainfluenzae	4.0000E-13	37	2.5200E-13	Makarieva et al. 2008
174	Unicells	Halobacterium salinarum	3.9000E-12	30	1.1934E-11	Makarieva et al. 2008
175	Unicells	Halomonas halodenitrificans	4.0000E-13	25	4.8240E-12	Makarieva et al. 2008
176	Unicells	Isochrysis galbana	3.0000E-11	18	3.1320E-11	Makarieva et al. 2008
177	Unicells	Isochrysis galbana	3.0000E-11	18	3.1320E-11	Makarieva et al. 2008
178	Unicells	Isochrysis galbana	3.0000E-11	18	3.5100E-11	Makarieva et al. 2008
179	Unicells	Isochrysis galbana	3.1000E-11	18	4.3524E-11	Makarieva et al. 2008
180	Unicells	Isochrysis galbana	3.2000E-11	18	4.4928E-11	Makarieva et al. 2008
181	Unicells	Isochrysis galbana	3.3000E-11	18	4.6332E-11	Makarieva et al. 2008
182	Unicells	Isochrysis galbana	3.3000E-11	18	7.7220E-11	Makarieva et al. 2008
183	Unicells	Isochrysis galbana	3.4000E-11	18	5.9364E-11	Makarieva et al. 2008
184	Unicells	Isochrysis galbana	3.4000E-11	18	7.1604E-11	Makarieva et al. 2008
185	Unicells	Isochrysis galbana	3.7000E-11	18	7.9920E-11	Makarieva et al. 2008
186	Unicells	Isochrysis galbana	5.0000E-11	18	1.0800E-10	Makarieva et al. 2008
187	Unicells	Isochrysis galbana	5.2000E-11	18	1.8720E-10	Makarieva et al. 2008
188	Unicells	Isochrysis galbana	5.5000E-11	18	1.4850E-10	Makarieva et al. 2008
189	Unicells	Isochrysis galbana	5.7000E-11	18	1.3338E-10	Makarieva et al. 2008
190	Unicells	Klebsiella pheumoniae	4.0000E-13	30	2.3760E-13	Makarieva et al. 2008
191	Unicells	Klebsiella pheumoniae	4.0000E-13	30	4.8240E-13	Makarieva et al. 2008
192	Unicells	Klebsiella pheumoniae	4.0000E-13	37	1.4400E-12	Makarieva et al. 2008
193	Unicells	Lactobacillus brevis	1.6000E-12	30	5.7600E-14	Makarieva et al. 2008
194	Unicells	Lactococcus lactis	2.0000E-13	30	2.1600E-14	Makarieva et al. 2008
195	Unicells	Lactococcus lactis	2.0000E-13	30	8.2800E-14	Makarieva et al. 2008

196	Unicells	Lactococcus sp	8.0000E-13	30	3.3120E-13	Makarieva et al. 2008
197	Unicells	Legionella pneumophila	3.0000E-13	37	1.0800E-12	Makarieva et al. 2008
198	Unicells	Legionella pneumophila	3.0000E-13	37	4.1580E-12	Makarieva et al. 2008
199	Unicells	Leptocylindrus danicus	1.1580E-09	15	8.3376E-10	Makarieva et al. 2008
200	Unicells	Methylobacterium extorquens	1.0000E-12	30	1.0800E-12	Makarieva et al. 2008
201	Unicells	Methylomicrobium sp	3.0000E-12	30	5.9400E-12	Makarieva et al. 2008
202	Unicells	Methylophilus methylotrophus	1.5000E-13	40	4.3200E-14	Makarieva et al. 2008
203	Unicells	Methylosinus trichosporium	1.0000E-12	30	7.5600E-12	Makarieva et al. 2008
204	Unicells	Micrococcus luteus	1.1000E-12	37	2.3760E-13	Makarieva et al. 2008
205	Unicells	Micrococcus luteus	1.1000E-12	35	1.1880E-12	Makarieva et al. 2008
206	Unicells	Micrococcus luteus	1.1000E-12	37	3.5640E-12	Makarieva et al. 2008
207	Unicells	Micrococcus luteus	1.1000E-12	30	2.9700E-12	Makarieva et al. 2008
208	Unicells	Monochrysis lutheri	2.5000E-11	20	3.7800E-11	Makarieva et al. 2008
209	Unicells	Monochrysis lutheri	2.6000E-11	20	6.0840E-11	Makarieva et al. 2008
210	Unicells	Monochrysis lutheri	2.9000E-11	20	7.3080E-11	Makarieva et al. 2008
211	Unicells	Monochrysis lutheri	4.0000E-11	20	5.7600E-11	Makarieva et al. 2008
212	Unicells	Moraxella catarrhalis	1.3000E-12	37	3.9780E-13	Makarieva et al. 2008
213	Unicells	Moraxella catarrhalis	1.3000E-12	30	5.3820E-12	Makarieva et al. 2008
214	Unicells	Moraxella osloensis	2.3000E-12	30	5.3820E-12	Makarieva et al. 2008
215	Unicells	Mycobacterium phlei	4.0000E-13	37	1.0080E-12	Makarieva et al. 2008
216	Unicells	Mycobacterium phlei	4.0000E-13	30	1.9440E-12	Makarieva et al. 2008
217	Unicells	Mycobacterium tuberculosis	2.0000E-13	37	7.9200E-14	Makarieva et al. 2008
218	Unicells	Mycobacterium tuberculosis	2.0000E-13	37.8	2.5200E-13	Makarieva et al. 2008
219	Unicells	Mycobacterium tuberculosis	2.0000E-13	37	1.3320E-12	Makarieva et al. 2008
220	Unicells	Mycobacterium tuberculosis	2.0000E-13	37	1.4400E-13	Makarieva et al. 2008
221	Unicells	Myxococcus xanthus	1.0000E-12	30	8.2800E-13	Makarieva et al. 2008

222	Unicells	Nannochloris atomus	6.0000E-12	23	9.7200E-12	Makarieva et al. 2008
223	Unicells	Navicula pelliculosa	9.5000E-11	20	2.2230E-10	Makarieva et al. 2008
224	Unicells	Neisseria elongata	2.0000E-13	30	2.9880E-13	Makarieva et al. 2008
225	Unicells	Neisseria flava	2.0000E-13	30	4.3200E-13	Makarieva et al. 2008
226	Unicells	Neisseria gonorrhoeae	2.0000E-13	37	2.1600E-14	Makarieva et al. 2008
227	Unicells	Neisseria mucosa	2.0000E-13	30	5.4000E-13	Makarieva et al. 2008
228	Unicells	Neisseria sicca	2.0000E-13	30	4.6800E-13	Makarieva et al. 2008
229	Unicells	Nitrobacter winogradskyi	2.4000E-13	30	4.3200E-13	Makarieva et al. 2008
230	Unicells	Nitrobacter winogradskyi	2.4000E-13	25	4.7520E-13	Makarieva et al. 2008
231	Unicells	Nitrosomonas europaea	6.0000E-13	25	1.1880E-12	Makarieva et al. 2008
232	Unicells	Nocardia corallina	2.1000E-12	30	6.4260E-13	Makarieva et al. 2008
233	Unicells	Nocardia corallina	2.1000E-12	30.3	1.5120E-12	Makarieva et al. 2008
234	Unicells	Nocardia farcinica	1.0000E-13	30	1.4940E-13	Makarieva et al. 2008
235	Unicells	Nostoc commune	8.0000E-12	27	8.6400E-13	Makarieva et al. 2008
236	Unicells	Nostoc commune var. flagelliforme	8.0000E-12	27	2.0160E-12	Makarieva et al. 2008
237	Unicells	Nostoc muscorum G.	3.3000E-11	25	1.0692E-11	Makarieva et al. 2008
238	Unicells	Nostoc muscorum G.	3.3000E-11	25	4.3362E-11	Makarieva et al. 2008
239	Unicells	Ochromonas sp.	7.0000E-11	20	7.0560E-11	Makarieva et al. 2008
240	Unicells	Olisthodiscus luteus	8.3300E-10	15	1.3195E-09	Makarieva et al. 2008
241	Unicells	Olisthodiscus luteus	9.0500E-10	15	1.0426E-09	Makarieva et al. 2008
242	Unicells	Olisthodiscus luteus	9.4500E-10	15	1.2077E-09	Makarieva et al. 2008
243	Unicells	Olisthodiscus luteus	1.0230E-09	15	9.0229E-10	Makarieva et al. 2008
244	Unicells	Olisthodiscus luteus	1.1240E-09	15	2.1244E-09	Makarieva et al. 2008
245	Unicells	Olisthodiscus luteus	1.1500E-09	15	2.7117E-09	Makarieva et al. 2008
246	Unicells	Oscillatoria spp.	7.8500E-10	29.1	9.8910E-10	Makarieva et al. 2008
247	Unicells	Oscillatoria spp.	7.8500E-10	28.8	1.9782E-09	Makarieva et al. 2008

248	Unicells	Oscillatoria spp.	7.8500E-10	25.5	7.2063E-09	Makarieva et al. 2008
249	Unicells	Oscillatoria spp.	7.8500E-10	27	3.0803E-08	Makarieva et al. 2008
250	Unicells	Oscillatoria spp.	7.8500E-10	27	3.2923E-08	Makarieva et al. 2008
251	Unicells	Oscillatoria spp.	7.8500E-10	27	4.4227E-08	Makarieva et al. 2008
252	Unicells	Oscillatoria spp.	7.8500E-10	27	6.6128E-08	Makarieva et al. 2008
253	Unicells	Oscillatoria terebriformis	6.5000E-11	45	3.3930E-10	Makarieva et al. 2008
254	Unicells	Paracoccus denitrificans	1.6000E-13	30	2.3904E-13	Makarieva et al. 2008
255	Unicells	Pediococcus acidilactici	2.2000E-12	30	1.3068E-12	Makarieva et al. 2008
256	Unicells	Peridinium gatunense	1.0000E-08	22	5.9400E-08	Makarieva et al. 2008
257	Unicells	Phaeocystis globosa	1.0600E-10	17.5	4.9608E-10	Makarieva et al. 2008
258	Unicells	Phaeocystis globosa	1.0600E-10	17.5	6.1056E-10	Makarieva et al. 2008
259	Unicells	Phaeocystis globosa	1.0600E-10	17.5	8.7768E-10	Makarieva et al. 2008
260	Unicells	Phaeodactylum tricornutum	6.0000E-11	18	3.8880E-10	Makarieva et al. 2008
261	Unicells	Phaeodactylum tricornutum	1.2000E-10	18	4.5360E-10	Makarieva et al. 2008
262	Unicells	Phaeodactylum tricornutum	1.2000E-10	15	4.5360E-10	Makarieva et al. 2008
263	Unicells	Phaeodactylum tricornutum	1.2000E-10	15	4.9680E-10	Makarieva et al. 2008
264	Unicells	Phaeodactylum tricornutum	1.2000E-10	18	6.2640E-10	Makarieva et al. 2008
265	Unicells	Phaeodactylum tricornutum	1.2000E-10	15	5.6160E-10	Makarieva et al. 2008
266	Unicells	Phaeodactylum tricornutum	1.2000E-10	18	7.5600E-10	Makarieva et al. 2008
267	Unicells	Phaeodactylum tricornutum	1.2000E-10	18	8.2080E-10	Makarieva et al. 2008
268	Unicells	Phaeodactylum tricornutum	1.2000E-10	18	9.2880E-10	Makarieva et al. 2008
269	Unicells	Phaeodactylum tricornutum	1.2000E-10	18	9.7200E-10	Makarieva et al. 2008
270	Unicells	Phaeodactylum tricornutum	1.2000E-10	18	1.1232E-09	Makarieva et al. 2008
271	Unicells	Phaeospirillum fulvum	2.0000E-12	28	1.0080E-11	Makarieva et al. 2008
272	Unicells	Phormidium autumnale (Ag.) Gom.	3.0000E-11	2	6.4800E-13	Makarieva et al. 2008
273	Unicells	Phormidium autumnale (Ag.) Gom.	3.0000E-11	5	1.1340E-12	Makarieva et al. 2008

274	Unicells	Phormidium autumnale (Ag.) Gom.	3.0000E-11	10	1.6200E-12	Makarieva et al. 2008
275	Unicells	Phormidium autumnale (Ag.) Gom.	3.0000E-11	15	2.7000E-12	Makarieva et al. 2008
276	Unicells	Phormidium autumnale (Ag.) Gom.	3.0000E-11	20	4.8600E-12	Makarieva et al. 2008
277	Unicells	Phormidium luridum	4.4000E-12	25	6.7320E-12	Makarieva et al. 2008
278	Unicells	Picrophilus oshimae	1.0000E-12	60	4.5000E-12	Makarieva et al. 2008
279	Unicells	Planktothrix agardhii	1.9000E-11	20	1.8468E-11	Makarieva et al. 2008
280	Unicells	Plectonema boryanum	4.4000E-12	26	4.4352E-12	Makarieva et al. 2008
281	Unicells	Plectonema boryanum	4.4000E-12	26	1.5840E-11	Makarieva et al. 2008
282	Unicells	Plectonema boryanum	4.4000E-12	26	4.9104E-11	Makarieva et al. 2008
283	Unicells	Prochloron sp.	5.6000E-09	28	3.0240E-08	Makarieva et al. 2008
284	Unicells	Prochloron sp.	5.6000E-09	28	1.2802E-07	Makarieva et al. 2008
285	Unicells	Prorocentrum micans	4.3400E-09	18	5.7028E-09	Makarieva et al. 2008
286	Unicells	Prorocentrum micans	5.0960E-09	18	7.9803E-09	Makarieva et al. 2008
287	Unicells	Prorocentrum micans	5.1220E-09	18	1.1985E-08	Makarieva et al. 2008
288	Unicells	Prorocentrum micans	5.3500E-09	18	1.3482E-08	Makarieva et al. 2008
289	Unicells	Proteus morganii	4.0000E-13	30	3.6000E-13	Makarieva et al. 2008
290	Unicells	Proteus vulgaris	4.0000E-13	37	3.6000E-13	Makarieva et al. 2008
291	Unicells	Proteus vulgaris	4.0000E-13	30	3.6000E-13	Makarieva et al. 2008
292	Unicells	Pseudomonas aeruginosa	5.0000E-13	30	6.0300E-13	Makarieva et al. 2008
293	Unicells	Pseudomonas aeruginosa	5.0000E-13	30	9.0000E-13	Makarieva et al. 2008
294	Unicells	Pseudomonas aeruginosa	5.0000E-13	30	2.5200E-12	Makarieva et al. 2008
295	Unicells	Pseudomonas aeruginosa	5.0000E-13	37	4.2300E-12	Makarieva et al. 2008
296	Unicells	Pseudomonas aeruginosa	5.0000E-13	30	4.7700E-12	Makarieva et al. 2008
297	Unicells	Pseudomonas fluorescens	1.0000E-12	28	7.9200E-13	Makarieva et al. 2008
298	Unicells	Pseudomonas fluorescens	1.0000E-12	26	1.8000E-12	Makarieva et al. 2008
299	Unicells	Pseudomonas fluorescens	1.0000E-12	30	2.7000E-12	Makarieva et al. 2008

300	Unicells	Pseudomonas fluorescens	1.0000E-12	30	3.2400E-12	Makarieva et al. 2008
301	Unicells	Pseudomonas fluorescens	1.0000E-12	30	5.9400E-12	Makarieva et al. 2008
302	Unicells	Pseudomonas fluorescens	1.0000E-12	30	6.1200E-12	Makarieva et al. 2008
303	Unicells	Pseudomonas fluorescens	1.0000E-12	30	1.0800E-11	Makarieva et al. 2008
304	Unicells	Pseudomonas formicans	7.0000E-12	30	1.2600E-11	Makarieva et al. 2008
305	Unicells	Pseudomonas oleovorans	1.6000E-13	30	9.5040E-14	Makarieva et al. 2008
306	Unicells	Pseudomonas oleovorans	1.6000E-13	30	1.1520E-12	Makarieva et al. 2008
307	Unicells	Pseudomonas putida	1.7000E-12	30	3.0600E-13	Makarieva et al. 2008
308	Unicells	Pseudomonas putida	1.7000E-12	30	8.5680E-13	Makarieva et al. 2008
309	Unicells	Pseudomonas putida	1.7000E-12	30	1.3464E-12	Makarieva et al. 2008
310	Unicells	Pseudomonas putida	1.7000E-12	30	2.5398E-12	Makarieva et al. 2008
311	Unicells	Pseudomonas putida	1.7000E-12	37	5.8140E-12	Makarieva et al. 2008
312	Unicells	Pseudomonas putida	1.7000E-12	30	5.2020E-12	Makarieva et al. 2008
313	Unicells	Pseudomonas putida	1.7000E-12	30	1.0404E-11	Makarieva et al. 2008
314	Unicells	Rhizobium	1.5000E-12	30	5.4000E-12	Makarieva et al. 2008
315	Unicells	Rhizobium japonicum	7.0000E-13	23	2.0160E-12	Makarieva et al. 2008
316	Unicells	Rhizobium leguminosarum	6.0000E-13	30	1.0260E-12	Makarieva et al. 2008
317	Unicells	Rhizobium meliloti	7.0000E-13	30	1.5120E-12	Makarieva et al. 2008
318	Unicells	Rhodobacter sphaeroides	8.0000E-13	28	2.4480E-12	Makarieva et al. 2008
319	Unicells	Rhodobacter sphaeroides	8.0000E-13	26	5.0400E-12	Makarieva et al. 2008
320	Unicells	Rhodospirillum rubrum	9.0000E-12	28	6.1560E-12	Makarieva et al. 2008
321	Unicells	Sallinivibrio costicola	4.0000E-13	25	5.0400E-13	Makarieva et al. 2008
322	Unicells	Salmonella typhimurium	6.6000E-13	37	1.7820E-12	Makarieva et al. 2008
323	Unicells	Salmonella typhimurium	6.6000E-13	30	1.1880E-12	Makarieva et al. 2008
324	Unicells	Scenedesmus obliquus	2.2000E-10	20	3.9600E-11	Makarieva et al. 2008
325	Unicells	Scenedesmus quadricauda	7.0000E-11	20	5.5440E-11	Makarieva et al. 2008

326	Unicells	Scenedesmus quadricauda	8.0000E-11	20	5.7600E-11	Makarieva et al. 2008
327	Unicells	Selenastrum capricornutum	5.0000E-11	21	1.1700E-10	Makarieva et al. 2008
328	Unicells	Selenastrum capricornutum	5.0000E-11	21	1.2600E-10	Makarieva et al. 2008
329	Unicells	Selenastrum capricornutum	5.0000E-11	21	1.2600E-10	Makarieva et al. 2008
330	Unicells	Selenastrum capricornutum	5.0000E-11	21	1.2600E-10	Makarieva et al. 2008
331	Unicells	Selenastrum capricornutum	5.0000E-11	21	1.5300E-10	Makarieva et al. 2008
332	Unicells	Selenastrum capricornutum	5.0000E-11	21	1.8900E-10	Makarieva et al. 2008
333	Unicells	Selenastrum capricornutum	5.0000E-11	21	1.8900E-10	Makarieva et al. 2008
334	Unicells	Selenastrum capricornutum	5.0000E-11	21	2.3400E-10	Makarieva et al. 2008
335	Unicells	Selenastrum capricornutum	5.0000E-11	21	5.1300E-10	Makarieva et al. 2008
336	Unicells	Selenastrum minutum	6.9000E-11	20	5.3406E-10	Makarieva et al. 2008
337	Unicells	Selenastrum minutum	7.7000E-11	20	8.8704E-11	Makarieva et al. 2008
338	Unicells	Serratia marcescens	4.0000E-13	37	2.8800E-13	Makarieva et al. 2008
339	Unicells	Serratia marcescens	4.0000E-13	37	1.2960E-12	Makarieva et al. 2008
340	Unicells	Serratia marcescens	4.0000E-13	30	9.3600E-13	Makarieva et al. 2008
341	Unicells	Serratia marcescens	4.0000E-13	30	2.5200E-12	Makarieva et al. 2008
342	Unicells	Skeletonema costatum	6.5000E-11	15	2.4570E-11	Makarieva et al. 2008
343	Unicells	Skeletonema costatum	7.7000E-11	15	3.6036E-11	Makarieva et al. 2008
344	Unicells	Skeletonema costatum	7.9000E-11	15	4.6926E-11	Makarieva et al. 2008
345	Unicells	Skeletonema costatum	8.2000E-11	15	8.7084E-11	Makarieva et al. 2008
346	Unicells	Skeletonema costatum	8.4000E-11	15	1.4062E-10	Makarieva et al. 2008
347	Unicells	Skeletonema costatum	8.8000E-11	15	2.5344E-10	Makarieva et al. 2008
348	Unicells	Skeletonema costatum	9.1000E-11	15	2.6208E-10	Makarieva et al. 2008
349	Unicells	Skeletonema costatum	9.2000E-11	15	2.9808E-10	Makarieva et al. 2008
350	Unicells	Skeletonema costatum	1.2400E-10	15	3.7498E-10	Makarieva et al. 2008
351	Unicells	Skeletonema costatum	1.2400E-10	15	4.9997E-10	Makarieva et al. 2008

352	Unicells	Skeletonema costatum	1.2700E-10	18	1.9660E-10	Makarieva et al. 2008
353	Unicells	Sphaerotilus natans	6.5000E-12	28	5.2650E-11	Makarieva et al. 2008
354	Unicells	Spirulina platensis	1.8000E-10	25	2.9160E-10	Makarieva et al. 2008
355	Unicells	Spirulina platensis	1.8000E-10	25	4.8600E-10	Makarieva et al. 2008
356	Unicells	Spirulina platensis	1.8000E-10	25	6.1560E-10	Makarieva et al. 2008
357	Unicells	Spirulina platensis	1.8000E-10	25	8.1000E-10	Makarieva et al. 2008
358	Unicells	Spirulina platensis P 511	1.8000E-10	30	5.8320E-10	Makarieva et al. 2008
359	Unicells	Spirulina platensis P 511	1.8000E-10	30	8.1000E-10	Makarieva et al. 2008
360	Unicells	Spirulina platensis P 511	1.8000E-10	30	1.1340E-09	Makarieva et al. 2008
361	Unicells	Spirulina platensis P 511	1.8000E-10	30	1.1664E-09	Makarieva et al. 2008
362	Unicells	Sporosarcina ureae	3.8000E-12	30	8.2080E-12	Makarieva et al. 2008
363	Unicells	Staphylococcus aureus	2.7000E-13	37	7.7760E-13	Makarieva et al. 2008
364	Unicells	Staphylococcus aureus	2.7000E-13	30	6.3180E-14	Makarieva et al. 2008
365	Unicells	Staphylococcus aureus	2.7000E-13	37	1.0692E-13	Makarieva et al. 2008
366	Unicells	Staphylococcus aureus	2.7000E-13	37	1.3122E-13	Makarieva et al. 2008
367	Unicells	Staphylococcus aureus	2.7000E-13	37	3.4020E-13	Makarieva et al. 2008
368	Unicells	Staphylococcus aureus	2.7000E-13	30	2.4300E-13	Makarieva et al. 2008
369	Unicells	Staphylococcus epidermidis	5.0000E-13	30	2.4300E-12	Makarieva et al. 2008
370	Unicells	Stephanodiscus neoastraea	1.4000E-08	20	2.1168E-08	Makarieva et al. 2008
371	Unicells	Streptococcus agalactiae	3.0000E-13	37	9.1800E-14	Makarieva et al. 2008
372	Unicells	Streptococcus agalactiae	3.0000E-13	37	4.4820E-13	Makarieva et al. 2008
373	Unicells	Streptococcus pneumoniae	4.0000E-13	30	1.2240E-13	Makarieva et al. 2008
374	Unicells	Streptococcus pyogenes	2.0000E-13	30	1.1880E-13	Makarieva et al. 2008
375	Unicells	Strombidium capitatum	2.0000E-07	15	6.8400E-07	Makarieva et al. 2008
376	Unicells	Sulfolobus acidocalcadareus	4.0000E-13	60	1.0800E-12	Makarieva et al. 2008
377	Unicells	Symbiodinium sp.	5.0000E-10	25	1.3500E-09	Makarieva et al. 2008

378	Unicells	Symbiodinium sp.	5.0000E-10	25	1.9800E-09	Makarieva et al. 2008
379	Unicells	Symbiodinium sp.	5.0000E-10	32	3.6900E-09	Makarieva et al. 2008
380	Unicells	Symbiodinium sp.	5.0000E-10	32	6.9300E-09	Makarieva et al. 2008
381	Unicells	Symbiodinium sp.	5.0000E-10	34	9.8100E-09	Makarieva et al. 2008
382	Unicells	Symbiodinium sp.	5.0000E-10	34	9.8100E-09	Makarieva et al. 2008
383	Unicells	Synechococcus sp. RF-1	1.4000E-11	27	5.0400E-12	Makarieva et al. 2008
384	Unicells	Synechocystis aquatilis	8.0000E-12	30	4.8960E-11	Makarieva et al. 2008
385	Unicells	Synechocystis PCC 6803	8.0000E-12	25	3.4560E-12	Makarieva et al. 2008
386	Unicells	Synechocystis PCC 6803	8.0000E-12	27	3.4560E-11	Makarieva et al. 2008
387	Unicells	Taylorella equigenitalis	4.0000E-13	30	7.2000E-15	Makarieva et al. 2008
388	Unicells	Thalassiosira allenii	3.0000E-10	20	1.4040E-10	Makarieva et al. 2008
389	Unicells	Thalassiosira nordenskioeldii	4.3000E-10	0.5	2.8638E-10	Makarieva et al. 2008
390	Unicells	Thalassiosira nordenskioeldii	4.7000E-10	0.5	1.9458E-10	Makarieva et al. 2008
391	Unicells	Thalassiosira nordenskioeldii	5.0000E-10	0.5	1.8000E-10	Makarieva et al. 2008
392	Unicells	Thalassiosira nordenskioeldii	8.3000E-10	0.5	6.7230E-10	Makarieva et al. 2008
393	Unicells	Thalassiosira pseudonana	7.7000E-11	18	1.7186E-10	Makarieva et al. 2008
394	Unicells	Thalassiosira weissflogii	5.2900E-10	18	1.4378E-09	Makarieva et al. 2008
395	Unicells	Thalassiosira weissflogii	1.1720E-09	18	1.7088E-09	Makarieva et al. 2008
396	Unicells	Thalassiosira weissflogii	1.3640E-09	18	6.3835E-09	Makarieva et al. 2008
397	Unicells	Thalassiosira weissflogii	1.4600E-09	18	4.9932E-09	Makarieva et al. 2008
398	Unicells	Thalassiosira weissflogii	1.4800E-09	18	7.4592E-09	Makarieva et al. 2008
399	Unicells	Thalassiosira weissflogii	1.6750E-09	18	8.4420E-09	Makarieva et al. 2008
400	Unicells	Thiobacillus ferrooxidans	2.5000E-13	25	2.2500E-14	Makarieva et al. 2008
401	Unicells	Thiobacillus intermedius	4.0000E-13	30	7.9200E-13	Makarieva et al. 2008
402	Unicells	Thiobacillus thiooxidans	2.5000E-13	28	6.7500E-14	Makarieva et al. 2008
403	Unicells	Thiocapsa roseopersicina	1.0000E-12	30	1.0080E-12	Makarieva et al. 2008

404	Unicells	Thiocystis violacea	1.1000E-11	30	4.9500E-12	Makarieva et al. 2008
405	Unicells	Trebouxia sp.	1.0000E-09	20	2.5200E-10	Makarieva et al. 2008
406	Unicells	Trebouxia sp.	1.0000E-09	20	4.6800E-10	Makarieva et al. 2008
407	Unicells	Trebouxia sp.	1.0000E-09	20	3.0600E-10	Makarieva et al. 2008
408	Unicells	Unidentified bacterium	6.0000E-13	30	1.1880E-12	Makarieva et al. 2008
409	Unicells	Vibrio alginolyticus	6.0000E-13	30	3.5640E-13	Makarieva et al. 2008
410	Unicells	Vibrio fischeri	6.0000E-13	20	2.3760E-12	Makarieva et al. 2008
411	Unicells	Vibrio metschnikovii	6.0000E-13	30	3.5640E-13	Makarieva et al. 2008
412	Unicells	Vibrio natriegens	1.5000E-12	30	7.1550E-11	Makarieva et al. 2008
413	Unicells	Vibrio parahaemolyticus	6.0000E-13	30	1.8360E-13	Makarieva et al. 2008
414	Unicells	Vibrio sp	1.4000E-13	5	2.7720E-15	Makarieva et al. 2008
415	Unicells	Xanthomonas axonopodis	2.5000E-13	30	1.6650E-12	Makarieva et al. 2008
416	Unicells	Yersinia pestis	5.5000E-13	28	4.7520E-13	Makarieva et al. 2008
417	Invertebrates	Abax	1.2570E-01	8	1.6467E-02	Ehnes et al. 2011
418	Invertebrates	Abax	1.4880E-01	11.5	1.4718E-02	Ehnes et al. 2011
419	Invertebrates	Abax	1.5230E-01	8	1.4718E-02	Ehnes et al. 2011
420	Invertebrates	Abax	1.5520E-01	11.5	1.2970E-02	Ehnes et al. 2011
421	Invertebrates	Abax	1.5680E-01	22	7.7089E-02	Ehnes et al. 2011
422	Invertebrates	Abax	1.5770E-01	22	9.0350E-02	Ehnes et al. 2011
423	Invertebrates	Abax	1.5830E-01	8	1.3552E-02	Ehnes et al. 2011
424	Invertebrates	Abax	1.7030E-01	18.5	2.3170E-02	Ehnes et al. 2011
425	Invertebrates	Abax	1.7460E-01	22	6.2808E-02	Ehnes et al. 2011
426	Invertebrates	Abax	1.7770E-01	8	1.0638E-02	Ehnes et al. 2011
427	Invertebrates	Abax	1.8010E-01	18.5	5.0858E-02	Ehnes et al. 2011
428	Invertebrates	Abax	1.8040E-01	22	6.1205E-02	Ehnes et al. 2011
429	Invertebrates	Abax	1.8560E-01	15	1.4427E-02	Ehnes et al. 2011

430	Invertebrates	Abax	1.9760E-01	22	1.1906E-01	Ehnes et al. 2011
431	Invertebrates	Abax	2.1480E-01	18.5	2.5939E-02	Ehnes et al. 2011
432	Invertebrates	Abax	2.1550E-01	11.5	2.4190E-02	Ehnes et al. 2011
433	Invertebrates	Abax	2.1650E-01	15	2.1130E-02	Ehnes et al. 2011
434	Invertebrates	Abax	2.4560E-01	22	1.0755E-01	Ehnes et al. 2011
435	Invertebrates	Abax	2.4900E-01	15	1.5301E-02	Ehnes et al. 2011
436	Invertebrates	Abax	2.4950E-01	8	1.6758E-02	Ehnes et al. 2011
437	Invertebrates	Abax	2.5980E-01	11.5	2.6376E-02	Ehnes et al. 2011
438	Invertebrates	Abax	2.6550E-01	15	5.4647E-02	Ehnes et al. 2011
439	Invertebrates	Abax	2.6810E-01	18.5	5.4064E-02	Ehnes et al. 2011
440	Invertebrates	Abax	2.8350E-01	11.5	2.7542E-02	Ehnes et al. 2011
441	Invertebrates	Abax	2.8520E-01	18.5	3.4246E-02	Ehnes et al. 2011
442	Invertebrates	Abax	2.9590E-01	11.5	2.0984E-02	Ehnes et al. 2011
443	Invertebrates	Abax	2.9750E-01	11.5	2.3608E-02	Ehnes et al. 2011
444	Invertebrates	Abax	2.9840E-01	8	1.6176E-02	Ehnes et al. 2011
445	Invertebrates	Abax	2.9940E-01	22	5.6104E-02	Ehnes et al. 2011
446	Invertebrates	Abax	2.9950E-01	18.5	4.2989E-02	Ehnes et al. 2011
447	Invertebrates	Abax	3.0290E-01	18.5	6.4411E-02	Ehnes et al. 2011
448	Invertebrates	Abax	3.0310E-01	15	3.5557E-02	Ehnes et al. 2011
449	Invertebrates	Abax	3.1000E-01	15	3.2060E-02	Ehnes et al. 2011
450	Invertebrates	Abax	3.1530E-01	15	8.5541E-02	Ehnes et al. 2011
451	Invertebrates	Abax	3.1720E-01	18.5	6.4556E-02	Ehnes et al. 2011
452	Invertebrates	Abax	3.4820E-01	18.5	7.0385E-02	Ehnes et al. 2011
453	Invertebrates	Abax	3.4870E-01	22	7.2426E-02	Ehnes et al. 2011
454	Invertebrates	Abax	3.6300E-01	15	3.8909E-02	Ehnes et al. 2011
455	Invertebrates	Abax ovalis	1.5460E-01	15	1.6030E-02	Ehnes et al. 2011

456	Invertebrates	Abax ovalis	1.5507E-01	15	1.2678E-02	Ehnes et al. 2011
457	Invertebrates	Abax ovalis	1.6881E-01	15	1.6613E-02	Ehnes et al. 2011
458	Invertebrates	Abax ovalis	2.4460E-01	15	1.6904E-02	Ehnes et al. 2011
459	Invertebrates	Abax ovalis	2.5442E-01	15	2.2150E-02	Ehnes et al. 2011
460	Invertebrates	Abax paralellepipedus	1.3151E-01	15	1.5738E-02	Ehnes et al. 2011
461	Invertebrates	Abax paralellepipedus	1.3921E-01	15	1.3115E-02	Ehnes et al. 2011
462	Invertebrates	Abax paralellepipedus	1.8185E-01	20	2.2733E-02	Ehnes et al. 2011
463	Invertebrates	Abax paralellepipedus	2.1520E-01	25	3.9054E-02	Ehnes et al. 2011
464	Invertebrates	Abax paralellepipedus	2.2019E-01	25	3.5994E-02	Ehnes et al. 2011
465	Invertebrates	Abax paralellepipedus	2.2430E-01	25	5.2898E-02	Ehnes et al. 2011
466	Invertebrates	Abax paralellepipedus	2.2706E-01	10	1.0201E-02	Ehnes et al. 2011
467	Invertebrates	Abax paralellepipedus	2.3209E-01	15	2.6668E-02	Ehnes et al. 2011
468	Invertebrates	Abax paralellepipedus	2.4043E-01	25	4.6632E-02	Ehnes et al. 2011
469	Invertebrates	Abax paralellepipedus	2.4116E-01	25	5.2607E-02	Ehnes et al. 2011
470	Invertebrates	Abax paralellepipedus	2.4157E-01	25	6.6159E-02	Ehnes et al. 2011
471	Invertebrates	Abax paralellepipedus	2.4160E-01	20	5.8436E-02	Ehnes et al. 2011
472	Invertebrates	Abax paralellepipedus	2.4449E-01	25	5.6542E-02	Ehnes et al. 2011
473	Invertebrates	Abax paralellepipedus	2.4473E-01	20	8.7290E-02	Ehnes et al. 2011
474	Invertebrates	Abax paralellepipedus	2.4867E-01	25	4.8527E-02	Ehnes et al. 2011
475	Invertebrates	Abax paralellepipedus	2.5097E-01	10	1.3990E-02	Ehnes et al. 2011
476	Invertebrates	Abax paralellepipedus	2.5181E-01	30	9.8948E-02	Ehnes et al. 2011
477	Invertebrates	Abax paralellepipedus	2.5206E-01	15	4.0366E-02	Ehnes et al. 2011
478	Invertebrates	Abax paralellepipedus	2.5582E-01	15	1.9527E-02	Ehnes et al. 2011
479	Invertebrates	Abax paralellepipedus	2.5790E-01	10	1.5738E-02	Ehnes et al. 2011
480	Invertebrates	Abax paralellepipedus	2.5795E-01	10	1.4427E-02	Ehnes et al. 2011
481	Invertebrates	Abax paralellepipedus	2.5844E-01	15	1.8653E-02	Ehnes et al. 2011

482	Invertebrates	Abax paralellepipedus	2.5875E-01	25	1.0580E-01	Ehnes et al. 2011
483	Invertebrates	Abax paralellepipedus	2.6360E-01	20	2.7834E-02	Ehnes et al. 2011
484	Invertebrates	Abax paralellepipedus	2.6667E-01	20	3.2497E-02	Ehnes et al. 2011
485	Invertebrates	Abax paralellepipedus	2.6716E-01	10	1.5301E-02	Ehnes et al. 2011
486	Invertebrates	Abax paralellepipedus	2.7082E-01	30	1.0099E-01	Ehnes et al. 2011
487	Invertebrates	Abax paralellepipedus	2.7147E-01	20	1.0609E-01	Ehnes et al. 2011
488	Invertebrates	Abax paralellepipedus	2.7244E-01	15	2.3025E-02	Ehnes et al. 2011
489	Invertebrates	Abax paralellepipedus	2.7292E-01	15	2.3753E-02	Ehnes et al. 2011
490	Invertebrates	Abax paralellepipedus	2.7553E-01	30	1.0070E-01	Ehnes et al. 2011
491	Invertebrates	Abax paralellepipedus	2.7610E-01	15	6.6305E-02	Ehnes et al. 2011
492	Invertebrates	Abax paralellepipedus	2.7674E-01	20	1.9382E-02	Ehnes et al. 2011
493	Invertebrates	Abax paralellepipedus	2.7921E-01	30	8.5395E-02	Ehnes et al. 2011
494	Invertebrates	Abax paralellepipedus	2.8135E-01	30	9.7199E-02	Ehnes et al. 2011
495	Invertebrates	Abax paralellepipedus	2.8176E-01	30	8.7872E-02	Ehnes et al. 2011
496	Invertebrates	Abax paralellepipedus	2.8351E-01	10	1.5447E-02	Ehnes et al. 2011
497	Invertebrates	Abax paralellepipedus	2.8471E-01	15	3.7160E-02	Ehnes et al. 2011
498	Invertebrates	Abax paralellepipedus	2.8495E-01	10	1.8070E-02	Ehnes et al. 2011
499	Invertebrates	Abax paralellepipedus	2.8672E-01	20	5.4939E-02	Ehnes et al. 2011
500	Invertebrates	Abax paralellepipedus	2.8939E-01	10	2.4773E-02	Ehnes et al. 2011
501	Invertebrates	Abax paralellepipedus	3.0065E-01	10	1.3115E-02	Ehnes et al. 2011
502	Invertebrates	Abax paralellepipedus	3.1059E-01	20	4.1678E-02	Ehnes et al. 2011
503	Invertebrates	Abax paralellepipedus	3.2343E-01	30	1.0011E-01	Ehnes et al. 2011
504	Invertebrates	Abax paralellepipedus	3.3285E-01	15	5.4939E-02	Ehnes et al. 2011
505	Invertebrates	Abax paralellepipedus	3.6740E-01	15	9.0641E-02	Ehnes et al. 2011
506	Invertebrates	Abax paralellepipedus	3.7643E-01	20	1.4558E-01	Ehnes et al. 2011
507	Invertebrates	Abax paralellepipedus	3.8075E-01	15	3.2934E-02	Ehnes et al. 2011

508	Invertebrates	Abax paralellepipedus	3.9045E-01	20	1.4063E-01	Ehnes et al. 2011
509	Invertebrates	Acanthephyra acutifrons	8.6400E+00	10	3.2815E-01	Makarieva et al. 2008
510	Invertebrates	Acanthephyra acutifrons	8.6400E+00	5	3.2037E-01	Makarieva et al. 2008
511	Invertebrates	Acanthephyra curtirostris	2.2900E+00	10	9.6455E-03	Makarieva et al. 2008
512	Invertebrates	Acanthephyra curtirostris	2.2900E+00	5	9.3157E-03	Makarieva et al. 2008
513	Invertebrates	Acanthephyra curtirostris	3.5300E+00	5.5	5.2103E-01	Makarieva et al. 2008
514	Invertebrates	Acanthephyra curtirostris	3.5300E+00	5.5	2.4145E-02	Makarieva et al. 2008
515	Invertebrates	Acanthephyra smithi	3.8400E+00	10	4.0090E-01	Makarieva et al. 2008
516	Invertebrates	Acanthephyra smithi	3.8400E+00	20	9.6768E-01	Makarieva et al. 2008
517	Invertebrates	Acanthephyra smithi	3.8400E+00	5	6.2899E-01	Makarieva et al. 2008
518	Invertebrates	Acanthoderes circumflexa	1.3900E-01	25	4.3524E-02	Ehnes et al. 2011
519	Invertebrates	Acanthophorus confinis	3.4900E+00	25	1.1126E+00	Ehnes et al. 2011
520	Invertebrates	Acartia clausi	2.1000E-05	5	1.2852E-05	Makarieva et al. 2008
521	Invertebrates	Acartia clausi	2.2000E-05	5.9	1.6632E-05	Makarieva et al. 2008
522	Invertebrates	Acartia clausi	2.4000E-05	5	1.5552E-05	Makarieva et al. 2008
523	Invertebrates	Acartia clausi	2.5000E-05	5.9	9.0000E-06	Makarieva et al. 2008
524	Invertebrates	Acartia clausi	2.7500E-05	15	1.2999E-05	Makarieva et al. 2008
525	Invertebrates	Acartia clausi	3.6000E-05	5	2.8512E-05	Makarieva et al. 2008
526	Invertebrates	Acartia clausi	3.7000E-05	5.6	2.8305E-05	Makarieva et al. 2008
527	Invertebrates	Acartia clausi	4.2500E-05	14.8	3.1365E-05	Makarieva et al. 2008
528	Invertebrates	Acartia latisetosa	2.9700E-05	20	4.4372E-05	Makarieva et al. 2008
529	Invertebrates	Acartia longiremis	4.0500E-05	9.6	2.7994E-05	Makarieva et al. 2008
530	Invertebrates	Acartia pacifica	3.9000E-05	26	4.6332E-05	Makarieva et al. 2008
531	Invertebrates	Acartia tonsa	3.5000E-05	22	5.6070E-05	Makarieva et al. 2008
532	Invertebrates	Achaeta	2.0000E-05	20	1.1100E-05	Ehnes et al. 2011
533	Invertebrates	Achaeta	5.2000E-05	20	2.5400E-05	Ehnes et al. 2011

534	Invertebrates	Achaeta	1.1400E-04	20	3.4450E-05	Ehnes et al. 2011
535	Invertebrates	Achaeta	1.7700E-04	20	3.1700E-05	Ehnes et al. 2011
536	Invertebrates	Acheta domesticus	3.6900E-01	25	2.0495E-01	Ehnes et al. 2011
537	Invertebrates	Achipteria coleoptrata	4.0500E-05	5	4.7000E-06	Ehnes et al. 2011
538	Invertebrates	Achipteria coleoptrata	4.0500E-05	10	6.3000E-06	Ehnes et al. 2011
539	Invertebrates	Achipteria coleoptrata	4.0500E-05	15	1.8150E-05	Ehnes et al. 2011
540	Invertebrates	Achipteria coleoptrata	4.0500E-05	20	2.4700E-05	Ehnes et al. 2011
541	Invertebrates	Achipteria holomonensis	1.0000E-05	5	1.4000E-06	Ehnes et al. 2011
542	Invertebrates	Achipteria holomonensis	1.0000E-05	10	3.3500E-06	Ehnes et al. 2011
543	Invertebrates	Achipteria holomonensis	1.0000E-05	15	6.1000E-06	Ehnes et al. 2011
544	Invertebrates	Achipteria oudemansi	3.7000E-05	7	1.3000E-06	Ehnes et al. 2011
545	Invertebrates	Achipteria oudemansi	3.7000E-05	11	1.4000E-06	Ehnes et al. 2011
546	Invertebrates	Achipteria oudemansi	3.7000E-05	15	2.6500E-06	Ehnes et al. 2011
547	Invertebrates	Achipteria oudemansi	3.7000E-05	19	4.4000E-06	Ehnes et al. 2011
548	Invertebrates	Achipteria oudemansi	3.7000E-05	24	4.4500E-06	Ehnes et al. 2011
549	Invertebrates	Achipteria oudemansi	3.7000E-05	29	4.6000E-06	Ehnes et al. 2011
550	Invertebrates	Aciculioiditermes sp. A	1.8000E-03	25	3.2400E-04	Ehnes et al. 2011
551	Invertebrates	Acrocinus longimanus	5.3830E+00	25	2.9818E+00	Ehnes et al. 2011
552	Invertebrates	Adeloneivaia boisduvalii	1.0340E+00	25	3.2321E-01	Ehnes et al. 2011
553	Invertebrates	Adeloneivaia subungulata	4.8700E-01	25	3.3302E-01	Ehnes et al. 2011
554	Invertebrates	Adesmia baccata	4.0700E-01	25	3.2256E-02	Ehnes et al. 2011
555	Invertebrates	Adoristes ovatus	3.4500E-05	5	3.3000E-06	Ehnes et al. 2011
556	Invertebrates	Adoristes ovatus	3.4500E-05	10	5.4000E-06	Ehnes et al. 2011
557	Invertebrates	Adoristes ovatus	3.4500E-05	15	9.8500E-06	Ehnes et al. 2011
558	Invertebrates	Adoristes ovatus	3.4500E-05	20	1.7200E-05	Ehnes et al. 2011
559	Invertebrates	Aedes campestris	6.7200E-03	25	5.3100E-02	Ehnes et al. 2011

560	Invertebrates	Aega sp.	2.6000E-02	17	1.2196E-02	Makarieva et al. 2008
561	Invertebrates	Aegina citrea	1.9000E+00	5	7.8660E-03	Makarieva et al. 2008
562	Invertebrates	Aglantha digitale	2.5000E-01	1.1	2.2950E-03	Makarieva et al. 2008
563	Invertebrates	Aglelenopsis aperta	5.5600E-01	22	1.2350E-01	Ehnes et al. 2011
564	Invertebrates	Agrypnus bocandei	1.6620E-01	25	1.2888E-02	Ehnes et al. 2011
565	Invertebrates	Alaskozetes antarcticus	1.3290E-05	0	1.6000E-06	Ehnes et al. 2011
566	Invertebrates	Alaskozetes antarcticus	1.3290E-05	5	2.0000E-06	Ehnes et al. 2011
567	Invertebrates	Alaskozetes antarcticus	1.3290E-05	10	2.1500E-06	Ehnes et al. 2011
568	Invertebrates	Alaskozetes antarcticus	2.5990E-05	0	1.9500E-06	Ehnes et al. 2011
569	Invertebrates	Alaskozetes antarcticus	2.5990E-05	5	4.1500E-06	Ehnes et al. 2011
570	Invertebrates	Alaskozetes antarcticus	2.5990E-05	10	7.2500E-06	Ehnes et al. 2011
571	Invertebrates	Alaskozetes antarcticus	4.6080E-05	0	5.9000E-06	Ehnes et al. 2011
572	Invertebrates	Alaskozetes antarcticus	4.6080E-05	5	8.0000E-06	Ehnes et al. 2011
573	Invertebrates	Alaskozetes antarcticus	4.6080E-05	10	1.4150E-05	Ehnes et al. 2011
574	Invertebrates	Alaskozetes antarcticus	1.2665E-04	0	1.3150E-05	Ehnes et al. 2011
575	Invertebrates	Alaskozetes antarcticus	1.2665E-04	5	2.1950E-05	Ehnes et al. 2011
576	Invertebrates	Alaskozetes antarcticus	1.2665E-04	10	3.0100E-05	Ehnes et al. 2011
577	Invertebrates	Alaskozetes antarcticus	1.5697E-04	0	1.2400E-05	Ehnes et al. 2011
578	Invertebrates	Alaskozetes antarcticus	1.5697E-04	5	2.7750E-05	Ehnes et al. 2011
579	Invertebrates	Alaskozetes antarcticus	1.5697E-04	10	3.5650E-05	Ehnes et al. 2011
580	Invertebrates	Alaskozetes antarcticus	1.6802E-04	0	1.0600E-05	Ehnes et al. 2011
581	Invertebrates	Alaskozetes antarcticus	1.6802E-04	5	1.9000E-05	Ehnes et al. 2011
582	Invertebrates	Alaskozetes antarcticus	1.8767E-04	0	9.6500E-06	Ehnes et al. 2011
583	Invertebrates	Alaskozetes antarcticus	1.8767E-04	5	2.0850E-05	Ehnes et al. 2011
584	Invertebrates	Alaskozetes antarcticus	1.8767E-04	10	4.2700E-05	Ehnes et al. 2011
585	Invertebrates	Alaskozetes antarcticus	1.9485E-04	10	3.6500E-05	Ehnes et al. 2011

586	Invertebrates	Alaskozetes antarcticus	1.9621E-04	0	9.1500E-06	Ehnes et al. 2011
587	Invertebrates	Alaskozetes antarcticus	1.9621E-04	5	2.1500E-05	Ehnes et al. 2011
588	Invertebrates	Alaskozetes antarcticus	1.9621E-04	10	4.4650E-05	Ehnes et al. 2011
589	Invertebrates	Alaskozetes antarcticus	2.1272E-04	5	3.8900E-05	Ehnes et al. 2011
590	Invertebrates	Alaskozetes antarcticus	2.1371E-04	10	3.8600E-05	Ehnes et al. 2011
591	Invertebrates	Alaskozetes antarcticus	2.1418E-04	5	3.6100E-05	Ehnes et al. 2011
592	Invertebrates	Allacma fusca	2.2500E-03	18	1.1150E-03	Ehnes et al. 2011
593	Invertebrates	Allacma fusca	2.9000E-03	18	1.4400E-03	Ehnes et al. 2011
594	Invertebrates	Allolobophora caliginosa	3.3500E-01	6	2.3050E-02	Ehnes et al. 2011
595	Invertebrates	Allolobophora caliginosa	3.3500E-01	10	3.4600E-02	Ehnes et al. 2011
596	Invertebrates	Allolobophora caliginosa	3.3500E-01	15	4.3950E-02	Ehnes et al. 2011
597	Invertebrates	Allolobophora caliginosa	8.7500E-01	19	7.0000E-02	Ehnes et al. 2011
598	Invertebrates	Alopecosa juv.	8.0000E-04	15	1.3116E-03	Ehnes et al. 2011
599	Invertebrates	Alopecosa juv.	9.0000E-04	15	7.2865E-04	Ehnes et al. 2011
600	Invertebrates	Alopecosa juv.	1.1000E-03	15	1.4573E-03	Ehnes et al. 2011
601	Invertebrates	Alopecosa juv.	1.2000E-03	15	2.1859E-03	Ehnes et al. 2011
602	Invertebrates	Alopecosa juv.	1.5000E-03	18.5	3.0603E-03	Ehnes et al. 2011
603	Invertebrates	Alopecosa juv.	1.7000E-03	11.5	2.1859E-03	Ehnes et al. 2011
604	Invertebrates	Alopecosa juv.	1.7000E-03	18.5	2.3316E-03	Ehnes et al. 2011
605	Invertebrates	Alopecosa juv.	1.8000E-03	11.5	1.1658E-03	Ehnes et al. 2011
606	Invertebrates	Alopecosa juv.	1.8000E-03	11.5	1.0201E-03	Ehnes et al. 2011
607	Invertebrates	Alopecosa juv.	1.8000E-03	18.5	2.0402E-03	Ehnes et al. 2011
608	Invertebrates	Alopecosa juv.	1.8000E-03	18.5	1.8945E-03	Ehnes et al. 2011
609	Invertebrates	Alopecosa juv.	2.1000E-03	18.5	8.7435E-04	Ehnes et al. 2011
610	Invertebrates	Alopecosa kochi	1.0000E-01	30	3.6350E-02	Ehnes et al. 2011
611	Invertebrates	Alopecosa kochi	1.0000E-01	20	1.7150E-02	Ehnes et al. 2011

612	Invertebrates	Alopecosa spec	2.8000E-02	15	5.2461E-03	Ehnes et al. 2011
613	Invertebrates	Alopecosa spec	2.8000E-02	22	1.1658E-02	Ehnes et al. 2011
614	Invertebrates	Alopecosa spec	3.1000E-02	22	1.2824E-02	Ehnes et al. 2011
615	Invertebrates	Alopecosa spec	3.4000E-02	8	2.9145E-04	Ehnes et al. 2011
616	Invertebrates	Alopecosa spec	3.4000E-02	15	9.1807E-03	Ehnes et al. 2011
617	Invertebrates	Alopecosa spec	3.4000E-02	22	1.3407E-02	Ehnes et al. 2011
618	Invertebrates	Alopecosa spec	3.6000E-02	8	2.3316E-03	Ehnes et al. 2011
619	Invertebrates	Alopecosa spec	3.6000E-02	15	7.2863E-03	Ehnes et al. 2011
620	Invertebrates	Alopecosa spec	3.6000E-02	22	1.8361E-02	Ehnes et al. 2011
621	Invertebrates	Alopecosa spec	3.7000E-02	8	3.2060E-03	Ehnes et al. 2011
622	Invertebrates	Alopecosa spec	3.7000E-02	15	5.9748E-03	Ehnes et al. 2011
623	Invertebrates	Alopecosa spec	3.7000E-02	22	1.2678E-02	Ehnes et al. 2011
624	Invertebrates	Alopecosa spec	3.8000E-02	22	4.8090E-03	Ehnes et al. 2011
625	Invertebrates	Alopecosa spec	3.9000E-02	8	3.9346E-03	Ehnes et al. 2011
626	Invertebrates	Alopecosa spec	3.9000E-02	15	1.1075E-02	Ehnes et al. 2011
627	Invertebrates	Alopecosa spec	3.9000E-02	22	1.5738E-02	Ehnes et al. 2011
628	Invertebrates	Alopecosa spec	4.0000E-02	15	3.9346E-03	Ehnes et al. 2011
629	Invertebrates	Alopecosa spec	4.0000E-02	22	1.1804E-02	Ehnes et al. 2011
630	Invertebrates	Alopecosa spec	4.2000E-02	15	6.9948E-03	Ehnes et al. 2011
631	Invertebrates	Alopecosa spec	4.2000E-02	22	2.1276E-02	Ehnes et al. 2011
632	Invertebrates	Alopecosa spec	4.4000E-02	8	2.7688E-03	Ehnes et al. 2011
633	Invertebrates	Alopecosa spec	4.4000E-02	22	1.4281E-02	Ehnes et al. 2011
634	Invertebrates	Alopecosa spec	4.8000E-02	22	1.4135E-02	Ehnes et al. 2011
635	Invertebrates	Alopecosa spec	5.0500E-02	11.5	8.1607E-03	Ehnes et al. 2011
636	Invertebrates	Alopecosa spec	5.0500E-02	18.5	9.6179E-03	Ehnes et al. 2011
637	Invertebrates	Alopecosa spec	5.2000E-02	15	7.8692E-03	Ehnes et al. 2011

638	Invertebrates	Alopecosa spec	5.2000E-02	22	1.8653E-02	Ehnes et al. 2011
639	Invertebrates	Alopecosa spec	5.7000E-02	8	5.5376E-03	Ehnes et al. 2011
640	Invertebrates	Alopecosa spec	5.7000E-02	15	3.9346E-03	Ehnes et al. 2011
641	Invertebrates	Alopecosa spec	5.7000E-02	15	7.1406E-03	Ehnes et al. 2011
642	Invertebrates	Alopecosa spec	5.7000E-02	15	8.1607E-03	Ehnes et al. 2011
643	Invertebrates	Alopecosa spec	5.7000E-02	22	1.1367E-02	Ehnes et al. 2011
644	Invertebrates	Alopecosa spec	5.7000E-02	22	1.8799E-02	Ehnes et al. 2011
645	Invertebrates	Alopecosa spec	5.8000E-02	8	3.3517E-03	Ehnes et al. 2011
646	Invertebrates	Alopecosa spec	5.8000E-02	15	4.9547E-03	Ehnes et al. 2011
647	Invertebrates	Alopecosa spec	5.8000E-02	22	1.6321E-02	Ehnes et al. 2011
648	Invertebrates	Alopecosa spec	6.2000E-02	22	1.5738E-02	Ehnes et al. 2011
649	Invertebrates	Alopecosa spec	9.3500E-02	11.5	1.1658E-02	Ehnes et al. 2011
650	Invertebrates	Alopecosa spec	9.3500E-02	18.5	1.2095E-02	Ehnes et al. 2011
651	Invertebrates	Amara quenseli	1.5000E-02	25	2.5200E-04	Ehnes et al. 2011
652	Invertebrates	Anachalcos convexus	1.4210E+00	25	2.7716E-01	Ehnes et al. 2011
653	Invertebrates	Anax junius	1.0190E+00	25	7.4671E-01	Ehnes et al. 2011
654	Invertebrates	Anconia integra	8.1670E-01	25	4.7594E-01	Ehnes et al. 2011
655	Invertebrates	Anepsius brunneus	6.1500E-03	25	4.4640E-03	Ehnes et al. 2011
656	Invertebrates	Anomala sp.	1.0000E-01	25	2.4660E-02	Ehnes et al. 2011
657	Invertebrates	Anoplolepis steinergroeveri	9.4000E-04	20	1.0080E-03	Ehnes et al. 2011
658	Invertebrates	Anoplolepis steinergroeveri	9.8000E-04	20	3.2400E-04	Ehnes et al. 2011
659	Invertebrates	Anoplolepis steinergroeveri	1.1500E-03	20	4.1400E-04	Ehnes et al. 2011
660	Invertebrates	Anoplolepis steinergroeveri	1.1700E-03	20	6.4800E-04	Ehnes et al. 2011
661	Invertebrates	Anoplolepis steinergroeveri	1.5900E-03	20	6.3000E-04	Ehnes et al. 2011
662	Invertebrates	Anoplolepis steinergroeveri	1.9000E-03	20	6.6600E-04	Ehnes et al. 2011
663	Invertebrates	Anoplolepis steinergroeveri	2.9000E-03	20	1.5840E-03	Ehnes et al. 2011

664	Invertebrates	Anoplolepis steinergroeveri	3.3200E-03	20	1.3860E-03	Ehnes et al. 2011
665	Invertebrates	Anoplolepis steinergroeveri	4.3000E-03	20	2.0160E-03	Ehnes et al. 2011
666	Invertebrates	Anoplolepis steinergroeveri	4.6400E-03	20	1.2420E-03	Ehnes et al. 2011
667	Invertebrates	Anoplolepis steinergroeveri	4.7400E-03	20	1.6200E-03	Ehnes et al. 2011
668	Invertebrates	Anoplolepis steinergroeveri	4.8700E-03	20	1.6380E-03	Ehnes et al. 2011
669	Invertebrates	Anoplolepis steinergroeveri	4.9400E-03	25	2.5380E-03	Ehnes et al. 2011
670	Invertebrates	Anoplolepis steinergroeveri	5.5800E-03	20	1.7640E-03	Ehnes et al. 2011
671	Invertebrates	Anoplolepis steinergroeveri	6.0200E-03	20	2.3400E-03	Ehnes et al. 2011
672	Invertebrates	Anoplolepis steinergroeveri	6.5800E-03	20	2.3940E-03	Ehnes et al. 2011
673	Invertebrates	Anoplolepis steinergroeveri	6.6000E-03	20	2.1600E-03	Ehnes et al. 2011
674	Invertebrates	Anoplolepis steinergroeveri	6.9500E-03	20	2.3580E-03	Ehnes et al. 2011
675	Invertebrates	Anoplolepis steinergroeveri	6.9700E-03	20	1.4220E-03	Ehnes et al. 2011
676	Invertebrates	Anoplolepis steinergroeveri	7.1100E-03	20	1.2960E-03	Ehnes et al. 2011
677	Invertebrates	Anoplolepis steinergroeveri	7.2800E-03	20	1.6380E-03	Ehnes et al. 2011
678	Invertebrates	Anoplolepis steinergroeveri	7.3300E-03	20	1.7640E-03	Ehnes et al. 2011
679	Invertebrates	Anoplolepis steinergroeveri	7.4800E-03	20	1.2420E-03	Ehnes et al. 2011
680	Invertebrates	Anoplolepis steinergroeveri	7.8700E-03	20	2.6460E-03	Ehnes et al. 2011
681	Invertebrates	Anoplolepis steinergroeveri	8.3000E-03	20	2.9520E-03	Ehnes et al. 2011
682	Invertebrates	Anoplolepis steinergroeveri	8.8000E-03	20	1.3500E-03	Ehnes et al. 2011
683	Invertebrates	Anoplolepis steinergroeveri	8.9000E-03	20	1.7460E-03	Ehnes et al. 2011
684	Invertebrates	Anoplolepis steinergroeveri	1.0240E-02	20	1.6920E-03	Ehnes et al. 2011
685	Invertebrates	Anoplolepis steinergroeveri	1.0480E-02	20	2.2500E-03	Ehnes et al. 2011
686	Invertebrates	Antarctomysis maxima	7.2100E-01	20	1.4743E-01	Makarieva et al. 2008
687	Invertebrates	Antheraea pernyi	1.2100E+00	25	1.1042E+00	Ehnes et al. 2011
688	Invertebrates	Anthia fabricii	2.2500E+00	25	3.3752E-01	Ehnes et al. 2011
689	Invertebrates	Antrops truncipennis	2.2100E-03	25	9.2880E-03	Ehnes et al. 2011

690	Invertebrates	Anurogryllis arboreus	3.1000E-01	25	9.9162E-02	Ehnes et al. 2011
691	Invertebrates	Anuropus bathypelagicus	3.4500E+00	5.5	1.4283E-02	Makarieva et al. 2008
692	Invertebrates	Anuropus bathypelagicus	3.4500E+00	5.5	8.0109E-02	Makarieva et al. 2008
693	Invertebrates	Apetaloides firmiana	1.6900E-01	25	1.4909E-01	Ehnes et al. 2011
694	Invertebrates	Aphaenogaster cockerelli	4.7200E-03	25	1.4850E-02	Ehnes et al. 2011
695	Invertebrates	Aphodius contaminatus	1.4000E-02	25	1.8666E-02	Ehnes et al. 2011
696	Invertebrates	Aphodius distinctus	7.8000E-03	25	1.8882E-02	Ehnes et al. 2011
697	Invertebrates	Aphodius fimetarius	3.0900E-02	25	3.3714E-02	Ehnes et al. 2011
698	Invertebrates	Aphodius fossor	1.2130E-01	25	4.8384E-02	Ehnes et al. 2011
699	Invertebrates	Aphodius prodromus	1.3800E-02	25	3.0060E-02	Ehnes et al. 2011
700	Invertebrates	Aphodius rufipes	8.4000E-02	25	6.8922E-02	Ehnes et al. 2011
701	Invertebrates	Aphodius rufus	2.0900E-01	25	3.0672E-02	Ehnes et al. 2011
702	Invertebrates	Apis mellifera ligustica	9.4400E-02	25	1.0924E-01	Ehnes et al. 2011
703	Invertebrates	Aporectodea caliginosa	1.8350E-02	15	4.6632E-03	Ehnes et al. 2011
704	Invertebrates	Aporectodea caliginosa	2.1910E-02	10	1.4573E-03	Ehnes et al. 2011
705	Invertebrates	Aporectodea caliginosa	4.1900E-02	5	7.2865E-04	Ehnes et al. 2011
706	Invertebrates	Aporectodea caliginosa	5.4120E-02	5	1.1658E-03	Ehnes et al. 2011
707	Invertebrates	Aporectodea caliginosa	5.9430E-02	5	5.8290E-04	Ehnes et al. 2011
708	Invertebrates	Aporectodea caliginosa	6.0880E-02	24.9	1.0492E-02	Ehnes et al. 2011
709	Invertebrates	Aporectodea caliginosa	6.8180E-02	10.6	4.9547E-03	Ehnes et al. 2011
710	Invertebrates	Aporectodea caliginosa	6.9260E-02	15	7.1406E-03	Ehnes et al. 2011
711	Invertebrates	Aporectodea caliginosa	7.4310E-02	30	1.5301E-02	Ehnes et al. 2011
712	Invertebrates	Aporectodea caliginosa	7.6050E-02	20	5.3919E-03	Ehnes et al. 2011
713	Invertebrates	Aporectodea caliginosa	7.9430E-02	20	5.6833E-03	Ehnes et al. 2011
714	Invertebrates	Aporectodea caliginosa	7.9490E-02	5	1.8945E-03	Ehnes et al. 2011
715	Invertebrates	Aporectodea caliginosa	8.1740E-02	20	6.9948E-03	Ehnes et al. 2011

716	Invertebrates	Aporectodea caliginosa	8.4380E-02	5	3.9346E-03	Ehnes et al. 2011
717	Invertebrates	Aporectodea caliginosa	8.8890E-02	24.9	1.1512E-02	Ehnes et al. 2011
718	Invertebrates	Aporectodea caliginosa	9.6610E-02	15	8.8893E-03	Ehnes et al. 2011
719	Invertebrates	Aporectodea caliginosa	1.0320E-01	20	1.0929E-02	Ehnes et al. 2011
720	Invertebrates	Aporectodea caliginosa	1.0474E-01	30	1.6904E-02	Ehnes et al. 2011
721	Invertebrates	Aporectodea caliginosa	1.0685E-01	25	1.5593E-02	Ehnes et al. 2011
722	Invertebrates	Aporectodea caliginosa	1.0728E-01	10	4.3718E-03	Ehnes et al. 2011
723	Invertebrates	Aporectodea caliginosa	1.0737E-01	30	2.0110E-02	Ehnes et al. 2011
724	Invertebrates	Aporectodea caliginosa	1.0928E-01	10.6	7.8692E-03	Ehnes et al. 2011
725	Invertebrates	Aporectodea caliginosa	1.1001E-01	5	1.1658E-03	Ehnes et al. 2011
726	Invertebrates	Aporectodea caliginosa	1.1279E-01	5	9.4722E-03	Ehnes et al. 2011
727	Invertebrates	Aporectodea caliginosa	1.2160E-01	25	9.7636E-03	Ehnes et al. 2011
728	Invertebrates	Aporectodea caliginosa	1.2205E-01	25	1.1950E-02	Ehnes et al. 2011
729	Invertebrates	Aporectodea caliginosa	1.2278E-01	10	5.5376E-03	Ehnes et al. 2011
730	Invertebrates	Aporectodea caliginosa	1.3049E-01	10	4.8090E-03	Ehnes et al. 2011
731	Invertebrates	Aporectodea caliginosa	1.3058E-01	10	5.8290E-03	Ehnes et al. 2011
732	Invertebrates	Aporectodea caliginosa	1.3372E-01	15	1.2824E-02	Ehnes et al. 2011
733	Invertebrates	Aporectodea caliginosa	1.4095E-01	5	1.4573E-03	Ehnes et al. 2011
734	Invertebrates	Aporectodea caliginosa	1.4576E-01	25	1.3115E-02	Ehnes et al. 2011
735	Invertebrates	Aporectodea caliginosa	1.5343E-01	20	1.3990E-02	Ehnes et al. 2011
736	Invertebrates	Aporectodea caliginosa	1.5700E-01	5	4.0803E-03	Ehnes et al. 2011
737	Invertebrates	Aporectodea caliginosa	1.5715E-01	10.6	7.8692E-03	Ehnes et al. 2011
738	Invertebrates	Aporectodea caliginosa	1.6267E-01	5	6.2662E-03	Ehnes et al. 2011
739	Invertebrates	Aporectodea caliginosa	1.6427E-01	5	2.7688E-03	Ehnes et al. 2011
740	Invertebrates	Aporectodea caliginosa	1.6555E-01	15	1.0492E-02	Ehnes et al. 2011
741	Invertebrates	Aporectodea caliginosa	1.6610E-01	30	1.4281E-02	Ehnes et al. 2011

742	Invertebrates	Aporectodea caliginosa	1.6664E-01	25	8.7436E-03	Ehnes et al. 2011
743	Invertebrates	Aporectodea caliginosa	1.6842E-01	15	1.2532E-02	Ehnes et al. 2011
744	Invertebrates	Aporectodea caliginosa	1.7561E-01	25	1.7050E-02	Ehnes et al. 2011
745	Invertebrates	Aporectodea caliginosa	1.7620E-01	10.6	7.7235E-03	Ehnes et al. 2011
746	Invertebrates	Aporectodea caliginosa	1.7971E-01	10	6.7034E-03	Ehnes et al. 2011
747	Invertebrates	Aporectodea caliginosa	1.9781E-01	10	4.5175E-03	Ehnes et al. 2011
748	Invertebrates	Aporectodea caliginosa	1.9819E-01	5	8.3064E-03	Ehnes et al. 2011
749	Invertebrates	Aporectodea caliginosa	2.1910E-01	20	1.5593E-02	Ehnes et al. 2011
750	Invertebrates	Aporectodea caliginosa	2.3000E-01	30	3.5266E-02	Ehnes et al. 2011
751	Invertebrates	Aporectodea caliginosa	2.3519E-01	30	2.7542E-02	Ehnes et al. 2011
752	Invertebrates	Aporectodea caliginosa	2.4559E-01	5	2.0402E-03	Ehnes et al. 2011
753	Invertebrates	Aporectodea caliginosa	2.5339E-01	20	7.1406E-03	Ehnes et al. 2011
754	Invertebrates	Aporectodea caliginosa	3.2968E-01	15	1.1658E-02	Ehnes et al. 2011
755	Invertebrates	Aporectodea caliginosa	3.4457E-01	30	3.3225E-02	Ehnes et al. 2011
756	Invertebrates	Aporectodea rosea	4.1340E-02	20	4.9547E-03	Ehnes et al. 2011
757	Invertebrates	Aporectodea rosea	4.3020E-02	10	4.2261E-03	Ehnes et al. 2011
758	Invertebrates	Aporectodea rosea	5.3100E-02	10	3.2060E-03	Ehnes et al. 2011
759	Invertebrates	Aporectodea rosea	5.4410E-02	15	4.8090E-03	Ehnes et al. 2011
760	Invertebrates	Aporectodea rosea	5.8880E-02	15	4.9547E-03	Ehnes et al. 2011
761	Invertebrates	Aporectodea rosea	5.9510E-02	15	5.2461E-03	Ehnes et al. 2011
762	Invertebrates	Aporectodea rosea	6.9100E-02	10	3.2060E-03	Ehnes et al. 2011
763	Invertebrates	Aporectodea rosea	9.0470E-02	20	6.7034E-03	Ehnes et al. 2011
764	Invertebrates	Aporectodea rosea	9.7680E-02	15	6.9948E-03	Ehnes et al. 2011
765	Invertebrates	Aporectodea rosea	1.0406E-01	20	1.5301E-02	Ehnes et al. 2011
766	Invertebrates	Aporectodea rosea	1.0908E-01	10	2.1859E-03	Ehnes et al. 2011
767	Invertebrates	Aporectodea rosea	1.3426E-01	15	1.2387E-02	Ehnes et al. 2011

768	Invertebrates	Aporectodea rosea	1.4641E-01	15	1.0347E-02	Ehnes et al. 2011
769	Invertebrates	Aporectodea rosea	1.6595E-01	15	1.0929E-02	Ehnes et al. 2011
770	Invertebrates	Archaeomysis grebnitzkii	1.0800E-03	20	4.5441E-04	Makarieva et al. 2008
771	Invertebrates	Archaeomysis grebnitzkii	1.2400E-03	20	4.9481E-04	Makarieva et al. 2008
772	Invertebrates	Archaeomysis grebnitzkii	2.6900E-03	20	8.5834E-04	Makarieva et al. 2008
773	Invertebrates	Archaeomysis grebnitzkii	3.3900E-03	20	1.0098E-03	Makarieva et al. 2008
774	Invertebrates	Archaeomysis grebnitzkii	6.4500E-03	20	1.5148E-03	Makarieva et al. 2008
775	Invertebrates	Archaeomysis grebnitzkii	7.1000E-03	20	1.6863E-03	Makarieva et al. 2008
776	Invertebrates	Archaeomysis grebnitzkii	8.0600E-03	20	1.8479E-03	Makarieva et al. 2008
777	Invertebrates	Archaeomysis grebnitzkii	9.3500E-03	20	2.0499E-03	Makarieva et al. 2008
778	Invertebrates	Archaeomysis grebnitzkii	1.0600E-02	20	2.2316E-03	Makarieva et al. 2008
779	Invertebrates	Arietellus cf. plumifer	9.5000E-03	5	3.7620E-04	Makarieva et al. 2008
780	Invertebrates	Armadillidium	7.0020E-02	29.9	1.2095E-02	Ehnes et al. 2011
781	Invertebrates	Armadillidium nasatum	3.0000E-02	20	3.9080E-03	Makarieva et al. 2008
782	Invertebrates	Armadillidium pallasii	9.0000E-04	20	5.0398E-04	Makarieva et al. 2008
783	Invertebrates	Armadillidium pallasii	2.5000E-03	20	1.1502E-03	Makarieva et al. 2008
784	Invertebrates	Armadillidium pallasii	1.5000E-02	20	2.7000E-03	Makarieva et al. 2008
785	Invertebrates	Armadillidium pallasii	2.4000E-02	20	8.8819E-03	Makarieva et al. 2008
786	Invertebrates	Armadillidium pallasii	3.0000E-02	20	4.7412E-03	Makarieva et al. 2008
787	Invertebrates	Armadillidium pallasii	5.0000E-02	20	5.9490E-03	Makarieva et al. 2008
788	Invertebrates	Armadillidium pallasii	5.3000E-02	20	1.2774E-02	Makarieva et al. 2008
789	Invertebrates	Armadillidium pallasii	5.3000E-02	20	1.4205E-02	Makarieva et al. 2008
790	Invertebrates	Armadillidium pallasii	9.0000E-02	20	1.2604E-02	Makarieva et al. 2008
791	Invertebrates	Armadillidium pallasii	1.0000E-01	20	9.3060E-03	Makarieva et al. 2008
792	Invertebrates	Armadillidium pallasii	1.3000E-01	20	1.3525E-02	Makarieva et al. 2008
793	Invertebrates	Armadillidium pallasii	1.6000E-01	20	1.5206E-02	Makarieva et al. 2008

794	Invertebrates	Armadillidium pallasii	2.7000E-01	20	2.4300E-02	Makarieva et al. 2008
795	Invertebrates	Armadillidium vulgare	6.4000E-04	20	9.1515E-04	Makarieva et al. 2008
796	Invertebrates	Armadillidium vulgare	2.0000E-03	20	5.0500E-04	Ehnes et al. 2011
797	Invertebrates	Armadillidium vulgare	4.0000E-03	20	2.0801E-03	Makarieva et al. 2008
798	Invertebrates	Armadillidium vulgare	6.0000E-03	5	3.6050E-04	Ehnes et al. 2011
799	Invertebrates	Armadillidium vulgare	6.0000E-03	10	7.9500E-04	Ehnes et al. 2011
800	Invertebrates	Armadillidium vulgare	6.0000E-03	15	1.1650E-03	Ehnes et al. 2011
801	Invertebrates	Armadillidium vulgare	6.0000E-03	20	1.4150E-03	Ehnes et al. 2011
802	Invertebrates	Armadillidium vulgare	6.0000E-03	25	1.8200E-03	Ehnes et al. 2011
803	Invertebrates	Armadillidium vulgare	2.2440E-02	10.2	3.0603E-03	Ehnes et al. 2011
804	Invertebrates	Armadillidium vulgare	2.4850E-02	10.2	1.8945E-03	Ehnes et al. 2011
805	Invertebrates	Armadillidium vulgare	3.0000E-02	20	3.9100E-03	Ehnes et al. 2011
806	Invertebrates	Armadillidium vulgare	3.6890E-02	14.2	3.3517E-03	Ehnes et al. 2011
807	Invertebrates	Armadillidium vulgare	5.3200E-02	10.2	1.0201E-03	Ehnes et al. 2011
808	Invertebrates	Armadillidium vulgare	5.4260E-02	29.2	2.2587E-02	Ehnes et al. 2011
809	Invertebrates	Armadillidium vulgare	5.4780E-02	5	1.3116E-03	Ehnes et al. 2011
810	Invertebrates	Armadillidium vulgare	5.6460E-02	14.6	4.2261E-03	Ehnes et al. 2011
811	Invertebrates	Armadillidium vulgare	5.6530E-02	14.2	5.1004E-03	Ehnes et al. 2011
812	Invertebrates	Armadillidium vulgare	5.8460E-02	14.2	4.9547E-03	Ehnes et al. 2011
813	Invertebrates	Armadillidium vulgare	5.9380E-02	5	3.6432E-03	Ehnes et al. 2011
814	Invertebrates	Armadillidium vulgare	5.9990E-02	14.2	3.7889E-03	Ehnes et al. 2011
815	Invertebrates	Armadillidium vulgare	6.0260E-02	14.6	2.6231E-03	Ehnes et al. 2011
816	Invertebrates	Armadillidium vulgare	6.2920E-02	10.2	3.9346E-03	Ehnes et al. 2011
817	Invertebrates	Armadillidium vulgare	6.3400E-02	10.2	1.4573E-03	Ehnes et al. 2011
818	Invertebrates	Armadillidium vulgare	6.3520E-02	14.2	7.4320E-03	Ehnes et al. 2011
819	Invertebrates	Armadillidium vulgare	6.5000E-02	20	9.4887E-03	Makarieva et al. 2008

820	Invertebrates	Armadillidium vulgare	6.5140E-02	10.2	6.8491E-03	Ehnes et al. 2011
821	Invertebrates	Armadillidium vulgare	6.5450E-02	14.2	4.8090E-03	Ehnes et al. 2011
822	Invertebrates	Armadillidium vulgare	6.5500E-02	20	9.7000E-03	Ehnes et al. 2011
823	Invertebrates	Armadillidium vulgare	6.8300E-02	10.2	4.2261E-03	Ehnes et al. 2011
824	Invertebrates	Armadillidium vulgare	7.0110E-02	25	1.4135E-02	Ehnes et al. 2011
825	Invertebrates	Armadillidium vulgare	7.1840E-02	29.2	2.1276E-02	Ehnes et al. 2011
826	Invertebrates	Armadillidium vulgare	7.3660E-02	29.2	1.1950E-02	Ehnes et al. 2011
827	Invertebrates	Armadillidium vulgare	7.4000E-02	10	6.2500E-03	Ehnes et al. 2011
828	Invertebrates	Armadillidium vulgare	7.4000E-02	20	1.0350E-02	Ehnes et al. 2011
829	Invertebrates	Armadillidium vulgare	7.4000E-02	30	1.7550E-02	Ehnes et al. 2011
830	Invertebrates	Armadillidium vulgare	7.4260E-02	10.2	1.1658E-03	Ehnes et al. 2011
831	Invertebrates	Armadillidium vulgare	7.5370E-02	14.2	3.3517E-03	Ehnes et al. 2011
832	Invertebrates	Armadillidium vulgare	7.7390E-02	14.6	3.4974E-03	Ehnes et al. 2011
833	Invertebrates	Armadillidium vulgare	8.0330E-02	14.2	2.9145E-03	Ehnes et al. 2011
834	Invertebrates	Armadillidium vulgare	8.1610E-02	25	3.0602E-02	Ehnes et al. 2011
835	Invertebrates	Armadillidium vulgare	8.7360E-02	5	5.8290E-04	Ehnes et al. 2011
836	Invertebrates	Armadillidium vulgare	9.0370E-02	5	3.7889E-03	Ehnes et al. 2011
837	Invertebrates	Armadillidium vulgare	9.2310E-02	5	7.5777E-03	Ehnes et al. 2011
838	Invertebrates	Armadillidium vulgare	9.2500E-02	5.4	5.9748E-03	Ehnes et al. 2011
839	Invertebrates	Armadillidium vulgare	9.2650E-02	5	7.2865E-04	Ehnes et al. 2011
840	Invertebrates	Armadillidium vulgare	9.5720E-02	5	7.2865E-04	Ehnes et al. 2011
841	Invertebrates	Armadillidium vulgare	9.5720E-02	5	7.2865E-04	Ehnes et al. 2011
842	Invertebrates	Armadillidium vulgare	9.6820E-02	14.2	8.0149E-03	Ehnes et al. 2011
843	Invertebrates	Armadillidium vulgare	9.9840E-02	5.4	3.9346E-03	Ehnes et al. 2011
844	Invertebrates	Armadillidium vulgare	1.0206E-01	5.4	3.2060E-03	Ehnes et al. 2011
845	Invertebrates	Armadillidium vulgare	1.0479E-01	10.2	1.1658E-03	Ehnes et al. 2011

846	Invertebrates	Armadillidium vulgare	1.0681E-01	25	1.3698E-02	Ehnes et al. 2011
847	Invertebrates	Armadillidium vulgare	1.2000E-01	20	2.1600E-02	Makarieva et al. 2008
848	Invertebrates	Armadillidium vulgare	1.4400E-01	20	1.4412E-02	Makarieva et al. 2008
849	Invertebrates	Artace sp.	1.3200E-01	25	5.9850E-02	Ehnes et al. 2011
850	Invertebrates	Asellus aquaticus	1.6800E-02	10	3.3566E-03	Makarieva et al. 2008
851	Invertebrates	Atolla vanhoeffeni	6.0000E-01	5	2.7000E-03	Makarieva et al. 2008
852	Invertebrates	Atolla wyvillei	1.9400E+00	5	5.9364E-03	Makarieva et al. 2008
853	Invertebrates	Atta columbica	3.6000E-03	25	2.6280E-03	Ehnes et al. 2011
854	Invertebrates	Atta columbica	4.5000E-03	25	1.5480E-03	Ehnes et al. 2011
855	Invertebrates	Atta columbica	4.5000E-03	25	2.6280E-03	Ehnes et al. 2011
856	Invertebrates	Atta columbica	4.9000E-03	25	2.3940E-03	Ehnes et al. 2011
857	Invertebrates	Atta columbica	7.4000E-03	25	4.1220E-03	Ehnes et al. 2011
858	Invertebrates	Atta columbica	8.3000E-03	25	2.3760E-03	Ehnes et al. 2011
859	Invertebrates	Atta columbica	1.0400E-02	25	3.8340E-03	Ehnes et al. 2011
860	Invertebrates	Atta columbica	1.0400E-02	25	4.2480E-03	Ehnes et al. 2011
861	Invertebrates	Atta columbica	1.1400E-02	25	4.1580E-03	Ehnes et al. 2011
862	Invertebrates	Atta columbica	1.2000E-02	25	3.4020E-03	Ehnes et al. 2011
863	Invertebrates	Atta columbica	1.2000E-02	25	3.2220E-03	Ehnes et al. 2011
864	Invertebrates	Atta columbica	1.2300E-02	25	8.8020E-03	Ehnes et al. 2011
865	Invertebrates	Atta columbica	1.2700E-02	25	4.7880E-03	Ehnes et al. 2011
866	Invertebrates	Atta columbica	1.3500E-02	25	4.7520E-03	Ehnes et al. 2011
867	Invertebrates	Atta columbica	1.5000E-02	25	4.6260E-03	Ehnes et al. 2011
868	Invertebrates	Atta columbica	1.6000E-02	25	3.0060E-03	Ehnes et al. 2011
869	Invertebrates	Atta columbica	1.6000E-02	25	4.2480E-03	Ehnes et al. 2011
870	Invertebrates	Atta columbica	1.7600E-02	25	5.3820E-03	Ehnes et al. 2011
871	Invertebrates	Atta columbica	1.9000E-02	25	9.3240E-03	Ehnes et al. 2011

872	Invertebrates	Atta columbica	1.9200E-02	25	6.5160E-03	Ehnes et al. 2011
873	Invertebrates	Atta columbica	1.9900E-02	25	5.3640E-03	Ehnes et al. 2011
874	Invertebrates	Atta columbica	2.1500E-02	25	6.0660E-03	Ehnes et al. 2011
875	Invertebrates	Atta columbica	2.2200E-02	25	8.8380E-03	Ehnes et al. 2011
876	Invertebrates	Atta columbica	2.3500E-02	25	9.4500E-03	Ehnes et al. 2011
877	Invertebrates	Atta columbica	2.4400E-02	25	5.5260E-03	Ehnes et al. 2011
878	Invertebrates	Atta columbica	2.6700E-02	25	7.4160E-03	Ehnes et al. 2011
879	Invertebrates	Atta columbica	3.3300E-02	25	6.5700E-03	Ehnes et al. 2011
880	Invertebrates	Atta laevigata	1.5000E-02	25	6.3360E-03	Ehnes et al. 2011
881	Invertebrates	Atta sexdens	1.5000E-02	25	7.7580E-03	Ehnes et al. 2011
882	Invertebrates	Automerina auletes	7.2000E-01	25	3.2648E-01	Ehnes et al. 2011
883	Invertebrates	Automeris fieldi	3.9400E-01	25	1.9589E-01	Ehnes et al. 2011
884	Invertebrates	Automeris hamata	5.6400E-01	25	3.5914E-01	Ehnes et al. 2011
885	Invertebrates	Automeris jacunda	6.5325E-01	25	2.7887E-01	Ehnes et al. 2011
886	Invertebrates	Automeris zugana	5.3975E-01	25	1.9861E-01	Ehnes et al. 2011
887	Invertebrates	Balanus balanoides	4.6000E-03	5	4.8604E-04	Makarieva et al. 2008
888	Invertebrates	Balanus balanoides	5.0000E-03	20	1.9800E-03	Makarieva et al. 2008
889	Invertebrates	Balanus balanoides	6.3000E-03	10	1.4400E-03	Makarieva et al. 2008
890	Invertebrates	Balanus balanoides	7.6000E-03	15	2.4480E-03	Makarieva et al. 2008
891	Invertebrates	Balanus balanus	4.9800E-03	20	2.0297E-03	Makarieva et al. 2008
892	Invertebrates	Balanus balanus	7.6000E-03	20	3.5848E-03	Makarieva et al. 2008
893	Invertebrates	Balanus balanus	9.2000E-02	15	7.7401E-03	Makarieva et al. 2008
894	Invertebrates	Balanus balanus	9.2000E-02	20	1.1411E-02	Makarieva et al. 2008
895	Invertebrates	Balanus balanus	1.0200E-01	10	6.2993E-03	Makarieva et al. 2008
896	Invertebrates	Balanus balanus	1.1000E-01	20	1.0260E-02	Makarieva et al. 2008
897	Invertebrates	Balanus balanus	1.1290E-01	20	1.0401E-02	Makarieva et al. 2008

898	Invertebrates	Balanus balanus	1.1700E-01	5	3.9593E-03	Makarieva et al. 2008
899	Invertebrates	Bathycalanus bradyi	2.9900E-02	4	7.2657E-04	Makarieva et al. 2008
900	Invertebrates	Bathycalanus bradyi	2.9900E-02	4	1.9913E-03	Makarieva et al. 2008
901	Invertebrates	Bathycalanus bradyi	5.3000E-02	1.5	2.0034E-03	Makarieva et al. 2008
902	Invertebrates	Bathycalanus bradyi	5.5500E-02	5	2.0380E-03	Makarieva et al. 2008
903	Invertebrates	Bathycalanus bradyi	7.5600E-02	5	2.5039E-03	Makarieva et al. 2008
904	Invertebrates	Bathycalanus princeps	3.5500E-02	5.5	3.7701E-04	Makarieva et al. 2008
905	Invertebrates	Bathycalanus princeps	3.5500E-02	5.5	2.0895E-03	Makarieva et al. 2008
906	Invertebrates	Bathycalanus richard	3.8000E-02	5	1.2517E-03	Makarieva et al. 2008
907	Invertebrates	Bathycalanus richardi	3.7700E-02	1.5	1.5879E-03	Makarieva et al. 2008
908	Invertebrates	Bathycalanus richardi	5.6000E-02	5	1.3406E-03	Makarieva et al. 2008
909	Invertebrates	Bathycalanus sp.	8.9500E-02	5.1	6.7662E-03	Makarieva et al. 2008
910	Invertebrates	Bathycalanus sp. A	5.7200E-02	5	2.1004E-03	Makarieva et al. 2008
911	Invertebrates	Bathypolypus articus	3.0000E+00	5	1.2636E-01	Makarieva et al. 2008
912	Invertebrates	Bathyporeia pelgica	8.9000E-04	20	1.0098E-03	Makarieva et al. 2008
913	Invertebrates	Bathyporeia pelgica	1.3300E-03	20	1.2114E-03	Makarieva et al. 2008
914	Invertebrates	Bathyporeia pelgica	2.2000E-03	20	1.7424E-06	Makarieva et al. 2008
915	Invertebrates	Bathyporeia pelgica	2.2200E-03	20	1.9188E-03	Makarieva et al. 2008
916	Invertebrates	Bathyporeia pilosa	8.9000E-04	20	1.2114E-03	Makarieva et al. 2008
917	Invertebrates	Bathyporeia pilosa	1.3300E-03	20	1.5156E-03	Makarieva et al. 2008
918	Invertebrates	Bathyporeia pilosa	2.1900E-03	20	1.5768E-06	Makarieva et al. 2008
919	Invertebrates	Bathyporeia pilosa	2.2200E-03	20	1.9188E-03	Makarieva et al. 2008
920	Invertebrates	Bathyporeia pilosa	4.4400E-03	20	1.8198E-03	Makarieva et al. 2008
921	Invertebrates	Belba corynopus	5.9000E-05	5	6.6500E-06	Ehnes et al. 2011
922	Invertebrates	Belba corynopus	5.9000E-05	10	1.0750E-05	Ehnes et al. 2011
923	Invertebrates	Belba corynopus	5.9000E-05	15	1.6450E-05	Ehnes et al. 2011

924	Invertebrates	Belba corynopus	5.9000E-05	20	2.6950E-05	Ehnes et al. 2011
925	Invertebrates	Bembidion	1.9620E-03	20	8.1605E-04	Ehnes et al. 2011
926	Invertebrates	Bembidion	1.9800E-03	20	9.9095E-04	Ehnes et al. 2011
927	Invertebrates	Bembidion	2.0360E-03	20	1.3698E-03	Ehnes et al. 2011
928	Invertebrates	Bembidion	2.0770E-03	10	8.5980E-04	Ehnes et al. 2011
929	Invertebrates	Bembidion	2.0800E-03	22	2.4482E-03	Ehnes et al. 2011
930	Invertebrates	Bembidion	2.0940E-03	20	9.0350E-04	Ehnes et al. 2011
931	Invertebrates	Bembidion	2.0980E-03	20	1.0784E-03	Ehnes et al. 2011
932	Invertebrates	Bembidion	2.1000E-03	15	1.2241E-03	Ehnes et al. 2011
933	Invertebrates	Bembidion	2.1020E-03	10	4.3720E-04	Ehnes et al. 2011
934	Invertebrates	Bembidion	2.1320E-03	15	4.3720E-04	Ehnes et al. 2011
935	Invertebrates	Bembidion	2.1560E-03	10	6.4120E-04	Ehnes et al. 2011
936	Invertebrates	Bembidion	2.1610E-03	10	5.2460E-04	Ehnes et al. 2011
937	Invertebrates	Bembidion	2.1860E-03	15	6.4120E-04	Ehnes et al. 2011
938	Invertebrates	Bembidion	2.1960E-03	5	2.1860E-04	Ehnes et al. 2011
939	Invertebrates	Bembidion	2.2180E-03	5	3.2060E-04	Ehnes et al. 2011
940	Invertebrates	Bembidion	2.2300E-03	5	2.1860E-04	Ehnes et al. 2011
941	Invertebrates	Bembidion	2.2400E-03	5	5.1005E-04	Ehnes et al. 2011
942	Invertebrates	Bembidion	2.2480E-03	10	4.9545E-04	Ehnes et al. 2011
943	Invertebrates	Bembidion	2.2620E-03	15	4.3720E-04	Ehnes et al. 2011
944	Invertebrates	Bembidion	2.2660E-03	25	1.7196E-03	Ehnes et al. 2011
945	Invertebrates	Bembidion	2.3060E-03	10	4.6630E-04	Ehnes et al. 2011
946	Invertebrates	Bembidion	2.3060E-03	25	1.8945E-03	Ehnes et al. 2011
947	Invertebrates	Bembidion	2.3125E-03	20	1.2023E-03	Ehnes et al. 2011
948	Invertebrates	Bembidion	2.3210E-03	10	6.8490E-04	Ehnes et al. 2011
949	Invertebrates	Bembidion	2.3480E-03	10	1.7485E-04	Ehnes et al. 2011

950	Invertebrates	Bembidion	2.3680E-03	20	1.0201E-03	Ehnes et al. 2011
951	Invertebrates	Bembidion	2.3780E-03	10	5.5375E-04	Ehnes et al. 2011
952	Invertebrates	Bembidion	2.3860E-03	15	7.2865E-04	Ehnes et al. 2011
953	Invertebrates	Bembidion	2.3920E-03	10	5.2460E-04	Ehnes et al. 2011
954	Invertebrates	Bembidion	2.4340E-03	15	6.4120E-04	Ehnes et al. 2011
955	Invertebrates	Bembidion	2.4350E-03	5	2.6230E-04	Ehnes et al. 2011
956	Invertebrates	Bembidion	2.4360E-03	5	3.4975E-04	Ehnes et al. 2011
957	Invertebrates	Bembidion	2.4440E-03	10	3.2060E-04	Ehnes et al. 2011
958	Invertebrates	Bembidion	2.5080E-03	10	4.6630E-04	Ehnes et al. 2011
959	Invertebrates	Bembidion	2.5320E-03	5	1.4575E-04	Ehnes et al. 2011
960	Invertebrates	Bembidion	2.5780E-03	10	3.4975E-04	Ehnes et al. 2011
961	Invertebrates	Bembidion	2.6360E-03	10	8.4520E-04	Ehnes et al. 2011
962	Invertebrates	Bembidion	2.6620E-03	10	6.1205E-04	Ehnes et al. 2011
963	Invertebrates	Bembidion	2.8500E-03	5	2.6230E-04	Ehnes et al. 2011
964	Invertebrates	Bembidion	2.8700E-03	5	2.6230E-04	Ehnes et al. 2011
965	Invertebrates	Bembidion	1.0100E-02	25	1.0347E-02	Ehnes et al. 2011
966	Invertebrates	Bembidion	1.1700E-02	15	6.7034E-03	Ehnes et al. 2011
967	Invertebrates	Blaberus discoidalis	4.0800E+00	25	7.0427E-01	Ehnes et al. 2011
968	Invertebrates	Blaberus giganteus	4.3300E+00	25	6.5635E-01	Ehnes et al. 2011
969	Invertebrates	Blaps gigas	1.7760E+00	25	8.1149E-01	Ehnes et al. 2011
970	Invertebrates	Blatella germanica	5.4760E-02	25	3.6594E-02	Ehnes et al. 2011
971	Invertebrates	Blatta orientalis	3.2500E-01	25	1.0006E-01	Ehnes et al. 2011
972	Invertebrates	Bombus terrestris	7.4000E-01	25	1.4792E-01	Ehnes et al. 2011
973	Invertebrates	Bombyx mori	4.9000E-01	25	6.2867E-01	Ehnes et al. 2011
974	Invertebrates	Bootettix punctatus	4.7010E-02	25	6.5646E-02	Ehnes et al. 2011
975	Invertebrates	Boreomysis californica	3.5000E-02	5.5	5.4180E-04	Makarieva et al. 2008

976	Invertebrates	Boreomysis californica	3.5000E-02	5.5	2.0412E-03	Makarieva et al. 2008
977	Invertebrates	Bothrometopus elongatus	1.7000E-03	25	2.7360E-03	Ehnes et al. 2011
978	Invertebrates	Bothrometopus parvulus	3.6000E-03	25	3.4920E-03	Ehnes et al. 2011
979	Invertebrates	Bothrometopus randi	2.4600E-02	25	9.1980E-03	Ehnes et al. 2011
980	Invertebrates	Botrynema brucei	1.3300E+00	5	4.0698E-03	Makarieva et al. 2008
981	Invertebrates	Bovallia gigantea	5.2800E-01	1	5.2842E-02	Makarieva et al. 2008
982	Invertebrates	Brachymesia gravida	3.4400E-01	25	2.8001E-01	Ehnes et al. 2011
983	Invertebrates	Brachyscelus latipes	1.2800E-03	29	4.6465E-03	Makarieva et al. 2008
984	Invertebrates	Brasilanus batus	1.9650E+00	25	1.1219E+00	Ehnes et al. 2011
985	Invertebrates	Bulbitermes sp. C	2.8000E-03	25	4.1400E-04	Ehnes et al. 2011
986	Invertebrates	Burmoniscus ocellatus	7.9400E-03	23	1.9750E-03	Ehnes et al. 2011
987	Invertebrates	Burmoniscus sp.	7.9400E-03	23	1.9300E-03	Ehnes et al. 2011
988	Invertebrates	Byblis securiger	2.3900E-01	2.4	4.1127E-02	Makarieva et al. 2008
989	Invertebrates	Byrsotria fumagata	4.9500E+00	25	1.0052E+00	Ehnes et al. 2011
990	Invertebrates	Caeosagitta macrocephala	2.1000E-02	5	3.6666E-04	Makarieva et al. 2008
991	Invertebrates	Calandra oryzae	1.7000E-03	25	3.1860E-03	Ehnes et al. 2011
992	Invertebrates	Calanoides acutus	1.1000E-03	-0.5	1.3068E-04	Makarieva et al. 2008
993	Invertebrates	Calanoides acutus	2.0000E-03	-0.2	2.1240E-04	Makarieva et al. 2008
994	Invertebrates	Calanoides acutus	3.3290E-03	0	9.6474E-05	Makarieva et al. 2008
995	Invertebrates	Calanopia elliptica	2.8000E-04	27.4	2.8022E-04	Makarieva et al. 2008
996	Invertebrates	Calanus cristatus	7.0400E-03	10.9	1.4788E-03	Makarieva et al. 2008
997	Invertebrates	Calanus cristatus	7.5500E-03	8	2.9368E-03	Makarieva et al. 2008
998	Invertebrates	Calanus cristatus	7.6000E-03	8.7	3.2750E-03	Makarieva et al. 2008
999	Invertebrates	Calanus cristatus	8.6200E-03	11.2	1.6975E-03	Makarieva et al. 2008
1000	Invertebrates	Calanus cristatus	9.0800E-03	10.7	1.7978E-03	Makarieva et al. 2008
1001	Invertebrates	Calanus cristatus	9.1800E-03	7.8	3.0569E-03	Makarieva et al. 2008

1002	Invertebrates	Calanus finmarchicus	9.4000E-04	7.5	1.7935E-04	Makarieva et al. 2008
1003	Invertebrates	Calanus finmarchicus	1.5000E-03	0.1	3.3750E-04	Makarieva et al. 2008
1004	Invertebrates	Calanus finmarchicus	1.7000E-03	8	1.5300E-04	Makarieva et al. 2008
1005	Invertebrates	Calanus glacialis	2.0000E-03	3.5	6.2280E-04	Makarieva et al. 2008
1006	Invertebrates	Calanus glacialis	2.4000E-03	-0.5	5.7888E-04	Makarieva et al. 2008
1007	Invertebrates	Calanus helgolandicus	4.1500E-04	15	2.9200E-04	Makarieva et al. 2008
1008	Invertebrates	Calanus helgolandicus	5.9500E-04	10	4.8056E-04	Makarieva et al. 2008
1009	Invertebrates	Calanus hyperboreus	8.1000E-03	0.9	1.0206E-03	Makarieva et al. 2008
1010	Invertebrates	Calanus hyperboreus	8.5000E-03	-0.3	1.0404E-03	Makarieva et al. 2008
1011	Invertebrates	Calanus hyperboreus	1.2000E-02	10	1.0368E-03	Makarieva et al. 2008
1012	Invertebrates	Calanus hyperboreus	1.2000E-02	1.3	1.4472E-03	Makarieva et al. 2008
1013	Invertebrates	Calanus hyperboreus	1.3000E-02	10	8.6580E-04	Makarieva et al. 2008
1014	Invertebrates	Calanus pacificus	5.1300E-04	17	4.4120E-04	Makarieva et al. 2008
1015	Invertebrates	Calanus propinquus	4.2000E-03	-1	6.7284E-04	Makarieva et al. 2008
1016	Invertebrates	Calanus propinquus	4.7180E-03	0	2.4968E-04	Makarieva et al. 2008
1017	Invertebrates	Calanus propinquus	5.2000E-03	-1	1.1794E-03	Makarieva et al. 2008
1018	Invertebrates	Calanus propinquus	5.6000E-03	-1	1.1794E-03	Makarieva et al. 2008
1019	Invertebrates	Calanus tonsus	1.0000E-03	-1	3.9204E-04	Makarieva et al. 2008
1020	Invertebrates	Calanus vulgaris	1.0600E-03	30	4.5050E-03	Makarieva et al. 2008
1021	Invertebrates	Calastacus quinqueseriatus	1.3800E+00	15	6.7068E-02	Makarieva et al. 2008
1022	Invertebrates	Calastacus quinqueseriatus	1.7300E+00	10	5.2938E-02	Makarieva et al. 2008
1023	Invertebrates	Calastacus quinqueseriatus	3.1300E+00	5	5.6340E-02	Makarieva et al. 2008
1024	Invertebrates	Calastacus quinqueseriatus	3.2100E+00	2.5	2.0223E-02	Makarieva et al. 2008
1025	Invertebrates	Calathus fuscipes	1.5990E-02	10	2.4774E-03	Ehnes et al. 2011
1026	Invertebrates	Calathus fuscipes	3.0070E-02	15	2.2150E-02	Ehnes et al. 2011
1027	Invertebrates	Calathus fuscipes	4.7770E-02	30	2.9437E-02	Ehnes et al. 2011

1028	Invertebrates	Calathus fuscipes	4.7980E-02	30	4.0220E-02	Ehnes et al. 2011
1029	Invertebrates	Calathus fuscipes	4.8740E-02	20	1.2387E-02	Ehnes et al. 2011
1030	Invertebrates	Calathus fuscipes	4.9000E-02	25	2.0984E-02	Ehnes et al. 2011
1031	Invertebrates	Calathus fuscipes	5.1470E-02	30	4.0366E-02	Ehnes et al. 2011
1032	Invertebrates	Calathus fuscipes	5.1520E-02	10	8.0149E-03	Ehnes et al. 2011
1033	Invertebrates	Calathus fuscipes	5.2140E-02	10	1.1950E-02	Ehnes et al. 2011
1034	Invertebrates	Calathus fuscipes	5.2140E-02	25	1.3407E-02	Ehnes et al. 2011
1035	Invertebrates	Calathus fuscipes	5.2440E-02	20	2.2879E-02	Ehnes et al. 2011
1036	Invertebrates	Calathus fuscipes	5.2750E-02	20	2.0402E-02	Ehnes et al. 2011
1037	Invertebrates	Calathus fuscipes	5.2990E-02	10	1.0201E-03	Ehnes et al. 2011
1038	Invertebrates	Calathus fuscipes	5.4040E-02	25	2.5793E-02	Ehnes et al. 2011
1039	Invertebrates	Calathus fuscipes	5.4440E-02	20	1.4864E-02	Ehnes et al. 2011
1040	Invertebrates	Calathus fuscipes	5.4870E-02	5	4.8090E-03	Ehnes et al. 2011
1041	Invertebrates	Calathus fuscipes	5.4920E-02	15	1.0784E-02	Ehnes et al. 2011
1042	Invertebrates	Calathus fuscipes	5.5170E-02	25	2.7979E-02	Ehnes et al. 2011
1043	Invertebrates	Calathus fuscipes	5.5960E-02	10	4.2261E-03	Ehnes et al. 2011
1044	Invertebrates	Calathus fuscipes	5.6000E-02	10	8.1607E-03	Ehnes et al. 2011
1045	Invertebrates	Calathus fuscipes	5.6270E-02	25	1.5884E-02	Ehnes et al. 2011
1046	Invertebrates	Calathus fuscipes	5.6290E-02	20	1.9964E-02	Ehnes et al. 2011
1047	Invertebrates	Calathus fuscipes	5.6300E-02	25	2.4482E-02	Ehnes et al. 2011
1048	Invertebrates	Calathus fuscipes	5.6480E-02	15	9.0350E-03	Ehnes et al. 2011
1049	Invertebrates	Calathus fuscipes	5.7630E-02	30	4.1823E-02	Ehnes et al. 2011
1050	Invertebrates	Calathus fuscipes	5.8850E-02	10	2.3608E-02	Ehnes et al. 2011
1051	Invertebrates	Calathus fuscipes	5.9860E-02	25	1.5301E-02	Ehnes et al. 2011
1052	Invertebrates	Calathus fuscipes	6.0320E-02	5	1.1658E-03	Ehnes et al. 2011
1053	Invertebrates	Calathus fuscipes	6.1140E-02	5	1.1658E-03	Ehnes et al. 2011

1054	Invertebrates	Calathus fuscipes	6.2410E-02	5	1.7924E-02	Ehnes et al. 2011
1055	Invertebrates	Calathus fuscipes	6.2740E-02	25	1.6758E-02	Ehnes et al. 2011
1056	Invertebrates	Calathus fuscipes	6.2770E-02	10	4.6632E-03	Ehnes et al. 2011
1057	Invertebrates	Calathus fuscipes	6.3210E-02	5	8.0149E-03	Ehnes et al. 2011
1058	Invertebrates	Calathus fuscipes	6.3590E-02	5	4.3718E-03	Ehnes et al. 2011
1059	Invertebrates	Calathus fuscipes	6.4100E-02	25	2.2150E-02	Ehnes et al. 2011
1060	Invertebrates	Calathus fuscipes	6.4620E-02	10	7.7235E-03	Ehnes et al. 2011
1061	Invertebrates	Calathus fuscipes	6.5720E-02	25	1.1658E-02	Ehnes et al. 2011
1062	Invertebrates	Calathus fuscipes	6.5720E-02	20	1.0201E-02	Ehnes et al. 2011
1063	Invertebrates	Calathus fuscipes	6.5900E-02	20	1.4427E-02	Ehnes et al. 2011
1064	Invertebrates	Calathus fuscipes	6.5940E-02	15	1.4718E-02	Ehnes et al. 2011
1065	Invertebrates	Calathus fuscipes	6.6100E-02	5	5.5376E-03	Ehnes et al. 2011
1066	Invertebrates	Calathus fuscipes	6.7060E-02	15	1.5884E-02	Ehnes et al. 2011
1067	Invertebrates	Calathus fuscipes	6.7350E-02	10	9.0350E-03	Ehnes et al. 2011
1068	Invertebrates	Calathus fuscipes	6.7480E-02	20	1.8070E-02	Ehnes et al. 2011
1069	Invertebrates	Calathus fuscipes	6.8580E-02	5	5.6833E-03	Ehnes et al. 2011
1070	Invertebrates	Calathus fuscipes	6.8980E-02	5	3.2060E-03	Ehnes et al. 2011
1071	Invertebrates	Calathus fuscipes	6.9270E-02	15	1.7196E-02	Ehnes et al. 2011
1072	Invertebrates	Calathus fuscipes	6.9710E-02	10	1.0784E-02	Ehnes et al. 2011
1073	Invertebrates	Calathus fuscipes	6.9930E-02	25	2.2442E-02	Ehnes et al. 2011
1074	Invertebrates	Calathus fuscipes	7.0240E-02	10	9.4722E-03	Ehnes et al. 2011
1075	Invertebrates	Calathus fuscipes	7.0430E-02	5	5.3919E-03	Ehnes et al. 2011
1076	Invertebrates	Calathus fuscipes	7.1230E-02	15	1.7924E-02	Ehnes et al. 2011
1077	Invertebrates	Calathus fuscipes	7.1500E-02	25	2.3753E-02	Ehnes et al. 2011
1078	Invertebrates	Calathus fuscipes	7.1520E-02	5	7.4320E-03	Ehnes et al. 2011
1079	Invertebrates	Calathus fuscipes	7.2230E-02	25	1.3115E-02	Ehnes et al. 2011

1080	Invertebrates	Calathus fuscipes	7.2430E-02	15	9.0350E-03	Ehnes et al. 2011
1081	Invertebrates	Calathus fuscipes	7.2510E-02	25	2.4045E-02	Ehnes et al. 2011
1082	Invertebrates	Calathus fuscipes	7.3120E-02	20	1.4573E-02	Ehnes et al. 2011
1083	Invertebrates	Calathus fuscipes	7.3350E-02	10	1.0638E-02	Ehnes et al. 2011
1084	Invertebrates	Calathus fuscipes	7.4340E-02	15	2.2005E-02	Ehnes et al. 2011
1085	Invertebrates	Calathus fuscipes	7.4520E-02	25	1.3990E-02	Ehnes et al. 2011
1086	Invertebrates	Calathus fuscipes	7.4550E-02	10	6.7034E-03	Ehnes et al. 2011
1087	Invertebrates	Calathus fuscipes	7.4730E-02	20	2.1276E-02	Ehnes et al. 2011
1088	Invertebrates	Calathus fuscipes	7.5140E-02	5	1.3844E-02	Ehnes et al. 2011
1089	Invertebrates	Calathus fuscipes	7.5490E-02	30	3.5266E-02	Ehnes et al. 2011
1090	Invertebrates	Calathus fuscipes	7.6320E-02	30	3.0165E-02	Ehnes et al. 2011
1091	Invertebrates	Calathus fuscipes	7.6830E-02	15	1.2576E-01	Ehnes et al. 2011
1092	Invertebrates	Calathus fuscipes	7.7530E-02	15	1.5010E-02	Ehnes et al. 2011
1093	Invertebrates	Calathus fuscipes	7.7630E-02	5	5.8290E-04	Ehnes et al. 2011
1094	Invertebrates	Calathus fuscipes	7.9330E-02	10	1.6613E-02	Ehnes et al. 2011
1095	Invertebrates	Calathus fuscipes	7.9870E-02	30	3.2205E-02	Ehnes et al. 2011
1096	Invertebrates	Calathus fuscipes	8.0440E-02	10	8.8893E-03	Ehnes et al. 2011
1097	Invertebrates	Calathus fuscipes	8.0620E-02	10	8.5978E-03	Ehnes et al. 2011
1098	Invertebrates	Calathus fuscipes	8.5120E-02	15	2.0547E-02	Ehnes et al. 2011
1099	Invertebrates	Calathus fuscipes	8.6710E-02	10	8.3064E-03	Ehnes et al. 2011
1100	Invertebrates	Calathus fuscipes	8.9280E-02	10	1.1367E-02	Ehnes et al. 2011
1101	Invertebrates	Calathus fuscipes	9.1640E-02	15	1.7633E-02	Ehnes et al. 2011
1102	Invertebrates	Calathus fuscipes	9.4100E-02	15	2.7251E-02	Ehnes et al. 2011
1103	Invertebrates	Calathus fuscipes	9.6230E-02	15	2.7105E-02	Ehnes et al. 2011
1104	Invertebrates	Calathus fuscipes	1.0146E-01	15	1.7196E-02	Ehnes et al. 2011
1105	Invertebrates	Calathus fuscipes	1.0480E-01	10	1.6904E-02	Ehnes et al. 2011

1106	Invertebrates	Calathus melanocephalus	9.0500E-03	15	3.4974E-03	Ehnes et al. 2011
1107	Invertebrates	Calathus melanocephalus	9.4000E-03	20	3.2060E-03	Ehnes et al. 2011
1108	Invertebrates	Calathus melanocephalus	9.4000E-03	30	9.6179E-03	Ehnes et al. 2011
1109	Invertebrates	Calathus melanocephalus	1.0460E-02	25	1.5155E-02	Ehnes et al. 2011
1110	Invertebrates	Calathus melanocephalus	1.0470E-02	25	9.1807E-03	Ehnes et al. 2011
1111	Invertebrates	Calathus melanocephalus	1.1750E-02	20	7.5777E-03	Ehnes et al. 2011
1112	Invertebrates	Calathus melanocephalus	1.1760E-02	5	2.7688E-03	Ehnes et al. 2011
1113	Invertebrates	Calathus melanocephalus	1.1810E-02	15	5.8290E-03	Ehnes et al. 2011
1114	Invertebrates	Calathus melanocephalus	1.2480E-02	15	3.7889E-03	Ehnes et al. 2011
1115	Invertebrates	Calathus melanocephalus	1.2710E-02	10	2.7688E-03	Ehnes et al. 2011
1116	Invertebrates	Calathus melanocephalus	1.2810E-02	25	6.7034E-03	Ehnes et al. 2011
1117	Invertebrates	Calathus melanocephalus	1.3200E-02	15	4.8090E-03	Ehnes et al. 2011
1118	Invertebrates	Calathus melanocephalus	1.3240E-02	25	1.0929E-02	Ehnes et al. 2011
1119	Invertebrates	Calathus melanocephalus	1.3300E-02	25	8.1607E-03	Ehnes et al. 2011
1120	Invertebrates	Calathus melanocephalus	1.3500E-02	15	4.8090E-03	Ehnes et al. 2011
1121	Invertebrates	Calathus melanocephalus	1.3610E-02	10	2.3316E-03	Ehnes et al. 2011
1122	Invertebrates	Calathus melanocephalus	1.4390E-02	25	1.1221E-02	Ehnes et al. 2011
1123	Invertebrates	Calathus melanocephalus	1.4400E-02	5	3.2060E-03	Ehnes et al. 2011
1124	Invertebrates	Calathus melanocephalus	1.4470E-02	15	7.1406E-03	Ehnes et al. 2011
1125	Invertebrates	Calathus melanocephalus	1.4680E-02	10	1.0201E-03	Ehnes et al. 2011
1126	Invertebrates	Calathus melanocephalus	1.4840E-02	10	1.7487E-03	Ehnes et al. 2011
1127	Invertebrates	Calathus melanocephalus	1.5030E-02	15	4.8090E-03	Ehnes et al. 2011
1128	Invertebrates	Calathus melanocephalus	1.5160E-02	15	2.4774E-03	Ehnes et al. 2011
1129	Invertebrates	Calathus melanocephalus	1.5460E-02	5	1.4573E-03	Ehnes et al. 2011
1130	Invertebrates	Calathus melanocephalus	1.5460E-02	5	5.8290E-04	Ehnes et al. 2011
1131	Invertebrates	Calathus melanocephalus	1.5710E-02	30	1.1075E-02	Ehnes et al. 2011

1132	Invertebrates	Calathus melanocephalus	1.6100E-02	10	1.3116E-03	Ehnes et al. 2011
1133	Invertebrates	Calathus melanocephalus	1.6130E-02	25	8.3064E-03	Ehnes et al. 2011
1134	Invertebrates	Calathus melanocephalus	1.6270E-02	25	8.7436E-03	Ehnes et al. 2011
1135	Invertebrates	Calathus melanocephalus	1.6370E-02	15	5.3919E-03	Ehnes et al. 2011
1136	Invertebrates	Calathus melanocephalus	1.6580E-02	10	2.4774E-03	Ehnes et al. 2011
1137	Invertebrates	Calathus melanocephalus	1.7090E-02	10	3.6432E-03	Ehnes et al. 2011
1138	Invertebrates	Calathus melanocephalus	1.7440E-02	25	8.7436E-03	Ehnes et al. 2011
1139	Invertebrates	Calathus melanocephalus	1.7780E-02	20	5.2461E-03	Ehnes et al. 2011
1140	Invertebrates	Calathus melanocephalus	1.7780E-02	30	1.4718E-02	Ehnes et al. 2011
1141	Invertebrates	Calathus melanocephalus	1.8170E-02	30	1.1658E-02	Ehnes et al. 2011
1142	Invertebrates	Calathus melanocephalus	1.8480E-02	10	5.2461E-03	Ehnes et al. 2011
1143	Invertebrates	Calathus melanocephalus	1.8560E-02	20	8.4521E-03	Ehnes et al. 2011
1144	Invertebrates	Calathus melanocephalus	1.8660E-02	20	3.3517E-03	Ehnes et al. 2011
1145	Invertebrates	Calathus melanocephalus	1.8660E-02	30	1.2532E-02	Ehnes et al. 2011
1146	Invertebrates	Calathus melanocephalus	1.9410E-02	20	6.5577E-03	Ehnes et al. 2011
1147	Invertebrates	Calathus melanocephalus	1.9790E-02	20	3.9346E-03	Ehnes et al. 2011
1148	Invertebrates	Calathus melanocephalus	1.9790E-02	30	9.4722E-03	Ehnes et al. 2011
1149	Invertebrates	Calathus melanocephalus	2.0960E-02	10	5.6833E-03	Ehnes et al. 2011
1150	Invertebrates	Calathus melanocephalus	2.0970E-02	20	5.2461E-03	Ehnes et al. 2011
1151	Invertebrates	Calathus melanocephalus	2.1190E-02	10	6.2662E-03	Ehnes et al. 2011
1152	Invertebrates	Calathus melanocephalus	2.1240E-02	10	4.8090E-03	Ehnes et al. 2011
1153	Invertebrates	Calathus melanocephalus	2.1820E-02	20	4.6632E-03	Ehnes et al. 2011
1154	Invertebrates	Calathus melanocephalus	2.1820E-02	30	1.2241E-02	Ehnes et al. 2011
1155	Invertebrates	Calathus melanocephalus	2.2120E-02	10	7.2865E-04	Ehnes et al. 2011
1156	Invertebrates	Calathus melanocephalus	2.3330E-02	5	2.6231E-03	Ehnes et al. 2011
1157	Invertebrates	Calathus melanocephalus	2.4120E-02	5	3.6432E-03	Ehnes et al. 2011

1158	Invertebrates	Calathus melanocephalus	6.9100E-02	10	5.3919E-03	Ehnes et al. 2011
1159	Invertebrates	Calathus piceus	3.0560E-02	15	7.4320E-03	Ehnes et al. 2011
1160	Invertebrates	Calathus piceus	3.0960E-02	15	1.0055E-02	Ehnes et al. 2011
1161	Invertebrates	Calathus piceus	3.5250E-02	15	5.6833E-03	Ehnes et al. 2011
1162	Invertebrates	Calathus piceus	3.8840E-02	15	1.4281E-02	Ehnes et al. 2011
1163	Invertebrates	Calathus piceus	4.0760E-02	15	1.1804E-02	Ehnes et al. 2011
1164	Invertebrates	Calathus piceus	4.1190E-02	15	1.2824E-02	Ehnes et al. 2011
1165	Invertebrates	Callinectes sapidus	1.0720E+02	20	1.0603E+01	Makarieva et al. 2008
1166	Invertebrates	Callinectes sapidus	1.3410E+02	20	1.1714E+01	Makarieva et al. 2008
1167	Invertebrates	Callinectes sapidus	1.4240E+02	20	1.0401E+01	Makarieva et al. 2008
1168	Invertebrates	Callinectes sapidus	1.5890E+02	20	1.5551E+01	Makarieva et al. 2008
1169	Invertebrates	Calosoma affine	6.2000E-01	25	1.4170E-01	Ehnes et al. 2011
1170	Invertebrates	Calosoma sp.	1.5123E-01	25	1.5095E-01	Ehnes et al. 2011
1171	Invertebrates	Campalita chlorostictum	5.2100E-01	25	1.4364E-01	Ehnes et al. 2011
1172	Invertebrates	Camponotus sp.	1.5410E-02	25	1.4076E-02	Ehnes et al. 2011
1173	Invertebrates	Camponotus detritus	4.2900E-02	25	1.4040E-02	Ehnes et al. 2011
1174	Invertebrates	Camponotus fulvopilosus	1.0850E-02	25	3.2220E-03	Ehnes et al. 2011
1175	Invertebrates	Camponotus fulvopilosus	1.3860E-02	25	4.7700E-03	Ehnes et al. 2011
1176	Invertebrates	Camponotus fulvopilosus	1.4480E-02	25	4.6260E-03	Ehnes et al. 2011
1177	Invertebrates	Camponotus fulvopilosus	1.4710E-02	25	4.4820E-03	Ehnes et al. 2011
1178	Invertebrates	Camponotus fulvopilosus	2.2150E-02	25	5.5260E-03	Ehnes et al. 2011
1179	Invertebrates	Camponotus fulvopilosus	2.2690E-02	25	7.9020E-03	Ehnes et al. 2011
1180	Invertebrates	Camponotus fulvopilosus	2.3380E-02	25	8.0100E-03	Ehnes et al. 2011
1181	Invertebrates	Camponotus fulvopilosus	2.4310E-02	25	4.7520E-03	Ehnes et al. 2011
1182	Invertebrates	Camponotus fulvopilosus	2.4380E-02	25	7.8300E-03	Ehnes et al. 2011
1183	Invertebrates	Camponotus fulvopilosus	2.7460E-02	25	1.1790E-02	Ehnes et al. 2011

1184	Invertebrates	Camponotus fulvopilosus	2.7620E-02	25	7.1460E-03	Ehnes et al. 2011
1185	Invertebrates	Camponotus fulvopilosus	3.2020E-02	25	5.0400E-03	Ehnes et al. 2011
1186	Invertebrates	Camponotus fulvopilosus	3.3510E-02	25	8.8020E-03	Ehnes et al. 2011
1187	Invertebrates	Camponotus fulvopilosus	3.9680E-02	25	8.9460E-03	Ehnes et al. 2011
1188	Invertebrates	Camponotus fulvopilosus	4.3000E-02	25	1.1412E-02	Ehnes et al. 2011
1189	Invertebrates	Camponotus fulvopilosus	4.7920E-02	25	1.1376E-02	Ehnes et al. 2011
1190	Invertebrates	Camponotus fulvopilosus	4.9170E-02	25	6.8220E-03	Ehnes et al. 2011
1191	Invertebrates	Camponotus fulvopilosus	5.3900E-02	25	1.4274E-02	Ehnes et al. 2011
1192	Invertebrates	Camponotus fulvopilosus	5.8310E-02	25	7.7580E-03	Ehnes et al. 2011
1193	Invertebrates	Camponotus fulvopilosus	5.9490E-02	25	8.8020E-03	Ehnes et al. 2011
1194	Invertebrates	Camponotus fulvopilosus	5.9660E-02	25	1.4580E-02	Ehnes et al. 2011
1195	Invertebrates	Camponotus fulvopilosus	8.4740E-02	25	1.5282E-02	Ehnes et al. 2011
1196	Invertebrates	Camponotus fulvopilosus	8.8950E-02	25	1.4418E-02	Ehnes et al. 2011
1197	Invertebrates	Camponotus fulvopilosus	1.0606E-01	25	1.5498E-02	Ehnes et al. 2011
1198	Invertebrates	Camponotus fulvopilosus	1.5530E-01	25	1.3086E-02	Ehnes et al. 2011
1199	Invertebrates	Camponotus laevigatus	1.9150E-02	25	2.1204E-02	Ehnes et al. 2011
1200	Invertebrates	Camponotus maculatus	7.7000E-03	20	3.1320E-03	Ehnes et al. 2011
1201	Invertebrates	Camponotus maculatus	1.0400E-02	20	2.9340E-03	Ehnes et al. 2011
1202	Invertebrates	Camponotus maculatus	1.0800E-02	20	4.3380E-03	Ehnes et al. 2011
1203	Invertebrates	Camponotus maculatus	1.0900E-02	20	3.8160E-03	Ehnes et al. 2011
1204	Invertebrates	Camponotus maculatus	1.1000E-02	20	2.9340E-03	Ehnes et al. 2011
1205	Invertebrates	Camponotus maculatus	1.1000E-02	20	3.7980E-03	Ehnes et al. 2011
1206	Invertebrates	Camponotus maculatus	1.1100E-02	20	2.7720E-03	Ehnes et al. 2011
1207	Invertebrates	Camponotus maculatus	1.1200E-02	20	3.6720E-03	Ehnes et al. 2011
1208	Invertebrates	Camponotus maculatus	1.3400E-02	20	3.6000E-03	Ehnes et al. 2011
1209	Invertebrates	Camponotus maculatus	1.4200E-02	20	2.5740E-03	Ehnes et al. 2011

1210	Invertebrates	Camponotus maculatus	1.5100E-02	20	4.3020E-03	Ehnes et al. 2011
1211	Invertebrates	Camponotus maculatus	1.5200E-02	20	3.7620E-03	Ehnes et al. 2011
1212	Invertebrates	Camponotus maculatus	1.5700E-02	20	5.0400E-03	Ehnes et al. 2011
1213	Invertebrates	Camponotus maculatus	1.6200E-02	20	3.2400E-03	Ehnes et al. 2011
1214	Invertebrates	Camponotus maculatus	1.6600E-02	20	4.5720E-03	Ehnes et al. 2011
1215	Invertebrates	Camponotus maculatus	1.8300E-02	20	4.0140E-03	Ehnes et al. 2011
1216	Invertebrates	Camponotus maculatus	1.8400E-02	20	4.5360E-03	Ehnes et al. 2011
1217	Invertebrates	Camponotus maculatus	1.8700E-02	20	4.4280E-03	Ehnes et al. 2011
1218	Invertebrates	Camponotus maculatus	1.8900E-02	20	4.3020E-03	Ehnes et al. 2011
1219	Invertebrates	Camponotus maculatus	1.9200E-02	20	5.5440E-03	Ehnes et al. 2011
1220	Invertebrates	Camponotus maculatus	2.1700E-02	20	3.8700E-03	Ehnes et al. 2011
1221	Invertebrates	Camponotus maculatus	2.2200E-02	20	4.3740E-03	Ehnes et al. 2011
1222	Invertebrates	Camponotus maculatus	2.3500E-02	20	5.0220E-03	Ehnes et al. 2011
1223	Invertebrates	Camponotus maculatus	2.3600E-02	20	4.1940E-03	Ehnes et al. 2011
1224	Invertebrates	Camponotus maculatus	2.4300E-02	20	5.1120E-03	Ehnes et al. 2011
1225	Invertebrates	Camponotus maculatus	2.4800E-02	20	4.4280E-03	Ehnes et al. 2011
1226	Invertebrates	Camponotus maculatus	2.5400E-02	20	5.9760E-03	Ehnes et al. 2011
1227	Invertebrates	Camponotus maculatus	2.5600E-02	20	3.9600E-03	Ehnes et al. 2011
1228	Invertebrates	Camponotus maculatus	2.5700E-02	20	4.1580E-03	Ehnes et al. 2011
1229	Invertebrates	Camponotus maculatus	2.7000E-02	20	3.6540E-03	Ehnes et al. 2011
1230	Invertebrates	Camponotus maculatus	2.9500E-02	20	2.6460E-03	Ehnes et al. 2011
1231	Invertebrates	Camponotus maculatus	2.9800E-02	20	1.2204E-02	Ehnes et al. 2011
1232	Invertebrates	Camponotus maculatus	3.0700E-02	20	4.1940E-03	Ehnes et al. 2011
1233	Invertebrates	Camponotus maculatus	3.3200E-02	20	3.8340E-03	Ehnes et al. 2011
1234	Invertebrates	Camponotus maculatus	3.3200E-02	20	4.0140E-03	Ehnes et al. 2011
1235	Invertebrates	Camponotus maculatus	3.3900E-02	20	8.7840E-03	Ehnes et al. 2011

1236	Invertebrates	Camponotus maculatus	3.7200E-02	20	4.2840E-03	Ehnes et al. 2011
1237	Invertebrates	Camponotus maculatus	3.7200E-02	20	3.6720E-03	Ehnes et al. 2011
1238	Invertebrates	Camponotus maculatus	4.2490E-02	25	9.1980E-03	Ehnes et al. 2011
1239	Invertebrates	Camponotus maculatus	7.1700E-02	20	1.0926E-02	Ehnes et al. 2011
1240	Invertebrates	Camponotus maculatus	9.0200E-02	20	1.2096E-02	Ehnes et al. 2011
1241	Invertebrates	Camponotus maculatus	1.0000E-01	20	2.5632E-02	Ehnes et al. 2011
1242	Invertebrates	Camponotus maculatus	1.0610E-01	20	9.7200E-03	Ehnes et al. 2011
1243	Invertebrates	Camponotus maculatus	1.0660E-01	20	1.0098E-02	Ehnes et al. 2011
1244	Invertebrates	Camponotus maculatus	1.0750E-01	20	2.6604E-02	Ehnes et al. 2011
1245	Invertebrates	Camponotus maculatus	1.1020E-01	20	1.2636E-02	Ehnes et al. 2011
1246	Invertebrates	Camponotus maculatus	1.1060E-01	20	1.0422E-02	Ehnes et al. 2011
1247	Invertebrates	Camponotus maculatus	1.1170E-01	20	9.8460E-03	Ehnes et al. 2011
1248	Invertebrates	Camponotus maculatus	1.1190E-01	20	8.9280E-03	Ehnes et al. 2011
1249	Invertebrates	Camponotus maculatus	1.2060E-01	20	1.7100E-02	Ehnes et al. 2011
1250	Invertebrates	Camponotus maculatus	1.2920E-01	20	1.4688E-02	Ehnes et al. 2011
1251	Invertebrates	Camponotus sericeiventris	4.0200E-02	25	1.3176E-02	Ehnes et al. 2011
1252	Invertebrates	Camponotus vafer	4.5100E-03	25	1.0584E-02	Ehnes et al. 2011
1253	Invertebrates	Camponotus vicinus	1.0700E-01	25	2.5830E-02	Ehnes et al. 2011
1254	Invertebrates	Cancer magister	9.4800E+02	20	6.7147E+01	Makarieva et al. 2008
1255	Invertebrates	Cancer pagurus	4.6000E+02	10	2.7324E+00	Makarieva et al. 2008
1256	Invertebrates	Candacia aethiopica	4.6000E-04	28.75	1.7618E-03	Makarieva et al. 2008
1257	Invertebrates	Candacia columbiae	3.1200E-03	6.7	3.0551E-04	Makarieva et al. 2008
1258	Invertebrates	Candacia columbiae	3.6700E-03	6.3	1.2816E-04	Makarieva et al. 2008
1259	Invertebrates	Candacia sp.	1.5500E-04	15	2.8500E-04	Makarieva et al. 2008
1260	Invertebrates	Canonopsis sericeus	5.8900E-02	25	3.1518E-02	Ehnes et al. 2011
1261	Invertebrates	Carabodes coriaceus	7.5000E-05	5	2.1500E-06	Ehnes et al. 2011

1262	Invertebrates	Carabodes coriaceus	7.5000E-05	10	4.6500E-06	Ehnes et al. 2011
1263	Invertebrates	Carabodes coriaceus	7.5000E-05	15	7.6000E-06	Ehnes et al. 2011
1264	Invertebrates	Carabodes marginatus	4.2600E-05	5	1.4500E-06	Ehnes et al. 2011
1265	Invertebrates	Carabodes marginatus	4.2600E-05	10	1.9500E-06	Ehnes et al. 2011
1266	Invertebrates	Carabodes marginatus	4.2600E-05	15	3.7500E-06	Ehnes et al. 2011
1267	Invertebrates	Carabus auratus	3.5670E-01	11.5	2.7251E-02	Ehnes et al. 2011
1268	Invertebrates	Carabus auratus	3.5970E-01	15	4.7944E-02	Ehnes et al. 2011
1269	Invertebrates	Carabus auratus	4.2020E-01	11.5	3.8034E-02	Ehnes et al. 2011
1270	Invertebrates	Carabus auratus	4.4690E-01	22	1.7779E-01	Ehnes et al. 2011
1271	Invertebrates	Carabus auratus	4.5500E-01	8	3.0457E-02	Ehnes et al. 2011
1272	Invertebrates	Carabus auratus	4.6050E-01	15	1.0521E-01	Ehnes et al. 2011
1273	Invertebrates	Carabus auratus	4.6570E-01	18.5	1.2197E-01	Ehnes et al. 2011
1274	Invertebrates	Carabus auratus	4.6620E-01	18.5	1.2868E-01	Ehnes et al. 2011
1275	Invertebrates	Carabus auratus	4.6630E-01	11.5	4.6632E-02	Ehnes et al. 2011
1276	Invertebrates	Carabus auratus	4.6830E-01	15	6.7471E-02	Ehnes et al. 2011
1277	Invertebrates	Carabus auratus	4.7150E-01	22	1.4063E-01	Ehnes et al. 2011
1278	Invertebrates	Carabus auratus	4.9770E-01	8	3.4246E-02	Ehnes et al. 2011
1279	Invertebrates	Carabus auratus	5.0620E-01	18.5	2.2019E-01	Ehnes et al. 2011
1280	Invertebrates	Carabus auratus	5.1020E-01	15	8.0732E-02	Ehnes et al. 2011
1281	Invertebrates	Carabus auratus	5.1040E-01	22	1.2678E-01	Ehnes et al. 2011
1282	Invertebrates	Carabus auratus	5.1780E-01	15	7.0094E-02	Ehnes et al. 2011
1283	Invertebrates	Carabus auratus	5.3010E-01	15	8.6998E-02	Ehnes et al. 2011
1284	Invertebrates	Carabus auratus	5.3100E-01	18.5	1.9323E-01	Ehnes et al. 2011
1285	Invertebrates	Carabus auratus	5.5410E-01	22	1.4033E-01	Ehnes et al. 2011
1286	Invertebrates	Carabus auratus	5.6670E-01	15	8.4229E-02	Ehnes et al. 2011
1287	Invertebrates	Carabus auratus	5.7540E-01	8	2.3462E-02	Ehnes et al. 2011

1288	Invertebrates	Carabus auratus	5.9290E-01	18.5	1.9221E-01	Ehnes et al. 2011
1289	Invertebrates	Carabus auratus	6.0910E-01	8	1.6904E-02	Ehnes et al. 2011
1290	Invertebrates	Carabus auratus	6.1040E-01	11.5	1.0390E-01	Ehnes et al. 2011
1291	Invertebrates	Carabus auratus	6.2920E-01	18.5	1.2882E-01	Ehnes et al. 2011
1292	Invertebrates	Carabus auratus	6.3560E-01	22	1.9731E-01	Ehnes et al. 2011
1293	Invertebrates	Carabus auratus	6.4550E-01	22	1.8172E-01	Ehnes et al. 2011
1294	Invertebrates	Carabus auratus	6.5840E-01	11.5	2.4628E-02	Ehnes et al. 2011
1295	Invertebrates	Carabus auratus	6.5840E-01	18.5	6.0476E-02	Ehnes et al. 2011
1296	Invertebrates	Carabus auratus	6.8830E-01	8	3.5703E-02	Ehnes et al. 2011
1297	Invertebrates	Carabus auratus	6.8860E-01	15	1.0915E-01	Ehnes et al. 2011
1298	Invertebrates	Carabus auratus	7.1310E-01	22	1.5024E-01	Ehnes et al. 2011
1299	Invertebrates	Carabus auratus	7.1720E-01	11.5	7.3008E-02	Ehnes et al. 2011
1300	Invertebrates	Carabus auratus	7.3520E-01	8	3.6723E-02	Ehnes et al. 2011
1301	Invertebrates	Carabus auratus	7.4200E-01	18.5	1.3115E-01	Ehnes et al. 2011
1302	Invertebrates	Carabus auratus	7.5100E-01	22	1.6977E-01	Ehnes et al. 2011
1303	Invertebrates	Carabus auratus	7.7080E-01	22	8.3501E-02	Ehnes et al. 2011
1304	Invertebrates	Carabus auratus	8.4330E-01	11.5	2.1276E-02	Ehnes et al. 2011
1305	Invertebrates	Carabus auratus	8.4330E-01	18.5	7.7380E-02	Ehnes et al. 2011
1306	Invertebrates	Carabus auratus	8.8350E-01	11.5	1.2766E-01	Ehnes et al. 2011
1307	Invertebrates	Carabus auratus	9.5320E-01	22	1.1352E-01	Ehnes et al. 2011
1308	Invertebrates	Carabus violaceus	9.7285E-01	15	1.1367E-01	Ehnes et al. 2011
1309	Invertebrates	Carcinus maenas	1.2700E+01	20	9.9898E-01	Makarieva et al. 2008
1310	Invertebrates	Carcinus maenas	1.3300E+01	20	8.9057E-01	Makarieva et al. 2008
1311	Invertebrates	Carcinus maenas	5.2800E+01	20	2.4986E+00	Makarieva et al. 2008
1312	Invertebrates	Carcinus maenas	5.2800E+01	20	2.5851E+00	Makarieva et al. 2008
1313	Invertebrates	Cardiosis fairmarei	3.2000E-02	25	5.3280E-03	Ehnes et al. 2011

1314	Invertebrates	Carinum sp.	4.2400E+00	25	4.3720E-01	Ehnes et al. 2011
1315	Invertebrates	Cataglyphis bicolor	3.4000E-02	25	6.8040E-03	Ehnes et al. 2011
1316	Invertebrates	Centrioptera muricata	1.3260E-01	25	8.4348E-02	Ehnes et al. 2011
1317	Invertebrates	Centropages abdominaris	8.5000E-05	15.9	8.5680E-05	Makarieva et al. 2008
1318	Invertebrates	Centropages brachiatus	1.0000E-04	14.3	1.1520E-04	Makarieva et al. 2008
1319	Invertebrates	Centropages kroyeri	3.9000E-05	20	4.7034E-05	Makarieva et al. 2008
1320	Invertebrates	Centropages trispinosus	6.6000E-05	20	2.9082E-04	Makarieva et al. 2008
1321	Invertebrates	Centropages trispinosus	6.6000E-05	29	5.5800E-04	Makarieva et al. 2008
1322	Invertebrates	Ceratoppia bipilis	6.4500E-05	10	8.5000E-06	Ehnes et al. 2011
1323	Invertebrates	Ceratoppia bipilis	9.0300E-05	10	1.5350E-05	Ehnes et al. 2011
1324	Invertebrates	Ceratozetes gracilis	2.0000E-05	10	4.8500E-06	Ehnes et al. 2011
1325	Invertebrates	Ceratozetes gracilis	2.4000E-05	5	2.5000E-06	Ehnes et al. 2011
1326	Invertebrates	Ceratozetes gracilis	2.4000E-05	10	5.9000E-06	Ehnes et al. 2011
1327	Invertebrates	Ceratozetes gracilis	2.4000E-05	15	1.0800E-05	Ehnes et al. 2011
1328	Invertebrates	Ceratozetes gracilis	2.4000E-05	20	1.5700E-05	Ehnes et al. 2011
1329	Invertebrates	Ceratozetes gracilis	2.8200E-05	15	5.4500E-06	Ehnes et al. 2011
1330	Invertebrates	Ceratozetes kananaskis	8.1000E-06	5	5.0000E-07	Ehnes et al. 2011
1331	Invertebrates	Ceratozetes kananaskis	8.1000E-06	10	9.0000E-07	Ehnes et al. 2011
1332	Invertebrates	Ceratozetes kananaskis	8.1000E-06	15	1.8000E-06	Ehnes et al. 2011
1333	Invertebrates	Ceratozetes kananaskis	8.1000E-06	20	2.2500E-06	Ehnes et al. 2011
1334	Invertebrates	Cerotalis sp.	5.6200E-01	25	8.5428E-02	Ehnes et al. 2011
1335	Invertebrates	Ceuthophilis fossor	7.0150E-02	25	1.4717E-01	Ehnes et al. 2011
1336	Invertebrates	Ceuthophilis gracilipes	2.5910E-01	25	9.7848E-02	Ehnes et al. 2011
1337	Invertebrates	Chamobates cuspidatus	6.5000E-06	5	8.0000E-07	Ehnes et al. 2011
1338	Invertebrates	Chamobates cuspidatus	6.5000E-06	10	2.1000E-06	Ehnes et al. 2011
1339	Invertebrates	Chamobates cuspidatus	6.5000E-06	15	4.7000E-06	Ehnes et al. 2011

1340	Invertebrates	Chamobates sp.	7.2000E-06	10	3.9500E-06	Ehnes et al. 2011
1341	Invertebrates	Chelaner rothsteini	2.5000E-04	25	1.9800E-04	Ehnes et al. 2011
1342	Invertebrates	Cherax destructor	5.3000E+01	20	2.8620E+00	Makarieva et al. 2008
1343	Invertebrates	Chilanthia cavernosa	1.0260E+00	25	2.8287E-01	Ehnes et al. 2011
1344	Invertebrates	Chironomus riparius	1.0000E-03	25	2.8980E-03	Ehnes et al. 2011
1345	Invertebrates	Chorismus antarcticus	2.2730E+00	-1.7	5.4416E-02	Makarieva et al. 2008
1346	Invertebrates	Chorismus antarcticus	5.0000E+00	2	2.8800E-01	Makarieva et al. 2008
1347	Invertebrates	Cicindela longilabris	1.2430E-01	25	4.8528E-02	Ehnes et al. 2011
1348	Invertebrates	Cicindela repanda	6.3400E-02	25	2.6190E-02	Ehnes et al. 2011
1349	Invertebrates	Cilliba cassidea	4.5300E-05	10	8.2000E-06	Ehnes et al. 2011
1350	Invertebrates	Circellium bacchus	7.2850E+00	25	7.2311E-01	Ehnes et al. 2011
1351	Invertebrates	Clausocalanus furcatus	1.9000E-05	20	5.0407E-05	Makarieva et al. 2008
1352	Invertebrates	Cleistosoma edwardsii	8.1800E-01	20	1.7672E-01	Makarieva et al. 2008
1353	Invertebrates	Cleistosoma edwardsii	8.1800E-01	20	1.9187E-01	Makarieva et al. 2008
1354	Invertebrates	Coeloesis biloba	3.7350E+00	25	6.7036E-01	Ehnes et al. 2011
1355	Invertebrates	Coenobita compressus	3.5000E+00	20	3.5545E-01	Makarieva et al. 2008
1356	Invertebrates	Coenobita compressus	3.7000E+00	20	5.1502E-01	Makarieva et al. 2008
1357	Invertebrates	Cognettia	5.7000E-05	20	3.9650E-05	Ehnes et al. 2011
1358	Invertebrates	Cognettia	1.9000E-04	20	8.3400E-05	Ehnes et al. 2011
1359	Invertebrates	Cognettia	3.6000E-04	20	8.5950E-05	Ehnes et al. 2011
1360	Invertebrates	Colobenema sericeum	4.2000E+00	5	8.3160E-03	Makarieva et al. 2008
1361	Invertebrates	Conchoecia sp.	4.5000E-05	20	1.0143E-04	Makarieva et al. 2008
1362	Invertebrates	Conchoecia sp.	7.9000E-05	20	8.8477E-05	Makarieva et al. 2008
1363	Invertebrates	Conocephalus fasciatus	5.1370E-02	25	1.2908E-01	Ehnes et al. 2011
1364	Invertebrates	Coptotermes formosanus	2.9500E-03	25	1.4580E-03	Ehnes et al. 2011
1365	Invertebrates	Corycaeus typicus	5.1500E-05	15	3.5995E-05	Makarieva et al. 2008

1366	Invertebrates	Corydalis cornutus	3.2600E-01	25	8.5041E-01	Ehnes et al. 2011
1367	Invertebrates	Cranchia scabra	3.5390E+01	5	2.2996E-01	Makarieva et al. 2008
1368	Invertebrates	Crangon abyssorum	1.2000E+00	7.5	1.6848E-02	Makarieva et al. 2008
1369	Invertebrates	Crangon abyssorum	1.2800E+00	2	2.0506E-02	Makarieva et al. 2008
1370	Invertebrates	Crangon affinis	5.0600E-02	20	9.1189E-03	Makarieva et al. 2008
1371	Invertebrates	Crangon communis	2.1400E+00	15	1.8836E-01	Makarieva et al. 2008
1372	Invertebrates	Crangon communis	2.9100E+00	10	9.8998E-02	Makarieva et al. 2008
1373	Invertebrates	Crangon communis	4.6900E+00	5	1.5027E-01	Makarieva et al. 2008
1374	Invertebrates	Crepidogaster bioculata	1.7100E-01	25	5.1570E-02	Ehnes et al. 2011
1375	Invertebrates	Crossota alba	5.3000E-01	5	3.9114E-03	Makarieva et al. 2008
1376	Invertebrates	Crossota rufobrunnea	2.6000E-01	5	8.8920E-04	Makarieva et al. 2008
1377	Invertebrates	Crossota sp. A	1.4000E-01	5	5.2920E-04	Makarieva et al. 2008
1378	Invertebrates	Cryptoglossa verrucosa	7.0000E-01	25	7.5384E-02	Ehnes et al. 2011
1379	Invertebrates	Cryptopygus antarcticus	2.7900E-06	10	1.5000E-06	Ehnes et al. 2011
1380	Invertebrates	Cryptopygus antarcticus	2.9970E-06	10	4.8750E-05	Ehnes et al. 2011
1381	Invertebrates	Cryptopygus antarcticus	2.9970E-06	6	1.6700E-05	Ehnes et al. 2011
1382	Invertebrates	Cryptopygus antarcticus	3.0000E-06	0	7.0000E-07	Ehnes et al. 2011
1383	Invertebrates	Cryptopygus antarcticus	3.0000E-06	5	1.4000E-06	Ehnes et al. 2011
1384	Invertebrates	Cryptopygus antarcticus	3.0000E-06	10	1.3500E-06	Ehnes et al. 2011
1385	Invertebrates	Cryptopygus antarcticus	3.0000E-06	2	4.2500E-06	Ehnes et al. 2011
1386	Invertebrates	Cryptopygus antarcticus	3.0400E-06	5	1.4500E-06	Ehnes et al. 2011
1387	Invertebrates	Cryptopygus antarcticus	3.0900E-06	20	2.0000E-06	Ehnes et al. 2011
1388	Invertebrates	Cryptopygus antarcticus	3.8300E-06	5	1.0000E-06	Ehnes et al. 2011
1389	Invertebrates	Cryptopygus antarcticus	1.0200E-05	0	1.7000E-06	Ehnes et al. 2011
1390	Invertebrates	Cryptopygus antarcticus	1.0200E-05	5	3.2500E-06	Ehnes et al. 2011
1391	Invertebrates	Cryptopygus antarcticus	1.0200E-05	10	3.7000E-06	Ehnes et al. 2011

1392	Invertebrates	Cryptopygus antarcticus	1.0260E-05	5	3.2500E-06	Ehnes et al. 2011
1393	Invertebrates	Cryptopygus antarcticus	1.0323E-05	10	5.0150E-05	Ehnes et al. 2011
1394	Invertebrates	Cryptopygus antarcticus	1.0323E-05	2	8.7000E-06	Ehnes et al. 2011
1395	Invertebrates	Cryptopygus antarcticus	1.0323E-05	6	2.1100E-05	Ehnes et al. 2011
1396	Invertebrates	Cryptopygus antarcticus	1.1090E-05	5	2.7000E-06	Ehnes et al. 2011
1397	Invertebrates	Cryptopygus antarcticus	1.1650E-05	20	9.0500E-06	Ehnes et al. 2011
1398	Invertebrates	Cryptopygus antarcticus	1.1780E-05	10	3.9000E-06	Ehnes et al. 2011
1399	Invertebrates	Cryptopygus antarcticus	1.9510E-05	20	1.3000E-05	Ehnes et al. 2011
1400	Invertebrates	Cryptopygus antarcticus	2.3120E-05	5	5.6000E-06	Ehnes et al. 2011
1401	Invertebrates	Cryptopygus antarcticus	2.5641E-05	10	5.0200E-05	Ehnes et al. 2011
1402	Invertebrates	Cryptopygus antarcticus	2.5641E-05	2	1.4600E-05	Ehnes et al. 2011
1403	Invertebrates	Cryptopygus antarcticus	2.5641E-05	6	2.4750E-05	Ehnes et al. 2011
1404	Invertebrates	Cryptopygus antarcticus	2.5700E-05	0	3.4000E-06	Ehnes et al. 2011
1405	Invertebrates	Cryptopygus antarcticus	2.5700E-05	5	6.0000E-06	Ehnes et al. 2011
1406	Invertebrates	Cryptopygus antarcticus	2.5700E-05	10	7.9000E-06	Ehnes et al. 2011
1407	Invertebrates	Cryptopygus antarcticus	2.5720E-05	5	6.0000E-06	Ehnes et al. 2011
1408	Invertebrates	Cryptopygus antarcticus	2.6130E-05	10	8.1500E-06	Ehnes et al. 2011
1409	Invertebrates	Cryptopygus antarcticus	4.0980E-05	20	2.0050E-05	Ehnes et al. 2011
1410	Invertebrates	Cryptopygus antarcticus	4.6580E-05	10	1.0050E-05	Ehnes et al. 2011
1411	Invertebrates	Cryptopygus antarcticus	5.2500E-05	0	5.8500E-06	Ehnes et al. 2011
1412	Invertebrates	Cryptopygus antarcticus	5.2500E-05	5	9.6500E-06	Ehnes et al. 2011
1413	Invertebrates	Cryptopygus antarcticus	5.2500E-05	10	1.4300E-05	Ehnes et al. 2011
1414	Invertebrates	Cryptopygus antarcticus	5.2570E-05	5	9.7000E-06	Ehnes et al. 2011
1415	Invertebrates	Cryptopygus antarcticus	5.2614E-05	10	5.0350E-05	Ehnes et al. 2011
1416	Invertebrates	Cryptopygus antarcticus	5.2614E-05	2	2.2100E-05	Ehnes et al. 2011
1417	Invertebrates	Cryptopygus antarcticus	5.2614E-05	6	2.8250E-05	Ehnes et al. 2011

1418	Invertebrates	Cryptopygus antarcticus	9.2574E-05	10	5.5000E-05	Ehnes et al. 2011
1419	Invertebrates	Cryptopygus antarcticus	9.2574E-05	2	3.0650E-05	Ehnes et al. 2011
1420	Invertebrates	Cryptopygus antarcticus	9.2574E-05	6	3.1400E-05	Ehnes et al. 2011
1421	Invertebrates	Cryptopygus antarcticus	9.2800E-05	0	8.9500E-06	Ehnes et al. 2011
1422	Invertebrates	Cryptopygus antarcticus	9.2800E-05	5	1.4150E-05	Ehnes et al. 2011
1423	Invertebrates	Cryptopygus antarcticus	9.2800E-05	10	2.2850E-05	Ehnes et al. 2011
1424	Invertebrates	Cryptopygus antarcticus	9.8810E-05	5	1.5000E-05	Ehnes et al. 2011
1425	Invertebrates	Culex tarsalis	2.2300E-03	25	4.2480E-03	Ehnes et al. 2011
1426	Invertebrates	Cyclocephala sp.	2.4600E-01	25	1.0665E-01	Ehnes et al. 2011
1427	Invertebrates	Cylisticus convexus	3.4800E-02	20	7.2000E-03	Ehnes et al. 2011
1428	Invertebrates	Cylisticus convexus	3.8400E-02	20	9.0782E-03	Makarieva et al. 2008
1429	Invertebrates	Cyphocaris richardi	1.0620E+00	-1.4	4.1482E-02	Makarieva et al. 2008
1430	Invertebrates	Cyphocaris sp	3.2000E-01	0.2	1.7914E-02	Makarieva et al. 2008
1431	Invertebrates	Cypholoba bihamata	2.4500E-01	25	1.7309E-01	Ehnes et al. 2011
1432	Invertebrates	Cypholoba chanleri	3.2600E-01	25	1.4044E-01	Ehnes et al. 2011
1433	Invertebrates	Cypholoba sp.	1.9200E-01	25	1.6873E-01	Ehnes et al. 2011
1434	Invertebrates	Cypholoba tenuicollis	5.9000E-02	25	4.4226E-02	Ehnes et al. 2011
1435	Invertebrates	Cypholoba tetrastigma	1.3200E-01	25	1.4443E-01	Ehnes et al. 2011
1436	Invertebrates	Cypholoba trilunata	2.1300E-01	25	9.5436E-02	Ehnes et al. 2011
1437	Invertebrates	Cypridina hilgendorfii	1.9800E-03	30	4.8510E-03	Makarieva et al. 2008
1438	Invertebrates	Cystosoma saundersii	1.1580E+00	25	4.2212E-01	Ehnes et al. 2011
1439	Invertebrates	Damaeus clavipes	1.3300E-04	5	1.1250E-05	Ehnes et al. 2011
1440	Invertebrates	Damaeus clavipes	1.3300E-04	10	1.5950E-05	Ehnes et al. 2011
1441	Invertebrates	Damaeus clavipes	1.3300E-04	15	2.3850E-05	Ehnes et al. 2011
1442	Invertebrates	Damaeus clavipes	1.3300E-04	20	4.9750E-05	Ehnes et al. 2011
1443	Invertebrates	Damaeus clavipes	2.5800E-04	10	3.2650E-05	Ehnes et al. 2011

1444	Invertebrates	Damaeus onustus	3.3100E-05	10	7.3500E-06	Ehnes et al. 2011
1445	Invertebrates	Damaeus onustus	1.0000E-04	10	1.3250E-05	Ehnes et al. 2011
1446	Invertebrates	Damaeus onustus	2.1400E-04	10	1.8800E-05	Ehnes et al. 2011
1447	Invertebrates	Damaeus onustus	3.5300E-04	10	2.6550E-05	Ehnes et al. 2011
1448	Invertebrates	Damaeus onustus	6.2500E-04	18	5.3000E-05	Ehnes et al. 2011
1449	Invertebrates	Damaeus onustus	6.8000E-04	10	3.8300E-05	Ehnes et al. 2011
1450	Invertebrates	Damaeus onustus	7.8100E-04	10	3.2650E-05	Ehnes et al. 2011
1451	Invertebrates	Daphnia ambigua	4.7000E-06	6.5	6.2037E-06	Makarieva et al. 2008
1452	Invertebrates	Daphnia ambigua	5.8700E-06	22.9	3.0701E-05	Makarieva et al. 2008
1453	Invertebrates	Daphnia ambigua	5.9700E-06	22.9	2.9098E-05	Makarieva et al. 2008
1454	Invertebrates	Daphnia ambigua	8.9300E-06	22.9	3.5094E-05	Makarieva et al. 2008
1455	Invertebrates	Daphnia ambigua	1.3600E-05	22.9	3.6394E-05	Makarieva et al. 2008
1456	Invertebrates	Daphnia ambigua	1.7800E-05	22.9	3.8893E-05	Makarieva et al. 2008
1457	Invertebrates	Daphnia ambigua	1.8400E-05	26.6	5.0488E-05	Makarieva et al. 2008
1458	Invertebrates	Daphnia ambigua	1.9000E-05	22.9	3.8571E-05	Makarieva et al. 2008
1459	Invertebrates	Daphnia ambigua	1.9000E-05	22.9	3.8588E-05	Makarieva et al. 2008
1460	Invertebrates	Daphnia ambigua	1.9700E-05	18.2	2.1297E-05	Makarieva et al. 2008
1461	Invertebrates	Daphnia ambigua	2.2100E-05	22.9	5.6997E-05	Makarieva et al. 2008
1462	Invertebrates	Daphnia ambigua	2.5100E-05	13.3	3.5692E-05	Makarieva et al. 2008
1463	Invertebrates	Daphnia ambigua	2.5400E-05	11.3	5.8192E-05	Makarieva et al. 2008
1464	Invertebrates	Daphnia ambigua	2.5800E-05	6.5	3.4263E-05	Makarieva et al. 2008
1465	Invertebrates	Daphnia ambigua	2.6500E-05	22.9	6.9113E-05	Makarieva et al. 2008
1466	Invertebrates	Daphnia ambigua	2.6700E-05	9.9	4.9982E-05	Makarieva et al. 2008
1467	Invertebrates	Daphnia ambigua	2.8700E-05	22.9	6.4172E-05	Makarieva et al. 2008
1468	Invertebrates	Daphnia ambigua	3.0800E-05	22.9	6.6711E-05	Makarieva et al. 2008
1469	Invertebrates	Daphnia magma	5.0000E-05	20	2.1000E-04	Makarieva et al. 2008

1470	Invertebrates	Daphnia magma	6.1000E-05	20	2.8102E-04	Makarieva et al. 2008
1471	Invertebrates	Daphnia magma	6.6000E-05	25	5.6549E-04	Makarieva et al. 2008
1472	Invertebrates	Daphnia magma	8.7000E-05	15	2.9781E-04	Makarieva et al. 2008
1473	Invertebrates	Daphnia magma	1.0000E-04	20	3.8000E-04	Makarieva et al. 2008
1474	Invertebrates	Daphnia magma	1.1800E-04	10	2.6573E-04	Makarieva et al. 2008
1475	Invertebrates	Daphnia magma	1.4600E-04	5	1.8585E-04	Makarieva et al. 2008
1476	Invertebrates	Daphnia magma	1.5000E-04	20	5.1000E-04	Makarieva et al. 2008
1477	Invertebrates	Daphnia pulex	5.5000E-05	20	3.5805E-04	Makarieva et al. 2008
1478	Invertebrates	Daphnia pulex	1.4700E-04	30	2.1756E-03	Makarieva et al. 2008
1479	Invertebrates	Daphnia pulex	2.0000E-04	25	2.3064E-03	Makarieva et al. 2008
1480	Invertebrates	Daphnia pulex	2.0800E-04	20	1.7353E-03	Makarieva et al. 2008
1481	Invertebrates	Daphnia pulex	3.7400E-04	15	2.2066E-03	Makarieva et al. 2008
1482	Invertebrates	Daphnia pulex	4.8100E-04	10	1.2944E-03	Makarieva et al. 2008
1483	Invertebrates	Daphnia pulex	5.8100E-04	5	9.8483E-04	Makarieva et al. 2008
1484	Invertebrates	Daphnia pulex	6.0000E-04	21.5	1.1221E-02	Makarieva et al. 2008
1485	Invertebrates	Dasylabris sp.	8.6500E-02	25	6.7140E-03	Ehnes et al. 2011
1486	Invertebrates	Dasymutilla gloriosa	7.6450E-02	25	3.3651E-01	Ehnes et al. 2011
1487	Invertebrates	Decipisagitta decipiens	5.4000E-03	5	2.1481E-04	Makarieva et al. 2008
1488	Invertebrates	Deilephila elpenor	6.0000E-01	25	1.6078E-01	Ehnes et al. 2011
1489	Invertebrates	Deilephila euphorbiae	7.0000E-01	25	4.2262E-01	Ehnes et al. 2011
1490	Invertebrates	Dendrobaena octaedra	2.3000E-01	19	4.2350E-02	Ehnes et al. 2011
1491	Invertebrates	Dendrobaena veneta	2.3267E-01	14.6	2.9437E-02	Ehnes et al. 2011
1492	Invertebrates	Dendrobaena veneta	2.9044E-01	20	4.3135E-02	Ehnes et al. 2011
1493	Invertebrates	Dendrobaena veneta	5.8798E-01	26.3	1.1425E-01	Ehnes et al. 2011
1494	Invertebrates	Dendrobaena veneta	6.7980E-01	10	4.1678E-02	Ehnes et al. 2011
1495	Invertebrates	Dendrobaena veneta	7.0780E-01	5	2.7105E-02	Ehnes et al. 2011

1496	Invertebrates	Dendrobaena veneta	7.3059E-01	20	6.9074E-02	Ehnes et al. 2011
1497	Invertebrates	Dendrobaena veneta	7.8250E-01	15	7.7089E-02	Ehnes et al. 2011
1498	Invertebrates	Dendrobaena veneta	7.8950E-01	5	1.9673E-02	Ehnes et al. 2011
1499	Invertebrates	Dendrobaena veneta	8.3543E-01	24.8	1.5855E-01	Ehnes et al. 2011
1500	Invertebrates	Dendrobaena veneta	8.6650E-01	25	1.3319E-01	Ehnes et al. 2011
1501	Invertebrates	Dendrobaena veneta	8.7330E-01	25	3.4974E-01	Ehnes et al. 2011
1502	Invertebrates	Dendrobaena veneta	8.7351E-01	26.3	1.3727E-01	Ehnes et al. 2011
1503	Invertebrates	Dendrobaena veneta	8.9870E-01	10	3.6723E-02	Ehnes et al. 2011
1504	Invertebrates	Dendrobaena veneta	9.1480E-01	25	3.9637E-02	Ehnes et al. 2011
1505	Invertebrates	Dendrobaena veneta	9.3651E-01	26.3	1.2503E-01	Ehnes et al. 2011
1506	Invertebrates	Dendrobaena veneta	9.4010E-01	15	6.7617E-02	Ehnes et al. 2011
1507	Invertebrates	Dendrobaena veneta	9.5369E-01	5	5.3190E-02	Ehnes et al. 2011
1508	Invertebrates	Dendrobaena veneta	1.0814E+00	20	1.2212E-01	Ehnes et al. 2011
1509	Invertebrates	Dendrobaena veneta	1.0897E+00	5	4.4301E-02	Ehnes et al. 2011
1510	Invertebrates	Dendrobaena veneta	1.0902E+00	20	1.1658E-01	Ehnes et al. 2011
1511	Invertebrates	Dendrobaena veneta	1.1194E+00	14.6	9.7053E-02	Ehnes et al. 2011
1512	Invertebrates	Dendrobaena veneta	1.1279E+00	10	9.2390E-02	Ehnes et al. 2011
1513	Invertebrates	Dendrobaena veneta	1.1652E+00	25	1.3552E-01	Ehnes et al. 2011
1514	Invertebrates	Dendrobaena veneta	1.1680E+00	15	6.7617E-02	Ehnes et al. 2011
1515	Invertebrates	Dendrobaena veneta	1.1776E+00	25	1.5112E-01	Ehnes et al. 2011
1516	Invertebrates	Dendrobaena veneta	1.1783E+00	20	8.5832E-02	Ehnes et al. 2011
1517	Invertebrates	Dendrobaena veneta	1.1841E+00	15	1.1498E-01	Ehnes et al. 2011
1518	Invertebrates	Dendrobaena veneta	1.2131E+00	5	6.8345E-02	Ehnes et al. 2011
1519	Invertebrates	Dendrobaena veneta	1.2493E+00	5	4.8818E-02	Ehnes et al. 2011
1520	Invertebrates	Dendrobaena veneta	1.2976E+00	20	9.5887E-02	Ehnes et al. 2011
1521	Invertebrates	Dendrobaena veneta	1.3485E+00	10	9.5596E-02	Ehnes et al. 2011

1522	Invertebrates	Dendrobaena veneta	1.4429E+00	24.8	1.0667E-01	Ehnes et al. 2011
1523	Invertebrates	Dendrobaena veneta	1.4825E+00	10	5.2170E-02	Ehnes et al. 2011
1524	Invertebrates	Dendrobaena veneta	1.5692E+00	10	4.2552E-02	Ehnes et al. 2011
1525	Invertebrates	Dendrobaena veneta	1.6757E+00	24.8	1.3319E-01	Ehnes et al. 2011
1526	Invertebrates	Dendrocoris contaminatus	1.1000E-02	25	4.7160E-03	Ehnes et al. 2011
1527	Invertebrates	Desoria hiemalis	2.2000E-04	-2	2.4950E-05	Ehnes et al. 2011
1528	Invertebrates	Desoria saltans	9.0000E-05	-2	9.6500E-06	Ehnes et al. 2011
1529	Invertebrates	Diastylis sp.	6.0000E-04	17	3.1018E-04	Makarieva et al. 2008
1530	Invertebrates	Diceroprocta apache	6.2200E-01	25	2.8894E-01	Ehnes et al. 2011
1531	Invertebrates	Dicuspiditermes nemorosus	3.5000E-03	25	3.0600E-04	Ehnes et al. 2011
1532	Invertebrates	Dicuspiditermes santschii	2.6000E-03	25	1.0800E-04	Ehnes et al. 2011
1533	Invertebrates	Dicyrtomina minuta	2.4000E-04	6	4.6850E-05	Ehnes et al. 2011
1534	Invertebrates	Dicyrtomina minuta	2.4000E-04	10	9.3500E-05	Ehnes et al. 2011
1535	Invertebrates	Dicyrtomina minuta	2.4000E-04	15	1.5750E-04	Ehnes et al. 2011
1536	Invertebrates	Dicyrtomina minuta	5.9000E-04	8	8.7000E-05	Ehnes et al. 2011
1537	Invertebrates	Dikerogammarus haemobaphes	8.0000E-03	20	4.5946E-03	Makarieva et al. 2008
1538	Invertebrates	Diplocapsis sp.	5.3400E-03	25	1.6038E-02	Ehnes et al. 2011
1539	Invertebrates	Dirphea agis	1.9700E-01	25	8.7066E-02	Ehnes et al. 2011
1540	Invertebrates	Disseta grandis	1.1400E-02	5	3.5500E-04	Makarieva et al. 2008
1541	Invertebrates	Disseta scopularis	1.9500E-02	5	2.9133E-04	Makarieva et al. 2008
1542	Invertebrates	Disseta scopularis	2.5700E-02	1.5	6.2914E-04	Makarieva et al. 2008
1543	Invertebrates	Djerboa furcipes	1.0000E-01	0	7.5960E-03	Makarieva et al. 2008
1544	Invertebrates	Dosidicus gigas	1.2200E+04	5	9.5658E+02	Makarieva et al. 2008
1545	Invertebrates	Drosophila americana	1.1000E-03	25	2.7540E-03	Ehnes et al. 2011
1546	Invertebrates	Drosophila melanogaster	9.5000E-04	25	5.7600E-03	Ehnes et al. 2011
1547	Invertebrates	Drosophila mimica	2.4400E-03	25	8.9100E-03	Ehnes et al. 2011

1548	Invertebrates	Drosophila nikananu	4.9000E-04	25	3.4920E-03	Ehnes et al. 2011
1549	Invertebrates	Drosophila repleta	3.4300E-03	25	5.8140E-03	Ehnes et al. 2011
1550	Invertebrates	Drosophila virilis	1.6700E-03	25	8.2260E-03	Ehnes et al. 2011
1551	Invertebrates	Dryoctenes scrupulosa	1.8230E+00	25	5.0855E-01	Ehnes et al. 2011
1552	Invertebrates	Dyschirius	7.8000E-04	22	1.1658E-03	Ehnes et al. 2011
1553	Invertebrates	Dyschirius	3.5600E-03	15	1.7487E-03	Ehnes et al. 2011
1554	Invertebrates	Dyscinetus sp.	3.9000E-01	25	8.4528E-02	Ehnes et al. 2011
1555	Invertebrates	Eacles imperialis	1.1050E+00	25	5.9092E-01	Ehnes et al. 2011
1556	Invertebrates	Eburia sp.	5.8200E-01	25	2.6878E-01	Ehnes et al. 2011
1557	Invertebrates	Eciton hamatum	2.0600E-03	28	2.3040E-03	Ehnes et al. 2011
1558	Invertebrates	Eciton hamatum	2.8200E-03	28	4.2840E-03	Ehnes et al. 2011
1559	Invertebrates	Eciton hamatum	4.5300E-03	28	3.2400E-03	Ehnes et al. 2011
1560	Invertebrates	Eciton hamatum	5.5300E-03	28	5.1480E-03	Ehnes et al. 2011
1561	Invertebrates	Eciton hamatum	5.6300E-03	28	4.5000E-03	Ehnes et al. 2011
1562	Invertebrates	Eciton hamatum	5.6300E-03	28	5.5440E-03	Ehnes et al. 2011
1563	Invertebrates	Eciton hamatum	6.0000E-03	25	4.5540E-03	Ehnes et al. 2011
1564	Invertebrates	Eciton hamatum	6.0000E-03	28	6.0480E-03	Ehnes et al. 2011
1565	Invertebrates	Eciton hamatum	6.7400E-03	28	4.8600E-03	Ehnes et al. 2011
1566	Invertebrates	Eciton hamatum	8.0300E-03	28	3.2040E-03	Ehnes et al. 2011
1567	Invertebrates	Eciton hamatum	8.0300E-03	28	5.1480E-03	Ehnes et al. 2011
1568	Invertebrates	Eciton hamatum	8.2400E-03	28	4.4460E-03	Ehnes et al. 2011
1569	Invertebrates	Eciton hamatum	8.6600E-03	28	4.1400E-03	Ehnes et al. 2011
1570	Invertebrates	Eciton hamatum	9.0800E-03	28	3.4380E-03	Ehnes et al. 2011
1571	Invertebrates	Eciton hamatum	9.0800E-03	28	7.9920E-03	Ehnes et al. 2011
1572	Invertebrates	Eciton hamatum	9.2300E-03	28	4.3920E-03	Ehnes et al. 2011
1573	Invertebrates	Eciton hamatum	9.7100E-03	28	6.6240E-03	Ehnes et al. 2011

1574	Invertebrates	Eciton hamatum	1.1640E-02	28	7.7580E-03	Ehnes et al. 2011
1575	Invertebrates	Eciton hamatum	1.2120E-02	28	6.3720E-03	Ehnes et al. 2011
1576	Invertebrates	Eciton hamatum	1.4030E-02	28	8.2080E-03	Ehnes et al. 2011
1577	Invertebrates	Eciton hamatum	1.4650E-02	28	1.6020E-02	Ehnes et al. 2011
1578	Invertebrates	Eciton hamatum	1.4870E-02	28	1.0080E-02	Ehnes et al. 2011
1579	Invertebrates	Eciton hamatum	1.8630E-02	28	1.5318E-02	Ehnes et al. 2011
1580	Invertebrates	Eciton hamatum	2.2500E-02	28	1.9926E-02	Ehnes et al. 2011
1581	Invertebrates	Eciton hamatum	2.3820E-02	28	1.9854E-02	Ehnes et al. 2011
1582	Invertebrates	Eciton hamatum	2.3880E-02	28	1.7064E-02	Ehnes et al. 2011
1583	Invertebrates	Ectemnorhinus marioni	9.6000E-03	25	4.9140E-03	Ehnes et al. 2011
1584	Invertebrates	Ectemnorhinus similis	1.3400E-02	25	5.8500E-03	Ehnes et al. 2011
1585	Invertebrates	Edrotes ventricosus	2.4350E-02	25	1.6542E-02	Ehnes et al. 2011
1586	Invertebrates	Eirene mollis	2.2000E-01	15	3.8412E-03	Makarieva et al. 2008
1587	Invertebrates	Eisenia foetida	3.6670E-02	5.4	6.2662E-03	Ehnes et al. 2011
1588	Invertebrates	Eisenia foetida	4.2200E-02	14.6	4.5175E-03	Ehnes et al. 2011
1589	Invertebrates	Eisenia foetida	6.5180E-02	14.6	1.0201E-02	Ehnes et al. 2011
1590	Invertebrates	Eisenia foetida	6.7560E-02	5.4	9.4722E-03	Ehnes et al. 2011
1591	Invertebrates	Eisenia foetida	9.4500E-02	14.6	1.0347E-02	Ehnes et al. 2011
1592	Invertebrates	Eisenia foetida	1.0789E-01	24.8	1.6321E-02	Ehnes et al. 2011
1593	Invertebrates	Eisenia foetida	1.1187E-01	24.9	3.6869E-02	Ehnes et al. 2011
1594	Invertebrates	Eisenia foetida	1.8864E-01	24.8	3.7743E-02	Ehnes et al. 2011
1595	Invertebrates	Eisenia foetida	2.0034E-01	24.8	4.0075E-02	Ehnes et al. 2011
1596	Invertebrates	Eisenia foetida	2.3519E-01	14.6	1.7341E-02	Ehnes et al. 2011
1597	Invertebrates	Eisenia foetida	2.5391E-01	14.6	1.4864E-02	Ehnes et al. 2011
1598	Invertebrates	Eisenia foetida	2.5683E-01	5.4	8.3064E-03	Ehnes et al. 2011
1599	Invertebrates	Eisenia foetida	2.6538E-01	5	1.0784E-02	Ehnes et al. 2011

1600	Invertebrates	Eisenia foetida	2.6680E-01	10	8.7436E-03	Ehnes et al. 2011
1601	Invertebrates	Eisenia foetida	3.0730E-01	25	6.0330E-02	Ehnes et al. 2011
1602	Invertebrates	Eisenia foetida	3.1866E-01	5.4	8.8893E-03	Ehnes et al. 2011
1603	Invertebrates	Eisenia foetida	3.2340E-01	25	5.7416E-02	Ehnes et al. 2011
1604	Invertebrates	Eisenia foetida	3.3403E-01	24.8	6.4556E-02	Ehnes et al. 2011
1605	Invertebrates	Eisenia foetida	3.3500E-01	19	3.1550E-02	Ehnes et al. 2011
1606	Invertebrates	Eisenia foetida	3.3500E-01	15	3.5200E-02	Ehnes et al. 2011
1607	Invertebrates	Eisenia foetida	3.4400E-01	20	4.7798E-02	Ehnes et al. 2011
1608	Invertebrates	Eisenia foetida	3.5543E-01	14.6	1.9819E-02	Ehnes et al. 2011
1609	Invertebrates	Eisenia foetida	3.6420E-01	20	5.9310E-02	Ehnes et al. 2011
1610	Invertebrates	Eisenia foetida	3.7790E-01	10	5.0712E-02	Ehnes et al. 2011
1611	Invertebrates	Eisenia foetida	3.8210E-01	20	5.7124E-02	Ehnes et al. 2011
1612	Invertebrates	Eisenia foetida	4.0240E-01	20	5.0567E-02	Ehnes et al. 2011
1613	Invertebrates	Eisenia foetida	4.0656E-01	14.6	2.7834E-02	Ehnes et al. 2011
1614	Invertebrates	Eisenia foetida	4.0693E-01	10	1.8944E-02	Ehnes et al. 2011
1615	Invertebrates	Eisenia foetida	4.1795E-01	5	1.1950E-02	Ehnes et al. 2011
1616	Invertebrates	Eisenia foetida	4.2858E-01	10	2.5356E-02	Ehnes et al. 2011
1617	Invertebrates	Eisenia foetida	4.4040E-01	25	6.2225E-02	Ehnes et al. 2011
1618	Invertebrates	Eisenia foetida	4.4300E-01	25	5.5521E-02	Ehnes et al. 2011
1619	Invertebrates	Eisenia foetida	4.5803E-01	10	2.3753E-02	Ehnes et al. 2011
1620	Invertebrates	Eisenia foetida	5.0030E-01	25	1.1104E-01	Ehnes et al. 2011
1621	Invertebrates	Eisenia foetida	5.4401E-01	10	2.0256E-02	Ehnes et al. 2011
1622	Invertebrates	Eisenia foetida	5.4490E-01	25	8.7144E-02	Ehnes et al. 2011
1623	Invertebrates	Eisenia foetida	6.1000E-01	21	4.6351E-02	Ehnes et al. 2011
1624	Invertebrates	Eisenia foetida	6.2449E-01	14.6	5.8873E-02	Ehnes et al. 2011
1625	Invertebrates	Eisenia foetida	6.3000E-01	27	8.1466E-02	Ehnes et al. 2011

1626	Invertebrates	Eisenia foetida	6.6000E-01	24	6.3909E-02	Ehnes et al. 2011
1627	Invertebrates	Eisenia foetida	6.7000E-01	15	3.0199E-02	Ehnes et al. 2011
1628	Invertebrates	Eisenia foetida	6.8000E-01	30	1.2220E-01	Ehnes et al. 2011
1629	Invertebrates	Eisenia foetida	7.4000E-01	36	1.2220E-01	Ehnes et al. 2011
1630	Invertebrates	Eisenia foetida	7.5000E-01	18	4.4244E-02	Ehnes et al. 2011
1631	Invertebrates	Eisenia foetida	7.6000E-01	33	1.6434E-01	Ehnes et al. 2011
1632	Invertebrates	Eisenia foetida	7.8000E-01	12	2.9496E-02	Ehnes et al. 2011
1633	Invertebrates	Eisenia rosea	3.3500E-01	19	3.1450E-02	Ehnes et al. 2011
1634	Invertebrates	Eiseniella tetraedra	1.1000E-01	19	1.5600E-02	Ehnes et al. 2011
1635	Invertebrates	Eiseniella tetraedra	1.1000E-01	18	8.0061E-03	Ehnes et al. 2011
1636	Invertebrates	Eiseniella tetraedra	1.1000E-01	21	1.1096E-02	Ehnes et al. 2011
1637	Invertebrates	Eiseniella tetraedra	1.1000E-01	30	1.4116E-02	Ehnes et al. 2011
1638	Invertebrates	Eiseniella tetraedra	1.2000E-01	24	1.4608E-02	Ehnes et al. 2011
1639	Invertebrates	Eiseniella tetraedra	1.3000E-01	15	6.5313E-03	Ehnes et al. 2011
1640	Invertebrates	Eiseniella tetraedra	1.4000E-01	12	6.0397E-03	Ehnes et al. 2011
1641	Invertebrates	Eiseniella tetraedra	1.5000E-01	27	2.1139E-02	Ehnes et al. 2011
1642	Invertebrates	Eledone cirrhosa	1.5000E+02	5	3.9312E+00	Makarieva et al. 2008
1643	Invertebrates	Eledonella pygmaea	2.0200E+01	5	2.2543E-02	Makarieva et al. 2008
1644	Invertebrates	Eleodes armata	9.1700E-01	25	1.4825E-01	Ehnes et al. 2011
1645	Invertebrates	Eleodes grandicollis	5.2193E-01	25	4.5666E-01	Ehnes et al. 2011
1646	Invertebrates	Eleodes sp.	8.8850E-02	25	6.4116E-02	Ehnes et al. 2011
1647	Invertebrates	Eleodes tenebrosa	5.1530E-02	25	4.4532E-02	Ehnes et al. 2011
1648	Invertebrates	Encoptolophus s. costalis	1.7460E-01	25	8.3268E-02	Ehnes et al. 2011
1649	Invertebrates	Enyaliopsis petersi	7.5000E-01	25	2.7463E-01	Ehnes et al. 2011
1650	Invertebrates	Enyo ocypete	4.5333E-01	25	2.0677E-01	Ehnes et al. 2011
1651	Invertebrates	Epiphysa arenicola	1.2370E+00	25	8.4492E-02	Ehnes et al. 2011

1652	Invertebrates	Ereynetes macquariensis	1.5000E-06	0	1.6500E-06	Ehnes et al. 2011
1653	Invertebrates	Ereynetes macquariensis	2.0000E-06	0	1.9500E-06	Ehnes et al. 2011
1654	Invertebrates	Ereynetes macquariensis	2.0000E-06	5	2.6000E-06	Ehnes et al. 2011
1655	Invertebrates	Ereynetes macquariensis	2.0000E-06	10	2.7500E-06	Ehnes et al. 2011
1656	Invertebrates	Erinnyis ello	1.2100E+00	25	7.4437E-01	Ehnes et al. 2011
1657	Invertebrates	Erinnyis oenotrus	9.6400E-01	25	6.5079E-01	Ehnes et al. 2011
1658	Invertebrates	Eriphia spinifrons	3.5000E+02	20	1.2159E+01	Makarieva et al. 2008
1659	Invertebrates	Erodius nanus	2.1900E-02	25	1.8234E-02	Ehnes et al. 2011
1660	Invertebrates	Erythemis simplicicollis	2.6300E-01	25	1.5795E-01	Ehnes et al. 2011
1661	Invertebrates	Erythrodiplax berenice	1.2500E-01	25	7.8984E-02	Ehnes et al. 2011
1662	Invertebrates	Erythrodiplax connata	5.2000E-02	25	3.4470E-02	Ehnes et al. 2011
1663	Invertebrates	Euatideus giesbrechti	2.1500E-04	20	1.6254E-04	Makarieva et al. 2008
1664	Invertebrates	Euaugaptilus antarcticus	2.2500E-02	5	5.1840E-04	Makarieva et al. 2008
1665	Invertebrates	Euaugaptilus magnus	3.0900E-02	5	4.0046E-04	Makarieva et al. 2008
1666	Invertebrates	Euaugaptilus nodifrons	5.9700E-02	5	7.5222E-04	Makarieva et al. 2008
1667	Invertebrates	Eublaberus posticus	2.2000E+00	25	7.5951E-01	Ehnes et al. 2011
1668	Invertebrates	Euborellia annulipes	3.1760E-02	25	1.4922E-02	Ehnes et al. 2011
1669	Invertebrates	Eucalanus attenuatus	6.8500E-04	27.4	9.0502E-04	Makarieva et al. 2008
1670	Invertebrates	Eucalanus attenuatus	1.3890E-03	15	2.9977E-04	Makarieva et al. 2008
1671	Invertebrates	Eucalanus bungii	5.0000E-03	6	7.8300E-04	Makarieva et al. 2008
1672	Invertebrates	Eucalanus bungii	8.0700E-03	5.75	8.5558E-04	Makarieva et al. 2008
1673	Invertebrates	Eucalanus bungii	8.2200E-03	6.3	7.4868E-04	Makarieva et al. 2008
1674	Invertebrates	Eucalanus crassus	2.0700E-03	30.2	5.7753E-03	Makarieva et al. 2008
1675	Invertebrates	Eucalanus elongatus	5.4000E-04	15	2.5301E-04	Makarieva et al. 2008
1676	Invertebrates	Eucalanus elongatus	1.7860E-03	20	4.2500E-04	Makarieva et al. 2008
1677	Invertebrates	Eucalanus marina	8.2000E-04	28.75	2.2386E-03	Makarieva et al. 2008

1678	Invertebrates	Eucalanus marina	1.4300E-03	28	2.6883E-03	Makarieva et al. 2008
1679	Invertebrates	Eucalanus monachus	1.4500E-03	30.2	6.2930E-03	Makarieva et al. 2008
1680	Invertebrates	Eucalanus mucronatus	7.2000E-04	30	6.9841E-04	Makarieva et al. 2008
1681	Invertebrates	Eucalanus subcrassus	1.9100E-04	20	4.4316E-04	Makarieva et al. 2008
1682	Invertebrates	Eucalanus subcrassus	1.9100E-04	29	8.6397E-04	Makarieva et al. 2008
1683	Invertebrates	Eucalanus subcrassus	5.2000E-04	24	6.5520E-04	Makarieva et al. 2008
1684	Invertebrates	Euchaeta antarctica	3.0000E-03	0	9.6120E-05	Makarieva et al. 2008
1685	Invertebrates	Euchaeta marina	1.4600E-03	24	1.3403E-03	Makarieva et al. 2008
1686	Invertebrates	Euchaeta plana	9.5000E-04	28	2.6030E-03	Makarieva et al. 2008
1687	Invertebrates	Euchaeta plana	3.0000E-03	20	4.2611E-04	Makarieva et al. 2008
1688	Invertebrates	Euchirella amoena	2.6600E-03	28	5.3999E-03	Makarieva et al. 2008
1689	Invertebrates	Euchirella bitumida	9.4000E-03	5	5.0591E-04	Makarieva et al. 2008
1690	Invertebrates	Euchirella bitumida	1.2800E-02	1.5	6.4051E-04	Makarieva et al. 2008
1691	Invertebrates	Euchirella maxima	1.8800E-02	5	8.5615E-04	Makarieva et al. 2008
1692	Invertebrates	Euchirella rostrata	1.5800E-03	15	4.5987E-04	Makarieva et al. 2008
1693	Invertebrates	Euchirella rostrata	4.1000E-03	6.5	9.2250E-04	Makarieva et al. 2008
1694	Invertebrates	Euchirella rostrata	4.5000E-03	6.5	9.7200E-04	Makarieva et al. 2008
1695	Invertebrates	Euchirella rostrata	4.7000E-03	6.5	1.3536E-03	Makarieva et al. 2008
1696	Invertebrates	Euconocephalus nasutus	6.5000E-01	25	1.8481E-01	Ehnes et al. 2011
1697	Invertebrates	Eucyllus unicolor	2.5900E-03	25	2.3760E-03	Ehnes et al. 2011
1698	Invertebrates	Eucyllus vagans	7.4300E-03	25	5.7240E-03	Ehnes et al. 2011
1699	Invertebrates	Eugaster loricatus	1.5800E+00	25	3.1718E-01	Ehnes et al. 2011
1700	Invertebrates	Euglyphis sp.	8.7000E-02	25	7.3998E-02	Ehnes et al. 2011
1701	Invertebrates	Eukrohnia bathypelagica	2.6600E-02	5.5	2.8728E-04	Makarieva et al. 2008
1702	Invertebrates	Eukrohnia fowleri	9.4000E-02	5	7.2756E-04	Makarieva et al. 2008
1703	Invertebrates	Eukrohnia hamata	4.0000E-02	5.5	3.9600E-04	Makarieva et al. 2008

1704	Invertebrates	Euphausia crystallorophias	3.0000E-02	-1.4	3.1806E-03	Makarieva et al. 2008
1705	Invertebrates	Euphausia crystallorophias	5.0000E-02	-0.5	2.7990E-03	Makarieva et al. 2008
1706	Invertebrates	Euphausia crystallorophias	5.3000E-02	-1	1.0704E-02	Makarieva et al. 2008
1707	Invertebrates	Euphausia crystallorophias	7.9000E-02	-1.7	9.7123E-03	Makarieva et al. 2008
1708	Invertebrates	Euphausia crystallorophias	9.0000E-02	-1.5	8.8128E-03	Makarieva et al. 2008
1709	Invertebrates	Euphausia crystallorophias	2.1100E-01	-1.7	2.4915E-02	Makarieva et al. 2008
1710	Invertebrates	Euphausia crystallorophias	2.4900E-01	-1.7	3.7604E-02	Makarieva et al. 2008
1711	Invertebrates	Euphausia mutica	3.2600E-03	28.75	1.1051E-02	Makarieva et al. 2008
1712	Invertebrates	Euphausia pacifica	1.7700E-02	17.2	8.4429E-03	Makarieva et al. 2008
1713	Invertebrates	Euphausia pacifica	3.3800E-02	10	2.8777E-03	Makarieva et al. 2008
1714	Invertebrates	Euphausia pacifica	3.3800E-02	10	1.9736E-02	Makarieva et al. 2008
1715	Invertebrates	Euphausia superba	1.4500E-01	-1.1	1.3102E-02	Makarieva et al. 2008
1716	Invertebrates	Euphausia superba	3.6900E-01	-1.1	3.3941E-02	Makarieva et al. 2008
1717	Invertebrates	Euphausia superba	6.1600E-01	-1.1	5.8212E-02	Makarieva et al. 2008
1718	Invertebrates	Euphausia superba	1.0100E+00	-1.1	9.1264E-02	Makarieva et al. 2008
1719	Invertebrates	Euphausia superba	1.4300E+00	-1	1.2355E-01	Makarieva et al. 2008
1720	Invertebrates	Euphausia tricantha	3.2000E-01	0.1	2.5344E-02	Makarieva et al. 2008
1721	Invertebrates	Eupodes minutus	2.0000E-06	0	2.0000E-06	Ehnes et al. 2011
1722	Invertebrates	Eupodes minutus	2.0000E-06	5	1.8500E-06	Ehnes et al. 2011
1723	Invertebrates	Eupterotegaeus rostratus	3.4700E-05	15	5.2500E-06	Ehnes et al. 2011
1724	Invertebrates	Eurymera monticulosa	2.3500E-01	1	2.7241E-02	Makarieva et al. 2008
1725	Invertebrates	Eurythenes gryllus	9.5000E-01	-1	8.6526E-02	Makarieva et al. 2008
1726	Invertebrates	Eusattus dubius	2.7220E-02	25	5.0868E-02	Ehnes et al. 2011
1727	Invertebrates	Euschides luctata	8.3800E-02	25	8.3664E-02	Ehnes et al. 2011
1728	Invertebrates	Eusirus perdentatus	1.6280E+00	-1	3.8974E-02	Makarieva et al. 2008
1729	Invertebrates	Euterpina acutifrons	1.0700E-05	15	5.7992E-06	Makarieva et al. 2008

1730	Invertebrates	Euthemisto compressa	2.2000E-02	4	3.0492E-03	Makarieva et al. 2008
1731	Invertebrates	Euthemisto compressa	2.4000E-02	4	3.5856E-03	Makarieva et al. 2008
1732	Invertebrates	Euthemisto compressa	2.5000E-02	4	3.8700E-03	Makarieva et al. 2008
1733	Invertebrates	Euthemisto libellula	4.5500E-03	6.7	2.5479E-03	Makarieva et al. 2008
1734	Invertebrates	Euthemisto libellula	7.9100E-03	5.75	1.7712E-03	Makarieva et al. 2008
1735	Invertebrates	Euthemisto libellula	1.7000E-02	-0.1	2.5398E-03	Makarieva et al. 2008
1736	Invertebrates	Euzetes globulus	3.2000E-04	18	5.2000E-05	Ehnes et al. 2011
1737	Invertebrates	Euzetes globulus	3.3000E-04	0	4.1500E-06	Ehnes et al. 2011
1738	Invertebrates	Euzetes globulus	3.3000E-04	5	1.0300E-05	Ehnes et al. 2011
1739	Invertebrates	Euzetes globulus	3.3000E-04	10	1.9350E-05	Ehnes et al. 2011
1740	Invertebrates	Euzetes globulus	3.3000E-04	15	3.7700E-05	Ehnes et al. 2011
1741	Invertebrates	Euzetes globulus	3.3000E-04	25	1.0450E-04	Ehnes et al. 2011
1742	Invertebrates	Euzetes globulus	3.4800E-04	10	4.5950E-05	Ehnes et al. 2011
1743	Invertebrates	Evarthrus sodalis	1.6410E-01	25	3.3606E-02	Ehnes et al. 2011
1744	Invertebrates	Eviphis ostrinus	2.7500E-05	10	1.2200E-05	Ehnes et al. 2011
1745	Invertebrates	Fidicina mannifera	2.8400E+00	25	1.7442E+00	Ehnes et al. 2011
1746	Invertebrates	Flaccisagitta hexaptera	1.9300E-01	5	2.5360E-03	Makarieva et al. 2008
1747	Invertebrates	Folsomia manolachei	3.0000E-05	6	4.4000E-06	Ehnes et al. 2011
1748	Invertebrates	Folsomia manolachei	3.0000E-05	10	1.3050E-05	Ehnes et al. 2011
1749	Invertebrates	Folsomia manolachei	3.0000E-05	15	4.4300E-05	Ehnes et al. 2011
1750	Invertebrates	Folsomia quadrioculata	3.0000E-05	10	9.0000E-06	Ehnes et al. 2011
1751	Invertebrates	Folsomia quadrioculata	3.0000E-05	15	1.9050E-05	Ehnes et al. 2011
1752	Invertebrates	Folsomia quadrioculata	3.0000E-05	6	7.4000E-06	Ehnes et al. 2011
1753	Invertebrates	Folsomia quadrioculata	3.0000E-05	10	9.2500E-06	Ehnes et al. 2011
1754	Invertebrates	Folsomia quadrioculata	3.0000E-05	15	1.7100E-05	Ehnes et al. 2011
1755	Invertebrates	Folsomia quadrioculata	3.0000E-05	6	6.2500E-06	Ehnes et al. 2011

1756	Invertebrates	Forelius foetidus	1.0000E-04	25	2.3400E-04	Ehnes et al. 2011
1757	Invertebrates	Formica exsecta	4.0500E-03	25	3.9420E-03	Ehnes et al. 2011
1758	Invertebrates	Formica fusca	1.3500E-03	25	5.6340E-03	Ehnes et al. 2011
1759	Invertebrates	Formica occulta	1.3000E-03	25	3.5100E-03	Ehnes et al. 2011
1760	Invertebrates	Formica pratensis	7.1700E-03	25	5.6520E-03	Ehnes et al. 2011
1761	Invertebrates	Formica rufa	4.2000E-03	25	2.0880E-03	Ehnes et al. 2011
1762	Invertebrates	Formica rufa	5.4000E-03	25	2.7900E-03	Ehnes et al. 2011
1763	Invertebrates	Formica rufa	6.6000E-03	25	2.4660E-03	Ehnes et al. 2011
1764	Invertebrates	Formica rufa	6.6000E-03	25	2.6820E-03	Ehnes et al. 2011
1765	Invertebrates	Formica rufa	6.6000E-03	25	2.4660E-03	Ehnes et al. 2011
1766	Invertebrates	Formica rufa	6.8000E-03	25	2.2320E-03	Ehnes et al. 2011
1767	Invertebrates	Formica rufa	6.8000E-03	25	1.9800E-03	Ehnes et al. 2011
1768	Invertebrates	Formica rufa	7.0000E-03	25	3.1320E-03	Ehnes et al. 2011
1769	Invertebrates	Formica rufa	8.4000E-03	25	2.7540E-03	Ehnes et al. 2011
1770	Invertebrates	Formica rufa	1.0900E-02	25	3.4380E-03	Ehnes et al. 2011
1771	Invertebrates	Formica rufa	1.0900E-02	25	3.2220E-03	Ehnes et al. 2011
1772	Invertebrates	Formica rufa	1.2900E-02	25	4.2300E-03	Ehnes et al. 2011
1773	Invertebrates	Formica rufa	1.3000E-02	25	5.2380E-03	Ehnes et al. 2011
1774	Invertebrates	Formica rufa	1.3100E-02	25	4.6260E-03	Ehnes et al. 2011
1775	Invertebrates	Formica rufa	1.3100E-02	25	4.7880E-03	Ehnes et al. 2011
1776	Invertebrates	Formica rufa	1.3800E-02	25	4.3200E-03	Ehnes et al. 2011
1777	Invertebrates	Formica rufa	1.6800E-02	25	4.1400E-03	Ehnes et al. 2011
1778	Invertebrates	Formica rufa	1.6900E-02	25	5.6520E-03	Ehnes et al. 2011
1779	Invertebrates	Formica rufa	1.7200E-02	25	3.7620E-03	Ehnes et al. 2011
1780	Invertebrates	Formica rufa	1.9300E-02	25	6.1560E-03	Ehnes et al. 2011
1781	Invertebrates	Frontinella communis	5.6700E-03	22	3.8700E-02	Ehnes et al. 2011

1782	Invertebrates	Gaetanus antarcticus	2.5700E-02	1.5	4.1634E-04	Makarieva et al. 2008
1783	Invertebrates	Gaetanus antarcticus	2.5700E-02	5	7.2628E-04	Makarieva et al. 2008
1784	Invertebrates	Gaetanus kruppi	7.2000E-03	5	2.2291E-04	Makarieva et al. 2008
1785	Invertebrates	Gaetanus pileatus	8.5000E-03	5	8.1549E-04	Makarieva et al. 2008
1786	Invertebrates	Gaetanus tenuispinus	3.6400E-03	0	3.6691E-05	Makarieva et al. 2008
1787	Invertebrates	Galiteuthis phyllura	5.1900E+00	5	7.0906E-02	Makarieva et al. 2008
1788	Invertebrates	Galleria mellonella	5.3600E-02	25	1.1430E-01	Ehnes et al. 2011
1789	Invertebrates	Galumna sp.	4.4500E-05	10	5.9500E-06	Ehnes et al. 2011
1790	Invertebrates	Gamasellus racovitzai	4.4000E-06	0	3.2500E-06	Ehnes et al. 2011
1791	Invertebrates	Gamasellus racovitzai	4.4000E-06	5	4.6000E-06	Ehnes et al. 2011
1792	Invertebrates	Gamasellus racovitzai	4.4000E-06	10	7.2000E-06	Ehnes et al. 2011
1793	Invertebrates	Gamasellus racovitzai	2.3650E-05	0	9.0000E-06	Ehnes et al. 2011
1794	Invertebrates	Gamasellus racovitzai	2.3650E-05	5	9.1000E-06	Ehnes et al. 2011
1795	Invertebrates	Gamasellus racovitzai	2.3650E-05	10	2.2800E-05	Ehnes et al. 2011
1796	Invertebrates	Gamasellus racovitzai	5.4640E-05	0	1.9500E-05	Ehnes et al. 2011
1797	Invertebrates	Gamasellus racovitzai	5.4640E-05	5	1.8050E-05	Ehnes et al. 2011
1798	Invertebrates	Gamasellus racovitzai	5.4640E-05	10	1.9800E-05	Ehnes et al. 2011
1799	Invertebrates	Gamasellus racovitzai	1.0220E-04	0	2.5000E-05	Ehnes et al. 2011
1800	Invertebrates	Gamasellus racovitzai	1.0220E-04	5	3.1900E-05	Ehnes et al. 2011
1801	Invertebrates	Gamasellus racovitzai	1.0220E-04	10	4.6350E-05	Ehnes et al. 2011
1802	Invertebrates	Gamasellus racovitzai	1.0880E-04	0	3.0000E-05	Ehnes et al. 2011
1803	Invertebrates	Gamasellus racovitzai	1.0880E-04	5	4.1950E-05	Ehnes et al. 2011
1804	Invertebrates	Gamasellus racovitzai	1.0880E-04	10	6.9000E-05	Ehnes et al. 2011
1805	Invertebrates	Gamasellus racovitzai	1.1550E-04	0	2.4100E-05	Ehnes et al. 2011
1806	Invertebrates	Gamasellus racovitzai	1.1550E-04	5	3.9150E-05	Ehnes et al. 2011
1807	Invertebrates	Gamasellus racovitzai	1.1550E-04	10	7.5500E-05	Ehnes et al. 2011

1808	Invertebrates	Gammarus duebeni	5.0000E-02	10	9.9000E-03	Makarieva et al. 2008
1809	Invertebrates	Gammarus fossarum	1.8900E-02	10	2.4562E-03	Makarieva et al. 2008
1810	Invertebrates	Gammarus locusta	1.5000E-02	20	4.4331E-03	Makarieva et al. 2008
1811	Invertebrates	Gammarus locusta	1.5000E-02	20	5.0185E-03	Makarieva et al. 2008
1812	Invertebrates	Gammarus locusta	5.0000E-02	15	2.1600E-02	Makarieva et al. 2008
1813	Invertebrates	Gammarus oceanicus	5.0000E-02	15	1.2600E-02	Makarieva et al. 2008
1814	Invertebrates	Gammarus salinus	5.0000E-02	10	1.0800E-02	Makarieva et al. 2008
1815	Invertebrates	Gammarus zaddachi	5.0000E-02	10	9.9000E-03	Makarieva et al. 2008
1816	Invertebrates	Gaussia princeps	3.2800E-02	7	4.9594E-04	Makarieva et al. 2008
1817	Invertebrates	Gaussia princeps	3.2800E-02	7	2.5446E-03	Makarieva et al. 2008
1818	Invertebrates	Gaussia princeps	3.4600E-02	5	1.6068E-03	Makarieva et al. 2008
1819	Invertebrates	Gaussia princeps	3.5600E-02	5	1.5315E-03	Makarieva et al. 2008
1820	Invertebrates	Gecarcinus lateralis	1.0000E+01	20	5.5836E-01	Makarieva et al. 2008
1821	Invertebrates	Gecarcinus lateralis	2.0000E+01	20	4.4640E-01	Makarieva et al. 2008
1822	Invertebrates	Gecarcinus lateralis	4.0000E+01	20	5.0256E-01	Makarieva et al. 2008
1823	Invertebrates	Gecarcinus lateralis	5.3400E+01	20	4.0236E+00	Makarieva et al. 2008
1824	Invertebrates	Gecarcinus lateralis	1.4000E+02	20	5.6650E+00	Makarieva et al. 2008
1825	Invertebrates	Gecarcoidea natalis	1.6000E+02	25	2.0160E+01	Makarieva et al. 2008
1826	Invertebrates	Geholaspis longiponosus	1.6900E-04	10	4.4500E-05	Ehnes et al. 2011
1827	Invertebrates	Gennadas kempi /Petalidium foliaceum	5.5000E-01	0.2	2.6730E-02	Makarieva et al. 2008
1828	Invertebrates	Gennadas kempi /Petalidium foliaceum	9.6000E-01	0.2	4.4928E-02	Makarieva et al. 2008
1829	Invertebrates	Gennadas kempi /Petalidium foliaceum	1.6400E+00	0.3	6.1992E-02	Makarieva et al. 2008
1830	Invertebrates	Gennadas propinquus	1.6200E+00	5.5	3.1201E-02	Makarieva et al. 2008

1831	Invertebrates	Gennadas propinquus	1.6200E+00	5.5	7.8732E-02	Makarieva et al. 2008
1832	Invertebrates	Geolycosa domifex	4.0000E-01	22	4.5800E-02	Ehnes et al. 2011
1833	Invertebrates	Geolycosa domifex	4.0000E-01	15	1.3900E-02	Ehnes et al. 2011
1834	Invertebrates	Geolycosa domifex	4.0000E-01	18	3.1800E-02	Ehnes et al. 2011
1835	Invertebrates	Geophilidae	2.2350E-03	10.2	3.2790E-04	Ehnes et al. 2011
1836	Invertebrates	Geophilidae	3.9300E-03	15.6	1.4573E-03	Ehnes et al. 2011
1837	Invertebrates	Geophilidae	4.1625E-03	14.9	8.7500E-04	Ehnes et al. 2011
1838	Invertebrates	Geophilidae	5.6900E-03	14.7	1.3116E-03	Ehnes et al. 2011
1839	Invertebrates	Geophilidae	6.1800E-03	10.2	5.8290E-04	Ehnes et al. 2011
1840	Invertebrates	Geophilidae	7.3000E-03	10.3	4.3720E-04	Ehnes et al. 2011
1841	Invertebrates	Geophilidae	8.2233E-03	19.8	3.5000E-03	Ehnes et al. 2011
1842	Invertebrates	Geophilidae	8.5100E-03	23.4	4.9547E-03	Ehnes et al. 2011
1843	Invertebrates	Geophilidae	9.0100E-03	9.9	8.7435E-04	Ehnes et al. 2011
1844	Invertebrates	Geophilidae	9.1200E-03	14.9	2.5000E-03	Ehnes et al. 2011
1845	Invertebrates	Geophilidae	9.2000E-03	9.9	4.3720E-04	Ehnes et al. 2011
1846	Invertebrates	Geophilidae	9.3250E-03	30	7.5049E-03	Ehnes et al. 2011
1847	Invertebrates	Geophilidae	9.3300E-03	10.9	1.3000E-02	Ehnes et al. 2011
1848	Invertebrates	Geophilidae	9.4050E-03	19.8	3.7500E-03	Ehnes et al. 2011
1849	Invertebrates	Geophilidae	9.5300E-03	5	2.0402E-03	Ehnes et al. 2011
1850	Invertebrates	Geophilidae	9.6900E-03	15.6	3.6432E-03	Ehnes et al. 2011
1851	Invertebrates	Geophilidae	9.8800E-03	19.8	3.0000E-03	Ehnes et al. 2011
1852	Invertebrates	Geophilidae	1.0280E-02	5	1.4575E-04	Ehnes et al. 2011
1853	Invertebrates	Geophilidae	1.0320E-02	15.6	4.5175E-03	Ehnes et al. 2011
1854	Invertebrates	Geophilidae	1.0320E-02	23.4	7.7235E-03	Ehnes et al. 2011
1855	Invertebrates	Geophilidae	1.0570E-02	14.9	1.8945E-03	Ehnes et al. 2011
1856	Invertebrates	Geophilidae	1.0620E-02	29.9	6.8491E-03	Ehnes et al. 2011

1857	Invertebrates	Geophilidae	1.0670E-02	10.3	2.4774E-03	Ehnes et al. 2011
1858	Invertebrates	Geophilidae	1.1000E-02	14.7	2.1859E-03	Ehnes et al. 2011
1859	Invertebrates	Geophilidae	1.1070E-02	14.8	3.9346E-03	Ehnes et al. 2011
1860	Invertebrates	Geophilidae	1.1130E-02	14.7	2.1859E-03	Ehnes et al. 2011
1861	Invertebrates	Geophilidae	1.1270E-02	23.4	6.1205E-03	Ehnes et al. 2011
1862	Invertebrates	Geophilidae	1.1275E-02	29.9	7.7963E-03	Ehnes et al. 2011
1863	Invertebrates	Geophilidae	1.1395E-02	5	3.0603E-03	Ehnes et al. 2011
1864	Invertebrates	Geophilidae	1.1410E-02	14.9	3.0000E-03	Ehnes et al. 2011
1865	Invertebrates	Geophilidae	1.1430E-02	14.9	1.7487E-03	Ehnes et al. 2011
1866	Invertebrates	Geophilidae	1.1460E-02	15.6	4.5175E-03	Ehnes et al. 2011
1867	Invertebrates	Geophilidae	1.1550E-02	14.7	2.3316E-03	Ehnes et al. 2011
1868	Invertebrates	Geophilidae	1.1620E-02	14.7	2.3316E-03	Ehnes et al. 2011
1869	Invertebrates	Geophilidae	1.1700E-02	30	1.2970E-02	Ehnes et al. 2011
1870	Invertebrates	Geophilidae	1.1860E-02	23.4	1.0201E-03	Ehnes et al. 2011
1871	Invertebrates	Geophilidae	1.1960E-02	19.8	4.5000E-03	Ehnes et al. 2011
1872	Invertebrates	Geophilidae	1.1970E-02	5	5.8290E-04	Ehnes et al. 2011
1873	Invertebrates	Geophilidae	1.1980E-02	10.8	2.5000E-03	Ehnes et al. 2011
1874	Invertebrates	Geophilidae	1.3140E-02	23.4	6.5577E-03	Ehnes et al. 2011
1875	Invertebrates	Geophilidae	1.4050E-02	23.4	5.2461E-03	Ehnes et al. 2011
1876	Invertebrates	Geophilidae	1.4235E-02	29.9	8.1607E-03	Ehnes et al. 2011
1877	Invertebrates	Geophilidae	1.7510E-02	14.9	5.0000E-03	Ehnes et al. 2011
1878	Invertebrates	Geophilidae	1.8490E-02	15.6	5.9748E-03	Ehnes et al. 2011
1879	Invertebrates	Geophilidae	1.8540E-02	9.5	1.8945E-03	Ehnes et al. 2011
1880	Invertebrates	Geophilidae	1.9500E-02	23.4	6.8491E-03	Ehnes et al. 2011
1881	Invertebrates	Geophilidae	2.1130E-02	11	3.3517E-03	Ehnes et al. 2011
1882	Invertebrates	Geophilidae	2.2930E-02	30	1.4427E-02	Ehnes et al. 2011

1883	Invertebrates	Geophilidae	2.3900E-02	14.7	4.3718E-03	Ehnes et al. 2011
1884	Invertebrates	Geophilidae	2.3980E-02	19.9	9.1807E-03	Ehnes et al. 2011
1885	Invertebrates	Geophilidae	2.3990E-02	9.5	4.3720E-04	Ehnes et al. 2011
1886	Invertebrates	Geophilidae	2.4780E-02	5	2.9145E-04	Ehnes et al. 2011
1887	Invertebrates	Geophilidae	2.5520E-02	19.8	9.5000E-03	Ehnes et al. 2011
1888	Invertebrates	Geophilidae	3.2080E-02	5	1.0929E-02	Ehnes et al. 2011
1889	Invertebrates	Geophilidae	3.5800E-02	9.5	4.3718E-03	Ehnes et al. 2011
1890	Invertebrates	Geophilidae	3.5890E-02	9.7	2.1859E-03	Ehnes et al. 2011
1891	Invertebrates	Geophilidae	3.7440E-02	30	2.5648E-02	Ehnes et al. 2011
1892	Invertebrates	Geophilidae	3.7960E-02	19.9	1.2387E-02	Ehnes et al. 2011
1893	Invertebrates	Geophilidae	3.9320E-02	14.7	5.5376E-03	Ehnes et al. 2011
1894	Invertebrates	Geophilidae	3.9680E-02	9.5	4.5175E-03	Ehnes et al. 2011
1895	Invertebrates	Geophilidae	4.0200E-02	30	2.2879E-02	Ehnes et al. 2011
1896	Invertebrates	Geophilidae	4.0970E-02	10.8	1.5000E-03	Ehnes et al. 2011
1897	Invertebrates	Geophilidae	4.1090E-02	9.5	7.2863E-03	Ehnes et al. 2011
1898	Invertebrates	Geophilidae	4.1420E-02	5.8	2.9145E-04	Ehnes et al. 2011
1899	Invertebrates	Geophilidae	4.1770E-02	9.5	2.1859E-03	Ehnes et al. 2011
1900	Invertebrates	Geophilidae	4.2720E-02	9.5	1.0645E-03	Ehnes et al. 2011
1901	Invertebrates	Geophilidae	4.3770E-02	9.7	3.2060E-03	Ehnes et al. 2011
1902	Invertebrates	Geophilidae	4.3860E-02	20.2	1.1075E-02	Ehnes et al. 2011
1903	Invertebrates	Geophilidae	4.4480E-02	23.4	1.7924E-02	Ehnes et al. 2011
1904	Invertebrates	Geophilidae	4.4550E-02	19.9	1.2387E-02	Ehnes et al. 2011
1905	Invertebrates	Geophilidae	4.5080E-02	9.5	4.8575E-04	Ehnes et al. 2011
1906	Invertebrates	Geophilidae	4.5340E-02	9.5	4.0803E-03	Ehnes et al. 2011
1907	Invertebrates	Geophilidae	4.5430E-02	14.7	6.5577E-03	Ehnes et al. 2011
1908	Invertebrates	Geophilidae	4.5600E-02	9.9	1.8945E-03	Ehnes et al. 2011

1909	Invertebrates	Geophilidae	4.5910E-02	11	4.8090E-03	Ehnes et al. 2011
1910	Invertebrates	Geophilidae	4.5960E-02	29.9	2.4045E-02	Ehnes et al. 2011
1911	Invertebrates	Geophilidae	4.6760E-02	14.8	6.5577E-03	Ehnes et al. 2011
1912	Invertebrates	Geophilidae	4.7090E-02	9.5	3.9346E-03	Ehnes et al. 2011
1913	Invertebrates	Geophilidae	4.7220E-02	23.4	2.2296E-02	Ehnes et al. 2011
1914	Invertebrates	Geophilidae	4.8840E-02	9.5	3.6432E-03	Ehnes et al. 2011
1915	Invertebrates	Geophilidae	4.8980E-02	9.7	1.4573E-03	Ehnes et al. 2011
1916	Invertebrates	Geophilidae	4.9140E-02	9.5	5.1004E-03	Ehnes et al. 2011
1917	Invertebrates	Geophilidae	4.9690E-02	9.5	3.4974E-03	Ehnes et al. 2011
1918	Invertebrates	Geophilidae	5.0470E-02	9.5	3.2060E-03	Ehnes et al. 2011
1919	Invertebrates	Geophilidae	5.0480E-02	9.9	2.1859E-03	Ehnes et al. 2011
1920	Invertebrates	Geophilidae	5.0910E-02	5.8	9.1807E-03	Ehnes et al. 2011
1921	Invertebrates	Geophilidae	5.0940E-02	30	2.5502E-02	Ehnes et al. 2011
1922	Invertebrates	Geophilidae	5.1490E-02	9.5	3.6432E-03	Ehnes et al. 2011
1923	Invertebrates	Geophilidae	5.2130E-02	9.5	8.2135E-04	Ehnes et al. 2011
1924	Invertebrates	Geophilidae	5.2400E-02	9.9	2.9145E-03	Ehnes et al. 2011
1925	Invertebrates	Geophilidae	5.2510E-02	23.4	1.3407E-02	Ehnes et al. 2011
1926	Invertebrates	Geophilidae	5.3080E-02	30	3.5266E-02	Ehnes et al. 2011
1927	Invertebrates	Geophilidae	5.3770E-02	11	1.8945E-03	Ehnes et al. 2011
1928	Invertebrates	Geophilidae	5.3880E-02	9.5	2.7688E-03	Ehnes et al. 2011
1929	Invertebrates	Geophilidae	5.4170E-02	23.4	2.9291E-02	Ehnes et al. 2011
1930	Invertebrates	Geophilidae	5.4490E-02	11	6.1205E-03	Ehnes et al. 2011
1931	Invertebrates	Geophilidae	5.4500E-02	11	2.9145E-03	Ehnes et al. 2011
1932	Invertebrates	Geophilidae	5.5550E-02	5	1.6030E-03	Ehnes et al. 2011
1933	Invertebrates	Geophilidae	5.5620E-02	9.9	2.4774E-03	Ehnes et al. 2011
1934	Invertebrates	Geophilidae	5.6190E-02	9.5	6.9950E-04	Ehnes et al. 2011

1935	Invertebrates	Geophilidae	5.6880E-02	20.2	9.6179E-03	Ehnes et al. 2011
1936	Invertebrates	Geophilidae	5.7020E-02	9.5	1.2687E-03	Ehnes et al. 2011
1937	Invertebrates	Geophilidae	5.8190E-02	30	1.7924E-02	Ehnes et al. 2011
1938	Invertebrates	Geophilidae	5.8640E-02	23.4	1.4864E-02	Ehnes et al. 2011
1939	Invertebrates	Geophilidae	5.9460E-02	23.4	1.7196E-02	Ehnes et al. 2011
1940	Invertebrates	Geophilidae	5.9800E-02	9.5	4.2261E-03	Ehnes et al. 2011
1941	Invertebrates	Geophilidae	5.9960E-02	14.8	1.6904E-02	Ehnes et al. 2011
1942	Invertebrates	Geophilidae	6.0390E-02	14.9	8.1607E-03	Ehnes et al. 2011
1943	Invertebrates	Geophilidae	6.1190E-02	9.5	7.4945E-04	Ehnes et al. 2011
1944	Invertebrates	Geophilidae	6.2470E-02	5	2.1859E-03	Ehnes et al. 2011
1945	Invertebrates	Geophilidae	6.3640E-02	9.9	3.0603E-03	Ehnes et al. 2011
1946	Invertebrates	Geophilidae	6.4160E-02	9.5	5.8290E-03	Ehnes et al. 2011
1947	Invertebrates	Geophilidae	6.4630E-02	5	1.4573E-03	Ehnes et al. 2011
1948	Invertebrates	Geophilidae	6.4690E-02	9.5	1.2751E-03	Ehnes et al. 2011
1949	Invertebrates	Geophilidae	6.7760E-02	9.5	5.9748E-03	Ehnes et al. 2011
1950	Invertebrates	Geophilidae	7.0760E-02	30	2.6231E-02	Ehnes et al. 2011
1951	Invertebrates	Geophilidae	7.1940E-02	9.9	3.2060E-03	Ehnes et al. 2011
1952	Invertebrates	Geophilidae	7.4930E-02	9.9	2.1859E-03	Ehnes et al. 2011
1953	Invertebrates	Geophilidae	7.5460E-02	11	4.8090E-03	Ehnes et al. 2011
1954	Invertebrates	Geophilidae	7.5560E-02	30	2.6522E-02	Ehnes et al. 2011
1955	Invertebrates	Geophilidae	7.5890E-02	9.9	3.2060E-03	Ehnes et al. 2011
1956	Invertebrates	Geophilidae	7.6110E-02	11	4.3718E-03	Ehnes et al. 2011
1957	Invertebrates	Geophilidae	7.6190E-02	14.9	1.3115E-02	Ehnes et al. 2011
1958	Invertebrates	Geophilidae	7.8190E-02	14.8	1.0055E-02	Ehnes et al. 2011
1959	Invertebrates	Geophilidae	7.8420E-02	9.5	2.3316E-03	Ehnes et al. 2011
1960	Invertebrates	Geophilidae	7.8720E-02	9.5	2.7688E-03	Ehnes et al. 2011

1961	Invertebrates	Geophilidae	7.8940E-02	14.8	9.3265E-03	Ehnes et al. 2011
1962	Invertebrates	Geophilidae	8.0260E-02	14.8	8.5978E-03	Ehnes et al. 2011
1963	Invertebrates	Geophilidae	8.1440E-02	30	3.1331E-02	Ehnes et al. 2011
1964	Invertebrates	Geophilidae	8.5110E-02	14.8	1.2970E-02	Ehnes et al. 2011
1965	Invertebrates	Geophilidae	1.0388E-01	5	1.6030E-03	Ehnes et al. 2011
1966	Invertebrates	Geophilidae	1.0571E-01	11	6.2662E-03	Ehnes et al. 2011
1967	Invertebrates	Geophilidae	1.0803E-01	30	2.9874E-02	Ehnes et al. 2011
1968	Invertebrates	Geophilidae	1.0841E-01	9.9	1.7487E-03	Ehnes et al. 2011
1969	Invertebrates	Geophilidae	1.0953E-01	23.4	1.9382E-02	Ehnes et al. 2011
1970	Invertebrates	Geotrupes sp.	1.6030E-01	25	1.2236E-01	Ehnes et al. 2011
1971	Invertebrates	Geotrupes spiniger	8.2500E-01	25	8.9030E-01	Ehnes et al. 2011
1972	Invertebrates	Gigantocypris agassizii	5.7000E+00	4	3.3858E-03	Makarieva et al. 2008
1973	Invertebrates	Gigantocypris agassizii	5.7000E+00	4	1.2312E-02	Makarieva et al. 2008
1974	Invertebrates	Gigantocypris mulleri	7.4000E-01	-0.9	2.7972E-03	Makarieva et al. 2008
1975	Invertebrates	Gigantocypris mulleri	8.1000E-01	0.2	5.1030E-03	Makarieva et al. 2008
1976	Invertebrates	Gigantocypris mulleri	1.8400E+00	-1	4.9680E-03	Makarieva et al. 2008
1977	Invertebrates	Gigantocypris mulleri	1.8800E+00	0.2	8.1216E-03	Makarieva et al. 2008
1978	Invertebrates	Globitermes globosus	1.9000E-03	25	2.5200E-04	Ehnes et al. 2011
1979	Invertebrates	Glomeris	1.6990E-02	29.9	6.9948E-03	Ehnes et al. 2011
1980	Invertebrates	Glomeris	1.7380E-02	30	6.7034E-03	Ehnes et al. 2011
1981	Invertebrates	Glomeris	2.1720E-02	16	8.0149E-03	Ehnes et al. 2011
1982	Invertebrates	Glomeris	2.2130E-02	9.9	5.8290E-04	Ehnes et al. 2011
1983	Invertebrates	Glomeris	2.3170E-02	29.9	8.5978E-03	Ehnes et al. 2011
1984	Invertebrates	Glomeris	2.7070E-02	29.9	8.4521E-03	Ehnes et al. 2011
1985	Invertebrates	Glomeris	2.8060E-02	9.2	1.0201E-03	Ehnes et al. 2011
1986	Invertebrates	Glomeris	3.8180E-02	30	8.7436E-03	Ehnes et al. 2011

1987	Invertebrates	Glomeris	4.0320E-02	5	4.3720E-04	Ehnes et al. 2011
1988	Invertebrates	Glomeris	4.8160E-02	14.6	3.4974E-03	Ehnes et al. 2011
1989	Invertebrates	Glomeris	4.9470E-02	14.6	2.1859E-03	Ehnes et al. 2011
1990	Invertebrates	Glomeris	5.6960E-02	29.9	3.4313E-02	Ehnes et al. 2011
1991	Invertebrates	Glomeris	6.4810E-02	9.2	6.2662E-03	Ehnes et al. 2011
1992	Invertebrates	Glomeris	6.5270E-02	30	1.6321E-02	Ehnes et al. 2011
1993	Invertebrates	Glomeris	6.5420E-02	30	1.1804E-02	Ehnes et al. 2011
1994	Invertebrates	Glomeris	6.6380E-02	24.9	1.1367E-02	Ehnes et al. 2011
1995	Invertebrates	Glomeris	6.6640E-02	29.9	3.4708E-02	Ehnes et al. 2011
1996	Invertebrates	Glomeris	7.3180E-02	14.6	1.2095E-02	Ehnes et al. 2011
1997	Invertebrates	Glomeris	7.3630E-02	30	1.2678E-02	Ehnes et al. 2011
1998	Invertebrates	Glomeris	9.7090E-02	14.2	1.0638E-02	Ehnes et al. 2011
1999	Invertebrates	Glomeris	1.0487E-01	9.9	1.4573E-03	Ehnes et al. 2011
2000	Invertebrates	Glomeris	1.1067E-01	30	2.2150E-02	Ehnes et al. 2011
2001	Invertebrates	Glomeris	1.1475E-01	9.9	1.8945E-03	Ehnes et al. 2011
2002	Invertebrates	Glomeris	1.2051E-01	9.9	1.7487E-03	Ehnes et al. 2011
2003	Invertebrates	Glomeris	1.3316E-01	9.7	5.6833E-03	Ehnes et al. 2011
2004	Invertebrates	Glomeris	1.3326E-01	14.6	8.0149E-03	Ehnes et al. 2011
2005	Invertebrates	Glomeris	1.3379E-01	14.6	8.4521E-03	Ehnes et al. 2011
2006	Invertebrates	Glomeris	1.3387E-01	9.9	1.8945E-03	Ehnes et al. 2011
2007	Invertebrates	Glomeris	1.3405E-01	5	2.3316E-03	Ehnes et al. 2011
2008	Invertebrates	Glomeris	1.3530E-01	14.2	6.2662E-03	Ehnes et al. 2011
2009	Invertebrates	Glomeris	1.3658E-01	5	2.6231E-03	Ehnes et al. 2011
2010	Invertebrates	Glomeris	1.4170E-01	30	2.2733E-02	Ehnes et al. 2011
2011	Invertebrates	Glomeris	1.4183E-01	16	2.0402E-03	Ehnes et al. 2011
2012	Invertebrates	Glomeris	1.4384E-01	9.9	2.1859E-03	Ehnes et al. 2011

2013	Invertebrates	Glomeris	1.5351E-01	5	1.6030E-03	Ehnes et al. 2011
2014	Invertebrates	Glomeris	1.5440E-01	9.7	1.6030E-03	Ehnes et al. 2011
2015	Invertebrates	Glomeris	1.5641E-01	16	1.1512E-02	Ehnes et al. 2011
2016	Invertebrates	Glomeris	1.5935E-01	30	9.3265E-03	Ehnes et al. 2011
2017	Invertebrates	Glomeris	1.7002E-01	14.2	4.2261E-03	Ehnes et al. 2011
2018	Invertebrates	Glomeris	1.7161E-01	30	2.0693E-02	Ehnes et al. 2011
2019	Invertebrates	Glomeris	1.7220E-01	9.7	2.3316E-03	Ehnes et al. 2011
2020	Invertebrates	Glomeris	1.7326E-01	9.7	3.9346E-03	Ehnes et al. 2011
2021	Invertebrates	Glomeris	1.7392E-01	9.9	2.7688E-03	Ehnes et al. 2011
2022	Invertebrates	Glomeris	1.7846E-01	29.9	5.1578E-02	Ehnes et al. 2011
2023	Invertebrates	Glomeris	1.8314E-01	5	3.4974E-03	Ehnes et al. 2011
2024	Invertebrates	Glomeris	1.8599E-01	9.7	2.9145E-03	Ehnes et al. 2011
2025	Invertebrates	Glomeris	1.9438E-01	16	2.4774E-03	Ehnes et al. 2011
2026	Invertebrates	Glomeris	2.0931E-01	5	1.3116E-03	Ehnes et al. 2011
2027	Invertebrates	Glomeris	2.0996E-01	30	2.0110E-02	Ehnes et al. 2011
2028	Invertebrates	Glomeris	2.1952E-01	14.6	1.1804E-02	Ehnes et al. 2011
2029	Invertebrates	Glomeris	2.5347E-01	30	2.1567E-02	Ehnes et al. 2011
2030	Invertebrates	Glomeris	2.9262E-01	30	3.6431E-02	Ehnes et al. 2011
2031	Invertebrates	Glomeris	2.9324E-01	14.6	1.1367E-02	Ehnes et al. 2011
2032	Invertebrates	Glossina morsitans	2.2340E-02	25	2.2230E-02	Ehnes et al. 2011
2033	Invertebrates	Glossina pallidipes	3.8450E-02	25	6.7374E-02	Ehnes et al. 2011
2034	Invertebrates	Glossoscolex paulistus	9.5000E+00	20	4.4350E-01	Ehnes et al. 2011
2035	Invertebrates	Glossoscolex paulistus	1.0000E+01	25	5.2500E-01	Ehnes et al. 2011
2036	Invertebrates	Glossoscolex paulistus	1.0200E+01	15	4.3250E-01	Ehnes et al. 2011
2037	Invertebrates	Glossoscolex paulistus	1.0400E+01	30	7.1500E-01	Ehnes et al. 2011
2038	Invertebrates	Glossoscolex paulistus	1.0500E+01	35	7.8500E-01	Ehnes et al. 2011

2039	Invertebrates	Glyphocrangon vicaria	9.7100E+00	2	2.0974E-01	Makarieva et al. 2008
2040	Invertebrates	Glyphocrangon vicaria	9.8500E+00	11	3.5460E-01	Makarieva et al. 2008
2041	Invertebrates	Glyptonotus antarcticus	3.3000E+01	0	3.3858E-01	Makarieva et al. 2008
2042	Invertebrates	Gnathophausia gigas	7.7000E-01	0.2	1.9404E-02	Makarieva et al. 2008
2043	Invertebrates	Gnathophausia gigas	1.0100E+00	5	1.5635E-02	Makarieva et al. 2008
2044	Invertebrates	Gnathophausia gigas	1.0520E+00	4	1.9693E-02	Makarieva et al. 2008
2045	Invertebrates	Gnathophausia gigas	1.0520E+00	4	2.1398E-01	Makarieva et al. 2008
2046	Invertebrates	Gnathophausia gracilis	2.0500E+00	4	2.2878E-02	Makarieva et al. 2008
2047	Invertebrates	Gnathophausia gracilis	2.0500E+00	4	1.5572E-01	Makarieva et al. 2008
2048	Invertebrates	Gnathophausia gracilis	2.4500E+00	5	4.9833E-02	Makarieva et al. 2008
2049	Invertebrates	Gnathophausia ingens	5.1100E+00	10	1.7476E-01	Makarieva et al. 2008
2050	Invertebrates	Gnathophausia ingens	5.1100E+00	5	2.4835E-01	Makarieva et al. 2008
2051	Invertebrates	Gnathophausia ingens	5.5420E+00	5.5	9.2773E-02	Makarieva et al. 2008
2052	Invertebrates	Gnathophausia ingens	5.5420E+00	5.5	8.3695E-01	Makarieva et al. 2008
2053	Invertebrates	Gnathophausia ingens	5.2420E+01	4	1.7833E-01	Makarieva et al. 2008
2054	Invertebrates	Gnathophausia zoea	3.9900E+00	10	7.9002E-02	Makarieva et al. 2008
2055	Invertebrates	Gnathophausia zoea	3.9900E+00	5	1.4364E-01	Makarieva et al. 2008
2056	Invertebrates	Gnathophausia zoea	5.2000E+00	5.5	8.7048E-02	Makarieva et al. 2008
2057	Invertebrates	Gnathophausia zoea	5.2000E+00	5.5	1.1326E-01	Makarieva et al. 2008
2058	Invertebrates	Gomphiocephalus hodgsoni	1.4600E-05	10	2.5000E-06	Ehnes et al. 2011
2059	Invertebrates	Gomphiocephalus hodgsoni	1.8600E-05	10	1.3450E-05	Ehnes et al. 2011
2060	Invertebrates	Gomphiocephalus hodgsoni	2.1000E-05	10	1.4000E-06	Ehnes et al. 2011
2061	Invertebrates	Gomphiocephalus hodgsoni	2.4000E-05	10	2.7000E-06	Ehnes et al. 2011
2062	Invertebrates	Gomphiocephalus hodgsoni	2.8000E-05	10	1.6700E-05	Ehnes et al. 2011
2063	Invertebrates	Gomphiocephalus hodgsoni	3.0900E-05	10	1.0550E-05	Ehnes et al. 2011
2064	Invertebrates	Gomphiocephalus hodgsoni	3.4300E-05	10	9.8500E-06	Ehnes et al. 2011

2065	Invertebrates	Gonatus onyx	1.1700E-01	5	6.5791E-03	Makarieva et al. 2008
2066	Invertebrates	Gonatus pyros	3.8400E+00	5	2.7109E-01	Makarieva et al. 2008
2067	Invertebrates	Gondogeneia antarctica	2.9000E-02	1	1.0440E-02	Makarieva et al. 2008
2068	Invertebrates	Griburius sp.1	3.3100E-03	25	1.0818E-02	Ehnes et al. 2011
2069	Invertebrates	Griburius sp.2	9.4200E-03	25	7.8660E-03	Ehnes et al. 2011
2070	Invertebrates	Gromphadorhina chopardi	3.4000E+00	25	4.8332E-01	Ehnes et al. 2011
2071	Invertebrates	Gromphadorihna portentosa	4.9500E+00	25	8.5446E-01	Ehnes et al. 2011
2072	Invertebrates	Gryllotalpa australis	8.7400E-01	25	4.0241E-01	Ehnes et al. 2011
2073	Invertebrates	Gryllus domesticus	1.8000E-02	19.8	8.2871E-03	Ehnes et al. 2011
2074	Invertebrates	Gryllus domesticus	3.1000E-02	19.9	1.5029E-02	Ehnes et al. 2011
2075	Invertebrates	Gryllus domesticus	3.3000E-02	20.2	1.5872E-02	Ehnes et al. 2011
2076	Invertebrates	Gryllus domesticus	3.5000E-02	19.8	1.9945E-02	Ehnes et al. 2011
2077	Invertebrates	Gryllus domesticus	4.9000E-02	20	2.1350E-02	Ehnes et al. 2011
2078	Invertebrates	Gryllus domesticus	4.9000E-02	19.9	2.0788E-02	Ehnes et al. 2011
2079	Invertebrates	Gryllus domesticus	5.3000E-02	20.1	2.3457E-02	Ehnes et al. 2011
2080	Invertebrates	Gryllus domesticus	6.1000E-02	20.9	1.7838E-02	Ehnes et al. 2011
2081	Invertebrates	Gryllus domesticus	6.8000E-02	20	2.7951E-02	Ehnes et al. 2011
2082	Invertebrates	Gryllus domesticus	8.0000E-02	20.2	2.1350E-02	Ehnes et al. 2011
2083	Invertebrates	Gryllus domesticus	1.2500E-01	20	3.6800E-02	Ehnes et al. 2011
2084	Invertebrates	Gryllus domesticus	1.3200E-01	20.2	3.2446E-02	Ehnes et al. 2011
2085	Invertebrates	Gryllus domesticus	1.4300E-01	20.1	4.9301E-02	Ehnes et al. 2011
2086	Invertebrates	Gryllus domesticus	2.0300E-01	20.4	3.9188E-02	Ehnes et al. 2011
2087	Invertebrates	Gryllus domesticus	2.6700E-01	20	7.6831E-02	Ehnes et al. 2011
2088	Invertebrates	Gryllus domesticus	2.6700E-01	20.4	1.0113E-01	Ehnes et al. 2011
2089	Invertebrates	Gryllus domesticus	2.8900E-01	20	1.0759E-01	Ehnes et al. 2011
2090	Invertebrates	Gryllus domesticus	3.0000E-01	19.8	8.1606E-02	Ehnes et al. 2011

2091	Invertebrates	Gryllus domesticus	3.2400E-01	20	1.0871E-01	Ehnes et al. 2011
2092	Invertebrates	Gryllus domesticus	3.3000E-01	20	1.0801E-01	Ehnes et al. 2011
2093	Invertebrates	Gryllus domesticus	3.3000E-01	20.1	7.4724E-02	Ehnes et al. 2011
2094	Invertebrates	Gryllus domesticus	3.5700E-01	20	9.6776E-02	Ehnes et al. 2011
2095	Invertebrates	Gryllus domesticus	3.6600E-01	20.2	1.2304E-01	Ehnes et al. 2011
2096	Invertebrates	Gryllus domesticus	3.9000E-01	19.8	1.4411E-01	Ehnes et al. 2011
2097	Invertebrates	Gryllus domesticus	4.0400E-01	20.5	1.2192E-01	Ehnes et al. 2011
2098	Invertebrates	Gryllus domesticus	4.2800E-01	20.3	9.4809E-02	Ehnes et al. 2011
2099	Invertebrates	Gryllus domesticus	4.3000E-01	20	9.4809E-02	Ehnes et al. 2011
2100	Invertebrates	Gryllus domesticus	4.8000E-01	19.5	1.8386E-01	Ehnes et al. 2011
2101	Invertebrates	Gryllus domesticus	5.0200E-01	20	1.6560E-01	Ehnes et al. 2011
2102	Invertebrates	Gryllus domesticus	5.1300E-01	20	1.4200E-01	Ehnes et al. 2011
2103	Invertebrates	Gryllus domesticus	5.4300E-01	20.1	1.1279E-01	Ehnes et al. 2011
2104	Invertebrates	Haliscera bigelowi	4.7000E-01	5	1.3536E-03	Makarieva et al. 2008
2105	Invertebrates	Halitrehees maasi	1.9000E+01	5	1.9494E-02	Makarieva et al. 2008
2106	Invertebrates	Haloptilus longicornis	1.6000E-04	20	6.6067E-05	Makarieva et al. 2008
2107	Invertebrates	Haloptilus longicornis	2.2000E-04	20	4.7758E-05	Makarieva et al. 2008
2108	Invertebrates	Hapigia simplex	4.7800E-01	25	2.8512E-01	Ehnes et al. 2011
2109	Invertebrates	Harpalus	3.3600E-02	8	6.2662E-03	Ehnes et al. 2011
2110	Invertebrates	Harpalus	3.5400E-02	8	3.3517E-03	Ehnes et al. 2011
2111	Invertebrates	Harpalus	3.7200E-02	18.5	1.1221E-02	Ehnes et al. 2011
2112	Invertebrates	Harpalus	3.7900E-02	11.5	4.8090E-03	Ehnes et al. 2011
2113	Invertebrates	Harpalus	3.9600E-02	11.5	7.8692E-03	Ehnes et al. 2011
2114	Invertebrates	Harpalus	3.9700E-02	11.5	6.8491E-03	Ehnes et al. 2011
2115	Invertebrates	Harpalus	4.0100E-02	8	4.9547E-03	Ehnes et al. 2011
2116	Invertebrates	Harpalus	4.3800E-02	18.5	1.8653E-02	Ehnes et al. 2011

2117	Invertebrates	Harpalus	4.4400E-02	8	3.3517E-03	Ehnes et al. 2011
2118	Invertebrates	Harpalus	4.4700E-02	11.5	8.4521E-03	Ehnes et al. 2011
2119	Invertebrates	Harpalus	4.5200E-02	15	1.9090E-02	Ehnes et al. 2011
2120	Invertebrates	Harpalus	4.6900E-02	11.5	8.4521E-03	Ehnes et al. 2011
2121	Invertebrates	Harpalus	4.7100E-02	15	6.7034E-03	Ehnes et al. 2011
2122	Invertebrates	Harpalus	4.8200E-02	8	3.2060E-03	Ehnes et al. 2011
2123	Invertebrates	Harpalus	4.8300E-02	15	7.1406E-03	Ehnes et al. 2011
2124	Invertebrates	Harpalus	5.0100E-02	18.5	2.1713E-02	Ehnes et al. 2011
2125	Invertebrates	Harpalus	5.0700E-02	22	1.6321E-02	Ehnes et al. 2011
2126	Invertebrates	Harpalus	5.0900E-02	18.5	1.3261E-02	Ehnes et al. 2011
2127	Invertebrates	Harpalus	5.1100E-02	22	1.9236E-02	Ehnes et al. 2011
2128	Invertebrates	Harpalus	5.1700E-02	15	8.0149E-03	Ehnes et al. 2011
2129	Invertebrates	Harpalus	5.2100E-02	11.5	1.1804E-02	Ehnes et al. 2011
2130	Invertebrates	Harpalus	5.2600E-02	22	1.7196E-02	Ehnes et al. 2011
2131	Invertebrates	Harpalus	5.3000E-02	15	9.3265E-03	Ehnes et al. 2011
2132	Invertebrates	Harpalus	5.3000E-02	15	1.7341E-02	Ehnes et al. 2011
2133	Invertebrates	Harpalus	5.3100E-02	18.5	1.3115E-02	Ehnes et al. 2011
2134	Invertebrates	Harpalus	5.3400E-02	22	3.7306E-02	Ehnes et al. 2011
2135	Invertebrates	Harpalus	5.3400E-02	22	1.9673E-02	Ehnes et al. 2011
2136	Invertebrates	Harpalus	5.3600E-02	18.5	1.7341E-02	Ehnes et al. 2011
2137	Invertebrates	Harpalus	5.3900E-02	15	8.8893E-03	Ehnes et al. 2011
2138	Invertebrates	Harpalus	5.4800E-02	15	5.6833E-03	Ehnes et al. 2011
2139	Invertebrates	Harpalus	5.4800E-02	18.5	3.3954E-02	Ehnes et al. 2011
2140	Invertebrates	Harpalus	5.5700E-02	22	1.9090E-02	Ehnes et al. 2011
2141	Invertebrates	Harpalus	5.7100E-02	15	7.7235E-03	Ehnes et al. 2011
2142	Invertebrates	Harpalus	5.9000E-02	11.5	2.2442E-02	Ehnes et al. 2011

2143	Invertebrates	Harpalus	6.2000E-02	18.5	2.4045E-02	Ehnes et al. 2011
2144	Invertebrates	Harpalus	6.3700E-02	11.5	7.8692E-03	Ehnes et al. 2011
2145	Invertebrates	Harpalus	9.4620E-02	5	6.5000E-03	Ehnes et al. 2011
2146	Invertebrates	Harpalus	1.0260E-01	5	5.5000E-03	Ehnes et al. 2011
2147	Invertebrates	Havilanditermes atripennis	8.0000E-03	25	1.4400E-03	Ehnes et al. 2011
2148	Invertebrates	Helicocranchia pfeferi	3.6000E+00	5	3.3048E-02	Makarieva et al. 2008
2149	Invertebrates	Helius waiti	6.7200E-01	25	3.0152E-01	Ehnes et al. 2011
2150	Invertebrates	Hemigrapsus nudus	2.0000E+01	20	2.7000E-02	Makarieva et al. 2008
2151	Invertebrates	Hemigrapsus nudus	2.0000E+01	20	3.8520E-02	Makarieva et al. 2008
2152	Invertebrates	Hemigrapsus oregonensis	2.0000E+01	20	3.8160E-02	Makarieva et al. 2008
2153	Invertebrates	Hemigrapsus oregonensis	2.0000E+01	20	5.0760E-02	Makarieva et al. 2008
2154	Invertebrates	Hemihenlea	2.6000E-05	20	1.3950E-05	Ehnes et al. 2011
2155	Invertebrates	Hemihenlea	6.4000E-05	20	2.7200E-05	Ehnes et al. 2011
2156	Invertebrates	Hemihenlea	1.6000E-04	20	3.3700E-05	Ehnes et al. 2011
2157	Invertebrates	Hemihenlea	2.3700E-04	20	2.2450E-05	Ehnes et al. 2011
2158	Invertebrates	Hemileius initialis	2.1000E-05	5	2.8000E-06	Ehnes et al. 2011
2159	Invertebrates	Hemileius initialis	2.1000E-05	10	4.9500E-06	Ehnes et al. 2011
2160	Invertebrates	Hemileius initialis	2.1000E-05	15	8.0000E-06	Ehnes et al. 2011
2161	Invertebrates	Hemileius initialis	2.1000E-05	20	1.2250E-05	Ehnes et al. 2011
2162	Invertebrates	Hemilepistus elegans	1.1500E-01	20	2.2418E-02	Makarieva et al. 2008
2163	Invertebrates	Hemilepistus elegans	2.7500E-01	20	2.5839E-02	Makarieva et al. 2008
2164	Invertebrates	Hemirhabdus grimaldii	3.8200E-02	5	4.3319E-04	Makarieva et al. 2008
2165	Invertebrates	Hentzia palmarum	2.3500E-02	20	5.2000E-03	Ehnes et al. 2011
2166	Invertebrates	Hermannia gibba	1.7600E-05	15	1.2300E-05	Ehnes et al. 2011
2167	Invertebrates	Hermannia gibba	4.4000E-05	15	1.7600E-05	Ehnes et al. 2011
2168	Invertebrates	Hermannia gibba	1.0050E-04	15	2.4050E-05	Ehnes et al. 2011

2169	Invertebrates	Hermannia gibba	1.7200E-04	15	3.1650E-05	Ehnes et al. 2011
2170	Invertebrates	Hermannia gibba	2.0700E-04	15	4.0250E-05	Ehnes et al. 2011
2171	Invertebrates	Heterokrohnia murina	2.0400E-01	5	2.1665E-03	Makarieva et al. 2008
2172	Invertebrates	Heterorhabdus farrani	3.2150E-03	0	2.2569E-04	Makarieva et al. 2008
2173	Invertebrates	Heterotermes tenuior	1.4000E-03	25	3.4200E-04	Ehnes et al. 2011
2174	Invertebrates	Hippodamia convergens	1.7000E-02	25	3.4776E-02	Ehnes et al. 2011
2175	Invertebrates	Hipporhinus tenuegranosus	1.0800E+00	25	9.8640E-02	Ehnes et al. 2011
2176	Invertebrates	Histioteuthis heteropsis	3.6980E+01	5	3.7276E-01	Makarieva et al. 2008
2177	Invertebrates	Histioteuthis hoylei	8.5100E+00	5	2.1598E-01	Makarieva et al. 2008
2178	Invertebrates	Hogna carolinensis	1.0000E+00	40	2.3250E-01	Ehnes et al. 2011
2179	Invertebrates	Hogna lenta	4.9800E-01	20	5.3500E-02	Ehnes et al. 2011
2180	Invertebrates	Hogna lenta	9.7000E-01	10	2.7450E-02	Ehnes et al. 2011
2181	Invertebrates	Hogna lenta	9.7000E-01	30	1.9500E-01	Ehnes et al. 2011
2182	Invertebrates	Hogna lenta	9.7000E-01	20	9.8000E-02	Ehnes et al. 2011
2183	Invertebrates	Holoparasitus inornatus	6.6000E-05	10	2.0300E-05	Ehnes et al. 2011
2184	Invertebrates	Holthuisana transversa	2.0000E+00	20	1.9289E-01	Makarieva et al. 2008
2185	Invertebrates	Holthuisana transversa	5.0000E+00	20	2.7567E-01	Makarieva et al. 2008
2186	Invertebrates	Holthuisana transversa	1.0000E+01	20	3.8376E-01	Makarieva et al. 2008
2187	Invertebrates	Holthuisana transversa	1.2000E+01	20	3.9290E-01	Makarieva et al. 2008
2188	Invertebrates	Holthuisana transversa	1.5000E+01	20	4.2930E-01	Makarieva et al. 2008
2189	Invertebrates	Holthuisana transversa	2.0000E+01	20	4.8888E-01	Makarieva et al. 2008
2190	Invertebrates	Holthuisana transversa	3.0000E+01	20	5.6538E-01	Makarieva et al. 2008
2191	Invertebrates	Homallotermes eleanorae	2.2000E-03	25	2.7000E-04	Ehnes et al. 2011
2192	Invertebrates	Homarus americanus	1.8000E+02	20	7.9121E+00	Makarieva et al. 2008
2193	Invertebrates	Hophlosphyrum griseus	3.6200E-02	25	1.4382E-02	Ehnes et al. 2011
2194	Invertebrates	Hospitalitermes hospitalis	7.7000E-03	25	1.0080E-03	Ehnes et al. 2011

2195	Invertebrates	Hydromedion sparsutum	2.3500E-02	25	1.5030E-02	Ehnes et al. 2011
2196	Invertebrates	Hygrolycosa rubrofasciata	1.8600E-02	25	1.4550E-02	Ehnes et al. 2011
2197	Invertebrates	Hygrolycosa rubrofasciata	1.9900E-02	25	7.9500E-03	Ehnes et al. 2011
2198	Invertebrates	Hygrolycosa rubrofasciata	2.1200E-02	25	5.7500E-03	Ehnes et al. 2011
2199	Invertebrates	Hylesia praeda	1.4600E-01	25	1.4692E-01	Ehnes et al. 2011
2200	Invertebrates	Hylesia sp.	2.3914E-01	25	1.2281E-01	Ehnes et al. 2011
2201	Invertebrates	Hylobius abietis	1.8300E-01	25	7.5258E-02	Ehnes et al. 2011
2202	Invertebrates	Hymenodora frontalis	1.4000E+00	5.5	2.2932E-02	Makarieva et al. 2008
2203	Invertebrates	Hymenodora frontalis	1.4000E+00	5.5	4.5612E-02	Makarieva et al. 2008
2204	Invertebrates	Hypera postica	5.8000E-03	25	2.8710E-02	Ehnes et al. 2011
2205	Invertebrates	Hyperchirica nausica	1.9950E-01	25	1.4148E-01	Ehnes et al. 2011
2206	Invertebrates	Hypererythrops sp.	1.1100E-03	17	8.7133E-04	Makarieva et al. 2008
2207	Invertebrates	Hyperia galba	1.0150E-02	5.6	4.7502E-04	Makarieva et al. 2008
2208	Invertebrates	Hyperia galba	3.5200E-02	5.6	2.1542E-03	Makarieva et al. 2008
2209	Invertebrates	Hyperia galba	3.5700E-02	10	2.7311E-03	Makarieva et al. 2008
2210	Invertebrates	Hyperia galba	3.5700E-02	10	5.1022E-03	Makarieva et al. 2008
2211	Invertebrates	Hyperia galba	4.6200E-02	5.6	2.9106E-03	Makarieva et al. 2008
2212	Invertebrates	Hyperia galba	4.9700E-02	12.2	7.6041E-03	Makarieva et al. 2008
2213	Invertebrates	Hyperia galba	8.2400E-02	5.6	3.4114E-03	Makarieva et al. 2008
2214	Invertebrates	Hyperia galba	2.5270E-01	8	1.7421E-02	Makarieva et al. 2008
2215	Invertebrates	Hyperia gaudichaudii	5.2500E-01	-0.8	2.2680E-02	Makarieva et al. 2008
2216	Invertebrates	Hyperia sp.	4.2800E-04	17	3.0046E-04	Makarieva et al. 2008
2217	Invertebrates	Hyperia sp.	1.3100E-03	17	7.1919E-04	Makarieva et al. 2008
2218	Invertebrates	Hyperia sp.	9.2700E-03	6.7	1.3632E-03	Makarieva et al. 2008
2219	Invertebrates	Hyperiella antarctica	2.0000E-02	20	3.1810E-03	Makarieva et al. 2008
2220	Invertebrates	Hypochthonius rufulus	2.2100E-05	0	1.0500E-06	Ehnes et al. 2011

2221	Invertebrates	Hypochthonius rufulus	2.2100E-05	5	1.7500E-06	Ehnes et al. 2011
2222	Invertebrates	Hypochthonius rufulus	2.2100E-05	10	4.2000E-06	Ehnes et al. 2011
2223	Invertebrates	Hypochthonius rufulus	2.2100E-05	15	7.8000E-06	Ehnes et al. 2011
2224	Invertebrates	Hypotermes xenotermites	2.6000E-03	25	5.2200E-04	Ehnes et al. 2011
2225	Invertebrates	Hysteropterum sp.	1.7100E-03	25	7.2540E-03	Ehnes et al. 2011
2226	Invertebrates	Idotea baltica basteri	1.3850E-01	20	3.6149E-02	Makarieva et al. 2008
2227	Invertebrates	Illex illecebrosus	4.4300E+02	5	3.9695E+01	Makarieva et al. 2008
2228	Invertebrates	Insara covilleae	9.2600E-02	25	2.9396E-01	Ehnes et al. 2011
2229	Invertebrates	Iphinoe sp.	6.2000E-04	30	3.4719E-04	Makarieva et al. 2008
2230	Invertebrates	Iphinoe sp.	6.6400E-03	29	1.1952E-03	Makarieva et al. 2008
2231	Invertebrates	Ips acuminatus	2.8400E-03	25	6.5340E-03	Ehnes et al. 2011
2232	Invertebrates	Isopoda	1.6792E-03	5	5.8290E-04	Ehnes et al. 2011
2233	Invertebrates	Isopoda	1.6971E-03	23.4	1.2699E-03	Ehnes et al. 2011
2234	Invertebrates	Isopoda	2.0925E-03	30	2.0766E-03	Ehnes et al. 2011
2235	Invertebrates	Isopoda	2.4550E-03	30	1.8580E-03	Ehnes et al. 2011
2236	Invertebrates	Isopoda	2.5350E-03	30	2.0038E-03	Ehnes et al. 2011
2237	Invertebrates	Isopoda	2.6325E-03	30	2.7688E-03	Ehnes et al. 2011
2238	Invertebrates	Isopoda	2.7200E-03	29.9	1.5301E-03	Ehnes et al. 2011
2239	Invertebrates	Isopoda	3.4733E-03	30	2.8174E-03	Ehnes et al. 2011
2240	Invertebrates	Isopoda	3.4967E-03	29.9	1.6030E-03	Ehnes et al. 2011
2241	Invertebrates	Isopoda	3.5133E-03	30	3.1088E-03	Ehnes et al. 2011
2242	Invertebrates	Isopoda	3.5967E-03	23.4	2.0888E-03	Ehnes et al. 2011
2243	Invertebrates	Isopoda	4.1267E-03	30	4.7604E-03	Ehnes et al. 2011
2244	Invertebrates	Isopoda	4.4167E-03	30	3.2546E-03	Ehnes et al. 2011
2245	Invertebrates	Isopoda	4.6100E-03	30	2.5745E-03	Ehnes et al. 2011
2246	Invertebrates	Isopoda	5.2850E-03	30	3.4974E-03	Ehnes et al. 2011

2247	Invertebrates	Isopoda	5.4367E-03	23.4	3.3031E-03	Ehnes et al. 2011
2248	Invertebrates	Isopoda	5.4600E-03	30	5.1733E-03	Ehnes et al. 2011
2249	Invertebrates	Isopoda	5.6850E-03	30	5.2461E-03	Ehnes et al. 2011
2250	Invertebrates	Isopoda	5.8300E-03	29.9	4.2746E-03	Ehnes et al. 2011
2251	Invertebrates	Isopoda	5.9600E-03	30	4.5175E-03	Ehnes et al. 2011
2252	Invertebrates	Isopoda	5.9800E-03	30	5.4890E-03	Ehnes et al. 2011
2253	Invertebrates	Isopoda	2.1460E-02	15	1.8945E-03	Ehnes et al. 2011
2254	Invertebrates	Isopoda	2.8020E-02	15	4.6632E-03	Ehnes et al. 2011
2255	Invertebrates	Isopoda	3.1780E-02	15	3.3517E-03	Ehnes et al. 2011
2256	Invertebrates	Isotoma viridis	2.8000E-04	8	4.0550E-05	Ehnes et al. 2011
2257	Invertebrates	Isotoma viridis	4.0000E-04	18	2.1800E-04	Ehnes et al. 2011
2258	Invertebrates	Isotoma viridis	7.2000E-04	18	3.9550E-04	Ehnes et al. 2011
2259	Invertebrates	Isotoma viridis	7.7000E-04	18	4.2100E-04	Ehnes et al. 2011
2260	Invertebrates	Isotomiella minor	1.0000E-05	6	2.2500E-06	Ehnes et al. 2011
2261	Invertebrates	Isotomiella minor	1.0000E-05	10	2.6500E-06	Ehnes et al. 2011
2262	Invertebrates	Isotomiella minor	1.0000E-05	15	4.6000E-06	Ehnes et al. 2011
2263	Invertebrates	Janicella spinicauda	3.5000E-01	10	3.9690E-02	Makarieva et al. 2008
2264	Invertebrates	Janicella spinicauda	3.5000E-01	20	1.2096E-01	Makarieva et al. 2008
2265	Invertebrates	Japetella diaphana	2.4217E+02	5	2.1795E-01	Makarieva et al. 2008
2266	Invertebrates	Japetella heathi	1.6250E+02	5	1.0823E-01	Makarieva et al. 2008
2267	Invertebrates	Julidae	7.0050E-03	30	4.0075E-03	Ehnes et al. 2011
2268	Invertebrates	Julidae	7.3300E-03	5	2.1860E-04	Ehnes et al. 2011
2269	Invertebrates	Julidae	7.4400E-03	10	6.5575E-04	Ehnes et al. 2011
2270	Invertebrates	Julidae	7.8050E-03	30	4.0075E-03	Ehnes et al. 2011
2271	Invertebrates	Julidae	8.4550E-03	10	5.1005E-04	Ehnes et al. 2011
2272	Invertebrates	Julidae	8.6350E-03	30	4.0803E-03	Ehnes et al. 2011

2273	Invertebrates	Julidae	8.8500E-03	30	1.0492E-02	Ehnes et al. 2011
2274	Invertebrates	Julidae	9.1450E-03	30	5.6833E-03	Ehnes et al. 2011
2275	Invertebrates	Julidae	9.5700E-03	14.8	1.6030E-03	Ehnes et al. 2011
2276	Invertebrates	Julidae	9.7250E-03	30	4.2989E-03	Ehnes et al. 2011
2277	Invertebrates	Julidae	9.8900E-03	30	4.4447E-03	Ehnes et al. 2011
2278	Invertebrates	Julidae	1.0430E-02	14.8	1.7487E-03	Ehnes et al. 2011
2279	Invertebrates	Julidae	1.0800E-02	23.4	4.2989E-03	Ehnes et al. 2011
2280	Invertebrates	Julidae	1.1110E-02	23.4	5.9748E-03	Ehnes et al. 2011
2281	Invertebrates	Julidae	1.1220E-02	5	2.6959E-03	Ehnes et al. 2011
2282	Invertebrates	Julidae	1.1440E-02	9.9	5.1005E-04	Ehnes et al. 2011
2283	Invertebrates	Julidae	1.2290E-02	19.9	7.1406E-03	Ehnes et al. 2011
2284	Invertebrates	Julidae	1.2510E-02	10	5.8290E-04	Ehnes et al. 2011
2285	Invertebrates	Julidae	1.3240E-02	20.2	3.4974E-03	Ehnes et al. 2011
2286	Invertebrates	Julidae	1.3700E-02	14.6	2.7688E-03	Ehnes et al. 2011
2287	Invertebrates	Julidae	1.4770E-02	19.9	4.0803E-03	Ehnes et al. 2011
2288	Invertebrates	Julidae	1.6100E-02	30	6.1934E-03	Ehnes et al. 2011
2289	Invertebrates	Julidae	1.6945E-02	9.9	8.0150E-04	Ehnes et al. 2011
2290	Invertebrates	Julidae	1.9020E-02	30	9.4722E-03	Ehnes et al. 2011
2291	Invertebrates	Julidae	1.9410E-02	30	8.5978E-03	Ehnes et al. 2011
2292	Invertebrates	Julidae	2.0430E-02	19.9	4.3718E-03	Ehnes et al. 2011
2293	Invertebrates	Julidae	2.0500E-02	23.4	6.2662E-03	Ehnes et al. 2011
2294	Invertebrates	Julidae	2.1080E-02	14.6	4.3718E-03	Ehnes et al. 2011
2295	Invertebrates	Julidae	2.3320E-02	29.9	1.1367E-02	Ehnes et al. 2011
2296	Invertebrates	Julidae	2.3980E-02	19.9	5.8290E-03	Ehnes et al. 2011
2297	Invertebrates	Julidae	2.8180E-02	14.6	3.6432E-03	Ehnes et al. 2011
2298	Invertebrates	Julidae	2.8490E-02	29.9	1.2824E-02	Ehnes et al. 2011

2299	Invertebrates	Julidae	2.9220E-02	29.9	1.3990E-02	Ehnes et al. 2011
2300	Invertebrates	Julidae	2.9920E-02	20.2	7.4320E-03	Ehnes et al. 2011
2301	Invertebrates	Julidae	2.9990E-02	29.9	9.6179E-03	Ehnes et al. 2011
2302	Invertebrates	Julidae	3.1420E-02	29.9	1.6176E-02	Ehnes et al. 2011
2303	Invertebrates	Julidae	3.5730E-02	29.9	1.3261E-02	Ehnes et al. 2011
2304	Invertebrates	Julidae	4.0270E-02	30	3.4974E-01	Ehnes et al. 2011
2305	Invertebrates	Julidae	4.1040E-02	14.6	4.8090E-03	Ehnes et al. 2011
2306	Invertebrates	Julidae	4.3120E-02	5	1.1512E-02	Ehnes et al. 2011
2307	Invertebrates	Julidae	4.8630E-02	19.9	4.5175E-03	Ehnes et al. 2011
2308	Invertebrates	Julidae	5.5850E-02	5	7.2865E-04	Ehnes et al. 2011
2309	Invertebrates	Julidae	5.7700E-02	19.9	8.4521E-03	Ehnes et al. 2011
2310	Invertebrates	Julidae	5.9610E-02	10	1.1658E-03	Ehnes et al. 2011
2311	Invertebrates	Julidae	6.1190E-02	5	8.7435E-04	Ehnes et al. 2011
2312	Invertebrates	Julidae	6.3240E-02	10.5	3.7889E-03	Ehnes et al. 2011
2313	Invertebrates	Julidae	6.3710E-02	29.9	1.4427E-02	Ehnes et al. 2011
2314	Invertebrates	Julidae	6.9470E-02	5	1.7487E-03	Ehnes et al. 2011
2315	Invertebrates	Julidae	7.3060E-02	10.5	8.0149E-03	Ehnes et al. 2011
2316	Invertebrates	Julidae	7.3170E-02	5	2.3316E-03	Ehnes et al. 2011
2317	Invertebrates	Julidae	7.5360E-02	14.6	5.1004E-03	Ehnes et al. 2011
2318	Invertebrates	Julidae	7.6070E-02	20.2	9.3265E-03	Ehnes et al. 2011
2319	Invertebrates	Julidae	8.5900E-02	10.3	6.4119E-03	Ehnes et al. 2011
2320	Invertebrates	Julidae	8.8660E-02	10.5	3.6432E-03	Ehnes et al. 2011
2321	Invertebrates	Julidae	9.7030E-02	10.3	4.3718E-03	Ehnes et al. 2011
2322	Invertebrates	Julidae	9.7640E-02	14.9	1.3500E-02	Ehnes et al. 2011
2323	Invertebrates	Julidae	1.0035E-01	14.9	4.0000E-03	Ehnes et al. 2011
2324	Invertebrates	Julidae	1.0111E-01	15.6	9.1807E-03	Ehnes et al. 2011

2325	Invertebrates	Julidae	1.0111E-01	14.9	8.0000E-03	Ehnes et al. 2011
2326	Invertebrates	Julidae	1.0245E-01	10.5	5.6833E-03	Ehnes et al. 2011
2327	Invertebrates	Julidae	1.0457E-01	19.8	9.5000E-03	Ehnes et al. 2011
2328	Invertebrates	Julidae	1.0678E-01	19.8	1.6000E-02	Ehnes et al. 2011
2329	Invertebrates	Julidae	1.0735E-01	23.4	1.9527E-02	Ehnes et al. 2011
2330	Invertebrates	Julidae	1.0784E-01	14.9	6.5000E-03	Ehnes et al. 2011
2331	Invertebrates	Julidae	1.1457E-01	10.3	5.9748E-03	Ehnes et al. 2011
2332	Invertebrates	Julidae	1.1607E-01	11.3	6.0000E-03	Ehnes et al. 2011
2333	Invertebrates	Julidae	1.1622E-01	11.3	2.5000E-03	Ehnes et al. 2011
2334	Invertebrates	Julidae	1.1736E-01	11.3	9.5000E-03	Ehnes et al. 2011
2335	Invertebrates	Julidae	1.1856E-01	14.9	1.0500E-02	Ehnes et al. 2011
2336	Invertebrates	Julidae	1.2181E-01	14.9	6.0000E-03	Ehnes et al. 2011
2337	Invertebrates	Julidae	1.2904E-01	14.9	1.6500E-02	Ehnes et al. 2011
2338	Invertebrates	Julidae	1.2958E-01	29.9	2.4482E-02	Ehnes et al. 2011
2339	Invertebrates	Julidae	1.3028E-01	14.9	8.5000E-03	Ehnes et al. 2011
2340	Invertebrates	Julidae	1.3326E-01	14.9	8.0000E-03	Ehnes et al. 2011
2341	Invertebrates	Julidae	1.3652E-01	10.5	4.0803E-03	Ehnes et al. 2011
2342	Invertebrates	Julidae	1.4010E-01	19.8	3.0000E-03	Ehnes et al. 2011
2343	Invertebrates	Julidae	1.4083E-01	10.5	4.3718E-03	Ehnes et al. 2011
2344	Invertebrates	Julidae	1.4092E-01	5	3.6432E-03	Ehnes et al. 2011
2345	Invertebrates	Julidae	1.4181E-01	15.6	1.1658E-02	Ehnes et al. 2011
2346	Invertebrates	Julidae	1.4238E-01	19.8	1.1000E-02	Ehnes et al. 2011
2347	Invertebrates	Julidae	1.4256E-01	30	2.7396E-02	Ehnes et al. 2011
2348	Invertebrates	Julidae	1.4552E-01	5	3.6432E-03	Ehnes et al. 2011
2349	Invertebrates	Julidae	1.4602E-01	19.8	1.8500E-02	Ehnes et al. 2011
2350	Invertebrates	Julidae	1.4739E-01	23.4	1.8653E-02	Ehnes et al. 2011

2351	Invertebrates	Julidae	1.5036E-01	30	3.0894E-02	Ehnes et al. 2011
2352	Invertebrates	Julidae	1.5053E-01	10.3	7.1406E-03	Ehnes et al. 2011
2353	Invertebrates	Julidae	1.5054E-01	10.5	6.5577E-03	Ehnes et al. 2011
2354	Invertebrates	Julidae	1.5064E-01	11.3	6.0000E-03	Ehnes et al. 2011
2355	Invertebrates	Julidae	1.5077E-01	19.8	1.5500E-02	Ehnes et al. 2011
2356	Invertebrates	Julidae	1.5175E-01	5	3.7889E-03	Ehnes et al. 2011
2357	Invertebrates	Julidae	1.5460E-01	11.3	4.5000E-03	Ehnes et al. 2011
2358	Invertebrates	Julidae	1.5515E-01	11.3	1.0000E-02	Ehnes et al. 2011
2359	Invertebrates	Julidae	1.5576E-01	5	4.6632E-03	Ehnes et al. 2011
2360	Invertebrates	Julidae	1.5642E-01	10.3	4.6632E-03	Ehnes et al. 2011
2361	Invertebrates	Julidae	1.5684E-01	19.8	1.4500E-02	Ehnes et al. 2011
2362	Invertebrates	Julidae	1.5980E-01	10.3	4.2261E-03	Ehnes et al. 2011
2363	Invertebrates	Julidae	1.6313E-01	10.5	4.2261E-03	Ehnes et al. 2011
2364	Invertebrates	Julidae	1.6325E-01	14.9	1.4500E-02	Ehnes et al. 2011
2365	Invertebrates	Julidae	1.6452E-01	14.9	7.0000E-03	Ehnes et al. 2011
2366	Invertebrates	Julidae	1.6500E-01	14.9	6.5000E-03	Ehnes et al. 2011
2367	Invertebrates	Julidae	1.7239E-01	23.4	2.0402E-02	Ehnes et al. 2011
2368	Invertebrates	Julidae	1.7676E-01	11.3	1.0000E-02	Ehnes et al. 2011
2369	Invertebrates	Julidae	1.7979E-01	30	2.6522E-02	Ehnes et al. 2011
2370	Invertebrates	Julidae	1.8183E-01	5	5.6833E-03	Ehnes et al. 2011
2371	Invertebrates	Julidae	1.8281E-01	10.3	6.5577E-03	Ehnes et al. 2011
2372	Invertebrates	Julidae	1.8472E-01	5	4.0803E-03	Ehnes et al. 2011
2373	Invertebrates	Julidae	1.8606E-01	19.8	1.9500E-02	Ehnes et al. 2011
2374	Invertebrates	Julidae	1.8794E-01	19.8	1.1000E-02	Ehnes et al. 2011
2375	Invertebrates	Julidae	1.8890E-01	5.8	2.7688E-03	Ehnes et al. 2011
2376	Invertebrates	Julidae	1.9106E-01	14.9	2.6500E-02	Ehnes et al. 2011

2377	Invertebrates	Julidae	1.9717E-01	5	4.6632E-03	Ehnes et al. 2011
2378	Invertebrates	Julidae	2.0259E-01	11.3	9.5000E-03	Ehnes et al. 2011
2379	Invertebrates	Julidae	2.0356E-01	15.6	1.0784E-02	Ehnes et al. 2011
2380	Invertebrates	Julidae	2.0562E-01	14.9	2.9000E-02	Ehnes et al. 2011
2381	Invertebrates	Julidae	2.0569E-01	29.9	2.4336E-02	Ehnes et al. 2011
2382	Invertebrates	Julidae	2.0692E-01	30	1.7050E-02	Ehnes et al. 2011
2383	Invertebrates	Julidae	2.0716E-01	10.3	5.1004E-03	Ehnes et al. 2011
2384	Invertebrates	Julidae	2.3855E-01	15.6	1.2532E-02	Ehnes et al. 2011
2385	Invertebrates	Julidae	2.6316E-01	29.9	4.0075E-02	Ehnes et al. 2011
2386	Invertebrates	Julidae	2.6639E-01	5	2.1859E-03	Ehnes et al. 2011
2387	Invertebrates	Julidae	2.6642E-01	11.3	1.0500E-02	Ehnes et al. 2011
2388	Invertebrates	Julidae	2.7477E-01	5	7.7235E-03	Ehnes et al. 2011
2389	Invertebrates	Julidae	2.8003E-01	14.9	1.6500E-02	Ehnes et al. 2011
2390	Invertebrates	Julidae	2.8208E-01	19.8	2.6000E-02	Ehnes et al. 2011
2391	Invertebrates	Julidae	2.8671E-01	29.9	3.4391E-02	Ehnes et al. 2011
2392	Invertebrates	Julidae	2.9196E-01	30	3.4100E-02	Ehnes et al. 2011
2393	Invertebrates	Julidae	2.9349E-01	5.8	5.3919E-03	Ehnes et al. 2011
2394	Invertebrates	Karoophasma biedouwensis	1.0395E-01	25	5.7222E-02	Ehnes et al. 2011
2395	Invertebrates	Labidocera acuta	9.9000E-04	30	2.8612E-03	Makarieva et al. 2008
2396	Invertebrates	Labidocera acuta	1.1400E-03	28.5	2.0520E-03	Makarieva et al. 2008
2397	Invertebrates	Labidocera acuta	1.1700E-03	24	1.8954E-03	Makarieva et al. 2008
2398	Invertebrates	Labidocera detruncata	9.3000E-04	28	7.1888E-03	Makarieva et al. 2008
2399	Invertebrates	Labidocera jollae	4.1600E-02	20	2.0340E-02	Makarieva et al. 2008
2400	Invertebrates	Labidocera jollae	4.2000E-02	17	1.6299E-02	Makarieva et al. 2008
2401	Invertebrates	Labidocera sp.	8.2000E-04	30	2.6896E-03	Makarieva et al. 2008
2402	Invertebrates	Labritermes kistneri	7.0000E-04	25	3.6000E-04	Ehnes et al. 2011

2403	Invertebrates	Landrumius gigas	3.0900E-02	5	1.5796E-03	Makarieva et al. 2008
2404	Invertebrates	Lasius alienus	1.3800E-03	25	1.0800E-03	Ehnes et al. 2011
2405	Invertebrates	Lasius flavus	2.5800E-03	25	2.0700E-03	Ehnes et al. 2011
2406	Invertebrates	Lasius niger	1.7400E-03	25	1.0980E-03	Ehnes et al. 2011
2407	Invertebrates	Lasius sitiens	2.9000E-04	25	5.7600E-04	Ehnes et al. 2011
2408	Invertebrates	Lepidocyrtus	7.0000E-05	6	1.6500E-05	Ehnes et al. 2011
2409	Invertebrates	Lepidocyrtus	7.0000E-05	10	3.3950E-05	Ehnes et al. 2011
2410	Invertebrates	Lepidocyrtus	7.0000E-05	15	4.1450E-05	Ehnes et al. 2011
2411	Invertebrates	Leptinotarsa decemlineata	1.5000E-01	25	1.2490E-01	Ehnes et al. 2011
2412	Invertebrates	Leptogenys attenuata	4.0000E-03	25	2.2140E-03	Ehnes et al. 2011
2413	Invertebrates	Leptogenys nitida	1.7200E-03	25	9.3600E-04	Ehnes et al. 2011
2414	Invertebrates	Leptogenys schwabi	8.9600E-03	25	3.7800E-03	Ehnes et al. 2011
2415	Invertebrates	Leptothorax acerovorum	3.7000E-04	25	9.1800E-04	Ehnes et al. 2011
2416	Invertebrates	Leptothorax unifasciatus	4.9000E-04	25	4.3200E-04	Ehnes et al. 2011
2417	Invertebrates	Leucocelis elegans	8.0000E-02	25	6.0660E-02	Ehnes et al. 2011
2418	Invertebrates	Leucophaea maderae	2.8000E+00	25	5.2270E-01	Ehnes et al. 2011
2419	Invertebrates	Leukartiara octona	1.5000E-01	5	1.3770E-03	Makarieva et al. 2008
2420	Invertebrates	Liacarus coracinus	9.0000E-05	5	7.6500E-06	Ehnes et al. 2011
2421	Invertebrates	Liacarus coracinus	9.0000E-05	10	1.1950E-05	Ehnes et al. 2011
2422	Invertebrates	Liacarus coracinus	9.0000E-05	15	2.8400E-05	Ehnes et al. 2011
2423	Invertebrates	Liacarus xylariae	1.1300E-04	10	1.3150E-05	Ehnes et al. 2011
2424	Invertebrates	Liacarus xylariae	2.0700E-04	10	1.8700E-05	Ehnes et al. 2011
2425	Invertebrates	Libellula auripennis	4.6400E-01	25	2.5130E-01	Ehnes et al. 2011
2426	Invertebrates	Libellula needhami	5.1800E-01	25	3.0155E-01	Ehnes et al. 2011
2427	Invertebrates	Ligia oceanica	8.0000E-01	20	1.2802E-01	Makarieva et al. 2008
2428	Invertebrates	Ligia oceanica	8.0000E-01	20	1.3594E-01	Makarieva et al. 2008

2429	Invertebrates	Ligidium japonica	4.5000E-03	20	1.7998E-03	Makarieva et al. 2008
2430	Invertebrates	Ligidium japonica	2.5000E-02	20	5.7510E-03	Makarieva et al. 2008
2431	Invertebrates	Liocranchia pacificus	2.1200E+00	5	1.1410E-02	Makarieva et al. 2008
2432	Invertebrates	Liocranchia valdivia	2.1280E+01	5	1.2870E-01	Makarieva et al. 2008
2433	Invertebrates	Lirimiris sp.	5.6400E-01	25	3.1777E-01	Ehnes et al. 2011
2434	Invertebrates	Lithobius forficatus	7.5000E-04	14.6	5.8290E-04	Ehnes et al. 2011
2435	Invertebrates	Lithobius forficatus	8.5000E-04	14.7	1.3116E-03	Ehnes et al. 2011
2436	Invertebrates	Lithobius forficatus	8.5000E-04	14.7	1.4573E-03	Ehnes et al. 2011
2437	Invertebrates	Lithobius forficatus	9.4000E-04	14.7	1.4573E-03	Ehnes et al. 2011
2438	Invertebrates	Lithobius forficatus	9.6000E-04	15	1.3116E-03	Ehnes et al. 2011
2439	Invertebrates	Lithobius forficatus	1.1000E-03	15	1.1658E-03	Ehnes et al. 2011
2440	Invertebrates	Lithobius forficatus	1.1300E-03	20	1.6030E-03	Ehnes et al. 2011
2441	Invertebrates	Lithobius forficatus	1.3900E-03	20	2.1859E-03	Ehnes et al. 2011
2442	Invertebrates	Lithobius forficatus	1.5500E-03	15	1.1658E-03	Ehnes et al. 2011
2443	Invertebrates	Lithobius forficatus	1.6300E-03	20	2.4774E-03	Ehnes et al. 2011
2444	Invertebrates	Lithobius forficatus	1.7200E-03	20	2.6231E-03	Ehnes et al. 2011
2445	Invertebrates	Lithobius forficatus	2.0300E-03	20	1.7487E-03	Ehnes et al. 2011
2446	Invertebrates	Lithobius forficatus	2.4100E-03	30	9.7636E-03	Ehnes et al. 2011
2447	Invertebrates	Lithobius forficatus	2.4100E-03	14.6	8.7435E-04	Ehnes et al. 2011
2448	Invertebrates	Lithobius forficatus	2.6000E-03	30	1.5884E-02	Ehnes et al. 2011
2449	Invertebrates	Lithobius forficatus	2.6700E-03	15	2.0402E-03	Ehnes et al. 2011
2450	Invertebrates	Lithobius forficatus	2.7800E-03	14.6	1.1658E-03	Ehnes et al. 2011
2451	Invertebrates	Lithobius forficatus	2.8800E-03	20	1.8945E-03	Ehnes et al. 2011
2452	Invertebrates	Lithobius forficatus	3.2300E-03	30	1.6321E-02	Ehnes et al. 2011
2453	Invertebrates	Lithobius forficatus	3.4400E-03	30	1.1512E-02	Ehnes et al. 2011
2454	Invertebrates	Lithobius forficatus	5.5900E-03	15	2.9145E-03	Ehnes et al. 2011

2455	Invertebrates	Lithobius forficatus	5.5900E-03	29.9	8.3064E-03	Ehnes et al. 2011
2456	Invertebrates	Lithobius forficatus	6.0500E-03	29.9	9.4722E-03	Ehnes et al. 2011
2457	Invertebrates	Lithobius forficatus	6.5600E-03	29.9	1.1221E-02	Ehnes et al. 2011
2458	Invertebrates	Lithobius forficatus	6.6800E-03	29.9	9.3265E-03	Ehnes et al. 2011
2459	Invertebrates	Lithobius forficatus	7.1000E-03	29.9	8.8893E-03	Ehnes et al. 2011
2460	Invertebrates	Lithobius forficatus	7.3300E-03	5.2	1.0201E-03	Ehnes et al. 2011
2461	Invertebrates	Lithobius forficatus	7.4600E-03	10	1.0201E-03	Ehnes et al. 2011
2462	Invertebrates	Lithobius forficatus	8.0800E-03	10	4.3720E-04	Ehnes et al. 2011
2463	Invertebrates	Lithobius forficatus	8.1400E-03	5	8.7435E-04	Ehnes et al. 2011
2464	Invertebrates	Lithobius forficatus	8.3950E-03	5	7.2865E-04	Ehnes et al. 2011
2465	Invertebrates	Lithobius forficatus	8.9600E-03	10	7.2865E-04	Ehnes et al. 2011
2466	Invertebrates	Lithobius forficatus	9.0400E-03	29.9	1.0201E-02	Ehnes et al. 2011
2467	Invertebrates	Lithobius forficatus	1.0040E-02	10	1.1658E-03	Ehnes et al. 2011
2468	Invertebrates	Lithobius forficatus	1.0510E-02	5	3.4974E-03	Ehnes et al. 2011
2469	Invertebrates	Lithobius forficatus	1.1310E-02	5.8	2.4774E-03	Ehnes et al. 2011
2470	Invertebrates	Lithobius forficatus	1.1670E-02	24.5	1.7050E-02	Ehnes et al. 2011
2471	Invertebrates	Lithobius forficatus	1.2060E-02	10	1.8945E-03	Ehnes et al. 2011
2472	Invertebrates	Lithobius forficatus	1.2080E-02	24.5	1.5738E-02	Ehnes et al. 2011
2473	Invertebrates	Lithobius forficatus	1.2300E-02	5	4.3720E-04	Ehnes et al. 2011
2474	Invertebrates	Lithobius forficatus	1.2560E-02	10	1.3116E-03	Ehnes et al. 2011
2475	Invertebrates	Lithobius forficatus	1.3380E-02	14.6	2.4774E-03	Ehnes et al. 2011
2476	Invertebrates	Lithobius forficatus	1.3400E-02	10	3.2060E-03	Ehnes et al. 2011
2477	Invertebrates	Lithobius forficatus	1.3610E-02	5	3.1331E-03	Ehnes et al. 2011
2478	Invertebrates	Lithobius forficatus	1.4350E-02	5.8	4.3720E-04	Ehnes et al. 2011
2479	Invertebrates	Lithobius forficatus	1.4420E-02	20	5.9748E-03	Ehnes et al. 2011
2480	Invertebrates	Lithobius forficatus	1.4570E-02	9.5	5.6833E-03	Ehnes et al. 2011

2481	Invertebrates	Lithobius forficatus	1.5020E-02	5.8	4.3720E-04	Ehnes et al. 2011
2482	Invertebrates	Lithobius forficatus	1.5490E-02	20	5.1004E-03	Ehnes et al. 2011
2483	Invertebrates	Lithobius forficatus	1.5510E-02	10	2.6231E-03	Ehnes et al. 2011
2484	Invertebrates	Lithobius forficatus	1.5840E-02	20	1.4573E-02	Ehnes et al. 2011
2485	Invertebrates	Lithobius forficatus	1.6160E-02	10	2.1859E-03	Ehnes et al. 2011
2486	Invertebrates	Lithobius forficatus	1.6300E-02	24.1	9.9094E-03	Ehnes et al. 2011
2487	Invertebrates	Lithobius forficatus	1.6930E-02	10	7.2865E-04	Ehnes et al. 2011
2488	Invertebrates	Lithobius forficatus	1.7410E-02	5	2.0402E-03	Ehnes et al. 2011
2489	Invertebrates	Lithobius forficatus	1.7470E-02	14.6	4.9547E-03	Ehnes et al. 2011
2490	Invertebrates	Lithobius forficatus	1.7940E-02	20	5.6833E-03	Ehnes et al. 2011
2491	Invertebrates	Lithobius forficatus	1.8200E-02	20	4.3718E-03	Ehnes et al. 2011
2492	Invertebrates	Lithobius forficatus	1.8250E-02	10	4.0803E-03	Ehnes et al. 2011
2493	Invertebrates	Lithobius forficatus	1.8352E-02	5	1.0055E-02	Ehnes et al. 2011
2494	Invertebrates	Lithobius forficatus	1.9200E-02	20	4.2261E-03	Ehnes et al. 2011
2495	Invertebrates	Lithobius forficatus	1.9215E-02	5	5.1005E-04	Ehnes et al. 2011
2496	Invertebrates	Lithobius forficatus	1.9610E-02	9.5	2.7688E-03	Ehnes et al. 2011
2497	Invertebrates	Lithobius forficatus	2.0060E-02	20	6.5577E-03	Ehnes et al. 2011
2498	Invertebrates	Lithobius forficatus	2.0890E-02	20	7.2863E-03	Ehnes et al. 2011
2499	Invertebrates	Lithobius forficatus	2.1070E-02	20	8.1606E-03	Ehnes et al. 2011
2500	Invertebrates	Lithobius forficatus	2.1450E-02	10	3.7889E-03	Ehnes et al. 2011
2501	Invertebrates	Lithobius forficatus	2.1510E-02	20	7.5777E-03	Ehnes et al. 2011
2502	Invertebrates	Lithobius forficatus	2.1530E-02	5	3.7889E-03	Ehnes et al. 2011
2503	Invertebrates	Lithobius forficatus	2.2260E-02	20	9.9093E-03	Ehnes et al. 2011
2504	Invertebrates	Lithobius forficatus	2.3060E-02	20	3.0603E-03	Ehnes et al. 2011
2505	Invertebrates	Lithobius forficatus	2.3110E-02	20	1.0638E-02	Ehnes et al. 2011
2506	Invertebrates	Lithobius forficatus	2.3810E-02	10	5.3919E-03	Ehnes et al. 2011

2507	Invertebrates	Lithobius forficatus	2.3940E-02	10	4.1635E-04	Ehnes et al. 2011
2508	Invertebrates	Lithobius forficatus	2.4260E-02	5	1.5010E-02	Ehnes et al. 2011
2509	Invertebrates	Lithobius forficatus	2.4790E-02	10	3.9346E-03	Ehnes et al. 2011
2510	Invertebrates	Lithobius forficatus	2.4850E-02	25	1.4718E-02	Ehnes et al. 2011
2511	Invertebrates	Lithobius forficatus	2.5210E-02	10	3.2060E-03	Ehnes et al. 2011
2512	Invertebrates	Lithobius forficatus	2.6000E-02	15	4.3718E-03	Ehnes et al. 2011
2513	Invertebrates	Lithobius forficatus	2.6250E-02	10	1.6030E-03	Ehnes et al. 2011
2514	Invertebrates	Lithobius forficatus	2.6450E-02	14.6	3.2060E-03	Ehnes et al. 2011
2515	Invertebrates	Lithobius forficatus	2.7000E-02	24.5	2.4190E-02	Ehnes et al. 2011
2516	Invertebrates	Lithobius forficatus	2.7280E-02	5	1.0201E-03	Ehnes et al. 2011
2517	Invertebrates	Lithobius forficatus	2.7630E-02	30	1.9382E-02	Ehnes et al. 2011
2518	Invertebrates	Lithobius forficatus	2.8550E-02	10	3.4974E-03	Ehnes et al. 2011
2519	Invertebrates	Lithobius forficatus	2.8710E-02	10	2.4774E-03	Ehnes et al. 2011
2520	Invertebrates	Lithobius forficatus	2.8990E-02	24.1	2.4628E-02	Ehnes et al. 2011
2521	Invertebrates	Lithobius forficatus	2.9330E-02	10	4.8090E-03	Ehnes et al. 2011
2522	Invertebrates	Lithobius forficatus	2.9750E-02	10	3.0603E-03	Ehnes et al. 2011
2523	Invertebrates	Lithobius forficatus	2.9950E-02	10	5.9748E-03	Ehnes et al. 2011
2524	Invertebrates	Lithobius forficatus	3.0030E-02	5	1.6030E-03	Ehnes et al. 2011
2525	Invertebrates	Lithobius forficatus	3.0200E-02	10	4.0803E-03	Ehnes et al. 2011
2526	Invertebrates	Lithobius forficatus	3.0970E-02	5	2.6231E-03	Ehnes et al. 2011
2527	Invertebrates	Lithobius forficatus	3.1110E-02	10	2.6231E-03	Ehnes et al. 2011
2528	Invertebrates	Lithobius forficatus	3.1380E-02	25	1.3698E-02	Ehnes et al. 2011
2529	Invertebrates	Lithobius forficatus	3.1720E-02	29.6	2.3316E-02	Ehnes et al. 2011
2530	Invertebrates	Lithobius forficatus	3.2170E-02	25	1.4281E-02	Ehnes et al. 2011
2531	Invertebrates	Lithobius forficatus	3.3280E-02	10	2.7688E-03	Ehnes et al. 2011
2532	Invertebrates	Lithobius forficatus	3.3690E-02	5	6.7034E-03	Ehnes et al. 2011

2533	Invertebrates	Lithobius forficatus	3.3710E-02	30	2.4336E-02	Ehnes et al. 2011
2534	Invertebrates	Lithobius forficatus	3.4310E-02	10	9.7636E-03	Ehnes et al. 2011
2535	Invertebrates	Lithobius forficatus	3.4540E-02	30	1.8507E-02	Ehnes et al. 2011
2536	Invertebrates	Lithobius forficatus	3.5380E-02	25	1.7050E-02	Ehnes et al. 2011
2537	Invertebrates	Lithobius forficatus	3.5900E-02	24.5	3.4828E-02	Ehnes et al. 2011
2538	Invertebrates	Lithobius forficatus	3.7000E-02	30	2.3170E-02	Ehnes et al. 2011
2539	Invertebrates	Lithobius forficatus	3.8090E-02	24.5	4.2260E-02	Ehnes et al. 2011
2540	Invertebrates	Lithobius forficatus	3.8310E-02	15	3.9346E-03	Ehnes et al. 2011
2541	Invertebrates	Lithobius forficatus	3.9730E-02	24.1	1.9673E-02	Ehnes et al. 2011
2542	Invertebrates	Lithobius forficatus	3.9750E-02	10	3.2351E-02	Ehnes et al. 2011
2543	Invertebrates	Lithobius forficatus	4.0560E-02	30	1.7779E-02	Ehnes et al. 2011
2544	Invertebrates	Lithobius forficatus	4.0580E-02	5	4.3720E-04	Ehnes et al. 2011
2545	Invertebrates	Lithobius forficatus	4.0990E-02	24.1	2.5356E-02	Ehnes et al. 2011
2546	Invertebrates	Lithobius forficatus	4.1010E-02	10	1.4573E-03	Ehnes et al. 2011
2547	Invertebrates	Lithobius forficatus	4.1030E-02	24.1	2.4336E-02	Ehnes et al. 2011
2548	Invertebrates	Lithobius forficatus	4.2000E-02	25	2.2442E-02	Ehnes et al. 2011
2549	Invertebrates	Lithobius forficatus	4.2780E-02	24.1	1.5447E-02	Ehnes et al. 2011
2550	Invertebrates	Lithobius forficatus	4.6950E-02	5	1.0201E-03	Ehnes et al. 2011
2551	Invertebrates	Lithobius forficatus	4.7440E-02	4	8.4521E-03	Ehnes et al. 2011
2552	Invertebrates	Lithobius forficatus	4.8320E-02	10	1.0055E-02	Ehnes et al. 2011
2553	Invertebrates	Lithobius forficatus	4.9110E-02	5	2.6231E-03	Ehnes et al. 2011
2554	Invertebrates	Lithobius forficatus	4.9130E-02	10	2.9145E-03	Ehnes et al. 2011
2555	Invertebrates	Lithobius forficatus	4.9630E-02	5	2.1130E-02	Ehnes et al. 2011
2556	Invertebrates	Lithobius forficatus	5.1940E-02	14.6	1.3407E-02	Ehnes et al. 2011
2557	Invertebrates	Lithobius forficatus	5.4830E-02	10	2.0402E-03	Ehnes et al. 2011
2558	Invertebrates	Lithobius forficatus	5.5590E-02	5	5.3919E-03	Ehnes et al. 2011

2559	Invertebrates	Lithobius forficatus	5.5840E-02	30	2.8416E-02	Ehnes et al. 2011
2560	Invertebrates	Lithobius forficatus	5.5880E-02	14.6	5.3919E-03	Ehnes et al. 2011
2561	Invertebrates	Lithobius forficatus	5.8110E-02	15	1.1367E-02	Ehnes et al. 2011
2562	Invertebrates	Lithobius forficatus	6.2820E-02	20	2.0547E-02	Ehnes et al. 2011
2563	Invertebrates	Lithobius forficatus	6.3050E-02	14.6	5.9748E-03	Ehnes et al. 2011
2564	Invertebrates	Lithobius forficatus	6.3080E-02	10	1.8945E-03	Ehnes et al. 2011
2565	Invertebrates	Lithobius forficatus	6.5540E-02	14.6	3.2351E-02	Ehnes et al. 2011
2566	Invertebrates	Lithobius forficatus	6.5780E-02	20	6.9948E-03	Ehnes et al. 2011
2567	Invertebrates	Lithobius forficatus	6.7100E-02	5	2.3316E-03	Ehnes et al. 2011
2568	Invertebrates	Lithobius forficatus	6.7860E-02	20	6.9948E-03	Ehnes et al. 2011
2569	Invertebrates	Lithobius forficatus	6.9080E-02	20	1.7487E-02	Ehnes et al. 2011
2570	Invertebrates	Lithobius forficatus	6.9650E-02	25	2.4919E-02	Ehnes et al. 2011
2571	Invertebrates	Lithobius forficatus	7.1550E-02	10	1.3844E-03	Ehnes et al. 2011
2572	Invertebrates	Lithobius forficatus	7.2060E-02	5	2.3316E-03	Ehnes et al. 2011
2573	Invertebrates	Lithobius forficatus	7.2950E-02	25	3.5120E-02	Ehnes et al. 2011
2574	Invertebrates	Lithobius forficatus	7.3340E-02	20	1.2824E-02	Ehnes et al. 2011
2575	Invertebrates	Lithobius forficatus	7.4120E-02	20	1.7487E-02	Ehnes et al. 2011
2576	Invertebrates	Lithobius forficatus	7.5260E-02	20	1.7487E-02	Ehnes et al. 2011
2577	Invertebrates	Lithobius forficatus	7.5350E-02	5	3.0602E-02	Ehnes et al. 2011
2578	Invertebrates	Lithobius forficatus	7.8240E-02	30	2.3025E-02	Ehnes et al. 2011
2579	Invertebrates	Lithobius forficatus	7.8840E-02	25	4.2260E-02	Ehnes et al. 2011
2580	Invertebrates	Lithobius forficatus	7.9450E-02	20	1.8507E-02	Ehnes et al. 2011
2581	Invertebrates	Lithobius forficatus	7.9460E-02	20	1.7924E-02	Ehnes et al. 2011
2582	Invertebrates	Lithobius forficatus	7.9470E-02	25	3.0602E-02	Ehnes et al. 2011
2583	Invertebrates	Lithobius forficatus	7.9530E-02	5	2.4919E-02	Ehnes et al. 2011
2584	Invertebrates	Lithobius forficatus	8.1260E-02	30	5.7999E-02	Ehnes et al. 2011

2585	Invertebrates	Lithobius forficatus	8.2720E-02	20	1.8653E-02	Ehnes et al. 2011
2586	Invertebrates	Lithobius forficatus	8.2760E-02	14.6	7.4320E-03	Ehnes et al. 2011
2587	Invertebrates	Lithobius forficatus	8.2940E-02	20	2.2150E-02	Ehnes et al. 2011
2588	Invertebrates	Lithobius forficatus	8.3090E-02	10	5.3919E-03	Ehnes et al. 2011
2589	Invertebrates	Lithobius forficatus	8.3330E-02	20	1.8944E-02	Ehnes et al. 2011
2590	Invertebrates	Lithobius forficatus	8.3370E-02	25	4.4446E-02	Ehnes et al. 2011
2591	Invertebrates	Lithobius forficatus	8.5160E-02	25	7.2571E-02	Ehnes et al. 2011
2592	Invertebrates	Lithobius forficatus	8.5480E-02	25	6.7762E-02	Ehnes et al. 2011
2593	Invertebrates	Lithobius forficatus	8.5500E-02	10	3.9346E-03	Ehnes et al. 2011
2594	Invertebrates	Lithobius forficatus	8.5680E-02	20	1.8361E-02	Ehnes et al. 2011
2595	Invertebrates	Lithobius forficatus	8.6320E-02	30	2.1130E-02	Ehnes et al. 2011
2596	Invertebrates	Lithobius forficatus	8.8380E-02	10	2.3316E-03	Ehnes et al. 2011
2597	Invertebrates	Lithobius forficatus	8.9390E-02	30	3.1477E-02	Ehnes et al. 2011
2598	Invertebrates	Lithobius forficatus	8.9850E-02	30	2.5939E-02	Ehnes et al. 2011
2599	Invertebrates	Lithobius forficatus	9.1800E-02	20	9.3265E-03	Ehnes et al. 2011
2600	Invertebrates	Lithobius forficatus	9.2060E-02	20	2.1713E-02	Ehnes et al. 2011
2601	Invertebrates	Lithobius forficatus	9.3420E-02	14.6	3.2351E-02	Ehnes et al. 2011
2602	Invertebrates	Lithobius forficatus	9.5220E-02	20	2.2733E-02	Ehnes et al. 2011
2603	Invertebrates	Lithobius forficatus	9.5520E-02	30	2.8096E-01	Ehnes et al. 2011
2604	Invertebrates	Lithobius forficatus	9.6490E-02	25	3.2351E-02	Ehnes et al. 2011
2605	Invertebrates	Lithobius forficatus	9.6610E-02	25	2.5648E-02	Ehnes et al. 2011
2606	Invertebrates	Lithobius forficatus	9.6920E-02	4	2.4774E-03	Ehnes et al. 2011
2607	Invertebrates	Lithobius forficatus	9.7900E-02	10	4.3718E-03	Ehnes et al. 2011
2608	Invertebrates	Lithobius forficatus	9.8690E-02	25	1.6467E-02	Ehnes et al. 2011
2609	Invertebrates	Lithobius forficatus	9.9260E-02	10	2.6555E-03	Ehnes et al. 2011
2610	Invertebrates	Lithobius forficatus	9.9770E-02	15	1.0492E-02	Ehnes et al. 2011

2611	Invertebrates	Lithobius forficatus	1.0258E-01	10	4.6632E-03	Ehnes et al. 2011
2612	Invertebrates	Lithobius forficatus	1.0376E-01	15	1.9236E-02	Ehnes et al. 2011
2613	Invertebrates	Lithobius forficatus	1.0460E-01	25	3.4246E-02	Ehnes et al. 2011
2614	Invertebrates	Lithobius forficatus	1.0475E-01	15	2.1859E-02	Ehnes et al. 2011
2615	Invertebrates	Lithobius forficatus	1.0560E-01	30	3.6577E-02	Ehnes et al. 2011
2616	Invertebrates	Lithobius forficatus	1.0580E-01	25	4.8527E-02	Ehnes et al. 2011
2617	Invertebrates	Lithobius forficatus	1.0635E-01	20	2.8416E-02	Ehnes et al. 2011
2618	Invertebrates	Lithobius forficatus	1.0767E-01	30	5.3773E-02	Ehnes et al. 2011
2619	Invertebrates	Lithobius forficatus	1.0928E-01	10	6.5577E-03	Ehnes et al. 2011
2620	Invertebrates	Lithobius forficatus	1.0999E-01	15	1.4427E-02	Ehnes et al. 2011
2621	Invertebrates	Lithobius forficatus	1.1159E-01	20	2.2296E-02	Ehnes et al. 2011
2622	Invertebrates	Lithobius forficatus	1.1250E-01	25	2.4336E-02	Ehnes et al. 2011
2623	Invertebrates	Lithobius forficatus	1.1451E-01	5	4.3718E-03	Ehnes et al. 2011
2624	Invertebrates	Lithobius forficatus	1.1754E-01	30	8.6561E-02	Ehnes et al. 2011
2625	Invertebrates	Lithobius forficatus	1.1837E-01	10	5.6833E-03	Ehnes et al. 2011
2626	Invertebrates	Lithobius forficatus	1.1855E-01	5	5.8290E-04	Ehnes et al. 2011
2627	Invertebrates	Lithobius forficatus	1.2097E-01	10	1.0929E-02	Ehnes et al. 2011
2628	Invertebrates	Lithobius forficatus	1.2147E-01	25	2.6668E-02	Ehnes et al. 2011
2629	Invertebrates	Lithobius forficatus	1.2166E-01	30	3.2934E-02	Ehnes et al. 2011
2630	Invertebrates	Lithobius forficatus	1.2168E-01	15	1.1075E-02	Ehnes et al. 2011
2631	Invertebrates	Lithobius forficatus	1.2238E-01	20	1.9964E-02	Ehnes et al. 2011
2632	Invertebrates	Lithobius forficatus	1.2335E-01	24.1	1.0274E-01	Ehnes et al. 2011
2633	Invertebrates	Lithobius forficatus	1.2390E-01	15	1.3407E-02	Ehnes et al. 2011
2634	Invertebrates	Lithobius forficatus	1.2481E-01	30	4.5466E-02	Ehnes et al. 2011
2635	Invertebrates	Lithobius forficatus	1.2610E-01	10	4.5904E-02	Ehnes et al. 2011
2636	Invertebrates	Lithobius forficatus	1.2669E-01	25	2.4190E-02	Ehnes et al. 2011

2637	Invertebrates	Lithobius forficatus	1.2752E-01	25	2.3608E-02	Ehnes et al. 2011
2638	Invertebrates	Lithobius forficatus	1.2767E-01	20	1.9964E-02	Ehnes et al. 2011
2639	Invertebrates	Lithobius forficatus	1.2796E-01	4	2.6231E-03	Ehnes et al. 2011
2640	Invertebrates	Lithobius forficatus	1.2824E-01	30	9.8802E-02	Ehnes et al. 2011
2641	Invertebrates	Lithobius forficatus	1.2866E-01	25	2.3462E-02	Ehnes et al. 2011
2642	Invertebrates	Lithobius forficatus	1.2873E-01	14.6	1.6904E-02	Ehnes et al. 2011
2643	Invertebrates	Lithobius forficatus	1.2891E-01	10	2.9145E-03	Ehnes et al. 2011
2644	Invertebrates	Lithobius forficatus	1.2910E-01	20	2.2005E-02	Ehnes et al. 2011
2645	Invertebrates	Lithobius forficatus	1.3096E-01	10	1.5738E-02	Ehnes et al. 2011
2646	Invertebrates	Lithobius forficatus	1.3188E-01	4	1.8945E-03	Ehnes et al. 2011
2647	Invertebrates	Lithobius forficatus	1.3258E-01	10	3.3517E-03	Ehnes et al. 2011
2648	Invertebrates	Lithobius forficatus	1.3359E-01	10	6.1205E-03	Ehnes et al. 2011
2649	Invertebrates	Lithobius forficatus	1.3402E-01	20	8.8164E-02	Ehnes et al. 2011
2650	Invertebrates	Lithobius forficatus	1.3409E-01	20	2.4336E-02	Ehnes et al. 2011
2651	Invertebrates	Lithobius forficatus	1.3460E-01	20	1.3407E-02	Ehnes et al. 2011
2652	Invertebrates	Lithobius forficatus	1.3461E-01	5	4.5175E-03	Ehnes et al. 2011
2653	Invertebrates	Lithobius forficatus	1.3531E-01	14.6	2.1130E-02	Ehnes et al. 2011
2654	Invertebrates	Lithobius forficatus	1.3594E-01	30	9.8219E-02	Ehnes et al. 2011
2655	Invertebrates	Lithobius forficatus	1.3622E-01	10	8.5978E-03	Ehnes et al. 2011
2656	Invertebrates	Lithobius forficatus	1.3686E-01	5	2.3316E-03	Ehnes et al. 2011
2657	Invertebrates	Lithobius forficatus	1.3967E-01	20	2.5648E-02	Ehnes et al. 2011
2658	Invertebrates	Lithobius forficatus	1.4083E-01	10	4.2260E-02	Ehnes et al. 2011
2659	Invertebrates	Lithobius forficatus	1.4120E-01	20	2.5065E-02	Ehnes et al. 2011
2660	Invertebrates	Lithobius forficatus	1.4316E-01	20	1.5447E-02	Ehnes et al. 2011
2661	Invertebrates	Lithobius forficatus	1.4336E-01	20	2.6085E-02	Ehnes et al. 2011
2662	Invertebrates	Lithobius forficatus	1.4433E-01	20	1.8653E-02	Ehnes et al. 2011

2663	Invertebrates	Lithobius forficatus	1.4470E-01	20	3.6286E-02	Ehnes et al. 2011
2664	Invertebrates	Lithobius forficatus	1.4510E-01	5	5.2461E-03	Ehnes et al. 2011
2665	Invertebrates	Lithobius forficatus	1.4512E-01	20	1.8070E-02	Ehnes et al. 2011
2666	Invertebrates	Lithobius forficatus	1.4585E-01	20	1.5301E-02	Ehnes et al. 2011
2667	Invertebrates	Lithobius forficatus	1.4751E-01	10	2.7688E-03	Ehnes et al. 2011
2668	Invertebrates	Lithobius forficatus	1.4821E-01	25	2.3462E-02	Ehnes et al. 2011
2669	Invertebrates	Lithobius forficatus	1.4938E-01	15	1.5884E-02	Ehnes et al. 2011
2670	Invertebrates	Lithobius forficatus	1.4978E-01	5	2.1859E-03	Ehnes et al. 2011
2671	Invertebrates	Lithobius forficatus	1.5130E-01	10	1.0638E-02	Ehnes et al. 2011
2672	Invertebrates	Lithobius forficatus	1.5505E-01	20	4.0657E-02	Ehnes et al. 2011
2673	Invertebrates	Lithobius forficatus	1.6046E-01	20	2.6668E-02	Ehnes et al. 2011
2674	Invertebrates	Lithobius forficatus	1.6144E-01	20	3.5120E-02	Ehnes et al. 2011
2675	Invertebrates	Lithobius forficatus	1.6255E-01	10	9.6179E-03	Ehnes et al. 2011
2676	Invertebrates	Lithobius forficatus	1.6629E-01	20	3.4245E-02	Ehnes et al. 2011
2677	Invertebrates	Lithobius forficatus	1.6793E-01	20	3.2060E-02	Ehnes et al. 2011
2678	Invertebrates	Lithobius forficatus	1.7839E-01	24.1	4.7944E-02	Ehnes et al. 2011
2679	Invertebrates	Lithobius forficatus	1.8074E-01	10	8.4521E-03	Ehnes et al. 2011
2680	Invertebrates	Lithobius forficatus	1.8137E-01	5	2.6231E-03	Ehnes et al. 2011
2681	Invertebrates	Lithobius forficatus	1.8746E-01	4	2.1859E-03	Ehnes et al. 2011
2682	Invertebrates	Lithobius forficatus	3.3750E-03	30	5.0276E-03	Ehnes et al. 2011
2683	Invertebrates	Lithobius forficatus	9.2150E-03	5	8.7435E-04	Ehnes et al. 2011
2684	Invertebrates	Lithobius forficatus	9.2650E-03	5	1.4575E-04	Ehnes et al. 2011
2685	Invertebrates	Lithobius forficatus	1.0180E-02	10.2	1.4573E-03	Ehnes et al. 2011
2686	Invertebrates	Lixus bisulcatus	4.0500E-01	25	2.4426E-01	Ehnes et al. 2011
2687	Invertebrates	Locusta migratoria migratorioides	1.5000E+00	25	8.8812E-01	Ehnes et al. 2011
2688	Invertebrates	Loligo forbesi	9.3700E+02	5	8.7737E+01	Makarieva et al. 2008

2689	Invertebrates	Loligo opalescens	3.0000E+01	5	3.7163E+00	Makarieva et al. 2008
2690	Invertebrates	Loligo pealei	1.0000E+02	5	1.7248E+01	Makarieva et al. 2008
2691	Invertebrates	Lolliguncula brevis	4.1100E+01	5	3.5540E+00	Makarieva et al. 2008
2692	Invertebrates	Loricera pilicornis	1.2160E-02	20	4.9547E-03	Ehnes et al. 2011
2693	Invertebrates	Loricera pilicornis	1.2260E-02	30	1.7487E-02	Ehnes et al. 2011
2694	Invertebrates	Loricera pilicornis	1.2280E-02	15	6.1205E-03	Ehnes et al. 2011
2695	Invertebrates	Loricera pilicornis	1.2380E-02	25	1.2532E-02	Ehnes et al. 2011
2696	Invertebrates	Loricera pilicornis	1.3350E-02	15	5.9748E-03	Ehnes et al. 2011
2697	Invertebrates	Loricera pilicornis	1.3620E-02	25	1.1367E-02	Ehnes et al. 2011
2698	Invertebrates	Loricera pilicornis	1.3730E-02	15	6.2662E-03	Ehnes et al. 2011
2699	Invertebrates	Loricera pilicornis	1.4060E-02	20	4.9547E-03	Ehnes et al. 2011
2700	Invertebrates	Loricera pilicornis	1.4100E-02	15	4.0803E-03	Ehnes et al. 2011
2701	Invertebrates	Loricera pilicornis	1.4410E-02	25	9.4722E-03	Ehnes et al. 2011
2702	Invertebrates	Loricera pilicornis	1.4450E-02	10	2.3316E-03	Ehnes et al. 2011
2703	Invertebrates	Loricera pilicornis	1.4480E-02	10	2.1859E-03	Ehnes et al. 2011
2704	Invertebrates	Loricera pilicornis	1.4650E-02	25	1.3552E-02	Ehnes et al. 2011
2705	Invertebrates	Loricera pilicornis	1.4720E-02	5	2.3316E-03	Ehnes et al. 2011
2706	Invertebrates	Loricera pilicornis	1.4780E-02	30	1.0784E-02	Ehnes et al. 2011
2707	Invertebrates	Loricera pilicornis	1.4870E-02	10	2.4774E-03	Ehnes et al. 2011
2708	Invertebrates	Loricera pilicornis	1.4980E-02	15	7.1406E-03	Ehnes et al. 2011
2709	Invertebrates	Loricera pilicornis	1.5120E-02	15	4.5175E-03	Ehnes et al. 2011
2710	Invertebrates	Loricera pilicornis	1.5160E-02	15	8.5978E-03	Ehnes et al. 2011
2711	Invertebrates	Loricera pilicornis	1.5160E-02	20	4.2261E-03	Ehnes et al. 2011
2712	Invertebrates	Loricera pilicornis	1.5190E-02	30	1.1658E-02	Ehnes et al. 2011
2713	Invertebrates	Loricera pilicornis	1.5190E-02	10	2.7688E-03	Ehnes et al. 2011
2714	Invertebrates	Loricera pilicornis	1.5250E-02	5	2.6231E-03	Ehnes et al. 2011

2715	Invertebrates	Loricera pilicornis	1.5270E-02	20	5.5376E-03	Ehnes et al. 2011
2716	Invertebrates	Loricera pilicornis	1.5420E-02	20	1.2241E-02	Ehnes et al. 2011
2717	Invertebrates	Loricera pilicornis	1.5600E-02	20	6.4119E-03	Ehnes et al. 2011
2718	Invertebrates	Loricera pilicornis	1.5720E-02	30	2.8562E-02	Ehnes et al. 2011
2719	Invertebrates	Loricera pilicornis	1.5750E-02	10	1.1658E-03	Ehnes et al. 2011
2720	Invertebrates	Loricera pilicornis	1.5780E-02	30	1.8216E-02	Ehnes et al. 2011
2721	Invertebrates	Loricera pilicornis	1.5890E-02	25	1.4281E-02	Ehnes et al. 2011
2722	Invertebrates	Loricera pilicornis	1.6290E-02	5	3.2060E-03	Ehnes et al. 2011
2723	Invertebrates	Loricera pilicornis	1.6330E-02	20	6.7034E-03	Ehnes et al. 2011
2724	Invertebrates	Loricera pilicornis	1.6530E-02	15	9.3265E-03	Ehnes et al. 2011
2725	Invertebrates	Loricera pilicornis	1.6670E-02	10	2.3316E-03	Ehnes et al. 2011
2726	Invertebrates	Loricera pilicornis	1.6740E-02	30	2.2150E-02	Ehnes et al. 2011
2727	Invertebrates	Loricera pilicornis	1.6740E-02	5	1.8945E-03	Ehnes et al. 2011
2728	Invertebrates	Loricera pilicornis	1.6810E-02	20	7.5777E-03	Ehnes et al. 2011
2729	Invertebrates	Loricera pilicornis	1.7000E-02	20	6.5577E-03	Ehnes et al. 2011
2730	Invertebrates	Loricera pilicornis	1.7000E-02	25	1.3698E-02	Ehnes et al. 2011
2731	Invertebrates	Loricera pilicornis	1.7130E-02	30	3.7743E-02	Ehnes et al. 2011
2732	Invertebrates	Loricera pilicornis	1.7330E-02	25	1.2970E-02	Ehnes et al. 2011
2733	Invertebrates	Loricera pilicornis	1.7490E-02	10	2.0402E-03	Ehnes et al. 2011
2734	Invertebrates	Loricera pilicornis	1.7730E-02	15	7.2863E-03	Ehnes et al. 2011
2735	Invertebrates	Loricera pilicornis	1.7870E-02	30	2.6522E-02	Ehnes et al. 2011
2736	Invertebrates	Loricera pilicornis	1.8020E-02	5	2.0402E-03	Ehnes et al. 2011
2737	Invertebrates	Loricera pilicornis	1.8130E-02	30	1.8799E-02	Ehnes et al. 2011
2738	Invertebrates	Loricera pilicornis	1.8150E-02	5	1.8945E-03	Ehnes et al. 2011
2739	Invertebrates	Loricera pilicornis	1.8340E-02	30	2.3899E-02	Ehnes et al. 2011
2740	Invertebrates	Loricera pilicornis	1.9460E-02	5	3.0603E-03	Ehnes et al. 2011

2741	Invertebrates	Lucicutia bicornuta	1.0500E-02	5	2.5893E-04	Makarieva et al. 2008
2742	Invertebrates	Lucicutia flavicornis	1.2900E-04	20	1.3275E-04	Makarieva et al. 2008
2743	Invertebrates	Lucicutia maxima	1.0400E-02	10	6.4397E-04	Makarieva et al. 2008
2744	Invertebrates	Lucicutia maxima	1.1800E-02	5	3.6958E-04	Makarieva et al. 2008
2745	Invertebrates	Lucicutia maxima	1.4700E-02	1.5	3.8896E-04	Makarieva et al. 2008
2746	Invertebrates	Lucifer typus	4.6600E-04	17	5.2568E-04	Makarieva et al. 2008
2747	Invertebrates	Lumbricus castaneus	2.4000E-01	19	3.4850E-02	Ehnes et al. 2011
2748	Invertebrates	Lumbricus castaneus	1.5000E+00	11	1.0250E-01	Ehnes et al. 2011
2749	Invertebrates	Lumbricus castaneus	1.5000E+00	14	8.3500E-02	Ehnes et al. 2011
2750	Invertebrates	Lumbricus castaneus	1.5000E+00	17	1.1000E-01	Ehnes et al. 2011
2751	Invertebrates	Lumbricus castaneus	1.5000E+00	20	8.7500E-02	Ehnes et al. 2011
2752	Invertebrates	Lumbricus castaneus	1.5000E+00	23	1.0600E-01	Ehnes et al. 2011
2753	Invertebrates	Lumbricus castaneus	1.5000E+00	26	1.3600E-01	Ehnes et al. 2011
2754	Invertebrates	Lumbricus rubellus	7.4500E-01	19	7.3500E-02	Ehnes et al. 2011
2755	Invertebrates	Lumbricus terrestris	4.3360E-02	11.3	4.6632E-03	Ehnes et al. 2011
2756	Invertebrates	Lumbricus terrestris	6.1270E-02	11.3	5.9748E-03	Ehnes et al. 2011
2757	Invertebrates	Lumbricus terrestris	8.2900E-02	11.3	1.0784E-02	Ehnes et al. 2011
2758	Invertebrates	Lumbricus terrestris	8.3300E-02	11.3	5.6833E-03	Ehnes et al. 2011
2759	Invertebrates	Lumbricus terrestris	8.9300E-02	10.6	6.1205E-03	Ehnes et al. 2011
2760	Invertebrates	Lumbricus terrestris	1.7400E-01	10.6	9.4722E-03	Ehnes et al. 2011
2761	Invertebrates	Lumbricus terrestris	1.9160E-01	5	1.1950E-02	Ehnes et al. 2011
2762	Invertebrates	Lumbricus terrestris	2.3614E-01	5	1.1658E-02	Ehnes et al. 2011
2763	Invertebrates	Lumbricus terrestris	2.6158E-01	10.6	1.1950E-02	Ehnes et al. 2011
2764	Invertebrates	Lumbricus terrestris	3.2496E-01	5	1.5884E-02	Ehnes et al. 2011
2765	Invertebrates	Lumbricus terrestris	3.4575E-01	10.6	1.6321E-02	Ehnes et al. 2011
2766	Invertebrates	Lumbricus terrestris	3.7115E-01	11.3	1.9964E-02	Ehnes et al. 2011

2767	Invertebrates	Lumbricus terrestris	3.8664E-01	10.6	1.5593E-02	Ehnes et al. 2011
2768	Invertebrates	Lumbricus terrestris	3.8715E-01	10.6	1.7779E-02	Ehnes et al. 2011
2769	Invertebrates	Lumbricus terrestris	4.0970E-01	11.3	2.8854E-02	Ehnes et al. 2011
2770	Invertebrates	Lumbricus terrestris	4.4937E-01	5	1.0784E-02	Ehnes et al. 2011
2771	Invertebrates	Lumbricus terrestris	4.5454E-01	5	1.2532E-02	Ehnes et al. 2011
2772	Invertebrates	Lumbricus terrestris	4.7148E-01	5	1.2532E-02	Ehnes et al. 2011
2773	Invertebrates	Lumbricus terrestris	4.8102E-01	11.3	2.1422E-02	Ehnes et al. 2011
2774	Invertebrates	Lumbricus terrestris	5.5133E-01	5	1.6613E-02	Ehnes et al. 2011
2775	Invertebrates	Lumbricus terrestris	5.7702E-01	5	1.6904E-02	Ehnes et al. 2011
2776	Invertebrates	Lumbricus terrestris	5.7820E-01	5	1.5155E-02	Ehnes et al. 2011
2777	Invertebrates	Lumbricus terrestris	5.9280E-01	5	1.6613E-02	Ehnes et al. 2011
2778	Invertebrates	Lumbricus terrestris	5.9381E-01	11.3	2.2879E-02	Ehnes et al. 2011
2779	Invertebrates	Lumbricus terrestris	6.0640E-01	5	1.9090E-02	Ehnes et al. 2011
2780	Invertebrates	Lumbricus terrestris	6.8758E-01	11.3	2.8708E-02	Ehnes et al. 2011
2781	Invertebrates	Lumbricus terrestris	7.8180E-01	11.3	3.0457E-02	Ehnes et al. 2011
2782	Invertebrates	Lumbricus terrestris	1.6328E+00	20	1.5053E-01	Ehnes et al. 2011
2783	Invertebrates	Lumbricus terrestris	2.1762E+00	20	1.6146E-01	Ehnes et al. 2011
2784	Invertebrates	Lumbricus terrestris	2.4598E+00	15	1.4893E-01	Ehnes et al. 2011
2785	Invertebrates	Lumbricus terrestris	2.4680E+00	5	6.7471E-02	Ehnes et al. 2011
2786	Invertebrates	Lumbricus terrestris	2.5752E+00	5	9.8219E-02	Ehnes et al. 2011
2787	Invertebrates	Lumbricus terrestris	2.6000E+00	19	1.6600E-01	Ehnes et al. 2011
2788	Invertebrates	Lumbricus terrestris	2.6441E+00	14.6	1.0769E-01	Ehnes et al. 2011
2789	Invertebrates	Lumbricus terrestris	2.6528E+00	5	8.4667E-02	Ehnes et al. 2011
2790	Invertebrates	Lumbricus terrestris	2.6821E+00	10	1.0988E-01	Ehnes et al. 2011
2791	Invertebrates	Lumbricus terrestris	3.1015E+00	5	9.1370E-02	Ehnes et al. 2011
2792	Invertebrates	Lumbricus terrestris	3.4411E+00	10	9.0204E-02	Ehnes et al. 2011

2793	Invertebrates	Lumbricus terrestris	3.5604E+00	20	1.1789E-01	Ehnes et al. 2011
2794	Invertebrates	Lumbricus terrestris	3.5751E+00	10	1.4558E-01	Ehnes et al. 2011
2795	Invertebrates	Lumbricus terrestris	3.8074E+00	25	2.1422E-01	Ehnes et al. 2011
2796	Invertebrates	Lumbricus terrestris	3.8247E+00	20	1.2751E-01	Ehnes et al. 2011
2797	Invertebrates	Lumbricus terrestris	4.0131E+00	5	1.0070E-01	Ehnes et al. 2011
2798	Invertebrates	Lumbricus terrestris	4.0547E+00	15	1.6205E-01	Ehnes et al. 2011
2799	Invertebrates	Lumbricus terrestris	4.0860E+00	20	1.4660E-01	Ehnes et al. 2011
2800	Invertebrates	Lumbricus terrestris	4.2171E+00	26.3	2.2471E-01	Ehnes et al. 2011
2801	Invertebrates	Lumbricus terrestris	4.3000E+00	5	1.2200E-01	Ehnes et al. 2011
2802	Invertebrates	Lumbricus terrestris	4.3000E+00	10	1.6400E-01	Ehnes et al. 2011
2803	Invertebrates	Lumbricus terrestris	4.3000E+00	15	1.8850E-01	Ehnes et al. 2011
2804	Invertebrates	Lumbricus terrestris	4.3000E+00	20	2.8050E-01	Ehnes et al. 2011
2805	Invertebrates	Lumbricus terrestris	4.3007E+00	10	1.1381E-01	Ehnes et al. 2011
2806	Invertebrates	Lumbricus terrestris	4.4112E+00	15	1.2401E-01	Ehnes et al. 2011
2807	Invertebrates	Lumbricus terrestris	4.4166E+00	26.3	2.0489E-01	Ehnes et al. 2011
2808	Invertebrates	Lumbricus terrestris	4.4416E+00	25	2.0780E-01	Ehnes et al. 2011
2809	Invertebrates	Lumbricus terrestris	4.6533E+00	20	3.4974E-01	Ehnes et al. 2011
2810	Invertebrates	Lumbricus terrestris	4.6724E+00	5	4.2406E-02	Ehnes et al. 2011
2811	Invertebrates	Lumbricus terrestris	4.7181E+00	10	1.2489E-01	Ehnes et al. 2011
2812	Invertebrates	Lumbricus terrestris	4.7664E+00	25	2.2806E-01	Ehnes et al. 2011
2813	Invertebrates	Lumbricus terrestris	4.8378E+00	20	1.7414E-01	Ehnes et al. 2011
2814	Invertebrates	Lumbricus terrestris	4.8410E+00	15	1.1935E-01	Ehnes et al. 2011
2815	Invertebrates	Lumbricus terrestris	4.9953E+00	24.8	2.2864E-01	Ehnes et al. 2011
2816	Invertebrates	Lumbricus terrestris	5.1911E+00	15	1.6875E-01	Ehnes et al. 2011
2817	Invertebrates	Lumbricus terrestris	5.2456E+00	5	1.0274E-01	Ehnes et al. 2011
2818	Invertebrates	Lumbricus terrestris	5.2542E+00	25	3.1724E-01	Ehnes et al. 2011

2819	Invertebrates	Lumbricus terrestris	5.3944E+00	10	1.1687E-01	Ehnes et al. 2011
2820	Invertebrates	Lumbricus terrestris	5.5374E+00	10	1.1352E-01	Ehnes et al. 2011
2821	Invertebrates	Lumbricus terrestris	5.7500E+00	26.3	3.3517E-01	Ehnes et al. 2011
2822	Invertebrates	Lumbricus terrestris	5.8083E+00	15	1.5170E-01	Ehnes et al. 2011
2823	Invertebrates	Lumbricus terrestris	5.9072E+00	25	2.2937E-01	Ehnes et al. 2011
2824	Invertebrates	Lumbricus terrestris	6.2341E+00	20	2.0270E-01	Ehnes et al. 2011
2825	Invertebrates	Lumbricus terrestris	6.2376E+00	5	9.6616E-02	Ehnes et al. 2011
2826	Invertebrates	Lumbricus terrestris	6.2777E+00	5	1.0070E-01	Ehnes et al. 2011
2827	Invertebrates	Lumbricus terrestris	6.5089E+00	25	2.2937E-01	Ehnes et al. 2011
2828	Invertebrates	Lumbricus terrestris	6.6100E+00	10	1.1323E-01	Ehnes et al. 2011
2829	Invertebrates	Lycosa godeffroyi	2.0000E-01	20	2.8350E-02	Ehnes et al. 2011
2830	Invertebrates	Lycosa godeffroyi	2.0000E-01	30	4.3500E-02	Ehnes et al. 2011
2831	Invertebrates	Lycosa godeffroyi	2.0000E-01	40	6.6500E-02	Ehnes et al. 2011
2832	Invertebrates	Lycosa godeffroyi	2.0000E-01	30	6.4000E-02	Ehnes et al. 2011
2833	Invertebrates	Lycosa godeffroyi	2.0000E-01	4	7.4500E-03	Ehnes et al. 2011
2834	Invertebrates	Lycosa godeffroyi	2.0000E-01	20	5.1000E-02	Ehnes et al. 2011
2835	Invertebrates	Lycosa godeffroyi	2.0000E-01	35	3.0300E-02	Ehnes et al. 2011
2836	Invertebrates	Lycosa godeffroyi	2.0000E-01	10	1.1800E-02	Ehnes et al. 2011
2837	Invertebrates	Lycosa sp	1.2300E-01	19	2.1600E-02	Ehnes et al. 2011
2838	Invertebrates	Lycosa sp	2.0000E-01	20	5.0500E-02	Ehnes et al. 2011
2839	Invertebrates	Lycosidae	1.9800E-01	20	5.2000E-02	Ehnes et al. 2011
2840	Invertebrates	Lycosidae	2.2600E-01	15	4.3150E-02	Ehnes et al. 2011
2841	Invertebrates	Lycosidae	2.3100E-01	25	7.1500E-02	Ehnes et al. 2011
2842	Invertebrates	Lygus kalmii	1.0170E-02	25	2.6334E-02	Ehnes et al. 2011
2843	Invertebrates	Lyniphiidae	7.2000E-04	15	4.9545E-04	Ehnes et al. 2011
2844	Invertebrates	Lysmata sp.	1.0900E-02	30	1.4062E-02	Makarieva et al. 2008

2845	Invertebrates	Macrobrachium acanthurus	1.7000E+00	20	2.8152E-01	Makarieva et al. 2008
2846	Invertebrates	Macrobrachium heterochirus	2.6000E+00	20	4.1652E-01	Makarieva et al. 2008
2847	Invertebrates	Macrobrachium olfersi	1.2000E+00	20	1.2960E-01	Makarieva et al. 2008
2848	Invertebrates	Macrobrachium potiuna	6.8000E-01	20	5.9976E-02	Makarieva et al. 2008
2849	Invertebrates	Macrodontia dejeani	4.8600E+00	25	1.4039E+00	Ehnes et al. 2011
2850	Invertebrates	Macrotermes carbonarius	1.1930E-02	25	9.3240E-03	Ehnes et al. 2011
2851	Invertebrates	Macrotermes gilvus	7.0000E-03	25	9.9000E-04	Ehnes et al. 2011
2852	Invertebrates	Macrotermes malaccensis	1.1600E-02	25	2.5380E-03	Ehnes et al. 2011
2853	Invertebrates	Madoryx oeclus	1.6990E+00	25	9.6638E-01	Ehnes et al. 2011
2854	Invertebrates	Maja squinado	5.9100E+02	10	2.3404E+00	Makarieva et al. 2008
2855	Invertebrates	Mamestra configurata	1.3905E-01	25	8.3754E-02	Ehnes et al. 2011
2856	Invertebrates	Manduca corallina	1.6183E+00	25	5.8304E-01	Ehnes et al. 2011
2857	Invertebrates	Manduca lefeburei	5.7100E-01	25	1.6650E-01	Ehnes et al. 2011
2858	Invertebrates	Manduca rustica	2.8100E+00	25	1.2069E+00	Ehnes et al. 2011
2859	Invertebrates	Marpissa bina	1.6800E-01	20	2.6250E-02	Ehnes et al. 2011
2860	Invertebrates	Marpissa muscosa	2.7500E-02	20	3.9600E-03	Ehnes et al. 2011
2861	Invertebrates	Marpissa muscosa	3.9100E-02	20	5.5000E-03	Ehnes et al. 2011
2862	Invertebrates	Megacalanus princeps	5.5600E-02	5	2.0516E-03	Makarieva et al. 2008
2863	Invertebrates	Megacalanus sp. A	3.2600E-02	10	2.7404E-03	Makarieva et al. 2008
2864	Invertebrates	Megacalanus sp. A	3.4500E-02	5	1.1551E-03	Makarieva et al. 2008
2865	Invertebrates	Megacalanus sp. A	3.4500E-02	1.5	1.3041E-03	Makarieva et al. 2008
2866	Invertebrates	Megacalanus sp. A	3.4900E-02	5	1.3820E-03	Makarieva et al. 2008
2867	Invertebrates	Megacalanus sp. A	3.5600E-02	1.5	1.1534E-03	Makarieva et al. 2008
2868	Invertebrates	Megacalanus sp. B	3.7200E-02	5	1.0714E-03	Makarieva et al. 2008
2869	Invertebrates	Megalocranchia sp.	4.7900E+01	5	4.1817E-01	Makarieva et al. 2008
2870	Invertebrates	Megalpyge sp.	6.2700E-01	25	2.8078E-01	Ehnes et al. 2011

2871	Invertebrates	Megascolex mauritii	1.5000E-01	15	7.0229E-03	Ehnes et al. 2011
2872	Invertebrates	Megascolex mauritii	1.5000E-01	20	1.4046E-02	Ehnes et al. 2011
2873	Invertebrates	Megascolex mauritii	1.5000E-01	25	2.5283E-02	Ehnes et al. 2011
2874	Invertebrates	Megascolex mauritii	1.5000E-01	30	3.5115E-02	Ehnes et al. 2011
2875	Invertebrates	Megascolex mauritii	1.5000E-01	35	4.9160E-02	Ehnes et al. 2011
2876	Invertebrates	Megascolex mauritii	2.5000E-01	15	1.1237E-02	Ehnes et al. 2011
2877	Invertebrates	Megascolex mauritii	2.5000E-01	20	2.1069E-02	Ehnes et al. 2011
2878	Invertebrates	Megascolex mauritii	2.5000E-01	25	3.1603E-02	Ehnes et al. 2011
2879	Invertebrates	Megascolex mauritii	2.5000E-01	30	3.5115E-02	Ehnes et al. 2011
2880	Invertebrates	Megascolex mauritii	2.5000E-01	35	4.9160E-02	Ehnes et al. 2011
2881	Invertebrates	Megascolex mauritii	3.0000E-01	15	2.1069E-02	Ehnes et al. 2011
2882	Invertebrates	Megascolex mauritii	3.0000E-01	20	3.8626E-02	Ehnes et al. 2011
2883	Invertebrates	Megascolex mauritii	4.0000E-01	15	2.1069E-02	Ehnes et al. 2011
2884	Invertebrates	Megascolex mauritii	4.0000E-01	20	4.2138E-02	Ehnes et al. 2011
2885	Invertebrates	Megascolex mauritii	4.0000E-01	25	4.2138E-02	Ehnes et al. 2011
2886	Invertebrates	Megascolex mauritii	4.0000E-01	30	5.3374E-02	Ehnes et al. 2011
2887	Invertebrates	Megascolex mauritii	4.0000E-01	35	6.3206E-02	Ehnes et al. 2011
2888	Invertebrates	Megascolex mauritii	5.0000E-01	15	3.2305E-02	Ehnes et al. 2011
2889	Invertebrates	Megascolex mauritii	5.0000E-01	20	3.9328E-02	Ehnes et al. 2011
2890	Invertebrates	Megascolex mauritii	5.0000E-01	25	4.9160E-02	Ehnes et al. 2011
2891	Invertebrates	Megascolex mauritii	5.0000E-01	30	6.3206E-02	Ehnes et al. 2011
2892	Invertebrates	Megascolex mauritii	5.0000E-01	35	9.1298E-02	Ehnes et al. 2011
2893	Invertebrates	Megascolex mauritii	6.0000E-01	15	3.8626E-02	Ehnes et al. 2011
2894	Invertebrates	Megascolex mauritii	6.0000E-01	15	2.1069E-02	Ehnes et al. 2011
2895	Invertebrates	Megascolex mauritii	6.0000E-01	20	5.6183E-02	Ehnes et al. 2011
2896	Invertebrates	Megascolex mauritii	6.0000E-01	20	5.3374E-02	Ehnes et al. 2011

2897	Invertebrates	Megascolex mauritii	6.0000E-01	25	6.7420E-02	Ehnes et al. 2011
2898	Invertebrates	Megascolex mauritii	6.0000E-01	25	7.0931E-02	Ehnes et al. 2011
2899	Invertebrates	Megascolex mauritii	6.0000E-01	30	7.0229E-02	Ehnes et al. 2011
2900	Invertebrates	Megascolex mauritii	6.0000E-01	30	9.1298E-02	Ehnes et al. 2011
2901	Invertebrates	Megascolex mauritii	6.0000E-01	35	8.4275E-02	Ehnes et al. 2011
2902	Invertebrates	Megascolex mauritii	6.0000E-01	35	1.2641E-01	Ehnes et al. 2011
2903	Invertebrates	Megascolex mauritii	7.0000E-01	15	4.2138E-02	Ehnes et al. 2011
2904	Invertebrates	Megascolex mauritii	7.0000E-01	20	6.3206E-02	Ehnes et al. 2011
2905	Invertebrates	Megascolex mauritii	7.0000E-01	25	7.0229E-02	Ehnes et al. 2011
2906	Invertebrates	Megascolex mauritii	7.0000E-01	30	8.4275E-02	Ehnes et al. 2011
2907	Invertebrates	Megascolex mauritii	7.0000E-01	35	1.0324E-01	Ehnes et al. 2011
2908	Invertebrates	Megascolex mauritii	8.0000E-01	15	4.2138E-02	Ehnes et al. 2011
2909	Invertebrates	Megascolex mauritii	8.0000E-01	15	3.5115E-02	Ehnes et al. 2011
2910	Invertebrates	Megascolex mauritii	8.0000E-01	20	7.0229E-02	Ehnes et al. 2011
2911	Invertebrates	Megascolex mauritii	8.0000E-01	20	4.9160E-02	Ehnes et al. 2011
2912	Invertebrates	Megascolex mauritii	8.0000E-01	25	8.0764E-02	Ehnes et al. 2011
2913	Invertebrates	Megascolex mauritii	8.0000E-01	30	7.0229E-02	Ehnes et al. 2011
2914	Invertebrates	Megascolex mauritii	8.0000E-01	35	7.7252E-02	Ehnes et al. 2011
2915	Invertebrates	Megascolex mauritii	9.0000E-01	15	4.9160E-02	Ehnes et al. 2011
2916	Invertebrates	Megascolex mauritii	9.0000E-01	20	6.3206E-02	Ehnes et al. 2011
2917	Invertebrates	Megascolex mauritii	9.0000E-01	25	6.3206E-02	Ehnes et al. 2011
2918	Invertebrates	Megascolex mauritii	9.0000E-01	30	9.1298E-02	Ehnes et al. 2011
2919	Invertebrates	Megascolex mauritii	9.0000E-01	35	1.0183E-01	Ehnes et al. 2011
2920	Invertebrates	Megascolex mauritii	1.1000E+00	15	6.3206E-02	Ehnes et al. 2011
2921	Invertebrates	Megascolex mauritii	1.1000E+00	20	7.7252E-02	Ehnes et al. 2011
2922	Invertebrates	Megascolex mauritii	1.1000E+00	25	9.1298E-02	Ehnes et al. 2011

2923	Invertebrates	Megascolex mauritii	1.1000E+00	30	1.0534E-01	Ehnes et al. 2011
2924	Invertebrates	Megascolex mauritii	1.1000E+00	35	1.6153E-01	Ehnes et al. 2011
2925	Invertebrates	Megascolex mauritii	1.2000E+00	15	4.2138E-02	Ehnes et al. 2011
2926	Invertebrates	Megascolex mauritii	1.2000E+00	20	8.4275E-02	Ehnes et al. 2011
2927	Invertebrates	Megascolex mauritii	1.2000E+00	25	9.8321E-02	Ehnes et al. 2011
2928	Invertebrates	Megascolex mauritii	1.2000E+00	30	1.1237E-01	Ehnes et al. 2011
2929	Invertebrates	Megascolex mauritii	1.2000E+00	35	1.0886E-01	Ehnes et al. 2011
2930	Invertebrates	Megascolex mauritii	1.3000E+00	15	6.3206E-02	Ehnes et al. 2011
2931	Invertebrates	Megascolex mauritii	1.3000E+00	15	4.2138E-02	Ehnes et al. 2011
2932	Invertebrates	Megascolex mauritii	1.3000E+00	20	8.4275E-02	Ehnes et al. 2011
2933	Invertebrates	Megascolex mauritii	1.3000E+00	20	7.7252E-02	Ehnes et al. 2011
2934	Invertebrates	Megascolex mauritii	1.3000E+00	25	9.1298E-02	Ehnes et al. 2011
2935	Invertebrates	Megascolex mauritii	1.3000E+00	25	9.4809E-02	Ehnes et al. 2011
2936	Invertebrates	Megascolex mauritii	1.3000E+00	30	1.2641E-01	Ehnes et al. 2011
2937	Invertebrates	Megascolex mauritii	1.3000E+00	30	1.4748E-01	Ehnes et al. 2011
2938	Invertebrates	Megascolex mauritii	1.3000E+00	35	1.9664E-01	Ehnes et al. 2011
2939	Invertebrates	Megascolex mauritii	1.3000E+00	35	1.8611E-01	Ehnes et al. 2011
2940	Invertebrates	Megascolex mauritii	1.4000E+00	15	6.3206E-02	Ehnes et al. 2011
2941	Invertebrates	Megascolex mauritii	1.4000E+00	20	8.4275E-02	Ehnes et al. 2011
2942	Invertebrates	Megascolex mauritii	1.4000E+00	25	9.1298E-02	Ehnes et al. 2011
2943	Invertebrates	Megascolex mauritii	1.4000E+00	30	1.1237E-01	Ehnes et al. 2011
2944	Invertebrates	Megascolex mauritii	1.4000E+00	35	1.6855E-01	Ehnes et al. 2011
2945	Invertebrates	Megascolex mauritii	1.5000E+00	15	5.6183E-02	Ehnes et al. 2011
2946	Invertebrates	Megascolex mauritii	1.5000E+00	20	9.1298E-02	Ehnes et al. 2011
2947	Invertebrates	Megascolex mauritii	1.5000E+00	25	1.0534E-01	Ehnes et al. 2011
2948	Invertebrates	Megascolex mauritii	1.5000E+00	30	1.1939E-01	Ehnes et al. 2011

2949	Invertebrates	Megascolex mauritii	1.5000E+00	35	1.2641E-01	Ehnes et al. 2011
2950	Invertebrates	Melanoplus bivittatus	1.6500E+00	25	9.1026E-01	Ehnes et al. 2011
2951	Invertebrates	Melanoplus complanipes	1.4155E-01	25	1.1237E-01	Ehnes et al. 2011
2952	Invertebrates	Meloid sp.	1.7340E-02	25	7.3206E-02	Ehnes et al. 2011
2953	Invertebrates	Melyrid sp.	1.1600E-03	25	3.7620E-03	Ehnes et al. 2011
2954	Invertebrates	Menemerus bivittatus	2.7900E-02	22	1.0200E-02	Ehnes et al. 2011
2955	Invertebrates	Menippe mercenaria	3.0300E-04	20	2.5346E-04	Makarieva et al. 2008
2956	Invertebrates	Menippe mercenaria	2.4200E-02	20	9.9265E-03	Makarieva et al. 2008
2957	Invertebrates	Menippe mercenaria	3.1600E+01	20	6.9678E-01	Makarieva et al. 2008
2958	Invertebrates	Mesidothea entomon	5.0000E-01	20	1.3733E-01	Makarieva et al. 2008
2959	Invertebrates	Mesidothea entomon	2.0000E+00	20	4.2916E-01	Makarieva et al. 2008
2960	Invertebrates	Mesidothea entomon	3.2000E+00	20	6.5940E-01	Makarieva et al. 2008
2961	Invertebrates	Mesidothea entomon	5.0000E+00	20	9.2295E-01	Makarieva et al. 2008
2962	Invertebrates	Mesidothea entomon	1.0000E+01	20	1.6400E+00	Makarieva et al. 2008
2963	Invertebrates	Mesocalanus tenuicornis	1.5500E-04	15.8	2.2010E-04	Makarieva et al. 2008
2964	Invertebrates	Mesopodopsis slabberi	3.2500E-02	20	5.9980E-03	Makarieva et al. 2008
2965	Invertebrates	Messor capensis	3.1400E-03	25	2.9160E-03	Ehnes et al. 2011
2966	Invertebrates	Messor capensis	3.5000E-03	20	1.9440E-03	Ehnes et al. 2011
2967	Invertebrates	Messor capensis	4.5000E-03	20	6.6600E-04	Ehnes et al. 2011
2968	Invertebrates	Messor capensis	4.8000E-03	20	2.0340E-03	Ehnes et al. 2011
2969	Invertebrates	Messor capensis	5.9000E-03	20	1.5300E-03	Ehnes et al. 2011
2970	Invertebrates	Messor capensis	6.5000E-03	20	5.5260E-03	Ehnes et al. 2011
2971	Invertebrates	Messor capensis	6.6000E-03	20	1.1520E-03	Ehnes et al. 2011
2972	Invertebrates	Messor capensis	6.6000E-03	20	2.8440E-03	Ehnes et al. 2011
2973	Invertebrates	Messor capensis	7.1000E-03	20	9.1800E-04	Ehnes et al. 2011
2974	Invertebrates	Messor capensis	7.6000E-03	20	3.9600E-04	Ehnes et al. 2011

2975	Invertebrates	Messor capensis	8.1000E-03	20	7.9200E-04	Ehnes et al. 2011
2976	Invertebrates	Messor capensis	8.4000E-03	20	7.2000E-04	Ehnes et al. 2011
2977	Invertebrates	Messor capensis	9.2000E-03	20	5.1840E-03	Ehnes et al. 2011
2978	Invertebrates	Messor capensis	9.5000E-03	20	4.6440E-03	Ehnes et al. 2011
2979	Invertebrates	Messor capensis	9.9000E-03	20	1.1340E-03	Ehnes et al. 2011
2980	Invertebrates	Messor capensis	9.9000E-03	20	2.1960E-03	Ehnes et al. 2011
2981	Invertebrates	Messor capensis	9.9000E-03	20	2.8080E-03	Ehnes et al. 2011
2982	Invertebrates	Messor capensis	9.9000E-03	20	2.7540E-03	Ehnes et al. 2011
2983	Invertebrates	Messor capensis	1.0400E-02	20	1.7460E-03	Ehnes et al. 2011
2984	Invertebrates	Messor capensis	1.0500E-02	20	8.6400E-04	Ehnes et al. 2011
2985	Invertebrates	Messor capensis	1.0700E-02	20	1.2600E-03	Ehnes et al. 2011
2986	Invertebrates	Messor capensis	1.0700E-02	20	9.5220E-03	Ehnes et al. 2011
2987	Invertebrates	Messor capensis	1.0900E-02	20	9.1800E-04	Ehnes et al. 2011
2988	Invertebrates	Messor capensis	1.1000E-02	20	5.9220E-03	Ehnes et al. 2011
2989	Invertebrates	Messor capensis	1.1000E-02	20	2.8260E-03	Ehnes et al. 2011
2990	Invertebrates	Messor capensis	1.1400E-02	20	1.0080E-03	Ehnes et al. 2011
2991	Invertebrates	Messor capensis	1.1600E-02	20	3.9240E-03	Ehnes et al. 2011
2992	Invertebrates	Messor capensis	1.2100E-02	20	1.2780E-03	Ehnes et al. 2011
2993	Invertebrates	Messor capensis	1.2200E-02	20	4.8600E-03	Ehnes et al. 2011
2994	Invertebrates	Messor capensis	1.2300E-02	20	3.1320E-03	Ehnes et al. 2011
2995	Invertebrates	Messor capensis	1.2600E-02	20	7.7400E-04	Ehnes et al. 2011
2996	Invertebrates	Messor capensis	1.2900E-02	20	6.0120E-03	Ehnes et al. 2011
2997	Invertebrates	Messor capensis	1.3600E-02	20	6.0300E-03	Ehnes et al. 2011
2998	Invertebrates	Messor capensis	1.3600E-02	20	7.7040E-03	Ehnes et al. 2011
2999	Invertebrates	Messor capensis	1.3700E-02	20	1.9440E-03	Ehnes et al. 2011
3000	Invertebrates	Messor capensis	1.3700E-02	25	6.8940E-03	Ehnes et al. 2011

3001	Invertebrates	Messor capensis	1.4000E-02	20	9.7020E-03	Ehnes et al. 2011
3002	Invertebrates	Messor capensis	1.5800E-02	20	1.2492E-02	Ehnes et al. 2011
3003	Invertebrates	Messor capensis	1.6300E-02	20	1.9080E-03	Ehnes et al. 2011
3004	Invertebrates	Messor capensis	1.8400E-02	20	1.3878E-02	Ehnes et al. 2011
3005	Invertebrates	Messor capensis	1.9100E-02	20	2.6640E-03	Ehnes et al. 2011
3006	Invertebrates	Messor capensis	1.9500E-02	20	4.1580E-03	Ehnes et al. 2011
3007	Invertebrates	Messor capensis	1.9800E-02	20	7.8480E-03	Ehnes et al. 2011
3008	Invertebrates	Messor capensis	2.1400E-02	20	1.3356E-02	Ehnes et al. 2011
3009	Invertebrates	Messor capensis	2.3300E-02	20	4.3560E-03	Ehnes et al. 2011
3010	Invertebrates	Messor capensis	2.3600E-02	20	9.0540E-03	Ehnes et al. 2011
3011	Invertebrates	Messor capensis	2.4800E-02	20	1.0962E-02	Ehnes et al. 2011
3012	Invertebrates	Messor capensis	2.5500E-02	20	6.6600E-03	Ehnes et al. 2011
3013	Invertebrates	Messor capensis	2.7500E-02	20	6.9840E-03	Ehnes et al. 2011
3014	Invertebrates	Messor capensis	3.2200E-02	20	1.4418E-02	Ehnes et al. 2011
3015	Invertebrates	Messor capensis	4.3400E-02	20	2.8224E-02	Ehnes et al. 2011
3016	Invertebrates	Messor julianus	5.0900E-03	25	1.2780E-03	Ehnes et al. 2011
3017	Invertebrates	Messor pergandei	1.3300E-03	30	1.3500E-03	Ehnes et al. 2011
3018	Invertebrates	Messor pergandei	1.5500E-03	30	1.6380E-03	Ehnes et al. 2011
3019	Invertebrates	Messor pergandei	1.7100E-03	30	1.8540E-03	Ehnes et al. 2011
3020	Invertebrates	Messor pergandei	2.4400E-03	30	1.8000E-03	Ehnes et al. 2011
3021	Invertebrates	Messor pergandei	2.5400E-03	30	2.5380E-03	Ehnes et al. 2011
3022	Invertebrates	Messor pergandei	2.7700E-03	30	2.7720E-03	Ehnes et al. 2011
3023	Invertebrates	Messor pergandei	3.1600E-03	30	2.2680E-03	Ehnes et al. 2011
3024	Invertebrates	Messor pergandei	3.1800E-03	30	2.4300E-03	Ehnes et al. 2011
3025	Invertebrates	Messor pergandei	3.2900E-03	30	2.3760E-03	Ehnes et al. 2011
3026	Invertebrates	Messor pergandei	3.8300E-03	30	2.3400E-03	Ehnes et al. 2011

3027	Invertebrates	Messor pergandei	5.2700E-03	30	4.2840E-03	Ehnes et al. 2011
3028	Invertebrates	Messor pergandei	5.2700E-03	30	4.2480E-03	Ehnes et al. 2011
3029	Invertebrates	Messor pergandei	6.1000E-03	30	3.5100E-03	Ehnes et al. 2011
3030	Invertebrates	Messor pergandei	6.2400E-03	30	3.6180E-03	Ehnes et al. 2011
3031	Invertebrates	Messor pergandei	6.2600E-03	30	3.0960E-03	Ehnes et al. 2011
3032	Invertebrates	Messor pergandei	7.1900E-03	25	2.5200E-03	Ehnes et al. 2011
3033	Invertebrates	Messor pergandei	7.6100E-03	30	3.2580E-03	Ehnes et al. 2011
3034	Invertebrates	Messor pergandei	7.8800E-03	30	3.5460E-03	Ehnes et al. 2011
3035	Invertebrates	Messor pergandei	8.4100E-03	30	5.9940E-03	Ehnes et al. 2011
3036	Invertebrates	Messor pergandei	8.6300E-03	30	5.1660E-03	Ehnes et al. 2011
3037	Invertebrates	Messor pergandei	8.7000E-03	30	3.7620E-03	Ehnes et al. 2011
3038	Invertebrates	Messor pergandei	9.7000E-03	30	5.7420E-03	Ehnes et al. 2011
3039	Invertebrates	Metabelba papillipes	1.9000E-05	10	4.9500E-06	Ehnes et al. 2011
3040	Invertebrates	Metapenaeus pruinosus	1.8400E-01	20	6.1471E-02	Makarieva et al. 2008
3041	Invertebrates	Metopsilus porcellus	2.8500E-01	25	2.4512E-01	Ehnes et al. 2011
3042	Invertebrates	Metridia gerlachei	1.1000E-03	-1	2.1780E-04	Makarieva et al. 2008
3043	Invertebrates	Metridia gerlachei	1.3000E-03	-1.4	3.1356E-04	Makarieva et al. 2008
3044	Invertebrates	Metridia gerlachei	1.4090E-03	0	1.0424E-04	Makarieva et al. 2008
3045	Invertebrates	Metridia longa	1.5500E-03	0.1	4.0734E-04	Makarieva et al. 2008
3046	Invertebrates	Metridia pacifica	4.7000E-04	13	2.7410E-04	Makarieva et al. 2008
3047	Invertebrates	Metridia pacifica	7.4000E-04	8.2	1.7849E-04	Makarieva et al. 2008
3048	Invertebrates	Metridia princeps	1.1100E-02	5	4.0360E-04	Makarieva et al. 2008
3049	Invertebrates	Metridia princeps	1.2500E-02	1.5	5.7375E-04	Makarieva et al. 2008
3050	Invertebrates	Miathyria marcella	1.7100E-02	25	1.3786E-01	Ehnes et al. 2011
3051	Invertebrates	Microcerotermes dubius	2.2000E-03	25	5.2200E-04	Ehnes et al. 2011
3052	Invertebrates	Microcerotermes serrula	1.9000E-03	25	3.9600E-04	Ehnes et al. 2011

3053	Invertebrates	Microcerus sp.	1.3200E-01	25	3.4128E-02	Ehnes et al. 2011
3054	Invertebrates	Miloderes sp.	3.4000E-03	25	1.1826E-02	Ehnes et al. 2011
3055	Invertebrates	Mimas tiliae	3.2000E-01	25	3.0002E-01	Ehnes et al. 2011
3056	Invertebrates	Misumenoides formosipes	4.8700E-02	22	1.7350E-02	Ehnes et al. 2011
3057	Invertebrates	Misumenoides formosipes	8.2500E-02	20	1.3750E-02	Ehnes et al. 2011
3058	Invertebrates	Misumenops celer	4.0000E-02	20	7.2000E-03	Ehnes et al. 2011
3059	Invertebrates	Misumenops sp	3.4100E-02	22	5.9000E-03	Ehnes et al. 2011
3060	Invertebrates	Monoxia sp.	4.6300E-03	25	1.4256E-02	Ehnes et al. 2011
3061	Invertebrates	Multareoides bifurcatus	1.3400E-03	25	2.7360E-03	Ehnes et al. 2011
3062	Invertebrates	Munidopsis verrilli	5.6200E+00	2.5	3.0348E-02	Makarieva et al. 2008
3063	Invertebrates	Musca autumnalis	2.2000E-02	25	6.4944E-02	Ehnes et al. 2011
3064	Invertebrates	Musca domestica	1.8000E-02	25	1.0577E-01	Ehnes et al. 2011
3065	Invertebrates	Myrmica alaskensis	9.1000E-04	25	1.9260E-03	Ehnes et al. 2011
3066	Invertebrates	Myrmica rubra	2.7600E-03	25	1.2240E-03	Ehnes et al. 2011
3067	Invertebrates	Mysis relicta	1.0000E-02	20	4.0795E-03	Makarieva et al. 2008
3068	Invertebrates	Nanhermannia elegantula	1.8100E-05	5	8.5000E-07	Ehnes et al. 2011
3069	Invertebrates	Nanhermannia elegantula	1.8100E-05	10	2.1500E-06	Ehnes et al. 2011
3070	Invertebrates	Nanhermannia elegantula	1.8100E-05	15	3.4500E-06	Ehnes et al. 2011
3071	Invertebrates	Nannocalanus minor	2.0000E-04	26.9	2.0880E-04	Makarieva et al. 2008
3072	Invertebrates	Nanorchestes antarcticus	3.5000E-08	5	2.0000E-07	Ehnes et al. 2011
3073	Invertebrates	Nanorchestes antarcticus	1.6200E-07	5	5.0000E-08	Ehnes et al. 2011
3074	Invertebrates	Nanorchestes antarcticus	2.4700E-07	5	5.0000E-08	Ehnes et al. 2011
3075	Invertebrates	Nanorchestes antarcticus	9.2700E-07	5	5.0000E-08	Ehnes et al. 2011
3076	Invertebrates	Nanorchestes antarcticus	1.0550E-06	5	1.5000E-07	Ehnes et al. 2011
3077	Invertebrates	Nanorchestes antarcticus	1.3100E-06	5	8.0000E-07	Ehnes et al. 2011
3078	Invertebrates	Nanorchestes antarcticus	2.5150E-06	5	1.0000E-07	Ehnes et al. 2011

3079	Invertebrates	Nanorchestes antarcticus	2.6100E-06	5	4.0000E-07	Ehnes et al. 2011
3080	Invertebrates	Nanorchestes antarcticus	5.0900E-06	0	5.0000E-07	Ehnes et al. 2011
3081	Invertebrates	Nanorchestes antarcticus	5.0900E-06	5	7.0000E-07	Ehnes et al. 2011
3082	Invertebrates	Nanorchestes antarcticus	5.0900E-06	10	8.0000E-07	Ehnes et al. 2011
3083	Invertebrates	Nanorchestes antarcticus	6.0700E-06	5	1.4000E-06	Ehnes et al. 2011
3084	Invertebrates	Nanorchestes antarcticus	8.5000E-06	5	1.9000E-06	Ehnes et al. 2011
3085	Invertebrates	Nanorchestes antarcticus	8.5000E-06	10	3.1000E-06	Ehnes et al. 2011
3086	Invertebrates	Nanorchestes antarcticus	8.7480E-06	5	2.6000E-06	Ehnes et al. 2011
3087	Invertebrates	Nasutitermes longinasus	2.6000E-03	25	2.7000E-04	Ehnes et al. 2011
3088	Invertebrates	Nauphoeta cinerea	5.1000E-01	25	1.3180E-01	Ehnes et al. 2011
3089	Invertebrates	Nausitho rubra	2.6000E+00	5	1.2636E-02	Makarieva et al. 2008
3090	Invertebrates	Neanura muscorum	3.3000E-04	18	6.4000E-05	Ehnes et al. 2011
3091	Invertebrates	Nebria brevicollis	3.6440E-02	5	3.9346E-03	Ehnes et al. 2011
3092	Invertebrates	Nebria brevicollis	3.8970E-02	5	8.4521E-03	Ehnes et al. 2011
3093	Invertebrates	Nebria brevicollis	4.0700E-02	5	4.5175E-03	Ehnes et al. 2011
3094	Invertebrates	Nebria brevicollis	4.1240E-02	25	8.3064E-03	Ehnes et al. 2011
3095	Invertebrates	Nebria brevicollis	4.2760E-02	15	1.2970E-02	Ehnes et al. 2011
3096	Invertebrates	Nebria brevicollis	4.3250E-02	5	5.1004E-03	Ehnes et al. 2011
3097	Invertebrates	Nebria brevicollis	4.3700E-02	5	5.1004E-03	Ehnes et al. 2011
3098	Invertebrates	Nebria brevicollis	4.4330E-02	25	1.5155E-02	Ehnes et al. 2011
3099	Invertebrates	Nebria brevicollis	4.5260E-02	5	7.7235E-03	Ehnes et al. 2011
3100	Invertebrates	Nebria brevicollis	4.5520E-02	5	7.5777E-03	Ehnes et al. 2011
3101	Invertebrates	Nebria brevicollis	4.5610E-02	5	8.8893E-03	Ehnes et al. 2011
3102	Invertebrates	Nebria brevicollis	4.5800E-02	5	7.2863E-03	Ehnes et al. 2011
3103	Invertebrates	Nebria brevicollis	4.6840E-02	30	2.2005E-02	Ehnes et al. 2011
3104	Invertebrates	Nebria brevicollis	4.6950E-02	10	2.1859E-03	Ehnes et al. 2011

3105	Invertebrates	Nebria brevicollis	4.7210E-02	25	1.3698E-02	Ehnes et al. 2011
3106	Invertebrates	Nebria brevicollis	4.7730E-02	30	1.6176E-02	Ehnes et al. 2011
3107	Invertebrates	Nebria brevicollis	4.8060E-02	10	5.3919E-03	Ehnes et al. 2011
3108	Invertebrates	Nebria brevicollis	4.8270E-02	5	1.5301E-02	Ehnes et al. 2011
3109	Invertebrates	Nebria brevicollis	5.0480E-02	5	1.1075E-02	Ehnes et al. 2011
3110	Invertebrates	Nebria brevicollis	5.0500E-02	5	9.0350E-03	Ehnes et al. 2011
3111	Invertebrates	Nebria brevicollis	5.0560E-02	10	6.1205E-03	Ehnes et al. 2011
3112	Invertebrates	Nebria brevicollis	5.0780E-02	30	2.1422E-02	Ehnes et al. 2011
3113	Invertebrates	Nebria brevicollis	5.1950E-02	15	1.1075E-02	Ehnes et al. 2011
3114	Invertebrates	Nebria brevicollis	5.2020E-02	25	9.9094E-03	Ehnes et al. 2011
3115	Invertebrates	Nebria brevicollis	5.2490E-02	25	1.3261E-02	Ehnes et al. 2011
3116	Invertebrates	Nebria brevicollis	5.2490E-02	10	3.6432E-03	Ehnes et al. 2011
3117	Invertebrates	Nebria brevicollis	5.2600E-02	15	2.5065E-02	Ehnes et al. 2011
3118	Invertebrates	Nebria brevicollis	5.3490E-02	25	1.2824E-02	Ehnes et al. 2011
3119	Invertebrates	Nebria brevicollis	5.3840E-02	15	1.9964E-02	Ehnes et al. 2011
3120	Invertebrates	Nebria brevicollis	5.4400E-02	20	9.7636E-03	Ehnes et al. 2011
3121	Invertebrates	Nebria brevicollis	5.4650E-02	15	1.2824E-02	Ehnes et al. 2011
3122	Invertebrates	Nebria brevicollis	5.5240E-02	5	1.3698E-02	Ehnes et al. 2011
3123	Invertebrates	Nebria brevicollis	5.5310E-02	5	1.8945E-03	Ehnes et al. 2011
3124	Invertebrates	Nebria brevicollis	5.5490E-02	5	7.7235E-03	Ehnes et al. 2011
3125	Invertebrates	Nebria brevicollis	5.6280E-02	25	2.4045E-02	Ehnes et al. 2011
3126	Invertebrates	Nebria brevicollis	5.6370E-02	10	2.1859E-03	Ehnes et al. 2011
3127	Invertebrates	Nebria brevicollis	5.6480E-02	15	1.1221E-02	Ehnes et al. 2011
3128	Invertebrates	Nebria brevicollis	5.6890E-02	30	1.7633E-02	Ehnes et al. 2011
3129	Invertebrates	Nebria brevicollis	5.7000E-02	5	6.4119E-03	Ehnes et al. 2011
3130	Invertebrates	Nebria brevicollis	5.7400E-02	25	2.5793E-02	Ehnes et al. 2011

3131	Invertebrates	Nebria brevicollis	5.7880E-02	10	2.6231E-03	Ehnes et al. 2011
3132	Invertebrates	Nebria brevicollis	5.8110E-02	30	2.3170E-02	Ehnes et al. 2011
3133	Invertebrates	Nebria brevicollis	5.8930E-02	15	2.1276E-02	Ehnes et al. 2011
3134	Invertebrates	Nebria brevicollis	5.9200E-02	5	9.3265E-03	Ehnes et al. 2011
3135	Invertebrates	Nebria brevicollis	6.0000E-02	30	1.9527E-02	Ehnes et al. 2011
3136	Invertebrates	Nebria brevicollis	6.1930E-02	10	5.9748E-03	Ehnes et al. 2011
3137	Invertebrates	Nebria brevicollis	6.2290E-02	15	1.9819E-02	Ehnes et al. 2011
3138	Invertebrates	Nebria brevicollis	6.3770E-02	20	5.2461E-03	Ehnes et al. 2011
3139	Invertebrates	Nebria brevicollis	6.5290E-02	10	2.8854E-02	Ehnes et al. 2011
3140	Invertebrates	Nebria brevicollis	6.6410E-02	10	9.7636E-03	Ehnes et al. 2011
3141	Invertebrates	Nebria brevicollis	6.6420E-02	15	2.1276E-02	Ehnes et al. 2011
3142	Invertebrates	Nebria brevicollis	6.6480E-02	25	3.4100E-02	Ehnes et al. 2011
3143	Invertebrates	Nebria brevicollis	6.6550E-02	10	1.8944E-02	Ehnes et al. 2011
3144	Invertebrates	Nebria brevicollis	6.6920E-02	30	2.1859E-02	Ehnes et al. 2011
3145	Invertebrates	Nebria brevicollis	6.7230E-02	15	3.6577E-02	Ehnes et al. 2011
3146	Invertebrates	Nebria brevicollis	6.8000E-02	25	1.3552E-02	Ehnes et al. 2011
3147	Invertebrates	Nebria brevicollis	6.8010E-02	5	1.3698E-02	Ehnes et al. 2011
3148	Invertebrates	Nebria brevicollis	7.0610E-02	5	1.3261E-02	Ehnes et al. 2011
3149	Invertebrates	Nebria brevicollis	7.0860E-02	10	1.0055E-02	Ehnes et al. 2011
3150	Invertebrates	Nebria brevicollis	7.2210E-02	5	1.2095E-02	Ehnes et al. 2011
3151	Invertebrates	Nebria brevicollis	7.2820E-02	15	1.5738E-02	Ehnes et al. 2011
3152	Invertebrates	Nebria brevicollis	7.3180E-02	20	6.9948E-03	Ehnes et al. 2011
3153	Invertebrates	Nebria brevicollis	7.3190E-02	10	9.0350E-03	Ehnes et al. 2011
3154	Invertebrates	Nebria brevicollis	7.6860E-02	25	2.7688E-02	Ehnes et al. 2011
3155	Invertebrates	Nebria brevicollis	7.7630E-02	15	1.7050E-02	Ehnes et al. 2011
3156	Invertebrates	Nebria brevicollis	8.1350E-02	15	3.0602E-02	Ehnes et al. 2011

3157	Invertebrates	Nebria brevicollis	9.3450E-02	15	3.1185E-02	Ehnes et al. 2011
3158	Invertebrates	Nemobius silvestris	2.9700E-02	20.3	7.5848E-03	Ehnes et al. 2011
3159	Invertebrates	Nemobius silvestris	3.6400E-02	20	1.0956E-02	Ehnes et al. 2011
3160	Invertebrates	Nemobius silvestris	5.6400E-02	20.5	2.1069E-02	Ehnes et al. 2011
3161	Invertebrates	Nemobius silvestris	5.7200E-02	20.2	2.5704E-02	Ehnes et al. 2011
3162	Invertebrates	Nemobius silvestris	5.7800E-02	20.4	1.6855E-02	Ehnes et al. 2011
3163	Invertebrates	Nemobius silvestris	6.2300E-02	20	2.2333E-02	Ehnes et al. 2011
3164	Invertebrates	Nemobius silvestris	6.4200E-02	20.5	2.0788E-02	Ehnes et al. 2011
3165	Invertebrates	Nemobius silvestris	6.5800E-02	22	2.3737E-02	Ehnes et al. 2011
3166	Invertebrates	Nemobius silvestris	6.6500E-02	20	1.9805E-02	Ehnes et al. 2011
3167	Invertebrates	Nemobius silvestris	6.8000E-02	19	1.9805E-02	Ehnes et al. 2011
3168	Invertebrates	Nemobius silvestris	7.4600E-02	20.2	2.6547E-02	Ehnes et al. 2011
3169	Invertebrates	Nemobius silvestris	7.8600E-02	20.4	3.0058E-02	Ehnes et al. 2011
3170	Invertebrates	Nemobius silvestris	8.0800E-02	20.8	4.0873E-02	Ehnes et al. 2011
3171	Invertebrates	Neocalanus cristatus	8.0000E-03	6.3	1.6848E-03	Makarieva et al. 2008
3172	Invertebrates	Neocalanus cristatus	9.0000E-03	2	7.3872E-04	Makarieva et al. 2008
3173	Invertebrates	Neocalanus cristatus	1.5300E-02	2	6.7198E-04	Makarieva et al. 2008
3174	Invertebrates	Neocalanus cristatus	1.6600E-02	6	9.8006E-04	Makarieva et al. 2008
3175	Invertebrates	Neocalanus cristatus	1.7200E-02	2	3.6223E-04	Makarieva et al. 2008
3176	Invertebrates	Neocalanus cristatus	1.9200E-02	8	9.7805E-04	Makarieva et al. 2008
3177	Invertebrates	Neocalanus cristatus	1.9500E-02	2	6.0372E-04	Makarieva et al. 2008
3178	Invertebrates	Neocalanus cristatus	2.0200E-02	2	1.6362E-03	Makarieva et al. 2008
3179	Invertebrates	Neocalanus cristatus	2.0600E-02	2	6.7856E-04	Makarieva et al. 2008
3180	Invertebrates	Neocalanus cristatus	2.1000E-02	2	5.2542E-04	Makarieva et al. 2008
3181	Invertebrates	Neocalanus cristatus	2.2100E-02	2	6.8422E-04	Makarieva et al. 2008
3182	Invertebrates	Neocalanus cristatus	2.2600E-02	2	1.0414E-03	Makarieva et al. 2008

3183	Invertebrates	Neocalanus cristatus	2.3000E-02	2	4.5954E-04	Makarieva et al. 2008
3184	Invertebrates	Neocalanus cristatus	2.3600E-02	2	6.3720E-04	Makarieva et al. 2008
3185	Invertebrates	Neocalanus cristatus	2.4200E-02	2	5.7935E-04	Makarieva et al. 2008
3186	Invertebrates	Neocalanus cristatus	2.6000E-02	2	7.5348E-04	Makarieva et al. 2008
3187	Invertebrates	Neocalanus cristatus	2.6400E-02	2	5.7974E-04	Makarieva et al. 2008
3188	Invertebrates	Neocalanus cristatus	2.6900E-02	2	6.4399E-04	Makarieva et al. 2008
3189	Invertebrates	Neocalanus cristatus	2.9900E-02	2	1.0441E-03	Makarieva et al. 2008
3190	Invertebrates	Neocalanus gracilis	2.1200E-03	28	2.9257E-03	Makarieva et al. 2008
3191	Invertebrates	Neocalanus gracilis	2.5000E-03	19.7	1.6380E-03	Makarieva et al. 2008
3192	Invertebrates	Neocalanus plumchrus	1.3000E-03	5.6	2.7612E-04	Makarieva et al. 2008
3193	Invertebrates	Neocalanus plumchrus	1.5000E-03	15.1	5.6700E-04	Makarieva et al. 2008
3194	Invertebrates	Neocalanus plumchrus	4.0000E-03	7.3	6.9120E-04	Makarieva et al. 2008
3195	Invertebrates	Neoconocephalus robustus	8.7000E-01	25	6.7138E-01	Ehnes et al. 2011
3196	Invertebrates	Neocossus sp.	1.7716E+00	25	7.2153E-01	Ehnes et al. 2011
3197	Invertebrates	Neomysis awatschensis	2.5800E-03	20	1.5349E-03	Makarieva et al. 2008
3198	Invertebrates	Neomysis awatschensis	3.3900E-03	20	1.8277E-03	Makarieva et al. 2008
3199	Invertebrates	Neomysis awatschensis	5.4300E-03	20	2.4437E-03	Makarieva et al. 2008
3200	Invertebrates	Neomysis awatschensis	8.5500E-03	20	3.2414E-03	Makarieva et al. 2008
3201	Invertebrates	Neomysis awatschensis	1.0860E-02	20	3.7665E-03	Makarieva et al. 2008
3202	Invertebrates	Neomysis awatschensis	1.3600E-02	20	4.3119E-03	Makarieva et al. 2008
3203	Invertebrates	Neomysis awatschensis	1.4800E-02	20	4.5541E-03	Makarieva et al. 2008
3204	Invertebrates	Neomysis awatschensis	1.6200E-02	20	4.8470E-03	Makarieva et al. 2008
3205	Invertebrates	Neomysis awatschensis	1.9500E-02	20	5.4124E-03	Makarieva et al. 2008
3206	Invertebrates	Neomysis awatschensis	2.3500E-02	20	6.0688E-03	Makarieva et al. 2008
3207	Invertebrates	Neomysis mirabilis	1.1800E-02	20	9.8099E-03	Makarieva et al. 2008
3208	Invertebrates	Neomysis mirabilis	1.1800E-02	20	8.2500E-03	Makarieva et al. 2008

3209	Invertebrates	Neomysis mirabilis	1.1800E-02	20	8.5529E-03	Makarieva et al. 2008
3210	Invertebrates	Neophilaenus lineatus	1.9100E-03	25	5.5080E-03	Ehnes et al. 2011
3211	Invertebrates	Nephila clavipes	8.4800E-01	20	1.5150E-01	Ehnes et al. 2011
3212	Invertebrates	Neriene litigiosa	1.9000E-02	25	4.6800E-03	Ehnes et al. 2011
3213	Invertebrates	Niphargus krameri	1.4900E-02	10	1.0460E-03	Makarieva et al. 2008
3214	Invertebrates	Niphargus rhenorhodanensis	1.3000E-02	11	1.0296E-03	Makarieva et al. 2008
3215	Invertebrates	Niphargus sphagnicolus	5.9000E-03	10	1.5930E-03	Makarieva et al. 2008
3216	Invertebrates	Niphargus stygius	3.0400E-02	10	1.8058E-03	Makarieva et al. 2008
3217	Invertebrates	Niphargus virei	9.3000E-02	11	2.8458E-03	Makarieva et al. 2008
3218	Invertebrates	Nothrus palustris	9.0000E-06	10	2.5500E-06	Ehnes et al. 2011
3219	Invertebrates	Nothrus palustris	2.2000E-05	10	2.5500E-06	Ehnes et al. 2011
3220	Invertebrates	Nothrus palustris	3.7500E-05	10	6.0500E-06	Ehnes et al. 2011
3221	Invertebrates	Nothrus palustris	7.0300E-05	10	6.8500E-06	Ehnes et al. 2011
3222	Invertebrates	Nothrus palustris	2.4700E-04	10	2.2000E-05	Ehnes et al. 2011
3223	Invertebrates	Nothrus silvestris	3.5000E-06	10	5.5000E-07	Ehnes et al. 2011
3224	Invertebrates	Nothrus silvestris	6.8000E-06	10	1.4500E-06	Ehnes et al. 2011
3225	Invertebrates	Nothrus silvestris	7.6000E-06	15	5.4000E-06	Ehnes et al. 2011
3226	Invertebrates	Nothrus silvestris	1.2900E-05	10	2.2000E-06	Ehnes et al. 2011
3227	Invertebrates	Nothrus silvestris	1.5200E-05	15	9.3000E-06	Ehnes et al. 2011
3228	Invertebrates	Nothrus silvestris	3.0000E-05	10	4.8000E-06	Ehnes et al. 2011
3229	Invertebrates	Nothrus silvestris	3.4000E-05	15	1.3050E-05	Ehnes et al. 2011
3230	Invertebrates	Nothrus silvestris	4.7200E-05	5	2.5000E-06	Ehnes et al. 2011
3231	Invertebrates	Nothrus silvestris	4.7200E-05	10	5.3000E-06	Ehnes et al. 2011
3232	Invertebrates	Nothrus silvestris	4.7200E-05	15	1.0250E-05	Ehnes et al. 2011
3233	Invertebrates	Nothrus silvestris	5.2400E-05	5	3.1000E-06	Ehnes et al. 2011
3234	Invertebrates	Nothrus silvestris	5.2400E-05	10	6.3000E-06	Ehnes et al. 2011

3235	Invertebrates	Nothrus silvestris	5.2400E-05	15	7.3000E-06	Ehnes et al. 2011
3236	Invertebrates	Nothrus silvestris	5.2400E-05	20	1.3200E-05	Ehnes et al. 2011
3237	Invertebrates	Nothrus silvestris	5.6600E-05	15	1.6600E-05	Ehnes et al. 2011
3238	Invertebrates	Notiophilus	4.2000E-03	22	5.5376E-03	Ehnes et al. 2011
3239	Invertebrates	Notiophilus	4.4000E-03	22	2.7688E-03	Ehnes et al. 2011
3240	Invertebrates	Notiophilus	4.6000E-03	11.5	2.0402E-03	Ehnes et al. 2011
3241	Invertebrates	Notiophilus	4.7000E-03	8	2.9145E-04	Ehnes et al. 2011
3242	Invertebrates	Notiophilus	4.9000E-03	15	1.3116E-03	Ehnes et al. 2011
3243	Invertebrates	Notiophilus	5.2000E-03	11.5	2.9145E-04	Ehnes et al. 2011
3244	Invertebrates	Notiophilus	5.2000E-03	22	8.1607E-03	Ehnes et al. 2011
3245	Invertebrates	Notiophilus	5.3000E-03	22	4.3718E-03	Ehnes et al. 2011
3246	Invertebrates	Notiophilus	5.5000E-03	8	5.8290E-04	Ehnes et al. 2011
3247	Invertebrates	Notiophilus	5.5000E-03	11.5	1.1658E-03	Ehnes et al. 2011
3248	Invertebrates	Notiophilus	5.5000E-03	18.5	2.1859E-03	Ehnes et al. 2011
3249	Invertebrates	Notiophilus	5.6000E-03	15	1.8945E-03	Ehnes et al. 2011
3250	Invertebrates	Notiophilus	6.0000E-03	11.5	2.9145E-03	Ehnes et al. 2011
3251	Invertebrates	Notiophilus	6.0000E-03	18.5	4.0803E-03	Ehnes et al. 2011
3252	Invertebrates	Notiophilus	6.4000E-03	22	7.2863E-03	Ehnes et al. 2011
3253	Invertebrates	Notiophilus	7.2000E-03	11.5	1.3116E-03	Ehnes et al. 2011
3254	Invertebrates	Notiophilus	7.2000E-03	11.5	3.3517E-03	Ehnes et al. 2011
3255	Invertebrates	Notiophilus	7.2000E-03	18.5	5.6833E-03	Ehnes et al. 2011
3256	Invertebrates	Notiophilus	7.3000E-03	15	2.9145E-03	Ehnes et al. 2011
3257	Invertebrates	Notiophilus	7.6000E-03	11.5	2.6231E-03	Ehnes et al. 2011
3258	Invertebrates	Notiophilus	7.6000E-03	18.5	2.6231E-03	Ehnes et al. 2011
3259	Invertebrates	Notiophilus	7.6000E-03	18.5	4.3718E-03	Ehnes et al. 2011
3260	Invertebrates	Notiophilus	7.9000E-03	22	1.6030E-03	Ehnes et al. 2011

3261	Invertebrates	Notiophilus	8.8000E-03	15	3.4974E-03	Ehnes et al. 2011
3262	Invertebrates	Notocrangon antarcticus	1.0000E+00	2	9.7020E-02	Makarieva et al. 2008
3263	Invertebrates	Notostomus elegans	2.2540E+01	5	6.7755E-01	Makarieva et al. 2008
3264	Invertebrates	Notostomus elegans	2.2540E+01	10	1.4525E+00	Makarieva et al. 2008
3265	Invertebrates	Notostomus gibbosus	1.0400E+01	5	1.1232E-01	Makarieva et al. 2008
3266	Invertebrates	Notostomus gibbosus	1.0400E+01	10	2.9952E-01	Makarieva et al. 2008
3267	Invertebrates	Notostomus sp.	4.0000E+01	4	1.9440E-01	Makarieva et al. 2008
3268	Invertebrates	Notostomus sp.	4.0000E+01	4	3.0240E-01	Makarieva et al. 2008
3269	Invertebrates	Nyctobates procerus	2.1980E+00	25	3.1559E-01	Ehnes et al. 2011
3270	Invertebrates	Nyssicus setosus	9.9600E-01	25	1.2879E-01	Ehnes et al. 2011
3271	Invertebrates	Octolasium lacteum	1.0750E+00	19	7.6000E-02	Ehnes et al. 2011
3272	Invertebrates	Octopus bimaculoides	3.6000E+02	5	4.5943E+00	Makarieva et al. 2008
3273	Invertebrates	Octopus briareus	3.4550E+02	5	5.0560E+00	Makarieva et al. 2008
3274	Invertebrates	Octopus californicus	3.3000E+02	5	2.8809E+00	Makarieva et al. 2008
3275	Invertebrates	Octopus cyanea	1.1500E+03	5	2.5502E+01	Makarieva et al. 2008
3276	Invertebrates	Octopus dofleini	8.2000E+03	5	5.5055E+01	Makarieva et al. 2008
3277	Invertebrates	Octopus maya	4.5100E+01	5	2.4752E+00	Makarieva et al. 2008
3278	Invertebrates	Octopus micropyrsus	4.6500E+00	5	1.7184E-01	Makarieva et al. 2008
3279	Invertebrates	Octopus rubescens	9.6000E-02	5	1.5913E-02	Makarieva et al. 2008
3280	Invertebrates	Octopus sp.	1.2000E-03	5	3.6020E-04	Makarieva et al. 2008
3281	Invertebrates	Octopus sp.	6.8000E-03	5	3.0997E-03	Makarieva et al. 2008
3282	Invertebrates	Octopus tuberculata	1.2100E+00	5	1.1302E-01	Makarieva et al. 2008
3283	Invertebrates	Octopus vulgaris	2.1600E+03	5	1.8390E+01	Makarieva et al. 2008
3284	Invertebrates	Ocypode platytarsis	5.0000E-01	20	2.0196E-01	Makarieva et al. 2008
3285	Invertebrates	Ocypode platytarsis	1.0000E+00	20	2.8881E-01	Makarieva et al. 2008
3286	Invertebrates	Ocypode platytarsis	2.0000E+00	20	4.2109E-01	Makarieva et al. 2008

3287	Invertebrates	Ocypode platytarsis	3.0000E+00	20	5.2007E-01	Makarieva et al. 2008
3288	Invertebrates	Ocypode platytarsis	3.5800E+00	27	1.3030E+00	Makarieva et al. 2008
3289	Invertebrates	Ocypode platytarsis	5.0000E+00	20	6.9777E-01	Makarieva et al. 2008
3290	Invertebrates	Ocypode platytarsis	1.0000E+01	20	9.9864E-01	Makarieva et al. 2008
3291	Invertebrates	Ocypode quadrata	2.1000E+00	20	3.9482E-01	Makarieva et al. 2008
3292	Invertebrates	Ocypode quadrata	2.6900E+01	20	2.4254E+00	Makarieva et al. 2008
3293	Invertebrates	Ocypode quadrata	3.3300E+01	24	4.8599E+00	Makarieva et al. 2008
3294	Invertebrates	Ocypode quadrata	7.0900E+01	20	3.7278E+00	Makarieva et al. 2008
3295	Invertebrates	Ocypus olens	7.4380E-02	10	5.1004E-03	Ehnes et al. 2011
3296	Invertebrates	Ocypus olens	8.6700E-02	10	2.0402E-03	Ehnes et al. 2011
3297	Invertebrates	Ocypus olens	1.0028E-01	10	6.7034E-03	Ehnes et al. 2011
3298	Invertebrates	Ocypus olens	2.3182E-01	15	6.2953E-02	Ehnes et al. 2011
3299	Invertebrates	Ocypus olens	2.3955E-01	15	5.9310E-02	Ehnes et al. 2011
3300	Invertebrates	Ocypus olens	2.8337E-01	15	3.8180E-02	Ehnes et al. 2011
3301	Invertebrates	Ocypus ophtalmicus	6.5010E-02	25	3.3371E-02	Ehnes et al. 2011
3302	Invertebrates	Ocypus ophtalmicus	6.5100E-02	25	4.6632E-02	Ehnes et al. 2011
3303	Invertebrates	Ocypus ophtalmicus	6.7330E-02	25	3.1477E-02	Ehnes et al. 2011
3304	Invertebrates	Ocypus ophtalmicus	7.0820E-02	25	5.5084E-02	Ehnes et al. 2011
3305	Invertebrates	Ocypus ophtalmicus	7.5160E-02	10	5.6833E-03	Ehnes et al. 2011
3306	Invertebrates	Ocypus ophtalmicus	7.7290E-02	25	2.1276E-02	Ehnes et al. 2011
3307	Invertebrates	Ocypus ophtalmicus	7.9390E-02	20	2.1422E-02	Ehnes et al. 2011
3308	Invertebrates	Ocypus ophtalmicus	8.4240E-02	25	2.4045E-02	Ehnes et al. 2011
3309	Invertebrates	Ocypus ophtalmicus	8.7460E-02	10	4.3718E-03	Ehnes et al. 2011
3310	Invertebrates	Ocypus ophtalmicus	8.8530E-02	30	6.2662E-02	Ehnes et al. 2011
3311	Invertebrates	Ocypus ophtalmicus	9.0160E-02	20	1.8944E-02	Ehnes et al. 2011
3312	Invertebrates	Ocypus ophtalmicus	1.0283E-01	10	5.5376E-03	Ehnes et al. 2011

3313	Invertebrates	Ocypus ophtalmicus	1.0478E-01	20	4.8089E-02	Ehnes et al. 2011
3314	Invertebrates	Ocypus ophtalmicus	1.0628E-01	30	9.2244E-02	Ehnes et al. 2011
3315	Invertebrates	Ocypus ophtalmicus	1.2160E-01	30	7.3008E-02	Ehnes et al. 2011
3316	Invertebrates	Ocypus ophtalmicus	1.2244E-01	25	3.9783E-02	Ehnes et al. 2011
3317	Invertebrates	Ocypus ophtalmicus	1.2261E-01	10	7.5777E-03	Ehnes et al. 2011
3318	Invertebrates	Ocypus ophtalmicus	1.2464E-01	25	4.1823E-02	Ehnes et al. 2011
3319	Invertebrates	Ocypus ophtalmicus	1.2698E-01	20	2.9291E-02	Ehnes et al. 2011
3320	Invertebrates	Ocypus ophtalmicus	1.2719E-01	10	6.7034E-03	Ehnes et al. 2011
3321	Invertebrates	Ocypus ophtalmicus	1.2758E-01	10	8.0149E-03	Ehnes et al. 2011
3322	Invertebrates	Ocypus ophtalmicus	1.2873E-01	25	4.5321E-02	Ehnes et al. 2011
3323	Invertebrates	Ocypus ophtalmicus	1.3127E-01	15	1.7779E-02	Ehnes et al. 2011
3324	Invertebrates	Ocypus ophtalmicus	1.3148E-01	15	3.1768E-02	Ehnes et al. 2011
3325	Invertebrates	Ocypus ophtalmicus	1.3172E-01	20	3.2934E-02	Ehnes et al. 2011
3326	Invertebrates	Ocypus ophtalmicus	1.3462E-01	25	3.5120E-02	Ehnes et al. 2011
3327	Invertebrates	Ocypus ophtalmicus	1.3932E-01	20	3.2060E-02	Ehnes et al. 2011
3328	Invertebrates	Ocypus ophtalmicus	1.4048E-01	30	7.8255E-02	Ehnes et al. 2011
3329	Invertebrates	Ocypus ophtalmicus	1.4079E-01	30	8.9621E-02	Ehnes et al. 2011
3330	Invertebrates	Ocypus ophtalmicus	1.4301E-01	15	2.2879E-02	Ehnes et al. 2011
3331	Invertebrates	Ocypus ophtalmicus	1.4754E-01	30	7.7672E-02	Ehnes et al. 2011
3332	Invertebrates	Ocypus ophtalmicus	1.5847E-01	25	7.5194E-02	Ehnes et al. 2011
3333	Invertebrates	Odonestis pruni	2.5000E-01	25	2.0340E-01	Ehnes et al. 2011
3334	Invertebrates	Oecanthus quadripunctatus	5.0000E-02	25	2.4174E-02	Ehnes et al. 2011
3335	Invertebrates	Oedalis instillatus	6.2700E-01	25	1.7395E-01	Ehnes et al. 2011
3336	Invertebrates	Oithona davisae	3.0000E-06	25	5.3190E-06	Makarieva et al. 2008
3337	Invertebrates	Oithona minuta	3.9000E-06	20	1.4812E-05	Makarieva et al. 2008
3338	Invertebrates	Oithona nana	2.6000E-06	12	3.2760E-06	Makarieva et al. 2008

3339	Invertebrates	Oithona plumifera	8.9000E-06	24	2.8836E-06	Makarieva et al. 2008
3340	Invertebrates	Oithona setigera	8.6000E-06	24	9.2880E-06	Makarieva et al. 2008
3341	Invertebrates	Oithona similis	7.0000E-06	25	6.0480E-07	Makarieva et al. 2008
3342	Invertebrates	Oithona similis	7.0000E-06	25	9.8280E-06	Makarieva et al. 2008
3343	Invertebrates	Oithona tenuis	6.7000E-06	24	6.6330E-06	Makarieva et al. 2008
3344	Invertebrates	Omorgus radula	2.0700E-01	25	2.0538E-02	Ehnes et al. 2011
3345	Invertebrates	Oncaea conifera	6.0000E-05	20	4.3265E-05	Makarieva et al. 2008
3346	Invertebrates	Oncaea mediterranea	6.2000E-05	20	5.2519E-05	Makarieva et al. 2008
3347	Invertebrates	Onchocalanus magnus	1.2400E-02	5	9.3744E-04	Makarieva et al. 2008
3348	Invertebrates	Oncideres putator	6.8100E-01	25	1.9726E-01	Ehnes et al. 2011
3349	Invertebrates	Oniscus asellus	1.4043E-03	15.8	4.1635E-04	Ehnes et al. 2011
3350	Invertebrates	Oniscus asellus	1.8000E-03	20	2.8081E-03	Makarieva et al. 2008
3351	Invertebrates	Oniscus asellus	2.1686E-03	15.8	5.8290E-04	Ehnes et al. 2011
3352	Invertebrates	Oniscus asellus	3.3767E-03	5	5.8290E-04	Ehnes et al. 2011
3353	Invertebrates	Oniscus asellus	6.1000E-03	20	5.4900E-03	Makarieva et al. 2008
3354	Invertebrates	Oniscus asellus	6.2567E-03	15.8	1.2630E-03	Ehnes et al. 2011
3355	Invertebrates	Oniscus asellus	7.4300E-03	18	5.8290E-04	Ehnes et al. 2011
3356	Invertebrates	Oniscus asellus	8.8100E-03	10.3	7.2865E-04	Ehnes et al. 2011
3357	Invertebrates	Oniscus asellus	8.8900E-03	19.5	4.0075E-03	Ehnes et al. 2011
3358	Invertebrates	Oniscus asellus	1.0000E-02	20	8.4006E-03	Makarieva et al. 2008
3359	Invertebrates	Oniscus asellus	1.0100E-02	16	4.2000E-03	Ehnes et al. 2011
3360	Invertebrates	Oniscus asellus	1.0560E-02	18	3.6432E-03	Ehnes et al. 2011
3361	Invertebrates	Oniscus asellus	1.1760E-02	19.5	6.2662E-03	Ehnes et al. 2011
3362	Invertebrates	Oniscus asellus	1.2290E-02	10.3	1.6030E-03	Ehnes et al. 2011
3363	Invertebrates	Oniscus asellus	1.4350E-02	18	5.2461E-03	Ehnes et al. 2011
3364	Invertebrates	Oniscus asellus	1.7720E-02	19.5	6.4119E-03	Ehnes et al. 2011

3365	Invertebrates	Oniscus asellus	1.7750E-02	5	1.0201E-03	Ehnes et al. 2011
3366	Invertebrates	Oniscus asellus	1.8000E-02	20	7.1993E-03	Makarieva et al. 2008
3367	Invertebrates	Oniscus asellus	1.8580E-02	19.5	5.3919E-03	Ehnes et al. 2011
3368	Invertebrates	Oniscus asellus	1.9510E-02	5	2.0402E-03	Ehnes et al. 2011
3369	Invertebrates	Oniscus asellus	2.0000E-02	20	6.0012E-03	Makarieva et al. 2008
3370	Invertebrates	Oniscus asellus	2.0140E-02	18	5.2461E-03	Ehnes et al. 2011
3371	Invertebrates	Oniscus asellus	2.1430E-02	18	3.9346E-03	Ehnes et al. 2011
3372	Invertebrates	Oniscus asellus	2.1490E-02	10.3	2.6231E-03	Ehnes et al. 2011
3373	Invertebrates	Oniscus asellus	2.1700E-02	19.5	5.5376E-03	Ehnes et al. 2011
3374	Invertebrates	Oniscus asellus	2.1860E-02	19.5	6.7034E-03	Ehnes et al. 2011
3375	Invertebrates	Oniscus asellus	2.2160E-02	19.5	7.1406E-03	Ehnes et al. 2011
3376	Invertebrates	Oniscus asellus	2.3830E-02	19.5	1.0492E-02	Ehnes et al. 2011
3377	Invertebrates	Oniscus asellus	2.4020E-02	9.2	2.7688E-03	Ehnes et al. 2011
3378	Invertebrates	Oniscus asellus	2.4600E-02	10.3	2.1859E-03	Ehnes et al. 2011
3379	Invertebrates	Oniscus asellus	2.5470E-02	18	4.2261E-03	Ehnes et al. 2011
3380	Invertebrates	Oniscus asellus	2.7050E-02	18	4.6632E-03	Ehnes et al. 2011
3381	Invertebrates	Oniscus asellus	3.1170E-02	14.7	4.6632E-03	Ehnes et al. 2011
3382	Invertebrates	Oniscus asellus	3.7550E-02	14.7	4.6632E-03	Ehnes et al. 2011
3383	Invertebrates	Oniscus asellus	3.9320E-02	14.6	2.7688E-03	Ehnes et al. 2011
3384	Invertebrates	Oniscus asellus	3.9510E-02	14.7	2.9145E-03	Ehnes et al. 2011
3385	Invertebrates	Oniscus asellus	4.3770E-02	29.9	1.8070E-02	Ehnes et al. 2011
3386	Invertebrates	Oniscus asellus	4.4000E-02	14.7	2.6231E-03	Ehnes et al. 2011
3387	Invertebrates	Oniscus asellus	4.4630E-02	14.9	4.5175E-03	Ehnes et al. 2011
3388	Invertebrates	Oniscus asellus	4.5220E-02	14.6	3.2060E-03	Ehnes et al. 2011
3389	Invertebrates	Oniscus asellus	4.5280E-02	14.9	3.3517E-03	Ehnes et al. 2011
3390	Invertebrates	Oniscus asellus	4.5390E-02	23.4	1.0492E-02	Ehnes et al. 2011

3391	Invertebrates	Oniscus asellus	4.6250E-02	10	2.1859E-03	Ehnes et al. 2011
3392	Invertebrates	Oniscus asellus	4.7600E-02	5	2.0402E-03	Ehnes et al. 2011
3393	Invertebrates	Oniscus asellus	4.7970E-02	5.2	1.6030E-03	Ehnes et al. 2011
3394	Invertebrates	Oniscus asellus	4.8750E-02	14.7	2.7688E-03	Ehnes et al. 2011
3395	Invertebrates	Oniscus asellus	5.1850E-02	5.2	5.8290E-04	Ehnes et al. 2011
3396	Invertebrates	Oniscus asellus	5.3500E-02	14.9	3.9346E-03	Ehnes et al. 2011
3397	Invertebrates	Oniscus asellus	5.4400E-02	14.6	5.3919E-03	Ehnes et al. 2011
3398	Invertebrates	Oniscus asellus	5.6620E-02	29.9	1.7341E-02	Ehnes et al. 2011
3399	Invertebrates	Oniscus asellus	5.6900E-02	16	1.0300E-02	Ehnes et al. 2011
3400	Invertebrates	Oniscus asellus	5.7300E-02	29.2	2.0402E-02	Ehnes et al. 2011
3401	Invertebrates	Oniscus asellus	6.0000E-02	20	2.1600E-02	Makarieva et al. 2008
3402	Invertebrates	Oniscus asellus	6.0520E-02	30	1.5155E-02	Ehnes et al. 2011
3403	Invertebrates	Oniscus asellus	6.5260E-02	10	1.7487E-03	Ehnes et al. 2011
3404	Invertebrates	Oniscus asellus	6.6240E-02	29.9	2.1276E-02	Ehnes et al. 2011
3405	Invertebrates	Oniscus asellus	6.6850E-02	14.6	1.2970E-02	Ehnes et al. 2011
3406	Invertebrates	Oniscus asellus	6.8300E-02	16	1.6900E-02	Ehnes et al. 2011
3407	Invertebrates	Oniscus asellus	6.9210E-02	14.6	8.3064E-03	Ehnes et al. 2011
3408	Invertebrates	Oniscus asellus	7.1720E-02	29.9	1.8070E-02	Ehnes et al. 2011
3409	Invertebrates	Oniscus asellus	7.1870E-02	5.2	2.3316E-03	Ehnes et al. 2011
3410	Invertebrates	Oniscus asellus	7.2950E-02	14.6	5.9748E-03	Ehnes et al. 2011
3411	Invertebrates	Oniscus asellus	7.3720E-02	23.4	1.2095E-02	Ehnes et al. 2011
3412	Invertebrates	Oniscus asellus	7.5950E-02	5	3.3517E-03	Ehnes et al. 2011
3413	Invertebrates	Oniscus asellus	7.7160E-02	10	3.2060E-03	Ehnes et al. 2011
3414	Invertebrates	Oniscus asellus	7.8030E-02	5	1.8945E-03	Ehnes et al. 2011
3415	Invertebrates	Oniscus asellus	7.8250E-02	14.6	4.6632E-03	Ehnes et al. 2011
3416	Invertebrates	Oniscus asellus	7.8540E-02	14.6	7.2863E-03	Ehnes et al. 2011

3417	Invertebrates	Oniscus asellus	7.9840E-02	10	2.3316E-03	Ehnes et al. 2011
3418	Invertebrates	Oniscus asellus	8.1180E-02	5	1.8945E-03	Ehnes et al. 2011
3419	Invertebrates	Oniscus asellus	8.9410E-02	14.6	8.7436E-03	Ehnes et al. 2011
3420	Invertebrates	Oniscus asellus	9.7750E-02	14.6	7.1406E-03	Ehnes et al. 2011
3421	Invertebrates	Oniscus asellus	9.8340E-02	5.2	2.4774E-03	Ehnes et al. 2011
3422	Invertebrates	Oniscus asellus	1.0000E-01	20	1.9998E-02	Makarieva et al. 2008
3423	Invertebrates	Oniscus asellus	1.0804E-01	14.6	1.6030E-02	Ehnes et al. 2011
3424	Invertebrates	Oniscus asellus	1.5000E-01	20	1.8009E-02	Makarieva et al. 2008
3425	Invertebrates	Onymacris laeviceps	5.2500E-01	25	7.7022E-02	Ehnes et al. 2011
3426	Invertebrates	Onymacris plana	7.6700E-01	25	1.6666E-01	Ehnes et al. 2011
3427	Invertebrates	Onymacris rugatipennis	4.9600E-01	25	8.0316E-02	Ehnes et al. 2011
3428	Invertebrates	Onymacris unguicularis	7.3700E-01	25	3.1680E-02	Ehnes et al. 2011
3429	Invertebrates	Ophryastes varius	2.7260E-02	25	2.1474E-02	Ehnes et al. 2011
3430	Invertebrates	Opiliones	3.0000E-03	8	1.0201E-03	Ehnes et al. 2011
3431	Invertebrates	Oplophorus gracilorostris	2.5800E+00	10	6.1301E-01	Makarieva et al. 2008
3432	Invertebrates	Oplophorus gracilorostris	2.5800E+00	20	1.3421E+00	Makarieva et al. 2008
3433	Invertebrates	Oplophorus gracilorostris	2.5800E+00	5	7.5697E-01	Makarieva et al. 2008
3434	Invertebrates	Oplophorus spinosus	2.9200E+00	10	3.3218E-01	Makarieva et al. 2008
3435	Invertebrates	Oplophorus spinosus	2.9200E+00	20	1.5348E+00	Makarieva et al. 2008
3436	Invertebrates	Oplophorus spinosus	2.9200E+00	5	5.5188E-01	Makarieva et al. 2008
3437	Invertebrates	Oppia nova	1.7000E-06	5	1.5000E-07	Ehnes et al. 2011
3438	Invertebrates	Oppia nova	1.7000E-06	10	2.0000E-07	Ehnes et al. 2011
3439	Invertebrates	Oppia nova	1.7000E-06	15	6.5000E-07	Ehnes et al. 2011
3440	Invertebrates	Oppia subpectinata	3.2000E-06	5	4.0000E-07	Ehnes et al. 2011
3441	Invertebrates	Oppia subpectinata	3.2000E-06	10	7.0000E-07	Ehnes et al. 2011
3442	Invertebrates	Oppia subpectinata	3.2000E-06	15	1.4500E-06	Ehnes et al. 2011

3443	Invertebrates	Orchesella cincta	1.0000E-03	15	3.1800E-04	Ehnes et al. 2011
3444	Invertebrates	Orchesella cincta	1.0000E-03	15	3.9700E-04	Ehnes et al. 2011
3445	Invertebrates	Orchesella cincta	1.3400E-03	18	5.6000E-04	Ehnes et al. 2011
3446	Invertebrates	Orchesella cincta	1.5500E-03	18	6.4500E-04	Ehnes et al. 2011
3447	Invertebrates	Orchesella flavescens	1.4800E-03	8	2.1300E-04	Ehnes et al. 2011
3448	Invertebrates	Orchesella villosa	2.3000E-03	19	5.2000E-04	Ehnes et al. 2011
3449	Invertebrates	Orchestia bottae	1.1000E-01	20	1.3969E-02	Makarieva et al. 2008
3450	Invertebrates	Orchestia bottae	1.1000E-01	20	4.0032E-02	Makarieva et al. 2008
3451	Invertebrates	Orchomene sp.	3.6200E-03	29.85	1.4335E-02	Makarieva et al. 2008
3452	Invertebrates	Orchomenella chilensis	5.0000E-02	0	5.7960E-03	Makarieva et al. 2008
3453	Invertebrates	Orchomenella plebs	7.2000E-02	2	6.9854E-03	Makarieva et al. 2008
3454	Invertebrates	Orchomenella plebs	7.3000E-02	-1.8	6.9379E-03	Makarieva et al. 2008
3455	Invertebrates	Orchomenella plebs	8.8000E-02	-1.8	8.0942E-03	Makarieva et al. 2008
3456	Invertebrates	Orchomenella plebs	9.1000E-02	-1.8	6.3718E-03	Makarieva et al. 2008
3457	Invertebrates	Orchomenella plebs	9.7000E-02	-1.8	8.6252E-03	Makarieva et al. 2008
3458	Invertebrates	Orchomenella plebs	1.0500E-01	1	7.0308E-03	Makarieva et al. 2008
3459	Invertebrates	Orchomenella plebs	1.4800E-01	-1	1.0496E-02	Makarieva et al. 2008
3460	Invertebrates	Orchomenella plebs	1.6100E-01	0	9.0128E-03	Makarieva et al. 2008
3461	Invertebrates	Orchomenella plebs	1.9400E-01	-1.8	1.2990E-02	Makarieva et al. 2008
3462	Invertebrates	Orchomenella sp.	2.0600E-01	-1	1.8948E-02	Makarieva et al. 2008
3463	Invertebrates	Oribatella quadricornuta	2.4000E-05	5	1.0000E-06	Ehnes et al. 2011
3464	Invertebrates	Oribatella quadricornuta	2.4000E-05	10	2.5000E-06	Ehnes et al. 2011
3465	Invertebrates	Oribatella quadricornuta	2.4000E-05	15	5.2500E-06	Ehnes et al. 2011
3466	Invertebrates	Orodillo maculatus	1.5800E-02	23	3.5200E-03	Ehnes et al. 2011
3467	Invertebrates	Oryba achemenides	2.8085E+00	25	1.0904E+00	Ehnes et al. 2011
3468	Invertebrates	Ostrinia nubilalis	4.8400E-02	25	8.7156E-02	Ehnes et al. 2011

3469	Invertebrates	Pachydiplax longipennis	2.0000E-01	25	3.1774E-01	Ehnes et al. 2011
3470	Invertebrates	Pachygonia drucei	7.0200E-01	25	2.6663E-01	Ehnes et al. 2011
3471	Invertebrates	Pachygrapsus marmoratus	1.5200E-01	20	1.3532E-02	Makarieva et al. 2008
3472	Invertebrates	Pachygrapsus marmoratus	3.2300E+00	20	3.5244E-01	Makarieva et al. 2008
3473	Invertebrates	Pachygrapsus marmoratus	4.4000E+00	20	6.6845E-01	Makarieva et al. 2008
3474	Invertebrates	Pachygrapsus marmoratus	5.0900E+00	20	4.9383E-01	Makarieva et al. 2008
3475	Invertebrates	Pachygrapsus marmoratus	5.3000E+00	20	4.5983E-01	Makarieva et al. 2008
3476	Invertebrates	Pachygrapsus marmoratus	7.7700E+00	20	6.8364E-01	Makarieva et al. 2008
3477	Invertebrates	Pachygrapsus marmoratus	1.4700E+01	20	1.0835E+00	Makarieva et al. 2008
3478	Invertebrates	Pachylaelaps lindrothi	8.1900E-05	10	1.8450E-05	Ehnes et al. 2011
3479	Invertebrates	Pachylia ficus	3.2250E+00	25	9.2068E-01	Ehnes et al. 2011
3480	Invertebrates	Pachylomerus femoralis	4.9150E+00	25	7.7080E-01	Ehnes et al. 2011
3481	Invertebrates	Pachyptilus pacificus	1.5800E-02	5	4.9201E-04	Makarieva et al. 2008
3482	Invertebrates	Pachyptilus pacificus	1.5800E-02	1.5	4.4366E-04	Makarieva et al. 2008
3483	Invertebrates	Palaemon adspersus	1.0000E-01	20	4.5441E-02	Makarieva et al. 2008
3484	Invertebrates	Palaemon adspersus	1.3200E-01	20	5.7155E-02	Makarieva et al. 2008
3485	Invertebrates	Palaemon adspersus	3.2000E+00	20	6.2404E-01	Makarieva et al. 2008
3486	Invertebrates	Palaemon adspersus	3.2800E+00	20	7.0080E-01	Makarieva et al. 2008
3487	Invertebrates	Palirhoeus eatoni	6.7000E-03	25	4.3740E-03	Ehnes et al. 2011
3488	Invertebrates	Pandalopsis ampla	5.9600E+00	3	1.1801E-01	Makarieva et al. 2008
3489	Invertebrates	Pandalopsis ampla	9.5500E+00	7.5	1.5299E-01	Makarieva et al. 2008
3490	Invertebrates	Pandalus jordani	1.2080E+01	5	5.4360E-01	Makarieva et al. 2008
3491	Invertebrates	Pandalus jordani	1.2670E+01	10	7.7540E-01	Makarieva et al. 2008
3492	Invertebrates	Pandalus jordani	1.2800E+01	15	7.8336E-01	Makarieva et al. 2008
3493	Invertebrates	Pandalus platyceros	2.5400E+01	5	1.0516E+00	Makarieva et al. 2008
3494	Invertebrates	Pandalus platyceros	3.2260E+01	15	2.3808E+00	Makarieva et al. 2008

3495	Invertebrates	Pandalus platyceros	4.0890E+01	10	2.4289E+00	Makarieva et al. 2008
3496	Invertebrates	Panopeus herbstii	1.0000E+00	20	6.9786E-02	Makarieva et al. 2008
3497	Invertebrates	Panopeus herbstii	1.0000E+01	20	5.2308E-01	Makarieva et al. 2008
3498	Invertebrates	Pantachogon sp. A	5.1000E-01	5	2.9376E-03	Makarieva et al. 2008
3499	Invertebrates	Pantala flavescens	3.3900E-01	25	3.0155E-01	Ehnes et al. 2011
3500	Invertebrates	Pantophthalmus tabaninus	1.7460E+00	25	5.4664E-01	Ehnes et al. 2011
3501	Invertebrates	Paracalanus parvus	1.9000E-05	13.9	3.0985E-05	Makarieva et al. 2008
3502	Invertebrates	Paracallisoma coecus	2.0000E-01	5.5	7.2000E-03	Makarieva et al. 2008
3503	Invertebrates	Paracallisoma coecus	2.0000E-01	5.5	1.0440E-02	Makarieva et al. 2008
3504	Invertebrates	Parachipteria willmanni	6.1000E-06	10	2.0000E-06	Ehnes et al. 2011
3505	Invertebrates	Parachipteria willmanni	1.2200E-05	10	3.0500E-06	Ehnes et al. 2011
3506	Invertebrates	Parachipteria willmanni	1.3100E-05	10	5.9500E-06	Ehnes et al. 2011
3507	Invertebrates	Parachipteria willmanni	2.6600E-05	10	6.4500E-06	Ehnes et al. 2011
3508	Invertebrates	Parachipteria willmanni	3.9600E-05	5	2.3500E-06	Ehnes et al. 2011
3509	Invertebrates	Parachipteria willmanni	3.9600E-05	10	6.0000E-06	Ehnes et al. 2011
3510	Invertebrates	Parachipteria willmanni	3.9600E-05	15	1.1300E-05	Ehnes et al. 2011
3511	Invertebrates	Parachipteria willmanni	7.3800E-05	10	9.8000E-06	Ehnes et al. 2011
3512	Invertebrates	Parachipteria willmanni	9.2600E-05	10	1.1300E-05	Ehnes et al. 2011
3513	Invertebrates	Paractora dreuxi	1.9170E-02	25	4.3902E-02	Ehnes et al. 2011
3514	Invertebrates	Paractora trichosterna	1.3260E-02	25	4.7034E-02	Ehnes et al. 2011
3515	Invertebrates	Paraeuchaeta antarctica	1.9551E-02	0	4.5046E-04	Makarieva et al. 2008
3516	Invertebrates	Paraeuchaeta birostrata	1.2600E-02	5	6.5545E-04	Makarieva et al. 2008
3517	Invertebrates	Paraeuchaeta birostrata	1.2900E-02	10	8.3824E-04	Makarieva et al. 2008
3518	Invertebrates	Paraeuchaeta brevicauda	2.6400E-02	5	1.0835E-03	Makarieva et al. 2008
3519	Invertebrates	Paraeuchaeta californica	1.3700E-02	5	7.4227E-04	Makarieva et al. 2008
3520	Invertebrates	Paraeuchaeta japonica	9.3000E-03	6.7	1.1249E-03	Makarieva et al. 2008

3521	Invertebrates	Paraeuchaeta norvegica	1.9000E-02	10	2.1888E-03	Makarieva et al. 2008
3522	Invertebrates	Paraeuchaeta norvegica	1.9400E-02	10	1.9555E-03	Makarieva et al. 2008
3523	Invertebrates	Paraeuchaeta norvegica	2.2300E-02	10	1.9669E-03	Makarieva et al. 2008
3524	Invertebrates	Paraeuchaeta rubra	1.2700E-02	5	4.8463E-04	Makarieva et al. 2008
3525	Invertebrates	Paraeuchaeta rubra	1.3100E-02	2	7.7814E-04	Makarieva et al. 2008
3526	Invertebrates	Paraeuchaeta sesquipedalis	2.4900E-02	5	1.1429E-03	Makarieva et al. 2008
3527	Invertebrates	Paraeuchaeta sp.	1.7000E-02	0	3.2803E-03	Makarieva et al. 2008
3528	Invertebrates	Paraeuchaeta tonsa	6.6000E-03	5	6.4271E-04	Makarieva et al. 2008
3529	Invertebrates	Paraledone characoti	1.3670E+02	5	1.3779E+00	Makarieva et al. 2008
3530	Invertebrates	Paralomis multispina	7.6400E+00	3	9.2138E-02	Makarieva et al. 2008
3531	Invertebrates	Paramoera walkeri	2.0000E-02	-1.9	1.0008E-03	Makarieva et al. 2008
3532	Invertebrates	Paramoera walkeri	2.0000E-02	0	1.4400E-03	Makarieva et al. 2008
3533	Invertebrates	Paramoera walkeri	2.0000E-02	2	2.0016E-03	Makarieva et al. 2008
3534	Invertebrates	Paramoera walkeri	2.1000E-02	-1.2	1.8673E-03	Makarieva et al. 2008
3535	Invertebrates	Paramoera walkeri	2.8000E-02	2.2	2.4091E-03	Makarieva et al. 2008
3536	Invertebrates	Paramoera walkeri	4.4000E-02	0	1.8454E-03	Makarieva et al. 2008
3537	Invertebrates	Paramoera walkeri	4.4000E-02	0	2.5978E-03	Makarieva et al. 2008
3538	Invertebrates	Paramoera walkeri	4.4000E-02	0	2.9938E-03	Makarieva et al. 2008
3539	Invertebrates	Paramysis kessleri	3.0000E-02	20	8.8862E-03	Makarieva et al. 2008
3540	Invertebrates	Paramysis kessleri	5.1500E-02	20	2.6153E-02	Makarieva et al. 2008
3541	Invertebrates	Parandania boecki	3.9500E-01	1.8	2.7658E-02	Makarieva et al. 2008
3542	Invertebrates	Paraphyllina ransoni	2.5000E-01	5	1.8450E-03	Makarieva et al. 2008
3543	Invertebrates	Paraponera clavata	1.9000E-01	25	7.7544E-02	Ehnes et al. 2011
3544	Invertebrates	Parasagitta elegans	2.0000E-02	4	5.4360E-04	Makarieva et al. 2008
3545	Invertebrates	Parasagitta euneritica	2.3000E-03	15	2.1445E-04	Makarieva et al. 2008
3546	Invertebrates	Parathelphusa hydrodromus	1.0000E+01	20	3.9186E-01	Makarieva et al. 2008

3547	Invertebrates	Parathelphusa hydrodromus	1.0000E+01	20	4.5954E-01	Makarieva et al. 2008
3548	Invertebrates	Parathemisto gaudichaudii	2.0000E-02	-0.8	2.6640E-03	Makarieva et al. 2008
3549	Invertebrates	Parathemisto gaudichaudii	7.0000E-02	-1	5.5440E-03	Makarieva et al. 2008
3550	Invertebrates	Parcoblatta sp.	7.3000E-02	25	1.6560E-02	Ehnes et al. 2011
3551	Invertebrates	Pardosa amenatata	5.5000E-02	9	4.0050E-03	Ehnes et al. 2011
3552	Invertebrates	Pardosa amenatata	5.5000E-02	3	1.2300E-03	Ehnes et al. 2011
3553	Invertebrates	Pardosa amenatata	5.5000E-02	13	5.8500E-03	Ehnes et al. 2011
3554	Invertebrates	Pardosa amenatata	5.5000E-02	14	6.7000E-03	Ehnes et al. 2011
3555	Invertebrates	Pardosa amenatata	5.5000E-02	10	4.2600E-03	Ehnes et al. 2011
3556	Invertebrates	Pardosa amenatata	5.5000E-02	10	4.6300E-03	Ehnes et al. 2011
3557	Invertebrates	Pardosa amenatata	5.5000E-02	4	1.8350E-03	Ehnes et al. 2011
3558	Invertebrates	Pardosa amenatata	5.5000E-02	5	2.3800E-03	Ehnes et al. 2011
3559	Invertebrates	Pardosa amenatata	5.5000E-02	7	2.4550E-03	Ehnes et al. 2011
3560	Invertebrates	Pardosa amenatata	5.5000E-02	15	7.0000E-03	Ehnes et al. 2011
3561	Invertebrates	Pardosa astrigera	3.9800E-02	25	1.3300E-02	Ehnes et al. 2011
3562	Invertebrates	Pardosa astrigera	4.3700E-02	25	1.9400E-02	Ehnes et al. 2011
3563	Invertebrates	Pardosa astrigera	5.2500E-02	25	2.2750E-02	Ehnes et al. 2011
3564	Invertebrates	Pardosa astrigera	5.2700E-02	25	2.4100E-02	Ehnes et al. 2011
3565	Invertebrates	Pardosa astrigera	6.0000E-02	20	1.1150E-02	Ehnes et al. 2011
3566	Invertebrates	Pardosa astrigera	6.0000E-02	30	2.7100E-02	Ehnes et al. 2011
3567	Invertebrates	Pardosa laura	3.0000E-02	25	2.2500E-02	Ehnes et al. 2011
3568	Invertebrates	Pardosa lugubris	8.0000E-03	8	2.0402E-03	Ehnes et al. 2011
3569	Invertebrates	Pardosa lugubris	8.0000E-03	15	4.2261E-03	Ehnes et al. 2011
3570	Invertebrates	Pardosa lugubris	8.0000E-03	22	6.7034E-03	Ehnes et al. 2011
3571	Invertebrates	Pardosa lugubris	1.0000E-02	8	1.8945E-03	Ehnes et al. 2011
3572	Invertebrates	Pardosa lugubris	1.0000E-02	8	1.6030E-03	Ehnes et al. 2011

3573	Invertebrates	Pardosa lugubris	1.0000E-02	15	4.0803E-03	Ehnes et al. 2011
3574	Invertebrates	Pardosa lugubris	1.0000E-02	15	2.3316E-03	Ehnes et al. 2011
3575	Invertebrates	Pardosa lugubris	1.0000E-02	15	7.1406E-03	Ehnes et al. 2011
3576	Invertebrates	Pardosa lugubris	1.0000E-02	22	7.5777E-03	Ehnes et al. 2011
3577	Invertebrates	Pardosa lugubris	1.0000E-02	22	8.7436E-03	Ehnes et al. 2011
3578	Invertebrates	Pardosa lugubris	1.1000E-02	15	3.6432E-03	Ehnes et al. 2011
3579	Invertebrates	Pardosa lugubris	1.1000E-02	15	4.2261E-03	Ehnes et al. 2011
3580	Invertebrates	Pardosa lugubris	1.1000E-02	22	8.1607E-03	Ehnes et al. 2011
3581	Invertebrates	Pardosa lugubris	1.1000E-02	22	9.9094E-03	Ehnes et al. 2011
3582	Invertebrates	Pardosa lugubris	1.2000E-02	15	4.3718E-03	Ehnes et al. 2011
3583	Invertebrates	Pardosa lugubris	1.2000E-02	15	3.3517E-03	Ehnes et al. 2011
3584	Invertebrates	Pardosa lugubris	1.2000E-02	22	8.4521E-03	Ehnes et al. 2011
3585	Invertebrates	Pardosa lugubris	1.2000E-02	22	8.5978E-03	Ehnes et al. 2011
3586	Invertebrates	Pardosa lugubris	1.3000E-02	15	4.5175E-03	Ehnes et al. 2011
3587	Invertebrates	Pardosa lugubris	1.3000E-02	22	7.8692E-03	Ehnes et al. 2011
3588	Invertebrates	Pardosa lugubris	1.6610E-02	29.9	8.0149E-03	Ehnes et al. 2011
3589	Invertebrates	Pardosa lugubris	1.7000E-02	8	2.6231E-03	Ehnes et al. 2011
3590	Invertebrates	Pardosa lugubris	1.7000E-02	15	3.9346E-03	Ehnes et al. 2011
3591	Invertebrates	Pardosa lugubris	1.7000E-02	22	8.8893E-03	Ehnes et al. 2011
3592	Invertebrates	Pardosa lugubris	1.8000E-02	15	4.5175E-03	Ehnes et al. 2011
3593	Invertebrates	Pardosa lugubris	1.8000E-02	22	9.6179E-03	Ehnes et al. 2011
3594	Invertebrates	Pardosa lugubris	2.0810E-02	29.9	1.0347E-02	Ehnes et al. 2011
3595	Invertebrates	Pardosa lugubris	2.1700E-02	20	3.6450E-03	Ehnes et al. 2011
3596	Invertebrates	Pardosa lugubris	2.2400E-02	20	3.9400E-03	Ehnes et al. 2011
3597	Invertebrates	Pardosa lugubris	2.5000E-02	15	5.1004E-03	Ehnes et al. 2011
3598	Invertebrates	Pardosa lugubris	2.5000E-02	22	8.3064E-03	Ehnes et al. 2011

3599	Invertebrates	Pardosa lugubris	2.7100E-02	11.5	1.7487E-03	Ehnes et al. 2011
3600	Invertebrates	Pardosa lugubris	2.7100E-02	18.5	3.9346E-03	Ehnes et al. 2011
3601	Invertebrates	Pardosa lugubris	2.9000E-02	8	8.7435E-04	Ehnes et al. 2011
3602	Invertebrates	Pardosa lugubris	2.9000E-02	8	8.7435E-04	Ehnes et al. 2011
3603	Invertebrates	Pardosa lugubris	2.9000E-02	15	4.0803E-03	Ehnes et al. 2011
3604	Invertebrates	Pardosa lugubris	2.9000E-02	15	5.5376E-03	Ehnes et al. 2011
3605	Invertebrates	Pardosa lugubris	2.9000E-02	22	1.8799E-02	Ehnes et al. 2011
3606	Invertebrates	Pardosa lugubris	2.9000E-02	22	1.0201E-02	Ehnes et al. 2011
3607	Invertebrates	Pardosa lugubris	2.9100E-02	11.5	2.6231E-03	Ehnes et al. 2011
3608	Invertebrates	Pardosa lugubris	2.9100E-02	18.5	6.9948E-03	Ehnes et al. 2011
3609	Invertebrates	Pardosa lugubris	3.0200E-02	11.5	3.7889E-03	Ehnes et al. 2011
3610	Invertebrates	Pardosa lugubris	3.0200E-02	18.5	7.7235E-03	Ehnes et al. 2011
3611	Invertebrates	Pardosa lugubris	3.0610E-02	29.9	2.0256E-02	Ehnes et al. 2011
3612	Invertebrates	Pardosa lugubris	4.0600E-02	11.5	6.8491E-03	Ehnes et al. 2011
3613	Invertebrates	Pardosa lugubris	4.0600E-02	18.5	9.9094E-03	Ehnes et al. 2011
3614	Invertebrates	Pardosa lugubris	4.7100E-02	11.5	5.8290E-03	Ehnes et al. 2011
3615	Invertebrates	Pardosa lugubris	4.7100E-02	18.5	1.0929E-02	Ehnes et al. 2011
3616	Invertebrates	Pardosa lugubris	4.9500E-02	11.5	6.9948E-03	Ehnes et al. 2011
3617	Invertebrates	Pardosa lugubris	4.9500E-02	18.5	9.4722E-03	Ehnes et al. 2011
3618	Invertebrates	Pardosa palustris	2.1000E-02	22	6.1205E-03	Ehnes et al. 2011
3619	Invertebrates	Pardosa palustris	2.2000E-02	8	3.3517E-03	Ehnes et al. 2011
3620	Invertebrates	Pardosa palustris	2.2000E-02	15	4.8090E-03	Ehnes et al. 2011
3621	Invertebrates	Pardosa palustris	2.2000E-02	15	2.1859E-03	Ehnes et al. 2011
3622	Invertebrates	Pardosa palustris	2.9000E-02	8	1.0201E-03	Ehnes et al. 2011
3623	Invertebrates	Pardosa palustris	2.9000E-02	15	3.2060E-03	Ehnes et al. 2011
3624	Invertebrates	Pardosa palustris	2.9000E-02	22	7.8692E-03	Ehnes et al. 2011

3625	Invertebrates	Pardosa palustris	3.0000E-02	8	1.6030E-03	Ehnes et al. 2011
3626	Invertebrates	Pardosa palustris	3.0000E-02	15	3.9346E-03	Ehnes et al. 2011
3627	Invertebrates	Pardosa palustris	3.0000E-02	22	8.0149E-03	Ehnes et al. 2011
3628	Invertebrates	Pardosa palustris	3.1000E-02	8	1.6030E-03	Ehnes et al. 2011
3629	Invertebrates	Pardosa palustris	3.1000E-02	8	5.8290E-04	Ehnes et al. 2011
3630	Invertebrates	Pardosa palustris	3.1000E-02	15	4.0803E-03	Ehnes et al. 2011
3631	Invertebrates	Pardosa palustris	3.1000E-02	15	4.3718E-03	Ehnes et al. 2011
3632	Invertebrates	Pardosa palustris	3.1000E-02	22	1.0784E-02	Ehnes et al. 2011
3633	Invertebrates	Pardosa palustris	3.1700E-02	11.5	6.5577E-03	Ehnes et al. 2011
3634	Invertebrates	Pardosa palustris	3.1700E-02	18.5	9.0350E-03	Ehnes et al. 2011
3635	Invertebrates	Pardosa palustris	3.1900E-02	11.5	7.2863E-03	Ehnes et al. 2011
3636	Invertebrates	Pardosa palustris	3.1900E-02	18.5	1.0347E-02	Ehnes et al. 2011
3637	Invertebrates	Pardosa palustris	3.4500E-02	11.5	5.9748E-03	Ehnes et al. 2011
3638	Invertebrates	Pardosa palustris	3.4500E-02	18.5	8.8893E-03	Ehnes et al. 2011
3639	Invertebrates	Pardosa palustris	3.4900E-02	11.5	7.5777E-03	Ehnes et al. 2011
3640	Invertebrates	Pardosa palustris	3.4900E-02	18.5	9.3265E-03	Ehnes et al. 2011
3641	Invertebrates	Pardosa palustris	3.8000E-02	8	2.7688E-03	Ehnes et al. 2011
3642	Invertebrates	Pardosa palustris	3.8000E-02	15	4.6632E-03	Ehnes et al. 2011
3643	Invertebrates	Pardosa palustris	3.8000E-02	22	8.0149E-03	Ehnes et al. 2011
3644	Invertebrates	Pardosa pseudoannulata	1.0000E-01	29	3.1250E-02	Ehnes et al. 2011
3645	Invertebrates	Pardosa pullata	9.0000E-03	20	1.5450E-03	Ehnes et al. 2011
3646	Invertebrates	Pardosa pullata	1.6200E-02	20	7.2000E-03	Ehnes et al. 2011
3647	Invertebrates	Parisotoma notabilis	1.0000E-05	6	4.9500E-06	Ehnes et al. 2011
3648	Invertebrates	Parisotoma notabilis	1.0000E-05	10	7.4000E-06	Ehnes et al. 2011
3649	Invertebrates	Parisotoma notabilis	1.0000E-05	15	9.7000E-06	Ehnes et al. 2011
3650	Invertebrates	Parisotoma octooculata	4.3600E-06	5	2.1000E-06	Ehnes et al. 2011

3651	Invertebrates	Parisotoma octooculata	1.1070E-05	5	4.0000E-06	Ehnes et al. 2011
3652	Invertebrates	Parisotoma octooculata	2.6270E-05	5	7.2500E-06	Ehnes et al. 2011
3653	Invertebrates	Parisotoma octooculata	4.6940E-05	5	1.1050E-05	Ehnes et al. 2011
3654	Invertebrates	Pasiphaea chacei	1.7300E+00	7.5	1.3141E-01	Makarieva et al. 2008
3655	Invertebrates	Pasiphaea chacei	1.7300E+00	7.5	2.2327E-01	Makarieva et al. 2008
3656	Invertebrates	Pasiphaea emarginata	1.1070E+01	5.5	1.3948E-01	Makarieva et al. 2008
3657	Invertebrates	Pasiphaea emarginata	1.1070E+01	5.5	1.4347E+00	Makarieva et al. 2008
3658	Invertebrates	Pasiphaea pacifica	1.9100E+00	7.5	1.4096E-01	Makarieva et al. 2008
3659	Invertebrates	Pasiphaea pacifica	1.9100E+00	7.5	1.8565E-01	Makarieva et al. 2008
3660	Invertebrates	Pasiphaea scotiae	6.1000E-01	0.3	2.6352E-02	Makarieva et al. 2008
3661	Invertebrates	Pasiphaea scotiae	1.2600E+00	0.2	5.4432E-02	Makarieva et al. 2008
3662	Invertebrates	Pelegrina galathea	8.3000E-03	20	1.7600E-03	Ehnes et al. 2011
3663	Invertebrates	Pelidnota sp.	1.0915E+00	25	2.7207E-01	Ehnes et al. 2011
3664	Invertebrates	Penaeus sp.	1.0300E-01	12.2	2.2971E-02	Makarieva et al. 2008
3665	Invertebrates	Penilia avirostris	4.0000E-05	20	1.5840E-05	Makarieva et al. 2008
3666	Invertebrates	Pergamasus sp.	5.8200E-04	10	1.0725E-04	Ehnes et al. 2011
3667	Invertebrates	Pericapritermes nitobei	1.2000E-03	25	2.1600E-04	Ehnes et al. 2011
3668	Invertebrates	Pericapritermes semarangi	1.3000E-03	25	1.4400E-04	Ehnes et al. 2011
3669	Invertebrates	Perigonia lusca	5.5833E-01	25	4.0774E-01	Ehnes et al. 2011
3670	Invertebrates	Perimylops antarcticus	1.4500E-02	25	8.7480E-03	Ehnes et al. 2011
3671	Invertebrates	Periphylla peryphylla	1.1000E+01	5	2.3760E-02	Makarieva et al. 2008
3672	Invertebrates	Periplaneta americana	9.0000E-01	25	1.7629E-01	Ehnes et al. 2011
3673	Invertebrates	Periplaneta orientalis	1.6500E-01	25	5.5422E-02	Ehnes et al. 2011
3674	Invertebrates	Perisphaeria sp.	3.2410E-01	25	3.5424E-02	Ehnes et al. 2011
3675	Invertebrates	Peristeptus sp.	5.5000E-02	25	9.8100E-03	Ehnes et al. 2011
3676	Invertebrates	Perithemis tenera	6.1000E-02	25	5.9598E-02	Ehnes et al. 2011

3677	Invertebrates	Phidippus audax	1.7100E-01	20	3.2500E-02	Ehnes et al. 2011
3678	Invertebrates	Phidippus clarus	2.6000E-01	20	4.6450E-02	Ehnes et al. 2011
3679	Invertebrates	Phidippus johnsoni	1.7300E-01	22	2.3150E-02	Ehnes et al. 2011
3680	Invertebrates	Phidippus otiosus	3.3700E-01	20	4.8500E-02	Ehnes et al. 2011
3681	Invertebrates	Phidippus pulcherrimus	1.0400E-01	20	1.7800E-02	Ehnes et al. 2011
3682	Invertebrates	Phidippus regius	5.6800E-01	10	2.4650E-02	Ehnes et al. 2011
3683	Invertebrates	Phidippus regius	5.6800E-01	20	5.0500E-02	Ehnes et al. 2011
3684	Invertebrates	Phidippus regius	5.6800E-01	30	1.3600E-01	Ehnes et al. 2011
3685	Invertebrates	Philaenus spumarius	3.9300E-03	25	1.0710E-02	Ehnes et al. 2011
3686	Invertebrates	Phileurus sp.	3.5010E+00	25	2.8127E-01	Ehnes et al. 2011
3687	Invertebrates	Philonthus	1.5600E-02	11.5	3.7889E-03	Ehnes et al. 2011
3688	Invertebrates	Philonthus	1.6300E-02	8	1.3116E-03	Ehnes et al. 2011
3689	Invertebrates	Philonthus	1.6600E-02	15	4.3718E-03	Ehnes et al. 2011
3690	Invertebrates	Philonthus	1.7000E-02	22	1.4427E-02	Ehnes et al. 2011
3691	Invertebrates	Philonthus	1.7600E-02	8	2.9145E-03	Ehnes et al. 2011
3692	Invertebrates	Philonthus	1.8400E-02	15	7.5777E-03	Ehnes et al. 2011
3693	Invertebrates	Philonthus	1.9200E-02	22	1.8070E-02	Ehnes et al. 2011
3694	Invertebrates	Philonthus	2.1100E-02	11.5	5.3919E-03	Ehnes et al. 2011
3695	Invertebrates	Philonthus	2.1800E-02	8	2.4774E-03	Ehnes et al. 2011
3696	Invertebrates	Philonthus	2.2200E-02	15	6.9948E-03	Ehnes et al. 2011
3697	Invertebrates	Philonthus	2.2300E-02	22	1.6904E-02	Ehnes et al. 2011
3698	Invertebrates	Philoscia	6.0900E-03	15.8	1.8945E-03	Ehnes et al. 2011
3699	Invertebrates	Philoscia	1.1460E-02	15.8	2.9145E-03	Ehnes et al. 2011
3700	Invertebrates	Philoscia muscorum	2.0000E-02	19	4.7250E-03	Ehnes et al. 2011
3701	Invertebrates	Philosciidae	4.5800E-02	25	1.1350E-02	Ehnes et al. 2011
3702	Invertebrates	Phormia regina	5.0000E-02	25	9.7470E-02	Ehnes et al. 2011

3703	Invertebrates	Phorocantha recurva	2.6000E-01	25	1.4404E-01	Ehnes et al. 2011
3704	Invertebrates	Phorocantha semipunctata	3.0000E-01	25	1.2631E-01	Ehnes et al. 2011
3705	Invertebrates	Phronima sedentaria	8.6000E-02	10	1.1146E-03	Makarieva et al. 2008
3706	Invertebrates	Phronima sedentaria	8.6000E-02	10	5.2477E-03	Makarieva et al. 2008
3707	Invertebrates	Phronima sp.	1.4500E-01	28.75	3.8419E-02	Makarieva et al. 2008
3708	Invertebrates	Phrynocolus auriculatus	1.9300E-01	25	3.0438E-02	Ehnes et al. 2011
3709	Invertebrates	Phrynocolus petrosus	1.1090E+00	25	6.4026E-02	Ehnes et al. 2011
3710	Invertebrates	Phrynocolus sp.	2.0400E-01	25	3.4452E-02	Ehnes et al. 2011
3711	Invertebrates	Phthiracarus piger	3.2000E-04	18	2.4550E-05	Ehnes et al. 2011
3712	Invertebrates	Phthiracarus piger	3.5000E-04	18	2.6850E-05	Ehnes et al. 2011
3713	Invertebrates	Phthiracarus sp.	5.8100E-05	10	5.6500E-06	Ehnes et al. 2011
3714	Invertebrates	Phthiracarus sp.	2.0300E-04	10	1.2750E-05	Ehnes et al. 2011
3715	Invertebrates	Physadesmia globosa	5.1600E-01	25	1.0793E-01	Ehnes et al. 2011
3716	Invertebrates	Physosterna cribripes	1.2260E+00	25	4.3987E-01	Ehnes et al. 2011
3717	Invertebrates	Phytocoris nigripubescens	1.2700E-03	25	5.5440E-03	Ehnes et al. 2011
3718	Invertebrates	Phytoseiulus persimilis	8.5900E-06	25	1.2500E-05	Ehnes et al. 2011
3719	Invertebrates	Phytoseiulus persimilis	1.3090E-05	25	1.1200E-05	Ehnes et al. 2011
3720	Invertebrates	Pilica formidolosa	2.0000E-01	25	1.4216E-01	Ehnes et al. 2011
3721	Invertebrates	Pilogalumna allifera	2.7200E-05	7	9.0000E-07	Ehnes et al. 2011
3722	Invertebrates	Pilogalumna allifera	2.7200E-05	11	1.2000E-06	Ehnes et al. 2011
3723	Invertebrates	Pilogalumna allifera	2.7200E-05	15	2.0500E-06	Ehnes et al. 2011
3724	Invertebrates	Pilogalumna allifera	2.7200E-05	19	3.5000E-06	Ehnes et al. 2011
3725	Invertebrates	Pilogalumna allifera	2.7200E-05	24	3.7500E-06	Ehnes et al. 2011
3726	Invertebrates	Pilogalumna allifera	2.7200E-05	29	4.1500E-06	Ehnes et al. 2011
3727	Invertebrates	Pimelia cenchronota	1.4740E+00	25	1.1176E-01	Ehnes et al. 2011
3728	Invertebrates	Pimelia grandis	2.0980E+00	25	6.7214E-01	Ehnes et al. 2011

3729	Invertebrates	Pimelia obsoleta	8.6100E-01	25	5.4203E-01	Ehnes et al. 2011
3730	Invertebrates	Pirata latitans	9.0000E-03	20	4.1100E-03	Ehnes et al. 2011
3731	Invertebrates	Pirata latitans	9.0000E-03	20	4.7150E-03	Ehnes et al. 2011
3732	Invertebrates	Pirata latitans	1.8000E-02	8	1.1658E-03	Ehnes et al. 2011
3733	Invertebrates	Pirata latitans	1.8000E-02	15	2.3316E-03	Ehnes et al. 2011
3734	Invertebrates	Pirata latitans	1.9000E-02	8	2.7688E-03	Ehnes et al. 2011
3735	Invertebrates	Pirata latitans	1.9000E-02	15	1.8945E-03	Ehnes et al. 2011
3736	Invertebrates	Pirata latitans	2.0000E-02	8	1.3116E-03	Ehnes et al. 2011
3737	Invertebrates	Pirata latitans	2.0000E-02	15	2.4774E-03	Ehnes et al. 2011
3738	Invertebrates	Pirata latitans	2.0000E-02	22	8.8893E-03	Ehnes et al. 2011
3739	Invertebrates	Pirata latitans	2.1000E-02	15	2.1859E-03	Ehnes et al. 2011
3740	Invertebrates	Pirata latitans	2.1000E-02	22	6.4119E-03	Ehnes et al. 2011
3741	Invertebrates	Pirata latitans	2.2000E-02	22	8.0149E-03	Ehnes et al. 2011
3742	Invertebrates	Pirata latitans	2.3000E-02	8	3.0603E-03	Ehnes et al. 2011
3743	Invertebrates	Pirata latitans	2.3000E-02	15	5.9748E-03	Ehnes et al. 2011
3744	Invertebrates	Pirata latitans	2.3000E-02	22	1.6904E-02	Ehnes et al. 2011
3745	Invertebrates	Pirata latitans	2.6000E-02	8	8.7435E-04	Ehnes et al. 2011
3746	Invertebrates	Pirata latitans	2.6000E-02	15	3.0603E-03	Ehnes et al. 2011
3747	Invertebrates	Pirata latitans	2.6000E-02	22	6.7034E-03	Ehnes et al. 2011
3748	Invertebrates	Pirata latitans	2.7000E-02	15	4.2261E-03	Ehnes et al. 2011
3749	Invertebrates	Pirata latitans	2.7000E-02	22	9.6179E-03	Ehnes et al. 2011
3750	Invertebrates	Pirata latitans	2.7900E-02	11.5	4.3718E-03	Ehnes et al. 2011
3751	Invertebrates	Pirata latitans	2.7900E-02	18.5	7.8692E-03	Ehnes et al. 2011
3752	Invertebrates	Pirata latitans	3.1200E-02	11.5	5.6833E-03	Ehnes et al. 2011
3753	Invertebrates	Pirata latitans	3.1200E-02	18.5	1.0347E-02	Ehnes et al. 2011
3754	Invertebrates	Pirata latitans	3.2800E-02	11.5	5.3919E-03	Ehnes et al. 2011

3755	Invertebrates	Pirata latitans	3.2800E-02	18.5	9.0350E-03	Ehnes et al. 2011
3756	Invertebrates	Pirata latitans	4.0100E-02	11.5	5.2461E-03	Ehnes et al. 2011
3757	Invertebrates	Pirata latitans	4.0100E-02	18.5	7.8692E-03	Ehnes et al. 2011
3758	Invertebrates	Pirata latitans	4.1200E-02	11.5	3.6432E-03	Ehnes et al. 2011
3759	Invertebrates	Pirata latitans	4.1200E-02	18.5	8.7436E-03	Ehnes et al. 2011
3760	Invertebrates	Pirata latitans	5.1800E-02	11.5	8.1607E-03	Ehnes et al. 2011
3761	Invertebrates	Pirata latitans	5.1800E-02	18.5	9.4722E-03	Ehnes et al. 2011
3762	Invertebrates	Pisaura mirabilis	6.5000E-02	15	1.2387E-02	Ehnes et al. 2011
3763	Invertebrates	Pisaura mirabilis	6.5000E-02	22	2.1276E-02	Ehnes et al. 2011
3764	Invertebrates	Pisaura mirabilis	6.8000E-02	8	3.7889E-03	Ehnes et al. 2011
3765	Invertebrates	Pisaura mirabilis	6.8000E-02	15	2.0402E-03	Ehnes et al. 2011
3766	Invertebrates	Pisaura mirabilis	6.8000E-02	22	2.0984E-02	Ehnes et al. 2011
3767	Invertebrates	Pisaura mirabilis	7.7000E-02	8	4.3718E-03	Ehnes et al. 2011
3768	Invertebrates	Pisaura mirabilis	7.7000E-02	15	9.6179E-03	Ehnes et al. 2011
3769	Invertebrates	Pisaura mirabilis	7.7000E-02	22	2.3025E-02	Ehnes et al. 2011
3770	Invertebrates	Pisaura mirabilis	7.9000E-02	8	3.7889E-03	Ehnes et al. 2011
3771	Invertebrates	Pisaura mirabilis	7.9000E-02	15	8.0149E-03	Ehnes et al. 2011
3772	Invertebrates	Pisaura mirabilis	7.9000E-02	22	2.1859E-02	Ehnes et al. 2011
3773	Invertebrates	Pisaura mirabilis	8.5000E-02	8	4.5175E-03	Ehnes et al. 2011
3774	Invertebrates	Pisaura mirabilis	8.5000E-02	15	9.0350E-03	Ehnes et al. 2011
3775	Invertebrates	Pisaura mirabilis	8.5000E-02	22	1.9819E-02	Ehnes et al. 2011
3776	Invertebrates	Pisaura mirabilis	8.6000E-02	8	5.9748E-03	Ehnes et al. 2011
3777	Invertebrates	Pisaura mirabilis	8.6000E-02	15	1.1367E-02	Ehnes et al. 2011
3778	Invertebrates	Pisaura mirabilis	8.6000E-02	22	1.8216E-02	Ehnes et al. 2011
3779	Invertebrates	Pisaura mirabilis	1.0400E-01	8	4.0803E-03	Ehnes et al. 2011
3780	Invertebrates	Pisaura mirabilis	1.0400E-01	15	9.0350E-03	Ehnes et al. 2011

3781	Invertebrates	Pisaura mirabilis	1.0400E-01	22	1.3990E-02	Ehnes et al. 2011
3782	Invertebrates	Pisaura mirabilis	1.0900E-01	8	1.0784E-02	Ehnes et al. 2011
3783	Invertebrates	Pisaura mirabilis	1.0900E-01	15	1.9964E-02	Ehnes et al. 2011
3784	Invertebrates	Pisaura mirabilis	1.0900E-01	22	4.6924E-02	Ehnes et al. 2011
3785	Invertebrates	Pisaura mirabilis	1.1400E-01	8	7.7235E-03	Ehnes et al. 2011
3786	Invertebrates	Pisaura mirabilis	1.1400E-01	15	2.2296E-02	Ehnes et al. 2011
3787	Invertebrates	Pisaura mirabilis	1.1400E-01	22	3.5411E-02	Ehnes et al. 2011
3788	Invertebrates	Pisaura mirabilis	1.1600E-01	8	1.4864E-02	Ehnes et al. 2011
3789	Invertebrates	Pisaura mirabilis	1.1600E-01	15	3.1040E-02	Ehnes et al. 2011
3790	Invertebrates	Pisaura mirabilis	1.1600E-01	15	1.0201E-02	Ehnes et al. 2011
3791	Invertebrates	Pisaura mirabilis	1.1600E-01	22	4.1532E-02	Ehnes et al. 2011
3792	Invertebrates	Pisaura mirabilis	1.2250E-01	11.5	1.4573E-02	Ehnes et al. 2011
3793	Invertebrates	Pisaura mirabilis	1.2250E-01	18.5	1.8507E-02	Ehnes et al. 2011
3794	Invertebrates	Pisaura mirabilis	1.2810E-01	11.5	1.2532E-02	Ehnes et al. 2011
3795	Invertebrates	Pisaura mirabilis	1.2810E-01	18.5	1.3698E-02	Ehnes et al. 2011
3796	Invertebrates	Pisaura mirabilis	1.4600E-01	15	3.8909E-02	Ehnes et al. 2011
3797	Invertebrates	Platynothrus peltifer	1.4500E-05	15	7.9500E-06	Ehnes et al. 2011
3798	Invertebrates	Platynothrus peltifer	3.7200E-05	15	1.5850E-05	Ehnes et al. 2011
3799	Invertebrates	Platynothrus peltifer	5.9800E-05	15	1.8900E-05	Ehnes et al. 2011
3800	Invertebrates	Platynothrus peltifer	6.2900E-05	0	1.0500E-06	Ehnes et al. 2011
3801	Invertebrates	Platynothrus peltifer	6.2900E-05	5	3.7500E-06	Ehnes et al. 2011
3802	Invertebrates	Platynothrus peltifer	6.2900E-05	10	6.5000E-06	Ehnes et al. 2011
3803	Invertebrates	Platynothrus peltifer	6.2900E-05	15	1.0450E-05	Ehnes et al. 2011
3804	Invertebrates	Platynothrus peltifer	6.2900E-05	25	1.5300E-05	Ehnes et al. 2011
3805	Invertebrates	Platynus dorsalis	9.0700E-03	30	1.3844E-02	Ehnes et al. 2011
3806	Invertebrates	Platynus dorsalis	9.2900E-03	5	2.6231E-03	Ehnes et al. 2011

3807	Invertebrates	Platynus dorsalis	9.3000E-03	22	8.8893E-03	Ehnes et al. 2011
3808	Invertebrates	Platynus dorsalis	9.4000E-03	18.5	4.5175E-03	Ehnes et al. 2011
3809	Invertebrates	Platynus dorsalis	9.8000E-03	22	6.8491E-03	Ehnes et al. 2011
3810	Invertebrates	Platynus dorsalis	9.9000E-03	20	1.8945E-03	Ehnes et al. 2011
3811	Invertebrates	Platynus dorsalis	9.9000E-03	11.5	4.2261E-03	Ehnes et al. 2011
3812	Invertebrates	Platynus dorsalis	9.9200E-03	10	2.7688E-03	Ehnes et al. 2011
3813	Invertebrates	Platynus dorsalis	1.0000E-02	22	4.5175E-03	Ehnes et al. 2011
3814	Invertebrates	Platynus dorsalis	1.0190E-02	20	8.1607E-03	Ehnes et al. 2011
3815	Invertebrates	Platynus dorsalis	1.0300E-02	15	5.9748E-03	Ehnes et al. 2011
3816	Invertebrates	Platynus dorsalis	1.0420E-02	25	1.4718E-02	Ehnes et al. 2011
3817	Invertebrates	Platynus dorsalis	1.0490E-02	25	1.1367E-02	Ehnes et al. 2011
3818	Invertebrates	Platynus dorsalis	1.0600E-02	11.5	3.0603E-03	Ehnes et al. 2011
3819	Invertebrates	Platynus dorsalis	1.0620E-02	30	8.1607E-03	Ehnes et al. 2011
3820	Invertebrates	Platynus dorsalis	1.0700E-02	8	1.8945E-03	Ehnes et al. 2011
3821	Invertebrates	Platynus dorsalis	1.0820E-02	30	8.0149E-03	Ehnes et al. 2011
3822	Invertebrates	Platynus dorsalis	1.0900E-02	11.5	3.2060E-03	Ehnes et al. 2011
3823	Invertebrates	Platynus dorsalis	1.0970E-02	15	3.7889E-03	Ehnes et al. 2011
3824	Invertebrates	Platynus dorsalis	1.1000E-02	15	6.4119E-03	Ehnes et al. 2011
3825	Invertebrates	Platynus dorsalis	1.1090E-02	20	5.3919E-03	Ehnes et al. 2011
3826	Invertebrates	Platynus dorsalis	1.1200E-02	8	2.1859E-03	Ehnes et al. 2011
3827	Invertebrates	Platynus dorsalis	1.1200E-02	18.5	5.9748E-03	Ehnes et al. 2011
3828	Invertebrates	Platynus dorsalis	1.1330E-02	20	4.8090E-03	Ehnes et al. 2011
3829	Invertebrates	Platynus dorsalis	1.1340E-02	15	3.7889E-03	Ehnes et al. 2011
3830	Invertebrates	Platynus dorsalis	1.1490E-02	15	4.2261E-03	Ehnes et al. 2011
3831	Invertebrates	Platynus dorsalis	1.1660E-02	5	1.4575E-04	Ehnes et al. 2011
3832	Invertebrates	Platynus dorsalis	1.1670E-02	25	9.3265E-03	Ehnes et al. 2011

3833	Invertebrates	Platynus dorsalis	1.1700E-02	22	1.2532E-02	Ehnes et al. 2011
3834	Invertebrates	Platynus dorsalis	1.1790E-02	20	6.4119E-03	Ehnes et al. 2011
3835	Invertebrates	Platynus dorsalis	1.1900E-02	5	2.3316E-03	Ehnes et al. 2011
3836	Invertebrates	Platynus dorsalis	1.2000E-02	22	9.0350E-03	Ehnes et al. 2011
3837	Invertebrates	Platynus dorsalis	1.2040E-02	15	5.3919E-03	Ehnes et al. 2011
3838	Invertebrates	Platynus dorsalis	1.2050E-02	20	7.2863E-03	Ehnes et al. 2011
3839	Invertebrates	Platynus dorsalis	1.2100E-02	15	5.3919E-03	Ehnes et al. 2011
3840	Invertebrates	Platynus dorsalis	1.2100E-02	22	1.1221E-02	Ehnes et al. 2011
3841	Invertebrates	Platynus dorsalis	1.2160E-02	5	1.7487E-03	Ehnes et al. 2011
3842	Invertebrates	Platynus dorsalis	1.2200E-02	18.5	6.1205E-03	Ehnes et al. 2011
3843	Invertebrates	Platynus dorsalis	1.2330E-02	10	2.3316E-03	Ehnes et al. 2011
3844	Invertebrates	Platynus dorsalis	1.2400E-02	11.5	3.0603E-03	Ehnes et al. 2011
3845	Invertebrates	Platynus dorsalis	1.2420E-02	30	1.8361E-02	Ehnes et al. 2011
3846	Invertebrates	Platynus dorsalis	1.2420E-02	30	1.6467E-02	Ehnes et al. 2011
3847	Invertebrates	Platynus dorsalis	1.2760E-02	25	1.2678E-02	Ehnes et al. 2011
3848	Invertebrates	Platynus dorsalis	1.2800E-02	8	1.4573E-03	Ehnes et al. 2011
3849	Invertebrates	Platynus dorsalis	1.2850E-02	20	7.1406E-03	Ehnes et al. 2011
3850	Invertebrates	Platynus dorsalis	1.3000E-02	11.5	5.5376E-03	Ehnes et al. 2011
3851	Invertebrates	Platynus dorsalis	1.3070E-02	30	1.0055E-02	Ehnes et al. 2011
3852	Invertebrates	Platynus dorsalis	1.3100E-02	11.5	3.7889E-03	Ehnes et al. 2011
3853	Invertebrates	Platynus dorsalis	1.3100E-02	18.5	5.6833E-03	Ehnes et al. 2011
3854	Invertebrates	Platynus dorsalis	1.3110E-02	25	1.2387E-02	Ehnes et al. 2011
3855	Invertebrates	Platynus dorsalis	1.3300E-02	8	1.6030E-03	Ehnes et al. 2011
3856	Invertebrates	Platynus dorsalis	1.3300E-02	8	4.0803E-03	Ehnes et al. 2011
3857	Invertebrates	Platynus dorsalis	1.3300E-02	15	8.1607E-03	Ehnes et al. 2011
3858	Invertebrates	Platynus dorsalis	1.3300E-02	15	6.2662E-03	Ehnes et al. 2011

3859	Invertebrates	Platynus dorsalis	1.3320E-02	25	9.0350E-03	Ehnes et al. 2011
3860	Invertebrates	Platynus dorsalis	1.3400E-02	10	2.4774E-03	Ehnes et al. 2011
3861	Invertebrates	Platynus dorsalis	1.3500E-02	15	5.9748E-03	Ehnes et al. 2011
3862	Invertebrates	Platynus dorsalis	1.3600E-02	11.5	3.4974E-03	Ehnes et al. 2011
3863	Invertebrates	Platynus dorsalis	1.3700E-02	22	1.1512E-02	Ehnes et al. 2011
3864	Invertebrates	Platynus dorsalis	1.3870E-02	10	4.0803E-03	Ehnes et al. 2011
3865	Invertebrates	Platynus dorsalis	1.3980E-02	5	1.4573E-03	Ehnes et al. 2011
3866	Invertebrates	Platynus dorsalis	1.4020E-02	10	2.3316E-03	Ehnes et al. 2011
3867	Invertebrates	Platynus dorsalis	1.4070E-02	10	1.4573E-03	Ehnes et al. 2011
3868	Invertebrates	Platynus dorsalis	1.4200E-02	15	5.3919E-03	Ehnes et al. 2011
3869	Invertebrates	Platynus dorsalis	1.4200E-02	22	7.8692E-03	Ehnes et al. 2011
3870	Invertebrates	Platynus dorsalis	1.4280E-02	20	7.2863E-03	Ehnes et al. 2011
3871	Invertebrates	Platynus dorsalis	1.4310E-02	10	1.6030E-03	Ehnes et al. 2011
3872	Invertebrates	Platynus dorsalis	1.4330E-02	25	1.4281E-02	Ehnes et al. 2011
3873	Invertebrates	Platynus dorsalis	1.4390E-02	30	1.8361E-02	Ehnes et al. 2011
3874	Invertebrates	Platynus dorsalis	1.4810E-02	10	4.3720E-04	Ehnes et al. 2011
3875	Invertebrates	Platynus dorsalis	1.5120E-02	25	1.0492E-02	Ehnes et al. 2011
3876	Invertebrates	Platynus dorsalis	1.5310E-02	5	1.1658E-03	Ehnes et al. 2011
3877	Invertebrates	Platynus dorsalis	1.5380E-02	5	8.7435E-04	Ehnes et al. 2011
3878	Invertebrates	Platynus dorsalis	1.5430E-02	15	8.1607E-03	Ehnes et al. 2011
3879	Invertebrates	Platynus dorsalis	1.5700E-02	25	1.5884E-02	Ehnes et al. 2011
3880	Invertebrates	Platynus dorsalis	1.5800E-02	11.5	4.6632E-03	Ehnes et al. 2011
3881	Invertebrates	Platynus dorsalis	1.6000E-02	30	1.1658E-02	Ehnes et al. 2011
3882	Invertebrates	Platynus dorsalis	1.6000E-02	18.5	7.7235E-03	Ehnes et al. 2011
3883	Invertebrates	Platynus dorsalis	1.6070E-02	20	5.6833E-03	Ehnes et al. 2011
3884	Invertebrates	Platynus dorsalis	1.6120E-02	5	4.3720E-04	Ehnes et al. 2011

3885	Invertebrates	Platynus dorsalis	1.6200E-02	11.5	6.4119E-03	Ehnes et al. 2011
3886	Invertebrates	Platynus dorsalis	1.6400E-02	30	3.5703E-02	Ehnes et al. 2011
3887	Invertebrates	Platynus dorsalis	1.6400E-02	25	1.9236E-02	Ehnes et al. 2011
3888	Invertebrates	Platynus dorsalis	1.6400E-02	18.5	9.4722E-03	Ehnes et al. 2011
3889	Invertebrates	Platynus dorsalis	1.6500E-02	22	1.2095E-02	Ehnes et al. 2011
3890	Invertebrates	Platynus dorsalis	1.6680E-02	5	2.9145E-04	Ehnes et al. 2011
3891	Invertebrates	Platynus dorsalis	1.6700E-02	8	2.1859E-03	Ehnes et al. 2011
3892	Invertebrates	Platynus dorsalis	1.6900E-02	22	1.9964E-02	Ehnes et al. 2011
3893	Invertebrates	Platynus dorsalis	1.7000E-02	22	1.2241E-02	Ehnes et al. 2011
3894	Invertebrates	Platynus dorsalis	1.7100E-02	15	9.3265E-03	Ehnes et al. 2011
3895	Invertebrates	Platynus dorsalis	1.7290E-02	30	2.2150E-02	Ehnes et al. 2011
3896	Invertebrates	Platynus dorsalis	1.7300E-02	22	1.7341E-02	Ehnes et al. 2011
3897	Invertebrates	Platynus dorsalis	1.7550E-02	10	3.3517E-03	Ehnes et al. 2011
3898	Invertebrates	Platynus dorsalis	1.7800E-02	8	3.2060E-03	Ehnes et al. 2011
3899	Invertebrates	Platynus dorsalis	1.8140E-02	5	1.4573E-03	Ehnes et al. 2011
3900	Invertebrates	Platynus dorsalis	1.8450E-02	5	1.7487E-03	Ehnes et al. 2011
3901	Invertebrates	Platynus dorsalis	1.9200E-02	22	1.2824E-02	Ehnes et al. 2011
3902	Invertebrates	Platynus dorsalis	2.2000E-02	15	1.1804E-02	Ehnes et al. 2011
3903	Invertebrates	Pleocoma australis	9.4000E-01	25	5.8298E-01	Ehnes et al. 2011
3904	Invertebrates	Plesionika sp.	1.9200E+00	5.5	1.8317E-01	Makarieva et al. 2008
3905	Invertebrates	Plesionika sp.	1.9200E+00	5.5	3.6979E-01	Makarieva et al. 2008
3906	Invertebrates	Pleuromamma abdominalis	4.1500E-04	15	3.7402E-04	Makarieva et al. 2008
3907	Invertebrates	Pleuromamma abdominalis	8.0000E-04	20	1.0305E-03	Makarieva et al. 2008
3908	Invertebrates	Pleuromamma abdominalis	1.2300E-03	20	7.6627E-04	Makarieva et al. 2008
3909	Invertebrates	Pleuromamma gracilis	1.8750E-04	15	1.8998E-04	Makarieva et al. 2008
3910	Invertebrates	Pleuromamma gracilis	2.7000E-04	20	1.8901E-04	Makarieva et al. 2008

3911	Invertebrates	Pleuromamma robusta	1.4000E-03	8	6.3000E-04	Makarieva et al. 2008
3912	Invertebrates	Pleuromamma robusta	1.4000E-03	8	7.8120E-04	Makarieva et al. 2008
3913	Invertebrates	Pleuroncodes planipes	1.9000E+00	15	1.9152E-01	Makarieva et al. 2008
3914	Invertebrates	Podon polyphemoides	1.2000E-04	20	6.2640E-05	Makarieva et al. 2008
3915	Invertebrates	Poecilus	4.0300E-02	11.5	6.2662E-03	Ehnes et al. 2011
3916	Invertebrates	Poecilus	4.2500E-02	8	9.9094E-03	Ehnes et al. 2011
3917	Invertebrates	Poecilus	4.2900E-02	11.5	4.9547E-03	Ehnes et al. 2011
3918	Invertebrates	Poecilus	4.4800E-02	8	4.2261E-03	Ehnes et al. 2011
3919	Invertebrates	Poecilus	4.5000E-02	18.5	2.6814E-02	Ehnes et al. 2011
3920	Invertebrates	Poecilus	4.6100E-02	15	1.1367E-02	Ehnes et al. 2011
3921	Invertebrates	Poecilus	4.6200E-02	18.5	1.9964E-02	Ehnes et al. 2011
3922	Invertebrates	Poecilus	4.9800E-02	8	5.6833E-03	Ehnes et al. 2011
3923	Invertebrates	Poecilus	5.1900E-02	15	1.4281E-02	Ehnes et al. 2011
3924	Invertebrates	Poecilus	5.2800E-02	18.5	2.8708E-02	Ehnes et al. 2011
3925	Invertebrates	Poecilus	5.3000E-02	11.5	1.4864E-02	Ehnes et al. 2011
3926	Invertebrates	Poecilus	5.3100E-02	18.5	1.8070E-02	Ehnes et al. 2011
3927	Invertebrates	Poecilus	5.3400E-02	8	5.2461E-03	Ehnes et al. 2011
3928	Invertebrates	Poecilus	5.5000E-02	11.5	6.5577E-03	Ehnes et al. 2011
3929	Invertebrates	Poecilus	5.5100E-02	15	8.0149E-03	Ehnes et al. 2011
3930	Invertebrates	Poecilus	5.5700E-02	22	3.4537E-02	Ehnes et al. 2011
3931	Invertebrates	Poecilus	5.6100E-02	18.5	2.5648E-02	Ehnes et al. 2011
3932	Invertebrates	Poecilus	5.6600E-02	15	8.5978E-03	Ehnes et al. 2011
3933	Invertebrates	Poecilus	5.7200E-02	22	3.4974E-02	Ehnes et al. 2011
3934	Invertebrates	Poecilus	5.8100E-02	22	3.5411E-02	Ehnes et al. 2011
3935	Invertebrates	Poecilus	5.8500E-02	8	5.6833E-03	Ehnes et al. 2011
3936	Invertebrates	Poecilus	5.8500E-02	18.5	1.6758E-02	Ehnes et al. 2011

3937	Invertebrates	Poecilus	5.9100E-02	11.5	6.4119E-03	Ehnes et al. 2011
3938	Invertebrates	Poecilus	5.9800E-02	8	5.8290E-03	Ehnes et al. 2011
3939	Invertebrates	Poecilus	5.9800E-02	22	4.2260E-02	Ehnes et al. 2011
3940	Invertebrates	Poecilus	5.9900E-02	22	2.2005E-02	Ehnes et al. 2011
3941	Invertebrates	Poecilus	6.0800E-02	15	1.4281E-02	Ehnes et al. 2011
3942	Invertebrates	Poecilus	6.1500E-02	8	6.7034E-03	Ehnes et al. 2011
3943	Invertebrates	Poecilus	6.2000E-02	11.5	1.1658E-02	Ehnes et al. 2011
3944	Invertebrates	Poecilus	6.3000E-02	18.5	1.7196E-02	Ehnes et al. 2011
3945	Invertebrates	Poecilus	6.3600E-02	15	1.2241E-02	Ehnes et al. 2011
3946	Invertebrates	Poecilus	6.4500E-02	22	3.2351E-02	Ehnes et al. 2011
3947	Invertebrates	Poecilus	6.4700E-02	15	1.9236E-02	Ehnes et al. 2011
3948	Invertebrates	Poecilus	6.5300E-02	18.5	2.3608E-02	Ehnes et al. 2011
3949	Invertebrates	Poecilus	6.5500E-02	11.5	1.5301E-02	Ehnes et al. 2011
3950	Invertebrates	Poecilus	6.6900E-02	15	1.7050E-02	Ehnes et al. 2011
3951	Invertebrates	Poecilus	6.7100E-02	22	3.0311E-02	Ehnes et al. 2011
3952	Invertebrates	Poecilus	6.8300E-02	18.5	2.5356E-02	Ehnes et al. 2011
3953	Invertebrates	Poecilus	6.9000E-02	22	3.2351E-02	Ehnes et al. 2011
3954	Invertebrates	Poecilus	7.6000E-02	11.5	7.2863E-03	Ehnes et al. 2011
3955	Invertebrates	Poecilus	7.8800E-02	15	1.3698E-02	Ehnes et al. 2011
3956	Invertebrates	Poecilus versicolor	3.9600E-02	10	2.3316E-03	Ehnes et al. 2011
3957	Invertebrates	Poecilus versicolor	4.1500E-02	10	3.9346E-03	Ehnes et al. 2011
3958	Invertebrates	Poecilus versicolor	4.2190E-02	25	1.6467E-02	Ehnes et al. 2011
3959	Invertebrates	Poecilus versicolor	4.2300E-02	30	1.7924E-02	Ehnes et al. 2011
3960	Invertebrates	Poecilus versicolor	4.4610E-02	25	1.6613E-02	Ehnes et al. 2011
3961	Invertebrates	Poecilus versicolor	4.6290E-02	25	1.5447E-02	Ehnes et al. 2011
3962	Invertebrates	Poecilus versicolor	4.7320E-02	20	8.7436E-03	Ehnes et al. 2011

3963	Invertebrates	Poecilus versicolor	4.7830E-02	20	1.2678E-02	Ehnes et al. 2011
3964	Invertebrates	Poecilus versicolor	4.8550E-02	30	1.9090E-02	Ehnes et al. 2011
3965	Invertebrates	Poecilus versicolor	4.9050E-02	30	2.5793E-02	Ehnes et al. 2011
3966	Invertebrates	Poecilus versicolor	4.9200E-02	20	1.2387E-02	Ehnes et al. 2011
3967	Invertebrates	Poecilus versicolor	4.9600E-02	25	1.8799E-02	Ehnes et al. 2011
3968	Invertebrates	Poecilus versicolor	4.9850E-02	10	1.8945E-03	Ehnes et al. 2011
3969	Invertebrates	Poecilus versicolor	4.9930E-02	10	2.0402E-03	Ehnes et al. 2011
3970	Invertebrates	Poecilus versicolor	5.1410E-02	30	3.0457E-02	Ehnes et al. 2011
3971	Invertebrates	Poecilus versicolor	5.1590E-02	30	2.6959E-02	Ehnes et al. 2011
3972	Invertebrates	Poecilus versicolor	5.3640E-02	20	1.5593E-02	Ehnes et al. 2011
3973	Invertebrates	Poecilus versicolor	5.4970E-02	20	1.0347E-02	Ehnes et al. 2011
3974	Invertebrates	Poecilus versicolor	5.6020E-02	25	3.5411E-02	Ehnes et al. 2011
3975	Invertebrates	Poecilus versicolor	5.7730E-02	10	2.6231E-03	Ehnes et al. 2011
3976	Invertebrates	Poecilus versicolor	5.8410E-02	20	1.3990E-02	Ehnes et al. 2011
3977	Invertebrates	Poecilus versicolor	5.9660E-02	10	2.1859E-03	Ehnes et al. 2011
3978	Invertebrates	Poecilus versicolor	6.0170E-02	20	1.0055E-02	Ehnes et al. 2011
3979	Invertebrates	Poecilus versicolor	6.1050E-02	20	8.8893E-03	Ehnes et al. 2011
3980	Invertebrates	Poecilus versicolor	6.1820E-02	20	1.1658E-02	Ehnes et al. 2011
3981	Invertebrates	Poecilus versicolor	6.1860E-02	25	1.9964E-02	Ehnes et al. 2011
3982	Invertebrates	Poecilus versicolor	6.3080E-02	20	1.4427E-02	Ehnes et al. 2011
3983	Invertebrates	Poecilus versicolor	6.3450E-02	20	9.4722E-03	Ehnes et al. 2011
3984	Invertebrates	Poecilus versicolor	6.3540E-02	10	2.9145E-03	Ehnes et al. 2011
3985	Invertebrates	Poecilus versicolor	6.6710E-02	10	7.5777E-03	Ehnes et al. 2011
3986	Invertebrates	Poecilus versicolor	6.9660E-02	10	3.6432E-03	Ehnes et al. 2011
3987	Invertebrates	Poecilus versicolor	7.1500E-02	30	2.6959E-02	Ehnes et al. 2011
3988	Invertebrates	Poecilus versicolor	7.2100E-02	30	4.5321E-02	Ehnes et al. 2011

3989	Invertebrates	Poecilus versicolor	7.2350E-02	25	2.0547E-02	Ehnes et al. 2011
3990	Invertebrates	Poecilus versicolor	7.5490E-02	25	1.6467E-02	Ehnes et al. 2011
3991	Invertebrates	Poecilus versicolor	7.6810E-02	20	1.6904E-02	Ehnes et al. 2011
3992	Invertebrates	Poecilus versicolor	7.6870E-02	5	4.2261E-03	Ehnes et al. 2011
3993	Invertebrates	Poecilus versicolor	7.6900E-02	5	6.4119E-03	Ehnes et al. 2011
3994	Invertebrates	Poecilus versicolor	7.7740E-02	10	3.7889E-03	Ehnes et al. 2011
3995	Invertebrates	Poecilus versicolor	7.8760E-02	25	4.8964E-02	Ehnes et al. 2011
3996	Invertebrates	Poecilus versicolor	7.9760E-02	5	5.9748E-03	Ehnes et al. 2011
3997	Invertebrates	Poecilus versicolor	8.0470E-02	5	4.3718E-03	Ehnes et al. 2011
3998	Invertebrates	Poecilus versicolor	8.0950E-02	5	9.1807E-03	Ehnes et al. 2011
3999	Invertebrates	Poecilus versicolor	8.3940E-02	5	5.2461E-03	Ehnes et al. 2011
4000	Invertebrates	Poecilus versicolor	8.5010E-02	5	6.9948E-03	Ehnes et al. 2011
4001	Invertebrates	Poecilus versicolor	8.7670E-02	5	3.7889E-03	Ehnes et al. 2011
4002	Invertebrates	Poecilus versicolor	9.4630E-02	5	7.7235E-03	Ehnes et al. 2011
4003	Invertebrates	Pogonognathellus flavescens	4.1000E-04	6	6.5000E-05	Ehnes et al. 2011
4004	Invertebrates	Pogonognathellus flavescens	4.1000E-04	10	1.1600E-04	Ehnes et al. 2011
4005	Invertebrates	Pogonognathellus flavescens	4.1000E-04	15	2.6000E-04	Ehnes et al. 2011
4006	Invertebrates	Pogonognathellus longicornis	3.2500E-03	8	2.9850E-04	Ehnes et al. 2011
4007	Invertebrates	Pogonognathellus longicornis	3.2500E-03	18	8.7500E-04	Ehnes et al. 2011
4008	Invertebrates	Pogonomyrmex californicus	5.9200E-03	25	1.6740E-03	Ehnes et al. 2011
4009	Invertebrates	Pogonomyrmex maricopa	1.1070E-02	25	7.3080E-03	Ehnes et al. 2011
4010	Invertebrates	Pogonomyrmex occidentalis	7.9600E-03	25	2.3040E-03	Ehnes et al. 2011
4011	Invertebrates	Pogonomyrmex rugosus	1.4300E-02	25	4.9680E-03	Ehnes et al. 2011
4012	Invertebrates	Pogonomyrmex sp.	3.7400E-03	25	2.2680E-03	Ehnes et al. 2011
4013	Invertebrates	Polyartemia forcipata	7.6900E-03	20	2.7684E-03	Makarieva et al. 2008
4014	Invertebrates	Polydesmida	4.4425E-03	19.8	1.7487E-03	Ehnes et al. 2011

4015	Invertebrates	Polydesmida	4.5100E-03	14.5	5.8290E-04	Ehnes et al. 2011
4016	Invertebrates	Polydesmida	4.7357E-03	19.8	1.3113E-03	Ehnes et al. 2011
4017	Invertebrates	Polydesmida	4.9440E-03	19.8	6.7000E-04	Ehnes et al. 2011
4018	Invertebrates	Polydesmida	5.2980E-03	10.8	1.3119E-03	Ehnes et al. 2011
4019	Invertebrates	Polydesmida	5.3280E-03	14.5	2.4777E-03	Ehnes et al. 2011
4020	Invertebrates	Polydesmida	5.4420E-03	10.8	1.7782E-03	Ehnes et al. 2011
4021	Invertebrates	Polydesmida	5.6300E-03	14.5	1.0787E-03	Ehnes et al. 2011
4022	Invertebrates	Polydesmida	5.8740E-03	10.8	5.8290E-04	Ehnes et al. 2011
4023	Invertebrates	Polydesmida	6.0325E-03	19.8	1.8576E-03	Ehnes et al. 2011
4024	Invertebrates	Polydesmida	6.0340E-03	14.5	1.2824E-03	Ehnes et al. 2011
4025	Invertebrates	Polydesmida	6.0660E-03	10.8	6.9950E-04	Ehnes et al. 2011
4026	Invertebrates	Polydesmida	6.0860E-03	10.8	8.1605E-04	Ehnes et al. 2011
4027	Invertebrates	Polydesmida	6.2640E-03	10.8	8.7505E-04	Ehnes et al. 2011
4028	Invertebrates	Polydesmida	6.2780E-03	10.8	7.8655E-04	Ehnes et al. 2011
4029	Invertebrates	Polydesmida	6.2940E-03	14.5	1.0492E-03	Ehnes et al. 2011
4030	Invertebrates	Polydesmida	6.2975E-03	14.5	9.1120E-04	Ehnes et al. 2011
4031	Invertebrates	Polydesmida	6.4060E-03	19.8	4.0875E-04	Ehnes et al. 2011
4032	Invertebrates	Polydesmida	6.4660E-03	19.8	1.8653E-03	Ehnes et al. 2011
4033	Invertebrates	Polydesmida	6.6140E-03	19.8	1.6322E-03	Ehnes et al. 2011
4034	Invertebrates	Polydesmida	6.6525E-03	19.8	2.2948E-03	Ehnes et al. 2011
4035	Invertebrates	Polydesmida	1.4930E-02	19.8	6.2645E-03	Ehnes et al. 2011
4036	Invertebrates	Polydesmida	1.5290E-02	14.5	3.6449E-03	Ehnes et al. 2011
4037	Invertebrates	Polydesmida	1.8590E-02	19.8	5.2461E-03	Ehnes et al. 2011
4038	Invertebrates	Pontella danae	3.5000E-03	26.4	3.4272E-03	Makarieva et al. 2008
4039	Invertebrates	Pontella sp.	3.4500E-04	15	3.5981E-04	Makarieva et al. 2008
4040	Invertebrates	Pontogeneia antarctica	5.0000E-03	-1	1.6200E-03	Makarieva et al. 2008

4041	Invertebrates	Popilius disjunctus	1.6305E+00	25	2.9009E-01	Ehnes et al. 2011
4042	Invertebrates	Popilius sp.	5.6300E-01	25	1.4389E-01	Ehnes et al. 2011
4043	Invertebrates	Porcellio laevis	1.2000E-02	20	2.3998E-03	Makarieva et al. 2008
4044	Invertebrates	Porcellio laevis	6.0000E-02	10	4.7050E-03	Ehnes et al. 2011
4045	Invertebrates	Porcellio laevis	6.0000E-02	20	8.8500E-03	Ehnes et al. 2011
4046	Invertebrates	Porcellio laevis	6.0000E-02	30	1.4500E-02	Ehnes et al. 2011
4047	Invertebrates	Porcellio laevis	6.0000E-02	18	2.1600E-02	Ehnes et al. 2011
4048	Invertebrates	Porcellio laevis	6.0000E-02	25	4.5850E-02	Ehnes et al. 2011
4049	Invertebrates	Porcellio laevis	7.0000E-02	5	3.3350E-03	Ehnes et al. 2011
4050	Invertebrates	Porcellio laevis	7.0000E-02	12	1.4850E-02	Ehnes et al. 2011
4051	Invertebrates	Porcellio laevis	7.0000E-02	18	2.7050E-02	Ehnes et al. 2011
4052	Invertebrates	Porcellio laevis	7.0000E-02	25	6.0000E-02	Ehnes et al. 2011
4053	Invertebrates	Porcellio laevis	8.0000E-02	5	5.6500E-03	Ehnes et al. 2011
4054	Invertebrates	Porcellio laevis	8.0000E-02	12	1.0400E-02	Ehnes et al. 2011
4055	Invertebrates	Porcellio laevis	1.8000E-01	20	1.6200E-02	Makarieva et al. 2008
4056	Invertebrates	Porcellio scaber	1.1100E-03	10	1.1658E-03	Ehnes et al. 2011
4057	Invertebrates	Porcellio scaber	1.3120E-03	10	1.6030E-03	Ehnes et al. 2011
4058	Invertebrates	Porcellio scaber	2.0000E-03	20	5.0500E-04	Ehnes et al. 2011
4059	Invertebrates	Porcellio scaber	4.7200E-03	29.6	7.2863E-03	Ehnes et al. 2011
4060	Invertebrates	Porcellio scaber	5.0750E-03	10.3	3.6430E-04	Ehnes et al. 2011
4061	Invertebrates	Porcellio scaber	7.0000E-03	20	2.7997E-03	Makarieva et al. 2008
4062	Invertebrates	Porcellio scaber	7.9900E-03	10.3	1.0201E-03	Ehnes et al. 2011
4063	Invertebrates	Porcellio scaber	1.0360E-02	18	4.3718E-03	Ehnes et al. 2011
4064	Invertebrates	Porcellio scaber	1.5360E-02	14.6	2.1859E-03	Ehnes et al. 2011
4065	Invertebrates	Porcellio scaber	1.6370E-02	10.3	1.3116E-03	Ehnes et al. 2011
4066	Invertebrates	Porcellio scaber	1.6530E-02	14.6	1.8945E-03	Ehnes et al. 2011

4067	Invertebrates	Porcellio scaber	1.7950E-02	14.6	2.3316E-03	Ehnes et al. 2011
4068	Invertebrates	Porcellio scaber	1.8050E-02	10.3	4.3720E-04	Ehnes et al. 2011
4069	Invertebrates	Porcellio scaber	1.8330E-02	29.2	1.2387E-02	Ehnes et al. 2011
4070	Invertebrates	Porcellio scaber	1.8750E-02	10.2	1.1658E-03	Ehnes et al. 2011
4071	Invertebrates	Porcellio scaber	2.0700E-02	14.6	3.4974E-03	Ehnes et al. 2011
4072	Invertebrates	Porcellio scaber	2.1510E-02	14.6	6.7034E-03	Ehnes et al. 2011
4073	Invertebrates	Porcellio scaber	2.2340E-02	14.6	2.7688E-03	Ehnes et al. 2011
4074	Invertebrates	Porcellio scaber	2.3580E-02	14.6	2.9145E-03	Ehnes et al. 2011
4075	Invertebrates	Porcellio scaber	2.3600E-02	14.2	4.0803E-03	Ehnes et al. 2011
4076	Invertebrates	Porcellio scaber	2.4790E-02	14.2	3.7889E-03	Ehnes et al. 2011
4077	Invertebrates	Porcellio scaber	2.5530E-02	14.6	2.7688E-03	Ehnes et al. 2011
4078	Invertebrates	Porcellio scaber	2.5810E-02	14.6	3.4974E-03	Ehnes et al. 2011
4079	Invertebrates	Porcellio scaber	2.6240E-02	29.6	1.3261E-02	Ehnes et al. 2011
4080	Invertebrates	Porcellio scaber	2.6500E-02	14.2	1.8945E-03	Ehnes et al. 2011
4081	Invertebrates	Porcellio scaber	2.7800E-02	19.5	9.6179E-03	Ehnes et al. 2011
4082	Invertebrates	Porcellio scaber	2.8530E-02	5	5.8290E-04	Ehnes et al. 2011
4083	Invertebrates	Porcellio scaber	3.0060E-02	14.6	3.6432E-03	Ehnes et al. 2011
4084	Invertebrates	Porcellio scaber	3.0480E-02	19.5	1.4573E-02	Ehnes et al. 2011
4085	Invertebrates	Porcellio scaber	3.1740E-02	14.2	4.2261E-03	Ehnes et al. 2011
4086	Invertebrates	Porcellio scaber	3.1940E-02	14.6	5.3919E-03	Ehnes et al. 2011
4087	Invertebrates	Porcellio scaber	3.2180E-02	29.6	1.7779E-02	Ehnes et al. 2011
4088	Invertebrates	Porcellio scaber	3.2440E-02	5	7.2865E-04	Ehnes et al. 2011
4089	Invertebrates	Porcellio scaber	3.3390E-02	14.6	4.3718E-03	Ehnes et al. 2011
4090	Invertebrates	Porcellio scaber	3.3520E-02	5	7.2865E-04	Ehnes et al. 2011
4091	Invertebrates	Porcellio scaber	3.3580E-02	5	5.8290E-04	Ehnes et al. 2011
4092	Invertebrates	Porcellio scaber	3.6170E-02	14.2	5.5376E-03	Ehnes et al. 2011

4093	Invertebrates	Porcellio scaber	3.7280E-02	14.9	1.0201E-03	Ehnes et al. 2011
4094	Invertebrates	Porcellio scaber	3.7310E-02	14.6	3.9346E-03	Ehnes et al. 2011
4095	Invertebrates	Porcellio scaber	3.7770E-02	23.4	1.3698E-02	Ehnes et al. 2011
4096	Invertebrates	Porcellio scaber	3.7880E-02	14.9	6.9948E-03	Ehnes et al. 2011
4097	Invertebrates	Porcellio scaber	3.8140E-02	14.9	9.3265E-03	Ehnes et al. 2011
4098	Invertebrates	Porcellio scaber	3.8670E-02	5	4.3720E-04	Ehnes et al. 2011
4099	Invertebrates	Porcellio scaber	3.9470E-02	5	4.3720E-04	Ehnes et al. 2011
4100	Invertebrates	Porcellio scaber	3.9990E-02	10	1.6030E-03	Ehnes et al. 2011
4101	Invertebrates	Porcellio scaber	4.0750E-02	14.6	3.7889E-03	Ehnes et al. 2011
4102	Invertebrates	Porcellio scaber	4.0810E-02	5	8.7435E-04	Ehnes et al. 2011
4103	Invertebrates	Porcellio scaber	4.2610E-02	5	2.1859E-03	Ehnes et al. 2011
4104	Invertebrates	Porcellio scaber	4.3150E-02	5	8.7435E-04	Ehnes et al. 2011
4105	Invertebrates	Porcellio scaber	4.3440E-02	5	1.7487E-03	Ehnes et al. 2011
4106	Invertebrates	Porcellio scaber	4.5010E-02	5	1.7633E-02	Ehnes et al. 2011
4107	Invertebrates	Porcellio scaber	4.5310E-02	14.9	6.2662E-03	Ehnes et al. 2011
4108	Invertebrates	Porcellio scaber	4.8360E-02	5	1.1658E-03	Ehnes et al. 2011
4109	Invertebrates	Porcellio scaber	5.3530E-02	5	6.7034E-03	Ehnes et al. 2011
4110	Invertebrates	Porcellio scaber	5.3640E-02	5	5.8290E-04	Ehnes et al. 2011
4111	Invertebrates	Porcellio scaber	6.0000E-02	20	7.2036E-03	Makarieva et al. 2008
4112	Invertebrates	Porcellio scaber	6.0000E-02	20	6.8000E-03	Ehnes et al. 2011
4113	Invertebrates	Porcellio scaber	6.0520E-02	5	1.0201E-03	Ehnes et al. 2011
4114	Invertebrates	Porcellio scaber	6.6740E-02	5	1.8945E-03	Ehnes et al. 2011
4115	Invertebrates	Porcellio scaber	6.9040E-02	23.4	1.1512E-02	Ehnes et al. 2011
4116	Invertebrates	Porcellio scaber	7.0090E-02	5	1.7487E-03	Ehnes et al. 2011
4117	Invertebrates	Porcellio scaber	1.0000E-01	20	1.9998E-02	Makarieva et al. 2008
4118	Invertebrates	Porcellio scaber	2.5000E-01	20	5.9985E-02	Makarieva et al. 2008

4119	Invertebrates	Porcellionides pruinosus	1.5000E-02	15	2.9700E-03	Ehnes et al. 2011
4120	Invertebrates	Porcellionides pruinosus	1.5000E-02	20	4.1000E-03	Ehnes et al. 2011
4121	Invertebrates	Porcellionides pruinosus	1.5000E-02	25	4.8500E-03	Ehnes et al. 2011
4122	Invertebrates	Porcellionides pruinosus	1.5000E-02	30	4.9200E-03	Ehnes et al. 2011
4123	Invertebrates	Porcellionides pruinosus	1.5000E-02	35	9.3000E-03	Ehnes et al. 2011
4124	Invertebrates	Porcellionides pruinosus	1.8400E-02	20	6.2000E-03	Ehnes et al. 2011
4125	Invertebrates	Porcellionides pruinosus	2.5000E-02	20	7.8500E-03	Ehnes et al. 2011
4126	Invertebrates	Porcellionides pruinosus	2.5000E-02	34	2.7800E-02	Ehnes et al. 2011
4127	Invertebrates	Proaciculitermes sp. A	1.1000E-03	25	1.4400E-04	Ehnes et al. 2011
4128	Invertebrates	Proaciculitermes sp. E	2.7000E-03	25	2.5200E-04	Ehnes et al. 2011
4129	Invertebrates	Procapritermes nr. Sandakanensis	5.9000E-03	25	4.3200E-04	Ehnes et al. 2011
4130	Invertebrates	Prohamitermes mirabilis	3.5000E-03	25	3.4200E-04	Ehnes et al. 2011
4131	Invertebrates	Promachus sp. 2	1.8000E-01	25	2.6053E-01	Ehnes et al. 2011
4132	Invertebrates	Protambulyx strigilis	1.0953E+00	25	4.0520E-01	Ehnes et al. 2011
4133	Invertebrates	Protaphorura armata	5.0000E-05	6	7.8000E-06	Ehnes et al. 2011
4134	Invertebrates	Protaphorura armata	5.0000E-05	10	1.2800E-05	Ehnes et al. 2011
4135	Invertebrates	Protaphorura armata	5.0000E-05	15	2.2550E-05	Ehnes et al. 2011
4136	Invertebrates	Protaphorura armata	1.1000E-04	3	2.0550E-05	Ehnes et al. 2011
4137	Invertebrates	Protaphorura armata	1.1000E-04	8	2.7400E-05	Ehnes et al. 2011
4138	Invertebrates	Protaphorura armata	1.1000E-04	18	7.1500E-05	Ehnes et al. 2011
4139	Invertebrates	Protaphorura armata	1.3000E-04	18	8.4000E-05	Ehnes et al. 2011
4140	Invertebrates	Protaphorura meridiata	1.0000E-05	5	7.0500E-06	Ehnes et al. 2011
4141	Invertebrates	Protaphorura meridiata	1.0000E-05	10	7.3000E-06	Ehnes et al. 2011
4142	Invertebrates	Protaphorura meridiata	1.0000E-05	15	7.8000E-06	Ehnes et al. 2011
4143	Invertebrates	Protaphorura meridiata	1.0000E-05	20	1.0650E-05	Ehnes et al. 2011
4144	Invertebrates	Protaphorura meridiata	1.0000E-05	25	1.2500E-05	Ehnes et al. 2011

4145	Invertebrates	Protophormia terraenovae	2.5000E-02	25	4.0536E-02	Ehnes et al. 2011
4146	Invertebrates	Psammodes striatus	3.0100E+00	25	7.6887E-01	Ehnes et al. 2011
4147	Invertebrates	Pseudocalanus elongatus	4.0000E-05	11.15	4.7441E-05	Makarieva et al. 2008
4148	Invertebrates	Pseudocalanus elongatus	5.9000E-05	20	6.4782E-05	Makarieva et al. 2008
4149	Invertebrates	Pseudocalanus elongatus	6.0000E-05	8.6	3.9960E-05	Makarieva et al. 2008
4150	Invertebrates	Pseudochirella polyspina	1.1700E-02	5	4.5068E-04	Makarieva et al. 2008
4151	Invertebrates	Pseudococcus citri	1.3000E-03	25	2.0340E-03	Ehnes et al. 2011
4152	Invertebrates	Pseudodiaptomus marinus	7.0000E-05	14.3	5.5440E-05	Makarieva et al. 2008
4153	Invertebrates	Pseudophonus rufipes	4.5560E-02	20	1.6321E-02	Ehnes et al. 2011
4154	Invertebrates	Pseudophonus rufipes	4.9540E-02	20	1.5738E-02	Ehnes et al. 2011
4155	Invertebrates	Pseudophonus rufipes	5.1920E-02	20	9.9094E-03	Ehnes et al. 2011
4156	Invertebrates	Pseudophonus rufipes	5.3550E-02	20	1.7196E-02	Ehnes et al. 2011
4157	Invertebrates	Pseudophonus rufipes	6.2380E-02	20	1.4718E-02	Ehnes et al. 2011
4158	Invertebrates	Pseudophonus rufipes	6.6320E-02	20	1.5447E-02	Ehnes et al. 2011
4159	Invertebrates	Pseudophonus rufipes	8.7460E-02	20	1.2095E-02	Ehnes et al. 2011
4160	Invertebrates	Pseudophonus rufipes	8.8900E-02	15	1.8653E-02	Ehnes et al. 2011
4161	Invertebrates	Pseudophonus rufipes	8.9310E-02	20	1.2387E-02	Ehnes et al. 2011
4162	Invertebrates	Pseudophonus rufipes	8.9670E-02	5	4.5175E-03	Ehnes et al. 2011
4163	Invertebrates	Pseudophonus rufipes	9.0830E-02	20	1.4135E-02	Ehnes et al. 2011
4164	Invertebrates	Pseudophonus rufipes	9.3120E-02	20	1.1950E-02	Ehnes et al. 2011
4165	Invertebrates	Pseudophonus rufipes	9.4600E-02	22	4.4155E-02	Ehnes et al. 2011
4166	Invertebrates	Pseudophonus rufipes	9.4670E-02	30	6.0622E-02	Ehnes et al. 2011
4167	Invertebrates	Pseudophonus rufipes	9.4800E-02	15	2.3608E-02	Ehnes et al. 2011
4168	Invertebrates	Pseudophonus rufipes	9.4870E-02	10	4.0803E-03	Ehnes et al. 2011
4169	Invertebrates	Pseudophonus rufipes	9.5810E-02	5	8.1607E-03	Ehnes et al. 2011
4170	Invertebrates	Pseudophonus rufipes	9.6200E-02	10	5.3919E-03	Ehnes et al. 2011

4171	Invertebrates	Pseudophonus rufipes	9.6600E-02	8	9.0350E-03	Ehnes et al. 2011
4172	Invertebrates	Pseudophonus rufipes	9.7290E-02	30	5.0858E-02	Ehnes et al. 2011
4173	Invertebrates	Pseudophonus rufipes	9.7600E-02	18.5	1.2532E-02	Ehnes et al. 2011
4174	Invertebrates	Pseudophonus rufipes	9.7630E-02	30	2.9145E-02	Ehnes et al. 2011
4175	Invertebrates	Pseudophonus rufipes	9.8400E-02	5	6.8491E-03	Ehnes et al. 2011
4176	Invertebrates	Pseudophonus rufipes	9.9410E-02	25	2.4336E-02	Ehnes et al. 2011
4177	Invertebrates	Pseudophonus rufipes	9.9800E-02	22	2.6231E-02	Ehnes et al. 2011
4178	Invertebrates	Pseudophonus rufipes	1.0010E-01	15	7.4320E-03	Ehnes et al. 2011
4179	Invertebrates	Pseudophonus rufipes	1.0029E-01	30	5.3627E-02	Ehnes et al. 2011
4180	Invertebrates	Pseudophonus rufipes	1.0110E-01	18.5	3.1040E-02	Ehnes et al. 2011
4181	Invertebrates	Pseudophonus rufipes	1.0110E-01	22	3.7160E-02	Ehnes et al. 2011
4182	Invertebrates	Pseudophonus rufipes	1.0180E-01	15	2.4482E-02	Ehnes et al. 2011
4183	Invertebrates	Pseudophonus rufipes	1.0190E-01	8	8.5978E-03	Ehnes et al. 2011
4184	Invertebrates	Pseudophonus rufipes	1.0260E-01	22	3.7889E-02	Ehnes et al. 2011
4185	Invertebrates	Pseudophonus rufipes	1.0354E-01	25	2.4919E-02	Ehnes et al. 2011
4186	Invertebrates	Pseudophonus rufipes	1.0417E-01	5	9.0350E-03	Ehnes et al. 2011
4187	Invertebrates	Pseudophonus rufipes	1.0477E-01	25	1.5884E-02	Ehnes et al. 2011
4188	Invertebrates	Pseudophonus rufipes	1.0502E-01	15	2.5065E-02	Ehnes et al. 2011
4189	Invertebrates	Pseudophonus rufipes	1.0550E-01	22	5.1295E-02	Ehnes et al. 2011
4190	Invertebrates	Pseudophonus rufipes	1.0560E-01	11.5	1.0638E-02	Ehnes et al. 2011
4191	Invertebrates	Pseudophonus rufipes	1.0634E-01	25	4.4155E-02	Ehnes et al. 2011
4192	Invertebrates	Pseudophonus rufipes	1.0680E-01	18.5	1.3990E-02	Ehnes et al. 2011
4193	Invertebrates	Pseudophonus rufipes	1.0870E-01	18.5	2.8562E-02	Ehnes et al. 2011
4194	Invertebrates	Pseudophonus rufipes	1.0890E-01	8	6.9948E-03	Ehnes et al. 2011
4195	Invertebrates	Pseudophonus rufipes	1.0900E-01	15	9.6179E-03	Ehnes et al. 2011
4196	Invertebrates	Pseudophonus rufipes	1.1057E-01	25	2.3170E-02	Ehnes et al. 2011

4197	Invertebrates	Pseudophonus rufipes	1.1090E-01	11.5	1.0929E-02	Ehnes et al. 2011
4198	Invertebrates	Pseudophonus rufipes	1.1130E-01	8	9.6179E-03	Ehnes et al. 2011
4199	Invertebrates	Pseudophonus rufipes	1.1235E-01	15	1.2824E-02	Ehnes et al. 2011
4200	Invertebrates	Pseudophonus rufipes	1.1270E-01	15	2.4919E-02	Ehnes et al. 2011
4201	Invertebrates	Pseudophonus rufipes	1.1280E-01	18.5	7.4466E-02	Ehnes et al. 2011
4202	Invertebrates	Pseudophonus rufipes	1.1338E-01	5	6.4119E-03	Ehnes et al. 2011
4203	Invertebrates	Pseudophonus rufipes	1.1346E-01	25	4.1095E-02	Ehnes et al. 2011
4204	Invertebrates	Pseudophonus rufipes	1.1385E-01	30	4.3426E-02	Ehnes et al. 2011
4205	Invertebrates	Pseudophonus rufipes	1.1470E-01	11.5	1.0347E-02	Ehnes et al. 2011
4206	Invertebrates	Pseudophonus rufipes	1.1473E-01	20	1.4573E-02	Ehnes et al. 2011
4207	Invertebrates	Pseudophonus rufipes	1.1487E-01	25	2.6085E-02	Ehnes et al. 2011
4208	Invertebrates	Pseudophonus rufipes	1.1488E-01	5	1.8945E-03	Ehnes et al. 2011
4209	Invertebrates	Pseudophonus rufipes	1.1491E-01	20	6.2662E-03	Ehnes et al. 2011
4210	Invertebrates	Pseudophonus rufipes	1.1536E-01	15	9.3265E-03	Ehnes et al. 2011
4211	Invertebrates	Pseudophonus rufipes	1.1610E-01	11.5	1.5738E-02	Ehnes et al. 2011
4212	Invertebrates	Pseudophonus rufipes	1.1630E-01	11.5	1.6321E-02	Ehnes et al. 2011
4213	Invertebrates	Pseudophonus rufipes	1.1631E-01	5	7.0000E-03	Ehnes et al. 2011
4214	Invertebrates	Pseudophonus rufipes	1.1690E-01	8	5.8290E-03	Ehnes et al. 2011
4215	Invertebrates	Pseudophonus rufipes	1.1750E-01	8	6.4119E-03	Ehnes et al. 2011
4216	Invertebrates	Pseudophonus rufipes	1.1759E-01	5	5.3919E-03	Ehnes et al. 2011
4217	Invertebrates	Pseudophonus rufipes	1.1806E-01	25	1.8944E-02	Ehnes et al. 2011
4218	Invertebrates	Pseudophonus rufipes	1.1808E-01	5	1.2387E-02	Ehnes et al. 2011
4219	Invertebrates	Pseudophonus rufipes	1.1886E-01	10	5.2461E-03	Ehnes et al. 2011
4220	Invertebrates	Pseudophonus rufipes	1.1935E-01	30	4.1532E-02	Ehnes et al. 2011
4221	Invertebrates	Pseudophonus rufipes	1.2106E-01	5	3.5000E-03	Ehnes et al. 2011
4222	Invertebrates	Pseudophonus rufipes	1.2141E-01	30	7.2134E-02	Ehnes et al. 2011

4223	Invertebrates	Pseudophonus rufipes	1.2699E-01	15	7.8692E-03	Ehnes et al. 2011
4224	Invertebrates	Pseudophonus rufipes	1.2700E-01	5	9.4722E-03	Ehnes et al. 2011
4225	Invertebrates	Pseudophonus rufipes	1.2820E-01	15	5.4210E-02	Ehnes et al. 2011
4226	Invertebrates	Pseudophonus rufipes	1.2958E-01	5	5.2461E-03	Ehnes et al. 2011
4227	Invertebrates	Pseudophonus rufipes	1.3150E-01	5	6.5577E-03	Ehnes et al. 2011
4228	Invertebrates	Pseudophonus rufipes	1.3276E-01	5	7.5777E-03	Ehnes et al. 2011
4229	Invertebrates	Pseudophonus rufipes	1.3379E-01	30	4.1969E-02	Ehnes et al. 2011
4230	Invertebrates	Pseudophonus rufipes	1.3437E-01	25	8.1315E-02	Ehnes et al. 2011
4231	Invertebrates	Pseudophonus rufipes	1.3490E-01	10	8.8893E-03	Ehnes et al. 2011
4232	Invertebrates	Pseudophonus rufipes	1.3630E-01	11.5	9.1807E-03	Ehnes et al. 2011
4233	Invertebrates	Pseudophonus rufipes	1.3650E-01	5	4.5000E-03	Ehnes et al. 2011
4234	Invertebrates	Pseudophonus rufipes	1.3709E-01	15	4.7361E-02	Ehnes et al. 2011
4235	Invertebrates	Pseudophonus rufipes	1.3719E-01	10	6.5577E-03	Ehnes et al. 2011
4236	Invertebrates	Pseudophonus rufipes	1.3818E-01	20	1.0055E-02	Ehnes et al. 2011
4237	Invertebrates	Pseudophonus rufipes	1.4070E-01	10	1.1950E-02	Ehnes et al. 2011
4238	Invertebrates	Pseudophonus rufipes	1.4414E-01	30	1.2751E-01	Ehnes et al. 2011
4239	Invertebrates	Pseudophonus rufipes	1.4434E-01	22	6.3099E-02	Ehnes et al. 2011
4240	Invertebrates	Pseudophonus rufipes	1.4650E-01	11.5	1.3844E-02	Ehnes et al. 2011
4241	Invertebrates	Pseudophonus rufipes	1.4821E-01	10	9.9094E-03	Ehnes et al. 2011
4242	Invertebrates	Pseudophonus rufipes	1.4940E-01	8	1.2532E-02	Ehnes et al. 2011
4243	Invertebrates	Pseudophonus rufipes	1.4977E-01	20	1.1804E-02	Ehnes et al. 2011
4244	Invertebrates	Pseudophonus rufipes	1.5110E-01	18.5	2.9728E-02	Ehnes et al. 2011
4245	Invertebrates	Pseudophonus rufipes	1.5803E-01	20	4.9692E-02	Ehnes et al. 2011
4246	Invertebrates	Pseudophonus rufipes	1.5818E-01	10	1.3407E-02	Ehnes et al. 2011
4247	Invertebrates	Pseudophonus rufipes	1.6360E-01	11.5	2.3025E-02	Ehnes et al. 2011
4248	Invertebrates	Pseudophonus rufipes	1.6704E-01	30	5.3918E-02	Ehnes et al. 2011

4249	Invertebrates	Pseudophonus rufipes	1.7090E-01	18.5	6.8782E-02	Ehnes et al. 2011
4250	Invertebrates	Pseudophonus rufipes	1.7515E-01	5	9.4722E-03	Ehnes et al. 2011
4251	Invertebrates	Pseudosagitta gazellae	3.8700E-01	-0.9	1.2539E-03	Makarieva et al. 2008
4252	Invertebrates	Pseudosagitta lyra	2.3700E-01	5	1.3651E-03	Makarieva et al. 2008
4253	Invertebrates	Pseudosagitta maxima	2.3500E-01	5	1.4805E-03	Makarieva et al. 2008
4254	Invertebrates	Pternonemobius fasciatus	2.6170E-02	25	8.4510E-02	Ehnes et al. 2011
4255	Invertebrates	Pterohelaeus sp.	2.4500E-01	25	8.6688E-02	Ehnes et al. 2011
4256	Invertebrates	Pterostichus melanarius	9.2200E-02	22	5.3918E-02	Ehnes et al. 2011
4257	Invertebrates	Pterostichus melanarius	9.4200E-02	22	5.1004E-02	Ehnes et al. 2011
4258	Invertebrates	Pterostichus melanarius	1.0238E-01	10	7.5777E-03	Ehnes et al. 2011
4259	Invertebrates	Pterostichus melanarius	1.0280E-01	22	5.2315E-02	Ehnes et al. 2011
4260	Invertebrates	Pterostichus melanarius	1.0643E-01	15	1.8070E-02	Ehnes et al. 2011
4261	Invertebrates	Pterostichus melanarius	1.1079E-01	10	9.9094E-03	Ehnes et al. 2011
4262	Invertebrates	Pterostichus melanarius	1.1494E-01	5	6.4119E-03	Ehnes et al. 2011
4263	Invertebrates	Pterostichus melanarius	1.1679E-01	5	7.7235E-03	Ehnes et al. 2011
4264	Invertebrates	Pterostichus melanarius	1.1800E-01	22	5.5521E-02	Ehnes et al. 2011
4265	Invertebrates	Pterostichus melanarius	1.1950E-01	25	5.4356E-02	Ehnes et al. 2011
4266	Invertebrates	Pterostichus melanarius	1.2029E-01	15	1.1367E-02	Ehnes et al. 2011
4267	Invertebrates	Pterostichus melanarius	1.2297E-01	10	1.2678E-02	Ehnes et al. 2011
4268	Invertebrates	Pterostichus melanarius	1.2351E-01	30	1.2372E-01	Ehnes et al. 2011
4269	Invertebrates	Pterostichus melanarius	1.2740E-01	15	5.7270E-02	Ehnes et al. 2011
4270	Invertebrates	Pterostichus melanarius	1.2761E-01	15	1.6176E-02	Ehnes et al. 2011
4271	Invertebrates	Pterostichus melanarius	1.2764E-01	15	2.0984E-02	Ehnes et al. 2011
4272	Invertebrates	Pterostichus melanarius	1.2972E-01	15	3.7597E-02	Ehnes et al. 2011
4273	Invertebrates	Pterostichus melanarius	1.3041E-01	5	7.4320E-03	Ehnes et al. 2011
4274	Invertebrates	Pterostichus melanarius	1.3118E-01	10	1.2678E-02	Ehnes et al. 2011

4275	Invertebrates	Pterostichus melanarius	1.3527E-01	10	6.5577E-03	Ehnes et al. 2011
4276	Invertebrates	Pterostichus melanarius	1.3590E-01	25	3.3371E-02	Ehnes et al. 2011
4277	Invertebrates	Pterostichus melanarius	1.3601E-01	5	8.4521E-03	Ehnes et al. 2011
4278	Invertebrates	Pterostichus melanarius	1.3603E-01	5	4.8090E-03	Ehnes et al. 2011
4279	Invertebrates	Pterostichus melanarius	1.3774E-01	10	1.3115E-02	Ehnes et al. 2011
4280	Invertebrates	Pterostichus melanarius	1.3976E-01	5	5.2461E-03	Ehnes et al. 2011
4281	Invertebrates	Pterostichus melanarius	1.4132E-01	10	1.4573E-02	Ehnes et al. 2011
4282	Invertebrates	Pterostichus melanarius	1.4145E-01	10	1.3261E-02	Ehnes et al. 2011
4283	Invertebrates	Pterostichus melanarius	1.4188E-01	20	4.9984E-02	Ehnes et al. 2011
4284	Invertebrates	Pterostichus melanarius	1.4246E-01	10	1.2241E-02	Ehnes et al. 2011
4285	Invertebrates	Pterostichus melanarius	1.4282E-01	25	4.2115E-02	Ehnes et al. 2011
4286	Invertebrates	Pterostichus melanarius	1.4360E-01	5	5.2461E-03	Ehnes et al. 2011
4287	Invertebrates	Pterostichus melanarius	1.4491E-01	10	1.4135E-02	Ehnes et al. 2011
4288	Invertebrates	Pterostichus melanarius	1.4586E-01	15	4.0657E-02	Ehnes et al. 2011
4289	Invertebrates	Pterostichus melanarius	1.4892E-01	5	1.0000E-02	Ehnes et al. 2011
4290	Invertebrates	Pterostichus melanarius	1.5355E-01	5	9.3265E-03	Ehnes et al. 2011
4291	Invertebrates	Pterostichus melanarius	1.5434E-01	10	2.3316E-02	Ehnes et al. 2011
4292	Invertebrates	Pterostichus melanarius	1.5536E-01	20	3.8617E-02	Ehnes et al. 2011
4293	Invertebrates	Pterostichus melanarius	1.5542E-01	25	6.6451E-02	Ehnes et al. 2011
4294	Invertebrates	Pterostichus melanarius	1.5588E-01	20	3.9783E-02	Ehnes et al. 2011
4295	Invertebrates	Pterostichus melanarius	1.5614E-01	30	1.0492E-01	Ehnes et al. 2011
4296	Invertebrates	Pterostichus melanarius	1.5660E-01	5	5.9748E-03	Ehnes et al. 2011
4297	Invertebrates	Pterostichus melanarius	1.5664E-01	5	5.1004E-03	Ehnes et al. 2011
4298	Invertebrates	Pterostichus melanarius	1.5778E-01	20	4.6632E-03	Ehnes et al. 2011
4299	Invertebrates	Pterostichus melanarius	1.5797E-01	20	3.6432E-03	Ehnes et al. 2011
4300	Invertebrates	Pterostichus melanarius	1.5924E-01	15	2.8854E-02	Ehnes et al. 2011

4301	Invertebrates	Pterostichus melanarius	1.6039E-01	10	1.6321E-02	Ehnes et al. 2011
4302	Invertebrates	Pterostichus melanarius	1.6175E-01	20	4.5466E-02	Ehnes et al. 2011
4303	Invertebrates	Pterostichus melanarius	1.6208E-01	20	1.1367E-02	Ehnes et al. 2011
4304	Invertebrates	Pterostichus melanarius	1.6243E-01	5	1.0000E-02	Ehnes et al. 2011
4305	Invertebrates	Pterostichus melanarius	1.6387E-01	10	2.3316E-02	Ehnes et al. 2011
4306	Invertebrates	Pterostichus melanarius	1.6435E-01	30	1.3217E-01	Ehnes et al. 2011
4307	Invertebrates	Pterostichus melanarius	1.6620E-01	25	4.9255E-02	Ehnes et al. 2011
4308	Invertebrates	Pterostichus melanarius	1.6681E-01	5	4.3718E-03	Ehnes et al. 2011
4309	Invertebrates	Pterostichus melanarius	1.6696E-01	10	1.0784E-02	Ehnes et al. 2011
4310	Invertebrates	Pterostichus melanarius	1.6718E-01	30	1.3203E-01	Ehnes et al. 2011
4311	Invertebrates	Pterostichus melanarius	1.6834E-01	25	2.7979E-02	Ehnes et al. 2011
4312	Invertebrates	Pterostichus melanarius	1.6838E-01	10	9.3265E-03	Ehnes et al. 2011
4313	Invertebrates	Pterostichus melanarius	1.6972E-01	5	9.5000E-03	Ehnes et al. 2011
4314	Invertebrates	Pterostichus melanarius	1.7056E-01	25	2.0547E-02	Ehnes et al. 2011
4315	Invertebrates	Pterostichus melanarius	1.7096E-01	20	1.5884E-02	Ehnes et al. 2011
4316	Invertebrates	Pterostichus melanarius	1.7166E-01	5	8.5000E-03	Ehnes et al. 2011
4317	Invertebrates	Pterostichus melanarius	1.7207E-01	30	1.6642E-01	Ehnes et al. 2011
4318	Invertebrates	Pterostichus melanarius	1.7244E-01	30	2.8416E-01	Ehnes et al. 2011
4319	Invertebrates	Pterostichus melanarius	1.7327E-01	25	5.5376E-02	Ehnes et al. 2011
4320	Invertebrates	Pterostichus melanarius	1.7373E-01	10	2.6231E-02	Ehnes et al. 2011
4321	Invertebrates	Pterostichus melanarius	1.7445E-01	15	5.3481E-02	Ehnes et al. 2011
4322	Invertebrates	Pterostichus melanarius	1.8092E-01	25	3.5703E-02	Ehnes et al. 2011
4323	Invertebrates	Pterostichus melanarius	1.8913E-01	15	3.6577E-02	Ehnes et al. 2011
4324	Invertebrates	Pterostichus melanarius	1.8931E-01	5	4.6632E-03	Ehnes et al. 2011
4325	Invertebrates	Pterostichus melanarius	1.8952E-01	10	1.1075E-02	Ehnes et al. 2011
4326	Invertebrates	Pterostichus melanarius	1.9164E-01	10	1.6321E-02	Ehnes et al. 2011

4327	Invertebrates	Pterostichus melanarius	1.9251E-01	30	1.5622E-01	Ehnes et al. 2011
4328	Invertebrates	Pterostichus melanarius	1.9470E-01	5	7.1406E-03	Ehnes et al. 2011
4329	Invertebrates	Pterostichus melanarius	1.9681E-01	25	3.1768E-02	Ehnes et al. 2011
4330	Invertebrates	Pterostichus melanarius	1.9988E-01	15	4.4446E-02	Ehnes et al. 2011
4331	Invertebrates	Pterostichus melanarius	2.0297E-01	15	5.9310E-02	Ehnes et al. 2011
4332	Invertebrates	Pterostichus melanarius	2.0387E-01	5	7.8692E-03	Ehnes et al. 2011
4333	Invertebrates	Pterostichus melanarius	2.0586E-01	25	1.7050E-02	Ehnes et al. 2011
4334	Invertebrates	Pterostichus melanarius	2.1061E-01	30	1.2664E-01	Ehnes et al. 2011
4335	Invertebrates	Pterostichus melanarius	2.1148E-01	20	6.6159E-02	Ehnes et al. 2011
4336	Invertebrates	Pterostichus melanarius	2.1206E-01	25	9.9094E-03	Ehnes et al. 2011
4337	Invertebrates	Pterostichus melanarius	2.2833E-01	15	7.0385E-02	Ehnes et al. 2011
4338	Invertebrates	Pterostichus melanarius	2.4920E-01	15	6.5139E-02	Ehnes et al. 2011
4339	Invertebrates	Pterostichus melanarius	2.5426E-01	15	5.7707E-02	Ehnes et al. 2011
4340	Invertebrates	Pterostichus niger	1.5407E-01	10	8.7436E-03	Ehnes et al. 2011
4341	Invertebrates	Pterostichus niger	1.6608E-01	10	1.8653E-02	Ehnes et al. 2011
4342	Invertebrates	Pterostichus niger	1.7508E-01	30	8.2043E-02	Ehnes et al. 2011
4343	Invertebrates	Pterostichus niger	1.7658E-01	15	2.4628E-02	Ehnes et al. 2011
4344	Invertebrates	Pterostichus niger	1.7686E-01	25	1.2270E-01	Ehnes et al. 2011
4345	Invertebrates	Pterostichus niger	1.8966E-01	10	1.6321E-02	Ehnes et al. 2011
4346	Invertebrates	Pterostichus niger	1.9150E-01	20	6.8345E-02	Ehnes et al. 2011
4347	Invertebrates	Pterostichus niger	1.9220E-01	10	1.9673E-02	Ehnes et al. 2011
4348	Invertebrates	Pterostichus niger	1.9393E-01	30	8.5249E-02	Ehnes et al. 2011
4349	Invertebrates	Pterostichus niger	1.9640E-01	10	1.0347E-02	Ehnes et al. 2011
4350	Invertebrates	Pterostichus niger	1.9717E-01	15	3.6869E-02	Ehnes et al. 2011
4351	Invertebrates	Pterostichus niger	2.0119E-01	25	1.0944E-01	Ehnes et al. 2011
4352	Invertebrates	Pterostichus niger	2.0386E-01	25	6.8928E-02	Ehnes et al. 2011

4353	Invertebrates	Pterostichus niger	2.0525E-01	30	8.3355E-02	Ehnes et al. 2011
4354	Invertebrates	Pterostichus niger	2.0647E-01	15	2.4773E-02	Ehnes et al. 2011
4355	Invertebrates	Pterostichus niger	2.0749E-01	25	5.9747E-02	Ehnes et al. 2011
4356	Invertebrates	Pterostichus niger	2.0894E-01	10	1.2532E-02	Ehnes et al. 2011
4357	Invertebrates	Pterostichus niger	2.1011E-01	30	7.8255E-02	Ehnes et al. 2011
4358	Invertebrates	Pterostichus niger	2.1133E-01	10	1.9819E-02	Ehnes et al. 2011
4359	Invertebrates	Pterostichus niger	2.1478E-01	10	1.3261E-02	Ehnes et al. 2011
4360	Invertebrates	Pterostichus niger	2.1632E-01	10	2.1130E-02	Ehnes et al. 2011
4361	Invertebrates	Pterostichus niger	2.1795E-01	25	1.1075E-01	Ehnes et al. 2011
4362	Invertebrates	Pterostichus niger	2.1844E-01	20	9.4139E-02	Ehnes et al. 2011
4363	Invertebrates	Pterostichus niger	2.1848E-01	20	8.5249E-02	Ehnes et al. 2011
4364	Invertebrates	Pterostichus niger	2.1931E-01	20	8.4229E-02	Ehnes et al. 2011
4365	Invertebrates	Pterostichus niger	2.1985E-01	10	1.6758E-02	Ehnes et al. 2011
4366	Invertebrates	Pterostichus niger	2.3323E-01	15	4.4155E-02	Ehnes et al. 2011
4367	Invertebrates	Pterostichus niger	2.3910E-01	15	3.5557E-02	Ehnes et al. 2011
4368	Invertebrates	Pterostichus niger	2.4933E-01	30	9.4430E-02	Ehnes et al. 2011
4369	Invertebrates	Pterostichus niger	2.6707E-01	15	4.7215E-02	Ehnes et al. 2011
4370	Invertebrates	Pterostichus niger	2.7985E-01	20	8.7727E-02	Ehnes et al. 2011
4371	Invertebrates	Pterostichus niger	3.1197E-01	30	9.0058E-02	Ehnes et al. 2011
4372	Invertebrates	Pterostichus niger	3.2289E-01	20	1.5287E-01	Ehnes et al. 2011
4373	Invertebrates	Pterostichus niger	3.3034E-01	15	4.1386E-02	Ehnes et al. 2011
4374	Invertebrates	Pterostichus niger	3.3198E-01	20	1.7239E-01	Ehnes et al. 2011
4375	Invertebrates	Pterostichus niger	3.7818E-01	15	8.0586E-02	Ehnes et al. 2011
4376	Invertebrates	Pterostichus oblongopunctatus	4.0980E-02	10	3.2060E-03	Ehnes et al. 2011
4377	Invertebrates	Pterostichus oblongopunctatus	4.3670E-02	5	1.7487E-03	Ehnes et al. 2011
4378	Invertebrates	Pterostichus oblongopunctatus	4.4170E-02	30	2.3753E-02	Ehnes et al. 2011

4379	Invertebrates	Pterostichus oblongopunctatus	4.5700E-02	22	4.4738E-02	Ehnes et al. 2011
4380	Invertebrates	Pterostichus oblongopunctatus	4.6420E-02	10	2.9145E-03	Ehnes et al. 2011
4381	Invertebrates	Pterostichus oblongopunctatus	4.6500E-02	22	2.2442E-02	Ehnes et al. 2011
4382	Invertebrates	Pterostichus oblongopunctatus	4.8740E-02	20	1.1367E-02	Ehnes et al. 2011
4383	Invertebrates	Pterostichus oblongopunctatus	4.9500E-02	11.5	7.7235E-03	Ehnes et al. 2011
4384	Invertebrates	Pterostichus oblongopunctatus	4.9520E-02	20	2.0402E-03	Ehnes et al. 2011
4385	Invertebrates	Pterostichus oblongopunctatus	5.0020E-02	20	1.8944E-02	Ehnes et al. 2011
4386	Invertebrates	Pterostichus oblongopunctatus	5.0470E-02	10	3.3517E-03	Ehnes et al. 2011
4387	Invertebrates	Pterostichus oblongopunctatus	5.1200E-02	18.5	2.8125E-02	Ehnes et al. 2011
4388	Invertebrates	Pterostichus oblongopunctatus	5.1900E-02	15	2.3025E-02	Ehnes et al. 2011
4389	Invertebrates	Pterostichus oblongopunctatus	5.2008E-02	30	2.0693E-02	Ehnes et al. 2011
4390	Invertebrates	Pterostichus oblongopunctatus	5.2130E-02	10	3.2060E-03	Ehnes et al. 2011
4391	Invertebrates	Pterostichus oblongopunctatus	5.2150E-02	30	4.6632E-02	Ehnes et al. 2011
4392	Invertebrates	Pterostichus oblongopunctatus	5.2220E-02	25	2.2733E-02	Ehnes et al. 2011
4393	Invertebrates	Pterostichus oblongopunctatus	5.3240E-02	10	3.3517E-03	Ehnes et al. 2011
4394	Invertebrates	Pterostichus oblongopunctatus	5.3900E-02	22	4.7215E-02	Ehnes et al. 2011
4395	Invertebrates	Pterostichus oblongopunctatus	5.3960E-02	20	9.6179E-03	Ehnes et al. 2011
4396	Invertebrates	Pterostichus oblongopunctatus	5.5010E-02	20	8.0149E-03	Ehnes et al. 2011
4397	Invertebrates	Pterostichus oblongopunctatus	5.6080E-02	25	1.7924E-02	Ehnes et al. 2011
4398	Invertebrates	Pterostichus oblongopunctatus	5.6870E-02	30	3.4537E-02	Ehnes et al. 2011
4399	Invertebrates	Pterostichus oblongopunctatus	5.8000E-02	22	3.7597E-02	Ehnes et al. 2011
4400	Invertebrates	Pterostichus oblongopunctatus	5.8800E-02	22	3.6140E-02	Ehnes et al. 2011
4401	Invertebrates	Pterostichus oblongopunctatus	5.9330E-02	25	3.2934E-02	Ehnes et al. 2011
4402	Invertebrates	Pterostichus oblongopunctatus	5.9490E-02	5	9.6179E-03	Ehnes et al. 2011
4403	Invertebrates	Pterostichus oblongopunctatus	6.0070E-02	10	3.0603E-03	Ehnes et al. 2011
4404	Invertebrates	Pterostichus oblongopunctatus	6.0460E-02	5	8.3064E-03	Ehnes et al. 2011

4405	Invertebrates	Pterostichus oblongopunctatus	6.0580E-02	25	2.1422E-02	Ehnes et al. 2011
4406	Invertebrates	Pterostichus oblongopunctatus	6.1500E-02	8	7.2863E-03	Ehnes et al. 2011
4407	Invertebrates	Pterostichus oblongopunctatus	6.2100E-02	8	6.1205E-03	Ehnes et al. 2011
4408	Invertebrates	Pterostichus oblongopunctatus	6.2300E-02	8	8.0149E-03	Ehnes et al. 2011
4409	Invertebrates	Pterostichus oblongopunctatus	6.3330E-02	30	3.9492E-02	Ehnes et al. 2011
4410	Invertebrates	Pterostichus oblongopunctatus	6.3750E-02	30	3.7451E-02	Ehnes et al. 2011
4411	Invertebrates	Pterostichus oblongopunctatus	6.4040E-02	20	6.1205E-03	Ehnes et al. 2011
4412	Invertebrates	Pterostichus oblongopunctatus	6.5030E-02	25	3.1477E-02	Ehnes et al. 2011
4413	Invertebrates	Pterostichus oblongopunctatus	6.5510E-02	30	2.3753E-02	Ehnes et al. 2011
4414	Invertebrates	Pterostichus oblongopunctatus	6.5600E-02	18.5	1.4427E-02	Ehnes et al. 2011
4415	Invertebrates	Pterostichus oblongopunctatus	6.5800E-02	11.5	7.2863E-03	Ehnes et al. 2011
4416	Invertebrates	Pterostichus oblongopunctatus	6.5800E-02	18.5	1.5301E-02	Ehnes et al. 2011
4417	Invertebrates	Pterostichus oblongopunctatus	6.5880E-02	10	3.7889E-03	Ehnes et al. 2011
4418	Invertebrates	Pterostichus oblongopunctatus	6.5900E-02	18.5	1.8216E-02	Ehnes et al. 2011
4419	Invertebrates	Pterostichus oblongopunctatus	6.6150E-02	25	2.5211E-02	Ehnes et al. 2011
4420	Invertebrates	Pterostichus oblongopunctatus	6.6700E-02	10	2.7688E-03	Ehnes et al. 2011
4421	Invertebrates	Pterostichus oblongopunctatus	6.6900E-02	15	2.3899E-02	Ehnes et al. 2011
4422	Invertebrates	Pterostichus oblongopunctatus	6.6900E-02	15	2.1130E-02	Ehnes et al. 2011
4423	Invertebrates	Pterostichus oblongopunctatus	6.6910E-02	20	1.0784E-02	Ehnes et al. 2011
4424	Invertebrates	Pterostichus oblongopunctatus	6.7200E-02	22	5.9893E-02	Ehnes et al. 2011
4425	Invertebrates	Pterostichus oblongopunctatus	6.7590E-02	25	3.1768E-02	Ehnes et al. 2011
4426	Invertebrates	Pterostichus oblongopunctatus	6.7600E-02	22	3.4974E-02	Ehnes et al. 2011
4427	Invertebrates	Pterostichus oblongopunctatus	6.7820E-02	30	6.0913E-02	Ehnes et al. 2011
4428	Invertebrates	Pterostichus oblongopunctatus	6.9000E-02	15	2.8416E-02	Ehnes et al. 2011
4429	Invertebrates	Pterostichus oblongopunctatus	7.1200E-02	15	3.0457E-02	Ehnes et al. 2011
4430	Invertebrates	Pterostichus oblongopunctatus	7.1600E-02	8	8.1607E-03	Ehnes et al. 2011

4431	Invertebrates	Pterostichus oblongopunctatus	7.2600E-02	15	2.1422E-02	Ehnes et al. 2011
4432	Invertebrates	Pterostichus oblongopunctatus	7.3000E-02	22	6.3536E-02	Ehnes et al. 2011
4433	Invertebrates	Pterostichus oblongopunctatus	7.3400E-02	11.5	8.4521E-03	Ehnes et al. 2011
4434	Invertebrates	Pterostichus oblongopunctatus	7.3630E-02	30	5.0275E-02	Ehnes et al. 2011
4435	Invertebrates	Pterostichus oblongopunctatus	7.3700E-02	18.5	1.2970E-02	Ehnes et al. 2011
4436	Invertebrates	Pterostichus oblongopunctatus	7.3830E-02	30	5.7853E-02	Ehnes et al. 2011
4437	Invertebrates	Pterostichus oblongopunctatus	7.4100E-02	18.5	1.3698E-02	Ehnes et al. 2011
4438	Invertebrates	Pterostichus oblongopunctatus	7.4680E-02	30	2.4190E-02	Ehnes et al. 2011
4439	Invertebrates	Pterostichus oblongopunctatus	7.4900E-02	8	4.9547E-03	Ehnes et al. 2011
4440	Invertebrates	Pterostichus oblongopunctatus	7.6300E-02	18.5	1.3698E-02	Ehnes et al. 2011
4441	Invertebrates	Pterostichus oblongopunctatus	7.7500E-02	15	2.4482E-02	Ehnes et al. 2011
4442	Invertebrates	Pterostichus oblongopunctatus	8.1000E-02	15	1.8507E-02	Ehnes et al. 2011
4443	Invertebrates	Pterostichus oblongopunctatus	8.1370E-02	20	3.0019E-02	Ehnes et al. 2011
4444	Invertebrates	Pterostichus oblongopunctatus	9.0530E-02	20	2.0693E-02	Ehnes et al. 2011
4445	Invertebrates	Pterostichus oblongopunctatus	9.2950E-02	20	3.3225E-02	Ehnes et al. 2011
4446	Invertebrates	Punctoribates punctum	1.0000E-05	10	2.5500E-06	Ehnes et al. 2011
4447	Invertebrates	Pyrocypris sp.	4.5000E-04	30	1.0125E-03	Makarieva et al. 2008
4448	Invertebrates	Rabidosa rabida	2.0400E-01	20	4.9550E-02	Ehnes et al. 2011
4449	Invertebrates	Rabidosa rabida	2.0700E-01	25	7.8000E-02	Ehnes et al. 2011
4450	Invertebrates	Rabidosa rabida	2.8600E-01	15	5.2000E-02	Ehnes et al. 2011
4451	Invertebrates	Requena verticalis	3.7000E-01	25	1.2294E-01	Ehnes et al. 2011
4452	Invertebrates	Reticulitermes flavipes	2.9700E-03	25	2.1600E-03	Ehnes et al. 2011
4453	Invertebrates	Rhincalanus gigas	9.0000E-03	-1.7	7.2900E-04	Makarieva et al. 2008
4454	Invertebrates	Rhincalanus gigas	1.1692E-02	0	2.1046E-04	Makarieva et al. 2008
4455	Invertebrates	Rhincalanus nasutus	4.1300E-03	6.5	5.4268E-04	Makarieva et al. 2008
4456	Invertebrates	Rhincalanus nasutus	5.1000E-03	6.5	6.2424E-04	Makarieva et al. 2008

4457	Invertebrates	Rhincalanus nasutus	5.4000E-03	6.5	6.4152E-04	Makarieva et al. 2008
4458	Invertebrates	Rhincalanus nasutus	9.0000E-03	20	1.6359E-03	Makarieva et al. 2008
4459	Invertebrates	Rhincalanus nasutus	1.5000E-02	20	2.7972E-03	Makarieva et al. 2008
4460	Invertebrates	Rhincalanus nasutus	2.4000E-02	20	1.5552E-03	Makarieva et al. 2008
4461	Invertebrates	Rhizopertha dominica	1.4000E-03	25	2.6640E-03	Ehnes et al. 2011
4462	Invertebrates	Rhodacarus roseus	7.9000E-06	10	7.3500E-06	Ehnes et al. 2011
4463	Invertebrates	Rhodnius prolixus	6.6750E-02	25	1.6686E-02	Ehnes et al. 2011
4464	Invertebrates	Rhynchaenus flagellum	5.1000E-04	25	3.6000E-05	Ehnes et al. 2011
4465	Invertebrates	Rhysotritia ardua	5.7000E-05	5	1.5500E-06	Ehnes et al. 2011
4466	Invertebrates	Rhysotritia ardua	5.7000E-05	10	3.9000E-06	Ehnes et al. 2011
4467	Invertebrates	Rhysotritia ardua	5.7000E-05	15	7.3500E-06	Ehnes et al. 2011
4468	Invertebrates	Rhytinota praelonga	1.0500E-01	25	1.8000E-02	Ehnes et al. 2011
4469	Invertebrates	Rhytonomus isobellina	8.1000E-03	25	1.6866E-02	Ehnes et al. 2011
4470	Invertebrates	Romalea guttata	2.8740E+00	25	4.8530E-01	Ehnes et al. 2011
4471	Invertebrates	Sabinea septemcarinata	4.1000E+00	0	1.4022E-01	Makarieva et al. 2008
4472	Invertebrates	Sagitta elegans	4.2000E-02	-0.3	1.3986E-03	Makarieva et al. 2008
4473	Invertebrates	Salticidae	2.0000E-03	22	2.6231E-03	Ehnes et al. 2011
4474	Invertebrates	Salticidae	4.0000E-03	15	1.6030E-03	Ehnes et al. 2011
4475	Invertebrates	Salticidae	4.0000E-03	15	1.1658E-03	Ehnes et al. 2011
4476	Invertebrates	Salticidae	7.0000E-03	15	1.3116E-03	Ehnes et al. 2011
4477	Invertebrates	Salticidae	7.0000E-03	22	4.0803E-03	Ehnes et al. 2011
4478	Invertebrates	Salticus scenicus	3.0000E-03	15	3.9346E-03	Ehnes et al. 2011
4479	Invertebrates	Salticus scenicus	3.0000E-03	22	6.8491E-03	Ehnes et al. 2011
4480	Invertebrates	Salticus scenicus	4.0000E-03	15	3.7889E-03	Ehnes et al. 2011
4481	Invertebrates	Salticus scenicus	4.0000E-03	22	8.1607E-03	Ehnes et al. 2011
4482	Invertebrates	Salticus scenicus	4.3400E-03	20	1.2950E-03	Ehnes et al. 2011

4483	Invertebrates	Salticus scenicus	4.4900E-03	20	4.3850E-03	Ehnes et al. 2011
4484	Invertebrates	Salticus scenicus	6.0000E-03	15	3.4974E-03	Ehnes et al. 2011
4485	Invertebrates	Salticus scenicus	6.0000E-03	22	4.3718E-03	Ehnes et al. 2011
4486	Invertebrates	Salticus scenicus	6.0000E-03	22	7.4320E-03	Ehnes et al. 2011
4487	Invertebrates	Salticus scenicus	7.0000E-03	8	3.7889E-03	Ehnes et al. 2011
4488	Invertebrates	Salticus scenicus	7.0000E-03	15	4.0803E-03	Ehnes et al. 2011
4489	Invertebrates	Salticus scenicus	8.0000E-03	8	1.4573E-03	Ehnes et al. 2011
4490	Invertebrates	Salticus scenicus	8.0000E-03	15	3.6432E-03	Ehnes et al. 2011
4491	Invertebrates	Salticus scenicus	8.0000E-03	15	4.6632E-03	Ehnes et al. 2011
4492	Invertebrates	Salticus scenicus	8.0000E-03	22	9.4722E-03	Ehnes et al. 2011
4493	Invertebrates	Salticus scenicus	8.0000E-03	22	9.9094E-03	Ehnes et al. 2011
4494	Invertebrates	Salticus scenicus	8.0000E-03	22	6.7034E-03	Ehnes et al. 2011
4495	Invertebrates	Salticus scenicus	9.0000E-03	8	1.6030E-03	Ehnes et al. 2011
4496	Invertebrates	Salticus scenicus	9.0000E-03	15	2.0402E-03	Ehnes et al. 2011
4497	Invertebrates	Salticus scenicus	9.0000E-03	15	5.3919E-03	Ehnes et al. 2011
4498	Invertebrates	Salticus scenicus	9.0000E-03	22	8.0149E-03	Ehnes et al. 2011
4499	Invertebrates	Salticus scenicus	1.0000E-02	8	2.1859E-03	Ehnes et al. 2011
4500	Invertebrates	Salticus scenicus	1.0000E-02	22	1.2532E-02	Ehnes et al. 2011
4501	Invertebrates	Salticus scenicus	1.1000E-02	8	2.3316E-03	Ehnes et al. 2011
4502	Invertebrates	Salticus scenicus	1.1000E-02	8	4.3718E-03	Ehnes et al. 2011
4503	Invertebrates	Salticus scenicus	1.1000E-02	15	3.4974E-03	Ehnes et al. 2011
4504	Invertebrates	Salticus scenicus	1.1000E-02	22	9.3265E-03	Ehnes et al. 2011
4505	Invertebrates	Salticus scenicus	1.1000E-02	22	9.4722E-03	Ehnes et al. 2011
4506	Invertebrates	Salticus scenicus	2.6300E-02	18.5	5.5376E-03	Ehnes et al. 2011
4507	Invertebrates	Salticus scenicus	3.7100E-02	11.5	6.8491E-03	Ehnes et al. 2011
4508	Invertebrates	Salticus scenicus	3.7100E-02	18.5	9.3265E-03	Ehnes et al. 2011

4509	Invertebrates	Sapphirina gemma	7.1000E-04	29	1.2780E-03	Makarieva et al. 2008
4510	Invertebrates	Sapphirina gemma	1.0000E-03	30	7.6300E-03	Makarieva et al. 2008
4511	Invertebrates	Saprinus sp.	8.2100E-03	25	1.1772E-02	Ehnes et al. 2011
4512	Invertebrates	Sarinda hentzi	4.6000E-03	20	2.0200E-03	Ehnes et al. 2011
4513	Invertebrates	Sassacus vitis	5.7000E-03	22	2.6250E-03	Ehnes et al. 2011
4514	Invertebrates	Scarabaeus flavicornis	3.2200E-01	25	8.7066E-02	Ehnes et al. 2011
4515	Invertebrates	Scarabaeus galenus	1.6810E+00	25	1.5871E-01	Ehnes et al. 2011
4516	Invertebrates	Scarabaeus gariepinus	1.1400E+00	25	5.1840E-02	Ehnes et al. 2011
4517	Invertebrates	Scarabaeus hippocrates	2.0100E+00	25	1.1198E-01	Ehnes et al. 2011
4518	Invertebrates	Scarabaeus rusticus	1.0700E+00	25	1.2532E-01	Ehnes et al. 2011
4519	Invertebrates	Scarabaeus striatum	7.9000E-01	25	5.0868E-02	Ehnes et al. 2011
4520	Invertebrates	Scarabaeus westwoodi	1.7800E+00	25	2.3845E-01	Ehnes et al. 2011
4521	Invertebrates	Schedorhinotermes javanicus	2.7000E-03	25	5.2200E-04	Ehnes et al. 2011
4522	Invertebrates	Schedorhinotermes sarawakensis	8.3000E-03	25	3.2220E-03	Ehnes et al. 2011
4523	Invertebrates	Scheloribates cf. latipes	1.3500E-05	7	5.5000E-07	Ehnes et al. 2011
4524	Invertebrates	Scheloribates cf. latipes	1.3500E-05	11	1.4000E-06	Ehnes et al. 2011
4525	Invertebrates	Scheloribates cf. latipes	1.3500E-05	15	1.4500E-06	Ehnes et al. 2011
4526	Invertebrates	Scheloribates cf. latipes	1.3500E-05	19	2.4000E-06	Ehnes et al. 2011
4527	Invertebrates	Scheloribates cf. latipes	1.3500E-05	24	2.5500E-06	Ehnes et al. 2011
4528	Invertebrates	Scheloribates cf. latipes	1.3500E-05	29	2.8000E-06	Ehnes et al. 2011
4529	Invertebrates	Scheloribates sp.	9.8000E-06	15	2.5500E-06	Ehnes et al. 2011
4530	Invertebrates	Schizocosa mccooki	5.1200E-01	22	8.5500E-02	Ehnes et al. 2011
4531	Invertebrates	Schizocosa sp.	2.3400E-02	20	5.9500E-03	Ehnes et al. 2011
4532	Invertebrates	Sclerocrangon ferox	1.3000E+01	0	1.5678E-01	Makarieva et al. 2008
4533	Invertebrates	Scolecithrix danae	3.0000E-03	20	1.1761E-03	Makarieva et al. 2008
4534	Invertebrates	Scylla serrata	5.0000E-01	20	2.0095E-01	Makarieva et al. 2008

4535	Invertebrates	Scylla serrata	1.0000E+00	20	3.2112E-01	Makarieva et al. 2008
4536	Invertebrates	Scylla serrata	2.0000E+00	20	4.8470E-01	Makarieva et al. 2008
4537	Invertebrates	Scylla serrata	3.0000E+00	20	6.3617E-01	Makarieva et al. 2008
4538	Invertebrates	Scylla serrata	3.5800E+00	20	8.8089E-01	Makarieva et al. 2008
4539	Invertebrates	Scylla serrata	3.5800E+00	27	1.2314E+00	Makarieva et al. 2008
4540	Invertebrates	Scylla serrata	5.0000E+00	20	8.8866E-01	Makarieva et al. 2008
4541	Invertebrates	Scylla serrata	1.0000E+01	20	1.3633E+00	Makarieva et al. 2008
4542	Invertebrates	Sepioteuthis lessoniana	6.8500E+01	5	4.4043E+00	Makarieva et al. 2008
4543	Invertebrates	Sergestes bisulcatus	2.5400E+00	5	1.4630E-01	Makarieva et al. 2008
4544	Invertebrates	Sergestes bisulcatus	2.5400E+00	20	4.2062E-01	Makarieva et al. 2008
4545	Invertebrates	Sergestes bisulcatus	2.5400E+00	10	2.6518E-01	Makarieva et al. 2008
4546	Invertebrates	Sergestes fulgens	1.0600E+00	5	8.9676E-02	Makarieva et al. 2008
4547	Invertebrates	Sergestes fulgens	1.0600E+00	10	1.6218E-01	Makarieva et al. 2008
4548	Invertebrates	Sergestes fulgens	1.0600E+00	20	4.7700E-01	Makarieva et al. 2008
4549	Invertebrates	Sergestes phorcus	3.9510E+00	5.5	8.9609E-02	Makarieva et al. 2008
4550	Invertebrates	Sergestes phorcus	3.9510E+00	5.5	2.9229E-01	Makarieva et al. 2008
4551	Invertebrates	Sergestes similis	5.7000E-01	10	4.9453E-02	Makarieva et al. 2008
4552	Invertebrates	Sergestes similis	5.7000E-01	10	1.6416E-01	Makarieva et al. 2008
4553	Invertebrates	Sergestes tenuiremis	2.6100E+00	5	8.9262E-02	Makarieva et al. 2008
4554	Invertebrates	Sergestes tenuiremis	2.6100E+00	20	2.7718E-01	Makarieva et al. 2008
4555	Invertebrates	Sergestes tenuiremis	2.6100E+00	10	2.3490E-01	Makarieva et al. 2008
4556	Invertebrates	Serolis cornuta	1.4000E+00	3	2.6712E-02	Makarieva et al. 2008
4557	Invertebrates	Serolis cornuta	1.4000E+00	3	1.4969E-01	Makarieva et al. 2008
4558	Invertebrates	Serolis cornuta	1.6400E+00	1.5	2.4206E-02	Makarieva et al. 2008
4559	Invertebrates	Serolis cornuta	1.6400E+00	1.5	1.3638E-01	Makarieva et al. 2008
4560	Invertebrates	Serolis cornuta	2.5900E+00	0	2.6573E-02	Makarieva et al. 2008

4561	Invertebrates	Serolis cornuta	2.5900E+00	0	1.5012E-01	Makarieva et al. 2008
4562	Invertebrates	Serolis cornuta	2.7750E+00	-1.5	2.4476E-02	Makarieva et al. 2008
4563	Invertebrates	Serolis cornuta	2.7750E+00	-1.5	1.3736E-01	Makarieva et al. 2008
4564	Invertebrates	Serolis polita	3.0000E-01	1.5	5.0760E-03	Makarieva et al. 2008
4565	Invertebrates	Serolis polita	3.0500E-01	0	4.5018E-03	Makarieva et al. 2008
4566	Invertebrates	Serolis polita	3.4500E-01	-1.5	4.1607E-03	Makarieva et al. 2008
4567	Invertebrates	Serolis polita	3.5000E-01	3	6.1740E-03	Makarieva et al. 2008
4568	Invertebrates	Sesarma quadratus	5.0000E-01	20	2.1105E-01	Makarieva et al. 2008
4569	Invertebrates	Sesarma quadratus	1.0000E+00	20	3.0395E-01	Makarieva et al. 2008
4570	Invertebrates	Sesarma quadratus	2.0000E+00	20	4.5137E-01	Makarieva et al. 2008
4571	Invertebrates	Sesarma quadratus	3.0000E+00	20	5.6549E-01	Makarieva et al. 2008
4572	Invertebrates	Sesarma quadratus	5.0000E+00	20	7.5231E-01	Makarieva et al. 2008
4573	Invertebrates	Sicyonia igentis	1.9030E+01	15	2.2265E+00	Makarieva et al. 2008
4574	Invertebrates	Sicyonia igentis	1.9260E+01	10	8.3203E-01	Makarieva et al. 2008
4575	Invertebrates	Simplocaria metallica	2.8500E-03	25	9.0000E-05	Ehnes et al. 2011
4576	Invertebrates	Simulium venustum	2.5300E-03	25	1.5480E-02	Ehnes et al. 2011
4577	Invertebrates	Sisiyphys fasciculatus	1.3600E-01	25	6.7518E-02	Ehnes et al. 2011
4578	Invertebrates	Sitophilus granarius	3.6800E-03	25	2.5128E-02	Ehnes et al. 2011
4579	Invertebrates	Slaterocoris sp.	8.6000E-04	25	3.0960E-03	Ehnes et al. 2011
4580	Invertebrates	Smicronyx imbricata	3.2000E-04	25	6.4800E-04	Ehnes et al. 2011
4581	Invertebrates	Sminthurinus	3.0000E-05	6	1.1450E-05	Ehnes et al. 2011
4582	Invertebrates	Sminthurinus	3.0000E-05	10	1.3300E-05	Ehnes et al. 2011
4583	Invertebrates	Sminthurinus	3.0000E-05	15	2.4950E-05	Ehnes et al. 2011
4584	Invertebrates	Sminthurus viridis	1.9000E-03	3	3.3400E-04	Ehnes et al. 2011
4585	Invertebrates	Sminthurus viridis	1.9000E-03	8	5.1000E-04	Ehnes et al. 2011
4586	Invertebrates	Sminthurus viridis	1.9000E-03	18	1.0550E-03	Ehnes et al. 2011

4587	Invertebrates	Sminthurus viridis	1.9000E-03	28	2.0850E-03	Ehnes et al. 2011
4588	Invertebrates	Solenopsis invicta	2.9600E-03	25	1.0080E-03	Ehnes et al. 2011
4589	Invertebrates	Solidosagitta zetesios	7.1000E-02	5	1.1119E-03	Makarieva et al. 2008
4590	Invertebrates	Sosippus janus	3.6000E-01	25	4.0750E-02	Ehnes et al. 2011
4591	Invertebrates	Species 1 (Sutherland)*	2.3040E-02	25	6.6420E-03	Ehnes et al. 2011
4592	Invertebrates	Species 2 (Stellenbosch)*	2.6640E-02	25	4.1400E-03	Ehnes et al. 2011
4593	Invertebrates	Species 3	9.0000E-05	25	5.4000E-05	Ehnes et al. 2011
4594	Invertebrates	Species 3 (Cederberg)*	1.7800E-02	25	7.6140E-03	Ehnes et al. 2011
4595	Invertebrates	Sphaeridium lunatum	3.4000E-02	25	3.1590E-02	Ehnes et al. 2011
4596	Invertebrates	Sphaeriontis dilatata	4.6000E-02	25	2.7126E-02	Ehnes et al. 2011
4597	Invertebrates	Sphaeroderus stenostomus	1.6960E-01	25	4.7178E-02	Ehnes et al. 2011
4598	Invertebrates	Spherillo raffaelei	1.0000E-02	23	1.5050E-03	Ehnes et al. 2011
4599	Invertebrates	Sphingicampa quadrilineata	8.1800E-01	25	3.8416E-01	Ehnes et al. 2011
4600	Invertebrates	Sphinx ligustri	1.4000E+00	25	1.3524E+00	Ehnes et al. 2011
4601	Invertebrates	Sphodromantis gastrica	3.3570E-01	25	1.1009E-01	Ehnes et al. 2011
4602	Invertebrates	Steganacarus magnus	1.6000E-05	18	3.4000E-06	Ehnes et al. 2011
4603	Invertebrates	Steganacarus magnus	2.9800E-05	18	5.3500E-06	Ehnes et al. 2011
4604	Invertebrates	Steganacarus magnus	5.7900E-05	18	1.3000E-05	Ehnes et al. 2011
4605	Invertebrates	Steganacarus magnus	1.0900E-04	18	1.5900E-05	Ehnes et al. 2011
4606	Invertebrates	Steganacarus magnus	1.0900E-04	18	1.0600E-05	Ehnes et al. 2011
4607	Invertebrates	Steganacarus magnus	1.1000E-04	18	9.7000E-06	Ehnes et al. 2011
4608	Invertebrates	Steganacarus magnus	1.6700E-04	10	1.0300E-05	Ehnes et al. 2011
4609	Invertebrates	Steganacarus magnus	1.9100E-04	10	1.0300E-05	Ehnes et al. 2011
4610	Invertebrates	Steganacarus magnus	2.5400E-04	18	1.6750E-05	Ehnes et al. 2011
4611	Invertebrates	Steganacarus magnus	2.9100E-04	18	2.0300E-05	Ehnes et al. 2011
4612	Invertebrates	Steganacarus magnus	2.9100E-04	18	2.0450E-05	Ehnes et al. 2011

4613	Invertebrates	Steganacarus magnus	3.3000E-04	5	5.6500E-06	Ehnes et al. 2011
4614	Invertebrates	Steganacarus magnus	3.3000E-04	10	1.9650E-05	Ehnes et al. 2011
4615	Invertebrates	Steganacarus magnus	3.3000E-04	15	3.6950E-05	Ehnes et al. 2011
4616	Invertebrates	Steganacarus magnus	3.3000E-04	20	6.6500E-05	Ehnes et al. 2011
4617	Invertebrates	Steganacarus magnus	3.6100E-04	25	5.5000E-05	Ehnes et al. 2011
4618	Invertebrates	Steganacarus magnus	3.6100E-04	0	4.0500E-06	Ehnes et al. 2011
4619	Invertebrates	Steganacarus magnus	3.6100E-04	5	8.8500E-06	Ehnes et al. 2011
4620	Invertebrates	Steganacarus magnus	3.6100E-04	10	1.8050E-05	Ehnes et al. 2011
4621	Invertebrates	Steganacarus magnus	3.6100E-04	15	3.0050E-05	Ehnes et al. 2011
4622	Invertebrates	Steganacarus magnus	4.2700E-04	25	4.4650E-05	Ehnes et al. 2011
4623	Invertebrates	Steganacarus magnus	4.3600E-04	18	6.0000E-05	Ehnes et al. 2011
4624	Invertebrates	Steganacarus magnus	4.3700E-04	11	1.7600E-05	Ehnes et al. 2011
4625	Invertebrates	Steganacarus magnus	5.9200E-04	10	2.1800E-05	Ehnes et al. 2011
4626	Invertebrates	Steganacarus spinosus	1.9500E-05	5	2.3500E-06	Ehnes et al. 2011
4627	Invertebrates	Steganacarus spinosus	1.9500E-05	10	2.6000E-06	Ehnes et al. 2011
4628	Invertebrates	Steganacarus spinosus	1.9500E-05	15	3.7000E-06	Ehnes et al. 2011
4629	Invertebrates	Steganacarus spinosus	1.9500E-05	20	5.5500E-06	Ehnes et al. 2011
4630	Invertebrates	Steganacarus striculus	1.8000E-05	10	3.1500E-06	Ehnes et al. 2011
4631	Invertebrates	Stenasellus virei	1.2000E-02	11	6.6960E-04	Makarieva et al. 2008
4632	Invertebrates	Stenocara gracilipes	2.6800E-01	25	8.5734E-02	Ehnes et al. 2011
4633	Invertebrates	Stenodontes molaria	2.9440E+00	25	1.1509E+00	Ehnes et al. 2011
4634	Invertebrates	Stenodontes sp.	1.0430E+00	25	3.6731E-01	Ehnes et al. 2011
4635	Invertebrates	Stereomastis sculpta	1.6760E+01	2.5	7.5420E-02	Makarieva et al. 2008
4636	Invertebrates	Stereotydeus villosus	6.0000E-06	5	1.5000E-06	Ehnes et al. 2011
4637	Invertebrates	Stereotydeus villosus	6.0000E-06	10	1.7500E-06	Ehnes et al. 2011
4638	Invertebrates	Stereotydeus villosus	1.5500E-05	10	9.7000E-06	Ehnes et al. 2011

4639	Invertebrates	Stereotydeus villosus	2.6600E-05	0	3.8500E-06	Ehnes et al. 2011
4640	Invertebrates	Stereotydeus villosus	2.6600E-05	5	8.8500E-06	Ehnes et al. 2011
4641	Invertebrates	Stereotydeus villosus	2.6600E-05	10	9.8500E-06	Ehnes et al. 2011
4642	Invertebrates	Stereotydeus villosus	3.0180E-05	0	3.4000E-06	Ehnes et al. 2011
4643	Invertebrates	Stereotydeus villosus	3.0180E-05	5	6.9500E-06	Ehnes et al. 2011
4644	Invertebrates	Stereotydeus villosus	3.0180E-05	10	9.0000E-06	Ehnes et al. 2011
4645	Invertebrates	Sthenoteuthis oualaniensis	7.5000E+02	5	4.0487E+01	Makarieva et al. 2008
4646	Invertebrates	Sthenoteuthis pteropus	1.3000E+03	5	8.4451E+01	Makarieva et al. 2008
4647	Invertebrates	Strategus aloeus	5.0500E+00	25	9.2693E-01	Ehnes et al. 2011
4648	Invertebrates	Supraphorura furcifera	3.0000E-05	6	8.4000E-06	Ehnes et al. 2011
4649	Invertebrates	Supraphorura furcifera	3.0000E-05	10	1.0650E-05	Ehnes et al. 2011
4650	Invertebrates	Supraphorura furcifera	3.0000E-05	15	2.0850E-05	Ehnes et al. 2011
4651	Invertebrates	Syncapritermes sp.A	6.7000E-03	25	5.5800E-04	Ehnes et al. 2011
4652	Invertebrates	Synopia ultramarina	7.0000E-04	30.2	1.4566E-02	Makarieva et al. 2008
4653	Invertebrates	Syssphinx molina	1.7570E+00	25	7.9771E-01	Ehnes et al. 2011
4654	Invertebrates	Systellaspis cristata	1.3900E+00	5.5	2.0266E-02	Makarieva et al. 2008
4655	Invertebrates	Systellaspis cristata	1.3900E+00	5.5	1.9741E-01	Makarieva et al. 2008
4656	Invertebrates	Systellaspis debilis	1.2200E+00	5	6.5880E-02	Makarieva et al. 2008
4657	Invertebrates	Systellaspis debilis	1.2200E+00	20	2.1301E-01	Makarieva et al. 2008
4658	Invertebrates	Systellaspis debilis	1.2200E+00	10	1.1858E-01	Makarieva et al. 2008
4659	Invertebrates	Tabanus affinis	1.6170E-01	25	2.0610E-02	Ehnes et al. 2011
4660	Invertebrates	Taeinotes scalaris	3.9700E-01	25	1.7141E-01	Ehnes et al. 2011
4661	Invertebrates	Taeniopoda eques	2.0430E+00	25	4.4316E-01	Ehnes et al. 2011
4662	Invertebrates	Talitrus sylvaticus	1.5000E-03	25	6.7500E-04	Makarieva et al. 2008
4663	Invertebrates	Talitrus sylvaticus	8.5000E-03	25	2.1252E-03	Makarieva et al. 2008
4664	Invertebrates	Talitrus sylvaticus	2.1000E-02	25	4.0522E-03	Makarieva et al. 2008

4665	Invertebrates	Talitrus sylvaticus	5.0000E-02	25	7.0020E-03	Makarieva et al. 2008
4666	Invertebrates	Talorchestia megalophtalma	1.1000E-01	22.5	3.6967E-02	Makarieva et al. 2008
4667	Invertebrates	Talorchestia megalophtalma	1.2000E-01	22.5	3.3847E-02	Makarieva et al. 2008
4668	Invertebrates	Talorchestia megalophtalma	1.5500E-01	22.5	3.9981E-02	Makarieva et al. 2008
4669	Invertebrates	Talorchestia megalophtalma	1.9000E-01	22.5	5.0171E-02	Makarieva et al. 2008
4670	Invertebrates	Talorchestia megalophtalma	2.0000E-01	22.5	4.6800E-02	Makarieva et al. 2008
4671	Invertebrates	Talorchestia megalophtalma	2.2500E-01	22.5	4.9937E-02	Makarieva et al. 2008
4672	Invertebrates	Talorchestia megalophtalma	2.3000E-01	22.5	4.5540E-02	Makarieva et al. 2008
4673	Invertebrates	Talorchestia megalophtalma	2.7800E-01	22.5	5.6996E-02	Makarieva et al. 2008
4674	Invertebrates	Talorchestia megalophtalma	2.9100E-01	22.5	5.0337E-02	Makarieva et al. 2008
4675	Invertebrates	Talorchestia megalophtalma	3.8800E-01	22.5	5.8596E-02	Makarieva et al. 2008
4676	Invertebrates	Tanaocerus koebeli	1.0250E-01	25	1.1266E-01	Ehnes et al. 2011
4677	Invertebrates	Tectocepheus velatus	4.2000E-06	5	2.5000E-07	Ehnes et al. 2011
4678	Invertebrates	Tectocepheus velatus	4.2000E-06	10	4.5000E-07	Ehnes et al. 2011
4679	Invertebrates	Tectocepheus velatus	4.2000E-06	15	9.5000E-07	Ehnes et al. 2011
4680	Invertebrates	Teleogryllus commodus	9.5000E-01	25	1.0236E+00	Ehnes et al. 2011
4681	Invertebrates	Temora longicornis	6.1000E-05	29	5.3099E-04	Makarieva et al. 2008
4682	Invertebrates	Tenebrio molitor	1.0000E-01	25	6.6996E-02	Ehnes et al. 2011
4683	Invertebrates	Tenuiphantes zimmernanni	4.0000E-03	9	6.5500E-04	Ehnes et al. 2011
4684	Invertebrates	Tenuiphantes zimmernanni	4.0000E-03	7	5.5000E-04	Ehnes et al. 2011
4685	Invertebrates	Tenuiphantes zimmernanni	4.0000E-03	5	4.9550E-04	Ehnes et al. 2011
4686	Invertebrates	Tenuiphantes zimmernanni	4.0000E-03	3	3.9900E-04	Ehnes et al. 2011
4687	Invertebrates	Tenuiphantes zimmernanni	4.0000E-03	3	3.8750E-04	Ehnes et al. 2011
4688	Invertebrates	Tenuiphantes zimmernanni	4.0000E-03	10	6.9500E-04	Ehnes et al. 2011
4689	Invertebrates	Tenuiphantes zimmernanni	4.0000E-03	10	8.1000E-04	Ehnes et al. 2011
4690	Invertebrates	Tenuiphantes zimmernanni	4.0000E-03	13	9.1500E-04	Ehnes et al. 2011

4691	Invertebrates	Tenuiphantes zimmernanni	4.0000E-03	14	9.5500E-04	Ehnes et al. 2011
4692	Invertebrates	Tenuiphantes zimmernanni	4.0000E-03	4	4.2700E-04	Ehnes et al. 2011
4693	Invertebrates	Tenuiphantes zimmernanni	4.0000E-03	15	9.9500E-04	Ehnes et al. 2011
4694	Invertebrates	Termes borneensis	3.0000E-03	25	3.7800E-04	Ehnes et al. 2011
4695	Invertebrates	Tetramorium caespitum	6.9000E-04	25	4.3200E-04	Ehnes et al. 2011
4696	Invertebrates	Tetranychus cinnabarinus	4.9400E-06	25	3.3000E-06	Ehnes et al. 2011
4697	Invertebrates	Tetranychus cinnabarinus	9.9800E-06	25	1.2550E-05	Ehnes et al. 2011
4698	Invertebrates	Tetrodontophora bielanensis	4.8000E-03	3	1.9900E-04	Ehnes et al. 2011
4699	Invertebrates	Tetrodontophora bielanensis	4.8000E-03	8	3.1500E-04	Ehnes et al. 2011
4700	Invertebrates	Tetrodontophora bielanensis	4.8000E-03	18	8.0000E-04	Ehnes et al. 2011
4701	Invertebrates	Tetrodontophora bielanensis	6.6100E-03	18	1.1000E-03	Ehnes et al. 2011
4702	Invertebrates	Tetrorchis erythrogaster	4.4000E-01	5	1.1880E-03	Makarieva et al. 2008
4703	Invertebrates	Thais cassandra	1.3000E-01	25	6.2791E-01	Ehnes et al. 2011
4704	Invertebrates	Thermophilum babaulti	1.2950E+00	25	5.8030E-01	Ehnes et al. 2011
4705	Invertebrates	Thermophilum hexastictum	2.0250E+00	25	4.3618E-01	Ehnes et al. 2011
4706	Invertebrates	Thiodana sylvana	6.7000E-02	20	1.3900E-02	Ehnes et al. 2011
4707	Invertebrates	Thysanoessa inermis	8.0100E-03	11.45	3.3248E-03	Makarieva et al. 2008
4708	Invertebrates	Thysanoessa inermis	1.7000E-02	0.1	3.0722E-03	Makarieva et al. 2008
4709	Invertebrates	Thysanoessa inermis	1.0800E-01	1.9	1.3802E-02	Makarieva et al. 2008
4710	Invertebrates	Thysanoessa macrura	6.0000E-03	-1	3.5964E-04	Makarieva et al. 2008
4711	Invertebrates	Thysanoessa macrura	9.0000E-03	-1	5.5728E-04	Makarieva et al. 2008
4712	Invertebrates	Thysanoessa macrura	3.4000E-02	2.6	3.6720E-03	Makarieva et al. 2008
4713	Invertebrates	Thysanoessa macrura	5.0000E-02	-0.5	1.5030E-03	Makarieva et al. 2008
4714	Invertebrates	Thysanoessa macrura	5.0000E-02	0.7	3.6000E-03	Makarieva et al. 2008
4715	Invertebrates	Thysanoessa macrura	5.0000E-02	-0.5	4.5990E-03	Makarieva et al. 2008
4716	Invertebrates	Thysanoessa macrura	9.0000E-02	1	8.3754E-03	Makarieva et al. 2008

4717	Invertebrates	Thysanoessa raschii	4.6600E-02	6.7	1.0812E-02	Makarieva et al. 2008
4718	Invertebrates	Thysanoessa raschii	1.0700E-01	6.75	1.6371E-02	Makarieva et al. 2008
4719	Invertebrates	Thysanoessa raschii	1.0800E-01	6.75	2.1054E-02	Makarieva et al. 2008
4720	Invertebrates	Thysanopoda cornuta	5.8800E+00	5	2.2226E-01	Makarieva et al. 2008
4721	Invertebrates	Tomocerus vulgaris	1.2800E-03	8	1.6600E-04	Ehnes et al. 2011
4722	Invertebrates	Tomocerus vulgaris	1.2800E-03	18	4.7000E-04	Ehnes et al. 2011
4723	Invertebrates	Tomocerus vulgaris	1.6200E-03	18	5.9500E-04	Ehnes et al. 2011
4724	Invertebrates	Tortanus discaudatus	2.0000E-04	11.15	2.2821E-04	Makarieva et al. 2008
4725	Invertebrates	Tortanus discaudatus	2.8500E-04	7	1.2004E-04	Makarieva et al. 2008
4726	Invertebrates	Tortanus gracilis	1.2000E-04	26	1.4299E-04	Makarieva et al. 2008
4727	Invertebrates	Trachelipus rathkii	2.6060E-02	20	4.3718E-03	Ehnes et al. 2011
4728	Invertebrates	Trachelipus rathkii	2.6270E-02	20	6.8491E-03	Ehnes et al. 2011
4729	Invertebrates	Trachelipus rathkii	2.6970E-02	15	4.9547E-03	Ehnes et al. 2011
4730	Invertebrates	Trachelipus rathkii	2.9370E-02	20	1.0201E-02	Ehnes et al. 2011
4731	Invertebrates	Trachelipus rathkii	3.3650E-02	20	6.1205E-03	Ehnes et al. 2011
4732	Invertebrates	Trachelipus rathkii	3.6380E-02	15	3.7889E-03	Ehnes et al. 2011
4733	Invertebrates	Trachelipus rathkii	3.7100E-02	10	2.4774E-03	Ehnes et al. 2011
4734	Invertebrates	Trachelipus rathkii	3.7600E-02	15	4.0803E-03	Ehnes et al. 2011
4735	Invertebrates	Trachelipus rathkii	4.2090E-02	20	1.1221E-02	Ehnes et al. 2011
4736	Invertebrates	Trachelipus rathkii	4.5250E-02	15	4.9547E-03	Ehnes et al. 2011
4737	Invertebrates	Trachelipus rathkii	4.6110E-02	20	6.5577E-03	Ehnes et al. 2011
4738	Invertebrates	Trachelipus rathkii	4.6820E-02	15	3.4974E-03	Ehnes et al. 2011
4739	Invertebrates	Trachelipus rathkii	5.0680E-02	20	6.7034E-03	Ehnes et al. 2011
4740	Invertebrates	Trachysomus peregrinus	5.4700E-01	25	1.0868E-01	Ehnes et al. 2011
4741	Invertebrates	Trachytes pyriformis	1.8000E-05	10	4.0500E-06	Ehnes et al. 2011
4742	Invertebrates	Tramea carolina	3.8300E-01	25	3.2310E-01	Ehnes et al. 2011

4743	Invertebrates	Triaenogeius scupturatus	1.7000E-01	25	6.1524E-02	Ehnes et al. 2011
4744	Invertebrates	Tribolium castaneum	2.4000E-03	25	2.3400E-03	Ehnes et al. 2011
4745	Invertebrates	Tribolium confusum	2.0100E-03	25	2.9340E-03	Ehnes et al. 2011
4746	Invertebrates	Trichoniscus pusillus	1.2000E-03	5	1.5750E-04	Ehnes et al. 2011
4747	Invertebrates	Trichoniscus pusillus	1.2000E-03	10	2.5450E-04	Ehnes et al. 2011
4748	Invertebrates	Trichoniscus pusillus	1.2000E-03	15	3.8800E-04	Ehnes et al. 2011
4749	Invertebrates	Trimerotropis pallidipennis	2.2135E-01	25	1.3379E-01	Ehnes et al. 2011
4750	Invertebrates	Trimerotropis saxatilis	1.5500E-01	25	5.7924E-02	Ehnes et al. 2011
4751	Invertebrates	Trimerotropis sp.	1.4590E-01	25	1.8135E-01	Ehnes et al. 2011
4752	Invertebrates	Trimerotropis suffusa	2.9750E-01	25	1.6312E-01	Ehnes et al. 2011
4753	Invertebrates	Triops cancriformis	1.6000E-01	20	7.2000E-02	Makarieva et al. 2008
4754	Invertebrates	Triops cancriformis	1.9000E-01	20	7.5240E-02	Makarieva et al. 2008
4755	Invertebrates	Triops cancriformis	2.3000E-01	20	4.5540E-02	Makarieva et al. 2008
4756	Invertebrates	Triops cancriformis	3.4000E-01	20	7.3440E-02	Makarieva et al. 2008
4757	Invertebrates	Triops cancriformis	4.1000E-01	20	9.1512E-02	Makarieva et al. 2008
4758	Invertebrates	Triorophus laevis	8.4600E-03	25	1.9386E-02	Ehnes et al. 2011
4759	Invertebrates	Trochosa	2.7400E-03	15	1.6030E-03	Ehnes et al. 2011
4760	Invertebrates	Trochosa	3.0200E-03	25	5.5376E-03	Ehnes et al. 2011
4761	Invertebrates	Trochosa	3.3800E-03	15	1.6030E-03	Ehnes et al. 2011
4762	Invertebrates	Trochosa	3.7100E-03	30	8.0149E-03	Ehnes et al. 2011
4763	Invertebrates	Trochosa	4.0100E-03	30	9.1807E-03	Ehnes et al. 2011
4764	Invertebrates	Trochosa	4.1100E-03	30	6.7034E-03	Ehnes et al. 2011
4765	Invertebrates	Trochosa	4.1500E-03	15	1.4573E-03	Ehnes et al. 2011
4766	Invertebrates	Trochosa	4.2800E-03	20	4.0803E-03	Ehnes et al. 2011
4767	Invertebrates	Trochosa	4.2800E-03	20	2.9145E-03	Ehnes et al. 2011
4768	Invertebrates	Trochosa	4.3400E-03	20	3.4974E-03	Ehnes et al. 2011

4769	Invertebrates	Trochosa	4.3700E-03	15	1.8945E-03	Ehnes et al. 2011
4770	Invertebrates	Trochosa	4.5100E-03	30	9.3265E-03	Ehnes et al. 2011
4771	Invertebrates	Trochosa	4.6200E-03	20	3.4974E-03	Ehnes et al. 2011
4772	Invertebrates	Trochosa	4.6500E-03	15	1.8945E-03	Ehnes et al. 2011
4773	Invertebrates	Trochosa	4.7900E-03	15	2.0402E-03	Ehnes et al. 2011
4774	Invertebrates	Trochosa	4.8200E-03	30	7.8692E-03	Ehnes et al. 2011
4775	Invertebrates	Trochosa	4.9800E-03	25	6.1205E-03	Ehnes et al. 2011
4776	Invertebrates	Trochosa	5.0000E-03	20	3.6432E-03	Ehnes et al. 2011
4777	Invertebrates	Trochosa	5.2100E-03	15	1.8945E-03	Ehnes et al. 2011
4778	Invertebrates	Trochosa	5.2200E-03	20	4.2261E-03	Ehnes et al. 2011
4779	Invertebrates	Trochosa	5.2300E-03	25	6.5577E-03	Ehnes et al. 2011
4780	Invertebrates	Trochosa	5.2700E-03	15	1.6030E-03	Ehnes et al. 2011
4781	Invertebrates	Trochosa	5.3000E-03	20	3.2060E-03	Ehnes et al. 2011
4782	Invertebrates	Trochosa	5.4000E-03	30	5.6833E-03	Ehnes et al. 2011
4783	Invertebrates	Trochosa	5.4400E-03	15	2.1859E-03	Ehnes et al. 2011
4784	Invertebrates	Trochosa	5.4900E-03	20	4.0803E-03	Ehnes et al. 2011
4785	Invertebrates	Trochosa	5.5300E-03	30	9.6179E-03	Ehnes et al. 2011
4786	Invertebrates	Trochosa	5.6100E-03	25	4.9547E-03	Ehnes et al. 2011
4787	Invertebrates	Trochosa	5.7000E-03	25	6.1205E-03	Ehnes et al. 2011
4788	Invertebrates	Trochosa	5.9800E-03	30	9.9094E-03	Ehnes et al. 2011
4789	Invertebrates	Trochosa	6.0600E-03	15	3.2060E-03	Ehnes et al. 2011
4790	Invertebrates	Trochosa	6.2900E-03	30	1.0929E-02	Ehnes et al. 2011
4791	Invertebrates	Trochosa	6.3100E-03	25	7.7235E-03	Ehnes et al. 2011
4792	Invertebrates	Trochosa	6.4400E-03	25	7.2863E-03	Ehnes et al. 2011
4793	Invertebrates	Trochosa	6.6600E-03	30	9.4722E-03	Ehnes et al. 2011
4794	Invertebrates	Trochosa	7.0300E-02	11.5	1.1804E-02	Ehnes et al. 2011

4795	Invertebrates	Trochosa	7.0300E-02	15	1.2678E-02	Ehnes et al. 2011
4796	Invertebrates	Trochosa	7.0300E-02	18.5	1.3552E-02	Ehnes et al. 2011
4797	Invertebrates	Trochosa	8.2900E-02	11.5	8.5978E-03	Ehnes et al. 2011
4798	Invertebrates	Trochosa	8.2900E-02	15	9.0350E-03	Ehnes et al. 2011
4799	Invertebrates	Trochosa	8.2900E-02	18.5	5.3919E-03	Ehnes et al. 2011
4800	Invertebrates	Trochosa	8.3900E-02	11.5	1.1367E-02	Ehnes et al. 2011
4801	Invertebrates	Trochosa	8.3900E-02	15	1.2532E-02	Ehnes et al. 2011
4802	Invertebrates	Trochosa	8.3900E-02	18.5	1.3261E-02	Ehnes et al. 2011
4803	Invertebrates	Trochosa	9.7700E-02	11.5	1.1221E-02	Ehnes et al. 2011
4804	Invertebrates	Trochosa	9.7700E-02	15	1.1075E-02	Ehnes et al. 2011
4805	Invertebrates	Trochosa	9.7700E-02	18.5	1.1075E-02	Ehnes et al. 2011
4806	Invertebrates	Trochosa	1.0970E-01	11.5	1.1075E-02	Ehnes et al. 2011
4807	Invertebrates	Trochosa	1.0970E-01	15	1.1512E-02	Ehnes et al. 2011
4808	Invertebrates	Trochosa	1.0970E-01	18.5	1.1804E-02	Ehnes et al. 2011
4809	Invertebrates	Trochosa	1.7800E-01	11.5	1.4135E-02	Ehnes et al. 2011
4810	Invertebrates	Trochosa	1.7800E-01	15	1.8507E-02	Ehnes et al. 2011
4811	Invertebrates	Trochosa	1.7800E-01	18.5	2.1130E-02	Ehnes et al. 2011
4812	Invertebrates	Trochosa ruricola	1.0100E-01	20	1.8300E-02	Ehnes et al. 2011
4813	Invertebrates	Trochosa ruricola	1.2100E-01	20	3.1650E-02	Ehnes et al. 2011
4814	Invertebrates	Trogloderus costatus	2.9870E-02	25	1.6758E-02	Ehnes et al. 2011
4815	Invertebrates	Tydeus tilbrooki	1.5000E-06	0	1.5000E-07	Ehnes et al. 2011
4816	Invertebrates	Tydeus tilbrooki	1.5000E-06	10	4.5000E-07	Ehnes et al. 2011
4817	Invertebrates	Tydeus tilbrooki	1.9000E-06	0	6.5000E-07	Ehnes et al. 2011
4818	Invertebrates	Tydeus tilbrooki	1.9000E-06	5	7.5000E-07	Ehnes et al. 2011
4819	Invertebrates	Tydeus tilbrooki	1.9000E-06	10	9.5000E-07	Ehnes et al. 2011
4820	Invertebrates	Uca leptodactyla	2.7000E-01	20	1.8278E-02	Makarieva et al. 2008

4821	Invertebrates	Uca minax	6.3700E+00	20	2.1912E-01	Makarieva et al. 2008
4822	Invertebrates	Uca mordax	2.5800E+00	20	1.5650E-01	Makarieva et al. 2008
4823	Invertebrates	Uca pugnas	2.2100E+00	20	7.0769E-02	Makarieva et al. 2008
4824	Invertebrates	Uca pugnas	2.2600E+00	20	9.3198E-02	Makarieva et al. 2008
4825	Invertebrates	Uca pugnas	2.2700E+00	20	1.3631E-01	Makarieva et al. 2008
4826	Invertebrates	Uca pugnas	2.6100E+00	20	1.3732E-01	Makarieva et al. 2008
4827	Invertebrates	Uca pugnas	3.0000E+00	20	1.6357E-01	Makarieva et al. 2008
4828	Invertebrates	Uca rapax	2.4800E+00	20	1.8785E-01	Makarieva et al. 2008
4829	Invertebrates	Uca rapax	3.8500E+00	20	2.5648E-01	Makarieva et al. 2008
4830	Invertebrates	Uca thayeri	4.6500E+00	20	2.1611E-01	Makarieva et al. 2008
4831	Invertebrates	Undinula vulgaris	8.3800E-04	27.6	1.4028E-03	Makarieva et al. 2008
4832	Invertebrates	Undinula vulgaris	9.2500E-04	23.5	9.6570E-04	Makarieva et al. 2008
4833	Invertebrates	Unknown	1.6000E-04	25	1.0080E-03	Ehnes et al. 2011
4834	Invertebrates	Unknown	1.6000E-04	25	6.3000E-04	Ehnes et al. 2011
4835	Invertebrates	Unknown	5.1000E-04	25	2.1600E-03	Ehnes et al. 2011
4836	Invertebrates	Unknown	9.5000E-04	25	5.0580E-03	Ehnes et al. 2011
4837	Invertebrates	Unknown	1.7000E-03	25	5.0040E-03	Ehnes et al. 2011
4838	Invertebrates	Unknown	1.8900E-03	25	4.6620E-03	Ehnes et al. 2011
4839	Invertebrates	Unknown	3.5600E-03	25	1.3122E-02	Ehnes et al. 2011
4840	Invertebrates	Unknown	3.8500E-03	25	6.3000E-03	Ehnes et al. 2011
4841	Invertebrates	Unknown	1.2750E-02	25	6.8940E-03	Ehnes et al. 2011
4842	Invertebrates	Unknown	1.4140E-02	25	3.6360E-02	Ehnes et al. 2011
4843	Invertebrates	Unknown	6.6700E-02	25	7.3890E-02	Ehnes et al. 2011
4844	Invertebrates	Urozelotes rusticus	5.2500E-02	22	6.3500E-03	Ehnes et al. 2011
4845	Invertebrates	Valdiviella oligartha	1.9400E-02	5	6.5650E-04	Makarieva et al. 2008
4846	Invertebrates	Vallentinia adherens	2.6000E-02	15	1.1232E-03	Makarieva et al. 2008

4847	Invertebrates	Vampyrocrossota childressi	3.4000E-01	5	1.0404E-03	Makarieva et al. 2008
4848	Invertebrates	Vampyroteuthis infernalis	1.0500E+03	5	5.8590E-01	Makarieva et al. 2008
4849	Invertebrates	Vanessa io	2.3000E-01	25	1.6600E-01	Ehnes et al. 2011
4850	Invertebrates	Viblia antarctica	6.1000E-02	-1.1	1.2078E-03	Makarieva et al. 2008
4851	Invertebrates	Vieta bulbifera	4.7900E-01	25	9.6984E-02	Ehnes et al. 2011
4852	Invertebrates	Vieta muscosa	1.4000E-01	25	2.4372E-02	Ehnes et al. 2011
4853	Invertebrates	Waldeckia obesa	2.1800E-01	0	5.8860E-03	Makarieva et al. 2008
4854	Invertebrates	Waldeckia obesa	2.2900E-01	-1	1.1006E-02	Makarieva et al. 2008
4855	Invertebrates	Waldeckia obesa	2.3800E-01	1	6.8972E-03	Makarieva et al. 2008
4856	Invertebrates	Waldeckia obesa	2.5200E-01	2	9.5710E-03	Makarieva et al. 2008
4857	Invertebrates	Waldeckia obesa	2.9500E-01	-1.8	1.1523E-02	Makarieva et al. 2008
4858	Invertebrates	Xanthippus corallipes	2.3022E+00	25	1.3515E+00	Ehnes et al. 2011
4859	Invertebrates	Xantho hydrophilus	9.0600E-01	20	8.8357E-02	Makarieva et al. 2008
4860	Invertebrates	Xantho hydrophilus	1.9100E+00	20	1.4845E-01	Makarieva et al. 2008
4861	Invertebrates	Xantho hydrophilus	2.5000E+00	20	1.6763E-01	Makarieva et al. 2008
4862	Invertebrates	Xantho hydrophilus	3.0600E+00	20	1.9592E-01	Makarieva et al. 2008
4863	Invertebrates	Xantho hydrophilus	3.9000E+00	20	2.6964E-01	Makarieva et al. 2008
4864	Invertebrates	Xantho hydrophilus	4.6100E+00	20	2.8578E-01	Makarieva et al. 2008
4865	Invertebrates	Xantho hydrophilus	5.8000E+00	20	2.9180E-01	Makarieva et al. 2008
4866	Invertebrates	Xantho hydrophilus	6.2000E+00	20	3.8167E-01	Makarieva et al. 2008
4867	Invertebrates	Xantho hydrophilus	8.3100E+00	20	3.4837E-01	Makarieva et al. 2008
4868	Invertebrates	Xantho hydrophilus	1.1300E+01	20	5.9271E-01	Makarieva et al. 2008
4869	Invertebrates	Xantho hydrophilus	1.3700E+01	20	6.8974E-01	Makarieva et al. 2008
4870	Invertebrates	Xantho hydrophilus	1.6520E+01	20	7.2794E-01	Makarieva et al. 2008
4871	Invertebrates	Xenambyx laticauda	1.2170E+00	25	2.8294E-01	Ehnes et al. 2011
4872	Invertebrates	Xenillus tegeocranus	1.4000E-04	5	7.9000E-06	Ehnes et al. 2011

4873	Invertebrates	Xenillus tegeocranus	1.4000E-04	10	1.3000E-05	Ehnes et al. 2011
4874	Invertebrates	Xenillus tegeocranus	1.4000E-04	15	2.2500E-05	Ehnes et al. 2011
4875	Invertebrates	Xenillus tegeocranus	1.4000E-04	20	4.1300E-05	Ehnes et al. 2011
4876	Invertebrates	Xenopsylla ramesis	1.6000E-04	25	1.0800E-04	Ehnes et al. 2011
4877	Invertebrates	Xiphoceriana sp.	1.5680E+00	25	1.3374E-01	Ehnes et al. 2011
4878	Invertebrates	Xylocopa capitata	1.3000E+00	25	1.2304E+00	Ehnes et al. 2011
4879	Invertebrates	Xylophanes chiron	7.0800E-01	25	2.4268E-01	Ehnes et al. 2011
4880	Invertebrates	Xylophanes libya	5.5900E-01	25	4.5925E-01	Ehnes et al. 2011
4881	Invertebrates	Xylophanes pluto	8.2900E-01	25	4.0648E-01	Ehnes et al. 2011
4882	Invertebrates	Xysticus funestus	2.9900E-02	20	5.8500E-03	Ehnes et al. 2011
4883	Invertebrates	Zophobas sp.	5.3500E-01	25	2.2853E-01	Ehnes et al. 2011
4884	Invertebrates	Zophosis orbicularis	1.0300E-01	25	2.1852E-02	Ehnes et al. 2011
4885	Invertebrates	Zygoballus rufipes	3.0000E-03	20	6.0500E-04	Ehnes et al. 2011
4886	Ecto.verts	Acanthodactylus boskianus	7.8000E+00	40	2.3400E+00	White et al. 2006
4887	Ecto.verts	Acanthodactylus boskianus	1.1640E+01	25	2.2523E+00	White et al. 2006
4888	Ecto.verts	Acanthodactylus boskianus	1.1640E+01	30	2.6190E+00	White et al. 2006
4889	Ecto.verts	Acanthodactylus boskianus	1.1640E+01	35	3.2825E+00	White et al. 2006
4890	Ecto.verts	Acanthodactylus boskianus	1.5480E+01	15	6.6564E-01	White et al. 2006
4891	Ecto.verts	Acanthodactylus boskianus	1.5480E+01	10	8.5140E-01	White et al. 2006
4892	Ecto.verts	Acanthodactylus boskianus	1.5480E+01	20	1.9505E+00	White et al. 2006
4893	Ecto.verts	Acanthodactylus erythrurus	9.0000E+00	20	1.1700E+00	White et al. 2006
4894	Ecto.verts	Acanthodactylus erythrurus	9.0000E+00	25	1.6200E+00	White et al. 2006
4895	Ecto.verts	Acanthodactylus erythrurus	9.0000E+00	30	2.2500E+00	White et al. 2006
4896	Ecto.verts	Acanthodactylus erythrurus	9.0000E+00	35	3.1500E+00	White et al. 2006
4897	Ecto.verts	Acanthodactylus opheodurus	3.8000E+00	20	3.8000E-01	White et al. 2006
4898	Ecto.verts	Acanthodactylus opheodurus	3.8000E+00	30	7.9040E-01	White et al. 2006

4899	Ecto.verts	Acanthodactylus opheodurus	3.8000E+00	35	1.3794E+00	White et al. 2006
4900	Ecto.verts	Acanthodactylus pardalis	9.7000E+00	25	3.4823E+00	White et al. 2006
4901	Ecto.verts	Acanthodactylus pardalis	9.7000E+00	30	3.8800E+00	White et al. 2006
4902	Ecto.verts	Acanthodactylus pardalis	9.7000E+00	35	4.5590E+00	White et al. 2006
4903	Ecto.verts	Acanthodactylus pardalis	9.7000E+00	40	6.7900E+00	White et al. 2006
4904	Ecto.verts	Acanthodactylus schmidti	1.4500E+01	20	1.0150E+00	White et al. 2006
4905	Ecto.verts	Acanthodactylus schmidti	1.4500E+01	30	2.1895E+00	White et al. 2006
4906	Ecto.verts	Acanthodactylus schmidti	1.4500E+01	35	3.2190E+00	White et al. 2006
4907	Ecto.verts	Acanthodactylus schreiberi	1.0900E+01	25	1.6241E+00	White et al. 2006
4908	Ecto.verts	Acanthodactylus schreiberi	1.0900E+01	30	2.1800E+00	White et al. 2006
4909	Ecto.verts	Acanthodactylus schreiberi	1.0900E+01	35	2.7141E+00	White et al. 2006
4910	Ecto.verts	Acanthodactylus schreiberi	1.0900E+01	40	3.0411E+00	White et al. 2006
4911	Ecto.verts	Acanthodactylus scutellatus	6.6000E+00	25	1.0560E+00	White et al. 2006
4912	Ecto.verts	Acanthodactylus scutellatus	6.6000E+00	30	1.2474E+00	White et al. 2006
4913	Ecto.verts	Acanthodactylus scutellatus	6.6000E+00	35	2.3760E+00	White et al. 2006
4914	Ecto.verts	Acanthodactylus scutellatus	6.6000E+00	40	3.0360E+00	White et al. 2006
4915	Ecto.verts	Acanthophis praelongus	1.0550E+02	24	3.7980E+00	White et al. 2006
4916	Ecto.verts	Acanthophis praelongus	1.0550E+02	30	4.9585E+00	White et al. 2006
4917	Ecto.verts	Acanthophis praelongus	1.0550E+02	27	5.3805E+00	White et al. 2006
4918	Ecto.verts	Acanthophis praelongus	1.0550E+02	33	7.3850E+00	White et al. 2006
4919	Ecto.verts	Acipenser transmontanus	9.0000E+02	10	2.7082E+01	FishBase
4920	Ecto.verts	Acipenser transmontanus	9.0000E+02	15	4.9125E+01	FishBase
4921	Ecto.verts	Acipenser transmontanus	9.5000E+02	15	5.0525E+01	FishBase
4922	Ecto.verts	Acontias meleagris	7.3000E+00	23	4.0880E-01	White et al. 2006
4923	Ecto.verts	Acontias meleagris	7.3000E+00	33	6.7890E-01	White et al. 2006
4924	Ecto.verts	Acrantophis dumerili	2.5485E+03	20	1.9389E+01	White et al. 2006

4925	Ecto.verts	Acrantophis dumerili	2.5485E+03	30	4.8225E+01	White et al. 2006
4926	Ecto.verts	Acrantophis dumerili	2.5485E+03	34	7.5323E+01	White et al. 2006
4927	Ecto.verts	Acris crepitans	5.0000E-01	15	3.3500E-02	White et al. 2006
4928	Ecto.verts	Acris crepitans	5.0000E-01	25	1.1700E-01	White et al. 2006
4929	Ecto.verts	Acris crepitans	9.0000E-01	15	6.1200E-02	White et al. 2006
4930	Ecto.verts	Acris crepitans	1.2700E+00	15	8.3800E-02	White et al. 2006
4931	Ecto.verts	Acris crepitans	1.5000E+00	15	1.1550E-01	White et al. 2006
4932	Ecto.verts	Acris crepitans	1.5000E+00	25	2.5350E-01	White et al. 2006
4933	Ecto.verts	Acris crepitans	1.5900E+00	15	4.7700E-02	White et al. 2006
4934	Ecto.verts	Acris crepitans	1.7350E+00	15	1.1180E-01	White et al. 2006
4935	Ecto.verts	Acris crepitans	1.8550E+00	25	3.2880E-01	White et al. 2006
4936	Ecto.verts	Acris crepitans	1.9350E+00	5	4.5900E-02	White et al. 2006
4937	Ecto.verts	Acrochordus aradurae	1.0477E+03	30	2.4621E+01	White et al. 2006
4938	Ecto.verts	Acrochordus aradurae	1.0477E+03	27	2.8288E+01	White et al. 2006
4939	Ecto.verts	Agalychnis callidryas	5.6500E+00	20	3.3700E-01	White et al. 2006
4940	Ecto.verts	Agalychnis callidryas	5.7000E+00	20	3.4770E-01	White et al. 2006
4941	Ecto.verts	Alcolapia grahami	4.7000E+01	37	1.3156E+01	FishBase
4942	Ecto.verts	Alligator mississippiensis	1.2870E+03	10	7.2072E+00	White et al. 2006
4943	Ecto.verts	Alligator mississippiensis	1.2870E+03	15	1.3449E+01	White et al. 2006
4944	Ecto.verts	Alligator mississippiensis	1.2870E+03	20	2.7091E+01	White et al. 2006
4945	Ecto.verts	Alligator mississippiensis	1.2870E+03	25	4.7169E+01	White et al. 2006
4946	Ecto.verts	Alligator mississippiensis	1.2870E+03	30	5.8365E+01	White et al. 2006
4947	Ecto.verts	Alligator mississippiensis	1.2870E+03	35	1.0373E+02	White et al. 2006
4948	Ecto.verts	Amblyrhynchus cristatus	4.8900E+02	30	2.8362E+01	White et al. 2006
4949	Ecto.verts	Amblyrhynchus cristatus	4.8900E+02	35	4.8900E+01	White et al. 2006
4950	Ecto.verts	Amblyrhynchus cristatus	4.8900E+02	40	8.8020E+01	White et al. 2006

4951	Ecto.verts	Amblyrhynchus cristatus	7.4750E+02	25	2.7284E+01	White et al. 2006
4952	Ecto.verts	Ambystoma gracile	3.0390E+01	15	6.3520E-01	White et al. 2006
4953	Ecto.verts	Ambystoma jeffersonianum	8.9200E+00	15	3.1580E-01	White et al. 2006
4954	Ecto.verts	Ambystoma macrodactylum	3.4140E+00	15	1.0980E-01	White et al. 2006
4955	Ecto.verts	Ambystoma macrodactylum	3.7000E+00	15	4.5140E-01	White et al. 2006
4956	Ecto.verts	Ambystoma maculatum	5.1200E-01	20	5.1800E-02	White et al. 2006
4957	Ecto.verts	Ambystoma maculatum	1.1750E+01	5	3.6310E-01	White et al. 2006
4958	Ecto.verts	Ambystoma maculatum	1.1750E+01	10	5.8750E-01	White et al. 2006
4959	Ecto.verts	Ambystoma maculatum	1.1750E+01	15	1.2396E+00	White et al. 2006
4960	Ecto.verts	Ambystoma maculatum	1.1750E+01	25	1.5440E+00	White et al. 2006
4961	Ecto.verts	Ambystoma maculatum	1.1750E+01	30	1.5592E+00	White et al. 2006
4962	Ecto.verts	Ambystoma maculatum	1.1900E+01	10	3.0450E-01	White et al. 2006
4963	Ecto.verts	Ambystoma maculatum	1.2900E+01	25	1.6770E+00	White et al. 2006
4964	Ecto.verts	Ambystoma maculatum	1.2900E+01	30	1.7183E+00	White et al. 2006
4965	Ecto.verts	Ambystoma maculatum	1.3100E+01	5	3.1570E-01	White et al. 2006
4966	Ecto.verts	Ambystoma maculatum	1.3400E+01	15	1.3199E+00	White et al. 2006
4967	Ecto.verts	Ambystoma maculatum	1.5830E+01	15	3.6250E-01	White et al. 2006
4968	Ecto.verts	Ambystoma mexicanum	2.1300E+01	22	3.1950E-01	White et al. 2006
4969	Ecto.verts	Ambystoma mexicanum	4.3700E+01	20	9.6140E-01	White et al. 2006
4970	Ecto.verts	Ambystoma opacum	4.6500E+00	14	8.6500E-02	White et al. 2006
4971	Ecto.verts	Ambystoma opacum	5.8000E+00	15	4.6400E-01	White et al. 2006
4972	Ecto.verts	Ambystoma talpoideum	7.0000E+00	15	5.6000E-01	White et al. 2006
4973	Ecto.verts	Ambystoma tigrinum	8.1000E+00	25	8.9100E-01	White et al. 2006
4974	Ecto.verts	Ambystoma tigrinum	1.0780E+01	15	1.3800E-01	White et al. 2006
4975	Ecto.verts	Ambystoma tigrinum	1.4900E+01	25	1.5675E+00	White et al. 2006
4976	Ecto.verts	Ambystoma tigrinum	1.5000E+01	10	8.8950E-01	White et al. 2006

4977	Ecto.verts	Ambystoma tigrinum	1.6490E+01	6	8.5950E-01	White et al. 2006
4978	Ecto.verts	Ambystoma tigrinum	1.6490E+01	15	1.3213E+00	White et al. 2006
4979	Ecto.verts	Ambystoma tigrinum	1.6490E+01	25	3.1908E+00	White et al. 2006
4980	Ecto.verts	Ambystoma tigrinum	1.6500E+01	15	1.5246E+00	White et al. 2006
4981	Ecto.verts	Ambystoma tigrinum	2.3000E+01	5	2.7600E-01	White et al. 2006
4982	Ecto.verts	Ambystoma tigrinum	2.3000E+01	25	1.9780E+00	White et al. 2006
4983	Ecto.verts	Ambystoma tigrinum	2.9400E+01	23	2.9988E+00	White et al. 2006
4984	Ecto.verts	Ambystoma tigrinum	3.3800E+01	23	2.6026E+00	White et al. 2006
4985	Ecto.verts	Ambystoma tigrinum	3.5000E+01	15	1.5484E+00	White et al. 2006
4986	Ecto.verts	Ambystoma tigrinum	3.5000E+01	25	1.7885E+00	White et al. 2006
4987	Ecto.verts	Ameiurus nebulosus	4.7000E+01	10	1.1183E+00	FishBase
4988	Ecto.verts	Ameiurus nebulosus	7.4900E+01	25	4.8745E+00	FishBase
4989	Ecto.verts	Ameiurus nebulosus	7.4900E+01	25	5.1890E+00	FishBase
4990	Ecto.verts	Ameiurus nebulosus	7.4900E+01	25	5.2414E+00	FishBase
4991	Ecto.verts	Ameiurus nebulosus	7.4900E+01	25	5.2938E+00	FishBase
4992	Ecto.verts	Ameiurus nebulosus	1.1500E+02	30	8.5304E+00	FishBase
4993	Ecto.verts	Ameiurus nebulosus	1.1600E+02	10	1.6235E+00	FishBase
4994	Ecto.verts	Ameiurus nebulosus	1.4700E+02	20	6.7894E+00	FishBase
4995	Ecto.verts	Amphibolurus barbatus	3.7300E+02	20	1.6375E+01	White et al. 2006
4996	Ecto.verts	Amphibolurus barbatus	3.7300E+02	30	3.6517E+01	White et al. 2006
4997	Ecto.verts	Amphibolurus barbatus	3.7300E+02	37	5.2220E+01	White et al. 2006
4998	Ecto.verts	Amphibolurus nuchalis	2.4650E+01	40	5.9407E+00	White et al. 2006
4999	Ecto.verts	Amphibolurus nuchalis	3.4300E+01	37	6.8600E+00	White et al. 2006
5000	Ecto.verts	Amphiuma means	8.3000E+01	20	2.3240E+00	White et al. 2006
5001	Ecto.verts	Amphiuma means	1.2500E+02	18	4.8750E+00	White et al. 2006
5002	Ecto.verts	Amphiuma means	3.5200E+02	5	8.8400E-01	White et al. 2006

5003	Ecto.verts	Amphiuma means	3.7500E+02	15	2.5310E+00	White et al. 2006
5004	Ecto.verts	Amphiuma means	3.7600E+02	25	5.0380E+00	White et al. 2006
5005	Ecto.verts	Amphiuma means	4.0800E+02	15	2.5130E+00	White et al. 2006
5006	Ecto.verts	Amphiuma means	5.0000E+02	5	1.6500E+00	White et al. 2006
5007	Ecto.verts	Amphiuma means	5.0000E+02	10	2.8000E+00	White et al. 2006
5008	Ecto.verts	Amphiuma means	5.0000E+02	15	3.1000E+00	White et al. 2006
5009	Ecto.verts	Amphiuma means	5.0000E+02	20	5.0500E+00	White et al. 2006
5010	Ecto.verts	Amphiuma means	5.0000E+02	25	9.2500E+00	White et al. 2006
5011	Ecto.verts	Amphiuma means	5.0000E+02	27	1.5800E+01	White et al. 2006
5012	Ecto.verts	Amphiuma means	5.0000E+02	30	3.5300E+01	White et al. 2006
5013	Ecto.verts	Amphiuma means	1.2700E+03	21.5	1.7304E+01	White et al. 2006
5014	Ecto.verts	Amphiuma tridactylum	4.9300E+02	25	9.8600E+00	White et al. 2006
5015	Ecto.verts	Amphiuma tridactylum	6.5200E+02	15	2.4520E+00	White et al. 2006
5016	Ecto.verts	Anarbylus switaki	9.4800E+00	25	6.9962E-01	White et al. 2006
5017	Ecto.verts	Aneides ferreus	2.7300E+00	15	7.4300E-02	White et al. 2006
5018	Ecto.verts	Aneides ferreus	3.2000E+00	25	2.1180E-01	White et al. 2006
5019	Ecto.verts	Aneides flavipunctatus	1.7900E+00	25	1.4210E-01	White et al. 2006
5020	Ecto.verts	Aneides flavipunctatus	4.5700E+00	15	1.1970E-01	White et al. 2006
5021	Ecto.verts	Aneides hardii	9.7000E-01	5	3.0100E-02	White et al. 2006
5022	Ecto.verts	Aneides hardii	9.7000E-01	10	3.5900E-02	White et al. 2006
5023	Ecto.verts	Aneides hardii	9.7000E-01	15	4.8500E-02	White et al. 2006
5024	Ecto.verts	Aneides hardii	9.7000E-01	20	5.1400E-02	White et al. 2006
5025	Ecto.verts	Aneides hardii	9.7000E-01	25	9.1200E-02	White et al. 2006
5026	Ecto.verts	Aneides lugubris	1.1600E-01	20	4.4700E-02	White et al. 2006
5027	Ecto.verts	Aneides lugubris	5.5800E+00	15	1.4950E-01	White et al. 2006
5028	Ecto.verts	Anguilla anguilla	4.7000E+01	18	3.9468E-01	FishBase

5029	Ecto.verts	Anguilla anguilla	4.7000E+01	20	2.9272E+00	FishBase
5030	Ecto.verts	Anguilla anguilla	4.7000E+01	24	4.4402E+00	FishBase
5031	Ecto.verts	Anguilla anguilla	4.7000E+01	27	5.5255E+00	FishBase
5032	Ecto.verts	Anguilla anguilla	8.0000E+01	25	2.8551E+00	FishBase
5033	Ecto.verts	Anguilla anguilla	8.0000E+01	25	3.1910E+00	FishBase
5034	Ecto.verts	Anguilla anguilla	8.0000E+01	25	3.1910E+00	FishBase
5035	Ecto.verts	Anguilla anguilla	8.0000E+01	25	3.6389E+00	FishBase
5036	Ecto.verts	Anguilla anguilla	8.0000E+01	25	3.8628E+00	FishBase
5037	Ecto.verts	Anguilla anguilla	8.0000E+01	25	4.0308E+00	FishBase
5038	Ecto.verts	Anguilla anguilla	8.0000E+01	25	4.0868E+00	FishBase
5039	Ecto.verts	Anguilla anguilla	8.0000E+01	25	4.1428E+00	FishBase
5040	Ecto.verts	Anguilla anguilla	8.0000E+01	25	4.1987E+00	FishBase
5041	Ecto.verts	Anguilla anguilla	8.0000E+01	25	4.2547E+00	FishBase
5042	Ecto.verts	Anguilla anguilla	8.0000E+01	25	4.3107E+00	FishBase
5043	Ecto.verts	Anguilla anguilla	8.0000E+01	25	4.7586E+00	FishBase
5044	Ecto.verts	Anguilla anguilla	8.0000E+01	25	4.8146E+00	FishBase
5045	Ecto.verts	Anguilla anguilla	8.0000E+01	25	4.9825E+00	FishBase
5046	Ecto.verts	Anguilla anguilla	8.0000E+01	25	5.0945E+00	FishBase
5047	Ecto.verts	Anguilla anguilla	8.0000E+01	25	5.6543E+00	FishBase
5048	Ecto.verts	Anguilla anguilla	8.0000E+01	25	3.6389E+00	FishBase
5049	Ecto.verts	Anguilla australis australis	7.0000E+02	20	1.0777E+01	FishBase
5050	Ecto.verts	Anguilla japonica	3.2500E+02	14.5	4.5486E+00	FishBase
5051	Ecto.verts	Anguilla japonica	3.2500E+02	13	4.7761E+00	FishBase
5052	Ecto.verts	Anguilla japonica	3.2500E+02	14.5	5.0035E+00	FishBase
5053	Ecto.verts	Anguilla japonica	3.2500E+02	15	5.9132E+00	FishBase
5054	Ecto.verts	Anguilla japonica	3.2500E+02	14.5	6.5955E+00	FishBase

5055	Ecto.verts	Anguilla japonica	3.2500E+02	15	7.0504E+00	FishBase
5056	Ecto.verts	Anguilla japonica	3.2500E+02	18.5	7.0504E+00	FishBase
5057	Ecto.verts	Anguilla japonica	3.2500E+02	19	7.5052E+00	FishBase
5058	Ecto.verts	Anguilla japonica	3.2500E+02	17	8.1875E+00	FishBase
5059	Ecto.verts	Anguilla japonica	3.2500E+02	16	8.1875E+00	FishBase
5060	Ecto.verts	Anguilla japonica	3.2500E+02	18	8.4150E+00	FishBase
5061	Ecto.verts	Anguilla japonica	3.2500E+02	20	8.4150E+00	FishBase
5062	Ecto.verts	Anguilla japonica	3.2500E+02	18	9.3247E+00	FishBase
5063	Ecto.verts	Anguilla japonica	3.2500E+02	19.5	9.3247E+00	FishBase
5064	Ecto.verts	Anguilla japonica	3.2500E+02	19.5	9.5521E+00	FishBase
5065	Ecto.verts	Anguilla japonica	3.2500E+02	19	9.5521E+00	FishBase
5066	Ecto.verts	Anguilla japonica	3.2500E+02	17	9.5521E+00	FishBase
5067	Ecto.verts	Anguilla japonica	3.2500E+02	20	1.0007E+01	FishBase
5068	Ecto.verts	Anguilla japonica	3.2500E+02	20	1.0234E+01	FishBase
5069	Ecto.verts	Anguilla japonica	3.2500E+02	19	1.1826E+01	FishBase
5070	Ecto.verts	Anguilla japonica	3.2500E+02	20	1.3191E+01	FishBase
5071	Ecto.verts	Anguilla japonica	3.2500E+02	20	1.3646E+01	FishBase
5072	Ecto.verts	Anguilla japonica	3.2500E+02	25	1.6830E+01	FishBase
5073	Ecto.verts	Anguilla japonica	3.2500E+02	24	1.8877E+01	FishBase
5074	Ecto.verts	Anguilla japonica	3.2500E+02	29	2.4563E+01	FishBase
5075	Ecto.verts	Anguilla japonica	3.2500E+02	26	2.6155E+01	FishBase
5076	Ecto.verts	Anguilla japonica	3.2500E+02	27.5	2.6610E+01	FishBase
5077	Ecto.verts	Anguilla japonica	3.2500E+02	30	2.8656E+01	FishBase
5078	Ecto.verts	Anguilla japonica	3.2500E+02	29	3.1158E+01	FishBase
5079	Ecto.verts	Anguilla rostrata	4.7000E+01	15	9.5381E-01	FishBase
5080	Ecto.verts	Anguilla rostrata	4.7000E+01	20	1.6445E+00	FishBase

5081	Ecto.verts	Anguilla rostrata	4.7000E+01	23	2.4010E+00	FishBase
5082	Ecto.verts	Anguilla rostrata	4.7000E+01	25	3.4535E+00	FishBase
5083	Ecto.verts	Anguilla rostrata	2.1400E+02	15	4.1931E+00	FishBase
5084	Ecto.verts	Anguilla rostrata	3.1500E+02	15	4.6291E+00	FishBase
5085	Ecto.verts	Anguis fragilis	1.2067E+01	30	7.1075E-01	White et al. 2006
5086	Ecto.verts	Anguis fragilis	1.3300E+01	20	1.4630E+00	White et al. 2006
5087	Ecto.verts	Anguis fragilis	1.3300E+01	35	2.9659E+00	White et al. 2006
5088	Ecto.verts	Anniella pulchra	4.5000E+00	20	3.4088E-01	White et al. 2006
5089	Ecto.verts	Anniella pulchra	4.8466E+00	25	3.1648E-01	White et al. 2006
5090	Ecto.verts	Anniella pulchra	4.9000E+00	6	1.3720E-01	White et al. 2006
5091	Ecto.verts	Anniella pulchra	4.9000E+00	30	5.6840E-01	White et al. 2006
5092	Ecto.verts	Anniella pulchra	5.2000E+00	13	1.5080E-01	White et al. 2006
5093	Ecto.verts	Anolis acutus	4.3000E+00	25	8.1958E-01	White et al. 2006
5094	Ecto.verts	Anolis acutus	4.3000E+00	30	1.2470E+00	White et al. 2006
5095	Ecto.verts	Anolis bonariensis	1.2000E+01	27	7.8000E-01	White et al. 2006
5096	Ecto.verts	Anolis bonariensis	1.2000E+01	33	1.6920E+00	White et al. 2006
5097	Ecto.verts	Anolis carolinensis	4.5000E+00	20	4.9500E-01	White et al. 2006
5098	Ecto.verts	Anolis carolinensis	4.5000E+00	30	8.4600E-01	White et al. 2006
5099	Ecto.verts	Anolis limifrons	1.4000E+00	30	3.3880E-01	White et al. 2006
5100	Ecto.verts	Anolis limifrons	1.5000E+00	20	1.2000E-01	White et al. 2006
5101	Ecto.verts	Antaresia childreni	3.3170E+02	24	1.1610E+01	White et al. 2006
5102	Ecto.verts	Antaresia childreni	3.3170E+02	30	2.4214E+01	White et al. 2006
5103	Ecto.verts	Antaresia childreni	3.7250E+02	33	3.3898E+01	White et al. 2006
5104	Ecto.verts	Antaresia childreni	3.7260E+02	27	1.7885E+01	White et al. 2006
5105	Ecto.verts	Antaresia stimsoni	3.4990E+02	30	1.9944E+01	White et al. 2006
5106	Ecto.verts	Antaresia stimsoni	3.6070E+02	27	1.8396E+01	White et al. 2006

5107	Ecto.verts	Antaresia stimsoni	3.6070E+02	24	2.1281E+01	White et al. 2006
5108	Ecto.verts	Antaresia stimsoni	3.7180E+02	33	2.9372E+01	White et al. 2006
5109	Ecto.verts	Arapaima gigas	2.3000E+03	28	4.0238E+01	FishBase
5110	Ecto.verts	Arapaima gigas	2.3000E+03	28	4.5066E+01	FishBase
5111	Ecto.verts	Arapaima gigas	2.3000E+03	28	4.6676E+01	FishBase
5112	Ecto.verts	Arnoglossus thori	4.7000E+01	11	1.6774E+00	FishBase
5113	Ecto.verts	Arnoglossus thori	4.7000E+01	19	1.8090E+00	FishBase
5114	Ecto.verts	Artediellus atlanticus	4.7000E+01	0	4.6046E-01	FishBase
5115	Ecto.verts	Artediellus uncinatus	4.7000E+01	0	3.6179E-01	FishBase
5116	Ecto.verts	Artediellus uncinatus	4.7000E+01	-1.5	9.8670E-01	FishBase
5117	Ecto.verts	Aspidites melanocephalus	1.0275E+03	24	3.7709E+01	White et al. 2006
5118	Ecto.verts	Aspidites melanocephalus	1.0275E+03	27	5.5485E+01	White et al. 2006
5119	Ecto.verts	Aspidites melanocephalus	1.0275E+03	30	7.5008E+01	White et al. 2006
5120	Ecto.verts	Aspidites melanocephalus	1.0275E+03	33	1.0378E+02	White et al. 2006
5121	Ecto.verts	Balistes capriscus	3.2000E+02	17.5	1.8810E+01	FishBase
5122	Ecto.verts	Balistes capriscus	3.9500E+02	17.9	2.5983E+01	FishBase
5123	Ecto.verts	Balistes capriscus	7.0000E+02	17.9	5.2414E+01	FishBase
5124	Ecto.verts	Bathylagus antarcticus	4.7000E+01	0.5	6.5780E-01	FishBase
5125	Ecto.verts	Batrachoseps attenuatus	6.5000E-01	5	1.6600E-02	White et al. 2006
5126	Ecto.verts	Batrachoseps attenuatus	7.4000E-01	25	8.3800E-02	White et al. 2006
5127	Ecto.verts	Batrachoseps attenuatus	7.7000E-01	15	3.1800E-02	White et al. 2006
5128	Ecto.verts	Batrachoseps attenuatus	9.3000E-01	15	4.5300E-02	White et al. 2006
5129	Ecto.verts	Batrachoseps attenuatus	9.3000E-01	25	8.0200E-02	White et al. 2006
5130	Ecto.verts	Batrachoseps attenuatus	9.3100E-01	20	6.9200E-02	White et al. 2006
5131	Ecto.verts	Batrachoseps attenuatus	1.5500E+00	20	9.3000E-02	White et al. 2006
5132	Ecto.verts	Blanus cinereus	2.4000E+00	20	2.8800E-01	White et al. 2006

5133	Ecto.verts	Blanus cinereus	2.4000E+00	30	4.2240E-01	White et al. 2006
5134	Ecto.verts	Blanus cinereus	2.4000E+00	35	7.9440E-01	White et al. 2006
5135	Ecto.verts	Boa constrictor	5.4063E+03	20	5.7036E+01	White et al. 2006
5136	Ecto.verts	Boa constrictor	7.8155E+03	30	1.2603E+02	White et al. 2006
5137	Ecto.verts	Boa constrictor	7.8155E+03	34	1.9657E+02	White et al. 2006
5138	Ecto.verts	Bolitoglossa franklini	3.1800E+00	15	5.5000E-02	White et al. 2006
5139	Ecto.verts	Bolitoglossa morio	2.0900E+00	15	4.4500E-02	White et al. 2006
5140	Ecto.verts	Bolitoglossa occidentalis	6.1000E-01	15	1.6800E-02	White et al. 2006
5141	Ecto.verts	Bolitoglossa occidentalis	6.1000E-01	25	6.7300E-02	White et al. 2006
5142	Ecto.verts	Bolitoglossa occidentalis	9.7000E-01	15	2.0200E-02	White et al. 2006
5143	Ecto.verts	Bolitoglossa occidentalis	9.8000E-01	25	1.0390E-01	White et al. 2006
5144	Ecto.verts	Bolitoglossa occidentalis	1.0700E+00	5	1.9600E-02	White et al. 2006
5145	Ecto.verts	Bolitoglossa subpalmata	1.6300E+00	5	1.5900E-02	White et al. 2006
5146	Ecto.verts	Bolitoglossa subpalmata	1.6300E+00	10	2.5100E-02	White et al. 2006
5147	Ecto.verts	Bolitoglossa subpalmata	1.6300E+00	15	5.1300E-02	White et al. 2006
5148	Ecto.verts	Bolitoglossa subpalmata	1.6300E+00	20	7.0800E-02	White et al. 2006
5149	Ecto.verts	Bombina orientalis	2.6000E+00	20	1.4560E-01	White et al. 2006
5150	Ecto.verts	Bombina orientalis	2.6200E+00	20	1.4900E-01	White et al. 2006
5151	Ecto.verts	Boreogadus saida	4.7000E+01	-1.5	1.5458E+00	FishBase
5152	Ecto.verts	Boulengerula taitanus	5.0000E+00	20	3.1750E-01	White et al. 2006
5153	Ecto.verts	Boulengerula taitanus	5.0000E+00	25	4.2050E-01	White et al. 2006
5154	Ecto.verts	Boulengerula taitanus	5.0000E+00	35	8.6000E-01	White et al. 2006
5155	Ecto.verts	Bufo alvaris	1.5080E+02	15	1.1868E+01	White et al. 2006
5156	Ecto.verts	Bufo americanus	1.0200E-01	23	2.4700E-02	White et al. 2006
5157	Ecto.verts	Bufo americanus	1.1000E+01	21.5	1.2815E+00	White et al. 2006
5158	Ecto.verts	Bufo americanus	1.9000E+01	15	2.0934E+00	White et al. 2006

5159	Ecto.verts	Bufo americanus	2.1100E+01	25	2.5873E+00	White et al. 2006
5160	Ecto.verts	Bufo americanus	2.5200E+01	5	6.0810E-01	White et al. 2006
5161	Ecto.verts	Bufo americanus	2.7000E+01	20	1.3770E+00	White et al. 2006
5162	Ecto.verts	Bufo americanus	2.7000E+01	20	1.3770E+00	White et al. 2006
5163	Ecto.verts	Bufo americanus	2.7030E+01	20	1.3800E+00	White et al. 2006
5164	Ecto.verts	Bufo americanus	4.0400E+01	25	2.9371E+00	White et al. 2006
5165	Ecto.verts	Bufo americanus	5.0000E+01	20	1.3900E+00	White et al. 2006
5166	Ecto.verts	Bufo boreas	2.5000E+00	20	2.5000E-01	White et al. 2006
5167	Ecto.verts	Bufo boreas	2.7300E+01	25	2.6400E+00	White et al. 2006
5168	Ecto.verts	Bufo boreas	2.8700E+01	15	3.8706E+00	White et al. 2006
5169	Ecto.verts	Bufo boreas	2.9500E+01	14	4.4840E+00	White et al. 2006
5170	Ecto.verts	Bufo boreas	4.0200E+01	10	5.5480E-01	White et al. 2006
5171	Ecto.verts	Bufo boreas	4.0200E+01	20	1.3025E+00	White et al. 2006
5172	Ecto.verts	Bufo boreas	4.0200E+01	30	3.0512E+00	White et al. 2006
5173	Ecto.verts	Bufo boreas	4.6400E+01	14	4.9648E+00	White et al. 2006
5174	Ecto.verts	Bufo boreas	4.7300E+01	10	6.2440E-01	White et al. 2006
5175	Ecto.verts	Bufo boreas	4.7300E+01	20	2.3177E+00	White et al. 2006
5176	Ecto.verts	Bufo boreas	4.7300E+01	30	2.4265E+00	White et al. 2006
5177	Ecto.verts	Bufo boreas	5.3800E+01	25	5.5812E+00	White et al. 2006
5178	Ecto.verts	Bufo boreas	5.7800E+01	10	7.2830E-01	White et al. 2006
5179	Ecto.verts	Bufo boreas	5.7800E+01	20	1.0520E+00	White et al. 2006
5180	Ecto.verts	Bufo boreas	5.7800E+01	30	6.4852E+00	White et al. 2006
5181	Ecto.verts	Bufo boreas	7.2700E+01	5	4.0908E+00	White et al. 2006
5182	Ecto.verts	Bufo bufo	2.8590E+01	6	2.0370E+00	White et al. 2006
5183	Ecto.verts	Bufo bufo	2.8590E+01	25	4.5851E+00	White et al. 2006
5184	Ecto.verts	Bufo bufo	2.8590E+01	15	4.8389E+00	White et al. 2006

5185	Ecto.verts	Bufo bufo	2.9000E+01	20	3.9150E+00	White et al. 2006
5186	Ecto.verts	Bufo bufo	2.9000E+01	20	4.9010E+00	White et al. 2006
5187	Ecto.verts	Bufo bufo	4.3000E+01	17	4.8246E+00	White et al. 2006
5188	Ecto.verts	Bufo bufo	7.6130E+01	6	1.7415E+00	White et al. 2006
5189	Ecto.verts	Bufo bufo	7.6130E+01	15	2.8549E+00	White et al. 2006
5190	Ecto.verts	Bufo bufo	7.6130E+01	25	9.6400E+00	White et al. 2006
5191	Ecto.verts	Bufo calamita	8.6700E+00	20	5.1100E-01	White et al. 2006
5192	Ecto.verts	Bufo calamita	8.7000E+00	20	5.0460E-01	White et al. 2006
5193	Ecto.verts	Bufo cognatus	3.9580E+01	10	1.9790E+00	White et al. 2006
5194	Ecto.verts	Bufo cognatus	3.9580E+01	20	4.4725E+00	White et al. 2006
5195	Ecto.verts	Bufo cognatus	3.9580E+01	30	7.2036E+00	White et al. 2006
5196	Ecto.verts	Bufo cognatus	4.0200E+01	25	2.9587E+00	White et al. 2006
5197	Ecto.verts	Bufo cognatus	4.5200E+01	5	1.2792E+00	White et al. 2006
5198	Ecto.verts	Bufo cognatus	5.0500E+01	15	3.8481E+00	White et al. 2006
5199	Ecto.verts	Bufo debilus	6.6000E-01	15	8.4500E-02	White et al. 2006
5200	Ecto.verts	Bufo marinus	8.8700E+01	25	6.3447E+00	White et al. 2006
5201	Ecto.verts	Bufo marinus	1.0100E+02	15	2.2877E+00	White et al. 2006
5202	Ecto.verts	Bufo marinus	1.0100E+02	20	2.6088E+00	White et al. 2006
5203	Ecto.verts	Bufo marinus	1.0100E+02	25	3.1704E+00	White et al. 2006
5204	Ecto.verts	Bufo marinus	1.0100E+02	30	5.7782E+00	White et al. 2006
5205	Ecto.verts	Bufo marinus	1.0100E+02	35	8.2669E+00	White et al. 2006
5206	Ecto.verts	Bufo marinus	1.2380E+02	15	4.6276E+00	White et al. 2006
5207	Ecto.verts	Bufo marinus	1.4500E+02	15	1.5515E+00	White et al. 2006
5208	Ecto.verts	Bufo marinus	1.5250E+02	22	7.3200E+00	White et al. 2006
5209	Ecto.verts	Bufo marinus	1.5600E+02	20	3.0420E+00	White et al. 2006
5210	Ecto.verts	Bufo marinus	2.5200E+02	22	1.1088E+01	White et al. 2006

5211	Ecto.verts	Bufo marinus	1.2000E+03	18.5	3.3150E+01	White et al. 2006
5212	Ecto.verts	Bufo terrestris	1.6600E+01	15	1.3360E+00	White et al. 2006
5213	Ecto.verts	Bufo terrestris	1.9800E+01	25	2.2425E+00	White et al. 2006
5214	Ecto.verts	Bufo terrestris	2.1400E+01	5	6.5270E-01	White et al. 2006
5215	Ecto.verts	Bufo terrestris	1.0200E+02	15	7.5000E+00	White et al. 2006
5216	Ecto.verts	Bufo terrestris	1.0200E+02	20	1.5100E+01	White et al. 2006
5217	Ecto.verts	Bufo terrestris	1.0200E+02	25	3.0420E+01	White et al. 2006
5218	Ecto.verts	Bufo terrestris	1.0200E+02	30	6.1250E+01	White et al. 2006
5219	Ecto.verts	Bufo viridis	3.5000E+01	20	8.7500E-01	White et al. 2006
5220	Ecto.verts	Bufo woodhousii	9.0000E+00	25	1.0170E+00	White et al. 2006
5221	Ecto.verts	Bufo woodhousii	5.2200E+01	23	2.1600E+00	White et al. 2006
5222	Ecto.verts	Bufo woodhousii	5.6300E+01	25	2.2351E+00	White et al. 2006
5223	Ecto.verts	Bufo woodhousii	6.4800E+01	20	4.4129E+00	White et al. 2006
5224	Ecto.verts	Bufo woodhousii	6.7400E+01	10	1.2739E+00	White et al. 2006
5225	Ecto.verts	Bufo woodhousii	6.9000E+01	15	2.7255E+00	White et al. 2006
5226	Ecto.verts	Buglossidium luteum	4.7000E+01	11	8.5514E-01	FishBase
5227	Ecto.verts	Buglossidium luteum	4.7000E+01	19	1.7103E+00	FishBase
5228	Ecto.verts	Bunopus tuberculatus	2.5000E+00	20	1.1750E+00	White et al. 2006
5229	Ecto.verts	Bunopus tuberculatus	2.5000E+00	30	1.8700E+00	White et al. 2006
5230	Ecto.verts	Bunopus tuberculatus	2.5000E+00	35	2.5575E+00	White et al. 2006
5231	Ecto.verts	Callionymus lyra	4.8700E+01	11	1.8062E+00	FishBase
5232	Ecto.verts	Callionymus lyra	8.5700E+01	11	3.2385E+00	FishBase
5233	Ecto.verts	Callionymus lyra	1.0500E+02	11.5	1.5430E+00	FishBase
5234	Ecto.verts	Callionymus lyra	1.0500E+02	11.5	1.9839E+00	FishBase
5235	Ecto.verts	Callionymus lyra	1.0500E+02	11.5	2.4248E+00	FishBase
5236	Ecto.verts	Callionymus lyra	1.0500E+02	11.5	2.6452E+00	FishBase

5237	Ecto.verts	Callionymus lyra	1.0500E+02	11.5	2.7922E+00	FishBase
5238	Ecto.verts	Callionymus lyra	1.0500E+02	11.5	3.0861E+00	FishBase
5239	Ecto.verts	Callionymus lyra	1.0500E+02	11.5	3.7474E+00	FishBase
5240	Ecto.verts	Callionymus lyra	1.0500E+02	11.5	4.0413E+00	FishBase
5241	Ecto.verts	Callionymus lyra	1.0500E+02	11.5	4.3352E+00	FishBase
5242	Ecto.verts	Callionymus lyra	1.0500E+02	11.5	4.9230E+00	FishBase
5243	Ecto.verts	Callionymus lyra	1.0500E+02	11.5	5.2169E+00	FishBase
5244	Ecto.verts	Callionymus lyra	1.0500E+02	11.5	5.5108E+00	FishBase
5245	Ecto.verts	Callionymus lyra	1.0500E+02	11.5	5.5843E+00	FishBase
5246	Ecto.verts	Callionymus lyra	1.0500E+02	11.5	5.9517E+00	FishBase
5247	Ecto.verts	Callionymus lyra	1.0500E+02	11.5	6.3926E+00	FishBase
5248	Ecto.verts	Callionymus lyra	1.0500E+02	11.5	6.4661E+00	FishBase
5249	Ecto.verts	Callionymus lyra	1.0500E+02	11.5	6.5395E+00	FishBase
5250	Ecto.verts	Callionymus lyra	1.0500E+02	11.5	6.6865E+00	FishBase
5251	Ecto.verts	Callionymus lyra	1.0500E+02	11.5	6.8334E+00	FishBase
5252	Ecto.verts	Callionymus lyra	1.0500E+02	11.5	6.9069E+00	FishBase
5253	Ecto.verts	Callionymus lyra	1.0500E+02	11.5	7.0539E+00	FishBase
5254	Ecto.verts	Candoia carinatus	5.2190E+02	20	3.8313E+00	White et al. 2006
5255	Ecto.verts	Candoia carinatus	5.2190E+02	30	1.0411E+01	White et al. 2006
5256	Ecto.verts	Candoia carinatus	5.2190E+02	34	1.6336E+01	White et al. 2006
5257	Ecto.verts	Carassius auratus	1.1000E+01	20	8.8523E-01	FishBase
5258	Ecto.verts	Carassius auratus	1.1300E+01	20	9.0938E-01	FishBase
5259	Ecto.verts	Carassius auratus	3.2000E+01	24	2.3961E+00	FishBase
5260	Ecto.verts	Carassius auratus	4.7000E+01	10	5.5913E-01	FishBase
5261	Ecto.verts	Carassius auratus	4.7000E+01	30	2.9272E+00	FishBase
5262	Ecto.verts	Carassius auratus	4.7000E+01	35	4.5388E+00	FishBase

5263	Ecto.verts	Carassius auratus	4.7000E+01	20	1.0558E+01	FishBase
5264	Ecto.verts	Carassius auratus	5.2000E+01	12	2.9475E+00	FishBase
5265	Ecto.verts	Carassius auratus	5.7000E+01	22	6.3821E+00	FishBase
5266	Ecto.verts	Carassius auratus	5.7500E+01	30	6.7600E+00	FishBase
5267	Ecto.verts	Carassius auratus	6.7000E+01	30	3.3758E+00	FishBase
5268	Ecto.verts	Carassius auratus	7.6000E+01	35	6.7544E+00	FishBase
5269	Ecto.verts	Carassius auratus	7.7000E+01	10	8.6214E-01	FishBase
5270	Ecto.verts	Carassius auratus	7.7000E+01	32	1.4118E+01	FishBase
5271	Ecto.verts	Carassius auratus	8.0000E+01	20	2.7992E+00	FishBase
5272	Ecto.verts	Carassius auratus	9.0000E+01	20	1.8894E+00	FishBase
5273	Ecto.verts	Carassius auratus	9.1000E+01	32	9.4885E+00	FishBase
5274	Ecto.verts	Carassius auratus	1.0000E+02	10	1.1197E+00	FishBase
5275	Ecto.verts	Carassius auratus	1.0000E+02	20	2.0994E+00	FishBase
5276	Ecto.verts	Carassius auratus	1.0000E+02	12	3.0091E+00	FishBase
5277	Ecto.verts	Carassius auratus	1.0000E+02	30	5.0385E+00	FishBase
5278	Ecto.verts	Carassius auratus	1.0000E+02	35	8.8873E+00	FishBase
5279	Ecto.verts	Carassius auratus	1.0000E+02	32	1.0427E+01	FishBase
5280	Ecto.verts	Carassius auratus	1.1000E+02	20	6.0812E+00	FishBase
5281	Ecto.verts	Carassius auratus	1.5750E+02	20	4.0780E+00	FishBase
5282	Ecto.verts	Carassius auratus	1.5750E+02	20	5.4006E+00	FishBase
5283	Ecto.verts	Carassius auratus	1.7800E+02	12	5.3562E+00	FishBase
5284	Ecto.verts	Catostomus commersonii	4.7000E+01	10	6.5780E-01	FishBase
5285	Ecto.verts	Catostomus commersonii	4.7000E+01	20	2.3352E+00	FishBase
5286	Ecto.verts	Catostomus commersonii	4.7000E+01	30	3.8481E+00	FishBase
5287	Ecto.verts	Catostomus commersonii	7.0000E+01	15	3.8698E+00	FishBase
5288	Ecto.verts	Catostomus commersonii	7.2000E+01	10	1.7635E+00	FishBase

5289	Ecto.verts	Catostomus commersonii	9.5000E+01	20	7.3128E+00	FishBase
5290	Ecto.verts	Catostomus commersonii	1.0000E+02	10	2.3793E+00	FishBase
5291	Ecto.verts	Catostomus commersonii	1.0000E+02	10	2.7992E+00	FishBase
5292	Ecto.verts	Catostomus commersonii	1.0000E+02	20	5.1784E+00	FishBase
5293	Ecto.verts	Catostomus commersonii	1.0000E+02	15	5.5983E+00	FishBase
5294	Ecto.verts	Catostomus commersonii	1.0000E+02	20	7.6977E+00	FishBase
5295	Ecto.verts	Ceratophrys calcarata	4.0434E+00	35	4.0434E+00	White et al. 2006
5296	Ecto.verts	Ceratophrys calcarata	5.5500E+01	25	2.9670E+00	White et al. 2006
5297	Ecto.verts	Chaenocephalus aceratus	4.7000E+01	1	1.8747E+00	FishBase
5298	Ecto.verts	Chaenocephalus aceratus	1.1300E+03	2	1.4234E+01	FishBase
5299	Ecto.verts	Chaenocephalus aceratus	1.1300E+03	2	1.9769E+01	FishBase
5300	Ecto.verts	Chaenocephalus aceratus	1.1300E+03	2	2.2141E+01	FishBase
5301	Ecto.verts	Chaenocephalus aceratus	1.1300E+03	6	2.4514E+01	FishBase
5302	Ecto.verts	Chaenocephalus aceratus	1.1300E+03	4	2.6886E+01	FishBase
5303	Ecto.verts	Chaenocephalus aceratus	1.1300E+03	10	2.8467E+01	FishBase
5304	Ecto.verts	Chaenocephalus aceratus	1.1300E+03	8	2.8467E+01	FishBase
5305	Ecto.verts	Chaenocephalus aceratus	1.1300E+03	8	3.0049E+01	FishBase
5306	Ecto.verts	Chaenocephalus aceratus	1.1300E+03	6	3.0049E+01	FishBase
5307	Ecto.verts	Chaenocephalus aceratus	1.1300E+03	10	3.0840E+01	FishBase
5308	Ecto.verts	Chaenocephalus aceratus	1.1300E+03	6	3.0840E+01	FishBase
5309	Ecto.verts	Chaenocephalus aceratus	1.1300E+03	4	3.1631E+01	FishBase
5310	Ecto.verts	Chaenocephalus aceratus	1.1300E+03	6	3.2421E+01	FishBase
5311	Ecto.verts	Chaenocephalus aceratus	1.1300E+03	4	3.4003E+01	FishBase
5312	Ecto.verts	Chaenocephalus aceratus	1.1300E+03	10	3.5584E+01	FishBase
5313	Ecto.verts	Chaenocephalus aceratus	1.1300E+03	4	3.7166E+01	FishBase
5314	Ecto.verts	Chaenocephalus aceratus	1.1300E+03	6	3.7957E+01	FishBase

5315	Ecto.verts	Chaenocephalus aceratus	1.1300E+03	4	3.7957E+01	FishBase
5316	Ecto.verts	Chaenocephalus aceratus	1.1300E+03	4	3.8747E+01	FishBase
5317	Ecto.verts	Chaenocephalus aceratus	1.1300E+03	6	5.0609E+01	FishBase
5318	Ecto.verts	Chaenocephalus aceratus	1.1300E+03	2	1.6606E+01	FishBase
5319	Ecto.verts	Chalcides ocellatus	2.2060E+01	5	4.4120E-01	White et al. 2006
5320	Ecto.verts	Chalcides ocellatus	2.2060E+01	10	5.7908E-01	White et al. 2006
5321	Ecto.verts	Chalcides ocellatus	2.2060E+01	15	7.5114E-01	White et al. 2006
5322	Ecto.verts	Chalcides ocellatus	2.2060E+01	20	1.0203E+00	White et al. 2006
5323	Ecto.verts	Chalcides ocellatus	2.2060E+01	25	1.5177E+00	White et al. 2006
5324	Ecto.verts	Chalcides ocellatus	2.2060E+01	30	2.0485E+00	White et al. 2006
5325	Ecto.verts	Chalcides ocellatus	2.2060E+01	33	2.0736E+00	White et al. 2006
5326	Ecto.verts	Chalcides ocellatus	2.2060E+01	35	3.1259E+00	White et al. 2006
5327	Ecto.verts	Channa punctata	8.3300E+00	20	4.4885E-01	FishBase
5328	Ecto.verts	Channa punctata	8.3300E+00	31	7.9861E-01	FishBase
5329	Ecto.verts	Channa punctata	1.2330E+01	20	5.5222E-01	FishBase
5330	Ecto.verts	Channa punctata	1.2660E+01	31	1.0100E+00	FishBase
5331	Ecto.verts	Channa punctata	2.3660E+01	31	1.4570E+00	FishBase
5332	Ecto.verts	Channa punctata	2.6660E+01	20	1.0074E+00	FishBase
5333	Ecto.verts	Channa punctata	3.3330E+01	20	1.1429E+00	FishBase
5334	Ecto.verts	Channa punctata	3.3660E+01	31	1.8608E+00	FishBase
5335	Ecto.verts	Channa punctata	4.3000E+01	31	2.0763E+00	FishBase
5336	Ecto.verts	Channa punctata	4.5660E+01	20	1.4698E+00	FishBase
5337	Ecto.verts	Channa punctata	5.3660E+01	31	2.1404E+00	FishBase
5338	Ecto.verts	Channa punctata	5.5000E+01	20	1.7320E+00	FishBase
5339	Ecto.verts	Channa punctata	6.2000E+01	20	1.9524E+00	FishBase
5340	Ecto.verts	Channa punctata	6.7000E+01	31	2.4850E+00	FishBase

5341	Ecto.verts	Channa punctata	7.5330E+01	31	2.7412E+00	FishBase
5342	Ecto.verts	Channa punctata	7.5660E+01	20	2.3826E+00	FishBase
5343	Ecto.verts	Channa punctata	8.1000E+01	31	2.9475E+00	FishBase
5344	Ecto.verts	Channa punctata	8.5660E+01	20	2.6375E+00	FishBase
5345	Ecto.verts	Channa punctata	9.3660E+01	20	2.8839E+00	FishBase
5346	Ecto.verts	Channa punctata	9.7000E+01	31	3.4619E+00	FishBase
5347	Ecto.verts	Channa punctata	1.0233E+02	20	3.1508E+00	FishBase
5348	Ecto.verts	Channa punctata	1.0500E+02	31	3.6739E+00	FishBase
5349	Ecto.verts	Channa punctata	1.1500E+02	31	4.0238E+00	FishBase
5350	Ecto.verts	Channa punctata	1.2566E+02	31	4.3968E+00	FishBase
5351	Ecto.verts	Channa punctata	1.3400E+02	31	4.6886E+00	FishBase
5352	Ecto.verts	Chelon labrosus	4.7000E+01	18	5.4598E+00	FishBase
5353	Ecto.verts	Chelon macrolepis	8.0000E+00	29	8.3975E-01	FishBase
5354	Ecto.verts	Chelydra serpentina	3.4730E+03	10	1.1635E+01	White et al. 2006
5355	Ecto.verts	Chelydra serpentina	3.4730E+03	20	3.9940E+01	White et al. 2006
5356	Ecto.verts	Chelydra serpentina	3.4730E+03	30	1.2503E+02	White et al. 2006
5357	Ecto.verts	Chiloscyllium plagiosum	8.8000E+02	23	2.8943E+01	FishBase
5358	Ecto.verts	Chiloscyllium plagiosum	8.8000E+02	23	3.5101E+01	FishBase
5359	Ecto.verts	Chiloscyllium plagiosum	8.8000E+02	23	3.8796E+01	FishBase
5360	Ecto.verts	Chiromantis petersi	9.0000E+00	25	7.2900E-01	White et al. 2006
5361	Ecto.verts	Chiromantis petersi	1.1200E+01	25	7.9520E-01	White et al. 2006
5362	Ecto.verts	Chironius quadricarinatus	6.1000E+01	20	2.0740E+00	White et al. 2006
5363	Ecto.verts	Chiropterotriton bromeliacia	5.9000E-01	15	1.4400E-02	White et al. 2006
5364	Ecto.verts	Cirrhinus cirrhosus	3.4000E+00	20	1.4514E-01	FishBase
5365	Ecto.verts	Cirrhinus cirrhosus	3.4000E+00	30	2.0462E-01	FishBase
5366	Ecto.verts	Cirrhinus cirrhosus	4.2400E+00	30.5	7.0617E-01	FishBase

5367	Ecto.verts	Cirrhinus cirrhosus	4.9400E+00	30.5	7.5016E-01	FishBase
5368	Ecto.verts	Cirrhinus cirrhosus	1.1300E+01	30.5	1.6132E+00	FishBase
5369	Ecto.verts	Cirrhinus cirrhosus	1.9780E+01	30.5	2.5469E+00	FishBase
5370	Ecto.verts	Cirrhinus cirrhosus	2.3320E+01	30.5	2.8722E+00	FishBase
5371	Ecto.verts	Cirrhinus cirrhosus	3.1800E+01	30.5	3.6941E+00	FishBase
5372	Ecto.verts	Cirrhinus cirrhosus	4.7000E+01	21.5	3.3877E+00	FishBase
5373	Ecto.verts	Cirrhinus cirrhosus	4.7000E+01	30.5	5.6900E+00	FishBase
5374	Ecto.verts	Cirrhinus cirrhosus	4.8760E+01	30.5	5.2889E+00	FishBase
5375	Ecto.verts	Cirrhinus cirrhosus	6.3600E+01	30.5	6.4980E+00	FishBase
5376	Ecto.verts	Cirrhinus cirrhosus	9.5000E+01	30.5	9.0413E+00	FishBase
5377	Ecto.verts	Cirrhinus cirrhosus	1.2200E+02	30.5	1.1099E+01	FishBase
5378	Ecto.verts	Cirrhinus cirrhosus	1.5500E+02	30.5	1.3558E+01	FishBase
5379	Ecto.verts	Cirrhinus cirrhosus	1.8000E+02	30.5	1.5493E+01	FishBase
5380	Ecto.verts	Clarias batrachus	1.5300E+01	26	9.3149E-01	FishBase
5381	Ecto.verts	Clarias batrachus	2.7250E+01	26	2.3837E+00	FishBase
5382	Ecto.verts	Clarias batrachus	3.2700E+01	26	2.1968E+00	FishBase
5383	Ecto.verts	Clarias batrachus	4.2500E+01	26	4.1043E+00	FishBase
5384	Ecto.verts	Clarias batrachus	5.4000E+01	26	4.3457E+00	FishBase
5385	Ecto.verts	Clarias batrachus	6.4400E+01	26	4.5968E+00	FishBase
5386	Ecto.verts	Clarias batrachus	7.7000E+01	26	2.9636E+00	FishBase
5387	Ecto.verts	Clarias batrachus	8.2000E+01	26	4.7628E+00	FishBase
5388	Ecto.verts	Clarias batrachus	9.5700E+01	26	5.3576E+00	FishBase
5389	Ecto.verts	Cnemidophorus murinus	8.5000E+01	27	4.9300E+00	White et al. 2006
5390	Ecto.verts	Cnemidophorus murinus	8.5000E+01	40	1.5555E+01	White et al. 2006
5391	Ecto.verts	Cnemidophorus tigris	1.8000E+01	20	1.1196E+00	White et al. 2006
5392	Ecto.verts	Cnemidophorus tigris	1.8000E+01	30	2.5200E+00	White et al. 2006

5393	Ecto.verts	Cnemidophorus tigris	1.8000E+01	37	4.8600E+00	White et al. 2006
5394	Ecto.verts	Coleonyx variegatus	3.4300E+00	25	5.0250E-01	White et al. 2006
5395	Ecto.verts	Colossoma macropomum	4.7000E+01	25	8.3212E+00	FishBase
5396	Ecto.verts	Colossoma macropomum	4.7000E+01	35	8.4528E+00	FishBase
5397	Ecto.verts	Colossoma macropomum	4.7000E+01	30	1.2761E+01	FishBase
5398	Ecto.verts	Colostethus inguinalis	1.5300E+00	25	2.1270E-01	White et al. 2006
5399	Ecto.verts	Colostethus inguinalis	1.5700E+00	20	1.3660E-01	White et al. 2006
5400	Ecto.verts	Colostethus nubicola	2.7000E-01	25	4.3500E-02	White et al. 2006
5401	Ecto.verts	Colostethus nubicola	2.8000E-01	20	3.1400E-02	White et al. 2006
5402	Ecto.verts	Colostethus trinitatus	1.0000E+00	25	2.0100E-01	White et al. 2006
5403	Ecto.verts	Coluber constrictor	2.6200E+02	35	1.6768E+01	White et al. 2006
5404	Ecto.verts	Conraua goliath	2.5100E+02	25	1.8860E+01	White et al. 2006
5405	Ecto.verts	Corallus caninus	5.5600E+02	20	4.9147E+00	White et al. 2006
5406	Ecto.verts	Corallus caninus	5.5600E+02	30	1.4754E+01	White et al. 2006
5407	Ecto.verts	Corallus caninus	5.5600E+02	34	1.9263E+01	White et al. 2006
5408	Ecto.verts	Corallus enhydris	8.0200E+02	20	7.8494E+00	White et al. 2006
5409	Ecto.verts	Corallus enhydris	8.0200E+02	30	1.8798E+01	White et al. 2006
5410	Ecto.verts	Corallus enhydris	8.0200E+02	34	2.7005E+01	White et al. 2006
5411	Ecto.verts	Crinia parinsignifera	4.5200E-01	30	1.7090E-01	White et al. 2006
5412	Ecto.verts	Crinia parinsignifera	5.9700E-01	10	5.0100E-02	White et al. 2006
5413	Ecto.verts	Crinia parinsignifera	6.3000E-01	20	1.1660E-01	White et al. 2006
5414	Ecto.verts	Crinia parinsignifera	6.5300E-01	5	3.9800E-02	White et al. 2006
5415	Ecto.verts	Crinia parinsignifera	6.5800E-01	15	1.0730E-01	White et al. 2006
5416	Ecto.verts	Crinia parinsignifera	6.6500E-01	35	3.7570E-01	White et al. 2006
5417	Ecto.verts	Crinia parinsignifera	6.6600E-01	25	1.7650E-01	White et al. 2006
5418	Ecto.verts	Crinia signifera	4.8500E-01	30	1.7610E-01	White et al. 2006

5419	Ecto.verts	Crinia signifera	6.1200E-01	35	3.9900E-01	White et al. 2006
5420	Ecto.verts	Crinia signifera	6.1500E-01	20	1.3100E-01	White et al. 2006
5421	Ecto.verts	Crinia signifera	6.2100E-01	25	1.4900E-01	White et al. 2006
5422	Ecto.verts	Crinia signifera	6.3800E-01	15	1.0780E-01	White et al. 2006
5423	Ecto.verts	Crinia signifera	6.8300E-01	5	4.1000E-02	White et al. 2006
5424	Ecto.verts	Crinia signifera	6.9000E-01	10	5.9300E-02	White et al. 2006
5425	Ecto.verts	Crinia signifera	8.5300E-01	15	1.3310E-01	White et al. 2006
5426	Ecto.verts	Crinia signifera	9.0800E-01	5	4.9900E-02	White et al. 2006
5427	Ecto.verts	Crinia signifera	9.6800E-01	10	8.2300E-02	White et al. 2006
5428	Ecto.verts	Crotalus viridis	3.0100E+02	35	1.9264E+01	White et al. 2006
5429	Ecto.verts	Crotalus viridis	3.0100E+02	35	2.4080E+01	White et al. 2006
5430	Ecto.verts	Crotaphytus collaris	3.0000E+01	20	2.4000E+00	White et al. 2006
5431	Ecto.verts	Crotaphytus collaris	3.0000E+01	30	5.4000E+00	White et al. 2006
5432	Ecto.verts	Crotaphytus collaris	3.0000E+01	37	8.4000E+00	White et al. 2006
5433	Ecto.verts	Cryptobranchus alleganiensis	4.2300E+02	25	1.3079E+01	White et al. 2006
5434	Ecto.verts	Cryptobranchus alleganiensis	5.1100E+02	15	1.0660E+01	White et al. 2006
5435	Ecto.verts	Cryptobranchus alleganiensis	5.1400E+02	5	4.4615E+00	White et al. 2006
5436	Ecto.verts	Ctenotus labillardieri	2.8000E+00	20	1.9992E-01	White et al. 2006
5437	Ecto.verts	Cyclothone acclinidens	2.3000E-01	3	7.2428E-03	FishBase
5438	Ecto.verts	Cyclothone acclinidens	2.3000E-01	3	8.0476E-03	FishBase
5439	Ecto.verts	Cyclothone acclinidens	5.2000E-01	3	1.0917E-02	FishBase
5440	Ecto.verts	Cyclothone acclinidens	5.2000E-01	3	1.2736E-02	FishBase
5441	Ecto.verts	Cyclothone acclinidens	8.7000E-01	3	1.4612E-02	FishBase
5442	Ecto.verts	Cyclothone acclinidens	8.7000E-01	3	1.6438E-02	FishBase
5443	Ecto.verts	Cyprinus carpio	1.0000E+01	10	7.9776E-01	FishBase
5444	Ecto.verts	Cyprinus carpio	1.0000E+01	15	1.0007E+00	FishBase

5445	Ecto.verts	Cyprinus carpio	1.0000E+01	20	1.4486E+00	FishBase
5446	Ecto.verts	Cyprinus carpio	4.7000E+01	10	5.5913E-01	FishBase
5447	Ecto.verts	Cyprinus carpio	4.7000E+01	20	1.6774E+00	FishBase
5448	Ecto.verts	Cyprinus carpio	4.7000E+01	30	3.7824E+00	FishBase
5449	Ecto.verts	Cyprinus carpio	4.7000E+01	35	4.4731E+00	FishBase
5450	Ecto.verts	Cyprinus carpio	4.7000E+01	25.1	5.1638E+00	FishBase
5451	Ecto.verts	Cyprinus carpio	1.0600E+02	30	7.7887E+00	FishBase
5452	Ecto.verts	Cyprinus carpio	1.3400E+02	35	1.0971E+01	FishBase
5453	Ecto.verts	Cyprinus carpio	1.4600E+02	20	4.9041E+00	FishBase
5454	Ecto.verts	Cyprinus carpio	1.7400E+02	10	2.0700E+00	FishBase
5455	Ecto.verts	Cyprinus carpio	2.1500E+02	10	3.1596E+00	FishBase
5456	Ecto.verts	Cyprinus carpio	3.1500E+02	20	1.0801E+01	FishBase
5457	Ecto.verts	Cyprinus carpio	3.1500E+02	20	1.1903E+01	FishBase
5458	Ecto.verts	Cyprinus carpio	5.5400E+02	24.5	3.2178E+01	FishBase
5459	Ecto.verts	Cyprinus carpio	5.6100E+02	24.5	2.5518E+01	FishBase
5460	Ecto.verts	Cyprinus carpio	5.6100E+02	24.5	2.7481E+01	FishBase
5461	Ecto.verts	Cyprinus carpio	5.6100E+02	24.5	2.9051E+01	FishBase
5462	Ecto.verts	Cyprinus carpio	5.6100E+02	24.5	2.9836E+01	FishBase
5463	Ecto.verts	Cyprinus carpio	5.6100E+02	24.5	3.1014E+01	FishBase
5464	Ecto.verts	Cyprinus carpio	5.6100E+02	24.5	3.3762E+01	FishBase
5465	Ecto.verts	Cyprinus carpio	5.6100E+02	24.5	3.6510E+01	FishBase
5466	Ecto.verts	Cyprinus carpio	5.6100E+02	24.5	3.6903E+01	FishBase
5467	Ecto.verts	Cyprinus carpio	5.9800E+02	25.5	3.1804E+01	FishBase
5468	Ecto.verts	Cyprinus carpio	1.4530E+03	15	4.4739E+01	FishBase
5469	Ecto.verts	Cyprinus carpio	1.7460E+03	15	7.8197E+01	FishBase
5470	Ecto.verts	Cyprinus carpio	1.9050E+03	15	6.2656E+01	FishBase

5471	Ecto.verts	Cyprinus carpio	1.9600E+03	15	7.9552E+01	FishBase
5472	Ecto.verts	Dasyatis sabina	3.2900E+02	23	1.6346E+01	FishBase
5473	Ecto.verts	Dasyatis sabina	3.5000E+02	23.5	2.3513E+01	FishBase
5474	Ecto.verts	Dasyatis sabina	4.0700E+02	24.2	2.0222E+01	FishBase
5475	Ecto.verts	Dasyatis sabina	4.3100E+02	23.8	2.2922E+01	FishBase
5476	Ecto.verts	Dasyatis sabina	4.6300E+02	22.5	2.4624E+01	FishBase
5477	Ecto.verts	Dasyatis sabina	5.0300E+02	22.2	2.1824E+01	FishBase
5478	Ecto.verts	Dasyatis sabina	5.0400E+02	23	2.5747E+01	FishBase
5479	Ecto.verts	Dasyatis sabina	5.0400E+02	22.4	3.3506E+01	FishBase
5480	Ecto.verts	Dasyatis sabina	5.6400E+02	21.5	3.1575E+01	FishBase
5481	Ecto.verts	Dasyatis sabina	8.5900E+02	22.5	6.5522E+01	FishBase
5482	Ecto.verts	Dendrobates auratus	1.7700E+00	25	1.5930E-01	White et al. 2006
5483	Ecto.verts	Dendrobates auratus	2.0900E+00	20	1.4210E-01	White et al. 2006
5484	Ecto.verts	Desmognathus fuscus	8.0000E-01	5	1.3200E-02	White et al. 2006
5485	Ecto.verts	Desmognathus fuscus	9.1000E-01	15	2.9100E-02	White et al. 2006
5486	Ecto.verts	Desmognathus fuscus	1.1700E+00	5	1.6300E-02	White et al. 2006
5487	Ecto.verts	Desmognathus fuscus	1.2400E+00	15	4.2300E-02	White et al. 2006
5488	Ecto.verts	Desmognathus fuscus	1.3100E+00	17	5.9000E-02	White et al. 2006
5489	Ecto.verts	Desmognathus fuscus	1.6700E+00	10	5.2500E-02	White et al. 2006
5490	Ecto.verts	Desmognathus fuscus	1.7000E+00	15	5.8400E-02	White et al. 2006
5491	Ecto.verts	Desmognathus fuscus	1.9200E+00	20	9.7400E-02	White et al. 2006
5492	Ecto.verts	Desmognathus fuscus	1.9500E+00	10	2.1390E-01	White et al. 2006
5493	Ecto.verts	Desmognathus fuscus	2.0700E+00	5	3.5000E-02	White et al. 2006
5494	Ecto.verts	Desmognathus fuscus	2.0900E+00	20	8.9500E-02	White et al. 2006
5495	Ecto.verts	Desmognathus fuscus	2.2600E+00	20	1.0220E-01	White et al. 2006
5496	Ecto.verts	Desmognathus fuscus	2.5300E+00	16.5	9.7200E-02	White et al. 2006

5497	Ecto.verts	Desmognathus fuscus	4.5000E+00	13	1.9350E-01	White et al. 2006
5498	Ecto.verts	Desmognathus monticola	3.4300E+00	20	1.4540E-01	White et al. 2006
5499	Ecto.verts	Desmognathus monticola	5.4000E+00	15	3.0780E-01	White et al. 2006
5500	Ecto.verts	Desmognathus ochrophaes	9.0000E-01	5	1.0000E-02	White et al. 2006
5501	Ecto.verts	Desmognathus ochrophaes	9.0000E-01	10	1.7000E-02	White et al. 2006
5502	Ecto.verts	Desmognathus ochrophaes	9.0000E-01	14	2.0000E-02	White et al. 2006
5503	Ecto.verts	Desmognathus ochrophaes	9.0000E-01	21	3.8000E-02	White et al. 2006
5504	Ecto.verts	Desmognathus ochrophaes	9.0000E-01	17.5	4.5000E-02	White et al. 2006
5505	Ecto.verts	Desmognathus ochrophaes	1.0700E+00	5	1.7300E-02	White et al. 2006
5506	Ecto.verts	Desmognathus ochrophaes	1.0900E+00	15	3.9200E-02	White et al. 2006
5507	Ecto.verts	Desmognathus ochrophaes	1.1100E+00	20	6.1500E-02	White et al. 2006
5508	Ecto.verts	Desmognathus ochrophaes	1.1600E+00	10	3.4100E-02	White et al. 2006
5509	Ecto.verts	Desmognathus ochrophaes	1.1700E+00	5	1.6300E-02	White et al. 2006
5510	Ecto.verts	Desmognathus ochrophaes	1.2400E+00	15	4.2300E-02	White et al. 2006
5511	Ecto.verts	Desmognathus ochrophaes	1.4000E+00	17.5	5.1000E-02	White et al. 2006
5512	Ecto.verts	Desmognathus ochrophaes	1.6000E+00	17.5	4.2700E-02	White et al. 2006
5513	Ecto.verts	Desmognathus ochrophaes	1.9650E+00	15	1.7690E-01	White et al. 2006
5514	Ecto.verts	Desmognathus ochrophaes	2.4300E+00	20	1.1960E-01	White et al. 2006
5515	Ecto.verts	Desmognathus ochrophaes	2.5000E+00	17.5	8.2000E-02	White et al. 2006
5516	Ecto.verts	Desmognathus ochrophaes	2.5600E+00	15	8.2200E-02	White et al. 2006
5517	Ecto.verts	Desmognathus ochrophaes	2.6800E+00	10	4.9600E-02	White et al. 2006
5518	Ecto.verts	Desmognathus ochrophaes	2.7500E+00	5	3.3800E-02	White et al. 2006
5519	Ecto.verts	Desmognathus quadramaculatus	2.2400E+00	20	9.7700E-02	White et al. 2006
5520	Ecto.verts	Desmognathus quadramaculatus	9.2500E+00	5	2.6460E-01	White et al. 2006
5521	Ecto.verts	Desmognathus quadramaculatus	9.2500E+00	10	4.0330E-01	White et al. 2006
5522	Ecto.verts	Desmognathus quadramaculatus	9.2500E+00	15	5.3370E-01	White et al. 2006

5523	Ecto.verts	Desmognathus quadramaculatus	9.2500E+00	20	6.2160E-01	White et al. 2006
5524	Ecto.verts	Desmognathus quadramaculatus	9.2500E+00	25	7.1040E-01	White et al. 2006
5525	Ecto.verts	Desmognathus quadramaculatus	1.0100E+01	10	4.4440E-01	White et al. 2006
5526	Ecto.verts	Desmognathus quadramaculatus	1.3300E+01	20	9.3230E-01	White et al. 2006
5527	Ecto.verts	Desmognathus quadramaculatus	1.5100E+01	25	1.1612E+00	White et al. 2006
5528	Ecto.verts	Desmognathus quadramaculatus	1.6400E+01	5	4.7230E-01	White et al. 2006
5529	Ecto.verts	Desmognathus quadramaculatus	1.6800E+01	15	9.3410E-01	White et al. 2006
5530	Ecto.verts	Desmognathus quadramaculatus	1.9360E+01	20	8.2670E-01	White et al. 2006
5531	Ecto.verts	Desmognathus quadramaculatus	2.2280E+01	15	4.3000E-01	White et al. 2006
5532	Ecto.verts	Desmognathus quadramaculatus	2.2820E+01	25	1.1866E+00	White et al. 2006
5533	Ecto.verts	Desmognathus quadramaculatus	2.3410E+01	5	1.6390E-01	White et al. 2006
5534	Ecto.verts	Diadophis punctatus	4.4000E+00	30	5.8080E-01	White et al. 2006
5535	Ecto.verts	Diadophis punctatus	5.0000E+00	20	2.8000E-01	White et al. 2006
5536	Ecto.verts	Dicamptodon ensatus	2.4000E+01	15	7.4880E-01	White et al. 2006
5537	Ecto.verts	Dicamptodon ensatus	1.0125E+02	15	1.3061E+00	White et al. 2006
5538	Ecto.verts	Diplometopon zarudnyi	6.3400E+00	10	2.5360E-01	White et al. 2006
5539	Ecto.verts	Diplometopon zarudnyi	6.3400E+00	15	3.1700E-01	White et al. 2006
5540	Ecto.verts	Diplometopon zarudnyi	6.3400E+00	20	5.7060E-01	White et al. 2006
5541	Ecto.verts	Diplometopon zarudnyi	6.3400E+00	25	7.0374E-01	White et al. 2006
5542	Ecto.verts	Diplometopon zarudnyi	6.3400E+00	30	7.0374E-01	White et al. 2006
5543	Ecto.verts	Diplometopon zarudnyi	6.3400E+00	35	9.7002E-01	White et al. 2006
5544	Ecto.verts	Dipsas albifrons	2.2000E+01	20	7.9200E-01	White et al. 2006
5545	Ecto.verts	Dipsosaurus dorsalis	4.0360E+01	20	1.6144E+00	White et al. 2006
5546	Ecto.verts	Dipsosaurus dorsalis	4.0360E+01	25	1.8767E+00	White et al. 2006
5547	Ecto.verts	Dipsosaurus dorsalis	4.0360E+01	30	2.8252E+00	White et al. 2006
5548	Ecto.verts	Dipsosaurus dorsalis	4.0360E+01	37	4.9643E+00	White et al. 2006

5549	Ecto.verts	Dipsosaurus dorsalis	4.0360E+01	35	6.0540E+00	White et al. 2006
5550	Ecto.verts	Dipsosaurus dorsalis	4.0360E+01	40	7.2648E+00	White et al. 2006
5551	Ecto.verts	Dipsosaurus dorsalis	4.0360E+01	45	1.0090E+01	White et al. 2006
5552	Ecto.verts	Discoglossus pictus	3.0700E+01	20	1.1359E+00	White et al. 2006
5553	Ecto.verts	Discoglossus pictus	3.0710E+01	20	1.1420E+00	White et al. 2006
5554	Ecto.verts	Egernia cunninghami	2.6100E+02	20	8.8740E+00	White et al. 2006
5555	Ecto.verts	Egernia cunninghami	2.6100E+02	30	2.2707E+01	White et al. 2006
5556	Ecto.verts	Elaphe guttata	8.0000E+02	25	9.0400E+01	White et al. 2006
5557	Ecto.verts	Electrona antarctica	4.7000E+01	0.5	1.6774E+00	FishBase
5558	Ecto.verts	Eleginus gracilis	4.7000E+01	2.5	2.2365E+00	FishBase
5559	Ecto.verts	Eleginus gracilis	4.7000E+01	7	3.3219E+00	FishBase
5560	Ecto.verts	Eleginus gracilis	4.7000E+01	12	4.6046E+00	FishBase
5561	Ecto.verts	Eleutherodactylus coqui	4.0600E+00	20	1.6700E-01	White et al. 2006
5562	Ecto.verts	Eleutherodactylus coqui	4.0600E+00	20	1.7860E-01	White et al. 2006
5563	Ecto.verts	Eleutherodactylus coqui	4.1000E+00	20	1.8040E-01	White et al. 2006
5564	Ecto.verts	Eleutherodactylus portoricensis	3.7000E+00	15	2.5660E-01	White et al. 2006
5565	Ecto.verts	Encheliophis homei	1.6000E-01	30	2.1721E-02	FishBase
5566	Ecto.verts	Encheliophis homei	2.3000E-01	30	3.7985E-02	FishBase
5567	Ecto.verts	Encheliophis homei	4.4000E-01	30	9.9454E-02	FishBase
5568	Ecto.verts	Encheliophis homei	4.6000E-01	30	7.2106E-02	FishBase
5569	Ecto.verts	Encheliophis homei	1.2000E+00	30	1.1756E-01	FishBase
5570	Ecto.verts	Encheliophis homei	7.5000E+00	30	4.5136E-01	FishBase
5571	Ecto.verts	Engraulis japonicus	6.2879E+02	16.2	9.1964E+01	FishBase
5572	Ecto.verts	Engraulis japonicus	6.2879E+02	16.2	1.1969E+02	FishBase
5573	Ecto.verts	Engraulis japonicus	6.2879E+02	16.2	1.4169E+02	FishBase
5574	Ecto.verts	Engraulis japonicus	6.3435E+02	16.2	3.2406E+02	FishBase

5575	Ecto.verts	Ensatina eschscholtzi	3.0800E-01	14	1.7600E-02	White et al. 2006
5576	Ecto.verts	Ensatina eschscholtzi	3.3900E+00	15	1.2920E-01	White et al. 2006
5577	Ecto.verts	Ensatina eschscholtzi	5.3000E+00	25	3.5720E-01	White et al. 2006
5578	Ecto.verts	Epicrates cenchria	4.1600E+02	20	5.0342E+00	White et al. 2006
5579	Ecto.verts	Epicrates cenchria	4.1600E+02	30	1.2159E+01	White et al. 2006
5580	Ecto.verts	Epicrates cenchria	4.1600E+02	34	1.9384E+01	White et al. 2006
5581	Ecto.verts	Erpetoichthys calabaricus	2.7400E+01	27	2.1859E+00	FishBase
5582	Ecto.verts	Eryx colubrinus	8.5850E+01	20	1.2724E+00	White et al. 2006
5583	Ecto.verts	Eryx colubrinus	8.5850E+01	30	3.3459E+00	White et al. 2006
5584	Ecto.verts	Eryx colubrinus	8.5850E+01	34	4.8111E+00	White et al. 2006
5585	Ecto.verts	Esox lucius	6.0000E+02	5	8.3975E+00	FishBase
5586	Ecto.verts	Esox lucius	6.0000E+02	10	1.6795E+01	FishBase
5587	Ecto.verts	Eumeces fasciatus	7.0000E+00	30	1.6800E+00	White et al. 2006
5588	Ecto.verts	Eumeces inexpectatus	9.6000E+00	30	1.4688E+00	White et al. 2006
5589	Ecto.verts	Eumeces obsoletus	3.0000E+01	20	1.5000E+00	White et al. 2006
5590	Ecto.verts	Eumeces obsoletus	3.0000E+01	30	5.1000E+00	White et al. 2006
5591	Ecto.verts	Eumeces obsoletus	3.0000E+01	37	1.0200E+01	White et al. 2006
5592	Ecto.verts	Eunectes murinus	1.1300E+03	20	2.3730E+01	White et al. 2006
5593	Ecto.verts	Eunectes notaeus	1.4400E+04	20	2.1600E+02	White et al. 2006
5594	Ecto.verts	Eurycea bislineata	9.3000E-01	15	4.9400E-02	White et al. 2006
5595	Ecto.verts	Eurycea bislineata	9.3000E-01	20	5.3900E-02	White et al. 2006
5596	Ecto.verts	Eurycea bislineata	1.0410E+00	1	1.6000E-02	White et al. 2006
5597	Ecto.verts	Eurycea bislineata	1.0700E+00	10	2.6300E-02	White et al. 2006
5598	Ecto.verts	Eurycea bislineata	1.0920E+00	10	4.8900E-02	White et al. 2006
5599	Ecto.verts	Eurycea bislineata	1.1400E+00	5	1.5700E-02	White et al. 2006
5600	Ecto.verts	Eurycea bislineata	1.1700E+00	16	6.7400E-02	White et al. 2006

5601	Ecto.verts	Eurycea longicauda	1.5700E+00	15	5.0400E-02	White et al. 2006
5602	Ecto.verts	Eurycea multiplicata	6.3000E-01	10	9.3000E-03	White et al. 2006
5603	Ecto.verts	Eurycea multiplicata	6.4000E-01	5	8.8000E-03	White et al. 2006
5604	Ecto.verts	Eurycea multiplicata	6.5000E-01	15	1.1620E-01	White et al. 2006
5605	Ecto.verts	Eurycea multiplicata	7.1000E-01	15	1.5500E-02	White et al. 2006
5606	Ecto.verts	Eurycea multiplicata	7.3000E-01	25	5.5500E-02	White et al. 2006
5607	Ecto.verts	Eurycea multiplicata	7.8000E-01	5	3.2200E-02	White et al. 2006
5608	Ecto.verts	Eurycea multiplicata	7.9000E-01	20	1.7640E-01	White et al. 2006
5609	Ecto.verts	Eurycea multiplicata	8.0000E-01	10	8.5800E-02	White et al. 2006
5610	Ecto.verts	Eurycea multiplicata	8.4000E-01	20	9.3100E-02	White et al. 2006
5611	Ecto.verts	Eurycea nana	1.5400E-01	25	1.3200E-02	White et al. 2006
5612	Ecto.verts	Eurycea neotenes	2.4300E-01	25	2.5800E-02	White et al. 2006
5613	Ecto.verts	Eurycea pterophila	1.9600E-01	25	1.7600E-02	White et al. 2006
5614	Ecto.verts	Euthynnus affinis	2.2600E+03	24	7.9551E+02	FishBase
5615	Ecto.verts	Exodon paradoxus	3.6000E+00	30	2.6704E-01	FishBase
5616	Ecto.verts	Exodon paradoxus	3.8500E+00	20	9.9685E-02	FishBase
5617	Ecto.verts	Exodon paradoxus	4.7700E+00	25	2.8707E-01	FishBase
5618	Ecto.verts	Fundulus parvipinnis	6.0000E+00	20	6.0042E-01	FishBase
5619	Ecto.verts	Gadus macrocephalus	4.7000E+01	6.5	3.2561E+00	FishBase
5620	Ecto.verts	Gadus morhua	1.4700E+01	12	1.6665E+00	FishBase
5621	Ecto.verts	Gadus morhua	1.4700E+01	12	1.8619E+00	FishBase
5622	Ecto.verts	Gadus morhua	1.8300E+01	12	1.5752E+00	FishBase
5623	Ecto.verts	Gadus morhua	1.8300E+01	12	1.7544E+00	FishBase
5624	Ecto.verts	Gadus morhua	2.0400E+01	12	1.6703E+00	FishBase
5625	Ecto.verts	Gadus morhua	2.0400E+01	12	2.0700E+00	FishBase
5626	Ecto.verts	Gadus morhua	4.7000E+01	3	3.6179E+00	FishBase

5627	Ecto.verts	Gadus morhua	4.7000E+01	12	4.0455E+00	FishBase
5628	Ecto.verts	Gadus morhua	4.7000E+01	10	4.6375E+00	FishBase
5629	Ecto.verts	Gadus morhua	6.1100E+01	12	5.2164E+00	FishBase
5630	Ecto.verts	Gadus morhua	6.1100E+01	12	6.1143E+00	FishBase
5631	Ecto.verts	Gadus morhua	7.2000E+01	12	4.6858E+00	FishBase
5632	Ecto.verts	Gadus morhua	1.4000E+02	15	1.2736E+01	FishBase
5633	Ecto.verts	Gadus morhua	1.5150E+02	15	1.3994E+01	FishBase
5634	Ecto.verts	Gadus morhua	1.7000E+02	11.5	1.3681E+01	FishBase
5635	Ecto.verts	Gadus morhua	1.7300E+02	12	9.9272E+00	FishBase
5636	Ecto.verts	Gadus morhua	1.8810E+02	10	1.2900E+01	FishBase
5637	Ecto.verts	Gadus morhua	2.1500E+02	12	1.6099E+01	FishBase
5638	Ecto.verts	Gadus morhua	2.1800E+02	11.5	1.2967E+01	FishBase
5639	Ecto.verts	Gadus morhua	2.2800E+02	12	1.3881E+01	FishBase
5640	Ecto.verts	Gadus morhua	2.2800E+02	12	2.0104E+01	FishBase
5641	Ecto.verts	Gadus morhua	2.4400E+02	11.5	1.4514E+01	FishBase
5642	Ecto.verts	Gadus morhua	2.5400E+02	12	1.3864E+01	FishBase
5643	Ecto.verts	Gadus morhua	2.6800E+02	12	1.1628E+01	FishBase
5644	Ecto.verts	Gadus morhua	2.8300E+02	12	1.3863E+01	FishBase
5645	Ecto.verts	Gadus morhua	2.8300E+02	11.5	1.9210E+01	FishBase
5646	Ecto.verts	Gadus morhua	2.8600E+02	11.5	1.7212E+01	FishBase
5647	Ecto.verts	Gadus morhua	3.0400E+02	11.5	9.5731E+00	FishBase
5648	Ecto.verts	Gadus morhua	3.1600E+02	11.5	1.4153E+01	FishBase
5649	Ecto.verts	Gadus morhua	3.3400E+02	12	3.2722E+01	FishBase
5650	Ecto.verts	Gadus morhua	3.5000E+02	15	2.0574E+01	FishBase
5651	Ecto.verts	Gadus morhua	3.5300E+02	12	1.9021E+01	FishBase
5652	Ecto.verts	Gadus morhua	3.8200E+02	11.5	1.4970E+01	FishBase

5653	Ecto.verts	Gadus morhua	4.4000E+02	11.5	2.8635E+01	FishBase
5654	Ecto.verts	Gadus morhua	4.6400E+02	12	3.2795E+01	FishBase
5655	Ecto.verts	Gadus morhua	5.1000E+02	15	2.9265E+01	FishBase
5656	Ecto.verts	Gadus morhua	5.1800E+02	12	2.1387E+01	FishBase
5657	Ecto.verts	Gadus morhua	5.1800E+02	12	3.8424E+01	FishBase
5658	Ecto.verts	Gadus morhua	6.1100E+02	12	5.3446E+01	FishBase
5659	Ecto.verts	Gadus morhua	6.4500E+02	12	5.0553E+01	FishBase
5660	Ecto.verts	Gadus morhua	7.0000E+02	15	3.6739E+01	FishBase
5661	Ecto.verts	Gadus morhua	7.2000E+02	12	2.6200E+01	FishBase
5662	Ecto.verts	Gadus morhua	8.0300E+02	12	4.0459E+01	FishBase
5663	Ecto.verts	Gadus morhua	8.4800E+02	12	2.7891E+01	FishBase
5664	Ecto.verts	Gadus morhua	9.4700E+02	12	3.4460E+01	FishBase
5665	Ecto.verts	Gadus morhua	1.0000E+03	12	3.8488E+01	FishBase
5666	Ecto.verts	Gadus morhua	1.1200E+03	12	6.6620E+01	FishBase
5667	Ecto.verts	Gadus morhua	1.1400E+03	5	3.1113E+01	FishBase
5668	Ecto.verts	Gadus morhua	1.1500E+03	10	4.6676E+01	FishBase
5669	Ecto.verts	Gadus morhua	1.4400E+03	15	6.3485E+01	FishBase
5670	Ecto.verts	Gadus morhua	1.5800E+03	15	8.6242E+01	FishBase
5671	Ecto.verts	Gadus morhua	2.2000E+03	15	1.0931E+02	FishBase
5672	Ecto.verts	Gadus morhua	2.3300E+03	5	5.8698E+01	FishBase
5673	Ecto.verts	Gadus morhua	3.0300E+03	10	1.1662E+02	FishBase
5674	Ecto.verts	Gambusia affinis	2.2000E-01	20	4.2029E-02	FishBase
5675	Ecto.verts	Gambusia affinis	2.2000E-01	25	5.6039E-02	FishBase
5676	Ecto.verts	Gambusia affinis	2.2000E-01	30	7.0049E-02	FishBase
5677	Ecto.verts	Gambusia affinis	4.7000E+01	28	1.4669E+01	FishBase
5678	Ecto.verts	Garthia gaudichaudi	8.0500E-01	25	7.6958E-02	White et al. 2006

5679	Ecto.verts	Garthia gaudichaudi	8.0500E-01	35	1.9240E-01	White et al. 2006
5680	Ecto.verts	Gastrophryne carolinesis	1.9000E+00	20	9.8800E-02	White et al. 2006
5681	Ecto.verts	Gastrophryne carolinesis	1.9400E+00	20	1.0300E-01	White et al. 2006
5682	Ecto.verts	Gekko gecko	6.1500E+01	30	5.9286E+00	White et al. 2006
5683	Ecto.verts	Genyagnus monopterygius	5.3920E+01	17	9.4332E-01	FishBase
5684	Ecto.verts	Genyagnus monopterygius	6.6500E+01	17	1.2099E+00	FishBase
5685	Ecto.verts	Genyagnus monopterygius	6.9500E+01	17	1.1672E+00	FishBase
5686	Ecto.verts	Genyagnus monopterygius	7.4800E+01	17	1.2039E+00	FishBase
5687	Ecto.verts	Genyagnus monopterygius	8.1900E+01	17	9.1700E-01	FishBase
5688	Ecto.verts	Genyagnus monopterygius	1.3900E+02	17	2.0427E+00	FishBase
5689	Ecto.verts	Genyagnus monopterygius	1.6400E+02	17	1.1477E+00	FishBase
5690	Ecto.verts	Genyagnus monopterygius	1.6400E+02	17	1.4920E+00	FishBase
5691	Ecto.verts	Genyagnus monopterygius	1.6400E+02	17	1.8362E+00	FishBase
5692	Ecto.verts	Genyagnus monopterygius	1.6400E+02	17	2.0658E+00	FishBase
5693	Ecto.verts	Genyagnus monopterygius	1.6400E+02	17	2.1805E+00	FishBase
5694	Ecto.verts	Genyagnus monopterygius	1.6400E+02	17	2.1805E+00	FishBase
5695	Ecto.verts	Genyagnus monopterygius	1.6400E+02	17	2.1805E+00	FishBase
5696	Ecto.verts	Genyagnus monopterygius	1.6400E+02	17	2.1805E+00	FishBase
5697	Ecto.verts	Genyagnus monopterygius	1.6400E+02	17	2.2953E+00	FishBase
5698	Ecto.verts	Genyagnus monopterygius	1.6400E+02	17	2.2953E+00	FishBase
5699	Ecto.verts	Genyagnus monopterygius	1.6400E+02	17	2.4101E+00	FishBase
5700	Ecto.verts	Genyagnus monopterygius	1.8200E+02	17	2.1652E+00	FishBase
5701	Ecto.verts	Genyagnus monopterygius	2.3100E+02	17	2.9097E+00	FishBase
5702	Ecto.verts	Genyagnus monopterygius	2.3800E+02	17	3.6641E+00	FishBase
5703	Ecto.verts	Genyagnus monopterygius	2.7600E+02	17	3.0903E+00	FishBase
5704	Ecto.verts	Geotrypetes seraphini	1.9300E+00	20	7.1400E-02	White et al. 2006

5705	Ecto.verts	Gerrhonotus multicarinatus	2.3000E+01	30	4.9300E+00	White et al. 2006
5706	Ecto.verts	Gerrhonotus multicarinatus	2.9000E+01	20	1.6472E+00	White et al. 2006
5707	Ecto.verts	Gillichthys mirabilis	2.0000E-01	15	1.9594E-02	FishBase
5708	Ecto.verts	Gillichthys mirabilis	3.4000E-01	15	2.3079E-02	FishBase
5709	Ecto.verts	Gillichthys mirabilis	4.1000E-01	15	2.8978E-02	FishBase
5710	Ecto.verts	Gillichthys mirabilis	4.8000E-01	15	2.7544E-02	FishBase
5711	Ecto.verts	Gillichthys mirabilis	6.1000E-01	15	4.8237E-02	FishBase
5712	Ecto.verts	Gillichthys mirabilis	6.3000E-01	15	2.7334E-02	FishBase
5713	Ecto.verts	Gillichthys mirabilis	6.8000E-01	15	4.1400E-02	FishBase
5714	Ecto.verts	Gillichthys mirabilis	9.1000E-01	15	4.5850E-02	FishBase
5715	Ecto.verts	Gillichthys mirabilis	1.2900E+00	15	6.8607E-02	FishBase
5716	Ecto.verts	Gillichthys mirabilis	1.8000E+00	15	6.8020E-02	FishBase
5717	Ecto.verts	Gillichthys mirabilis	1.8800E+00	15	1.0393E-01	FishBase
5718	Ecto.verts	Gillichthys mirabilis	2.1100E+00	15	1.1222E-01	FishBase
5719	Ecto.verts	Gillichthys mirabilis	2.2000E+00	15	1.1854E-01	FishBase
5720	Ecto.verts	Gillichthys mirabilis	2.4000E+00	15	9.2372E-02	FishBase
5721	Ecto.verts	Gillichthys mirabilis	2.4000E+00	15	1.0749E-01	FishBase
5722	Ecto.verts	Gillichthys mirabilis	2.8900E+00	15	1.4763E-01	FishBase
5723	Ecto.verts	Gillichthys mirabilis	2.9300E+00	15	1.2507E-01	FishBase
5724	Ecto.verts	Gillichthys mirabilis	2.9300E+00	15	1.4763E-01	FishBase
5725	Ecto.verts	Gillichthys mirabilis	3.2400E+00	15	9.9762E-02	FishBase
5726	Ecto.verts	Gillichthys mirabilis	4.1400E+00	15	1.5645E-01	FishBase
5727	Ecto.verts	Gillichthys mirabilis	4.7200E+00	15	1.9488E-01	FishBase
5728	Ecto.verts	Glossogobius giuris	1.6000E+00	22	3.7957E-01	FishBase
5729	Ecto.verts	Glossogobius giuris	2.5000E+00	22	4.9860E-01	FishBase
5730	Ecto.verts	Glossogobius giuris	3.8000E+00	22	7.5521E-01	FishBase

5731	Ecto.verts	Glossogobius giuris	4.6000E+00	22	6.9209E-01	FishBase
5732	Ecto.verts	Glossogobius giuris	7.3000E+00	22	1.2720E+00	FishBase
5733	Ecto.verts	Glossogobius giuris	8.3000E+00	22	1.2314E+00	FishBase
5734	Ecto.verts	Glossogobius giuris	1.0200E+01	22	1.3705E+00	FishBase
5735	Ecto.verts	Glossogobius giuris	1.1000E+01	22	1.2624E+00	FishBase
5736	Ecto.verts	Glossogobius giuris	1.2000E+01	22	1.4696E+00	FishBase
5737	Ecto.verts	Glossogobius giuris	1.2900E+01	25	1.1555E+00	FishBase
5738	Ecto.verts	Glossogobius giuris	1.3130E+01	25	1.3047E+00	FishBase
5739	Ecto.verts	Glossogobius giuris	1.3350E+01	25	1.4013E+00	FishBase
5740	Ecto.verts	Glossogobius giuris	1.3500E+01	22	1.4454E+00	FishBase
5741	Ecto.verts	Glossogobius giuris	1.3500E+01	25	1.7005E+00	FishBase
5742	Ecto.verts	Glossogobius giuris	1.3760E+01	25	1.2422E+00	FishBase
5743	Ecto.verts	Glossogobius giuris	1.4140E+01	25	1.2270E+00	FishBase
5744	Ecto.verts	Glossogobius giuris	1.4170E+01	25	1.2792E+00	FishBase
5745	Ecto.verts	Glossogobius giuris	1.4300E+01	22	1.7612E+00	FishBase
5746	Ecto.verts	Glossogobius giuris	1.6500E+01	22	1.3163E+00	FishBase
5747	Ecto.verts	Glossogobius giuris	1.7700E+01	22	1.5483E+00	FishBase
5748	Ecto.verts	Glossogobius giuris	1.8700E+01	22	2.2115E+00	FishBase
5749	Ecto.verts	Glossogobius giuris	1.9300E+01	22	2.2285E+00	FishBase
5750	Ecto.verts	Glossogobius giuris	2.0400E+01	22	1.9986E+00	FishBase
5751	Ecto.verts	Glossogobius giuris	2.3200E+01	22	1.9807E+00	FishBase
5752	Ecto.verts	Glossogobius giuris	4.7000E+01	22	3.3219E+00	FishBase
5753	Ecto.verts	Gobionotothen angustifrons	4.7000E+01	3	3.3219E+00	FishBase
5754	Ecto.verts	Gonatodes antillensis	1.8000E+00	27	1.9800E-01	White et al. 2006
5755	Ecto.verts	Gonatodes antillensis	1.8000E+00	34	2.9880E-01	White et al. 2006
5756	Ecto.verts	Gymnelus viridis	4.7000E+01	-1.5	5.9202E-01	FishBase

5757	Ecto.verts	Gymnocanthus tricuspis	4.7000E+01	-1.5	9.8670E-01	FishBase
5758	Ecto.verts	Gymnoscopelus braueri	4.7000E+01	0.5	1.2498E+00	FishBase
5759	Ecto.verts	Gymnoscopelus opisthopterus	4.7000E+01	0.5	9.5381E-01	FishBase
5760	Ecto.verts	Gyrinophilus danielsi	1.2780E+01	2	1.0350E-01	White et al. 2006
5761	Ecto.verts	Gyrinophilus danielsi	1.3760E+01	25	9.1090E-01	White et al. 2006
5762	Ecto.verts	Gyrinophilus danielsi	1.4440E+01	15	3.2200E-01	White et al. 2006
5763	Ecto.verts	Gyrinophilus porphyrictus	7.2800E+00	5	5.3100E-02	White et al. 2006
5764	Ecto.verts	Gyrinophilus porphyrictus	7.3600E+00	25	4.8580E-01	White et al. 2006
5765	Ecto.verts	Gyrinophilus porphyrictus	7.6700E+00	15	1.7950E-01	White et al. 2006
5766	Ecto.verts	Gyrinophilus porphyrictus	1.2400E+01	15.5	1.8850E-01	White et al. 2006
5767	Ecto.verts	Gyrinophilus porphyrictus	1.5300E+01	15	8.8740E-01	White et al. 2006
5768	Ecto.verts	Harpagifer georgianus	4.7000E+01	3	9.8670E-01	FishBase
5769	Ecto.verts	Helicops modestus	1.9600E+02	20	6.8600E+00	White et al. 2006
5770	Ecto.verts	Helicops modestus	1.9600E+02	25	7.8400E+00	White et al. 2006
5771	Ecto.verts	Helicops modestus	1.9600E+02	30	1.0780E+01	White et al. 2006
5772	Ecto.verts	Hemidactylus frenatus	2.0000E+00	27	2.1000E-01	White et al. 2006
5773	Ecto.verts	Hemidactylus frenatus	4.4000E+00	20	2.5960E-01	White et al. 2006
5774	Ecto.verts	Hemidactylus frenatus	4.4000E+00	26	6.8640E-01	White et al. 2006
5775	Ecto.verts	Hemidactylus frenatus	4.4000E+00	30	9.1960E-01	White et al. 2006
5776	Ecto.verts	Hemidactylus platyurus	3.5000E+00	27	3.5000E-01	White et al. 2006
5777	Ecto.verts	Heteropneustes fossilis	9.0000E+00	26	1.2281E+00	FishBase
5778	Ecto.verts	Heteropneustes fossilis	1.0000E+01	26	1.1966E+00	FishBase
5779	Ecto.verts	Heteropneustes fossilis	1.5000E+01	26	1.3961E+00	FishBase
5780	Ecto.verts	Heteropneustes fossilis	1.6000E+01	26	1.4556E+00	FishBase
5781	Ecto.verts	Heteropneustes fossilis	1.7000E+01	26	1.4752E+00	FishBase
5782	Ecto.verts	Heteropneustes fossilis	2.3000E+01	26	1.9797E+00	FishBase

5783	Ecto.verts	Heteropneustes fossilis	2.6000E+01	26	2.2015E+00	FishBase
5784	Ecto.verts	Heteropneustes fossilis	3.6000E+01	26	2.9223E+00	FishBase
5785	Ecto.verts	Heteropneustes fossilis	4.4000E+01	26	3.5717E+00	FishBase
5786	Ecto.verts	Heteropneustes fossilis	5.6000E+01	26	4.4675E+00	FishBase
5787	Ecto.verts	Heteropneustes fossilis	7.5000E+01	26	4.8810E+00	FishBase
5788	Ecto.verts	Hippoglossoides platessoides	4.7000E+01	3	8.8803E-01	FishBase
5789	Ecto.verts	Hippoglossoides platessoides	2.3100E+02	3.5	3.3947E+00	FishBase
5790	Ecto.verts	Hippoglossoides platessoides	2.6800E+02	3.5	4.1260E+00	FishBase
5791	Ecto.verts	Hippoglossoides platessoides	2.9400E+02	3.5	3.4976E+00	FishBase
5792	Ecto.verts	Hippoglossoides platessoides	3.4400E+02	3.5	4.0924E+00	FishBase
5793	Ecto.verts	Hippoglossoides platessoides	3.9000E+02	3.5	4.6396E+00	FishBase
5794	Ecto.verts	Hydrodynastes gigas	2.6800E+03	20	8.5760E+01	White et al. 2006
5795	Ecto.verts	Hydromantes sp.	3.2300E+00	15	1.2400E-01	White et al. 2006
5796	Ecto.verts	Hyla arborea	7.7800E+00	18.5	2.4429E+00	White et al. 2006
5797	Ecto.verts	Hyla arenicolor	3.3700E+00	20	3.0000E-01	White et al. 2006
5798	Ecto.verts	Hyla arenicolor	3.4000E+00	20	3.1620E-01	White et al. 2006
5799	Ecto.verts	Hyla chrysoscelis	3.9000E+00	10	1.4820E-01	White et al. 2006
5800	Ecto.verts	Hyla chrysoscelis	3.9000E+00	20	4.3680E-01	White et al. 2006
5801	Ecto.verts	Hyla chrysoscelis	3.9000E+00	30	1.1365E+00	White et al. 2006
5802	Ecto.verts	Hyla chrysoscelis	5.4700E+00	20	7.0560E-01	White et al. 2006
5803	Ecto.verts	Hyla cinerea	3.8200E+00	10	2.1700E-01	White et al. 2006
5804	Ecto.verts	Hyla cinerea	3.8200E+00	20	5.5960E-01	White et al. 2006
5805	Ecto.verts	Hyla cinerea	3.8200E+00	30	8.4880E-01	White et al. 2006
5806	Ecto.verts	Hyla cinerea	4.5000E+00	25	4.5900E-01	White et al. 2006
5807	Ecto.verts	Hyla cinerea	5.1000E+00	27	6.8900E-01	White et al. 2006
5808	Ecto.verts	Hyla cinerea	6.4000E+00	15	8.0970E-01	White et al. 2006

5809	Ecto.verts	Hyla cinerea	8.9000E+00	22	1.5130E+00	White et al. 2006
5810	Ecto.verts	Hyla crepitans	9.9000E+00	25	1.1484E+00	White et al. 2006
5811	Ecto.verts	Hyla crucifer	8.5000E-01	14	1.0370E-01	White et al. 2006
5812	Ecto.verts	Hyla crucifer	1.1500E+00	20	1.2650E-01	White et al. 2006
5813	Ecto.verts	Hyla crucifer	1.3000E+00	20	1.4430E-01	White et al. 2006
5814	Ecto.verts	Hyla crucifer	1.3000E+00	20	1.4430E-01	White et al. 2006
5815	Ecto.verts	Hyla crucifer	1.3100E+00	20	1.4900E-01	White et al. 2006
5816	Ecto.verts	Hyla gratiosa	9.8000E+00	25	1.4431E+00	White et al. 2006
5817	Ecto.verts	Hyla gratiosa	1.0200E+01	5	5.2810E-01	White et al. 2006
5818	Ecto.verts	Hyla gratiosa	1.0200E+01	15	9.0810E-01	White et al. 2006
5819	Ecto.verts	Hyla gratiosa	1.3900E+01	29	1.3210E+00	White et al. 2006
5820	Ecto.verts	Hyla maxima	4.1400E+01	25	3.9330E+00	White et al. 2006
5821	Ecto.verts	Hyla regilla	2.2700E+00	21	8.9000E-02	White et al. 2006
5822	Ecto.verts	Hyla regilla	2.7600E+00	20	1.6560E-01	White et al. 2006
5823	Ecto.verts	Hyla squirella	2.2000E+00	27	3.6300E-01	White et al. 2006
5824	Ecto.verts	Hyla versicolor	6.0900E+00	20	6.0290E-01	White et al. 2006
5825	Ecto.verts	Hyla versicolor	7.6000E+00	25	1.5252E+00	White et al. 2006
5826	Ecto.verts	Hyla versicolor	8.3000E+00	5	5.9970E-01	White et al. 2006
5827	Ecto.verts	Hyla versicolor	8.3000E+00	15	8.6440E-01	White et al. 2006
5828	Ecto.verts	Hyla versicolor	8.6200E+00	19	6.7240E-01	White et al. 2006
5829	Ecto.verts	Hyperolius marmoratus	1.0000E+00	20	6.7100E-02	White et al. 2006
5830	Ecto.verts	Hyperolius parallelus	1.0000E+00	20	7.6500E-02	White et al. 2006
5831	Ecto.verts	Hyperolius tuberilinguis	1.0000E+00	20	6.7900E-02	White et al. 2006
5832	Ecto.verts	Hyperolius viridiflavus	8.8000E-01	20	8.3000E-02	White et al. 2006
5833	Ecto.verts	Hyperolius viridiflavus	9.0000E-01	20	8.4600E-02	White et al. 2006
5834	Ecto.verts	Icelus bicornis	4.7000E+01	-1.5	9.8670E-01	FishBase

5835	Ecto.verts	Icelus spatula	4.7000E+01	-1.5	9.8670E-01	FishBase
5836	Ecto.verts	Ictalurus punctatus	4.4300E+00	23.5	4.6501E-01	FishBase
5837	Ecto.verts	Ictalurus punctatus	5.3700E+00	23.5	1.1574E+00	FishBase
5838	Ecto.verts	Ictalurus punctatus	5.4300E+00	23.5	3.9139E-01	FishBase
5839	Ecto.verts	Ictalurus punctatus	6.7100E+00	23.5	1.2960E+00	FishBase
5840	Ecto.verts	Ictalurus punctatus	7.2400E+00	23.5	8.8157E-01	FishBase
5841	Ecto.verts	Ictalurus punctatus	7.2500E+00	23.5	7.6102E-01	FishBase
5842	Ecto.verts	Ictalurus punctatus	7.6100E+00	23.5	5.4852E-01	FishBase
5843	Ecto.verts	Ictalurus punctatus	8.2700E+00	23.5	1.3253E+00	FishBase
5844	Ecto.verts	Ictalurus punctatus	8.2500E+02	18	2.3670E+01	FishBase
5845	Ecto.verts	Iguana iguana	7.9500E+02	20	3.1800E+01	White et al. 2006
5846	Ecto.verts	Iguana iguana	7.9500E+02	30	6.4395E+01	White et al. 2006
5847	Ecto.verts	Iguana iguana	7.9500E+02	37	1.1925E+02	White et al. 2006
5848	Ecto.verts	Kaloula pulchra	3.0660E+01	20	8.9000E-01	White et al. 2006
5849	Ecto.verts	Kaloula pulchra	3.0700E+01	20	7.9820E-01	White et al. 2006
5850	Ecto.verts	Kassina senegalensis	3.0000E+00	20	2.3100E-01	White et al. 2006
5851	Ecto.verts	Kassina senegalensis	3.0200E+00	20	2.2800E-01	White et al. 2006
5852	Ecto.verts	Kassina weali	6.2500E+00	20	3.1800E-01	White et al. 2006
5853	Ecto.verts	Kassina weali	6.3000E+00	20	3.4020E-01	White et al. 2006
5854	Ecto.verts	Katsuwonus pelamis	6.3200E+02	23.5	9.2876E+01	FishBase
5855	Ecto.verts	Katsuwonus pelamis	1.4700E+03	24	5.4932E+02	FishBase
5856	Ecto.verts	Katsuwonus pelamis	1.5100E+03	24	7.3439E+02	FishBase
5857	Ecto.verts	Katsuwonus pelamis	1.5300E+03	24	6.0279E+02	FishBase
5858	Ecto.verts	Katsuwonus pelamis	1.5500E+03	24	6.3670E+02	FishBase
5859	Ecto.verts	Katsuwonus pelamis	1.6000E+03	24	1.1253E+03	FishBase
5860	Ecto.verts	Katsuwonus pelamis	1.7400E+03	24	9.3393E+02	FishBase

5861	Ecto.verts	Katsuwonus pelamis	3.8340E+03	23.5	8.0490E+02	FishBase
5862	Ecto.verts	Kuhlia sandvicensis	1.3240E+01	23	8.0607E-01	FishBase
5863	Ecto.verts	Kuhlia sandvicensis	1.5670E+01	23	8.6629E-01	FishBase
5864	Ecto.verts	Kuhlia sandvicensis	1.6260E+01	23	8.3064E-01	FishBase
5865	Ecto.verts	Kuhlia sandvicensis	1.9610E+01	23	1.1116E+00	FishBase
5866	Ecto.verts	Kuhlia sandvicensis	2.0550E+01	23	9.9227E-01	FishBase
5867	Ecto.verts	Kuhlia sandvicensis	2.6220E+01	23	1.2477E+00	FishBase
5868	Ecto.verts	Kuhlia sandvicensis	2.7480E+01	23	1.2692E+00	FishBase
5869	Ecto.verts	Kuhlia sandvicensis	2.9060E+01	23	1.4642E+00	FishBase
5870	Ecto.verts	Kuhlia sandvicensis	3.6380E+01	23	1.5784E+00	FishBase
5871	Ecto.verts	Kuhlia sandvicensis	4.6410E+01	23	2.1110E+00	FishBase
5872	Ecto.verts	Kuhlia sandvicensis	4.7000E+01	23	2.1050E+00	FishBase
5873	Ecto.verts	Kuhlia sandvicensis	4.7290E+01	23	2.1841E+00	FishBase
5874	Ecto.verts	Kuhlia sandvicensis	5.5970E+01	23	2.3109E+00	FishBase
5875	Ecto.verts	Labeo rohita	6.0000E+00	25	4.6102E+00	FishBase
5876	Ecto.verts	Labeo rohita	7.0000E+00	25	3.3163E+00	FishBase
5877	Ecto.verts	Labeo rohita	8.5000E+00	25	3.3132E+00	FishBase
5878	Ecto.verts	Labeo rohita	1.1500E+01	25	3.3076E+00	FishBase
5879	Ecto.verts	Labeo rohita	1.1500E+01	25	3.3237E+00	FishBase
5880	Ecto.verts	Labeobarbus aeneus	4.7000E+01	10	1.5458E+00	FishBase
5881	Ecto.verts	Labeobarbus aeneus	4.7000E+01	15	2.3352E+00	FishBase
5882	Ecto.verts	Labeobarbus aeneus	4.7000E+01	20	3.1903E+00	FishBase
5883	Ecto.verts	Labeobarbus aeneus	4.7000E+01	25	4.7362E+00	FishBase
5884	Ecto.verts	Lacerta agilis	8.4000E+00	20	1.5204E+00	White et al. 2006
5885	Ecto.verts	Lacerta agilis	8.4000E+00	30	3.0240E+00	White et al. 2006
5886	Ecto.verts	Lacerta agilis	8.4000E+00	35	4.0908E+00	White et al. 2006

5887	Ecto.verts	Lacerta trilineata	5.4000E+01	30	6.6960E+00	White et al. 2006
5888	Ecto.verts	Lacerta trilineata	7.1000E+01	20	1.6259E+00	White et al. 2006
5889	Ecto.verts	Lacerta viridis	2.0000E+01	30	2.2000E+00	White et al. 2006
5890	Ecto.verts	Lacerta viridis	3.1000E+01	20	8.3700E-01	White et al. 2006
5891	Ecto.verts	Lacerta vivipara	3.1900E+00	5	1.0208E-01	White et al. 2006
5892	Ecto.verts	Lacerta vivipara	3.1900E+00	5	1.6365E-01	White et al. 2006
5893	Ecto.verts	Lacerta vivipara	3.1900E+00	10	2.0416E-01	White et al. 2006
5894	Ecto.verts	Lacerta vivipara	3.1900E+00	10	2.7147E-01	White et al. 2006
5895	Ecto.verts	Lacerta vivipara	3.1900E+00	15	2.8391E-01	White et al. 2006
5896	Ecto.verts	Lacerta vivipara	3.1900E+00	15	3.9333E-01	White et al. 2006
5897	Ecto.verts	Lacerta vivipara	3.1900E+00	20	4.3065E-01	White et al. 2006
5898	Ecto.verts	Lacerta vivipara	3.1900E+00	20	5.5825E-01	White et al. 2006
5899	Ecto.verts	Lacerta vivipara	3.1900E+00	25	7.8155E-01	White et al. 2006
5900	Ecto.verts	Lacerta vivipara	3.1900E+00	25	8.2940E-01	White et al. 2006
5901	Ecto.verts	Lacerta vivipara	3.1900E+00	30	1.0687E+00	White et al. 2006
5902	Ecto.verts	Lacerta vivipara	3.1900E+00	35	1.5950E+00	White et al. 2006
5903	Ecto.verts	Lacerta vivipara	3.9000E+00	35	2.1840E+00	White et al. 2006
5904	Ecto.verts	Lacerta vivipara	3.9500E+00	30	9.3615E-01	White et al. 2006
5905	Ecto.verts	Lampetra fluviatilis	1.4300E+00	4.4	1.6011E-02	FishBase
5906	Ecto.verts	Lampetra fluviatilis	1.4300E+00	4.8	3.5024E-02	FishBase
5907	Ecto.verts	Lampetra fluviatilis	1.4300E+00	10	3.8027E-02	FishBase
5908	Ecto.verts	Lampetra fluviatilis	1.4300E+00	10	4.0028E-02	FishBase
5909	Ecto.verts	Lampetra fluviatilis	1.4300E+00	9.4	4.1029E-02	FishBase
5910	Ecto.verts	Lampetra fluviatilis	1.4300E+00	10	4.4031E-02	FishBase
5911	Ecto.verts	Lampetra fluviatilis	1.4300E+00	10	4.8034E-02	FishBase
5912	Ecto.verts	Lampetra fluviatilis	1.4300E+00	14.4	7.8055E-02	FishBase

5913	Ecto.verts	Lampetra fluviatilis	1.4300E+00	10	8.3058E-02	FishBase
5914	Ecto.verts	Lampetra fluviatilis	1.4300E+00	9.8	8.3058E-02	FishBase
5915	Ecto.verts	Lampetra fluviatilis	1.4300E+00	14.7	1.4010E-01	FishBase
5916	Ecto.verts	Lampetra fluviatilis	2.3300E+01	16	1.8262E+00	FishBase
5917	Ecto.verts	Lampetra fluviatilis	2.8600E+01	16	1.7412E+00	FishBase
5918	Ecto.verts	Lampetra fluviatilis	2.9300E+01	9.5	9.4318E-01	FishBase
5919	Ecto.verts	Lampetra fluviatilis	3.0300E+01	9.5	8.6935E-01	FishBase
5920	Ecto.verts	Lampetra fluviatilis	3.2800E+01	9.5	8.2631E-01	FishBase
5921	Ecto.verts	Lampetra fluviatilis	3.2800E+01	9.5	1.2165E+00	FishBase
5922	Ecto.verts	Lampetra fluviatilis	3.3200E+01	16	1.7657E+00	FishBase
5923	Ecto.verts	Lampetra fluviatilis	3.9400E+01	9.5	1.2132E+00	FishBase
5924	Ecto.verts	Lampetra fluviatilis	4.1200E+01	9.5	1.0379E+00	FishBase
5925	Ecto.verts	Lampetra fluviatilis	4.2200E+01	16	2.3625E+00	FishBase
5926	Ecto.verts	Lampetra fluviatilis	4.2700E+01	9.5	1.1056E+00	FishBase
5927	Ecto.verts	Lampetra fluviatilis	4.3700E+01	9.5	1.1315E+00	FishBase
5928	Ecto.verts	Lampetra fluviatilis	4.4200E+01	9.5	1.0516E+00	FishBase
5929	Ecto.verts	Lampetra fluviatilis	4.5200E+01	16	3.1314E+00	FishBase
5930	Ecto.verts	Lampetra fluviatilis	4.5700E+01	9.5	1.5031E+00	FishBase
5931	Ecto.verts	Lampetra fluviatilis	4.6800E+01	9.5	1.2445E+00	FishBase
5932	Ecto.verts	Lampetra fluviatilis	4.6800E+01	9.5	1.2773E+00	FishBase
5933	Ecto.verts	Lampetra fluviatilis	4.6800E+01	9.5	1.3100E+00	FishBase
5934	Ecto.verts	Lampetra fluviatilis	4.6800E+01	9.5	1.3428E+00	FishBase
5935	Ecto.verts	Lampetra fluviatilis	4.8900E+01	16	3.6615E+00	FishBase
5936	Ecto.verts	Lampetra fluviatilis	4.9500E+01	9.5	1.3163E+00	FishBase
5937	Ecto.verts	Lampetra fluviatilis	4.9500E+01	16	3.5679E+00	FishBase
5938	Ecto.verts	Lampetra fluviatilis	5.0700E+01	16	2.6964E+00	FishBase

5939	Ecto.verts	Lampetra fluviatilis	5.2400E+01	9.5	1.2467E+00	FishBase
5940	Ecto.verts	Lampetra fluviatilis	5.3000E+01	9.5	1.4836E+00	FishBase
5941	Ecto.verts	Lampetra fluviatilis	5.3000E+01	16	3.7089E+00	FishBase
5942	Ecto.verts	Lampetra fluviatilis	5.4200E+01	16	3.1101E+00	FishBase
5943	Ecto.verts	Lampetra fluviatilis	5.5500E+01	9.5	1.6312E+00	FishBase
5944	Ecto.verts	Lampetra fluviatilis	5.5500E+01	9.5	1.8254E+00	FishBase
5945	Ecto.verts	Lampetra fluviatilis	5.6100E+01	9.5	1.3740E+00	FishBase
5946	Ecto.verts	Lampetra fluviatilis	5.6800E+01	9.5	1.5104E+00	FishBase
5947	Ecto.verts	Lampetra fluviatilis	5.6800E+01	9.5	2.0669E+00	FishBase
5948	Ecto.verts	Lampetra fluviatilis	5.8800E+01	9.5	1.5636E+00	FishBase
5949	Ecto.verts	Lampetra fluviatilis	6.2200E+01	9.5	1.9587E+00	FishBase
5950	Ecto.verts	Lampetra fluviatilis	6.3600E+01	9.5	1.5577E+00	FishBase
5951	Ecto.verts	Lampetra planeri	2.7900E+00	5.3	2.5381E-02	FishBase
5952	Ecto.verts	Lampetra planeri	2.7900E+00	10.3	5.4668E-02	FishBase
5953	Ecto.verts	Lampetra planeri	2.7900E+00	10	5.6620E-02	FishBase
5954	Ecto.verts	Lampetra planeri	2.7900E+00	5.7	5.8572E-02	FishBase
5955	Ecto.verts	Lampetra planeri	2.7900E+00	10	6.0525E-02	FishBase
5956	Ecto.verts	Lampetra planeri	2.7900E+00	10	7.0287E-02	FishBase
5957	Ecto.verts	Lampetra planeri	2.7900E+00	10	8.0049E-02	FishBase
5958	Ecto.verts	Lampetra planeri	2.7900E+00	10	8.7859E-02	FishBase
5959	Ecto.verts	Lampetra planeri	2.7900E+00	10	1.0738E-01	FishBase
5960	Ecto.verts	Lampetra planeri	2.7900E+00	15.2	1.1714E-01	FishBase
5961	Ecto.verts	Lampetra planeri	2.7900E+00	10	1.2105E-01	FishBase
5962	Ecto.verts	Lampetra planeri	2.7900E+00	10.6	1.3472E-01	FishBase
5963	Ecto.verts	Lampetra planeri	2.7900E+00	10	1.3862E-01	FishBase
5964	Ecto.verts	Lampetra planeri	2.7900E+00	10	1.4057E-01	FishBase

5965	Ecto.verts	Lampetra planeri	2.7900E+00	10	1.6010E-01	FishBase
5966	Ecto.verts	Lampetra planeri	2.7900E+00	10	1.9719E-01	FishBase
5967	Ecto.verts	Lampetra planeri	2.7900E+00	15.6	2.0110E-01	FishBase
5968	Ecto.verts	Lampropeltis getulus	1.2170E+03	26	3.4076E+01	White et al. 2006
5969	Ecto.verts	Lampropeltis miliaris	4.0100E+02	20	1.3634E+01	White et al. 2006
5970	Ecto.verts	Lampropeltis miliaris	4.0100E+02	25	1.9248E+01	White et al. 2006
5971	Ecto.verts	Lampropeltis miliaris	4.0100E+02	30	3.1278E+01	White et al. 2006
5972	Ecto.verts	Lepidobatrachus llanensis	8.8500E+01	25	8.5845E+00	White et al. 2006
5973	Ecto.verts	Lepidophyma gaigeae	5.0000E+00	15	1.3500E-01	White et al. 2006
5974	Ecto.verts	Lepidophyma gaigeae	5.0000E+00	20	3.0750E-01	White et al. 2006
5975	Ecto.verts	Lepidophyma gaigeae	5.0000E+00	25	3.9000E-01	White et al. 2006
5976	Ecto.verts	Lepidophyma gaigeae	5.0000E+00	30	6.5000E-01	White et al. 2006
5977	Ecto.verts	Lepidophyma smithi	2.5000E+01	20	7.7250E-01	White et al. 2006
5978	Ecto.verts	Lepidophyma smithi	2.5000E+01	25	1.2500E+00	White et al. 2006
5979	Ecto.verts	Lepidophyma smithi	2.5000E+01	30	1.7500E+00	White et al. 2006
5980	Ecto.verts	Lepomis gibbosus	4.4920E+01	20	1.4146E+00	FishBase
5981	Ecto.verts	Lepomis macrochirus	1.1800E+02	25	8.3401E+00	FishBase
5982	Ecto.verts	Lepomis macrochirus	1.1800E+02	25	8.5052E+00	FishBase
5983	Ecto.verts	Lepomis macrochirus	1.1800E+02	25	8.7530E+00	FishBase
5984	Ecto.verts	Lepomis macrochirus	1.1800E+02	25	8.8355E+00	FishBase
5985	Ecto.verts	Leporinus fasciatus	3.9500E+00	25	3.8422E-01	FishBase
5986	Ecto.verts	Leporinus fasciatus	7.6000E+00	30	8.4563E-01	FishBase
5987	Ecto.verts	Leptodactylus typhonius	5.1000E+00	25	5.8650E-01	White et al. 2006
5988	Ecto.verts	Leuciscus idus	6.0000E+02	5	7.9776E+00	FishBase
5989	Ecto.verts	Leuciscus idus	6.0000E+02	10	1.5955E+01	FishBase
5990	Ecto.verts	Liasis fuscus	1.3069E+03	24	1.8297E+01	White et al. 2006

5991	Ecto.verts	Liasis fuscus	1.3069E+03	27	2.3263E+01	White et al. 2006
5992	Ecto.verts	Liasis fuscus	1.3069E+03	30	2.8752E+01	White et al. 2006
5993	Ecto.verts	Liasis fuscus	1.3069E+03	33	5.2276E+01	White et al. 2006
5994	Ecto.verts	Liasis olivaceus	3.0007E+03	24	4.5011E+01	White et al. 2006
5995	Ecto.verts	Liasis olivaceus	3.0007E+03	27	7.5018E+01	White et al. 2006
5996	Ecto.verts	Liasis olivaceus	3.0007E+03	33	1.0202E+02	White et al. 2006
5997	Ecto.verts	Liasis olivaceus	3.3232E+03	30	1.0967E+02	White et al. 2006
5998	Ecto.verts	Lichanura roseofusca	3.1400E+02	32	2.1980E+01	White et al. 2006
5999	Ecto.verts	Lichanura roseofusca	3.1400E+02	32	2.0410E+02	White et al. 2006
6000	Ecto.verts	Lichanura trivirgata	1.8200E+02	20	2.3792E+00	White et al. 2006
6001	Ecto.verts	Lichanura trivirgata	1.8200E+02	30	6.0594E+00	White et al. 2006
6002	Ecto.verts	Lichanura trivirgata	1.8200E+02	34	1.0088E+01	White et al. 2006
6003	Ecto.verts	Limanda limanda	4.7000E+01	10	5.8216E+00	FishBase
6004	Ecto.verts	Limanda limanda	2.3600E+02	10	4.7894E+00	FishBase
6005	Ecto.verts	Limanda limanda	3.9600E+02	15	1.1639E+01	FishBase
6006	Ecto.verts	Limanda limanda	4.0000E+02	5	4.1987E+00	FishBase
6007	Ecto.verts	Lindbergichthys nudifrons	4.7000E+01	3	2.4010E+00	FishBase
6008	Ecto.verts	Liophis poecilogyrus	4.2000E+01	20	1.6800E+00	White et al. 2006
6009	Ecto.verts	Liparis atlanticus	4.7000E+01	-1.5	3.9468E-01	FishBase
6010	Ecto.verts	Liparis fabricii	4.7000E+01	-1.5	3.9468E-01	FishBase
6011	Ecto.verts	Lipophrys pholis	3.0000E-02	16	1.5220E-02	FishBase
6012	Ecto.verts	Lipophrys pholis	6.0000E-02	16	2.5612E-02	FishBase
6013	Ecto.verts	Lipophrys pholis	6.0000E-02	16	3.0231E-02	FishBase
6014	Ecto.verts	Lipophrys pholis	1.8000E-01	16	9.4724E-02	FishBase
6015	Ecto.verts	Lipophrys pholis	2.0000E-01	16	6.0462E-02	FishBase
6016	Ecto.verts	Lipophrys pholis	2.0000E-01	16	6.0882E-02	FishBase

6017	Ecto.verts	Lipophrys pholis	2.0000E-01	16	6.8160E-02	FishBase
6018	Ecto.verts	Lipophrys pholis	2.0000E-01	16	9.0273E-02	FishBase
6019	Ecto.verts	Lipophrys pholis	2.2000E-01	16	1.1747E-01	FishBase
6020	Ecto.verts	Lipophrys pholis	2.3000E-01	16	7.3394E-02	FishBase
6021	Ecto.verts	Lipophrys pholis	2.3000E-01	16	8.6592E-02	FishBase
6022	Ecto.verts	Lipophrys pholis	2.3000E-01	16	9.6732E-02	FishBase
6023	Ecto.verts	Lipophrys pholis	2.3000E-01	16	1.1428E-01	FishBase
6024	Ecto.verts	Lipophrys pholis	2.3000E-01	16	1.2087E-01	FishBase
6025	Ecto.verts	Lipophrys pholis	2.4000E-01	16	7.2386E-02	FishBase
6026	Ecto.verts	Lipophrys pholis	2.5000E-01	16	8.9048E-02	FishBase
6027	Ecto.verts	Lipophrys pholis	2.7000E-01	16	8.1246E-02	FishBase
6028	Ecto.verts	Lipophrys pholis	2.7000E-01	16	1.0146E-01	FishBase
6029	Ecto.verts	Lipophrys pholis	2.8000E-01	16	8.4255E-02	FishBase
6030	Ecto.verts	Lipophrys pholis	3.5000E-01	16	9.4542E-02	FishBase
6031	Ecto.verts	Lipophrys pholis	3.8000E-01	16	7.3128E-02	FishBase
6032	Ecto.verts	Lipophrys pholis	3.8000E-01	16	7.7383E-02	FishBase
6033	Ecto.verts	Lipophrys pholis	4.1000E-01	16	9.3247E-02	FishBase
6034	Ecto.verts	Lipophrys pholis	5.9000E-01	16	1.7630E-01	FishBase
6035	Ecto.verts	Lipophrys pholis	6.3000E-01	16	2.1029E-01	FishBase
6036	Ecto.verts	Lipophrys pholis	6.6000E-01	16	1.4918E-01	FishBase
6037	Ecto.verts	Lipophrys pholis	6.6000E-01	16	1.7643E-01	FishBase
6038	Ecto.verts	Lipophrys pholis	6.6000E-01	16	2.3278E-01	FishBase
6039	Ecto.verts	Lipophrys pholis	7.0000E-01	16	2.0868E-01	FishBase
6040	Ecto.verts	Lipophrys pholis	7.3000E-01	16	1.1136E-01	FishBase
6041	Ecto.verts	Lipophrys pholis	7.8000E-01	16	1.6648E-01	FishBase
6042	Ecto.verts	Lipophrys pholis	7.9000E-01	16	1.5092E-01	FishBase

6043	Ecto.verts	Lipophrys pholis	8.2000E-01	16	1.3944E-01	FishBase
6044	Ecto.verts	Lipophrys pholis	9.3000E-01	16	1.9850E-01	FishBase
6045	Ecto.verts	Lipophrys pholis	9.3000E-01	16	2.3429E-01	FishBase
6046	Ecto.verts	Lipophrys pholis	9.8000E-01	16	1.5705E-01	FishBase
6047	Ecto.verts	Lipophrys pholis	1.3800E+00	16	4.8286E-01	FishBase
6048	Ecto.verts	Lipophrys pholis	1.5400E+00	16	1.7566E-01	FishBase
6049	Ecto.verts	Lipophrys pholis	1.5500E+00	16	3.4710E-01	FishBase
6050	Ecto.verts	Lipophrys pholis	1.5500E+00	16	4.8485E-01	FishBase
6051	Ecto.verts	Lipophrys pholis	1.5500E+00	16	7.5602E-01	FishBase
6052	Ecto.verts	Lipophrys pholis	1.5600E+00	16	4.1156E-01	FishBase
6053	Ecto.verts	Lipophrys pholis	1.6300E+00	16	2.3383E-01	FishBase
6054	Ecto.verts	Lipophrys pholis	1.6300E+00	16	2.7490E-01	FishBase
6055	Ecto.verts	Lipophrys pholis	1.6300E+00	16	2.9087E-01	FishBase
6056	Ecto.verts	Lipophrys pholis	1.7300E+00	16	2.4576E-01	FishBase
6057	Ecto.verts	Lipophrys pholis	1.7300E+00	16	3.6682E-01	FishBase
6058	Ecto.verts	Lipophrys pholis	2.4200E+00	16	4.8434E-01	FishBase
6059	Ecto.verts	Lipophrys pholis	2.7100E+00	16	4.5704E-01	FishBase
6060	Ecto.verts	Lipophrys pholis	2.8400E+00	16	4.2729E-01	FishBase
6061	Ecto.verts	Lipophrys pholis	2.8400E+00	16	4.6307E-01	FishBase
6062	Ecto.verts	Lipophrys pholis	3.0300E+00	16	4.5800E-01	FishBase
6063	Ecto.verts	Lipophrys pholis	3.0300E+00	16	4.8344E-01	FishBase
6064	Ecto.verts	Lipophrys pholis	3.0600E+00	16	3.8544E-01	FishBase
6065	Ecto.verts	Lipophrys pholis	3.0800E+00	16	6.1212E-01	FishBase
6066	Ecto.verts	Lipophrys pholis	3.3400E+00	16	7.7832E-01	FishBase
6067	Ecto.verts	Lipophrys pholis	3.9200E+00	16	5.6509E-01	FishBase
6068	Ecto.verts	Lipophrys pholis	4.2500E+00	16	8.4465E-01	FishBase

6069	Ecto.verts	Lipophrys pholis	4.4900E+00	16	7.5409E-01	FishBase
6070	Ecto.verts	Lipophrys pholis	4.5700E+00	16	4.5732E-01	FishBase
6071	Ecto.verts	Lipophrys pholis	4.7500E+00	16	7.5455E-01	FishBase
6072	Ecto.verts	Lipophrys pholis	4.7500E+00	16	8.4430E-01	FishBase
6073	Ecto.verts	Lipophrys pholis	5.1200E+00	16	5.7327E-01	FishBase
6074	Ecto.verts	Lipophrys pholis	5.3100E+00	16	1.1110E+00	FishBase
6075	Ecto.verts	Lipophrys pholis	5.6200E+00	16	7.1577E-01	FishBase
6076	Ecto.verts	Lipophrys pholis	5.7400E+00	16	5.1013E-01	FishBase
6077	Ecto.verts	Lipophrys pholis	5.8500E+00	16	9.8660E-01	FishBase
6078	Ecto.verts	Lipophrys pholis	5.9500E+00	16	5.7460E-01	FishBase
6079	Ecto.verts	Lipophrys pholis	5.9500E+00	16	9.9930E-01	FishBase
6080	Ecto.verts	Lipophrys pholis	6.0800E+00	16	4.8504E-01	FishBase
6081	Ecto.verts	Lipophrys pholis	6.2900E+00	16	7.1307E-01	FishBase
6082	Ecto.verts	Lipophrys pholis	6.2900E+00	16	7.9670E-01	FishBase
6083	Ecto.verts	Lipophrys pholis	6.2900E+00	16	8.4512E-01	FishBase
6084	Ecto.verts	Lipophrys pholis	6.2900E+00	16	9.4196E-01	FishBase
6085	Ecto.verts	Lipophrys pholis	6.2900E+00	16	1.0520E+00	FishBase
6086	Ecto.verts	Lipophrys pholis	6.5900E+00	16	9.8689E-01	FishBase
6087	Ecto.verts	Lipophrys pholis	6.6500E+00	16	1.4705E+00	FishBase
6088	Ecto.verts	Lipophrys pholis	6.8600E+00	16	1.2529E+00	FishBase
6089	Ecto.verts	Lipophrys pholis	7.2200E+00	16	6.4167E-01	FishBase
6090	Ecto.verts	Lipophrys pholis	7.2200E+00	16	7.5787E-01	FishBase
6091	Ecto.verts	Lipophrys pholis	7.4400E+00	16	7.7576E-01	FishBase
6092	Ecto.verts	Lipophrys pholis	7.6400E+00	16	6.7899E-01	FishBase
6093	Ecto.verts	Lipophrys pholis	7.6400E+00	16	8.0196E-01	FishBase
6094	Ecto.verts	Lipophrys pholis	7.8700E+00	16	1.2447E+00	FishBase

6095	Ecto.verts	Lipophrys pholis	8.3200E+00	16	9.9561E-01	FishBase
6096	Ecto.verts	Lipophrys pholis	9.3100E+00	16	1.1141E+00	FishBase
6097	Ecto.verts	Lipophrys pholis	9.6000E+00	16	9.0021E-01	FishBase
6098	Ecto.verts	Lipophrys pholis	1.2300E+01	16	1.3858E+00	FishBase
6099	Ecto.verts	Lipophrys pholis	1.3000E+01	16	1.3100E+00	FishBase
6100	Ecto.verts	Lipophrys pholis	1.3000E+01	16	1.4647E+00	FishBase
6101	Ecto.verts	Lipophrys pholis	1.4300E+01	16	9.5066E-01	FishBase
6102	Ecto.verts	Lipophrys pholis	1.6600E+01	16	2.0213E+00	FishBase
6103	Ecto.verts	Lipophrys pholis	1.8700E+01	16	1.7928E+00	FishBase
6104	Ecto.verts	Lipophrys pholis	1.8700E+01	16	2.0153E+00	FishBase
6105	Ecto.verts	Lipophrys pholis	2.0200E+01	16	1.2581E+00	FishBase
6106	Ecto.verts	Lipophrys pholis	2.0400E+01	16	1.9415E+00	FishBase
6107	Ecto.verts	Lipophrys pholis	2.1600E+01	16	2.0557E+00	FishBase
6108	Ecto.verts	Lipophrys pholis	2.2600E+01	16	1.5815E+00	FishBase
6109	Ecto.verts	Lipophrys pholis	2.2800E+01	16	1.9306E+00	FishBase
6110	Ecto.verts	Litoria platycephala	1.1000E+01	15	5.9070E-01	White et al. 2006
6111	Ecto.verts	Liza dumerili	4.7000E+01	18	3.3548E+00	FishBase
6112	Ecto.verts	Liza dumerili	4.7000E+01	18	3.3877E+00	FishBase
6113	Ecto.verts	Liza dumerili	4.7000E+01	23	5.6242E+00	FishBase
6114	Ecto.verts	Liza dumerili	4.7000E+01	23	5.9531E+00	FishBase
6115	Ecto.verts	Liza richardsonii	4.7000E+01	18	4.3415E+00	FishBase
6116	Ecto.verts	Liza richardsonii	4.7000E+01	23	5.9860E+00	FishBase
6117	Ecto.verts	Lumpenus lampretaeformis	4.7000E+00	15	2.9930E-01	FishBase
6118	Ecto.verts	Lumpenus lampretaeformis	5.6000E+00	15	2.9783E-01	FishBase
6119	Ecto.verts	Lumpenus lampretaeformis	9.1000E+00	15	6.3044E-01	FishBase
6120	Ecto.verts	Lumpenus lampretaeformis	9.5000E+00	15	5.2519E-01	FishBase

6121	Ecto.verts	Lumpenus lampretaeformis	1.0200E+01	15	7.4234E-01	FishBase
6122	Ecto.verts	Lumpenus lampretaeformis	1.3200E+01	15	8.3135E-01	FishBase
6123	Ecto.verts	Lumpenus lampretaeformis	1.4300E+01	15	6.7047E-01	FishBase
6124	Ecto.verts	Lycodes eudipleurostictus	4.7000E+01	0	3.9468E-01	FishBase
6125	Ecto.verts	Lycodes mucosus	4.7000E+01	-1.5	5.9202E-01	FishBase
6126	Ecto.verts	Lycodes pallidus	4.7000E+01	0	2.9601E-01	FishBase
6127	Ecto.verts	Lycodes reticulatus	4.7000E+01	0	8.2225E-01	FishBase
6128	Ecto.verts	Lycodes seminudus	4.7000E+01	0	4.9335E-01	FishBase
6129	Ecto.verts	Lycodes turneri	4.7000E+01	-1.5	5.9202E-01	FishBase
6130	Ecto.verts	Macrognathus aculeatus	5.0000E+00	21	4.0238E-01	FishBase
6131	Ecto.verts	Macrognathus aculeatus	6.0000E+00	30	7.0539E-01	FishBase
6132	Ecto.verts	Macrognathus aculeatus	1.1000E+01	21	6.8509E-01	FishBase
6133	Ecto.verts	Macrognathus aculeatus	1.2880E+01	30	1.1898E+00	FishBase
6134	Ecto.verts	Macrognathus aculeatus	2.3500E+01	21	1.4472E+00	FishBase
6135	Ecto.verts	Macrognathus aculeatus	2.6170E+01	30	2.0694E+00	FishBase
6136	Ecto.verts	Macrognathus aculeatus	3.5000E+01	30	2.6207E+00	FishBase
6137	Ecto.verts	Macrognathus aculeatus	3.7000E+01	21	1.7607E+00	FishBase
6138	Ecto.verts	Macrognathus aculeatus	4.2500E+01	30	2.8551E+00	FishBase
6139	Ecto.verts	Macrognathus aculeatus	4.5500E+01	21	2.0059E+00	FishBase
6140	Ecto.verts	Macrognathus aculeatus	5.3000E+01	21	1.8174E+00	FishBase
6141	Ecto.verts	Macrognathus aculeatus	5.3500E+01	30	3.2946E+00	FishBase
6142	Ecto.verts	Masticodryas bifossatus	7.3500E+02	20	3.1605E+01	White et al. 2006
6143	Ecto.verts	Masticophis flagellum	2.6200E+02	35	2.2270E+02	White et al. 2006
6144	Ecto.verts	Melanogrammus aeglefinus	1.5590E+02	10	3.7093E+00	FishBase
6145	Ecto.verts	Melanogrammus aeglefinus	1.5590E+02	10	4.3639E+00	FishBase
6146	Ecto.verts	Melanogrammus aeglefinus	1.5590E+02	10	5.5640E+00	FishBase

6147	Ecto.verts	Melanogrammus aeglefinus	1.5590E+02	10	6.7640E+00	FishBase
6148	Ecto.verts	Melanogrammus aeglefinus	1.5590E+02	10	7.5277E+00	FishBase
6149	Ecto.verts	Melanogrammus aeglefinus	1.5600E+02	10	6.5500E+00	FishBase
6150	Ecto.verts	Microhyla carolinensis	3.5000E+00	15	2.3150E-01	White et al. 2006
6151	Ecto.verts	Micropterus salmoides	1.7800E+02	15	2.2421E+00	FishBase
6152	Ecto.verts	Micropterus salmoides	1.7800E+02	30	4.3597E+00	FishBase
6153	Ecto.verts	Micropterus salmoides	3.5000E+02	20	1.6655E+01	FishBase
6154	Ecto.verts	Micropterus salmoides	3.5000E+02	25	2.5227E+01	FishBase
6155	Ecto.verts	Micropterus salmoides	3.5000E+02	25	2.5717E+01	FishBase
6156	Ecto.verts	Micropterus salmoides	3.5000E+02	30	3.6984E+01	FishBase
6157	Ecto.verts	Micropterus salmoides	3.5000E+02	30	4.2372E+01	FishBase
6158	Ecto.verts	Microstomus kitt	2.2100E+02	15	8.0420E+00	FishBase
6159	Ecto.verts	Microstomus kitt	2.2900E+02	5	4.4871E+00	FishBase
6160	Ecto.verts	Microstomus kitt	2.4900E+02	10	5.7502E+00	FishBase
6161	Ecto.verts	Molga torosa	1.7500E+01	18.5	2.7300E+00	White et al. 2006
6162	Ecto.verts	Monopterus cuchia	1.9800E+00	25	1.3690E+00	FishBase
6163	Ecto.verts	Monopterus cuchia	2.9600E+00	25	1.3133E+00	FishBase
6164	Ecto.verts	Monopterus cuchia	7.7900E+00	25	1.7444E+00	FishBase
6165	Ecto.verts	Monopterus cuchia	2.8800E+01	25	2.4789E+00	FishBase
6166	Ecto.verts	Monopterus cuchia	5.3800E+01	25	3.0495E+00	FishBase
6167	Ecto.verts	Monopterus cuchia	5.9500E+01	25	3.2061E+00	FishBase
6168	Ecto.verts	Monopterus cuchia	6.0700E+01	25	2.5911E+00	FishBase
6169	Ecto.verts	Monopterus cuchia	8.3760E+01	25	2.8721E+00	FishBase
6170	Ecto.verts	Monopterus cuchia	1.6606E+02	25	3.3700E+00	FishBase
6171	Ecto.verts	Monopterus cuchia	1.7288E+02	25	3.5084E+00	FishBase
6172	Ecto.verts	Morelia spilota	1.0655E+03	20	1.2424E+01	White et al. 2006

6173	Ecto.verts	Morelia spilota	1.0655E+03	30	3.8371E+01	White et al. 2006
6174	Ecto.verts	Morelia spilota	1.0655E+03	34	5.2160E+01	White et al. 2006
6175	Ecto.verts	Morelia spilota spilota	1.5165E+03	24	4.2462E+01	White et al. 2006
6176	Ecto.verts	Morelia spilota spilota	1.5165E+03	27	6.0660E+01	White et al. 2006
6177	Ecto.verts	Morelia spilota spilota	1.5165E+03	33	7.8858E+01	White et al. 2006
6178	Ecto.verts	Morelia spilota spilota	1.5165E+03	30	8.0375E+01	White et al. 2006
6179	Ecto.verts	Morelia spilota variegata	1.9384E+03	24	5.0398E+01	White et al. 2006
6180	Ecto.verts	Morelia spilota variegata	2.1700E+03	27	6.2930E+01	White et al. 2006
6181	Ecto.verts	Morelia spilota variegata	2.1735E+03	30	5.6511E+01	White et al. 2006
6182	Ecto.verts	Morelia spilota variegata	2.8287E+03	33	1.2446E+02	White et al. 2006
6183	Ecto.verts	Mugil cephalus	4.7000E+01	18	3.3548E+00	FishBase
6184	Ecto.verts	Mugil cephalus	4.7000E+01	18	4.6704E+00	FishBase
6185	Ecto.verts	Mugil cephalus	4.7000E+01	23	5.9202E+00	FishBase
6186	Ecto.verts	Mugil cephalus	4.7000E+01	23	6.1833E+00	FishBase
6187	Ecto.verts	Mugil cephalus	7.9000E+01	16	4.8649E+00	FishBase
6188	Ecto.verts	Mugil cephalus	1.0000E+02	20.8	1.4486E+01	FishBase
6189	Ecto.verts	Mugil cephalus	1.0200E+02	21.5	1.3134E+01	FishBase
6190	Ecto.verts	Mugil cephalus	1.1000E+02	21	9.5451E+00	FishBase
6191	Ecto.verts	Mugil cephalus	1.1500E+02	15.4	4.9895E+00	FishBase
6192	Ecto.verts	Mugil cephalus	1.1600E+02	19.3	1.1933E+01	FishBase
6193	Ecto.verts	Mugil cephalus	1.4000E+02	20	9.6991E+00	FishBase
6194	Ecto.verts	Mugil cephalus	1.4000E+02	20	1.3128E+01	FishBase
6195	Ecto.verts	Mugil cephalus	1.4000E+02	20	1.5675E+01	FishBase
6196	Ecto.verts	Mugil cephalus	1.4000E+02	20	1.5871E+01	FishBase
6197	Ecto.verts	Mugil cephalus	2.1000E+02	28.5	3.1449E+01	FishBase
6198	Ecto.verts	Mugil cephalus	2.1400E+02	28.5	2.9502E+01	FishBase

6199	Ecto.verts	Mugil cephalus	2.1900E+02	28.5	3.2030E+01	FishBase
6200	Ecto.verts	Mugil cephalus	2.2100E+02	14.5	7.8873E+00	FishBase
6201	Ecto.verts	Mugil cephalus	2.2500E+02	14.5	1.1494E+01	FishBase
6202	Ecto.verts	Mugil cephalus	2.2500E+02	28.5	3.0546E+01	FishBase
6203	Ecto.verts	Mugil cephalus	2.3800E+02	14.5	8.8272E+00	FishBase
6204	Ecto.verts	Mugil cephalus	2.5900E+02	14.5	1.2868E+01	FishBase
6205	Ecto.verts	Mugil curema	1.4000E+02	20	9.7971E+00	FishBase
6206	Ecto.verts	Mugil curema	1.4000E+02	20	1.0679E+01	FishBase
6207	Ecto.verts	Mugil curema	1.4000E+02	20	1.4108E+01	FishBase
6208	Ecto.verts	Mugil curema	1.4000E+02	20	1.5773E+01	FishBase
6209	Ecto.verts	Myoxocephalus scorpius	4.7000E+01	-1.5	1.6774E+00	FishBase
6210	Ecto.verts	Mystus cavasius	4.0000E+00	29	2.0042E+00	FishBase
6211	Ecto.verts	Mystus cavasius	7.0000E+00	29	1.6312E+00	FishBase
6212	Ecto.verts	Mystus cavasius	9.0000E+00	29	2.5066E+00	FishBase
6213	Ecto.verts	Mystus cavasius	1.4000E+01	29	3.0077E+00	FishBase
6214	Ecto.verts	Mystus cavasius	1.5000E+01	29	2.7292E+00	FishBase
6215	Ecto.verts	Mystus cavasius	1.9000E+01	29	2.7922E+00	FishBase
6216	Ecto.verts	Mystus cavasius	2.4000E+01	29	3.2414E+00	FishBase
6217	Ecto.verts	Mystus cavasius	2.5000E+01	29	3.9188E+00	FishBase
6218	Ecto.verts	Mystus cavasius	3.3000E+01	29	5.4269E+00	FishBase
6219	Ecto.verts	Mystus cavasius	3.5000E+01	29	5.9517E+00	FishBase
6220	Ecto.verts	Mystus cavasius	3.7000E+01	29	5.7999E+00	FishBase
6221	Ecto.verts	Mystus cavasius	4.2000E+01	29	8.5528E+00	FishBase
6222	Ecto.verts	Mystus cavasius	4.5000E+01	29	5.6998E+00	FishBase
6223	Ecto.verts	Mystus cavasius	4.7000E+01	29	6.9069E+00	FishBase
6224	Ecto.verts	Mystus cavasius	4.7000E+01	29	7.1043E+00	FishBase

6225	Ecto.verts	Mystus cavasius	5.2000E+01	29	7.5325E+00	FishBase
6226	Ecto.verts	Mystus cavasius	5.3000E+01	29	8.8272E+00	FishBase
6227	Ecto.verts	Mystus cavasius	5.6000E+01	29	9.6011E+00	FishBase
6228	Ecto.verts	Myxine glutinosa	3.8400E+01	7	1.2899E+00	FishBase
6229	Ecto.verts	Myxine glutinosa	3.8400E+01	15	1.8542E+00	FishBase
6230	Ecto.verts	Natrix maura	2.2500E+01	5	1.4463E-01	White et al. 2006
6231	Ecto.verts	Natrix maura	2.2500E+01	15	4.0290E-01	White et al. 2006
6232	Ecto.verts	Natrix maura	2.2500E+01	20	6.6118E-01	White et al. 2006
6233	Ecto.verts	Natrix maura	2.2500E+01	25	1.0331E+00	White et al. 2006
6234	Ecto.verts	Natrix maura	2.2500E+01	30	1.4463E+00	White et al. 2006
6235	Ecto.verts	Natrix maura	2.2500E+01	35	2.7893E+00	White et al. 2006
6236	Ecto.verts	Natrix maura	2.2500E+01	10	6.3845E+00	White et al. 2006
6237	Ecto.verts	Natrix natrix	8.4000E+01	20	7.5600E+00	White et al. 2006
6238	Ecto.verts	Natrix natrix helretica	8.2500E+01	5	3.2849E-01	White et al. 2006
6239	Ecto.verts	Natrix natrix helretica	8.2500E+01	10	1.2318E+00	White et al. 2006
6240	Ecto.verts	Natrix natrix helretica	8.2500E+01	15	1.8067E+00	White et al. 2006
6241	Ecto.verts	Natrix natrix helretica	8.2500E+01	20	3.5586E+00	White et al. 2006
6242	Ecto.verts	Natrix natrix helretica	8.2500E+01	25	5.7486E+00	White et al. 2006
6243	Ecto.verts	Natrix natrix helretica	8.2500E+01	30	1.1223E+01	White et al. 2006
6244	Ecto.verts	Natrix natrix helretica	8.2500E+01	35	1.6151E+01	White et al. 2006
6245	Ecto.verts	Natrix natrix persa	3.7500E+01	5	2.5762E-01	White et al. 2006
6246	Ecto.verts	Natrix natrix persa	3.7500E+01	10	3.4854E-01	White et al. 2006
6247	Ecto.verts	Natrix natrix persa	3.7500E+01	15	6.5162E-01	White et al. 2006
6248	Ecto.verts	Natrix natrix persa	3.7500E+01	20	1.1820E+00	White et al. 2006
6249	Ecto.verts	Natrix natrix persa	3.7500E+01	25	1.8185E+00	White et al. 2006
6250	Ecto.verts	Natrix natrix persa	3.7500E+01	30	2.7277E+00	White et al. 2006

6251	Ecto.verts	Natrix natrix persa	3.7500E+01	35	5.1523E+00	White et al. 2006
6252	Ecto.verts	Necturus maculosus	1.0190E+02	5	5.0950E-01	White et al. 2006
6253	Ecto.verts	Necturus maculosus	1.0190E+02	15	1.2228E+00	White et al. 2006
6254	Ecto.verts	Necturus maculosus	1.1450E+02	22	3.1488E+00	White et al. 2006
6255	Ecto.verts	Necturus maculosus	1.1500E+02	21.5	3.8956E+00	White et al. 2006
6256	Ecto.verts	Necturus maculosus	1.2500E+02	25	2.4750E+00	White et al. 2006
6257	Ecto.verts	Necturus maculosus	1.5000E+02	20	7.2150E+00	White et al. 2006
6258	Ecto.verts	Necturus maculosus	2.0600E+02	15	2.3031E+00	White et al. 2006
6259	Ecto.verts	Necturus maculosus	2.1200E+02	25	5.6222E+00	White et al. 2006
6260	Ecto.verts	Necturus maculosus	2.1400E+02	5	1.1684E+00	White et al. 2006
6261	Ecto.verts	Nerodia rhombifera	2.3800E+02	20	1.4280E+01	White et al. 2006
6262	Ecto.verts	Nerodia rhombifera	2.3800E+02	30	2.2610E+01	White et al. 2006
6263	Ecto.verts	Notophthalmus viridescens	1.2300E+00	15	7.5200E-02	White et al. 2006
6264	Ecto.verts	Notophthalmus viridescens	1.3100E+00	20	1.5330E-01	White et al. 2006
6265	Ecto.verts	Notophthalmus viridescens	1.3500E+00	25	1.2500E-01	White et al. 2006
6266	Ecto.verts	Notophthalmus viridescens	1.5000E+00	25	1.5320E-01	White et al. 2006
6267	Ecto.verts	Notophthalmus viridescens	1.6300E+00	5	8.9800E-02	White et al. 2006
6268	Ecto.verts	Notophthalmus viridescens	1.7000E+00	15	1.3650E-01	White et al. 2006
6269	Ecto.verts	Notophthalmus viridescens	1.7200E+00	15	5.9200E-02	White et al. 2006
6270	Ecto.verts	Notophthalmus viridescens	2.4300E+00	10	1.9930E-01	White et al. 2006
6271	Ecto.verts	Notophthalmus viridescens	3.0000E+00	20	1.3500E-01	White et al. 2006
6272	Ecto.verts	Notothenia neglecta	4.7000E+01	0	9.8670E-01	FishBase
6273	Ecto.verts	Notothenia neglecta	4.7000E+01	0.6	3.3877E+00	FishBase
6274	Ecto.verts	Notothenia rossii	4.7000E+01	0.6	1.4143E+00	FishBase
6275	Ecto.verts	Notothenia rossii	4.7000E+01	3	2.7957E+00	FishBase
6276	Ecto.verts	Occidozyga martensii	9.3000E+00	15	4.1670E-01	White et al. 2006

6277	Ecto.verts	Occidozyga martensii	9.3000E+00	20	5.2400E-01	White et al. 2006
6278	Ecto.verts	Occidozyga martensii	9.3000E+00	25	8.8720E-01	White et al. 2006
6279	Ecto.verts	Occidozyga martensii	9.3000E+00	30	9.4020E-01	White et al. 2006
6280	Ecto.verts	Occidozyga martensii	9.3000E+00	35	1.0779E+00	White et al. 2006
6281	Ecto.verts	Odontophrynus americanus	1.5200E+01	20	5.6240E-01	White et al. 2006
6282	Ecto.verts	Odontophrynus americanus	1.5240E+01	20	5.4900E-01	White et al. 2006
6283	Ecto.verts	Oncorhynchus mykiss	4.7000E+01	5	2.2036E+00	FishBase
6284	Ecto.verts	Oncorhynchus mykiss	4.7000E+01	15	4.3744E+00	FishBase
6285	Ecto.verts	Oncorhynchus mykiss	4.7000E+01	11	4.7691E+00	FishBase
6286	Ecto.verts	Oncorhynchus mykiss	1.0000E+02	15	3.4990E+00	FishBase
6287	Ecto.verts	Oncorhynchus mykiss	1.0000E+02	5	3.7789E+00	FishBase
6288	Ecto.verts	Oncorhynchus mykiss	1.0000E+02	5	3.8488E+00	FishBase
6289	Ecto.verts	Oncorhynchus mykiss	1.0000E+02	15	3.9888E+00	FishBase
6290	Ecto.verts	Oncorhynchus mykiss	1.0000E+02	5	3.9888E+00	FishBase
6291	Ecto.verts	Oncorhynchus mykiss	1.0000E+02	5	4.1288E+00	FishBase
6292	Ecto.verts	Oncorhynchus mykiss	1.0000E+02	5	4.1987E+00	FishBase
6293	Ecto.verts	Oncorhynchus mykiss	1.0000E+02	5	4.3387E+00	FishBase
6294	Ecto.verts	Oncorhynchus mykiss	1.0000E+02	15	4.4787E+00	FishBase
6295	Ecto.verts	Oncorhynchus mykiss	1.0000E+02	5	4.5486E+00	FishBase
6296	Ecto.verts	Oncorhynchus mykiss	1.0000E+02	15	4.5486E+00	FishBase
6297	Ecto.verts	Oncorhynchus mykiss	1.0000E+02	15	4.8286E+00	FishBase
6298	Ecto.verts	Oncorhynchus mykiss	1.0000E+02	5	5.1784E+00	FishBase
6299	Ecto.verts	Oncorhynchus mykiss	1.0000E+02	15	6.5780E+00	FishBase
6300	Ecto.verts	Oncorhynchus mykiss	1.0000E+02	15	7.6277E+00	FishBase
6301	Ecto.verts	Oncorhynchus mykiss	1.0000E+02	15	7.8376E+00	FishBase
6302	Ecto.verts	Oncorhynchus mykiss	1.0000E+02	15	8.8873E+00	FishBase

6303	Ecto.verts	Oncorhynchus mykiss	1.1500E+02	12	7.7257E+00	FishBase
6304	Ecto.verts	Oncorhynchus mykiss	1.1500E+02	12	8.7719E+00	FishBase
6305	Ecto.verts	Oncorhynchus mykiss	1.1540E+02	14	6.4605E+00	FishBase
6306	Ecto.verts	Oncorhynchus mykiss	2.1000E+02	8.6	6.0252E+00	FishBase
6307	Ecto.verts	Oncorhynchus mykiss	2.1000E+02	8.6	6.4661E+00	FishBase
6308	Ecto.verts	Oncorhynchus mykiss	2.1000E+02	8.6	6.9069E+00	FishBase
6309	Ecto.verts	Oncorhynchus mykiss	2.1000E+02	8.6	7.0539E+00	FishBase
6310	Ecto.verts	Oncorhynchus mykiss	2.1000E+02	8.6	7.2008E+00	FishBase
6311	Ecto.verts	Oncorhynchus mykiss	2.1000E+02	8.6	7.3478E+00	FishBase
6312	Ecto.verts	Oncorhynchus mykiss	2.1000E+02	8.6	7.4948E+00	FishBase
6313	Ecto.verts	Oncorhynchus mykiss	2.1000E+02	8.6	7.6417E+00	FishBase
6314	Ecto.verts	Oncorhynchus mykiss	2.1000E+02	8.6	7.7887E+00	FishBase
6315	Ecto.verts	Oncorhynchus mykiss	2.1000E+02	8.6	7.9356E+00	FishBase
6316	Ecto.verts	Oncorhynchus mykiss	2.1000E+02	8.6	7.9356E+00	FishBase
6317	Ecto.verts	Oncorhynchus mykiss	2.1000E+02	8.6	8.3765E+00	FishBase
6318	Ecto.verts	Oncorhynchus mykiss	2.1000E+02	8.6	8.8174E+00	FishBase
6319	Ecto.verts	Oncorhynchus mykiss	2.1000E+02	8.6	8.9643E+00	FishBase
6320	Ecto.verts	Oncorhynchus mykiss	2.1000E+02	8.6	9.4052E+00	FishBase
6321	Ecto.verts	Oncorhynchus mykiss	2.1000E+02	8.6	9.5521E+00	FishBase
6322	Ecto.verts	Oncorhynchus mykiss	2.1000E+02	8.6	9.9930E+00	FishBase
6323	Ecto.verts	Oncorhynchus mykiss	2.1000E+02	8.6	1.1169E+01	FishBase
6324	Ecto.verts	Oncorhynchus mykiss	2.1700E+02	8.6	8.3520E+00	FishBase
6325	Ecto.verts	Oncorhynchus mykiss	2.1800E+02	12	6.8649E+00	FishBase
6326	Ecto.verts	Oncorhynchus mykiss	2.1900E+02	8	1.1494E+01	FishBase
6327	Ecto.verts	Oncorhynchus mykiss	2.2400E+02	8	1.0973E+01	FishBase
6328	Ecto.verts	Oncorhynchus mykiss	2.2500E+02	15	1.4643E+01	FishBase

6329	Ecto.verts	Oncorhynchus mykiss	2.2500E+02	15	1.4801E+01	FishBase
6330	Ecto.verts	Oncorhynchus mykiss	2.2500E+02	15	1.5273E+01	FishBase
6331	Ecto.verts	Oncorhynchus mykiss	2.2500E+02	15	1.6218E+01	FishBase
6332	Ecto.verts	Oncorhynchus mykiss	2.2500E+02	15	1.7792E+01	FishBase
6333	Ecto.verts	Oncorhynchus mykiss	2.3800E+02	8	1.1825E+01	FishBase
6334	Ecto.verts	Oncorhynchus mykiss	2.6500E+02	8.8	7.7887E+00	FishBase
6335	Ecto.verts	Oncorhynchus mykiss	2.8800E+02	15	1.5922E+01	FishBase
6336	Ecto.verts	Oncorhynchus mykiss	3.0000E+02	15	1.7425E+01	FishBase
6337	Ecto.verts	Oncorhynchus mykiss	3.5000E+02	10	1.6900E+01	FishBase
6338	Ecto.verts	Oncorhynchus mykiss	3.5000E+02	10	2.0329E+01	FishBase
6339	Ecto.verts	Oncorhynchus mykiss	3.5000E+02	10	2.1309E+01	FishBase
6340	Ecto.verts	Oncorhynchus mykiss	3.5000E+02	10	2.2288E+01	FishBase
6341	Ecto.verts	Oncorhynchus mykiss	3.5000E+02	10	2.4738E+01	FishBase
6342	Ecto.verts	Oncorhynchus mykiss	3.5000E+02	10	2.6697E+01	FishBase
6343	Ecto.verts	Oncorhynchus mykiss	4.5000E+02	5	1.2281E+01	FishBase
6344	Ecto.verts	Oncorhynchus mykiss	5.0000E+02	13.5	1.9244E+01	FishBase
6345	Ecto.verts	Oncorhynchus mykiss	5.0000E+02	13.5	2.1344E+01	FishBase
6346	Ecto.verts	Oncorhynchus mykiss	5.0000E+02	13.5	2.1693E+01	FishBase
6347	Ecto.verts	Oncorhynchus mykiss	5.0000E+02	13.5	2.3093E+01	FishBase
6348	Ecto.verts	Oncorhynchus mykiss	5.0000E+02	15	2.4493E+01	FishBase
6349	Ecto.verts	Oncorhynchus mykiss	5.0000E+02	15	2.4843E+01	FishBase
6350	Ecto.verts	Oncorhynchus mykiss	5.0000E+02	15	2.5192E+01	FishBase
6351	Ecto.verts	Oncorhynchus mykiss	5.0000E+02	13.5	2.9741E+01	FishBase
6352	Ecto.verts	Oncorhynchus mykiss	5.1500E+02	16	3.9643E+01	FishBase
6353	Ecto.verts	Oncorhynchus mykiss	5.1500E+02	18	4.5049E+01	FishBase
6354	Ecto.verts	Oncorhynchus mykiss	5.1500E+02	20	5.4059E+01	FishBase

6355	Ecto.verts	Oncorhynchus mykiss	5.1500E+02	22	7.5682E+01	FishBase
6356	Ecto.verts	Oncorhynchus mykiss	5.1500E+02	25	9.7306E+01	FishBase
6357	Ecto.verts	Oncorhynchus mykiss	5.1500E+02	26	9.9108E+01	FishBase
6358	Ecto.verts	Oncorhynchus mykiss	5.1500E+02	24	1.0632E+02	FishBase
6359	Ecto.verts	Oncorhynchus mykiss	5.6500E+02	15	3.3607E+01	FishBase
6360	Ecto.verts	Oncorhynchus mykiss	5.9500E+02	15	3.1228E+01	FishBase
6361	Ecto.verts	Oncorhynchus mykiss	7.0000E+02	15	3.8698E+01	FishBase
6362	Ecto.verts	Oncorhynchus mykiss	7.6800E+02	15	5.2132E+01	FishBase
6363	Ecto.verts	Oncorhynchus mykiss	7.6800E+02	15	5.8043E+01	FishBase
6364	Ecto.verts	Oncorhynchus mykiss	7.6800E+02	15	6.6105E+01	FishBase
6365	Ecto.verts	Oncorhynchus mykiss	7.6800E+02	15	6.7180E+01	FishBase
6366	Ecto.verts	Oncorhynchus mykiss	1.2000E+03	10	4.0308E+01	FishBase
6367	Ecto.verts	Oncorhynchus nerka	3.3800E+00	15	5.4402E-01	FishBase
6368	Ecto.verts	Oncorhynchus nerka	8.4700E+00	15	6.5199E-01	FishBase
6369	Ecto.verts	Oncorhynchus nerka	1.9100E+01	15	1.6975E+00	FishBase
6370	Ecto.verts	Oncorhynchus nerka	3.2900E+01	10	1.3814E+00	FishBase
6371	Ecto.verts	Oncorhynchus nerka	3.6700E+01	5	1.0530E+00	FishBase
6372	Ecto.verts	Oncorhynchus nerka	4.7000E+01	5	1.3485E+00	FishBase
6373	Ecto.verts	Oncorhynchus nerka	4.7000E+01	15	2.8286E+00	FishBase
6374	Ecto.verts	Oncorhynchus nerka	4.7000E+01	20	3.8810E+00	FishBase
6375	Ecto.verts	Oncorhynchus nerka	5.2200E+01	25	7.1597E+00	FishBase
6376	Ecto.verts	Oncorhynchus nerka	5.5200E+01	15	2.7426E+00	FishBase
6377	Ecto.verts	Oncorhynchus nerka	6.2600E+01	20	5.2568E+00	FishBase
6378	Ecto.verts	Oncorhynchus nerka	1.0000E+02	15	5.8083E+00	FishBase
6379	Ecto.verts	Oncorhynchus nerka	2.6400E+02	10.5	1.5519E+01	FishBase
6380	Ecto.verts	Oncorhynchus nerka	7.4600E+02	15	3.7065E+01	FishBase

6381	Ecto.verts	Oncorhynchus nerka	1.4320E+03	15	4.4092E+01	FishBase
6382	Ecto.verts	Ophiodon elongatus	2.7000E+02	12.2	1.1714E+01	FishBase
6383	Ecto.verts	Ophiodon elongatus	5.2000E+02	13.1	2.1833E+01	FishBase
6384	Ecto.verts	Ophiodon elongatus	8.5000E+02	13.2	3.2715E+01	FishBase
6385	Ecto.verts	Ophiodon elongatus	9.7000E+02	12	3.7334E+01	FishBase
6386	Ecto.verts	Ophiodon elongatus	1.5000E+03	13	5.7733E+01	FishBase
6387	Ecto.verts	Ophiodon elongatus	1.5910E+03	12.1	2.7834E+01	FishBase
6388	Ecto.verts	Ophiodon elongatus	1.7200E+03	12.1	3.2498E+01	FishBase
6389	Ecto.verts	Ophiodon elongatus	2.0900E+03	12	4.3877E+01	FishBase
6390	Ecto.verts	Ophiodon elongatus	3.4000E+03	10	7.3758E+01	FishBase
6391	Ecto.verts	Ophiodon elongatus	3.7700E+03	12.4	1.1608E+02	FishBase
6392	Ecto.verts	Ophiodon elongatus	4.4000E+03	13	9.8530E+01	FishBase
6393	Ecto.verts	Ophiodon elongatus	6.8200E+03	12	1.5272E+02	FishBase
6394	Ecto.verts	Ophiodon elongatus	7.3300E+03	11.8	1.5388E+02	FishBase
6395	Ecto.verts	Ophiodon elongatus	9.0900E+03	12	2.2264E+02	FishBase
6396	Ecto.verts	Ophisaurus ventralis	3.2185E+01	25	1.2423E+00	White et al. 2006
6397	Ecto.verts	Oreochromis aureus	6.9700E+02	26	3.1704E+01	FishBase
6398	Ecto.verts	Oreochromis mossambicus	4.7000E+01	16	1.7103E+00	FishBase
6399	Ecto.verts	Oreochromis mossambicus	4.7000E+01	19	2.2036E+00	FishBase
6400	Ecto.verts	Oreochromis mossambicus	4.7000E+01	15	2.8943E+00	FishBase
6401	Ecto.verts	Oreochromis mossambicus	4.7000E+01	22	3.2890E+00	FishBase
6402	Ecto.verts	Oreochromis mossambicus	4.7000E+01	25	4.5388E+00	FishBase
6403	Ecto.verts	Oreochromis mossambicus	4.7000E+01	28	5.4269E+00	FishBase
6404	Ecto.verts	Oreochromis mossambicus	4.7000E+01	20	5.7229E+00	FishBase
6405	Ecto.verts	Oreochromis mossambicus	4.7000E+01	32	7.8279E+00	FishBase
6406	Ecto.verts	Oreochromis mossambicus	4.7000E+01	25	9.3408E+00	FishBase

6407	Ecto.verts	Oreochromis mossambicus	4.7000E+01	37	9.5710E+00	FishBase
6408	Ecto.verts	Oreochromis mossambicus	4.7000E+01	30	1.3485E+01	FishBase
6409	Ecto.verts	Oreochromis mossambicus	4.7000E+01	40	1.8978E+01	FishBase
6410	Ecto.verts	Oreochromis mossambicus	4.7000E+01	35	1.9899E+01	FishBase
6411	Ecto.verts	Oreochromis mossambicus	4.7000E+01	34	8.2883E+00	FishBase
6412	Ecto.verts	Oreochromis niloticus	4.7000E+01	25	3.0917E+00	FishBase
6413	Ecto.verts	Oreochromis niloticus	4.7000E+01	25	1.0952E+01	FishBase
6414	Ecto.verts	Oreochromis niloticus	8.0000E+01	25	5.8223E+00	FishBase
6415	Ecto.verts	Oreochromis niloticus	8.0000E+01	25	7.5017E+00	FishBase
6416	Ecto.verts	Oreochromis niloticus	3.1000E+02	26	1.2365E+01	FishBase
6417	Ecto.verts	Orthodon microlepidotus	5.6400E+02	10	1.6577E+01	FishBase
6418	Ecto.verts	Orthodon microlepidotus	5.7400E+02	25	5.6235E+01	FishBase
6419	Ecto.verts	Orthodon microlepidotus	5.8300E+02	30	7.7924E+01	FishBase
6420	Ecto.verts	Orthodon microlepidotus	5.8800E+02	20	4.0736E+01	FishBase
6421	Ecto.verts	Orthodon microlepidotus	6.5000E+02	12	1.4556E+01	FishBase
6422	Ecto.verts	Orthodon microlepidotus	6.5000E+02	20	4.0938E+01	FishBase
6423	Ecto.verts	Orthodon microlepidotus	6.5000E+02	28	5.6858E+01	FishBase
6424	Ecto.verts	Orthodon microlepidotus	6.7800E+02	15	3.6533E+01	FishBase
6425	Ecto.verts	Osteopilus septentrionalis	4.9800E+00	20	3.3000E-01	White et al. 2006
6426	Ecto.verts	Osteopilus septentrionalis	5.0000E+00	20	3.3000E-01	White et al. 2006
6427	Ecto.verts	Oxyrhopus trigeminus	9.8000E+01	20	3.4300E+00	White et al. 2006
6428	Ecto.verts	Pagothenia borchgrevinki	4.7000E+01	0	1.8747E+00	FishBase
6429	Ecto.verts	Pelamis platurus	1.1600E+02	30	6.9600E+00	White et al. 2006
6430	Ecto.verts	Petromyzon marinus	2.2800E+01	5	8.4563E-01	FishBase
6431	Ecto.verts	Petromyzon marinus	3.4800E+01	10	1.5829E+00	FishBase
6432	Ecto.verts	Petromyzon marinus	3.5700E+01	15	2.8480E+00	FishBase

6433	Ecto.verts	Petromyzon marinus	7.5600E+01	20	6.5601E+00	FishBase
6434	Ecto.verts	Philodryas olfersii	1.7600E+02	20	6.8640E+00	White et al. 2006
6435	Ecto.verts	Philodryas patagoniensis	3.8800E+02	20	1.9788E+01	White et al. 2006
6436	Ecto.verts	Philodryas serra	1.3500E+02	20	4.7250E+00	White et al. 2006
6437	Ecto.verts	Phrynosoma cornutum	3.5000E+01	25	3.3600E+00	White et al. 2006
6438	Ecto.verts	Phrynosoma cornutum	3.5000E+01	35	5.8100E+00	White et al. 2006
6439	Ecto.verts	Phrynosoma douglassi	2.8000E+01	25	3.2200E+00	White et al. 2006
6440	Ecto.verts	Phrynosoma douglassi	2.8000E+01	35	4.7880E+00	White et al. 2006
6441	Ecto.verts	Phrynosoma m'calli	1.6000E+01	20	1.1200E+00	White et al. 2006
6442	Ecto.verts	Phrynosoma m'calli	1.6000E+01	30	2.7200E+00	White et al. 2006
6443	Ecto.verts	Phrynosoma m'calli	1.6000E+01	37	4.4000E+00	White et al. 2006
6444	Ecto.verts	Phyllobates subpunctatus	1.5000E+00	15	1.0800E-01	White et al. 2006
6445	Ecto.verts	Phyllomedusa sauvagei	1.7500E+01	10	5.0750E-01	White et al. 2006
6446	Ecto.verts	Phyllomedusa sauvagei	1.7500E+01	15	7.7000E-01	White et al. 2006
6447	Ecto.verts	Phyllomedusa sauvagei	1.7500E+01	25	1.8025E+00	White et al. 2006
6448	Ecto.verts	Phyllomedusa sauvagei	1.7500E+01	30	2.7475E+00	White et al. 2006
6449	Ecto.verts	Phyllomedusa sauvagei	1.7500E+01	35	4.1825E+00	White et al. 2006
6450	Ecto.verts	Phyllomedusa sauvagei	1.7500E+01	38	6.1250E+00	White et al. 2006
6451	Ecto.verts	Physalaemus pustulosus	1.7200E+00	25	2.6000E-01	White et al. 2006
6452	Ecto.verts	Physalaemus pustulosus	2.9000E+00	25	3.3930E-01	White et al. 2006
6453	Ecto.verts	Physignathus lesueurii	5.0400E+02	20	1.6582E+01	White et al. 2006
6454	Ecto.verts	Physignathus lesueurii	5.0400E+02	30	4.0320E+01	White et al. 2006
6455	Ecto.verts	Physignathus lesueurii	5.0400E+02	37	7.0560E+01	White et al. 2006
6456	Ecto.verts	Pituophis catenifer affinis	5.4800E+02	10	8.7680E+00	White et al. 2006
6457	Ecto.verts	Pituophis catenifer affinis	5.4800E+02	20	1.0960E+01	White et al. 2006
6458	Ecto.verts	Pituophis catenifer affinis	5.4800E+02	30	2.1920E+01	White et al. 2006

6459	Ecto.verts	Pituophis catenifer affinis	5.4800E+02	40	7.5076E+01	White et al. 2006
6460	Ecto.verts	Pituophis melanolecus	5.4800E+02	20	1.0412E+01	White et al. 2006
6461	Ecto.verts	Pituophis melanolecus	5.4800E+02	30	3.8360E+01	White et al. 2006
6462	Ecto.verts	Pituophis melanolecus	6.2200E+02	20	2.3014E+01	White et al. 2006
6463	Ecto.verts	Platichthys flesus	4.7000E+01	5	9.2092E-01	FishBase
6464	Ecto.verts	Platichthys flesus	4.7000E+01	2	9.8670E-01	FishBase
6465	Ecto.verts	Platichthys flesus	4.7000E+01	6	1.6445E+00	FishBase
6466	Ecto.verts	Platichthys flesus	4.7000E+01	15	1.8418E+00	FishBase
6467	Ecto.verts	Platichthys flesus	4.7000E+01	10	2.7957E+00	FishBase
6468	Ecto.verts	Platichthys flesus	4.7000E+01	14	3.6179E+00	FishBase
6469	Ecto.verts	Platichthys flesus	4.7000E+01	18	4.6046E+00	FishBase
6470	Ecto.verts	Platichthys flesus	4.7000E+01	22	4.9335E+00	FishBase
6471	Ecto.verts	Platichthys flesus	3.5000E+02	9	2.6942E+00	FishBase
6472	Ecto.verts	Platichthys flesus	3.5000E+02	9	4.8985E+00	FishBase
6473	Ecto.verts	Platichthys flesus	3.5000E+02	9	9.5521E+00	FishBase
6474	Ecto.verts	Platichthys flesus	3.9500E+02	15	1.0227E+01	FishBase
6475	Ecto.verts	Platichthys flesus	4.3400E+02	5	5.4668E+00	FishBase
6476	Ecto.verts	Platichthys flesus	4.9400E+02	10	1.5211E+01	FishBase
6477	Ecto.verts	Platichthys stellatus	5.8080E+02	9	1.5445E+01	FishBase
6478	Ecto.verts	Platichthys stellatus	6.8530E+02	9	1.8224E+01	FishBase
6479	Ecto.verts	Platichthys stellatus	1.1750E+03	11.5	2.4668E+01	FishBase
6480	Ecto.verts	Platichthys stellatus	1.1750E+03	11.2	2.7134E+01	FishBase
6481	Ecto.verts	Platichthys stellatus	1.1750E+03	20	6.9069E+01	FishBase
6482	Ecto.verts	Platichthys stellatus	1.2000E+03	11.4	1.7635E+01	FishBase
6483	Ecto.verts	Platichthys stellatus	1.2000E+03	19.8	6.1302E+01	FishBase
6484	Ecto.verts	Platichthys stellatus	1.2680E+03	9	2.8395E+01	FishBase

6485	Ecto.verts	Platichthys stellatus	1.2680E+03	9.5	3.0169E+01	FishBase
6486	Ecto.verts	Platichthys stellatus	1.2680E+03	17.8	5.6789E+01	FishBase
6487	Ecto.verts	Platichthys stellatus	1.2900E+03	9	1.4444E+01	FishBase
6488	Ecto.verts	Platichthys stellatus	1.2900E+03	9	2.1666E+01	FishBase
6489	Ecto.verts	Platichthys stellatus	1.2900E+03	16.6	3.5206E+01	FishBase
6490	Ecto.verts	Platichthys stellatus	1.4250E+03	11	2.8919E+01	FishBase
6491	Ecto.verts	Platichthys stellatus	1.4250E+03	11	4.1882E+01	FishBase
6492	Ecto.verts	Platichthys stellatus	1.4400E+03	13	4.4339E+01	FishBase
6493	Ecto.verts	Platichthys stellatus	1.4400E+03	12.3	5.3408E+01	FishBase
6494	Ecto.verts	Platichthys stellatus	1.4400E+03	19.8	8.8677E+01	FishBase
6495	Ecto.verts	Platichthys stellatus	1.4500E+03	11.8	1.7250E+01	FishBase
6496	Ecto.verts	Platichthys stellatus	1.4500E+03	19.5	9.2337E+01	FishBase
6497	Ecto.verts	Platichthys stellatus	1.4750E+03	11.5	3.3030E+01	FishBase
6498	Ecto.verts	Platichthys stellatus	1.4750E+03	10.2	4.6449E+01	FishBase
6499	Ecto.verts	Plethodon cinereus	6.0000E-01	17.5	1.6800E-02	White et al. 2006
6500	Ecto.verts	Plethodon cinereus	7.0000E-01	5	1.0000E-02	White et al. 2006
6501	Ecto.verts	Plethodon cinereus	7.0000E-01	10	1.5000E-02	White et al. 2006
6502	Ecto.verts	Plethodon cinereus	7.0000E-01	14	1.9000E-02	White et al. 2006
6503	Ecto.verts	Plethodon cinereus	7.0000E-01	17.5	2.8000E-02	White et al. 2006
6504	Ecto.verts	Plethodon cinereus	7.0000E-01	21	3.4000E-02	White et al. 2006
6505	Ecto.verts	Plethodon cinereus	8.0000E-01	15	3.2000E-02	White et al. 2006
6506	Ecto.verts	Plethodon cinereus	8.4400E-01	1	1.2000E-02	White et al. 2006
6507	Ecto.verts	Plethodon cinereus	8.6200E-01	10	3.2300E-02	White et al. 2006
6508	Ecto.verts	Plethodon cinereus	8.9000E-01	20	4.5100E-02	White et al. 2006
6509	Ecto.verts	Plethodon cinereus	9.0000E-01	17.5	3.0000E-02	White et al. 2006
6510	Ecto.verts	Plethodon cinereus	9.4000E-01	18.5	4.1500E-02	White et al. 2006

6511	Ecto.verts	Plethodon cinereus	9.9000E-01	15.5	3.9000E-02	White et al. 2006
6512	Ecto.verts	Plethodon cinereus	1.0000E+00	20	4.7600E-02	White et al. 2006
6513	Ecto.verts	Plethodon dorsalis	6.6000E-01	15	2.9300E-02	White et al. 2006
6514	Ecto.verts	Plethodon dorsalis	6.9000E-01	25	2.3900E-02	White et al. 2006
6515	Ecto.verts	Plethodon dorsalis	7.1000E-01	20	2.2200E-02	White et al. 2006
6516	Ecto.verts	Plethodon dorsalis	7.5000E-01	5	8.3000E-03	White et al. 2006
6517	Ecto.verts	Plethodon dorsalis	7.7000E-01	10	1.7800E-02	White et al. 2006
6518	Ecto.verts	Plethodon glutinosus	3.6500E+00	10	7.6200E-02	White et al. 2006
6519	Ecto.verts	Plethodon glutinosus	3.6500E+00	15	1.1670E-01	White et al. 2006
6520	Ecto.verts	Plethodon glutinosus	3.6500E+00	20	1.5830E-01	White et al. 2006
6521	Ecto.verts	Plethodon glutinosus	3.7500E+00	5	4.4300E-02	White et al. 2006
6522	Ecto.verts	Plethodon glutinosus	3.9100E+00	25	3.2370E-01	White et al. 2006
6523	Ecto.verts	Plethodon glutinosus	4.2000E+00	15	3.4440E-01	White et al. 2006
6524	Ecto.verts	Plethodon glutinosus	4.2500E+00	15	1.4410E-01	White et al. 2006
6525	Ecto.verts	Plethodon glutinosus	4.3300E+00	14	2.4640E-01	White et al. 2006
6526	Ecto.verts	Plethodon glutinosus	5.0100E+00	20	1.6710E-01	White et al. 2006
6527	Ecto.verts	Plethodon jordani	1.8300E+00	15	5.1100E-02	White et al. 2006
6528	Ecto.verts	Plethodon jordani	1.8300E+00	25	1.2430E-01	White et al. 2006
6529	Ecto.verts	Plethodon jordani	2.2000E+00	17.5	7.1000E-02	White et al. 2006
6530	Ecto.verts	Plethodon jordani	3.1000E+00	5	2.7000E-02	White et al. 2006
6531	Ecto.verts	Plethodon jordani	3.1000E+00	10	5.8000E-02	White et al. 2006
6532	Ecto.verts	Plethodon jordani	3.1000E+00	14	8.7000E-02	White et al. 2006
6533	Ecto.verts	Plethodon jordani	3.1000E+00	17.5	1.0900E-01	White et al. 2006
6534	Ecto.verts	Plethodon jordani	3.1000E+00	21	1.4800E-01	White et al. 2006
6535	Ecto.verts	Plethodon jordani	4.3000E+00	17.5	1.3800E-01	White et al. 2006
6536	Ecto.verts	Plethodon neomexicanus	2.4700E+00	5	9.1400E-02	White et al. 2006

6537	Ecto.verts	Plethodon neomexicanus	2.4700E+00	10	1.1860E-01	White et al. 2006
6538	Ecto.verts	Plethodon neomexicanus	2.4700E+00	15	1.3590E-01	White et al. 2006
6539	Ecto.verts	Plethodon neomexicanus	2.4700E+00	20	1.4080E-01	White et al. 2006
6540	Ecto.verts	Plethodon neomexicanus	2.4700E+00	25	1.5070E-01	White et al. 2006
6541	Ecto.verts	Plethodon spp.	3.2500E+00	22	1.8590E-01	White et al. 2006
6542	Ecto.verts	Pleuronectes platessa	2.7000E+01	10	2.3996E+00	FishBase
6543	Ecto.verts	Pleuronectes platessa	2.7000E+01	20	3.1742E+00	FishBase
6544	Ecto.verts	Pleuronectes platessa	4.7000E+01	2	1.0525E+00	FishBase
6545	Ecto.verts	Pleuronectes platessa	4.7000E+01	6	1.5458E+00	FishBase
6546	Ecto.verts	Pleuronectes platessa	4.7000E+01	10	2.4668E+00	FishBase
6547	Ecto.verts	Pleuronectes platessa	4.7000E+01	14	2.7299E+00	FishBase
6548	Ecto.verts	Pleuronectes platessa	4.7000E+01	18	3.1575E+00	FishBase
6549	Ecto.verts	Pleuronectes platessa	4.7000E+01	10	3.4206E+00	FishBase
6550	Ecto.verts	Pleuronectes platessa	4.7000E+01	22	4.4731E+00	FishBase
6551	Ecto.verts	Pleuronectes platessa	9.2000E+01	20	4.6998E+00	FishBase
6552	Ecto.verts	Pleuronectes platessa	1.0500E+02	15	4.6291E+00	FishBase
6553	Ecto.verts	Pleuronectes platessa	1.0600E+02	20	4.5990E+00	FishBase
6554	Ecto.verts	Pleuronectes platessa	1.1000E+02	10	3.3870E+00	FishBase
6555	Ecto.verts	Pleuronectes platessa	1.1000E+02	15	5.0035E+00	FishBase
6556	Ecto.verts	Pleuronectes platessa	1.1000E+02	20	1.0007E+01	FishBase
6557	Ecto.verts	Pleuronectes platessa	1.2000E+02	10	3.1071E+00	FishBase
6558	Ecto.verts	Pleuronectes platessa	1.2000E+02	10	4.1987E+00	FishBase
6559	Ecto.verts	Pleuronectes platessa	1.2000E+02	15	5.4584E+00	FishBase
6560	Ecto.verts	Pleuronectes platessa	1.2400E+02	10	2.6900E+00	FishBase
6561	Ecto.verts	Pleuronectes platessa	1.2700E+02	10	3.6438E+00	FishBase
6562	Ecto.verts	Pleuronectes platessa	1.3000E+02	20	7.7327E+00	FishBase

6563	Ecto.verts	Pleuronectes platessa	1.3200E+02	20	8.8677E+00	FishBase
6564	Ecto.verts	Pleuronectes platessa	1.5000E+02	10	4.3037E+00	FishBase
6565	Ecto.verts	Pleuronectes platessa	1.5800E+02	10	4.8649E+00	FishBase
6566	Ecto.verts	Pleuronectes platessa	1.6400E+02	10	5.3940E+00	FishBase
6567	Ecto.verts	Pleuronectes platessa	1.8600E+02	10	5.4668E+00	FishBase
6568	Ecto.verts	Pleuronectes platessa	2.8100E+02	15	1.0029E+01	FishBase
6569	Ecto.verts	Pleuronectes platessa	2.8860E+02	5	3.8372E+00	FishBase
6570	Ecto.verts	Pleuronectes platessa	2.9400E+02	10	8.4353E+00	FishBase
6571	Ecto.verts	Pleuronectes platessa	3.2200E+02	10	8.5626E+00	FishBase
6572	Ecto.verts	Pleuronectes platessa	3.8300E+02	10	8.5766E+00	FishBase
6573	Ecto.verts	Pleuronectes platessa	5.7200E+02	10	1.2809E+01	FishBase
6574	Ecto.verts	Pleuronectes platessa	6.3200E+02	10	2.0344E+01	FishBase
6575	Ecto.verts	Podarcis hispanica	3.4300E+00	5	4.4590E-02	White et al. 2006
6576	Ecto.verts	Podarcis hispanica	3.4300E+00	10	6.8600E-02	White et al. 2006
6577	Ecto.verts	Podarcis hispanica	3.4300E+00	15	1.1662E-01	White et al. 2006
6578	Ecto.verts	Podarcis hispanica	3.4300E+00	20	3.0870E-01	White et al. 2006
6579	Ecto.verts	Podarcis hispanica	3.4300E+00	25	5.3165E-01	White et al. 2006
6580	Ecto.verts	Podarcis hispanica	3.4300E+00	30	7.9233E-01	White et al. 2006
6581	Ecto.verts	Podarcis hispanica	3.4300E+00	35	9.6040E-01	White et al. 2006
6582	Ecto.verts	Podarcis hispanica	3.6000E+00	20	5.7600E-01	White et al. 2006
6583	Ecto.verts	Podarcis hispanica	3.6000E+00	30	1.3320E+00	White et al. 2006
6584	Ecto.verts	Podarcis hispanica	3.6000E+00	35	2.1060E+00	White et al. 2006
6585	Ecto.verts	Podarcis lilfordi brauni	7.4000E+00	20	5.4020E-01	White et al. 2006
6586	Ecto.verts	Podarcis lilfordi brauni	7.4000E+00	30	2.5160E+00	White et al. 2006
6587	Ecto.verts	Podarcis lilfordi lilfordi	8.3000E+00	20	7.0550E-01	White et al. 2006
6588	Ecto.verts	Podarcis lilfordi lilfordi	8.3000E+00	30	1.7015E+00	White et al. 2006

6589	Ecto.verts	Podarcis muralis	5.5000E+00	20	9.4600E-01	White et al. 2006
6590	Ecto.verts	Podarcis muralis	5.5000E+00	35	2.3485E+00	White et al. 2006
6591	Ecto.verts	Podarcis muralis	5.5000E+00	30	2.5795E+00	White et al. 2006
6592	Ecto.verts	Podarcis sicula	7.0000E+00	30	9.8000E-01	White et al. 2006
6593	Ecto.verts	Podarcis sicula	9.0000E+00	20	4.8960E-01	White et al. 2006
6594	Ecto.verts	Pogonophryne scotti	4.7000E+01	-1	1.4472E+00	FishBase
6595	Ecto.verts	Protopterus annectens	8.0000E+01	30	8.7334E+00	FishBase
6596	Ecto.verts	Protopterus annectens	1.0700E+02	30	1.1456E+01	FishBase
6597	Ecto.verts	Protopterus annectens	1.0700E+02	30	1.2579E+01	FishBase
6598	Ecto.verts	Protopterus annectens	1.0700E+02	30	1.2804E+01	FishBase
6599	Ecto.verts	Protopterus annectens	2.1200E+02	30	1.9435E+01	FishBase
6600	Ecto.verts	Protopterus annectens	2.1200E+02	30	2.3589E+01	FishBase
6601	Ecto.verts	Protopterus annectens	2.1200E+02	30	2.4775E+01	FishBase
6602	Ecto.verts	Protopterus annectens	3.6800E+02	30	2.0344E+01	FishBase
6603	Ecto.verts	Protopterus annectens	3.6800E+02	30	2.5237E+01	FishBase
6604	Ecto.verts	Protopterus annectens	3.6800E+02	30	2.8843E+01	FishBase
6605	Ecto.verts	Psammodromus algirus	5.2000E+00	20	6.3960E-01	White et al. 2006
6606	Ecto.verts	Psammodromus algirus	5.2000E+00	30	1.7888E+00	White et al. 2006
6607	Ecto.verts	Psammodromus algirus	5.2000E+00	35	2.6260E+00	White et al. 2006
6608	Ecto.verts	Pseudacris nigrita	1.0000E+00	25	1.6200E-01	White et al. 2006
6609	Ecto.verts	Pseudacris triseriata	9.4000E-01	5	1.4100E-02	White et al. 2006
6610	Ecto.verts	Pseudacris triseriata	9.4000E-01	25	1.1190E-01	White et al. 2006
6611	Ecto.verts	Pseudacris triseriata	1.0560E+00	20	4.8360E-01	White et al. 2006
6612	Ecto.verts	Pseudacris triseriata	1.0650E+00	10	2.5560E-01	White et al. 2006
6613	Ecto.verts	Pseudacris triseriata	1.1250E+00	20	2.6870E-01	White et al. 2006
6614	Ecto.verts	Pseudacris triseriata	1.1920E+00	10	1.7740E-01	White et al. 2006

6615	Ecto.verts	Pseudacris triseriata	1.3100E+00	25	5.0150E-01	White et al. 2006
6616	Ecto.verts	Pseudacris triseriata	1.3400E+00	15	3.0150E-01	White et al. 2006
6617	Ecto.verts	Pseudacris triseriata	1.4100E+00	5	1.6950E-01	White et al. 2006
6618	Ecto.verts	Pseudacris triseriata	1.9000E+00	18.5	2.0900E-01	White et al. 2006
6619	Ecto.verts	Pseudemys scripta	3.0500E+02	10	1.1590E+00	White et al. 2006
6620	Ecto.verts	Pseudemys scripta	3.0500E+02	20	3.3245E+00	White et al. 2006
6621	Ecto.verts	Pseudemys scripta	3.0500E+02	30	9.5465E+00	White et al. 2006
6622	Ecto.verts	Pseudemys scripta	3.0500E+02	40	2.7420E+01	White et al. 2006
6623	Ecto.verts	Pseudobranchus striatus	1.5800E+00	25	9.3200E-02	White et al. 2006
6624	Ecto.verts	Pseudobranchus striatus	2.1900E+00	25	1.1160E-01	White et al. 2006
6625	Ecto.verts	Pseudobranchus striatus	2.5000E+00	25	1.8400E-01	White et al. 2006
6626	Ecto.verts	Pseudocrenilabrus multicolor	4.7000E+01	27	3.1903E+00	FishBase
6627	Ecto.verts	Pseudoeurycea belli	1.0940E+01	15	1.8820E-01	White et al. 2006
6628	Ecto.verts	Pseudoeurycea belli	2.4450E+01	25	7.1880E-01	White et al. 2006
6629	Ecto.verts	Pseudoeurycea brunnata	3.3300E+00	15	6.5600E-01	White et al. 2006
6630	Ecto.verts	Pseudoeurycea cephalica	1.4500E+00	15	3.5400E-02	White et al. 2006
6631	Ecto.verts	Pseudoeurycea cochranae	2.2300E+00	15	4.5700E-02	White et al. 2006
6632	Ecto.verts	Pseudoeurycea gadovii	2.8800E+00	15	8.5000E-02	White et al. 2006
6633	Ecto.verts	Pseudoeurycea gadovii	2.9900E+00	25	2.4430E-01	White et al. 2006
6634	Ecto.verts	Pseudoeurycea gadovii	3.0900E+00	5	4.4200E-02	White et al. 2006
6635	Ecto.verts	Pseudoeurycea goebeli	3.6600E+00	15	8.3100E-02	White et al. 2006
6636	Ecto.verts	Pseudoeurycea goebeli	3.7300E+00	25	2.8420E-01	White et al. 2006
6637	Ecto.verts	Pseudoeurycea goebeli	3.7800E+00	5	3.5900E-02	White et al. 2006
6638	Ecto.verts	Pseudoeurycea leprosa	2.4600E+00	15	5.5600E-02	White et al. 2006
6639	Ecto.verts	Pseudoeurycea rex	1.8600E+00	15	3.9200E-02	White et al. 2006
6640	Ecto.verts	Pseudoeurycea smithii	4.2000E+00	15	1.0080E-01	White et al. 2006

6641	Ecto.verts	Pseudoeurycea smithii	4.2000E+00	25	2.6170E-01	White et al. 2006
6642	Ecto.verts	Pseudoeurycea smithii	6.6600E+00	15	1.1850E-01	White et al. 2006
6643	Ecto.verts	Pseudonaja nuchalis	2.1410E+02	24	7.9217E+00	White et al. 2006
6644	Ecto.verts	Pseudonaja nuchalis	2.1410E+02	27	1.1133E+01	White et al. 2006
6645	Ecto.verts	Pseudonaja nuchalis	2.1410E+02	30	1.4345E+01	White et al. 2006
6646	Ecto.verts	Pseudonaja nuchalis	2.1410E+02	33	1.5843E+01	White et al. 2006
6647	Ecto.verts	Pseudopleuronectes americanus	1.1250E+01	12	3.5427E-01	FishBase
6648	Ecto.verts	Pseudopleuronectes americanus	1.1250E+01	12	1.1337E+00	FishBase
6649	Ecto.verts	Pseudopleuronectes americanus	1.4800E+01	20	1.3153E+00	FishBase
6650	Ecto.verts	Pseudopleuronectes americanus	1.4800E+01	20	2.4235E+00	FishBase
6651	Ecto.verts	Pseudopleuronectes americanus	1.4960E+01	20	1.9681E+00	FishBase
6652	Ecto.verts	Pseudopleuronectes americanus	1.4960E+01	20	3.6432E+00	FishBase
6653	Ecto.verts	Pseudopleuronectes americanus	1.6090E+01	20	1.7227E+00	FishBase
6654	Ecto.verts	Pseudopleuronectes americanus	1.6090E+01	20	2.4433E+00	FishBase
6655	Ecto.verts	Pseudopleuronectes americanus	1.6890E+01	20	1.3001E+00	FishBase
6656	Ecto.verts	Pseudopleuronectes americanus	1.6890E+01	20	4.0186E+00	FishBase
6657	Ecto.verts	Pseudopleuronectes americanus	1.7170E+01	16	2.0426E+00	FishBase
6658	Ecto.verts	Pseudopleuronectes americanus	1.7170E+01	16	2.4391E+00	FishBase
6659	Ecto.verts	Pseudopleuronectes americanus	1.7190E+01	24	3.5487E+00	FishBase
6660	Ecto.verts	Pseudopleuronectes americanus	1.7190E+01	24	4.8358E+00	FishBase
6661	Ecto.verts	Pseudopleuronectes americanus	1.7460E+01	16	1.3074E+00	FishBase
6662	Ecto.verts	Pseudopleuronectes americanus	1.7460E+01	16	3.1279E+00	FishBase
6663	Ecto.verts	Pseudopleuronectes americanus	1.7920E+01	16	2.2572E+00	FishBase
6664	Ecto.verts	Pseudopleuronectes americanus	1.7920E+01	16	3.4987E+00	FishBase
6665	Ecto.verts	Pseudopleuronectes americanus	1.9110E+01	12	8.6924E-01	FishBase
6666	Ecto.verts	Pseudopleuronectes americanus	1.9110E+01	12	1.0832E+00	FishBase

6667	Ecto.verts	Pseudopleuronectes americanus	2.6150E+01	12	5.4899E-02	FishBase
6668	Ecto.verts	Pseudopleuronectes americanus	2.6150E+01	12	1.0797E+00	FishBase
6669	Ecto.verts	Pseudopleuronectes americanus	2.7410E+01	20	1.7455E+00	FishBase
6670	Ecto.verts	Pseudopleuronectes americanus	2.7410E+01	20	4.7953E+00	FishBase
6671	Ecto.verts	Pseudopleuronectes americanus	3.0180E+01	12	3.1679E-01	FishBase
6672	Ecto.verts	Pseudopleuronectes americanus	3.0180E+01	12	6.3359E-01	FishBase
6673	Ecto.verts	Pseudopleuronectes americanus	3.2630E+01	20	3.2196E+00	FishBase
6674	Ecto.verts	Pseudopleuronectes americanus	3.2630E+01	20	5.1377E+00	FishBase
6675	Ecto.verts	Pseudopleuronectes americanus	3.4120E+01	16	1.6714E+00	FishBase
6676	Ecto.verts	Pseudopleuronectes americanus	3.4120E+01	16	3.1756E+00	FishBase
6677	Ecto.verts	Pseudopleuronectes americanus	3.5480E+01	24	6.6789E+00	FishBase
6678	Ecto.verts	Pseudopleuronectes americanus	3.5480E+01	24	1.1620E+01	FishBase
6679	Ecto.verts	Pseudopleuronectes americanus	3.9480E+01	16	1.4643E+00	FishBase
6680	Ecto.verts	Pseudopleuronectes americanus	3.9480E+01	16	4.2547E+00	FishBase
6681	Ecto.verts	Pseudopleuronectes americanus	4.2500E+01	24	9.3387E+00	FishBase
6682	Ecto.verts	Pseudopleuronectes americanus	4.2500E+01	24	1.0737E+01	FishBase
6683	Ecto.verts	Pseudopleuronectes americanus	4.3180E+01	12	6.9499E-01	FishBase
6684	Ecto.verts	Pseudopleuronectes americanus	4.3180E+01	12	8.1586E-01	FishBase
6685	Ecto.verts	Pseudopleuronectes americanus	4.8660E+01	12	1.7026E-01	FishBase
6686	Ecto.verts	Pseudopleuronectes americanus	4.8660E+01	12	1.8728E+00	FishBase
6687	Ecto.verts	Pseudopleuronectes americanus	5.1860E+01	16	3.3388E+00	FishBase
6688	Ecto.verts	Pseudopleuronectes americanus	5.1860E+01	16	7.4034E+00	FishBase
6689	Ecto.verts	Pseudopleuronectes americanus	5.4060E+01	20	7.5283E+00	FishBase
6690	Ecto.verts	Pseudopleuronectes americanus	5.4060E+01	20	9.5712E+00	FishBase
6691	Ecto.verts	Pseudopleuronectes americanus	5.5030E+01	16	3.8895E+00	FishBase
6692	Ecto.verts	Pseudopleuronectes americanus	5.5030E+01	16	7.4708E+00	FishBase

6693	Ecto.verts	Pseudopleuronectes americanus	5.5970E+01	24	9.8701E+00	FishBase
6694	Ecto.verts	Pseudopleuronectes americanus	5.5970E+01	24	1.3160E+01	FishBase
6695	Ecto.verts	Pseudopleuronectes americanus	5.6880E+01	24	8.1200E+00	FishBase
6696	Ecto.verts	Pseudopleuronectes americanus	5.6880E+01	24	1.6200E+01	FishBase
6697	Ecto.verts	Pseudopleuronectes americanus	5.6800E+02	20	3.1003E+01	FishBase
6698	Ecto.verts	Pseudopleuronectes americanus	6.3400E+02	15	1.9078E+01	FishBase
6699	Ecto.verts	Pseudopleuronectes americanus	6.3500E+02	10	1.4220E+01	FishBase
6700	Ecto.verts	Pseudopleuronectes americanus	6.8100E+02	15	2.5734E+01	FishBase
6701	Ecto.verts	Pseudopleuronectes americanus	6.9600E+02	5	9.7411E+00	FishBase
6702	Ecto.verts	Pseudopleuronectes americanus	7.1600E+02	10	1.7036E+01	FishBase
6703	Ecto.verts	Pseudotriton ruber	5.8000E+00	15	4.2340E-01	White et al. 2006
6704	Ecto.verts	Pseudotriton ruber	1.0330E+01	15	2.3240E-01	White et al. 2006
6705	Ecto.verts	Pseudotriton ruber	1.0660E+01	25	6.1720E-01	White et al. 2006
6706	Ecto.verts	Pseudotriton ruber	1.0810E+01	5	6.8100E-02	White et al. 2006
6707	Ecto.verts	Ptyodactylus hasselquistii	8.5000E+00	20	5.1000E-01	White et al. 2006
6708	Ecto.verts	Ptyodactylus hasselquistii	8.5000E+00	30	1.2410E+00	White et al. 2006
6709	Ecto.verts	Python curtis	2.3735E+03	20	1.7725E+01	White et al. 2006
6710	Ecto.verts	Python curtis	2.3735E+03	30	4.5744E+01	White et al. 2006
6711	Ecto.verts	Python curtis	2.3735E+03	34	6.5588E+01	White et al. 2006
6712	Ecto.verts	Python molurus	2.3162E+04	30	3.6365E+02	White et al. 2006
6713	Ecto.verts	Python molurus	2.9777E+04	20	1.4782E+02	White et al. 2006
6714	Ecto.verts	Python molurus	3.3955E+04	34	6.7520E+02	White et al. 2006
6715	Ecto.verts	Python regius	7.8700E+02	20	6.8736E+00	White et al. 2006
6716	Ecto.verts	Python regius	8.3050E+02	34	2.8440E+01	White et al. 2006
6717	Ecto.verts	Python regius	8.8350E+02	30	2.0257E+01	White et al. 2006
6718	Ecto.verts	Python reticulatus	1.4326E+04	20	1.2882E+02	White et al. 2006

6719	Ecto.verts	Python reticulatus	1.4326E+04	30	3.3451E+02	White et al. 2006
6720	Ecto.verts	Python reticulatus	1.4326E+04	34	4.8686E+02	White et al. 2006
6721	Ecto.verts	Python sebae	1.6140E+04	20	1.1241E+02	White et al. 2006
6722	Ecto.verts	Python sebae	1.6140E+04	30	2.8667E+02	White et al. 2006
6723	Ecto.verts	Python sebae	1.6140E+04	34	3.9428E+02	White et al. 2006
6724	Ecto.verts	Pyxicephalus adspersus	5.6230E+02	20	1.4220E+01	White et al. 2006
6725	Ecto.verts	Rana arvalis	1.7000E+01	5	4.2330E-01	White et al. 2006
6726	Ecto.verts	Rana arvalis	1.7000E+01	10	9.5540E-01	White et al. 2006
6727	Ecto.verts	Rana arvalis	1.7000E+01	15	1.6337E+00	White et al. 2006
6728	Ecto.verts	Rana arvalis	1.7000E+01	20	2.8866E+00	White et al. 2006
6729	Ecto.verts	Rana arvalis	1.7000E+01	25	4.7685E+00	White et al. 2006
6730	Ecto.verts	Rana arvalis	1.7000E+01	30	7.6857E+00	White et al. 2006
6731	Ecto.verts	Rana aspersa	5.6300E+02	18.5	3.1669E+01	White et al. 2006
6732	Ecto.verts	Rana berlandieri	7.0000E+01	29	7.0350E+00	White et al. 2006
6733	Ecto.verts	Rana blythi	8.8700E+01	25	4.3729E+00	White et al. 2006
6734	Ecto.verts	Rana cancrivora	1.2500E+01	30	1.7500E+00	White et al. 2006
6735	Ecto.verts	Rana cancrivora	2.0450E+01	20	1.0409E+00	White et al. 2006
6736	Ecto.verts	Rana cancrivora	2.0450E+01	25	1.5767E+00	White et al. 2006
6737	Ecto.verts	Rana cancrivora	2.0450E+01	30	2.2597E+00	White et al. 2006
6738	Ecto.verts	Rana cancrivora	2.0450E+01	35	4.1043E+00	White et al. 2006
6739	Ecto.verts	Rana catesbeiana	1.4000E+01	25	2.1000E+00	White et al. 2006
6740	Ecto.verts	Rana catesbeiana	4.3550E+01	10	4.3550E-01	White et al. 2006
6741	Ecto.verts	Rana catesbeiana	4.3550E+01	20	1.6549E+00	White et al. 2006
6742	Ecto.verts	Rana catesbeiana	4.3550E+01	30	3.7889E+00	White et al. 2006
6743	Ecto.verts	Rana catesbeiana	4.7300E+01	15	2.7008E+00	White et al. 2006
6744	Ecto.verts	Rana catesbeiana	8.0900E+01	5	3.3638E+00	White et al. 2006

6745	Ecto.verts	Rana catesbeiana	1.7500E+02	22.5	7.5250E+00	White et al. 2006
6746	Ecto.verts	Rana catesbeiana	2.2820E+02	20	5.6137E+00	White et al. 2006
6747	Ecto.verts	Rana catesbeiana	2.4100E+02	20	1.7473E+01	White et al. 2006
6748	Ecto.verts	Rana catesbeiana	2.5100E+02	25	1.3052E+01	White et al. 2006
6749	Ecto.verts	Rana catesbeiana	2.6200E+02	5	1.3171E+00	White et al. 2006
6750	Ecto.verts	Rana catesbeiana	2.6900E+02	25	2.5017E+01	White et al. 2006
6751	Ecto.verts	Rana catesbeiana	2.7750E+02	20	6.9028E+00	White et al. 2006
6752	Ecto.verts	Rana catesbeiana	3.0000E+02	20	7.2000E+00	White et al. 2006
6753	Ecto.verts	Rana catesbeiana	3.0350E+02	19	1.0610E+01	White et al. 2006
6754	Ecto.verts	Rana catesbeiana	3.5000E+02	25	1.5050E+01	White et al. 2006
6755	Ecto.verts	Rana catesbeiana	5.0300E+02	21.5	1.8674E+01	White et al. 2006
6756	Ecto.verts	Rana catesbeiana	6.2100E+02	20	3.3099E+01	White et al. 2006
6757	Ecto.verts	Rana catesbeiana	6.4600E+02	5	1.0013E+01	White et al. 2006
6758	Ecto.verts	Rana chalconota	4.1000E+00	25	3.9110E-01	White et al. 2006
6759	Ecto.verts	Rana clamitans	1.9000E+01	21.5	2.1684E+00	White et al. 2006
6760	Ecto.verts	Rana clamitans	3.1000E+01	5	5.3970E-01	White et al. 2006
6761	Ecto.verts	Rana clamitans	3.2500E+01	25	2.7008E+00	White et al. 2006
6762	Ecto.verts	Rana clamitans	3.4000E+01	15	1.6085E+00	White et al. 2006
6763	Ecto.verts	Rana clamitans	3.4200E+01	14	1.7442E+00	White et al. 2006
6764	Ecto.verts	Rana cyanophlyctis	1.4200E-01	29	1.7290E-01	White et al. 2006
6765	Ecto.verts	Rana cyanophlyctis	2.9400E-01	29	2.1570E-01	White et al. 2006
6766	Ecto.verts	Rana erythraea	1.9000E+01	15	1.7860E-01	White et al. 2006
6767	Ecto.verts	Rana erythraea	1.9000E+01	20	7.8090E-01	White et al. 2006
6768	Ecto.verts	Rana erythraea	1.9000E+01	25	1.6245E+00	White et al. 2006
6769	Ecto.verts	Rana erythraea	1.9000E+01	30	2.1299E+00	White et al. 2006
6770	Ecto.verts	Rana erythraea	1.9000E+01	35	3.8703E+00	White et al. 2006

6771	Ecto.verts	Rana esculenta	1.5200E+01	23	2.5230E-01	White et al. 2006
6772	Ecto.verts	Rana esculenta	1.6400E+01	20	1.3874E+00	White et al. 2006
6773	Ecto.verts	Rana esculenta	1.7500E+01	7	2.4680E-01	White et al. 2006
6774	Ecto.verts	Rana esculenta	2.2150E+01	12	1.9100E+00	White et al. 2006
6775	Ecto.verts	Rana esculenta	2.8000E+01	15	8.1760E-01	White et al. 2006
6776	Ecto.verts	Rana esculenta	2.8400E+01	12	1.9937E+00	White et al. 2006
6777	Ecto.verts	Rana esculenta	2.8400E+01	12	2.1953E+00	White et al. 2006
6778	Ecto.verts	Rana esculenta	2.8800E+01	20	3.7037E+00	White et al. 2006
6779	Ecto.verts	Rana esculenta	3.0000E+01	18.5	6.3270E+00	White et al. 2006
6780	Ecto.verts	Rana esculenta	3.1200E+01	7	5.8870E-01	White et al. 2006
6781	Ecto.verts	Rana esculenta	3.4500E+01	24	5.9880E+00	White et al. 2006
6782	Ecto.verts	Rana esculenta	3.6000E+01	17	3.4488E+00	White et al. 2006
6783	Ecto.verts	Rana esculenta	4.5300E+01	20	4.3941E+00	White et al. 2006
6784	Ecto.verts	Rana esculenta	5.0320E+01	6	8.6170E-01	White et al. 2006
6785	Ecto.verts	Rana esculenta	5.0320E+01	25	4.1954E+00	White et al. 2006
6786	Ecto.verts	Rana esculenta	5.0320E+01	15	4.4533E+00	White et al. 2006
6787	Ecto.verts	Rana esculenta	5.7200E+01	15	2.5225E+00	White et al. 2006
6788	Ecto.verts	Rana esculenta	1.1600E+02	18.5	9.3612E+00	White et al. 2006
6789	Ecto.verts	Rana hexadactyla	3.0000E+01	29	7.3500E+00	White et al. 2006
6790	Ecto.verts	Rana hexadactyla	5.1900E+01	29	1.1989E+01	White et al. 2006
6791	Ecto.verts	Rana magna	3.4200E+01	20	1.4022E+00	White et al. 2006
6792	Ecto.verts	Rana magna	3.4200E+01	30	2.8523E+00	White et al. 2006
6793	Ecto.verts	Rana muscosa	1.3500E+01	4	9.9000E-02	White et al. 2006
6794	Ecto.verts	Rana muscosa	1.8000E+01	15	6.0610E-01	White et al. 2006
6795	Ecto.verts	Rana nicobariensis	2.6000E+00	25	2.5610E-01	White et al. 2006
6796	Ecto.verts	Rana palustris	1.4700E+01	15	9.4120E-01	White et al. 2006

6797	Ecto.verts	Rana palustris	3.6000E+01	21.5	3.6720E+00	White et al. 2006
6798	Ecto.verts	Rana pipiens	2.5000E+01	5	7.0250E-01	White et al. 2006
6799	Ecto.verts	Rana pipiens	2.5000E+01	15	1.2918E+00	White et al. 2006
6800	Ecto.verts	Rana pipiens	2.5000E+01	25	3.0280E+00	White et al. 2006
6801	Ecto.verts	Rana pipiens	2.5000E+01	24	4.0000E+00	White et al. 2006
6802	Ecto.verts	Rana pipiens	2.6500E+01	19	1.6800E+00	White et al. 2006
6803	Ecto.verts	Rana pipiens	2.7500E+01	20	2.6400E+00	White et al. 2006
6804	Ecto.verts	Rana pipiens	2.7500E+01	30	4.2900E+00	White et al. 2006
6805	Ecto.verts	Rana pipiens	3.0000E+01	23	4.6800E+00	White et al. 2006
6806	Ecto.verts	Rana pipiens	3.1800E+01	15	1.1528E+00	White et al. 2006
6807	Ecto.verts	Rana pipiens	3.2000E+01	17	2.9120E+00	White et al. 2006
6808	Ecto.verts	Rana pipiens	3.2000E+01	25	4.3520E+00	White et al. 2006
6809	Ecto.verts	Rana pipiens	3.2500E+01	15	1.6280E+00	White et al. 2006
6810	Ecto.verts	Rana pipiens	3.2500E+01	25	4.1470E+00	White et al. 2006
6811	Ecto.verts	Rana pipiens	3.3500E+01	5	5.2900E-01	White et al. 2006
6812	Ecto.verts	Rana pipiens	3.3600E+01	21.5	2.9820E+00	White et al. 2006
6813	Ecto.verts	Rana pipiens	3.4800E+01	10	4.9070E-01	White et al. 2006
6814	Ecto.verts	Rana pipiens	3.4800E+01	20	1.5208E+00	White et al. 2006
6815	Ecto.verts	Rana pipiens	3.4800E+01	30	4.6945E+00	White et al. 2006
6816	Ecto.verts	Rana pipiens	3.5000E+01	25	3.2550E+00	White et al. 2006
6817	Ecto.verts	Rana pipiens	3.5240E+01	25	3.4433E+00	White et al. 2006
6818	Ecto.verts	Rana pipiens	3.7100E+01	24	4.6900E+00	White et al. 2006
6819	Ecto.verts	Rana pipiens	3.8390E+01	10	1.6124E+00	White et al. 2006
6820	Ecto.verts	Rana pipiens	3.8390E+01	20	3.6854E+00	White et al. 2006
6821	Ecto.verts	Rana pipiens	3.8390E+01	30	5.7585E+00	White et al. 2006
6822	Ecto.verts	Rana pipiens	4.2000E+01	25	3.2760E+00	White et al. 2006

6823	Ecto.verts	Rana pipiens	4.3700E+01	15	2.8800E+00	White et al. 2006
6824	Ecto.verts	Rana pipiens	4.4300E+01	25	5.6400E+00	White et al. 2006
6825	Ecto.verts	Rana pipiens	4.9000E+01	20	3.5476E+00	White et al. 2006
6826	Ecto.verts	Rana pipiens	4.9500E+01	22	6.5046E+00	White et al. 2006
6827	Ecto.verts	Rana pipiens	5.2600E+01	22	3.4032E+00	White et al. 2006
6828	Ecto.verts	Rana ridibunda	3.5000E+01	20	1.1200E+00	White et al. 2006
6829	Ecto.verts	Rana ridibunda	3.5200E+01	20	2.3936E+01	White et al. 2006
6830	Ecto.verts	Rana ridibunda	4.7500E+01	23	4.0375E+00	White et al. 2006
6831	Ecto.verts	Rana sylvatica	6.0000E+00	25	6.4800E-01	White et al. 2006
6832	Ecto.verts	Rana sylvatica	7.4000E+00	15	7.3190E-01	White et al. 2006
6833	Ecto.verts	Rana sylvatica	8.8000E+00	14	5.8960E-01	White et al. 2006
6834	Ecto.verts	Rana sylvatica	9.0000E+00	5	4.8620E-01	White et al. 2006
6835	Ecto.verts	Rana sylvatica	1.2670E+01	20	1.0980E+00	White et al. 2006
6836	Ecto.verts	Rana sylvatica	1.2700E+01	20	1.0795E+00	White et al. 2006
6837	Ecto.verts	Rana sylvatica	1.2700E+01	20	1.0795E+00	White et al. 2006
6838	Ecto.verts	Rana temporaria	1.5800E+01	20	1.5800E+00	White et al. 2006
6839	Ecto.verts	Rana temporaria	2.1600E+01	24.6	3.4560E+00	White et al. 2006
6840	Ecto.verts	Rana temporaria	2.5000E+01	20	1.7500E+00	White et al. 2006
6841	Ecto.verts	Rana temporaria	3.0000E+01	14	3.5600E-01	White et al. 2006
6842	Ecto.verts	Rana temporaria	3.1100E+01	24.8	3.9373E+00	White et al. 2006
6843	Ecto.verts	Rana temporaria	3.2800E+01	26.3	2.0697E+00	White et al. 2006
6844	Ecto.verts	Rana temporaria	3.5000E+01	15.1	4.9000E-01	White et al. 2006
6845	Ecto.verts	Rana temporaria	3.7500E+01	20	5.8875E+00	White et al. 2006
6846	Ecto.verts	Rana temporaria	3.8000E+01	10	8.1430E-01	White et al. 2006
6847	Ecto.verts	Rana temporaria	3.8000E+01	19	1.5831E+00	White et al. 2006
6848	Ecto.verts	Rana temporaria	3.8530E+01	6	1.0066E+00	White et al. 2006

6849	Ecto.verts	Rana temporaria	3.8530E+01	15	2.6008E+00	White et al. 2006
6850	Ecto.verts	Rana temporaria	3.8530E+01	25	7.5037E+00	White et al. 2006
6851	Ecto.verts	Rana temporaria	3.9000E+01	15	2.8000E-01	White et al. 2006
6852	Ecto.verts	Rana temporaria	3.9600E+01	19.2	2.7166E+00	White et al. 2006
6853	Ecto.verts	Rana temporaria	4.2500E+01	23	4.3775E+00	White et al. 2006
6854	Ecto.verts	Rana temporaria	4.3000E+01	1	3.3712E+00	White et al. 2006
6855	Ecto.verts	Rana temporaria	4.4000E+01	9.5	1.2100E+00	White et al. 2006
6856	Ecto.verts	Rana temporaria	5.3000E+01	10.4	2.3320E+00	White et al. 2006
6857	Ecto.verts	Rana temporaria	5.3000E+01	22	6.0950E+00	White et al. 2006
6858	Ecto.verts	Rana virgatipes	7.0000E+00	5	1.4000E-01	White et al. 2006
6859	Ecto.verts	Rana virgatipes	7.0000E+00	15	3.5000E-01	White et al. 2006
6860	Ecto.verts	Rana virgatipes	7.0000E+00	25	1.0150E+00	White et al. 2006
6861	Ecto.verts	Rhinogobiops nicholsii	5.7000E-01	15	6.1029E-02	FishBase
6862	Ecto.verts	Rhinogobiops nicholsii	7.8000E-01	15	5.4584E-02	FishBase
6863	Ecto.verts	Rhinogobiops nicholsii	9.3000E-01	15	6.8334E-02	FishBase
6864	Ecto.verts	Rhinogobiops nicholsii	1.3900E+00	15	8.3653E-02	FishBase
6865	Ecto.verts	Rhinogobiops nicholsii	1.5800E+00	15	8.5136E-02	FishBase
6866	Ecto.verts	Rhinogobiops nicholsii	1.8200E+00	15	8.2785E-02	FishBase
6867	Ecto.verts	Rhinogobiops nicholsii	1.9300E+00	15	9.8593E-02	FishBase
6868	Ecto.verts	Rhinogobiops nicholsii	2.9300E+00	15	1.5993E-01	FishBase
6869	Ecto.verts	Rhinogobiops nicholsii	3.5900E+00	15	1.6832E-01	FishBase
6870	Ecto.verts	Rhinogobiops nicholsii	3.8000E+00	15	1.7551E-01	FishBase
6871	Ecto.verts	Rhinogobiops nicholsii	3.9100E+00	15	1.6691E-01	FishBase
6872	Ecto.verts	Rhinogobiops nicholsii	4.0800E+00	15	1.7987E-01	FishBase
6873	Ecto.verts	Rhyacotriton olympicus	2.6000E+00	15	2.4960E-01	White et al. 2006
6874	Ecto.verts	Salamandra maculosa	2.8320E+01	18.5	3.6448E+00	White et al. 2006

6875	Ecto.verts	Salamandra salamandra	1.0000E+01	16	4.1760E-01	White et al. 2006
6876	Ecto.verts	Salamandra salamandra	1.9100E+01	15	1.5471E+00	White et al. 2006
6877	Ecto.verts	Salamandra salamandra	2.9000E+01	16	6.2380E-01	White et al. 2006
6878	Ecto.verts	Salamandra salamandra	7.5000E+01	16	1.1273E+00	White et al. 2006
6879	Ecto.verts	Salmo salar	2.6900E+01	6	1.4307E+00	FishBase
6880	Ecto.verts	Salmo salar	2.6900E+01	18	3.9343E+00	FishBase
6881	Ecto.verts	Salmo salar	2.2700E+02	15	1.7633E+01	FishBase
6882	Ecto.verts	Salmo trutta	4.7000E+01	10	2.9272E+00	FishBase
6883	Ecto.verts	Salmo trutta	1.0000E+02	10	5.5983E+00	FishBase
6884	Ecto.verts	Salmo trutta	1.3500E+02	10	5.9517E+00	FishBase
6885	Ecto.verts	Salmo trutta	1.9000E+02	10	8.9083E+00	FishBase
6886	Ecto.verts	Salmo trutta	2.1400E+02	10	1.1980E+01	FishBase
6887	Ecto.verts	Salmo trutta	2.1600E+02	10	1.2244E+01	FishBase
6888	Ecto.verts	Salmo trutta	3.4000E+02	10	1.2372E+01	FishBase
6889	Ecto.verts	Salmo trutta	3.5000E+02	10	1.3961E+01	FishBase
6890	Ecto.verts	Salmo trutta	3.5000E+02	10	2.3023E+01	FishBase
6891	Ecto.verts	Salmo trutta	4.3000E+02	10	1.9559E+01	FishBase
6892	Ecto.verts	Salmo trutta	4.3500E+02	10	1.4916E+01	FishBase
6893	Ecto.verts	Salmo trutta	4.4300E+02	10	1.5810E+01	FishBase
6894	Ecto.verts	Salmo trutta	4.5300E+02	10	1.7118E+01	FishBase
6895	Ecto.verts	Salmo trutta	4.8500E+02	10	1.8328E+01	FishBase
6896	Ecto.verts	Salmo trutta	5.6500E+02	10	2.4118E+01	FishBase
6897	Ecto.verts	Salmo trutta	5.6500E+02	10	3.4794E+01	FishBase
6898	Ecto.verts	Salmo trutta	5.7500E+02	10	1.7302E+01	FishBase
6899	Ecto.verts	Salmo trutta	5.7500E+02	10	3.8226E+01	FishBase
6900	Ecto.verts	Salmo trutta	6.0000E+02	10	2.1833E+01	FishBase

6901	Ecto.verts	Salmo trutta	6.0500E+02	10	2.2015E+01	FishBase
6902	Ecto.verts	Salmo trutta	6.1500E+02	10	2.4101E+01	FishBase
6903	Ecto.verts	Salvadora hexalepis	6.5000E+01	20	2.9900E+00	White et al. 2006
6904	Ecto.verts	Salvadora hexalepis	6.5000E+01	30	6.9550E+00	White et al. 2006
6905	Ecto.verts	Salvelinus alpinus alpinus	4.7000E+01	2	9.5381E-01	FishBase
6906	Ecto.verts	Salvelinus alpinus alpinus	4.7000E+01	6	1.4143E+00	FishBase
6907	Ecto.verts	Salvelinus alpinus alpinus	2.1000E+02	15	1.1756E+01	FishBase
6908	Ecto.verts	Salvelinus fontinalis	5.0000E+00	5	1.8544E-01	FishBase
6909	Ecto.verts	Salvelinus fontinalis	5.0000E+00	10	3.7439E-01	FishBase
6910	Ecto.verts	Salvelinus fontinalis	5.0000E+00	15	5.3884E-01	FishBase
6911	Ecto.verts	Salvelinus fontinalis	5.0000E+00	20	7.0679E-01	FishBase
6912	Ecto.verts	Salvelinus fontinalis	5.0000E+00	5	1.7145E-01	FishBase
6913	Ecto.verts	Salvelinus fontinalis	5.0000E+00	10	3.6039E-01	FishBase
6914	Ecto.verts	Salvelinus fontinalis	5.0000E+00	15	6.0182E-01	FishBase
6915	Ecto.verts	Salvelinus fontinalis	5.0000E+00	20	6.7880E-01	FishBase
6916	Ecto.verts	Salvelinus fontinalis	1.5000E+01	5	4.8286E-01	FishBase
6917	Ecto.verts	Salvelinus fontinalis	1.5000E+01	10	9.5521E-01	FishBase
6918	Ecto.verts	Salvelinus fontinalis	1.5000E+01	15	1.3751E+00	FishBase
6919	Ecto.verts	Salvelinus fontinalis	1.5000E+01	20	1.7320E+00	FishBase
6920	Ecto.verts	Salvelinus fontinalis	4.5000E+01	5	1.2596E+00	FishBase
6921	Ecto.verts	Salvelinus fontinalis	4.5000E+01	10	2.4248E+00	FishBase
6922	Ecto.verts	Salvelinus fontinalis	4.5000E+01	15	3.4640E+00	FishBase
6923	Ecto.verts	Salvelinus fontinalis	4.5000E+01	20	4.1882E+00	FishBase
6924	Ecto.verts	Salvelinus fontinalis	4.7000E+01	10	1.6445E+00	FishBase
6925	Ecto.verts	Salvelinus fontinalis	4.7000E+01	15	3.5192E+00	FishBase
6926	Ecto.verts	Salvelinus fontinalis	4.7000E+01	20	4.7033E+00	FishBase

6927	Ecto.verts	Salvelinus fontinalis	6.4000E+01	15	4.7922E+00	FishBase
6928	Ecto.verts	Salvelinus fontinalis	1.0200E+02	10	5.6389E+00	FishBase
6929	Ecto.verts	Salvelinus fontinalis	1.1200E+02	20	1.1521E+01	FishBase
6930	Ecto.verts	Salvelinus fontinalis	1.2800E+02	15	5.1057E+00	FishBase
6931	Ecto.verts	Salvelinus fontinalis	1.3500E+02	5	3.2120E+00	FishBase
6932	Ecto.verts	Salvelinus fontinalis	1.3500E+02	10	6.1407E+00	FishBase
6933	Ecto.verts	Salvelinus fontinalis	1.3500E+02	15	8.7859E+00	FishBase
6934	Ecto.verts	Salvelinus fontinalis	1.3500E+02	20	1.0014E+01	FishBase
6935	Ecto.verts	Salvelinus fontinalis	1.3500E+02	5	3.7789E+00	FishBase
6936	Ecto.verts	Salvelinus fontinalis	1.3500E+02	10	6.9909E+00	FishBase
6937	Ecto.verts	Salvelinus fontinalis	1.3500E+02	15	9.7306E+00	FishBase
6938	Ecto.verts	Salvelinus fontinalis	1.3500E+02	20	1.1337E+01	FishBase
6939	Ecto.verts	Salvelinus fontinalis	1.5000E+02	15	9.4472E+00	FishBase
6940	Ecto.verts	Salvelinus fontinalis	1.5000E+02	15	9.5521E+00	FishBase
6941	Ecto.verts	Salvelinus fontinalis	1.8750E+02	10	1.0103E+01	FishBase
6942	Ecto.verts	Salvelinus fontinalis	4.0500E+02	5	7.9356E+00	FishBase
6943	Ecto.verts	Salvelinus fontinalis	4.0500E+02	10	1.5021E+01	FishBase
6944	Ecto.verts	Salvelinus fontinalis	4.0500E+02	15	2.1540E+01	FishBase
6945	Ecto.verts	Salvelinus fontinalis	4.0500E+02	20	2.4374E+01	FishBase
6946	Ecto.verts	Salvelinus fontinalis	4.1300E+02	10	1.6185E+01	FishBase
6947	Ecto.verts	Salvelinus fontinalis	5.1000E+02	10	1.9629E+01	FishBase
6948	Ecto.verts	Salvelinus fontinalis	6.5000E+02	10	3.2750E+01	FishBase
6949	Ecto.verts	Salvelinus fontinalis	6.9000E+02	10	1.0140E+01	FishBase
6950	Ecto.verts	Salvelinus fontinalis	6.9000E+02	10	2.0763E+01	FishBase
6951	Ecto.verts	Salvelinus fontinalis	8.0000E+02	10	4.2547E+01	FishBase
6952	Ecto.verts	Salvelinus fontinalis	8.0000E+02	10	5.0945E+01	FishBase

6953	Ecto.verts	Salvelinus fontinalis	8.1000E+02	10	2.6641E+01	FishBase
6954	Ecto.verts	Salvelinus fontinalis	8.3000E+02	10	3.3688E+01	FishBase
6955	Ecto.verts	Salvelinus fontinalis	8.5800E+02	10	3.7826E+01	FishBase
6956	Ecto.verts	Salvelinus fontinalis	8.9300E+02	10	2.8746E+01	FishBase
6957	Ecto.verts	Salvelinus fontinalis	9.4500E+02	10	4.1001E+01	FishBase
6958	Ecto.verts	Salvelinus fontinalis	9.6500E+02	10	4.7271E+01	FishBase
6959	Ecto.verts	Salvelinus fontinalis	9.8500E+02	10	3.1018E+01	FishBase
6960	Ecto.verts	Salvelinus fontinalis	1.0000E+03	5	1.7495E+01	FishBase
6961	Ecto.verts	Salvelinus fontinalis	1.0000E+03	10	3.3590E+01	FishBase
6962	Ecto.verts	Salvelinus fontinalis	1.0000E+03	15	4.5486E+01	FishBase
6963	Ecto.verts	Salvelinus fontinalis	1.0000E+03	20	5.0385E+01	FishBase
6964	Ecto.verts	Salvelinus fontinalis	1.0000E+03	5	1.8195E+01	FishBase
6965	Ecto.verts	Salvelinus fontinalis	1.0000E+03	10	3.7089E+01	FishBase
6966	Ecto.verts	Salvelinus fontinalis	1.0000E+03	15	4.8985E+01	FishBase
6967	Ecto.verts	Salvelinus fontinalis	1.0000E+03	20	5.5283E+01	FishBase
6968	Ecto.verts	Salvelinus fontinalis	1.0100E+03	10	3.3926E+01	FishBase
6969	Ecto.verts	Salvelinus fontinalis	1.1400E+03	10	3.7495E+01	FishBase
6970	Ecto.verts	Salvelinus fontinalis	1.1700E+03	10	7.9419E+01	FishBase
6971	Ecto.verts	Salvelinus fontinalis	1.2900E+03	10	5.5066E+01	FishBase
6972	Ecto.verts	Salvelinus fontinalis	1.3000E+03	10	8.0966E+01	FishBase
6973	Ecto.verts	Salvelinus fontinalis	1.3500E+03	10	6.5185E+01	FishBase
6974	Ecto.verts	Salvelinus fontinalis	1.7600E+03	10	7.6361E+01	FishBase
6975	Ecto.verts	Salvelinus namaycush	5.8000E+00	15	1.8670E-01	FishBase
6976	Ecto.verts	Salvelinus namaycush	2.7700E+01	9.1	6.2029E-01	FishBase
6977	Ecto.verts	Salvelinus namaycush	2.7700E+01	9.1	7.9475E-01	FishBase
6978	Ecto.verts	Salvelinus namaycush	2.7700E+01	9.1	8.9167E-01	FishBase

6979	Ecto.verts	Salvelinus namaycush	2.7700E+01	12.3	1.3957E+00	FishBase
6980	Ecto.verts	Salvelinus namaycush	2.7700E+01	12	1.3957E+00	FishBase
6981	Ecto.verts	Salvelinus namaycush	2.7700E+01	13.9	1.4732E+00	FishBase
6982	Ecto.verts	Salvelinus namaycush	2.7700E+01	13.9	1.5701E+00	FishBase
6983	Ecto.verts	Salvelinus namaycush	2.7700E+01	12	1.5701E+00	FishBase
6984	Ecto.verts	Salvelinus namaycush	2.7700E+01	15.9	1.7252E+00	FishBase
6985	Ecto.verts	Salvelinus namaycush	2.7700E+01	16.1	1.7252E+00	FishBase
6986	Ecto.verts	Salvelinus namaycush	2.7700E+01	13.9	1.7446E+00	FishBase
6987	Ecto.verts	Salvelinus namaycush	2.7700E+01	13.9	2.1710E+00	FishBase
6988	Ecto.verts	Salvelinus namaycush	2.7700E+01	16.1	2.3261E+00	FishBase
6989	Ecto.verts	Salvelinus namaycush	2.7700E+01	15.9	2.3261E+00	FishBase
6990	Ecto.verts	Salvelinus namaycush	2.7700E+01	20	2.3843E+00	FishBase
6991	Ecto.verts	Salvelinus namaycush	2.7700E+01	22.1	2.7138E+00	FishBase
6992	Ecto.verts	Salvelinus namaycush	2.7700E+01	22.1	2.8882E+00	FishBase
6993	Ecto.verts	Salvelinus namaycush	2.7700E+01	21.9	3.2953E+00	FishBase
6994	Ecto.verts	Salvelinus namaycush	8.2800E+01	9.2	1.3327E+00	FishBase
6995	Ecto.verts	Salvelinus namaycush	8.2800E+01	9.1	1.3327E+00	FishBase
6996	Ecto.verts	Salvelinus namaycush	8.2800E+01	10.4	1.8542E+00	FishBase
6997	Ecto.verts	Salvelinus namaycush	8.2800E+01	14.1	2.6074E+00	FishBase
6998	Ecto.verts	Salvelinus namaycush	8.2800E+01	10.7	2.6074E+00	FishBase
6999	Ecto.verts	Salvelinus namaycush	8.2800E+01	10.4	2.6654E+00	FishBase
7000	Ecto.verts	Salvelinus namaycush	8.2800E+01	16.1	3.1289E+00	FishBase
7001	Ecto.verts	Salvelinus namaycush	8.2800E+01	15.7	3.1289E+00	FishBase
7002	Ecto.verts	Salvelinus namaycush	8.2800E+01	14.1	3.1289E+00	FishBase
7003	Ecto.verts	Salvelinus namaycush	8.2800E+01	13.9	3.6504E+00	FishBase
7004	Ecto.verts	Salvelinus namaycush	8.2800E+01	16.1	4.1139E+00	FishBase

7005	Ecto.verts	Salvelinus namaycush	8.2800E+01	15.7	4.1139E+00	FishBase
7006	Ecto.verts	Salvelinus namaycush	8.2800E+01	20.1	4.8672E+00	FishBase
7007	Ecto.verts	Salvelinus namaycush	8.2800E+01	20.1	5.3307E+00	FishBase
7008	Ecto.verts	Sander lucioperca	6.0000E+02	5	1.2176E+01	FishBase
7009	Ecto.verts	Sander lucioperca	6.0000E+02	10	2.3513E+01	FishBase
7010	Ecto.verts	Sarotherodon galilaeus	2.8300E+02	26	1.3665E+01	FishBase
7011	Ecto.verts	Sauromalus hispidus	5.7400E+02	15	7.1750E+00	White et al. 2006
7012	Ecto.verts	Sauromalus hispidus	5.7400E+02	20	1.0275E+01	White et al. 2006
7013	Ecto.verts	Sauromalus hispidus	5.7400E+02	20	1.0906E+01	White et al. 2006
7014	Ecto.verts	Sauromalus hispidus	5.7400E+02	25	2.1238E+01	White et al. 2006
7015	Ecto.verts	Sauromalus hispidus	5.7400E+02	30	2.9274E+01	White et al. 2006
7016	Ecto.verts	Sauromalus hispidus	5.7400E+02	30	2.9848E+01	White et al. 2006
7017	Ecto.verts	Sauromalus hispidus	5.7400E+02	35	4.3624E+01	White et al. 2006
7018	Ecto.verts	Sauromalus hispidus	5.7400E+02	37	5.0455E+01	White et al. 2006
7019	Ecto.verts	Sauromalus hispidus	5.7400E+02	40	6.8880E+01	White et al. 2006
7020	Ecto.verts	Sauromalus obesus	1.5000E+02	20	4.3500E+00	White et al. 2006
7021	Ecto.verts	Sauromalus obesus	1.5000E+02	30	1.0800E+01	White et al. 2006
7022	Ecto.verts	Scaphiopus bombifrons	1.3300E+01	15	1.8510E+00	White et al. 2006
7023	Ecto.verts	Scaphiopus couchii	2.4600E+01	21	1.8179E+00	White et al. 2006
7024	Ecto.verts	Scaphiopus couchii	2.6900E+01	15	2.2609E+00	White et al. 2006
7025	Ecto.verts	Scaphiopus hammondii	1.5500E+00	15	2.6660E-01	White et al. 2006
7026	Ecto.verts	Scaphiopus hammondii	1.0870E+01	15	7.5000E-01	White et al. 2006
7027	Ecto.verts	Scaphiopus hammondii	1.2740E+01	21	1.1466E+00	White et al. 2006
7028	Ecto.verts	Scaphiopus hammondii	2.3600E+01	15	2.2163E+00	White et al. 2006
7029	Ecto.verts	Scaphiopus holbrooki	1.4000E+01	15	1.1712E+00	White et al. 2006
7030	Ecto.verts	Sceloporos occidentalis	1.0105E+01	40	8.1851E+01	White et al. 2006

7031	Ecto.verts	Sceloporus graciosus	5.0000E+00	25	4.4000E-01	White et al. 2006
7032	Ecto.verts	Sceloporus graciosus	5.0000E+00	30	8.2000E-01	White et al. 2006
7033	Ecto.verts	Sceloporus graciosus	5.0000E+00	37	1.7500E+00	White et al. 2006
7034	Ecto.verts	Sceloporus occidentalis	1.0105E+01	10	7.6798E-01	White et al. 2006
7035	Ecto.verts	Sceloporus occidentalis	1.0105E+01	25	1.0913E+00	White et al. 2006
7036	Ecto.verts	Sceloporus occidentalis	1.0105E+01	16	1.7229E+00	White et al. 2006
7037	Ecto.verts	Sceloporus occidentalis	1.0105E+01	31	2.2736E+00	White et al. 2006
7038	Ecto.verts	Sceloporus occidentalis	1.0105E+01	20	1.7583E+01	White et al. 2006
7039	Ecto.verts	Sceloporus occidentalis	1.0105E+01	35	2.8951E+01	White et al. 2006
7040	Ecto.verts	Sceloporus occidentalis	1.0105E+01	30	4.9515E+01	White et al. 2006
7041	Ecto.verts	Sceloporus olivaceus	1.3000E+01	30	3.9390E+00	White et al. 2006
7042	Ecto.verts	Sceloporus olivaceus	2.1000E+01	25	3.5070E+00	White et al. 2006
7043	Ecto.verts	Sceloporus olivaceus	2.4000E+01	20	1.5792E+00	White et al. 2006
7044	Ecto.verts	Sceloporus undulatus	3.8000E+00	20	1.4820E-01	White et al. 2006
7045	Ecto.verts	Sceloporus undulatus	3.8000E+00	25	4.4840E-01	White et al. 2006
7046	Ecto.verts	Sceloporus undulatus	3.8000E+00	30	1.0146E+00	White et al. 2006
7047	Ecto.verts	Sceloporus undulatus	3.8000E+00	35	1.3490E+00	White et al. 2006
7048	Ecto.verts	Sceloporus variabilis	1.2215E+01	10	2.0155E+00	White et al. 2006
7049	Ecto.verts	Sceloporus variabilis	1.2215E+01	16	3.7867E+00	White et al. 2006
7050	Ecto.verts	Sceloporus variabilis	1.2215E+01	35	2.1804E+01	White et al. 2006
7051	Ecto.verts	Scelotes gronovii	1.1000E+00	23	1.8040E-01	White et al. 2006
7052	Ecto.verts	Scelotes gronovii	1.1000E+00	33	2.4970E-01	White et al. 2006
7053	Ecto.verts	Scincella lateralis	1.0000E+00	20	1.2000E-01	White et al. 2006
7054	Ecto.verts	Scincella lateralis	1.0000E+00	30	3.1000E-01	White et al. 2006
7055	Ecto.verts	Scinus mitranus	1.4600E+01	10	3.9420E-01	White et al. 2006
7056	Ecto.verts	Scinus mitranus	1.4600E+01	15	4.8180E-01	White et al. 2006

7057	Ecto.verts	Scinus mitranus	1.4600E+01	20	7.5920E-01	White et al. 2006
7058	Ecto.verts	Scinus mitranus	1.4600E+01	25	1.5038E+00	White et al. 2006
7059	Ecto.verts	Scinus mitranus	1.4600E+01	30	1.5914E+00	White et al. 2006
7060	Ecto.verts	Scinus mitranus	1.4600E+01	35	2.1900E+00	White et al. 2006
7061	Ecto.verts	Scorpaena porcus	5.0000E+01	20	2.3093E+00	FishBase
7062	Ecto.verts	Scyliorhinus canicula	1.7500E+02	12	3.3065E+00	FishBase
7063	Ecto.verts	Scyliorhinus canicula	7.1200E+02	17	1.6442E+01	FishBase
7064	Ecto.verts	Scyliorhinus canicula	7.1200E+02	17	2.0428E+01	FishBase
7065	Ecto.verts	Scyliorhinus canicula	7.1200E+02	17	2.3418E+01	FishBase
7066	Ecto.verts	Scyliorhinus canicula	7.1200E+02	17	2.5909E+01	FishBase
7067	Ecto.verts	Scyliorhinus canicula	7.1200E+02	17	2.7902E+01	FishBase
7068	Ecto.verts	Scyliorhinus canicula	7.1200E+02	17	2.9397E+01	FishBase
7069	Ecto.verts	Scyliorhinus canicula	7.1200E+02	17	2.9397E+01	FishBase
7070	Ecto.verts	Scyliorhinus canicula	7.1200E+02	17	2.9895E+01	FishBase
7071	Ecto.verts	Scyliorhinus canicula	7.4800E+02	15	2.7742E+01	FishBase
7072	Ecto.verts	Scyliorhinus canicula	7.4800E+02	15	3.6118E+01	FishBase
7073	Ecto.verts	Scyliorhinus canicula	7.8800E+02	12	1.4337E+01	FishBase
7074	Ecto.verts	Scyliorhinus canicula	7.8800E+02	12	1.5992E+01	FishBase
7075	Ecto.verts	Scyliorhinus canicula	7.8800E+02	12	1.8197E+01	FishBase
7076	Ecto.verts	Scyliorhinus canicula	7.8800E+02	12	1.9852E+01	FishBase
7077	Ecto.verts	Scyliorhinus canicula	7.8800E+02	12	2.0403E+01	FishBase
7078	Ecto.verts	Scyliorhinus canicula	8.5700E+02	7	1.3194E+01	FishBase
7079	Ecto.verts	Scyliorhinus canicula	8.5700E+02	7	1.4993E+01	FishBase
7080	Ecto.verts	Scyliorhinus canicula	8.5700E+02	7	1.6792E+01	FishBase
7081	Ecto.verts	Scyliorhinus canicula	8.5700E+02	7	1.6792E+01	FishBase
7082	Ecto.verts	Scyliorhinus canicula	8.5700E+02	7	1.7392E+01	FishBase

7083	Ecto.verts	Scyliorhinus canicula	8.5700E+02	7	1.8591E+01	FishBase
7084	Ecto.verts	Scyliorhinus stellaris	1.7350E+03	17.5	5.8279E+01	FishBase
7085	Ecto.verts	Scyliorhinus stellaris	1.8600E+03	17	7.0287E+01	FishBase
7086	Ecto.verts	Scyliorhinus stellaris	2.3000E+03	16	1.7705E+01	FishBase
7087	Ecto.verts	Scyliorhinus stellaris	2.3000E+03	16	4.0238E+01	FishBase
7088	Ecto.verts	Scyliorhinus stellaris	2.3000E+03	16	4.5066E+01	FishBase
7089	Ecto.verts	Scyliorhinus stellaris	2.5300E+03	18.3	7.0819E+00	FishBase
7090	Ecto.verts	Scyliorhinus stellaris	2.8000E+03	19	9.7971E+00	FishBase
7091	Ecto.verts	Sebastes diploproa	2.0000E+00	10	1.6235E-01	FishBase
7092	Ecto.verts	Sebastes diploproa	2.0000E+00	10	1.6375E-01	FishBase
7093	Ecto.verts	Sebastes diploproa	2.0000E+00	10	2.0574E-01	FishBase
7094	Ecto.verts	Sebastes diploproa	2.0000E+00	15	2.2113E-01	FishBase
7095	Ecto.verts	Sebastes diploproa	2.0000E+00	15	2.2253E-01	FishBase
7096	Ecto.verts	Sebastes diploproa	2.0000E+00	20	2.3513E-01	FishBase
7097	Ecto.verts	Sebastes diploproa	2.0000E+00	15	2.5472E-01	FishBase
7098	Ecto.verts	Sebastes diploproa	2.0000E+00	20	2.9671E-01	FishBase
7099	Ecto.verts	Sebastes diploproa	2.0000E+00	20	3.5409E-01	FishBase
7100	Ecto.verts	Sebastolobus altivelis	1.7000E+00	5.7	5.7103E-02	FishBase
7101	Ecto.verts	Sebastolobus altivelis	1.9000E+00	5.7	4.9195E-02	FishBase
7102	Ecto.verts	Sebastolobus altivelis	1.2900E+02	5.7	3.6109E-01	FishBase
7103	Ecto.verts	Sebastolobus altivelis	1.3900E+02	5.7	4.8635E-01	FishBase
7104	Ecto.verts	Sebastolobus altivelis	1.5200E+02	5.7	4.2547E-01	FishBase
7105	Ecto.verts	Sebastolobus altivelis	1.9800E+02	5.7	4.1568E-01	FishBase
7106	Ecto.verts	Seriola quinqueradiata	9.8900E+02	19.2	1.0243E+02	FishBase
7107	Ecto.verts	Sibynomorphus mikanii	1.1000E+01	20	1.3530E+00	White et al. 2006
7108	Ecto.verts	Siren intermedia	7.0000E+00	25	2.8000E-01	White et al. 2006

7109	Ecto.verts	Siren intermedia	1.3700E+01	25	4.5210E-01	White et al. 2006
7110	Ecto.verts	Siren intermedia	1.8100E+01	25	8.6610E-01	White et al. 2006
7111	Ecto.verts	Siren intermedia	7.4900E+01	25	1.0763E+01	White et al. 2006
7112	Ecto.verts	Siren lacertina	3.6000E+01	25	1.2679E+00	White et al. 2006
7113	Ecto.verts	Siren lacertina	7.3000E+01	25	1.0800E+00	White et al. 2006
7114	Ecto.verts	Siren lacertina	1.2600E+02	22	6.6654E+00	White et al. 2006
7115	Ecto.verts	Siren lacertina	1.7200E+02	20	4.6268E+00	White et al. 2006
7116	Ecto.verts	Siren lacertina	2.6900E+02	25	3.0397E+00	White et al. 2006
7117	Ecto.verts	Siren lacertina	5.8100E+02	15	3.8114E+00	White et al. 2006
7118	Ecto.verts	Siren lacertina	6.0100E+02	5	2.0844E+00	White et al. 2006
7119	Ecto.verts	Siren lacertina	6.2800E+02	25	9.0118E+00	White et al. 2006
7120	Ecto.verts	Siren lacertina	1.1820E+03	25	3.2174E+01	White et al. 2006
7121	Ecto.verts	Smilisca baudinii	2.3700E+01	15	1.5832E+00	White et al. 2006
7122	Ecto.verts	Smilisca fodiens	1.5100E+01	20	4.8320E-01	White et al. 2006
7123	Ecto.verts	Smilisca fodiens	1.5130E+01	20	5.1400E-01	White et al. 2006
7124	Ecto.verts	Spalerosophis cliffordi	3.0000E+02	15	1.5600E+01	White et al. 2006
7125	Ecto.verts	Spalerosophis cliffordi	3.0000E+02	20	2.1600E+01	White et al. 2006
7126	Ecto.verts	Spalerosophis cliffordi	3.0000E+02	25	2.9700E+01	White et al. 2006
7127	Ecto.verts	Spalerosophis cliffordi	3.0000E+02	30	4.0500E+01	White et al. 2006
7128	Ecto.verts	Spalerosophis cliffordi	3.0000E+02	35	5.7000E+01	White et al. 2006
7129	Ecto.verts	Spalerosophis cliffordi	3.0000E+02	37	7.6500E+01	White et al. 2006
7130	Ecto.verts	Spalerosophis cliffordi	3.0000E+02	40	1.2750E+02	White et al. 2006
7131	Ecto.verts	Spalerosophis cliffordi	3.0000E+02	41.8	1.7100E+02	White et al. 2006
7132	Ecto.verts	Spalerosophis cliffordi	3.5100E+02	30	4.7736E+01	White et al. 2006
7133	Ecto.verts	Sphaerodactylus beattyi	4.0000E-01	25	6.0000E-02	White et al. 2006
7134	Ecto.verts	Sphaerodactylus beattyi	4.0000E-01	30	9.0000E-02	White et al. 2006

7135	Ecto.verts	Sphaerodactylus cinereus	4.0000E-01	27	4.0000E-02	White et al. 2006
7136	Ecto.verts	Sphaerodactylus macrolepis	5.0000E-01	30	1.3000E-01	White et al. 2006
7137	Ecto.verts	Sphenodon punctatum	4.3000E+02	21	9.4600E+00	White et al. 2006
7138	Ecto.verts	Sphenodon punctatum	4.3000E+02	25	1.3330E+01	White et al. 2006
7139	Ecto.verts	Sphenops sepsoides	7.4000E+00	30	5.8460E-01	White et al. 2006
7140	Ecto.verts	Squalus acanthias	1.0000E+02	13	1.0147E+01	FishBase
7141	Ecto.verts	Squalus acanthias	6.4000E+02	13	2.2841E+01	FishBase
7142	Ecto.verts	Squalus acanthias	9.4000E+02	13	3.5521E+01	FishBase
7143	Ecto.verts	Squalus acanthias	1.0500E+03	13	4.4087E+01	FishBase
7144	Ecto.verts	Squalus acanthias	1.1200E+03	13	4.9377E+01	FishBase
7145	Ecto.verts	Squalus acanthias	1.2400E+03	13	4.4255E+01	FishBase
7146	Ecto.verts	Squalus acanthias	1.5500E+03	13	5.0980E+01	FishBase
7147	Ecto.verts	Squalus acanthias	1.6150E+03	9	2.4864E+01	FishBase
7148	Ecto.verts	Squalus acanthias	1.7500E+03	13	6.7355E+01	FishBase
7149	Ecto.verts	Squalus acanthias	1.8120E+03	9	2.6628E+01	FishBase
7150	Ecto.verts	Squalus acanthias	1.8540E+03	10	4.1517E+01	FishBase
7151	Ecto.verts	Squalus acanthias	1.8700E+03	13	7.0665E+01	FishBase
7152	Ecto.verts	Squalus acanthias	2.7000E+03	13	8.6914E+01	FishBase
7153	Ecto.verts	Squalus acanthias	3.3000E+03	13	1.1777E+02	FishBase
7154	Ecto.verts	Squalus acanthias	3.5000E+03	11	1.4451E+02	FishBase
7155	Ecto.verts	Squalus acanthias	4.6000E+03	13	1.4808E+02	FishBase
7156	Ecto.verts	Squalus acanthias	5.7600E+03	13	1.9751E+02	FishBase
7157	Ecto.verts	Squalus acanthias	7.0400E+03	13	2.3155E+02	FishBase
7158	Ecto.verts	Squalus acanthias	7.8500E+03	13	2.1424E+02	FishBase
7159	Ecto.verts	Squalus acanthias	8.9700E+03	13	1.5693E+02	FishBase
7160	Ecto.verts	Storeria dekayi	7.2000E+00	20	6.3000E-01	White et al. 2006

7161	Ecto.verts	Storeria dekayi	7.2000E+00	30	1.1664E+00	White et al. 2006
7162	Ecto.verts	Tarentola mauritanica	6.6000E+00	20	4.4220E-01	White et al. 2006
7163	Ecto.verts	Tarentola mauritanica	6.6000E+00	30	8.3160E-01	White et al. 2006
7164	Ecto.verts	Tarentola mauritanica	6.6000E+00	35	1.2012E+00	White et al. 2006
7165	Ecto.verts	Taricha granulosa	6.5600E+00	15	1.6010E-01	White et al. 2006
7166	Ecto.verts	Taricha granulosa	8.7500E+00	20	3.9200E-01	White et al. 2006
7167	Ecto.verts	Taricha granulosa	9.0000E+00	5	1.1520E-01	White et al. 2006
7168	Ecto.verts	Taricha granulosa	1.0000E+01	5	4.0000E-01	White et al. 2006
7169	Ecto.verts	Taricha granulosa	1.0000E+01	10	6.0000E-01	White et al. 2006
7170	Ecto.verts	Taricha granulosa	1.0000E+01	15	7.6800E-01	White et al. 2006
7171	Ecto.verts	Taricha granulosa	1.0000E+01	20	1.0040E+00	White et al. 2006
7172	Ecto.verts	Taricha granulosa	1.0000E+01	25	1.4000E+00	White et al. 2006
7173	Ecto.verts	Taricha granulosa	1.0100E+01	20	1.0262E+00	White et al. 2006
7174	Ecto.verts	Taricha granulosa	1.1900E+01	25	1.6398E+00	White et al. 2006
7175	Ecto.verts	Taricha granulosa	1.2500E+01	10	7.4500E-01	White et al. 2006
7176	Ecto.verts	Taricha granulosa	1.3300E+01	5	5.4130E-01	White et al. 2006
7177	Ecto.verts	Taricha granulosa	1.3800E+01	15	1.0930E+00	White et al. 2006
7178	Ecto.verts	Taricha rivularis	1.0830E+01	15	3.0110E-01	White et al. 2006
7179	Ecto.verts	Taricha torosa	6.9400E+00	15	2.0890E-01	White et al. 2006
7180	Ecto.verts	Taricha torosa	9.7000E+00	15	2.4250E-01	White et al. 2006
7181	Ecto.verts	Taricha torosa	9.7000E+00	15	5.2380E-01	White et al. 2006
7182	Ecto.verts	Taricha torosa	9.8700E+00	10	8.8800E-02	White et al. 2006
7183	Ecto.verts	Taricha torosa	9.8700E+00	15	2.5660E-01	White et al. 2006
7184	Ecto.verts	Taricha torosa	9.8700E+00	20	2.6650E-01	White et al. 2006
7185	Ecto.verts	Taricha torosa	9.8700E+00	25	4.2440E-01	White et al. 2006
7186	Ecto.verts	Taricha torosa	1.0670E+01	15	2.8600E-01	White et al. 2006

7187	Ecto.verts	Taricha torosa	1.0670E+01	25	5.4520E-01	White et al. 2006
7188	Ecto.verts	Taricha torosa	1.1000E+01	25	5.5550E-01	White et al. 2006
7189	Ecto.verts	Taricha torosa	1.1230E+01	10	1.7970E-01	White et al. 2006
7190	Ecto.verts	Taricha torosa	1.1230E+01	15	6.0640E-01	White et al. 2006
7191	Ecto.verts	Taricha torosa	1.1230E+01	20	6.8500E-01	White et al. 2006
7192	Ecto.verts	Taricha torosa	1.1230E+01	25	1.3364E+00	White et al. 2006
7193	Ecto.verts	Taricha torosa	1.2500E+01	25	1.0738E+00	White et al. 2006
7194	Ecto.verts	Taricha torosa	1.9700E+01	17	1.3514E+00	White et al. 2006
7195	Ecto.verts	Telmatobius culeus	1.2230E+02	10	1.7244E+00	White et al. 2006
7196	Ecto.verts	Telmatobius marmoratus	1.8400E+01	10	9.2000E-01	White et al. 2006
7197	Ecto.verts	Terrapene ornata ornata	3.5400E+02	10	2.8320E-01	White et al. 2006
7198	Ecto.verts	Terrapene ornata ornata	3.5400E+02	20	3.2922E+00	White et al. 2006
7199	Ecto.verts	Terrapene ornata ornata	3.5400E+02	30	5.2746E+00	White et al. 2006
7200	Ecto.verts	Terrapene ornata ornata	3.5400E+02	40	3.2285E+01	White et al. 2006
7201	Ecto.verts	Thamnodyastes strigatus	5.5000E+01	20	2.2550E+00	White et al. 2006
7202	Ecto.verts	Thamnophis butleri	1.9020E+01	25	1.1707E+00	White et al. 2006
7203	Ecto.verts	Thamnophis proximus	3.1000E+01	20	2.3250E+00	White et al. 2006
7204	Ecto.verts	Thamnophis proximus	3.1000E+01	30	4.6500E+00	White et al. 2006
7205	Ecto.verts	Thamnophis sirtalis	4.3000E+01	20	2.3650E+00	White et al. 2006
7206	Ecto.verts	Thamnophis sirtalis	2.0000E+02	29	6.6000E+00	White et al. 2006
7207	Ecto.verts	Thamnophis sirtalis parietalis	2.5000E+01	5	4.2500E-01	White et al. 2006
7208	Ecto.verts	Thamnophis sirtalis parietalis	2.5000E+01	10	1.2750E+00	White et al. 2006
7209	Ecto.verts	Thamnophis sirtalis parietalis	2.5000E+01	15	1.2750E+00	White et al. 2006
7210	Ecto.verts	Thamnophis sirtalis parietalis	2.5000E+01	20	1.8500E+00	White et al. 2006
7211	Ecto.verts	Thamnophis sirtalis parietalis	2.5000E+01	25	2.7500E+00	White et al. 2006
7212	Ecto.verts	Thamnophis sirtalis parietalis	2.5000E+01	30	4.0000E+00	White et al. 2006

7213	Ecto.verts	Thamnophis sirtalis sirtalis	3.7500E+01	10	7.3125E-01	White et al. 2006
7214	Ecto.verts	Thamnophis sirtalis sirtalis	3.7500E+01	15	1.0500E+00	White et al. 2006
7215	Ecto.verts	Thamnophis sirtalis sirtalis	3.7500E+01	20	1.5000E+00	White et al. 2006
7216	Ecto.verts	Thamnophis sirtalis sirtalis	3.7500E+01	25	2.5875E+00	White et al. 2006
7217	Ecto.verts	Thamnophis sirtalis sirtalis	3.7500E+01	30	3.6000E+00	White et al. 2006
7218	Ecto.verts	Thorius sp.	2.5000E-01	25	2.1700E-02	White et al. 2006
7219	Ecto.verts	Thorius sp.	2.6000E-01	5	4.1000E-03	White et al. 2006
7220	Ecto.verts	Thorius sp.	3.1000E-01	15	8.0000E-03	White et al. 2006
7221	Ecto.verts	Thymallus arcticus	2.8300E+02	4	1.3467E+01	FishBase
7222	Ecto.verts	Thymallus arcticus	2.8300E+02	8	1.4853E+01	FishBase
7223	Ecto.verts	Thymallus arcticus	2.8300E+02	10	1.8022E+01	FishBase
7224	Ecto.verts	Thymallus arcticus	2.8300E+02	12	2.2775E+01	FishBase
7225	Ecto.verts	Tilapia zillii	3.1500E+02	26	1.3006E+01	FishBase
7226	Ecto.verts	Tiliqua rugosa	5.0860E+02	35	7.3238E+01	White et al. 2006
7227	Ecto.verts	Tiliqua rugosus	4.6100E+02	20	1.3830E+01	White et al. 2006
7228	Ecto.verts	Tiliqua rugosus	4.6100E+02	30	4.1490E+01	White et al. 2006
7229	Ecto.verts	Tiliqua rugosus	4.6100E+02	37	6.4540E+01	White et al. 2006
7230	Ecto.verts	Tiliqua scincoides	4.9300E+02	20	1.3311E+01	White et al. 2006
7231	Ecto.verts	Tiliqua scincoides	4.9300E+02	30	4.4370E+01	White et al. 2006
7232	Ecto.verts	Torpedo marmorata	4.4800E+02	16	6.8971E+00	FishBase
7233	Ecto.verts	Torpedo marmorata	8.0300E+02	16	1.6296E+01	FishBase
7234	Ecto.verts	Torpedo marmorata	3.3000E+03	16	6.4661E+01	FishBase
7235	Ecto.verts	Trematomus hansoni	4.7000E+01	3	1.0854E+00	FishBase
7236	Ecto.verts	Triturus cristatus	7.0000E+00	10	3.8500E-01	White et al. 2006
7237	Ecto.verts	Triturus vulgaris	8.7500E+00	15	8.1270E-01	White et al. 2006
7238	Ecto.verts	Triturus vulgaris	8.7500E+00	6	8.6300E-01	White et al. 2006

7239	Ecto.verts	Triturus vulgaris	8.7500E+00	25	1.6264E+00	White et al. 2006
7240	Ecto.verts	Trogonophis weigmanni	4.9850E+00	25	1.9217E-01	White et al. 2006
7241	Ecto.verts	Typhlogobius californiensis	4.3000E-01	15	9.0273E-03	FishBase
7242	Ecto.verts	Typhlogobius californiensis	4.4000E-01	15	8.3135E-03	FishBase
7243	Ecto.verts	Typhlogobius californiensis	6.1000E-01	15	1.1099E-02	FishBase
7244	Ecto.verts	Typhlogobius californiensis	6.7000E-01	15	1.4066E-02	FishBase
7245	Ecto.verts	Typhlogobius californiensis	1.1700E+00	15	1.4738E-02	FishBase
7246	Ecto.verts	Typhlogobius californiensis	1.1700E+00	15	1.7194E-02	FishBase
7247	Ecto.verts	Typhlogobius californiensis	1.1800E+00	15	1.9818E-02	FishBase
7248	Ecto.verts	Typhlogobius californiensis	1.3700E+00	15	2.0133E-02	FishBase
7249	Ecto.verts	Typhlogobius californiensis	1.5800E+00	15	1.8796E-02	FishBase
7250	Ecto.verts	Typhlogobius californiensis	1.7200E+00	15	2.5276E-02	FishBase
7251	Ecto.verts	Typhlogobius californiensis	1.8500E+00	15	2.2008E-02	FishBase
7252	Ecto.verts	Typhlogobius californiensis	1.8800E+00	15	2.1050E-02	FishBase
7253	Ecto.verts	Typhlogobius californiensis	2.0200E+00	15	2.5444E-02	FishBase
7254	Ecto.verts	Typhlogobius californiensis	2.1100E+00	15	2.3625E-02	FishBase
7255	Ecto.verts	Typhlogobius californiensis	2.5000E+00	15	2.2743E-02	FishBase
7256	Ecto.verts	Typhlogobius californiensis	3.1500E+00	15	2.8656E-02	FishBase
7257	Ecto.verts	Typhlogobius californiensis	3.3400E+00	15	3.2722E-02	FishBase
7258	Ecto.verts	Typhlonectes compressicauda	3.0620E+01	25	1.4420E+00	White et al. 2006
7259	Ecto.verts	Typhlonectes compressicauda	6.4000E+01	25	9.5460E+00	White et al. 2006
7260	Ecto.verts	Typhlonectes compressicauda	1.3500E+02	20	4.5200E+00	White et al. 2006
7261	Ecto.verts	Typhlonectes compressicauda	1.3800E+02	25	1.1253E+01	White et al. 2006
7262	Ecto.verts	Uromastyx microlepis	2.4794E+02	25	7.4283E+00	White et al. 2006
7263	Ecto.verts	Uromastyx microlepis	2.4815E+02	35	1.7435E+01	White et al. 2006
7264	Ecto.verts	Uromastyx microlepis	2.6904E+02	30	1.3156E+01	White et al. 2006

7265	Ecto.verts	Uromastyx microlepis	2.8984E+02	20	3.5071E+00	White et al. 2006
7266	Ecto.verts	Uromastyx microlepis	2.9155E+02	40	3.2537E+01	White et al. 2006
7267	Ecto.verts	Uta mearnsi	1.4000E+01	20	5.4600E-01	White et al. 2006
7268	Ecto.verts	Uta mearnsi	1.4000E+01	30	2.3800E+00	White et al. 2006
7269	Ecto.verts	Uta mearnsi	1.4000E+01	37	3.5000E+00	White et al. 2006
7270	Ecto.verts	Uta stansburiana	3.6250E+00	37	1.0875E+00	White et al. 2006
7271	Ecto.verts	Uta stansburiana	3.6250E+00	20	2.8638E+00	White et al. 2006
7272	Ecto.verts	Uta stansburiana	3.6250E+00	30	4.9481E+00	White et al. 2006
7273	Ecto.verts	Uta stansburiana	3.6250E+00	35	1.0368E+01	White et al. 2006
7274	Ecto.verts	Uta stansburiana	3.6250E+00	40	1.7581E+01	White et al. 2006
7275	Ecto.verts	Varanus albigularis	9.6300E+02	35	1.4927E+02	White et al. 2006
7276	Ecto.verts	Varanus bengalensis	3.4400E+03	20	9.2880E+01	White et al. 2006
7277	Ecto.verts	Varanus bengalensis	3.4400E+03	30	1.7544E+02	White et al. 2006
7278	Ecto.verts	Varanus exanthematicus	3.8360E+03	25	1.8936E+03	White et al. 2006
7279	Ecto.verts	Varanus exanthematicus	3.8360E+03	30	2.9695E+03	White et al. 2006
7280	Ecto.verts	Varanus exanthematicus	3.8360E+03	35	6.7294E+03	White et al. 2006
7281	Ecto.verts	Varanus giganteus	2.4960E+03	25.9	1.0708E+02	White et al. 2006
7282	Ecto.verts	Varanus giganteus	2.5020E+03	34.7	2.3844E+02	White et al. 2006
7283	Ecto.verts	Varanus gilleni	2.7500E+01	37	5.3625E+00	White et al. 2006
7284	Ecto.verts	Varanus gouldi	6.7400E+02	20	1.6176E+01	White et al. 2006
7285	Ecto.verts	Varanus gouldi	6.7400E+02	30	5.1898E+01	White et al. 2006
7286	Ecto.verts	Varanus gouldi	6.7400E+02	37	7.5488E+01	White et al. 2006
7287	Ecto.verts	Varanus gouldii	7.6900E+01	19.7	2.9376E+00	White et al. 2006
7288	Ecto.verts	Varanus gouldii	1.5460E+02	35.5	1.9047E+01	White et al. 2006
7289	Ecto.verts	Varanus gouldii	1.6900E+02	30.7	1.1458E+01	White et al. 2006
7290	Ecto.verts	Varanus gouldii	1.7910E+02	40	3.0035E+01	White et al. 2006

7291	Ecto.verts	Varanus gouldii	6.7400E+02	15	6.0660E+00	White et al. 2006
7292	Ecto.verts	Varanus gouldii	6.7400E+02	20	1.8198E+01	White et al. 2006
7293	Ecto.verts	Varanus gouldii	6.7400E+02	25	3.5722E+01	White et al. 2006
7294	Ecto.verts	Varanus gouldii	6.7400E+02	30	5.1898E+01	White et al. 2006
7295	Ecto.verts	Varanus gouldii	6.7400E+02	35	6.7400E+01	White et al. 2006
7296	Ecto.verts	Varanus gouldii	6.7400E+02	40	8.0880E+01	White et al. 2006
7297	Ecto.verts	Varanus gouldii	1.0860E+03	35	9.7740E+01	White et al. 2006
7298	Ecto.verts	Varanus mertensi	9.0400E+02	35	7.0512E+01	White et al. 2006
7299	Ecto.verts	Varanus panoptes	7.9900E+02	40	1.6435E+02	White et al. 2006
7300	Ecto.verts	Varanus panoptes	9.3100E+02	35	1.2289E+02	White et al. 2006
7301	Ecto.verts	Varanus panoptes	1.4270E+03	35.3	1.7124E+02	White et al. 2006
7302	Ecto.verts	Varanus panoptes	2.0030E+03	30.6	1.7807E+02	White et al. 2006
7303	Ecto.verts	Varanus panoptes	2.0050E+03	20.5	5.4937E+01	White et al. 2006
7304	Ecto.verts	Varanus varius	4.4100E+03	30	2.2050E+02	White et al. 2006
7305	Ecto.verts	Varnus rosenbergi	1.2693E+03	35	1.6755E+02	White et al. 2006
7306	Ecto.verts	Vipera berus	6.3000E+01	25	5.6700E+00	White et al. 2006
7307	Ecto.verts	Xantusia henshawi	3.5000E+00	15	1.2950E-01	White et al. 2006
7308	Ecto.verts	Xantusia henshawi	3.5000E+00	20	2.4850E-01	White et al. 2006
7309	Ecto.verts	Xantusia henshawi	3.5000E+00	25	3.6750E-01	White et al. 2006
7310	Ecto.verts	Xantusia henshawi	3.5000E+00	30	5.1800E-01	White et al. 2006
7311	Ecto.verts	Xantusia riversiana	1.9000E+01	35	3.8000E-01	White et al. 2006
7312	Ecto.verts	Xantusia riversiana	1.9000E+01	20	6.6500E-01	White et al. 2006
7313	Ecto.verts	Xantusia riversiana	1.9000E+01	25	1.1020E+00	White et al. 2006
7314	Ecto.verts	Xantusia riversiana	1.9000E+01	30	1.9950E+00	White et al. 2006
7315	Ecto.verts	Xantusia vigilis	1.5000E+00	25	1.6800E-01	White et al. 2006
7316	Ecto.verts	Xantusia vigilis	1.5000E+00	30	2.5890E-01	White et al. 2006

7317	Ecto.verts	Xenodon guentheri	5.0000E+01	20	1.7500E+00	White et al. 2006
7318	Ecto.verts	Xenodon merremii	5.0200E+02	20	2.1586E+01	White et al. 2006
7319	Ecto.verts	Xenodon neuwiedii	5.3000E+01	20	1.9610E+00	White et al. 2006
7320	Ecto.verts	Xenopus laevis	2.2100E+01	18	1.7901E+00	White et al. 2006
7321	Ecto.verts	Xenopus laevis	2.3900E+01	25	3.8240E+00	White et al. 2006
7322	Ecto.verts	Xenopus laevis	2.8390E+01	25	1.6063E+00	White et al. 2006
7323	Ecto.verts	Xenopus laevis	2.9300E+01	25	4.2000E+00	White et al. 2006
7324	Ecto.verts	Xenopus laevis	3.0030E+01	15	1.6847E+00	White et al. 2006
7325	Ecto.verts	Xenopus laevis	3.3300E+01	15	1.9106E+00	White et al. 2006
7326	Ecto.verts	Xenopus laevis	3.3300E+01	25	3.2218E+00	White et al. 2006
7327	Ecto.verts	Xenopus laevis	4.1000E+01	25	3.5260E+00	White et al. 2006
7328	Ecto.verts	Xenopus laevis	5.5000E+01	20	2.5300E+00	White et al. 2006
7329	Ecto.verts	Xenopus laevis	6.3600E+01	15	2.1516E+00	White et al. 2006
7330	Ecto.verts	Xenopus laevis	6.7000E+01	17	4.2277E+00	White et al. 2006
7331	Ecto.verts	Xenopus laevis	6.7300E+01	17.3	3.8340E+00	White et al. 2006
7332	Ecto.verts	Xenopus laevis	1.0000E+02	20	4.5000E+00	White et al. 2006
7333	Ecto.verts	Xenopus laevis	1.2200E+02	22	5.5998E+00	White et al. 2006
7334	Ecto.verts	Xenopus laevis	1.3900E+02	20	3.8364E+00	White et al. 2006
7335	Ecto.verts	Xenopus mulleri	2.4000E+01	17	2.5104E+00	White et al. 2006
